# Synopsis of the pelidnotine scarabs (Coleoptera, Scarabaeidae, Rutelinae, Rutelini) and annotated catalog of the species and subspecies

**DOI:** 10.3897/zookeys.666.9191

**Published:** 2017-04-06

**Authors:** Matthew R. Moore, Mary L. Jameson, Beulah H. Garner, Cédric Audibert, Andrew B. T. Smith, Matthias Seidel

**Affiliations:** 1 Department of Entomology and Nematology, University of Florida Building 1881 Natural Drive Area, Steinmetz Hall, Box 110620, Gainesville, FL 32611-0620, USA; 2 Department of Biological Sciences, Wichita State University 1845 Fairmount, Box 26, Wichita, KS 67260-0026, USA; 3 Natural History Museum, Insects Division, Department of Life Sciences, Cromwell Road, London SW7 5BD, UK; 4 Musée des Confluences, Centre de Conservation et d’Etude des Collections, 13A Rue Bancel, F-69007 Lyon, France; 5 Research Division, Canadian Museum of Nature, P.O. Box 3443, Station D, Ottawa, Ontario, K1P 6P4, Canada; 6 Department of Zoology, Faculty of Science, Charles University, Viničná 7, CZ-12383 Praha 2, Czech Republic; 7 Department of Entomology, National Museum, Cirkusová 1740, CZ-19300 Praha 9, Czech Republic

**Keywords:** leaf chafers, jewel beetles, New World, taxonomy

## Abstract

The pelidnotine scarabs (Scarabaeidae: Rutelinae: Rutelini) are a speciose, paraphyletic assemblage of beetles that includes spectacular metallic species (“jewel scarabs”) as well as species that are ecologically important as herbivores, pollinators, and bioindicators. These beetles suffer from a complicated nomenclatural history, due primarily to 20^th^ century taxonomic and nomenclatural errors. We review the taxonomic history of the pelidnotine scarabs, present a provisional key to genera with overviews of all genera, and synthesize a catalog of all taxa with synonyms, distributional data, type specimen information, and 107 images of exemplar species. As a result of our research, the pelidnotine leaf chafers (a paraphyletic group) include 27 (26 extant and 1 extinct) genera and 420 valid species and subspecies (419 extant and 1 extinct). Our research makes biodiversity research on this group tractable and accessible, thus setting the stage for future studies that address evolutionary and ecological trends. Based on our research, 1 new species is described, 1 new generic synonym and 12 new species synonyms are proposed, 11 new lectotypes and 1 new neotype are designated, many new or revised nomenclatural combinations, and many unavailable names are presented. The following taxonomic changes are made:

New generic synonym: The genus *Heteropelidnota* Ohaus, 1912 is a **new junior synonym** of *Pelidnota* MacLeay, 1819.

New species synonyms: *Plusiotis adelaida pavonacea* Casey, 1915 is a **syn. n.** of *Chrysina adelaida* (Hope, 1841); *Odontognathus gounellei* Ohaus, 1908 is a **revised synonym** of *Pelidnota ebenina* (Blanchard, 1842); *Pelidnota francoisgenieri* Moore & Jameson, 2013 is a **syn. n.** of *Pelidnota punctata* (Linnaeus, 1758); *Pelidnota genieri* Soula, 2009 is a **syn. n.** of *Pelidnota punctata* (Linnaeus, 1758); *Pelidnota lutea* (Olivier, 1758) is a **revised synonym** of *Pelidnota punctata* (Linnaeus, 1758); Pelidnota (Pelidnota) texensis Casey, 1915 is a **revised synonym** of *Pelidnota punctata* (Linnaeus, 1758); Pelidnota (Strigidia) zikani (Ohaus, 1922) is a **revised synonym** of *Pelidnota tibialis tibialis* Burmeister, 1844; *Pelidnota ludovici* Ohaus, 1905 is a **syn. n.** of *Pelidnota burmeisteri tricolor* Nonfried, 1894; *Rutela fulvipennis* Germar, 1824 is **syn. n.** of *Pelidnota cuprea* (Germar, 1824); *Pelidnota pulchella blanda* Burmeister, 1844 is a **syn. n.** of *Pelidnota pulchella pulchella* (Kirby, 1819); *Pelidnota pulchella scapularis* Burmeister, 1844 is a **syn. n.** of *Pelidnota pulchella pulchella* (Kirby, 1819); *Pelidnota xanthogramma* Perty, 1830 is a **syn. n.** of *Pelidnota pulchella pulchella* (Kirby, 1819).

New or revised statuses: *Pelidnota fabricelavalettei* Soula, 2009, **revised status**, is considered a species; *Pelidnota rioensis* Soula, 2009, **stat. n.**, is considered a species; *Pelidnota semiaurata semiaurata* Burmeister, 1844, **stat. rev.**, is considered a subspecies.

New or comb. rev. and revised status: *Plusiotis guaymi* Curoe, 2001 is formally transferred to the genus *Chrysina* (*C. guaymi* (Curoe, 2001), **comb. n.**); *Plusiotis transvolcanica* Morón & Nogueira, 2016 is transferred to the genus *Chrysina* (*C. transvolcanica* (Morón & Nogueira, 2016), **comb. n.**). *Heteropelidnota kuhnti* Ohaus, 1912 is transferred to the genus *Pelidnota* (*P. kuhnti* (Ohaus, 1912), **comb. n.**); *Odontognathus riedeli* Ohaus, 1905 is considered a subspecies of *Pelidnota rubripennis* Burmeister, 1844 (*Pelidnota rubripennis riedeli* (Ohaus, 1905), **revised status** and **comb. rev.)**; Pelidnota (Strigidia) acutipennis (F. Bates, 1904) is transferred to the genus *Sorocha* (*Sorocha acutipennis* (F. Bates, 1904), **comb. rev.**); Pelidnota (Odontognathus) nadiae Martínez, 1978 is transferred to the genus *Sorocha* (*Sorocha nadiae* (Martínez, 1978), **comb. rev.**); Pelidnota (Ganonota) plicipennis Ohaus, 1934 is transferred to the genus *Sorocha* (*Sorocha plicipennis* (Ohaus, 1934), **comb. rev.)**; *Pelidnota similis* Ohaus, 1908 is transferred to the genus *Sorocha* (*Sorocha similis* (Ohaus, 1908), **comb. rev.**); Pelidnota (Ganonota) yungana Ohaus, 1934 is transferred to *Sorocha* (*Sorocha yungana* (Ohaus, 1934), **comb. rev.**); *Pelidnota malyi* Soula, 2010: 58, **revised status**; *Xenopelidnota anomala porioni* Chalumeau, 1985, **revised subspecies status**.

To stabilize the classification of the group, a **neotype** is designated for the following species: *Pelidnota thiliezi* Soula, 2009. **Lectotypes** are designated for the following names (given in their original combinations): *Pelidnota brevicollis* Casey, 1915, *Pelidnota brevis* Casey, 1915, *Pelidnota debiliceps* Casey, 1915, *Pelidnota hudsonica* Casey, 1915, *Pelidnota oblonga* Casey, 1915, *Pelidnota pallidipes* Casey, 1915, *Pelidnota ponderella* Casey, 1915, *Pelidnota strenua* Casey, 1915, *Pelidnota tarsalis* Casey, 1915, *Pelidnota texensis* Casey, 1915, and *Scarabaeus punctatus* Linnaeus, 1758.

The following published infrasubspecific names are **unavailable** per ICZN Article 45.6.1: Pelidnota (Odontognathus) cuprea var. coerulea Ohaus, 1913; Pelidnota (Odontognathus) cuprea var. rufoviolacea Ohaus, 1913; Pelidnota (Odontognathus) cuprea var. nigrocoerulea Ohaus, 1913; Pelidnota pulchella var. fulvopunctata Ohaus, 1913; Pelidnota pulchella var. sellata Ohaus, 1913; Pelidnota pulchella var. reducta Ohaus, 1913; Pelidnota unicolor var. infuscata Ohaus, 1913.

The following published species name is **unavailable** per ICZN Article 11.5: *Neopatatra synonyma* Moore & Jameson, 2013.

The following published species name is **unavailable** per application of ICZN Article 16.1: *Parhoplognathus rubripennis* Soula, 2008.

The following published species name is **unavailable** per application of ICZN Article 16.4.1: *Strigidia testaceovirens argentinica* Soula, 2006, Pelidnota (Strigidia) testaceovirens argentinica (Soula, 2006), and *Pelidnota testaceovirens argentinica* (Soula, 2006).

The following published species names are **unavailable** per application of ICZN Article 16.4.2: *Homonyx digennaroi* Soula, 2010; *Homonyx lecourti* Soula, 2010; *Homonyx mulliei* Soula, 2010; *Homonyx simoensi* Soula, 2010; *Homonyx wagneri* Soula, 2010; *Homonyx zovii* Demez & Soula, 2011; *Pelidnota arnaudi* Soula, 2009; *Pelidnota brusteli* Soula, 2010; *Pelidnota chalcothorax septentrionalis* Soula, 2009; *Pelidnota degallieri* Soula, 2010; *Pelidnota lavalettei* Soula, 2008; *Pelidnota lavalettei* Soula, 2009; *Pelidnota dieteri* Soula, 2011; *Strigidia gracilis decaensi* Soula, 2008, Pelidnota (Strigidia) gracilis decaensi (Soula, 2008), and *Pelidnota gracilis decaensi* (Soula, 2008); *Pelidnota halleri* Demez & Soula, 2011; *Pelidnota injantepalominoi* Demez & Soula, 2011; *Pelidnota kucerai* Soula, 2009; *Pelidnota malyi* Soula, 2010: 36-37; *Pelidnota mezai* Soula, 2009; *Pelidnota polita darienensis* Soula, 2009; *Pelidnota polita orozcoi* Soula, 2009; *Pelidnota polita pittieri* Soula, 2009; *Pelidnota punctulata decolombia* Soula, 2009; *Pelidnota punctulata venezolana* Soula, 2009; *Pelidnota raingeardi* Soula, 2009; *Pelidnota schneideri* Soula, 2010; *Pelidnota simoensi* Soula, 2009; *Pelidnota unicolor subandina* Soula, 2009; *Sorocha carloti* Demez & Soula, 2011; *Sorocha castroi* Soula, 2008; *Sorocha fravali* Soula, 2011; *Sorocha jeanmaurettei* Demez & Soula, 2011; *Sorocha yelamosi* Soula, 2011; *Xenopelidnota bolivari* Soula, 2009; *Xenopelidnota pittieri pittieri* Soula, 2009.

Due to unavailability of the name *Pseudogeniates cordobaensis* Soula 2009, we describe the species as intentionally new (*Pseudogeniates cordobaensis* Moore, Jameson, Garner, Audibert, Smith, and Seidel, **sp. n.**).

## Introduction

The pelidnotine leaf chafers (Rutelinae: Rutelini) include the brilliantly metallic jewel scarabs (*Chrysina* spp.; e.g., Fig. 10), large, showy species that are used in ornamentation and jewelry (e.g., *Chrysophora chrysochlora* [Latreille]; Fig. 13), and species that exhibit dramatic sexual dimorphism (e.g., the bulging and dilated hind legs of male *Pelidnota burmeisteri burmeisteri* Burmeister; Fig. 56). The intensely lustrous, metallic colors of *Chrysina* Kirby species have been studied for their rare, cuticular reflection of circularly polarized light (Sharma et al. 2009, Pye 2010). Further studies have demonstrated that this may reduce predation by allowing for communication between conspecifics while remaining cryptic to avoid detection by predators (Brady and Cummings 2010). Ecologically, the leaf chafers have been proposed as valuable bioindicators of high-quality forest (Morón et al. 1997). The group is named for their leaf herbivory tendencies as adults, yet some species may also serve as pollinators of flowering plants (e.g., *Pelidnota sumptuosa* Vigors visits the flowers of *Rourea induta* Planch. and *Stryphnodendron adstringens* (Mart.) Coville in Brazil) (Gottsberger and Silberbauer-Gottsberger 2006). The extant pelidnotine leaf chafers are entirely distributed in the New World and include endemic genera such *Pseudogeniates* Ohaus (endemic to Argentina) and *Homothermon* Ohaus (endemic to Paulista center of endemism in Brazil), as well as widespread genera such as *Pelidnota* MacLeay (distributed from Canada to central Argentina).

Pelidnotine leaf chafers are a poorly studied group with a great need for systematics research. The lack of a taxonomic and phylogenetic framework remains an impediment to the circumscription of natural, monophyletic groups within the Rutelini. Lacking this essential foundation, we cannot understand the evolution of characters such as circular polarization of light in the cuticle of these beetles, the broad context of ecological services such as pollination that the species may provide, and we cannot reconstruct biogeographic patterns nor predict future distributional changes of genera and species of Rutelini.

The objective of this paper is to provide a foundation for understanding the taxonomy of 27 (26 extant and 1 extinct) genera and over 400 species of pelidnotine beetles, assist in stabilizing the classification and nomenclature of the genera, enable identification of genera, and provide a foundation for continued biodiversity research on leaf chafer scarabs. This work synthesizes the taxonomic and biodiversity literature for the pelidnotine scarabs, also encapsulating work that assisted in clarifying the nomenclature for the group (Moore and Jameson 2013, Moore et al. 2014). For the purposes of this research, we refer to this paraphyletic assemblage of taxa as the “pelidnotine scarabs,” and it is our aim that this work will set the stage for future research that addresses broad trends and patterns within the ruteline scarabs.

***Legacy and history.*** Fredrick Bates (1829–1903, collection at BMNH), the younger brother of the well-known tropical biologist and coleopterist Henry Walter Bates (see O’Hara 1995), also had a love for taxonomy and entomology. He conducted research on the Heteromera (Tenebrionoidea) and the pelidnotine scarab beetles. Fredrick Bates died in 1903, and his single work on scarabs was published posthumously with the aid of Gilbert Arrow (The Natural History Museum). In the introduction, Arrow stated: “this revision of a difficult group of beetles represents many months of constant and strenuous investigation, continued to within a very few days of my friend’s death” (Arrow in Bates 1904: 249). Bates had intended the work to be more comprehensive, but his health did not allow further research. Upon Bates’s request, Arrow finished the work. Arrow stated that he confined himself to editorial functions with the exceptions of a few additions and modifications. Some of these modifications are clear within the text as evidenced by brackets and Arrow’s initials. For example, the commentary and diagnosis for the genus *Mecopelidnota* F. Bates and *M. arrowi* F. Bates were clearly added by Arrow.

Friedrich Ohaus (1864–1946, collection at ZMHB) was a student of coleopterist Edgar von Harold and a practicing medical doctor, which allowed him to travel to South America as a ship’s doctor. Ohaus provided the most comprehensive body of literature on world Rutelinae, and he developed the classification of the subfamily Rutelinae that is still used today. His work synthesized the body of knowledge on this highly biodiverse group, providing catalogs of species and their distributions, keys to higher-level groups, natural history, illustrations, and interpretation of characters. Ohaus’s classification of subtribes and genera is largely artificial, but this was a reflection of the state of systematics at the time. *The Genera Insectorum* on the Rutelini (Ohaus 1934b) was delayed for more than 20 years before publication. Instead of waiting for this larger, comprehensive catalog to be completed, Ohaus (1915b) published his concepts on some subtribal taxa within Rutelini and included descriptions of genera. In this work, he formalized the use of the subtribe (as “Pelidnotinorum”) (Ohaus 1915b).

Johann W. Machatschke (1912–1975, collection at NHMB) continued Ohaus’s work, completing the *Genera Insectorum* volumes on Orthochilous Rutelinae (, Adoretini, ) as well as Anomalini. He was the curator of the Coleoptera at Deutsche Entomologische Institut (DEI) in Berlin and later at the Museum G. Frey in Tutzing near Munich.

Marc Soula (1947–2012, collection at CCECL) was a mathematics teacher and naturalist who lived in Massat, France. He traveled broadly to South America and Thailand where he collected Rutelinae. At the Muséum National d’Histoire Naturelle in Paris, he began his life-long work on Rutelinae. Soula, in the fashion of *Sciences Nat* volumes, created guides to assorted rutelines, particularly the larger and showier groups. These volumes, which were published in parts, provide a preliminary effort to understand the diversity in the group. The benefit of these guides is that they provide color images of most species (dorsal habitus and often male genitalia) and species names that, in most cases, were verified by type examination. However, unlike monographic revisions, these guides suffer many shortfalls. They are based on a very limited number of specimens (often holotype specimens only), lack generalized distributional data and, prior to his death in 2012, most holotypes of species named by Soula were unavailable for general study because they were deposited in his personal collection. Soula’s works were written in an unusual style for scientific work. In effect, they were a “stream of consciousness” and lacked synthesis, analysis, and did not make meaningful interpretations or comparisons of characters. Rather than synthesizing his body of work, he published his work in disjunct parts. Sprinkled throughout the volumes (sometimes in red font, sometimes in bold font) he provided corrections to previous volumes such as amended taxonomic decisions, new combinations, new synonymies, and new distributions. These notes are very difficult to track and contributed to Soula’s numerous errors (synonyms, homonyms, *lapsus calami*, unavailable names, and transcriptional errors) (Moore and Jameson 2013, Moore et al. 2014). Soula’s guides provided an outlet for description of many new genera, species, and subspecies, but lacked unified species- or generic-level concepts. Additionally, Soula’s ruteline volumes were not peer-reviewed, were not widely available, and were expensive (thus reducing access). Because the volumes were not peer-reviewed, the data in them were not subjected to the objective scrutiny of other experts on phytophagous scarabs or agreed upon through scientific consensus. The volumes were not well edited, and they suffer from many misspellings (e.g., localities and scientific names), language that is not concise, and omissions (e.g., in the index and catalog). In addition, the lack of peer review and proper scientific editing for Soula’s volumes left numerous published names unavailable when the zoological rules of nomenclature (ICZN 1999) are applied due to various shortcomings and rule violations in the descriptions. Soula’s large collection of Rutelinae now resides in the Musée des Confluences, Lyon, France where it is databased, curated, and accessible for biodiversity research.

***Higher-level nomenclature.*** Many of Soula’s descriptions of new genera within the Rutelini lack information regarding higher-level classification (e.g., *Patatra* Soula, *Pachacama* Soula, and *Homeochlorota* Soula were not clearly assigned to a subtribe of Rutelini at the time of their description). Because his work was published in parts, they included a mix of many genera from formerly accepted subtribes (, ) or accepted subtribes (, ), and they were not organized in a systematic manner. Thus, Soula’s tribal and subtribal classification within the Rutelinae was not clear. Soula recognized that his classification was not based on monophyletic groups (“La plupart des taxons supragénériques, n’étant pas monophylétiques...” [Soula 2011: 3]), but he maintained this classification pending further phylogenetic research. At the same time, however, he abandoned the subtribe (Soula 2006) based on “dissimilarity” of the two genera included by Ohaus (1934b), but he failed to reclassify taxa in the group. Later, Soula (2011) revalidated the subtribe without discussion. Soula’s classification (Soula 2011) omitted Rutelinae tribes ( and Adoretini) and included subtribes that are no longer accepted (e.g., , ) (Smith 2006, Bouchard et al. 2011). Additionally, Soula’s (2011) classification contradicted information in previous publications including the classification of *Minilasiocala* Soula (=*Microogenius* Gutiérrez) in the lasiocaline scarabs versus the pelidnotine scarabs (Soula 2006) and the classification of *Pseudochlorota* Ohaus and *Lasiocala* Blanchard as both pelidnotines and lasiocalines (Soula 2011). Two genera that were formerly included in the subtribe were omitted by Soula (2011): *Oogenius* Solier and *Eremophygus* Ohaus. Because Soula provided no characters or justification for his higher classification and existing phylogenetic evidence demonstrates that and are not monophyletic groups (Jameson 1998), we follow the classification of Bouchard et al. (2011) which lists these subtribes in synonymy under Rutelini.

***Nomenclature.*** *Pelidnota* was placed on the Official List of Generic Names in Zoology (ICZN 2003) and included in the subtribe (tribe Rutelini) by Bouchard et al. (2011). Although the subtribe was hypothesized to be paraphyletic (Jameson 1998), it is the name-bearer for higher-level taxa (Rutelinae, Rutelini). The name has nomenclatural priority over the names Burmeister, Burmeister, Burmeister, Burmeister, Antichirides Lacordaire, Plusiotina Bates, and Ohaus (Bouchard et al. 2011).

The type species of *Pelidnota* is *Scarabaeus punctatus* Linnaeus, 1758. To ensure nomenclatural stability, the name was conserved due to homonymy with *Scarabaeus punctatus* Villers, 1789 (the dynastine scarab *Pentodon bidens punctatus* [Villers]) (ICZN 1999, Krell et al. 2012, Moore and Jameson 2013).

The name “Pelidnota” (from which the subtribe takes its name) is derived from the blackish markings (“pelidnos” or “pelios” = Greek for black; “nota”=Latin for markings) that are common on the elytra of North American *Pelidnota* species.

***Life history and biology.*** Immature life stages are known for only a handful of the pelidnotine genera including *Homonyx* Guérin-Méneville (Morelli 1996), *Chrysina* (Ritcher 1966, Morón 1976, 1985, Morón and Deloya 1991), *Chrysophora* Dejean (Pardo-Locarno and Morón 2007), and *Pelidnota* (Ritcher 1945, 1966, Morón 1976, Morón and Deloya 2002, Rodriguez et al. 2012, Garcia et al. 2013). Based on life history studies, life cycles are one to two years in duration (Ritcher 1966, Morón 1976, Morelli 1996). Larvae are sapro-xylophagous (Morón 1991) and feed on dry, rotten wood (*Pelidnota virescens* Burmeister; Morón and Deloya 2002), hollow trunks and tree stumps (*P. punctata* (Linnaeus) [Hoffman 1926]; *Epichalcoplethis velutipes* Arrow [Chalumeau 1985]), organic matter in the soil, and rotten roots (Morón 1991). One species, *P. filippiniae* Soula, is a significant defoliator and high numbers could contribute to plantation damage (Lunz et al. 2011).

***Human cultural uses.*** The beauty, large size, and ease of collecting of many pelidnotine leaf chafers have promoted the cultural use of many species. For example, in Ecuador and Peru, the Jivaro and Sequoia Indians use the brilliant, metallic green elytra, pronota, or entire bodies of *Chrysophora chrysochlora* (Latreille) to make necklaces and headdresses (Ratcliffe 2006, Ratcliffe et al. 2015). In Guatemala, local people developed a cottage industry for tourists creating pendants, bracelets, and bola ties using local species of *Chrysina* (Woodruff 2009). The Yanomami people of Venezuela and Brazil extract and eat the larvae of *Pelidnota* sp. (known as “Makoia”) from logs in their gardens (Paoletti et al. 2000, Paoletti and Dufour 2005). Many attractive pelidnotine chafers are used in natural, artistic displays, including those of designer and photographer Christopher Marley (Marley 2008).

***Fossil pelidnotines*.** Fossil organisms provide important information on ancestral character states, habitats, ecosystems, and adaptations. The only known leaf chafer fossil sets the minimum age of the subfamily Rutelinae at 50-42 mya (Krell 2006). The pelidnotine-like *Pelidnotites atavus* Cockerell is from the Eocene of England in the Bartonian Bagshot Beds of Bournemouth (Cockerell 1920, Carpenter 1992). Cockerell (1920) described the fossil belonging to the Rutelini and “in the vicinity of *Pelidnota* and *Cotalpa*” (Cockerell 1920: 463). This fossil should be examined to place the taxon within the Rutelini and to hypothesize sister-group relationships. No pelidnotine relatives are currently distributed in England or the Old World. Thus, this fossil revealed distributional patterns quite different from the current range of the pelidnotine Rutelini.

***Identification of pelidnotine scarabs.*** Keys to the genera of “” were created by F. Bates (1904) and Ohaus (1934b), and these provided a weak foundation for future work in the group. Bates’s (1904) posthumous work was based almost exclusively upon specimens available to him at the Natural History Museum (BMNH). Because this collection did not contain all described taxa in , the revision and key were incomplete. Keys to genera and species groups that were provided by Ohaus (1934b) are not adequate for reliable identification of pelidnotine scarabs. Many of Ohaus’s (1934b) couplets are, in our estimation unclear, asymmetrical, and based on characters that vary widely across species and genera of . Soula’s (2006) key to pelidnotine genera was based on Ohaus’s keys. Soula’s (2006) key omitted five genera that were newly described or elevated to generic status by Soula (*Chipita* Soula, *Epichalcoplethis* Burmeister, *Pachacama*, *Patatra*, and *Sorocha* Soula) and included genera that previously had been transferred out of Rutelini or synonymized (*Pelidnotopsis* Ohaus, *Peltonotus* Burmeister, and *Plusiotis* Burmeister) (see Moore and Jameson 2013). To date, there is no reliable, comprehensive key that facilitates accurate and repeatable identification of pelidnotine genera by non-experts. We think that consistent generic- and species-level identification of pelidnotine scarabs is not possible at this time. This lack of basic information is a great impediment to biodiversity and ecological research on pelidnotines.

***Classification and phylogeny.*** The leaf chafers (Rutelini) are members of the phytophagous scarab beetle clade (Melolonthinae, Cetoniinae, Dynastinae, Rutelinae, and a few minor subfamilies), a group that has been widely accepted as monophyletic for about 150 years and corroborated by molecular and morphological phylogenetic studies (Smith et al. 2006, Ahrens et al. 2011, McKenna et al. 2014). At the tribal- and subtribal-level morphological phylogenetic analyses demonstrated the inadequacies of the Rutelini classification *sensu* Machatschke (1972) (Jameson 1998). Based on this research, several subtribes (e.g., , , and ) in the Rutelini were not monophyletic and, in fact, they were uninformative and logically inconsistent (Jameson 1998). Paraphyly of the ruteline subtribes has been a matter of discussion for well over a century. In his work on the pelidnotine scarabs, F. Bates (1904) noted the “close relationship” of *Pelidnota* and *Rutela* Latreille, and this idea was corroborated based on phylogenetic analyses of Rutelinae (Jameson 1998, Ahrens et al. 2011). Jameson (1998) provided the basis for elimination of the subtribe and concluded that many genera were poorly characterized and not based on synapomorphic or autapomorphic characters.

Relationships among the pelidnotine scarabs need to be addressed by phylogenetic analyses. Pelidnotine genera, especially *Pelidnota* and the generic-level synonyms of *Pelidnota* (e.g., *Strigidia* Burmeister), should be re-structured into monophyletic groups with clear hypothesized synapomorphies. Based on phylogenetic analyses (Jameson 1998), *Pelidnota* and several related genera form a grade of taxa that are currently treated as genera or subgenera. Because of the poor understanding of the group and the lack of synapomorphies delimiting genera, past workers elevated distinctive species to the rank of genus, thus creating many monotypic genera (e.g., *Pelidnotopsis* [=*Chrysina*] and *Ectinoplectron* Ohaus). Hardy (1975) did not discuss relationships among the subgenera of *Pelidnota*, although he noted that the classification and subgeneric concept as proposed by Ohaus were in need of study. In our estimation, several of the current pelidnotine genera are probably not valid and *Pelidnota*, in particular, is likely to include several distinct, monophyletic groups (i.e., at the generic-level).

Research on specific pelidnotine genera has led to classification changes that affected the composition of pelidnotine scarabs. For example, *Plusiotis* and *Chrysina* were historically separate genera. As new species were described, our understanding of characters that circumscribe these groups was broadened and, as stated by Morón and Howden (1992: 208), the “characters that have been used to separate *Plusiotis* and *Chrysina* form a non-concordant mosaic.” Based on molecular and morphological data, Hawks (2001) synonymized *Plusiotis* as well as *Pelidnotopsis* within the genus *Chrysina*. Additionally, revisionary research on the genus *Peltonotus*, which was considered a member of the pelidnotine scarabs, provided sound evidence that the genus *Peltonotus* is closely related to members of the subfamily Dynastinae rather than the Rutelinae (Jameson and Wada 2004, 2009, Smith et al. 2006, Jameson and Drumont 2013). In spite of phylogenetic evidence that the subtribe was paraphyletic, Soula (2006, 2008, 2009) maintained the subtribe, maintained the generic composition of the subtribe by including genera that had been transferred or synonymized, and he refrained from re-characterizing the group in any way.

We reiterate that , as historically defined, is a paraphyletic grouping of disparate genera and species, and it should not be considered a valid taxon. We consider the subtribe a synonym of the subtribe (Jameson 1998, Bouchard et al. 2011). We refer to “pelidnotine scarabs” in order to: 1) synthesize information on a group of genera that was chaotically treated by Soula, 2) incorporate genera new to the subfamily Rutelinae and previously of uncertain tribal placement (*Peruquime* Mondaca and Valencia and *Neogutierrezia* Martínez), 3) provide a mechanism for generic identification (in the form of a provisional dichotomous key), and 4) set the stage for future research that addresses broader trends within the Rutelini scarabs.

## Materials and methods

***Specimens and taxonomic material.*** Specimens examined for this study were provided by many institutions and private collections. We include information on type specimens to provide a foundation for continued research in the leaf chafers. Type specimens are international standards for scientific names (Knapp et al. 2004) and are tied to species hypotheses. The type specimen provides the nomenclatural stability that assures that the name reflects the described species and is linked through history in the literature. Acronyms for loaning institutions follow Evenhuis (2016).

**BIOG** Biodiversity Institute of Ontario, University of Guelph, Guelph, Ontario, Canada (Renee Labbee)

**BMNH** The Natural History Museum, London, United Kingdom (Max Barclay, Beulah Garner)

**CAS** California Academy of Sciences, San Francisco, California, USA (Norman Penny)

**CCECL** Musée des Confluences, Lyon, France (Cédric Audibert)

**CMNC** Canadian Museum of Nature Collection, Ottawa, Canada (Robert Anderson, François Génier)

**CNC** Canadian National Collection of Insects, Arachnids, and Nematodes, Ottawa, Canada (Pat Bouchard)

**CNIN** Colección Nacional de Insectos, Instituto de Biología, Universidad Nacional Autónoma de México (UNAM), México D. F., México (Harry Alperowitz, Santiago Zaragoza Caballero)

**DBPC** Denis Bouchard Personal Collection, Autouillet, France

**DEPC** David Ebrard Personal Collection, Velars sur Ouche, France

**DJCC** Daniel Curoe Personal Collection, Palo Alto, California, USA

**EAPZ** Escuela Agrícola Panamericana, Tegucigalpa, Honduras (Ron Cave, Jesús Orozco)

**EMEC** Essig Museum of Entomology, University of California, Berkeley, California, USA (Cheryl Barr, Peter Oboyski)

**FMNH** Field Museum of Natural History, Chicago, Illinois, USA (Alfred Newton, Crystal Maier)

**FSCA** Florida State Collection of Arthropods, Gainesville, Florida, USA (Paul Skelley)

**HNHM** Hungarian Natural History Museum, Budapest, Hungary (Ottó Merkl)

**IAZA** Instituto Argentino de Investigaciones de las Zonas Áridas, Mendoza, Argentina (Adriana Marvaldi)

**IEXA** Colección Entomológica, Instituto de Ecología, A.C., Xalapa, México (Miguel Ángel Morón)

**IFML** Instituto Fundación Miguel Lillo, Tucumán, Argentina (Dominga Carolina Berta)

**IRSNB** Institute Royal des Sciences Naturelles de Belgique, Brussels (Alain Drumont)

**INBC** Instituto Nacional de Biodiversidad, San José, Costa Rica (Ángel Solís)

**INPA** National Institute for Amazonian Research, Manaus, Brazil (Marcio Luiz de Oliveira)

**IREC** Institut de Recherches Entomologique de la Caribe, Pointe-a-Pitre, Guadeloupe (also known as Centre de Recherches Agronomiques Antilles Guyana, Duclos, Petit-Bourg [CRAAG] (Girard Chovet, Fortuné Chalumeau)

**JEMC** José Mondaca E. Personal Collection, Peñaflor, Chile

**JPBC** Jean-Pierre Beraud Personal Collection, Cuernavaca, Morelos, México

**LACM** Los Angeles County Museum of Natural History, Los Angeles, California, USA (Brian Brown, Weiping Xie)

**MACN** Museo Argentino de Ciencias Naturales, Buenos Aires, Argentina (Arturo Roig)

**MCZ** Museum of Comparative Zoology, Harvard University, Cambridge, Massachusetts, USA (Brian Farrell, Naomi Pierce)

**MHNN** Muséum d’Histoire Naturelle, Geneva, Switzerland (Peter Schwendinger)

**MHNP** Museo de Historia Natural, Universidad Nacional de San Antonio Abad, Cusco, Perú (Percy Yangue Yucra)

**MIUP** Museo de Invertebrados “G.B. Fairchild”, Universidad de Panamá, Panamá (Diomedes Quintero Arias)

**MIZA** Museo del Instituto de Zoología Agrícola, Maracay, Venezuela (José Clavijo)

**MLJC** Mary Liz Jameson Personal Collection, Wichita, Kansas, USA

**MLPA** Museo de la Plata, Universidad Nacional de la Plata, La Plata, Argentina (Analía Lanteri, Nora Cabrera)

**MLUH** Zentralmagazin Naturwissenschaftlicher Sammlungen, Martin-Luther-Universität Halle-Wittenberg, Halle, Germany (Karla Scheider, Joachim Händel)

**MNHN** Muséum National d’Histoire Naturelle, Paris, France (Olivier Montreuil)

**MNNC** Coleccion Nacional de Insectos, Museo Nacional de Historia Natural, Santiago, Chile (Mario Elgueta)

**MNCR** Museo Nacional de Costa Rica, San José, Costa Rica (formerly INBC, Ángel Solís)

**MSPC** Matthias Seidel Personal Collection, Prague, Czech Republic

**MTD** Museum für Tierkunde, Dresden, Germany (Klaus-Dieter Klass, Olaf Jäger)

**MXAL** Miguel Ángel Morón Collection, Xalapa, México

**NHMB** Naturhistorisches Museum, Basel, Switzerland (Daniel H. Burckhardt)

**NMPC** Department of Entomology, National Museum (Natural History), Prague, Czech Republic (Jiří Hájek)

**OUMNH** University Museum of Natural History, Oxford, United Kingdom (Darren Mann, Amoret Spooner)

**PAPC** Patrick Arnaud Personal Collection, Saintry sur Seine, France

**PMNH** Peabody Museum of Natural History, Yale University, New Haven, Connecticut, USA (Leonard Munstermann)

**PVGH** Pedro Vidal Personal Collection, Santiago, Chile

**SDEI** Senckenberg Deutsches Entomologisches Institut, Müncheberg, Germany (Lothar Zerche, Konstantin Nadein)

**STRI** Smithsonian Tropical Research Institute, Balboa, Panama (Annette Aiello)

**UAEH** Universidad Autónoma del Estado Hidalgo, Pachuca, Hidalgo, México (Juan Marquez Luna)

**UAG** Escuela de Biología de la Universidad Autónoma de Guadalajara, México (Jose Luis Navarette)

**UCCC** Museo de Zoología, Universidad de Concepción, Concepción, Chile (Jorge Artigas)

**UCRC** Entomology Research Museum, Department of Entomology, University of California, Riverside, California, USA (Doug Yanega)

**UFRJ** Museu Nacional, São Cristóvão, Rio de Janeiro, Brazil (Miguel Monné, Marcela Monné)

**UVGC** Colección de Artrópodos, Universidad del Valle de Guatemala, Guatemala City, Guatemala (Jack Schuster, Enio Cano)

**UNSM** University of Nebraska State Museum, Lincoln, Nebraska, USA (Brett Ratcliffe, M. J. Paulsen)

**USNM** U.S. National Museum, Washington, D.C. (currently housed at the University of Nebraska State Museum for off-site enhancement) (Brett Ratcliffe, Floyd Shockley)

**UUZM** Zoological Institute, Uppsala University, Uppsala, Sweden (Hans Mejlon)

**WBWC** William B. Warner Personal Collection, Chandler, Arizona, USA

**WSU** Maurice T. James Entomological Collection, Washington State University, Pullman, Washington, USA (Richard Zack)

**ZMHB** Museum für Naturkunde der Humboldt-Universität, Berlin, Germany (Manfred Uhlig, Joachim Willers, Johannes Frisch)

**ZSMC** Zoologische Staatssammlung des Bayerischen Staates, Munich, Germany (Martin Baehr)

***Images and terminology***. Digital images of type specimens were taken over a 10-year period and were captured using several imaging applications including the Leica Application Suite V3.8. Images were edited in Adobe Photoshop CS2 (background removed, contrast manipulated). Figure legends for type specimens provide the valid, accepted name (as in the catalog) and the original combination of the species. We provide images of type specimen labels, but we largely defer from designating specimens as lectotypes. In our view, this is incumbent upon future revisionary scientists who will observe the best practices of systematics (ICZN 2003) and properly assign type status to specimens based on thorough review of all literature. Morphological terminology follows Hardy (1975) and Jameson (1998). Characters and specimens were observed with 6.3–50.0x magnification and fiber-optic illumination.

***Literature reviewed.*** In compiling this work, we reviewed all available literature including major catalogs (Ohaus 1918, 1934b, Blackwelder 1944, Machatschke 1972, 1974, Smith 2009, Krajcik 2008, 2012, 2013). Some nomenclatural decisions were made by Krajcik (2012), but these were not indicated with “new synonymy” or “new combination,” and the rationale for these changes was not provided. For example, Krajcik did not accept Soula’s concepts of *Epichalcoplethis, Chalcoplethis* Burmeister, and *Sorocha*. Instead, he synonymized all names under *Pelidnota*. Also, Krajcik’s acceptance of names was not homogenous. He included some of Soula’s species and subspecies, but not all of them. For example, from the same publication (Soula 2009), Krajcik included *Pelidnota estebanabadiei* Soula (2009: 34), but he did not include *Pelidnota equatoriana* Soula (2009: 32). Krajcik included all of the following names: *Pelidnota bondili* Soula (2006: 10), *Pelidnota castroi* Soula (2008), and *P. belti boyacaensis* Soula (2006: 73), but he did not include any of the following names: *Pseudogeniates cordobaensis* Soula (2009), *Pelidnota brusteli* Soula (2010a), *P. ohausi piurensis* Soula (2006: 22), and *P. sanctidomini caliensis* Soula (2006: 79). Lacking his rationale, we did not follow Krajcik’s classification for the pelidnotine chafers. We discuss Krajcik’s (2008, 2012, 2013) catalogs to synthesize all available information for pelidnotine scarabs. However, we are agnostic about Krajcik’s listing of generic-, specific-, and subspecific-level synonyms amongst pelidnotine scarab taxa. Krajcik’s catalogs, in our opinion, are useful for tracking data on the proliferation of names in the hyper-diverse Scarabaeoidea, but should not be used to inform classifications. In our generic overviews and annotated catalog, we were forced in most cases to follow Soula’s generic- and species-level classifications (inadequate and uninformative though they are) because they are valid until addressed in broader systematic and revisionary works.

Soula’s descriptions of new taxa were often vague about the number, sex, and associated label data of type specimens. To rectify this lack of basic information, we report the verbatim label data of every pelidnotine scarab type specimen deposited in the Soula collection now housed at CCECL and CMNC. Due to the number of taxa that Soula named and described, both collections are rich in Rutelini type specimens. Among the pelidnotine scarabs, Soula’s material contains over 80 primary types (holotypes and neotypes) and nearly 700 secondary types (allotypes, paratypes, and paralectotypes) that are now all curated at CCECL. Additionally, examination of the CCECL collection revealed Soula specimens with type labels that had not been validly described. These “manuscript names” and the associated specimen data are listed in the appropriate genera (see “Annotated Catalog” below) as *in litteris*, and they are unavailable names. In a few instances, it appears that Soula omitted paratype data from the original published description or added paratype specimens to a type series after publication of a species description. For example, he added 17 “paratypes” collected in 2011 to *Pseudochlorota peruana lecourti* (described by Soula in 2005). Soula clearly knew that this violated nomenclatural rules, because he stated: “Répétons que de nombreux “cotypes” de cette Collection ne sont pas de “bons” types car ils ont été désignés et étiquetés après la description originale” (Soula 2005). We report these specimens as “invalid types” or “probable paratypes.” Soula also mislabeled type specimens (holotypes and paratypes), and we noted and corrected these mistakes when possible by referencing Soula’s original species descriptions.

During our study of the Soula collection at CCECL, we were able to return some primary type material to the institutions that had loaned specimens to Marc Soula (Garner and Audibert 2015). This type material had remained in his collection following his death. Additionally, we were able to gather information about what happened to the Soula collection after his passing, but before it was properly and legally accessioned by CCECL. Before CCECL acquired the Soula collection, it was briefly in control of his family. Unfortunately, the scientific value of some specimens was not recognized initially by the family and it seems likely that some material from the Soula collection or loaned specimens (possibly primary types) of an unknown species or several species (described only as a *Pelidnota*-like species with large legs [possibly some species similar to *Pelidnota burmeisteri* Burmeister or *Chrysina* species]) were lost in a sale to an antique collector (pers. comm. from Patrick Arnaud, June 2014) (Garner and Audibert 2015). Future systematists will have to deal with the uncertainty surrounding these possibly lost type specimens. Fortunately, it seems to us that this was an isolated and limited incident. We stress here that this is a cautionary tale that highlights the importance of properly maintaining loan records, providing temporary institutional labels on loaned specimens, and tracking the fate of personal collections that contain type material, regardless of taxonomic group.

***Annotated Catalog*.** We list the author and date of the description of the species and genera, type species of genera (indicated with an asterisk), subspecies and forms, and transfer of species to other genera. This catalog builds on the work of Ohaus (1918, 1934b) and Machatschke (1972, 1974) with additions by Hardy (1975), Soula (1998, 2002a, 2002b, 2003, 2006, 2008, 2009, 2010a, 2010b, 2011), Krajcik (2008, 2012, 2013), and other authors. References to original descriptions of all species and genera are provided. Entries for species in the catalog provide: 1) the valid species name, author, date, and abbreviated citation, 2) original spelling and combination (if applicable), misspellings, new combinations, and invalid names in chronological order, 3) synonyms and the reference in which the synonym was designated, 4) general distribution data including the country (in capital letters) and states/provinces/departments/communes when they are known. Distributions are based on the literature and on specimens that we examined.

***Rules of zoological nomenclature.*** Numerous nomenclatural changes within the pelidnotine scarabs are necessary due to misspellings, invalid type designations, and unavailability of infrasubspecific names (Moore and Jameson 2013, Moore et al. 2014). We follow the International Code of Zoological Nomenclature (ICZN 1999) as a means of stabilizing the taxonomy and classification of the pelidnotine scarabs.

Infrasubspecific names such as varieties and forms were widely used by authors such as Friedrich Ohaus and occasionally Marc Soula. These names are used to indicate unique color variants. Many of these names were treated as forms (forma; infrasubspecific entities) in catalogs (Machatschke 1972, 1974) as well as in works by Soula. According to ICZN Article 45.6.4: “A name is subspecific if first published before 1961 and its author expressly used one of the terms “variety” or “form” (including use of the terms “var.”, “forma”, “v.” and “f.”), unless its author also expressly gave it infrasubspecific rank, or the content of the work unambiguously reveals that the name was proposed for an infrasubspecific entity, in which case it is infrasubspecific” (see Lingafelter and Nearns 2013). Thus, named entities need to be interpreted within the context of the publication to discern if a name was unambiguously infrasubspecific. That is, if the author described or discussed both subspecies and varieties within a work, then it is clear that varieties can be treated as infrasubspecific, thus making the name unavailable unless further action was taken to correct the names prior to 1985 (ICZN Art. 45.6.4.1).

Species for which Soula designated type material but for which specimens are missing and presumed lost resulted in our neotype designations. The International Code of Zoological Nomenclature (ICZN 1999) requires that a neotype “is validly designated when there is an exceptional need and only when that need is stated expressly” (75.3). To be validly designated, Article 75.3.7 (ICZN 1999) requires a statement regarding the accessibility of the specimen. Upon publication, the specimen must be “the property of a recognized scientific or educational institution, cited by name.” Thus, some neotypes were invalidly designated by Soula. Designation of some neotype specimens was necessary for names proposed by Soula.

The lack of synthesis and attention to detail in Soula’s works resulted in some names that were not validly described (see Moore and Jameson 2013, Moore et al. 2014). For all new species-group names described after 1999, the holotype and the type depository must be explicitly stated for the name to be deemed available (ICZN Art. 16.4). Because Soula (2006, 2008, 2009, 2010a, 2011) did not explicitly state the location of holotypes for several species, these names are unavailable.

For groups that have dramatic sexual dimorphism, some taxonomists refer to the “alloréférent” or the “neallotype” specimen for the first specimen of the opposite sex that is described in a publication subsequent to the original description (Dechambre 2001). Unlike name-bearing type specimens (e.g., holotype, lectotype, neotype), these specimens have no formal nomenclatural status (Hawksworth 2010). Soula frequently made use of the term “alloréférent” in his collection and his published works. We stress that these specimens are not name-bearing type specimens.

Poor editing and many misspellings compromise the scientific value of Soula’s works (e.g., scientific names, localities, descriptive characters, figure legends, indices, and identification keys). These errors pose problems because they can be propagated by future researchers. And, in some cases, the error confuses or obscures Soula’s intended species name. We include these misspellings to limit confusion and promote future research.

***Type specimens and lectotype designation.*** For purposes of nomenclatural stability, we designate lectotypes for some species (ICZN Art. 74). In these cases, a specimen was selected among a group of syntype specimens or cotype specimens. During this research (initiated by MLJ in the late 1990s) many type series were studied, lectotype labels added, and specimens returned to museum collections. However, when research on the group became intractable due to Soula’s concurrent work on the group, these lectotypes were not published. Soula also designated lectotypes. In some cases, he removed previous lectotype labels and he changed the collection depository. In other cases, we have reason to reject Soula’s attempted lectotypification. For example, Soula designated paralectotypes without first designating a lectotype (see *P. laevissima* Burmeister in Soula 2009), he stated that the type series consists of only a holotype but he provided an image of a lectotype (see *Homothermon praemorsus* (Burmeister) in Soula 2008) and, for a species named by Sharp in 1877, he designated a holotype and paratype (rather than a lectotype and paralectotype) (see *P. prolixa* Sharp in Soula 2009). All of these cases gave us reason to be cautious of Soula’s lectotypification. Thus, in instances where we have verified label data, we refer to types specifically (lectotype, paralectotype, holotype). In other instances, however, we refer to type specimens as “types,” and we leave lectotype designation to future systematists. We provide images of many type specimens, but it is not our purpose to nomenclaturally fix or designate types with these images.

***Concepts for genera and species.*** In this work, we do not generally assess the validity of species, subspecies, or genera. In our view, this is best conducted as part of comprehensive, revisionary studies. Instead, we provide a taxonomic and nomenclatural framework for future research. Although we do not name new species within this work, we adhere to the phylogenetic species concept (Wheeler and Platnick 2000) in our interpretations: “A species is the smallest aggregation of (sexual) populations or (asexual) lineages diagnosable by a unique combination of character states.” New nomenclatural acts in this work, such as new synonyms and new homonyms, are based on examination of type specimens and in accordance with the rules of zoological nomenclature (International Code of Zoological Nomenclature, 1999).

Hardy’s standards for species circumscription provide a solid basis for ruteline systematics. Hardy’s (1975) classic work on *Pelidnota* from North and Central America provided the foundation for our knowledge of pelidnotine species as well as a rigorous foundation for interpretation of intraspecific variation. Hardy’s species concepts within the genus *Pelidnota* have endured for over four decades. Hardy (1975) considered species to be “variable entities” and he allowed for intraspecific variation in coloration, maculae, the degree of posterior coxal corner production, and even the form of male genitalia. Hardy allowed intraspecific variation in parameres in *P. punctata* (Hardy 1975; Figs 34–36) and *P. costaricensis* H. W. Bates (Hardy 1975; Figs 38, 39) just to name a few. Because all specimens of all populations of species cannot realistically be studied, actual distributions of characters must be theorized based on available specimens. Species-level lineages are hypothetically circumscribed and these hypotheses should be testable (i.e., subject to the consideration of additional data). Only by examining a large number of specimens and seeing continuity between populations was Hardy (1975) able to conclude that the observed variation was intraspecific rather than interspecific.

In our view, Hardy’s (1975) method of study provided a good test of historical species concepts in *Pelidnota* as additional specimens and populations were discovered and species boundaries could be critically evaluated (see Wheeler 2004). Critical evaluation of species boundaries is an important (but largely undiscussed) concept within pelidnotine leaf chafer taxonomy due to the elaborate male genital morphology (considered diagnostic for identification) present in many species. Pelidnotine scarab species, and Rutelini more generally, often have asymmetric parameres and ventral genital plates originating from the phallobase (e.g., in the genera *Homothermon*, *Xenopelidnota* F. Bates, and many *Pelidnota*). The cuticular generation of these asymmetric structures is certainly a complex and highly sensitive developmental process that we think gives rise to a great deal of intraspecific variation in male genital characters. Many historical *Pelidnota* workers, outside of Hardy (1975), have interpreted this type of genital shape variation to be interspecific and subsequently split species where we may have lumped them into broader, variable species.

In contrast to Hardy’s (1975) and our species concept, Soula’s species concept (1998, 2002a, b, 2003, 2006, 2008, 2009, 2010a, b, 2011) did not allow for intraspecific variation. In Soula’s works, slight differences in color, punctation, or form of male parameres equated to different species or subspecies. In fact, slight differences in male parameres could be attributed to minor deviation in the manner in which parameres were viewed. For example, Soula’s description of *S. purpurea esperitosantensis* was based on two male specimens from Espírito Santo, Brazil (Soula 2006). Soula’s line drawings of the male parameres are extremely similar to the nominate subspecies, and Soula remarked that parameres of both were “slightly different.” Both the nominate species and subspecies were known to Soula from fewer than five specimens in total, and only the nominate form of the species was included in Soula’s key (2006: 9-12).

In the pelidnotine scarabs, Soula (2006, 2008, 2009, 2010a, 2011) described over 150 new species and subspecies and ultimately classified approximately 100 new species and subspecies in *Pelidnota*. Quantifying the number of specimens that Soula’s pelidnotine species-group names were based upon illustrates the lack of intraspecific variation incorporated into his species concept. Forty-four percent (67 of 152) of Soula’s new species and subspecies of pelidnotine scarabs were described from two (minimum number for both sexes to be described) or one specimen. Approximately 33% (50 of 152) of Soula’s new pelidnotine names were based on descriptions of a single, male holotype specimen. In total, 41% (63 of 152) of Soula’s new pelidnotine names were based upon descriptions of only one sex. Soula’s species and subspecies concepts were almost singularly reliant on slight differences in male paramere morphology and/or broadly separated populations. These concepts, when paired with the limited number of specimens of some genera to which he had access, led to his inability to reliably diagnose species when either of these conditions was violated. For example, Soula stated that he could not diagnose the females of *Sorocha* Soula species when they are even narrowly sympatric. For example, for diagnosis of “*S. yelamosi*” Soula stated (2011: 82), “Là encore la femelle est à capturer et à repérer. Plusieurs espèces semblent cohabiter et il ne sera pas facile d’appareiller les couples”. In sum, adequate characters were not provided to circumscribe many of Soula’s species-level hypotheses. Soula’s guides provided the outlet for description of many new genera, species, and subspecies, but an adequate concept that guided his hypotheses was lacking. In our view, Soula’s species and subspecies concepts cast doubt on the validity of many of his taxa.

***DNA barcode analysis for Pelidnota punctata*.** Cytochrome Oxidase 1 (CO1) DNA data were used to address genetic variation in *Pelidnota punctata* across the distributional range of the species. Using the Barcode of Life Database (BOLD: http://www.boldsystems.org), CO1 data were gathered for *P. punctata* (13 specimens) and 10 other species of *Pelidnota* (38 specimens). The distance model used the Kimura 2 parameter with a neighbor-joining tree building method in BOLD. Nodes are labeled by species name, BOLD ID number, and country and state/province where the specimen was collected (Fig. 4).

***Overviews of genera.*** Biological and natural history data in the “Generic Overviews” were synthesized from the literature, specimens, and specimen labels from many institutions. Overviews do not summarize all literature and all specimens. Instead, they highlight: 1) potential complications such as paraphyly and nomenclatural issues, 2) potential synapomorphic characters and discuss possible sister-group relationships, 3) basic distribution and habitat affiliations, and 4) known larvae and natural history information.

### Overview of the pelidnotine genera

***Diagnosis.*** Pelidnotine scarabs are members of the tribe Rutelini (for a key to tribes of Rutelinae, see Jameson 1990, 2005). Characters that diagnose the Rutelini pelidnotine scarabs include: obvious membranous border on the elytra lacking (versus membranous border present at the elytral apex as in Anomalini); frontoclypeal suture obsolete at middle (versus complete as in the ruteline subtribes Heterosternina and Areodina); labrum that is horizontally produced with respect to the clypeus (versus vertically produced as in Geniatini, Anoplognathini, Anatistini, and Adoretini) and sinuate at apex; protarsomeres that are subcylindrical and lack ventral setose pads (versus dorsoventrally flattened and densely setose ventrally as in Geniatini); protibial spur apical (versus subapical as in Anomalini); and terminal spiracle positioned in pleural suture (versus not positioned in pleural suture as in Anomalini).

Males and females are generally separated based on the inner protarsal claw that is wider than the outer claw and may or may not possess a small, inner tubercle. Protarsal claws of the females, in comparison, are more similar in width and lack a small, inner tubercle.

***Identification key.*** We provide a provisional key to the pelidnotine scarabs that should be used with caution. First, the subtribe Pelidnotina is paraphyletic and users should not be misled into thinking that the key circumscribes a natural group. Second, some genera are also very likely paraphyletic, thus causing complications for circumscription and identification. Third, the key will not be useful for both males and females for some genera (e.g., *Mesomerodon* Ohaus, *Hoplopelidnota* F. Bates) due to use of sexually dimorphic characters. Fourth, owing to likely paraphyly, the genera *Microogenius* and *Eremophygus* could not be separated in the key. Fifth, two genera are keyed twice (*Chalcoplethis*, *Epichalcoplethis*). These complications in key construction are indicators of the complexity of the group and need for further systematics studies.

### Key to the genera of pelidnotine scarabs (Coleoptera, Scarabaeidae, Rutelinae, Rutelini)

Males: inner protarsal claw wider than the outer claw; may or may not possess a small, inner tubercle; sternites usually concave. Females: protarsal claws similar in width; lack a small, inner tubercle; sternites usually convex.

**Table:** 

1	Claws on all legs simple and of similar size; protarsal claw (male) lacking apical or subapical tubercle, lacking apical split	***Neogutierrezia* Martínez**
–	Claws on all legs with the inner claw different than the outer claw (wider or split apically); protarsal claw (male) wider than outer claw, with or without small, inner tubercle, and with or without apical incision	**2**
2	Labrum and clypeus fused anteriorly	***Peruquime* Mondaca and Valencia**
–	Labrum and clypeus not fused anteriorly, free	**3**
3	Lateral edge of mandible lobe-like and flattened, without reflexed teeth (e.g., Fig. 1A)	**4**
–	Lateral edge of mandible not flattened, with 1 or 2 reflexed teeth (Fig. 1B, C, E, F)	**8**
4	Apex of labrum extends beyond clypeal apex, visible from dorsal view	**5**
–	Apex of labrum does not extend beyond clypeal apex, not visible from dorsal view	**6**
5	Metatarsomere 4 at apex with 4-6 spinules, medial spinules thickened	***Oogenius* Solier**
–	Metatarsomere 4 at apex with 4-6 spinules, medial spinules seta-like (not thickened)	***Microogenius* Gutiérrez** and ***Eremophygus* Ohaus**
6	Clypeus with apex quadrate or subquadrate (Fig. 1D); apex emarginated	**7**
–	Clypeus with apex rounded, parabolic, or trapezoidal; apex with or without emargination (Fig. 1A, B, C, E, F)	**8**
7	Lateral edge of protibia with 2 teeth	***Chipita* Soula**
–	Lateral edge of protibia with 3 teeth	***Parhoplognathus* Ohaus**
8	Apex of elytra in males with acute, spiniform projections (Fig. 3C, D)	**9**
–	Apex of elytra in males rounded	**10**
9	Males without acute process on posterior margin of mesofemur. Females with 2 deep emarginations near the apex of the terminal sternite; pygidial disc with a concavity. Dorsal color metallic green	***Hoplopelidnota* F. Bates**
–	Males with acute process on posterior margin of mesofemur (Fig. 2E). Females lacking emarginations at apex of terminal sternite, instead apex is rounded; pygidial disc convex. Dorsal color testaceous or light-brown (with or without weak metallic-green reflections)	***Mesomerodon* Ohaus**
10	Pronotum with apical bead obsolete or lacking medially (Fig. 1A)	***Chrysina* Kirby**
–	Pronotum with apical bead complete medially (Fig. 1B, C, D, F)	**11**
11	Males with metatibia enlarged, curved, produced posteriorly at apex	***Chrysophora* Dejean**
–	Males without metatibia enlarged, curved, produced posteriorly at apex	**12**
12	Metatarsomeres 1–5 longer than metatibia	***Chalcoplethis* Burmeister**
–	Metatarsomeres 1–5 subequal to metatibia	**13**
13	Metatibia somewhat laterally flattened (Fig. 2A)	***Epichalcoplethis* F. Bates**
–	Metatibia not laterally flattened (Fig. 2B)	**14**
14	Prosternal projection (between procoxae) produced to level of procoxae	**15**
–	Prosternal projection (between procoxae) shorter, not produced to level of procoxae ***Xenopelidnota* F. Bates**
15	Base of metatibia with semicircular notch (Fig. 2C)	***Mecopelidnota* F. Bates**
–	Base of metatibia lacking semicircular notch, straight (Fig. 2D)	**16**
16	Apex of metatibia straight and with numerous spinules	***Ectinoplectron* Ohaus**
–	Apex of metatibia not straight (biemarginate or with external apex produced), with 0–8 spinules	**17**
17	Metatibia lacking produced external apex, lacking apical spinules	**18**
–	Metatibia with external apex produced posteriorly and with apical spinules	**26**
18	Disc of frons with weak V-shaped depression (Fig. 1E)	***Sorocha* Soula**
–	Disc of frons planar, smooth, lacking a V-shaped depression (Fig. 1A, B, C, D, F)	**19**
19	Metatibia laterally flattened	**20**
–	Metatibia not laterally flattened	**21**
20	Metatarsomeres 1-5 subequal to metatibia	***Epichalcoplethis* F. Bates**
–	Metatarsomeres 1-5 longer than metatibia	***Chalcoplethis* Burmeister**
21	Elytral shoulder rounded (not flat in ventral view), lacking bead	***Homothermon* Ohaus**
–	Elytral shoulder flat in ventral view, with bead	**22**
22	Mesometasternal keel surpassing mesocoxae (Fig. 3B)	***Pelidnota* MacLeay**
–	Mesometasternal keel not surpassing mesocoxae (Fig. 3A)	**23**
23	Lateral edge of mandible with two reflexed teeth (Fig. 1B, E, F)	**24**
–	Lateral edge of mandible with one reflexed tooth (Fig. 1C)	**25**
24	Metatarsomere 3 with apical setae (externally) of unequal length and width; color castaneous to black	***Homonyx* Guérin-Méneville**
–	Metatarsomere 3 with apical setae (externally) of equal length and width; color metallic green	***Catoclastus* Solier**
25	Fifth meso- and metatarsomeres without internal teeth, tarsomeres simple	***Pseudogeniates* Ohaus**
–	Fifth meso- and metatarsomeres with one or two internal teeth (may be rounded)	***Parhomonyx* Ohaus**
26	Protibia with 2 external teeth	***Pachacama* Soula**
–	Protibia with 3 external teeth	**27**
27	Labrum with apex bilobed	***Patatra* Soula**
–	Labrum with apex projecting anteriorly at middle, not bilobed	***Homeochlorota* Soula**

**Figure 1.**
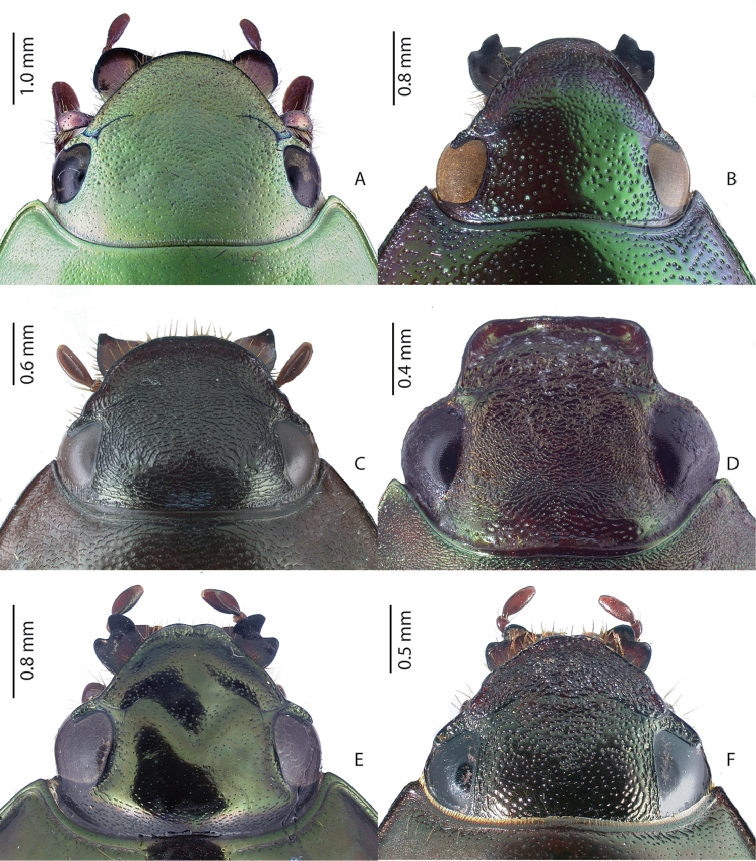
Clypeal shape varying from rounded, parabolic, trapezoidal, subquadrate (**A–D**), and emarginate (**E–F**). Lateral edge of mandibles with no reflexed teeth (lacking teeth in **A**, but the mandible is reflexed rather than flattened in **A**, two reflexed teeth (**B, E, F**), or one reflexed tooth (**C**)). Apical bead of pronotum varying from obsolete (**A**) to complete medially (**B, C, D, F**). Disc of frons with V-shaped depression (**E**) or frons planar, smooth, lacking V-shaped depression (**A–D, F**) **A** *Chrysina beyeri* Skinner **B** *Epichalcoplethis velutipes velutipes* (Arrow) **C** *Parhomonyx fuscoaeneus* (Ohaus) **D** *Chipita mexicana* (Ohaus) **E** *Sorocha* sp**. F** *Homonyx elongatus* (Blanchard).

**Figure 2.**
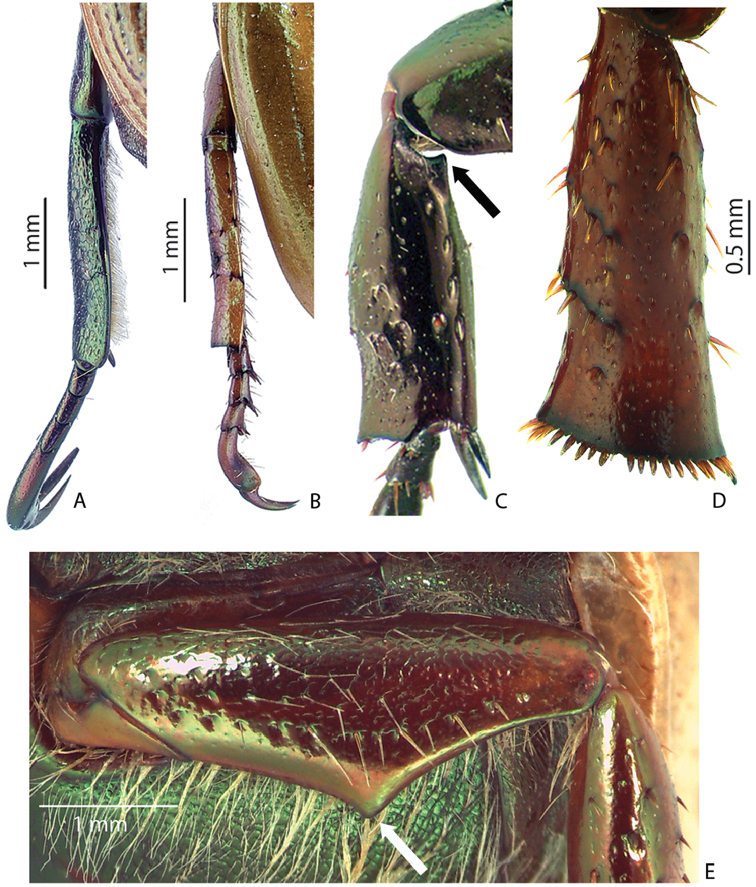
Characters of the mesofemora and metatibiae in pelidnotine genera. **A** *Epichalcoplethis aciculata* (F. Bates), metatibia somewhat flattened (dorsal view) **B** *Pelidnota virescens*, metatibia not flattened (dorsal view) **C** *Mecopelidnota* sp., metatibia at base with a semicircular notch **D** *Pseudogeniates cordobaensis* Moore et al., metatibia simple at base **E** *Mesomerodon gilletti* Soula male, acute production of posterior margin of mesotibia (ventral view).

**Figure 3.**
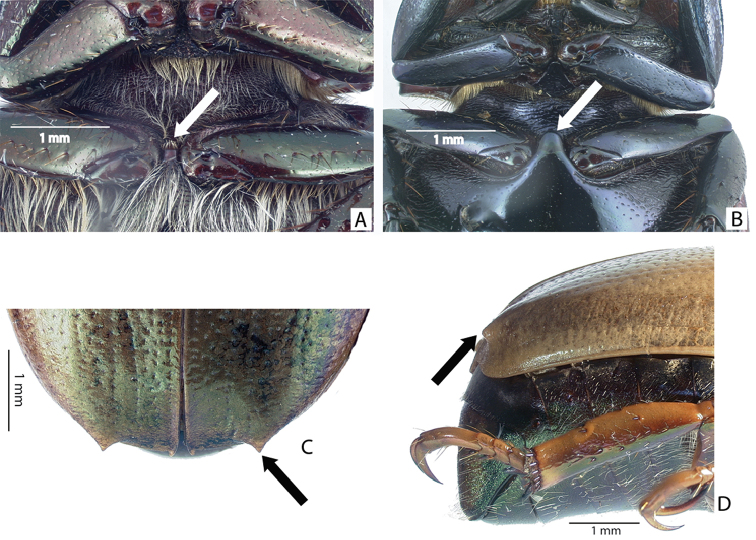
Characters of the thorax (ventral view) and elytral apex (dorsal and lateral views). Mesosternal keel not surpassing base of mesocoxae (**A**) or keel surpassing base of mesocoxae (**B**). Acute, spiniform projections at apex of elytra (**C, D**). **A** *Homonyx elongatus* **B** *Pelidnota dobleri* Frey **C** *Mesomerodon gilletti*, dorsal view **D** *Mesomerodon gilletti*, lateral view.

### Catoclastus

Taxon classificationAnimaliaColeopteraScarabaeidae

Solier, 1851

Fig. 5

#### Type species.

*Catoclastus chevrolatii* Solier, 1851.

#### Species.

3 species; length 14–23 mm.

Three species are included in this genus and are distributed in western Peru. Species are elongate-oval, metallic green with dark red appendages, and similar in overall appearance to species of *Mecopelidnota* and *Homonyx*. Soula (2010a) apparently overlooked *C. rabinovichi* Martínez, a species that is known only from the male holotype from Cusco, Peru. Species in the genus are characterized by having all claws simple; male protarsal claw with inner tubercle; bidentate mandibles; pronotum with bead complete apically, laterally and basally; elytral base with dimple lateral of scutellum; elytral epipleuron shelf-like (not rounded); fifth meso- and metatarsomeres lacking internomedial tooth; apex of the metatibia with weak corbel and with four to five spinules apically (biemarginate in *Homonyx*); mesosternal keel not surpassing mesocoxae; and metasternum with longitudinal groove (not paired as in *Hoplopelidnota*). Sister-group relationships of the genus require analysis. Specimens have been collected from 2000 to 3500 m elevation in the months of April and June. Specimens are rare in collections, and larvae are not described.

### Chalcoplethis

Taxon classificationAnimaliaColeopteraScarabaeidae

Burmeister, 1844

Fig. 6

#### Type species.

*Chrysophora kirbii* Gray, 1832.

#### Species.

2 subspecies; length 22–27 mm.

As circumscribed by Soula (2006), the genus *Chalcoplethis* includes only *C. kirbiikirbii* Gray and *C. kirbiimisionesensis* Soula. Whereas F. Bates (1904), Ohaus (1934b), and Hardy (1975) considered Pelidnota (Chalcoplethis) to include a broad group of *Pelidnota* species with metallic green, rugose elytra, Soula considered *Chalcoplethis* as unique and monotypic. Soula (2006) also considered *Epichalcoplethis* to be separate and distinct from *Chalcoplethis*. It is clear that species of *Chalcoplethis* and *Epichalcoplethis* share a number of characters (form of the male genitalia, pronotal bead obsolete apicomedially, lack of spinules at apex of metatibia, well-developed prosternal process, and mesometasternal keel surpassing the mesocoxae). Relationships of these three genera need to be studied and placed within the broader context of ruteline genera.

*Chalcoplethis kirbii* is diagnosed by its metallic green color, striate elytra, elytral epipleuron shelf-like (not rounded), pronotum with bead incomplete apically (complete laterally and basally), metatibia of the male that is strongly compressed (less so in females) and lacking apical spinules, meso- and metatarsomere 5 lacking an internomedial tooth; mandibles that are bidentate externally, prosternal process well developed, and mesometasternal keel surpassing the mesocoxae. The species is distributed in the Atlantic Coastal Forest of Brazil from Bahia in the north to Rio Grande do Sul in the south. Larvae are not described.

### Chipita

Taxon classificationAnimaliaColeopteraScarabaeidae

Soula, 2008

Fig. 7

#### Type species.

*Byrsopolis mexicana* Ohaus, 1905.

#### Species.

1 species; length 14–18 mm.

The monotypic genus *Chipita* was proposed by Soula (2008) for *Chipita mexicana* (formerly *Parhoplognathus mexicanus*), which is known from Sinaloa, Guerrero, Jalisco, Nayarit, and Oaxaca states in Mexico. Following Ohaus’s (1934b) classification of the genus *Parhoplognathus*, Soula (2008) created this monotypic genus. Several characters provide sufficient rationale for the genus: form of the thorax (broadest at base versus broadest at the middle in *Parhoplognathus*), elytra (striate versus not striate in *Parhoplognathus*), mesosternum not produced anteriorly (produced or not produced anteriorly in *Parhoplognathus*), and protibia with 2 external teeth (with 3 external teeth in *Parhoplognathus*). The taxon shares several similarities with species in the genus *Platyrutela* Bates (an anticheirine scarab), thus requiring examination within a phylogenetic framework.

*Chipita mexicana* is diagnosed by the following characters: profemur produced anteriorly and widest at middle (autapomorphic for the genus); protibia with 2 external teeth (shared with *Platyrutela*); mandibular palp with deep, horizontal sulcus (shared with *Platyrutela*); clypeus quadrate and apex reflexed (shared with *Platyrutela*); clypeus greatly declivous with respect to plane of frons; pronotum broadest at base (shared with *Platyrutela*); elytra striate; elytral epipleuron rounded; claws simple on all legs (male and female; shared with *Platyrutela*); male protarsal and mesotarsal claws with inner, apical tubercle; meso- and metatarsomere 5 with internomedial tooth; apex of metatibia with short spines (versus long setae in *Platyrutela*); color gray or castaneous with or without metallic green sheen.

Adult *C. mexicana* inhabit tropical deciduous and sub-deciduous forests at elevations between sea level and 200 m (Morón et al. 1997). Adults are temporally distributed between June and November, and are attracted to lights at night (Morón et al. 1997). The larvae of *C. mexicana* are undescribed and their biology is unknown. Male specimens of *Chipita mexicana* are rare in collections, and may be indicative of unusual natural history.

### Chrysina

Taxon classificationAnimaliaColeopteraScarabaeidae

Kirby, 1828

Figs 1A , 8 , 9 , 10 , 11 , 12

#### Type species.

*Chrysina peruviana* Kirby, 1828.

#### Species.

113 species; length 19–40 mm.

Species in the genus *Chrysina* are commonly known as the “jewel scarabs” for their spectacular metallic and iridescent coloration and large size. Species range from metallic green, pink, purple, gold, and silver, and their elytra may be adorned with metallic gold or silver pin stripes or polka dots. The males of some species have enlarged metafemora (e.g., *Chrysina macropus* [Francillon]). Morón (1990) reviewed the 73 species of *Chrysina* (then referred to as *Plusiotis, Chrysina*, and *Pelidnotopsis*). Since that time, an additional 40 species have been described, and no updated revision or monograph is available for the group. The following characters serve to diagnose species in the genus: clypeal apex rounded, with or without emargination; all claws simple; male protarsal claw with or without inner tubercle; mandibles rounded externally; pronotum with bead incomplete apically and basally (complete laterally) (*Chrysophora* with bead complete on all margins); elytral epipleuron shelf-like (not rounded); fifth meso- and metatarsomeres with internomedial tooth; metatarsi shorter than tibia (longer than tibia in *Chrysophora* and *Chalcoplethis*); apex of the metatibia with or without corbel; meso- and metatarsomere 5 with internomedial tooth; mesosternal keel surpassing mesocoxae.

The genera *Plusiotis* and *Chrysina* were historically separate genera. Morón and Howden (1992) noted an apparent grade of characters within the taxa. Based on molecular and morphological data, Hawks (2001) synonymized *Plusiotis* as well as *Pelidnotopsis* with *Chrysina*. Soula (2008) resurrected the genus *Pelidnotopsis*, asserting that the genus was “closer” to *Pelidnota* than to *Chrysina*. Moore and Jameson (2013) again synonymized *Pelidnotopsis* within *Chrysina*. In an effort to develop identification tools for species of conservation importance, Moron and Noguiera (2016) advocated for the use of both *Plusiotis* and *Chrysina*. Although they acknowledge that several species possess “transitional characters”, they argue that the evidence for synonymy of *Plusiotis* was based on unpublished data (Hawks 2001). Characters, they assert, clearly differentiate the two genera, but they do not provide a list of these characters nor a diagnosis for each genus. In our view, the transitional characters provide support for one clade, thus we advise the unity of these genera into the senior name, *Chrysina*. An analysis in preparation by Morón will elucidate the relationships of the genera (Morón and Noguiera 2016).

Species in the genus are distributed from the southwestern United States to Ecuador with the greatest diversity of species occurring between 1000–2000 m elevation (Morón 1991). Many species have narrow habitat requirements and are negatively impacted by unfaltering deforestation that serves to reduce and isolate populations, thus placing species at risk (Morón and Nogueira 2016). Species are found in primary forests (pine, juniper, and pine-oak) between 50-3800 m. Species feed on the foliage (adults) or rotting logs (larvae) of various trees including species in the genera *Abies, Alnus, Arbutus, Heliocarpus, Juglans, Juniperus, Liquidambar, Pinus, Platanus, Quercus*, and *Turpinia* (Morón 1991). Representative larvae have been described in the genus (Ritcher 1966, Morón 1976, 1985). Adults are frequently attracted to lights, and larvae live in rotten logs.

### Chrysophora

Taxon classificationAnimaliaColeopteraScarabaeidae

Dejean, 1821

Fig. 13

#### Type species.

*Melolontha chrysochlora* Latreille, 1812.

#### Species.

1 species; length 25–42 mm.

The dazzling, metallic green *Chrysophora chrysochlora* is a distinctive species and the only member of its genus. The large size, conspicuously rugose elytra, and elongate legs of the male are distinguishing characteristics. Additional characters include the metatibia of the male that is prolonged and acuminate at the apex, the 5^th^ tarsomere with an internal tooth (all legs), the mandibles that are broadly rounded externally, the pronotum with a complete bead, and the mesosternum that is not appreciably produced beyond mesometasternal suture. Research is needed to examine sister-group relationships of this monotypic genus.

The species is distributed in Colombia, Ecuador, and Peru where the Jivaro, Shuar, and Sequoia Indians use the elytra, pronota, legs, or entire body for adornment (Ratcliffe 2006, Ratcliffe et al. 2015, Le Tirant and Limoges 2016). The species is primarily found in dry and humid tropical forest between 180-550 m elevation (Pardo-Locarno and Morón 2007), although the species is recorded between 500-1000 m elevation (Morón 1990). The species is associated with *Buddleja* L. (Scrophulariaceae), *Gynerium sagittatum* (Aubl.) Beauvois (Poaceae; arrow cane, wild cane) (both Ohaus 1934b), *Senna reticulata* (Willd.) H. S. Irwin and Barneby (Leguminosae), and *Leucaena leucocephala* (Lam.) de Wit (Leguminosae) (both Pardo-Locarno and Morón 2007). Adults feed on the leaves of *G. sagittatum* during the day (Ohaus 1934b) from February to May (Morón 1990), and they fly at twilight or at night (Ohaus 1934b). Larvae and pupae are described and share several characters with *Pelidnota* larvae and pupae (Pardo-Locarno and Morón 2007).

### Ectinoplectron

Taxon classificationAnimaliaColeopteraScarabaeidae

Ohaus, 1915

Fig. 14

#### Type species.

*Homonyx oryctoides* Ohaus, 1905.

#### Species.

1 species; length 21–23 mm.

This monotypic genus is endemic to northwestern Mexico. Adults have a rufous dorsal coloration without metallic reflections, and are similar to Pelidnota (Pelidnota) in overall appearance. Adults in the genus *Ectinoplectron* are diagnosed by the disc of the prosternal peg that is weakly concave with reflexed margins (an autapomorph). Additional diagnostic characters include: lateral edge of mandibles with two reflexed teeth; apex of metatibia straight (not biemarginate) and lacking spinules or setae; meso- and metatarsomere 5 lacking an internomedial tooth; mesosternum not appreciably produced beyond the mesometasternal suture; pronotum with bead complete apically, basally, and laterally; lateral edge of protibia with three rounded teeth; and, apex of clypeus subtrapezoidal to subtriangular.

*Ectinoplectron oryctoides* is known from Pacific coastal states of Mexico (Durango, Jalisco, Michoacán, Nayarit, Sinaloa and Sonora), northern Chihuahua (Lugo et al. 2011), and western Durango (Machatschke 1972, Hardy 1975, Morón 1990) where it occupies tropical deciduous forests of oak and pine (Morón et al. 1997). Temporal distribution is from late June to September (Morón et al. 1997). Individuals of *E. oryctoides* occur from sea level to 2000 m elevation (Hardy 1975, Morón et al. 1997), are attracted to lights at night, and tend to fly near dusk (Morón et al. 1997). Larvae of *E. oryctoides* are undescribed.

### Epichalcoplethis

Taxon classificationAnimaliaColeopteraScarabaeidae

F. Bates 1904

Figs 2A , 15 , 16 , 17 , 18 , 19

#### Type species.

*Pelidnota velutipes* Arrow, 1900.

#### Species.

16 species and subspecies; length 15–19 mm.

Previously considered a subgenus of *Pelidnota*, *Epichalcoplethis* was circumscribed by Soula (2006) as distinct from the monotypic genus *Chalcoplethis* and composed of 16 species and subspecies. *Chalcoplethis kirbii* shares many characters with species of *Epichalcoplethis* including form of the male genitalia, pronotal bead which is obsolete apicomedially, lack of spinules at apex of metatibia, well-developed prosternal process, and mesometasternal keel surpassing the mesocoxae. Sister-group relationships require examination.

Species in the genus *Epichalcoplethis* can be diagnosed, in part, based on the following characters: metatibia weakly compressed (strongly compressed in *C. kirbii*) and apex lacking spinules; meso- and metatarsomere 5 lacking internomedial tooth; punctate-striate elytra; elytral epipleuron shelf-like (not rounded); pronotum with bead incomplete apically (complete laterally and basally); mandibles that are bidentate externally; prosternal process well-developed; and, mesometasternal keel surpassing the mesocoxae. *Epichalcoplethis chamaeleon* (Herbst) differs from other species in the genus based on the form of the male parameres, form of the metatibia in the male (not compressed laterally and apex with a well-developed corbel). For many years, this large and conspicuous species was misidentified as *Pelidnota rostrata* Burmeister.

Species in the genus are distributed from Guatemala and Belize, St. Vincent and the Grenadines, Trinidad and Tobago, and south to Argentina, Uruguay, and Paraguay. In Grenada, *E. velutipes* is common in the temperate zone from April to May (Chalumeau 1985). Although the larvae are not described for this species, Chalumeau (1985) noted that he obtained larvae from the decaying trunks of mango trees.

### Eremophygus

Taxon classificationAnimaliaColeopteraScarabaeidae

Ohaus, 1910

Figs 20 , 21 , 22 , 23 , 24

#### Type species.

*Eremophygus philippii* Ohaus, 1910.

#### Species.

6 species; length 14–15 mm.

Rarity of specimens in collections as well as possible paraphyly with the genera *Oogenius*, *Microogenius*, *Peruquime*, and *Lasiocala* hampers our understanding of the biodiversity of this group. Species in the genus *Eremophygus* are distributed in the altiplano of Bolivia, Argentina, Peru, and Chile. Gutiérrez described two species in the genus (Gutiérrez 1951, 1952), discussed the genus (Gutiérrez 1949, 1950, 1951, 1952), and provided the most recent key to species (Gutiérrez 1952), yet he did not discuss the group’s relationships or context within the Rutelinae.

Some species in the genus lack the independently movable claws that are diagnostic of Rutelinae (that is, the apex of meso- and metatarsomere 5 lack a longitudinal slit, a character suite shared with cyclocephaline rhinoceros beetles [Dynastinae: Cyclocephalini]). One species, *Eremophygus pereirai* Martínez (from Jujuy, Argentina), was transferred to the dynastine tribe Cyclocephalini and the genus *Cyclocephala* by Martínez (1975b) who compared the toothless maxillary galea of *E. pereirai* to the similar maxilla in *Cyclocephala zischkai* Martínez from Bolivia (Martínez 1960, 1965). Endrödi (1977) agreed with the tribal transfer and also compared *E. pereirai* to *C. zischkai*, considering these species distinctive enough so that, together, they could warrant subgeneric status within *Cyclocephala*. *Eremophygus pereirai* (as *C. pereirai*) was later included in the key to world Dynastinae and Cyclocephalini (Endrödi 1985). Krajcik (2012) included *E. pereirai* under *Cyclocephala* following Endrödi. *Cyclocephala zischkai* and *C. pereirai* have male parameres that are formed from two, laterally articulated plates, a character associated with Cyclocephalini and not Rutelini (male parameres are fused into a single plate that is not laterally articulating). This represents another example of genera historically considered to be part of Rutelini (e.g., *Peltonotus* and *Acrobolbia* Ohaus) that were later transferred to Cyclocephalini. This highlights the need for phylogenetic analyses including *Eremophygus* to broadly sample taxa from Cyclocephalini and Rutelini to resolve the tribal, and thus the subfamilial, placement of this genus.

Diagnostic characters have greatly diminished reliability because of overlap with *Lasiocala, Oogenius*, *Peruquime*, *Microogenius*, and *Cyclocephala* and should be used with great caution: dorsal surface often with long, tawny setae; apex of labrum extends beyond clypeal apex, visible in dorsal view; antenna 9- or 10-segmented (9-segmented according to Mondaca and Valencia [2016]); lateral edge of mandibles rounded and without reflexed teeth; apex of clypeus varies from rounded to subtrapezoidal; pronotum with apical bead complete medially, laterally, and basally; lateral edge of protibia with three rounded teeth; apex of fourth metatarsomere lacking spiniform attenuation; base of metatibia nearly straight, lacking distinct notch; apex of meso- and metatibia with many spinules, and; mesosternum not appreciably produced beyond the mesometasternal suture. In some species (e.g., *E. lasiocalinus* Ohaus), the protarsal claw is enlarged and deeply split; the meso- and metatarsal claws may be deeply split or simple; the unguitractor plate of meso- and metatarsus is subcylindrical with 2 or 3 setae; and the apex of tarsomere 5 (meso- and metatarsus) with 2 weak, longitudinal slits at apex (a character that is not shared by most other Rutelini; instead it is more common in the Melolonthinae and Dynastinae). Larvae, natural history, and sister-group relationships are not known.

### Homeochlorota

Taxon classificationAnimaliaColeopteraScarabaeidae

Soula, 2006

Fig. 25

#### Type species.

*Pseudochlorota chiriquina* Ohaus, 1905.

#### Species.

1 species; length 18–20 mm.

The monotypic genus *Homeochlorota* is rarely encountered in collections and is narrowly distributed in Costa Rica and Panama. As the generic name implies, the genus shares similarities with the genus *Chlorota* (an anticheirine leaf chafer) including the form of the metatibia (with emargination at apex and with external apex posteriorly produced), form of the claws (widely toothed), and metamesosternal peg that is produced ventrally. In general appearance, it could be confused with *Chlorota flavicollis* Bates. Analyses should closely examine relationships with *Chlorota* and other anticheirine leaf chafers in combination with lasiocaline and pelidnotine chafers.

The ruteline genera *Pseudochlorota* and *Lasiocala* comprise the subtribe (Ohaus 1934b). Soula (2006) abandoned the subtribe because “it clearly is not monophyletic” (translated from French) (Soula 2006: 144), then reinstituted it without reason (Soula 2011). Soula (2006) observed that the species of *Pseudochlorota* possessed “some similarities” as well as many characters that separate them. On this basis, Soula (2006) transferred *Pseudochlorota chiriquina* into the genus *Homeochlorota*, creating this monotypic genus.

The taxon is characterized by the following features: pronotum with apical bead lacking or obsolete medially; mesosternum not appreciably produced beyond metamesosternal suture; metamesosternal peg produced ventrally; lateral edge of mandible with one reflexed tooth; labrum extends beyond apex of the clypeus; apical margin of the labrum arcuate and with a small tooth at the middle; frontoclypeal suture obsolete; metatibia with emargination at apex and with external apex posteriorly produced, and; larger claw on all legs widely cleft (shared with *Lasiocala*). Natural history and larvae are not known, and sister-group relationships have not been examined.

### Homonyx

Taxon classificationAnimaliaColeopteraScarabaeidae

Guérin-Méneville, 1839

Figs 1F , 3A , 26 , 27 , 28 , 29 , 30 , 31 , 32 , 33

#### Type species.

*Homonyx cupreus* Guérin-Méneville, 1839.

#### Species.

14 species and subspecies; length 12–19 mm.

Species in the genus *Homonyx* are elongate, parallel-sided, subcylindrical, and dark-colored beetles. They strongly resemble the allied genus *Parhomonyx* but can be separated based on the form of the mandibles (bidentate in *Homonyx* and broadly rounded with one apical tooth in *Parhomonyx*), the apex of the metatibia (with many spinules in *Parhomonyx* and biemarginate in *Homonyx*), and the feathery fringe of setae at the apex of the elytra (exposed in *Parhomonyx*; hidden in *Homonyx*). These genera share additional characters: prosternal process short (well-developed in *H. planicostatus*); mesosternum not produced beyond the mesometasternal suture; pronotum with bead complete apically, laterally, and basally; claws simple; lateral set of setae on apical edge of 3^rd^ metatarsomere of unequal length and width (versus equal in length and width in *Catoclastus*).

Species in the genus are distributed in Argentina, Brazil, Bolivia, Ecuador, Uruguay, and Peru. Soula (2010a) provides the most current treatment of species in the genus, but did not include a key for identification. Larvae, sister-group relationships and natural history are poorly known for species in the genus.

### Homothermon

Taxon classificationAnimaliaColeopteraScarabaeidae

Ohaus, 1898

Figs 34 , 35

#### Type species.

*Homothermon bugre* Ohaus, 1898.

#### Species.

4 species; length 9–19 mm.

The genus *Homothermon* includes four uncommon species that are distributed in the Paulista center of endemism in Brazil and Argentina (Rio de Janeiro in the north to Santa Catarina, Rio Grande do Sul, and Misiones in the south) (Müller 1973). Species in the genus are characterized by greatly enlarged metatibia in the male, claws on all legs simple (both male and female), male with a medial tubercle on the protarsal claw, pronotal basal bead incomplete anterior to the scutellum, elytral margin without a bead (=rounded), scutellum that is nearly twice as wide as long, clypeus semi-circular or subtrapezoidal, apex of mandibles bidentate, and parameres with a well-developed ventral plate.

Classification and nomenclatural history of members of the genus has been confused. Ohaus (1898) postulated that the genus was closely related to *Thyridium* Burmeister and placed it in the subtribe . Bates (1904) omitted the genus *Homothermon* (even though it was described six years prior to his revision) and also overlooked *Homothermon praemorsus* (Burmeister) (then classified as *Strigidia praemorsus*) even though he treated *Strigidia* as a valid genus. In the *Coleopterorum Catalogus* (Ohaus 1898, 1918), the genus was placed in the subtribe . Soula (2008) commented that the genus “approaches” *Pelidnota*. Based on the elytral margin that lacks a bead, it is possible that the genus may be allied with *Plesiorutela* Jameson (Rutelini). Future research should examine the relationships of *Homothermon*, placing it within a broad context of Rutelini.

*Homothermon serrano* Ohaus and *H. bugre* are apparently sympatric. Based on our examination of specimens (including type specimens), the two species differ only in color but are conspecific in all other respects. Soula (2008) maintained *H. serrano* and *H. bugre* as separate species, and he stated that these are “good examples of two populations where the aedeagus is very similar, however the populations represent two good species that are sympatric” (Soula 2008: 31; translated from French). Lacking additional character evidence, we think that this is open to interpretation.

Natural history for the genus is unknown. *Homothermon serrano* is known from the forested mountains near Theresopolis, Santa Catarina, Brazil (Ohaus 1898).

### Hoplopelidnota

Taxon classificationAnimaliaColeopteraScarabaeidae

F. Bates, 1904

Figs 36 , 37

#### Type species.

*Hoplopelidnota candezei* F. Bates, 1904.

#### Species.

1 species; length 19–24 mm.

The monotypic genus *Hoplopelidnota* is rarely found in collections. Similar to species of *Chalcoplethis* and *Catoclastus*, it possesses metallic green, rugose elytra. Prior to this work, females were not associated with males. The elytral callus of the male possesses a well-developed spine (shared with the pelidnotine genus *Mesomerodon*; lacking in females of both *Mesomerodon* and *Hoplopelidnota*). In addition to the spinose elytra, several unusual characters serve to diagnose the genus: fringe of setae produced beyond apex of elytra; metatibial apex straight (lacking a corbel); mesosternum produced beyond the mesometasternal suture; metasternum with two parallel, longitudinal furrows; pygidium of female with a well-developed horizontal ridge and weak discal concavity; terminal sternite in the female with two deep emarginations on either side of the apex.

The genus includes one species, *Hoplopelidnota metallica* (Laporte), which has a turbulent nomenclatural history (see “Annotated Catalog”; Moore and Jameson 2013). No analyses have examined the relationships of the genus to other rutelines. *Hoplopelidnota metallica* is distributed in northern South America, and we provide country records for Brazil, Guyana, and Venezuela.

### Mecopelidnota

Taxon classificationAnimaliaColeopteraScarabaeidae

F. Bates, 1904

Figs 2C , 38

#### Type species.

*Mecopelidnota arrowi* F. Bates, 1904.

#### Species.

8 species; length 17–26 mm.

Species in the genus *Mecopelidnota* are distinctive for their dark metallic green coloration, large size, elongate body form, and emargination at the base of the metatibia in the male. As currently constituted, the genus includes eight species (Soula 2008), but this may be an over-estimate. Members are distributed both on the east and west sides of the Andes in Colombia, Ecuador, and Peru. The record for *M. cylindrica* from Guatemala is questionable (Monzón 1996).

The form of the male metatibia (base with an emargination) serves as a synapomorph for the group. Based on our analyses of external morphological characters, the genus includes two lineages: one to the west side of the Andes and one on the east of the Andes. Species on the west of the Andes (*M. arrowi, M. cylindrica* (Waterhouse), *M. marxi* Soula, and *M. obscura* [Taschenberg]) share the form of the male parameres (with enlarged “thumb” in lateral view) and greatly enlarged female gonocoxites. Species on the east side of the Andes (*M. witti* Ohaus, *M. gerardi* Soula, *M. mezai* Soula, and *M. dewynteri* Soula) share the form of the male parameres (lacking the enlarged “thumb” in lateral view) and reduced female gonocoxites. Both lineages exhibit north-south clinal variation in the form of the male parameres, and Soula (2008: 23) also alludes to this “transitional” variation in species on either side of the Andes.

Species in the genus are recorded from less than 10 m elevation (*M. cylindrica* and *M. obscura*; Paucar-Cabrera 2005) to 2700 m (*M. obscura*). Ohaus (1908b) recorded *M. arrowi* in the flowers of yellow *Mimosa* sp. (Leguminosae) in Guayaquil, Ecuador, during the rainy season. In Ecuador, species were collected January to April in tropical regions (Paucar-Cabrera 2005). Larvae and sister-group relationships are not known.

### Mesomerodon

Taxon classificationAnimaliaColeopteraScarabaeidae

Ohaus, 1905

Figs 2E , 3C, D , 39 , 40

#### Type species.

*Mesomerodon spinipenne* Ohaus, 1905.

#### Species.

2 species; length 17–24 mm.

The genus *Mesomerodon* includes two species that are distributed in Colombia, Ecuador, Peru, and Bolivia (Machatschke 1972, Soula 2008). Members are sexually dimorphic, with males having an acute, spiniform processes on the apical callus of the elytra (shared with *Hoplopelidnota*) and an acute process on the posterior margin of the mesofemur. Species in the genus are testaceous in color, ovate-shaped, and similar in overall gestalt to species of Pelidnota (Pelidnota). The genus is diagnosed by the following additional characters: lateral edge of mandibles without reflexed teeth; lateral edge of protibia with three teeth; pronotum with apical bead complete medially, laterally and basally; mesosternum produced beyond mesometasternal suture; male parameres with a well-developed ventral plate.

The biology of *Mesomerodon* species is unknown and larvae are not described; sister-group relationships have not been examined. Adults are collected at lights at elevations between 300–750 m.

### Microogenius

Taxon classificationAnimaliaColeopteraScarabaeidae

Gutiérrez, 1951

Figs 41 , 42 , 43

#### Type species.

*Oogenius martinezi* Gutiérrez 1951.

#### Species.

4 species; length 10–13 mm.

The classification and nomenclatural history of this genus are quite complicated due to two impediments: lack of robust circumscription of ruteline groups and access to literature. Historically, the genus *Microogenius* was considered a member of the subtribe and closely related to *Lasiocala* Blanchard (Martínez 1974) as well as a member of the subtribe closely related to *Eremophygus* and *Oogenius* (Ohaus 1934b). Based on similarities, the two subtribes were combined (Martínez 1974), but this publication was effectively lost until Soula (2006) noted Martínez’s synonymy of Oogenius (Microogenius) and created the new genus *Minilasiocala* Soula. However, based on the Principle of Priority, *Microogenius* should be considered the valid name (Moore and Jameson 2013). Similar problems circumscribing ruteline groups led Soula to initially consider the taxon a lasiocaline scarab (Soula 2006) and later to consider it a pelidnotine scarab (Soula 2011). Clearly, phylogenetic and revisionary research must examine relationships of the South American genera *Microogenius, Oogenius*, *Eremophygus*, and *Lasiocala*.

Although the validity of the genus requires evaluation, a few characters can be used with caution for diagnosis: apex of labrum extends beyond clypeal apex and visible in dorsal view (shared with *Eremophygus* and *Oogenius*); metatarsomere 4 at apex with 4-6 long setae that are subequal in length and thickness; mandible on external margin rounded (shared with *Eremophygus*); pronotal basal bead complete (shared with *Eremophygus*); terminal tergite of female rounded at apex (shared with *Eremophygus*).

As currently composed, species in the genus are distributed in the altiplano of Bolivia and Argentina. Larvae, natural history, and sister group relationships are not known.

### Neogutierrezia

Taxon classificationAnimaliaColeopteraScarabaeidae

Martínez, 1953

Fig. 44

#### Type species.

*Neogutierrezia mirabilis* Martínez, 1953.

#### Species.

10 species; length 6–9 mm.

Similar to *Peruquime*, the genus *Neogutierrezia* is a difficult-to-place taxon with affinities to both Melolonthinae and Rutelinae. Molecular and morphological phylogenetic analyses provided strong evidence that the genus is closely related to members of the Rutelinae, thus *Neogutierrezia* was transferred from Melolonthinae to Rutelinae (Ocampo et al. 2010). The recent discovery of *Peruquime* and comparison with *Neogutierrezia* and *Eremophygus* establishes an association with pelidnotine chafers, thus our rationale for including the genus herein.

The genus *Neogutierrezia* is endemic to the Monte biogeographic province in Argentina (Mendoza, Río Negro, Neuquén, Chubut), a shrub steppe region and that coincides with the distribution of *Larrea* spp., *Bulnesia* spp., and *Plectocarpha* spp. (all Zygophyllaceae) (Ocampo et al. 2010). The genus is hypothesized to be a relictual ruteline group that evolved and adapted *in situ* to the extreme arid conditions of the desert sand dunes (Ocampo et al. 2010).

The genus is diagnosed by the following characters: antennal club longer than stem, and club 3- or 4-segmented (3-segmented in *Peruquime*); labrum kidney-shaped; pygidial apex “recumbent towards metacoxae” in males; parameres with dorsal and ventral plates fused. Other characters include: frontoclypeal suture complete or obsolete at middle; pronotal apical bead obsolete at middle, complete laterally and basally; and all claws simple.

Species are associated with sandy habitats (sea shores, dunes), and females of one species (*N. araucana* Martínez) are known to be flightless, probably living underground and only coming to the surface to mate (Martínez 1973). Adults have been collected at light (UV and kerosene lamp) and with un-baited pitfall traps. An identification key to species is available (Ocampo et al. 2010). Larvae are not known.

### Oogenius

Taxon classificationAnimaliaColeopteraScarabaeidae

Solier, 1851

Figs 45 , 46 , 47 , 48 , 49

#### Type species.

*Oogenius virens* Solier, 1851.

#### Species.

7 species; length 12–23 mm.

Species in the genus *Oogenius* are egg-shaped (from which the generic name was derived) and distributed in Chile and Argentina. Based on prevailing usage of the name, Mondaca (2005) corrected the spelling of the genus from “Oogeneius” to *Oogenius* and provided a catalog of included species. *Oogenius* was later revised by Mondaca (2016), and this work provided a comprehensive key to species, a distribution map, high-quality images of diagnostic characters, and additional biological information.

Circumscription of the genus and phylogenetic analyses that include *Microogenius, Eremophygus*, and *Lasiocala* are necessary to better understand the composition of the genus and sister group relationships. Although Soula (2006) treated the genera *Lasiocala* and *Microogenius* (or *Minilasiocala* by Soula 2006), he omitted the genera *Oogenius* and *Eremophygus*. Species in these groups possess a broad overlap in characters and many species are quite rare in collections.

The genus *Oogenius* can provisionally be identified based on the following characters: pronotum with basal bead obsolete or complete medially, complete laterally and apically; clypeus broadly rounded apically, reflexed; labrum produced beyond apex of clypeus; mandibles broadly rounded externally; inner claw enlarged and weakly split in male; unguitractor plate subcylindrical; 5^th^ meso- and metatarsomeres lacking medial tooth; mesosternum not appreciably produced beyond mesometasternal suture; and ventral surface densely setose.

The immature stages of *Oogenius* have not been described, but Mondaca (2016) reported that larvae feed on roots and decaying plant matter in three species: *O. castilloi* Martínez and Peña, *O. chilensis* Ohaus, and *O. virens* Solier.

### Pachacama

Taxon classificationAnimaliaColeopteraScarabaeidae

Soula, 2006

Fig. 50

#### Type species.

*Pachacama ocampoi* Soula, 2006.

#### Species.

2 subspecies; length 15–17 mm.

As noted by Soula (2006; translated from French) in his description of this unusual genus, “cladistics or molecular analysis is needed more than ever.” Soula (2006) included this lustrous, dark green chafer in the pelidnotine scarabs, and he noted characters that it shared with *Minilasiocala* (now a junior synonym of *Microogenius*) and *Chrysophora*. Phylogenetic analyses are needed to address sister-group relationships of this taxon. *Pachacama ocampoi* possesses unusual autapomorphs (prosternum produced anteriorly, mesosternum posterior to prosternal peg with transverse fold), and it is possible that it is more closely related to some anticheirine scarabs.

*Pachacama* can be diagnosed based on the following characters: dorsal surface smooth (lacking striae, obvious punctures or rugosity); clypeus elongate with parabolic apex (subequal in length and width); external margin of mandible bisinuate with apical tooth reflexed; pronotum with apical bead incomplete at middle (bead complete medially and basally); protibia with 2 external teeth; apex of metatibia produced on external margin; metatarsus 1 short (half the length of metatarsus 2); metacoxal corner produced, acute; mesosternum appreciably produced beyond metamesosternal suture; protarsal claws of male with internal claw enlarged, split (female split); meso- and metatarsal claws simple in male (widely split in female); 5^th^ tarsomeres (all legs) with medial tooth; uncus subcylindrical, tapering at apex.

*Pachacama ocampoi* is endemic to Ecuador where it is recorded between 500 to 1650 m elevation in the provinces of Cañar and Pichincha. Natural history and larvae are unknown.

### Parhomonyx

Taxon classificationAnimaliaColeopteraScarabaeidae

Ohaus, 1915

Figs 1C , 51

#### Type species.

*Homonyx fuscoaeneus* Ohaus, 1905.

#### Species.

1 species; length 17–22 mm.

The monotypic genus *Parhomonyx* is endemic to northern Argentina. Ohaus (1905) described *P. fuscoaeneus* in the genus *Homonyx* and in conjunction with another unusual pelidnotine, *E. oryctoides* (originally *Homonyx orcytoides*). According to Ohaus (1915b), the genera *Homonyx* and *Parhomonyx* were closely related, and differences in the mandibular form (apex with two teeth in *Homonyx* versus apex rounded in *Parhomonyx*) provide character support for both genera (=lineages). Species in the pelidnotine genera *Parhomonyx, Homonyx*, and *Pseudogeniates* are distributed primarily in the southern half of South America. Ohaus (1915b) considered *Parhomonyx* to be an “intermediate stage” that “led *Homonyx* to *Pseudogeniates*” (Ohaus 1915b: 258), and that characters such as coloration, clypeus, mouthparts, elytra, metatibia, and antennae indicated a progression of forms (Jameson and Ocampo 2012). Additional research should examine sister-group relationships of the taxon.

*Parhomonyx fuscoaeneus* is castaneous-bronze in color and is diagnostic for its rounded mandibular apex with apical tooth (shared with *Pseudogeniates*; bidentate in *Homonyx*); metatibial apex with many spinules (biemarginate in *Homonyx*); elytral apex with a fringe of setae (shared with *Pseudogeniates*; hidden in *Homonyx*); protibia lacking weak, basal notch; all claws simple; fifth meso- and metatarsomeres with one or two internal teeth (lacking in *Pseudogeniates*, shared with *Homonyx*); lateral set of setae on apical edge of 3^rd^ metatarsomere of equal length and width (versus unequal length and width in *Pseudogeniates*); pronotum with bead complete apically, laterally, and basally; prosternal process short; mesosternal peg lacking (shared with *Homonyx* and *Pseudogeniates)*; elytra striate (shared with *Pseudogeniates* and *Homonyx*); and body form elongate and parallel-sided (shared with *Homonyx* and *Pseudogeniates)*. Larvae are not described. Label data indicate that specimens are collected at blacklight.

### Parhoplognathus

Taxon classificationAnimaliaColeopteraScarabaeidae

Ohaus, 1915

Fig. 52

#### Type species.

*Areoda maculata* Gory, 1833.

#### Species.

4 species; length 12–16 mm.

On first glance, the Brazilian Atlantic Coastal forest endemic genus *Parhoplognathus* appears similar to areodine leaf chafers such as *Areoda* MacLeay or *Byrsopolis* Burmeister due to their strongly convex form (in lateral view) and the apex of the metatibia that possesses many spinules. However, whereas areodine chafers possess a complete frontoclypeal suture, species in the genus *Parhoplognathus* have an obsolete frontoclypeal suture.

Ohaus (1934b) considered *Chipita mexicana*, to be a member of the genus *Parhoplognathus*, but several morphological characters (in addition to the disjunct distribution) provide rationale for the monotypic genus *Chipita*. The genera *Parhoplognathus, Chipita*, and *Platyrutela* (an anticheirine leaf chafer) share several similarities, and phylogenetic analyses could examine this overlap.

Species in the genus *Parhoplognathus* are diagnosed by the following characters: pronotum with apical bead obsolete or lacking medially; clypeal apex quadrate, reflexed, with or without emargination; external edge of protibia with 3 teeth; all claws simple (shared with *Chipita* and *Platyrutela*); mandibular palp with horizontal/longitudinal sulcus.

A synopsis of the species in the genus was provided by Soula (2008), but identification key, natural history, and general distributional information were omitted. Natural history, larvae, and sister-group relationships have not been examined for any species in the genus.

### Patatra

Taxon classificationAnimaliaColeopteraScarabaeidae

Soula, 2008

#### Type species.

*Patatra mathani* Soula, 2008.

#### Species.

1 species; length 15.5 mm.

*Patatra mathani* is metallic green, the internal protarsal claw is widely toothed and other claws are simple, and the parameres share some similarity to species of *Chlorota* Burmeister or *Pseudothyridium* Soula (anticheirine scarabs). Soula (2008, 2009) described this monotypic genus based on one male specimen from Pará (Brazil), and he placed it in the tribe Rutelini. He created a case of double homonymy by describing the genus and species identically in two publications (Soula 2008, 2009, see Moore and Jameson 2013). He noted that the taxon possessed characters of both pelidnotines scarabs (complete pronotal basal bead) as well as anticheirine scarabs (scutellum wider than long in the middle, male medial tarsal claws split and other claws simple). The genus ultimately was classified in the pelidnotine scarabs (Soula 2011), but characters that supported this were not provided. The genus was not included in any generic keys, and Soula’s descriptions do not provide adequate characters for separation from other genera. Future research should address the classification and relationships of *Patatra mathani*. Larvae and natural history for the species are not known.

### Pelidnota

Taxon classificationAnimaliaColeopteraScarabaeidae

MacLeay, 1819

Figs 2B , 3B , 53 , 54 , 55 , 56 , 57 , 58 , 59 , 60 , 61 , 62 , 63 , 64 , 65 , 66 , 67 , 68 , 69 , 70 , 71 , 72 , 73 , 74 , 75 , 76 , 77 , 78 , 79 , 80 , 81 , 82 , 83 , 84 , 85 , 86 , 87 , 88 , 89 , 90 , 91 , 92 , 93 , 94 , 95 , 96 , 97

#### Type species.

*Scarabaeus punctatus* Linnaeus, 1758.

#### Species.

195 species and subspecies; length 11–37 mm.

From southeastern Canada to Argentina and the Caribbean, members of the genus *Pelidnota* are obvious members of the entomofauna with diverse forms (some with enlarged metafemora such as *P. burmeisteri*), diverse colors (from metallic silver in *P. teocuitlamayatli* Delgado-Castillo, Deloya, and Morón to shiny red and blue in *P. rubripennis riedeli* [Ohaus]), and diverse maculations (striped green and tan in *P. liturella* [Kirby] or colorfully spotted in *P. xanthospila* [Germar]). Their large size, abundance, and beauty make them fairly recognizable. Some species are recognized as pests: *P. filippiniae* Soula, which defoliates plantations (Lunz et al. 2011) and *P. punctata* that feeds on leaves in vineyards (Ratcliffe and Paulsen 2008). Complete life cycle and representative larvae are described (Ritcher 1945, 1966, Morón 1976, Morón and Deloya 2002, Rodriguez et al. 2012, Garcia et al. 2013).

Hardy (1975) revised the genus *Pelidnota* from North and Central America and provided a key to species. He geographically restricted his revision to North and Central American species due to the large size of the group. The work stabilized the classification of North and Central American taxa and provided the only method of accurately identifying species in this region. He did not discuss relationships among the subgenera of *Pelidnota*, although he noted that the classification and subgeneric concept (as proposed by Ohaus) were in need of study. Subsequent to Hardy’s revision (Hardy 1975), many new species of *Pelidnota* have been described. Keys to the Mexican species (Delgado et al. 1988) and Costa Rican species (Solís and Morón 1994) of *Pelidnota* are available. Soula (2006, 2008, 2009, 2010a, 2010b, 2011) described 104 species and subspecies and provided difficult-to-use keys to many species.

Research on *Pelidnota* and its allies was initiated by one of us in the 1990s (MLJ). This research, however, became intractable when Soula began describing many pelidnotine taxa and depositing type specimens in his private collection where they were not accessible to other scientists. Additionally, Soula created many new species for North American morphotypes of *P. punctata* (see “*Pelidnota punctata* (Linnaeus) species hypothesis and synonyms” below). A comprehensive revision of the genus and its allies is needed, including identification resources for all species.

Molecular and morphological phylogenetic analyses are necessary to unravel the evolutionary and ecological patterns within this interesting group. For over a century, taxonomy and nomenclature of the genus has been mired with several genus-level nomenclatural and classification conflicts (F. Bates 1904, Ohaus 1918, 1934b, Machatschke 1970, 1972, 1974, Krajcik 2008, Özdikmen 2009, Soula 2006, 2008, 2009, 2010a, 2011) (see Moore and Jameson 2013). Whereas the taxonomy and composition of Pelidnota (Pelidnota) is stable (ICZN 2003) and fairly homogeneous, other genus-level names are much less stable, the composition unknown, and identification is problematic (including *Chalcoplethis, Epichalcoplethis, Strigidia, Odontognathus, Ganonota* Ohaus). It is possible that *Pelidnota* *sensu lato* includes several natural groups (=genera), but to truly unravel the group, an unabridged systematic revision (taxonomy, phylogeny) must be undertaken and the group must be examined within a broad context of the Rutelini.

Due to possible paraphyly, diagnosis of the genus is difficult. For most species of *Pelidnota*, the pronotal basal bead is complete (obsolete in some); external margin of the mandible is bidentate; mesosternum with a transverse suture that separates the metasternum; prosternal projection more or less prominent and beaded; scutellum as wide as long; mesosternal projection not well-developed, not strongly produced anteriorly; elytral shoulder with a bead; metatrochanters sometimes protruding; claws simple in both sexes; male protarsal claw with or without inner tubercle; metatibia simple, gradually widening from base or corbeled.

Ohaus (1912) described the genus *Heteropelidnota* based on one, unusual male specimen (Ohaus 1934b; Plate 2, Fig. 11) (Fig. 72). The color and form of the specimen (the holotype of *P. kuhnti* [Ohaus] and the only known representative of the taxon) (Fig. 72) was compared with individuals of P. aeruginosa var. citripennis (valid name *P. semiaurata citripennis*) (Ohaus 1912). Examination of this specimen reveals that it is an aberrant, teratological specimen (see discussion of *P. kuhnti* in “Annotated Catalog”). In Ohaus’ (1934b) discussion of the genus *Heteropelidnota*, he compared the genus with *Hoplopelidnota* and *Xenopelidnota*, both of which possess a dense row of setae near the ventral apex of the elytra. Ohaus (1934b) stated that *Hoplopelidnota* and *Xenopelidnota* differ from *Heteropelidnota* based on the bidentate mandible and produced mesometasternal peg. It should be noted that the dense row of setae on the ventral side of the elytra is observed within many rutelines, but the position (subapically, anteapically, apically) and the density of setae varies widely. The function of this character is unknown (possibly functioning in flight or preventing water loss) and should be investigated. Ohaus included a new species in the genus, *P. cribrata* (Ohaus), and he transferred *Pelidnota rostrata* Burmeister (Ohaus 1918) to the genus. Martínez (1967) described *P. ustarani* (Martínez), also including it in the genus. After examination of the species included in the genus and based on lack of sufficient “collective” characters that support the genus, Soula (2008) transferred *P. cribrata*, *P. ustarani*, and *P. rostrata* to the genus *Pelidnota* (Soula 2008). However, he retained *H. kuhnti* in the genus based on its many “singularities.” Indeed, Soula (2008) also seemed to imply that *H. kuhnti* was a member of the genus *Pelidnota*. Herein, we consider *Heteropelidnota* a new **junior synonym** of *Pelidnota*. Lacking certainty of the species association due to the extreme deformities, we retain the species name and transfer the species to the genus *Pelidnota*.

### Peruquime

Taxon classificationAnimaliaColeopteraScarabaeidae

Mondaca & Valencia, 2016

Fig. 98

#### Type species.

*Peruquime arequipensis* Mondaca & Valencia, 2016.

#### Species.

1 species; length 8.3–10.5 mm.

*Peruquime arequipensis* is a small, setose scarab that inhabits high elevation (3,800–4,000 m), arid regions in southern Peru. The monotypic taxon possesses several unusual characters that are not typically observed in the Rutelinae: labrum projects anteriorly beyond the clypeal apex and fused to the clypeus (similar to some Melolonthinae: Tanyproctini or “pachydemine” scarabs), labrum horizontally produced with respect to the clypeus, antennal club is greatly enlarged (Mondaca and Valencia 2016). The taxon was classified in the tribe Rutelini based on the independently movable claws and laterally flattened unguitractor plate. The taxon was compared with *Eremophygus*, but it differs based on the pyriform mentum (form oval in *Eremophygus*) and antenna with 10 segments and enlarged club (antenna 9- or 10-segmented and lacking enlarged club in *Eremophygus*). It was postulated that *Peruquime*, together with *Neogutierrezia*, possess convergent characters that allow for adaptions to arid habitats (Mondaca and Valencia 2016).

*Peruquime arequipensis* is endemic to the Puna biogeographic region of the Andes, an area known for high endemism. Adult *Peruquime arequipensis* are diurnal and are active during the rainy season where they were collected in traps (flight intercept, pan, and pitfall). Larvae and sister-group relationships are not known.

### Pseudogeniates

Taxon classificationAnimaliaColeopteraScarabaeidae

Ohaus, 1910

Fig. 99

#### Type species.

*Pseudogeniates richterianus* Ohaus, 1910.

#### Species.

3 species; length 12–19 mm.

The genus *Pseudogeniates* is endemic to Argentina, and species are associated with arid habitats in the Chaco, Pampa, Espinal, and Monte ecoregions (Jameson and Ocampo 2012). The genus includes three species that are poorly represented in collections. Ohaus (1910a) puzzled over the first specimens that he studied in the genus, and originally thought that they represented teratological abnormalities due to the unusual form of the clypeus and mouthparts that resemble species in the and (both Rutelinae). As the genus name implies, members resemble species in the genus *Geniates* (), but they are easily diagnosed by the feathery fringe of setae on the ventral edge of the elytra, the mesosternal peg that is lacking, claws on all legs that are simple (lacking inner tubercle), the incomplete frontoclypeal suture, the maxillae that lack teeth, and the mandibular apex that has only one, recurved tooth (Jameson and Ocampo 2012).

Species in the genus are reviewed and an identification key is available (Jameson and Ocampo 2012). Natural history is poorly known, and the immature stages have not been described. Adults have been collected at lights from December to February at elevations ranging from 500–750 m.

### Sorocha

Taxon classificationAnimaliaColeopteraScarabaeidae

Soula, 2006

Figs 1E , 100 , 101 , 102 , 103 , 104 , 105

#### Type species.

*Pelidnota acutipennis* F. Bates, 1904.

#### Species.

16 species and subspecies; length 16–19 mm.

Soula (2006) described the genus *Sorocha* for a homogeneous group of species that Ohaus (1934b) had placed in the “*P. pulchella* group” based on the smooth, shiny elytra that lack markings, and distribution in the Andean highlands. Based on overall gestalt, species in the genus *Sorocha* are similar to species in the genus *Pseudochlorota* Ohaus (Lasiocalina), but they differ in the following respects: larger claw on all legs (simple in *Sorocha*; widely split in *Pseudochlorota*); unguitractor plate (flat and with two apical setae in *Sorocha*; subcylindrical and with two or more setae in *Pseudochlorota*); pronotal basolateral corner (quadrate in *Sorocha*; rounded in *Pseudochlorota*); and apex of the metatibia (lacking spinules in *Sorocha*; possessing spinules in *Pseudochlorota*).

This taxon requires phylogenetic analysis because we think some species are probably more appropriately placed in *Pelidnota*. *Sorocha* can be characterized, in part, by the following characters: disc of the frons with a V-shaped depressed region (shared with *Pseudochlorota*); all claws simple; male protarsal claw with inner tubercle; bidentate mandibles; pronotum with bead complete or incomplete apically (complete laterally and basally); elytral base with a median “dimple” lateral of scutellum; elytral epipleuron shelf-like (not rounded); protibia with basal external tooth slightly removed from apical teeth; clypeal length shorter than length of frons; eyes large; apex of the metatibia biemarginate and lacking apical spinules; meso- and metatarsomere 5 lacking internomedial tooth; mesometasternal keel not surpassing mesocoxae; metasternum with dense pilosity.

Species in the genus are superficially similar, and identification is hampered due to lack of a key. Females cannot currently be identified due to similarity among species. Species in the genus are distributed at high elevations from Colombia and Venezuela to Ecuador, Bolivia, and Peru. Larvae are not known. Soula (2006) stated that species in the genus are not readily attracted to lights at night.

### Xenopelidnota

Taxon classificationAnimaliaColeopteraScarabaeidae

F. Bates, 1904

Figs 106 , 107

#### Type species.

*Plusiotis anomala* Burmeister, 1844.

#### Species.

3 species and subspecies; length 19–27 mm.

Species in the genus *Xenopelidnota* resemble castaneous-colored *Pelidnota*, but the taxon is easily diagnosed by its dark-brown color and parabolic clypeus. The apices of the mandibles are quite variable (weakly bidentate, unidentate, rounded), perhaps due to wear and age. Additional characters that diagnose the genus are as follows: claws simple; male protarsal claw with inner tubercle; pronotum with bead complete apically, laterally and basally; elytral epipleuron shelf-like (not rounded); elytral apex with dense, short tawny setae; fifth meso- and metatarsomeres lacking internomedial tooth; apex of metatibia expanded, straight (lacking corbel or emarginations), and with many spinules; prosternal keel short (not produced to level of procoxae); and mesosternum not appreciably produced beyond mesometasternal suture.

Species in the genus are distributed in northern South America (Colombia, Venezuela, Trinidad, St. Vincent and the Grenadines). As typical of rutelines in this region, species are externally quite similar but male parameres possess a great deal of variability. Phylogenomic analyses of the *Xenopelidnota* lineage may reveal a greater understanding of the biogeography of the region. Larvae, natural history, and sister-group relationships of the group are not known.

### *Pelidnota punctata* (Linnaeus) species hypothesis and synonyms

*Pelidnota punctata* (Linnaeus) is a widespread species in North America occurring from Ontario and Quebec to Florida west to South Dakota and Texas. The host plant of this species is grape (*Vitis* Linnaeus; Vitaceae) foliage and fruit and the larvae develop in rotting stumps and logs of various deciduous trees.

The taxonomic history of this species dates back to the very beginning of zoological binomial nomenclature with a brief description by Linnaeus (1758) (as *Scarabaeus punctatus*), followed by more extensive (and quite sufficient) descriptions in later editions of *Systema Naturae* (Linnaeus 1764, 1767). The locality was erroneously given as “India” (Linnaeus 1758, 1764), but was later corrected to “Carolina” (Linnaeus 1767). The photograph of the Linnaeus lectotype specimen leaves no doubt about the identity of this species (Fig. 79). The lectotype is formerly of the Ludovicae Ulricae collection, which is now housed in the Zoological Institute in Uppsala University, Sweden (UUZM).

*Melolontha lutea* Olivier, 1789 was later described, but it has since been recognized that Olivier (1789) was describing the lighter colored and non-spotted southeastern United States version of this same species (see Hardy 1975 for a detailed discussion). Casey (1915) has the unfortunate notoriety of describing a further 10 synonyms of *P. punctata* based largely on intraspecific color variations in this species. Hardy (1974, 1975) synonymized all of these Casey names during the course of his taxonomic revision of the genus *Pelidnota*. Hardy (1975) gave a good account of the color variation of *P. punctata* and detailed a north-south cline of variation. We have observed that specimens from Canada and the northern United States always have dark legs and clearly defined spots (six on the elytra and two on the pronotum) while the legs and spots can be much lighter in specimens from the southern United States. We have observed that many specimens from Florida and Texas have light legs and little to no trace of spots on the pronotum or elytra. Considering the scope of the variation, even within smaller regions, we postulate that larval diet/nutrition, environmental conditions during development, and the length of time spent in the larval stage can have a significant impact on color patterns along with genetics for this particular species. Ritcher (1966) stated that in Lexington, Kentucky, *P. punctata* pass the winter in the larval stage and appear to have a two-year life cycle. It is possible that the life cycle of this species is accelerated in the southern part of the range. *Pelidnota punctata* may have an extended life cycle in the northern part of the range in response to decreased temperature and more extreme seasonal climate fluctuation.

*Pelidnota genieri* Soula was described as a purported species endemic to Ottawa, Ontario, Canada (Soula 2009). Soula’s (2009) description was based on color patterns and trivial structural characters without any detailed comparisons with *P. punctata* specimens from other parts of Canada and North America. We studied the holotype, allotype, and ten paratypes (five males, five females) from Soula’s type series and have concluded that the color patterns observed are well within the range of variation observed from specimens of *P. punctata* from other parts of Ontario, Quebec, and across the eastern half of the United States (over 500 specimens were examined). In fact, the holotype, allotype, and two paratypes were from a larger series of 51 specimens from the same collecting event (Ottawa, ONT. / 5. VIII.1971 / J.E.H. Martin) all in the Canadian Museum of Nature Collection. Having seen only four of the 51 specimens from this collecting event, Soula was unaware that the color variation observed in this series alone undermined many of the characters used to justify his new species (varying shades of dorsal coloration, different sizes of dark spots, different amounts of metallic green reflections around and between the eyes). Therefore, we are placing *Pelidnota genieri* in synonymy with *Pelidnota punctata* (**syn. n.**).

Soula’s (2009) motivation for describing such an obvious synonymy is unclear, but the quality of his work is highly suspect in our opinions after using his publications and examining material identified and described by him in the Canadian Museum of Nature collection and CCECL. Since Marc Soula’s death in 2012, his taxonomic work has come under increased scrutiny and criticism for poor quality (e.g., Moore and Jameson 2013, Garner and Audibert 2015). A case in point relevant to *Pelidnota genieri* was the fact that Soula (2006) had previously described *Strigidia genieri* Soula, 2006, and then he transferred this species to *Pelidnota* (in Soula 2009), creating a secondary homonym with the *Pelidnota genieri* in the very same paper where the former name was described! Moore and Jameson (2013) fixed this homonymy problem by erecting *Pelidnota francoisgenieri* Moore & Jameson, 2013 as a replacement name for *Pelidnota genieri* Soula, 2009 (not *Pelidnota genieri* [Soula, 2006]). As an objective synonym of *Pelidnota genieri*, *Pelidnota francoisgenieri* is also here placed in synonym with *Pelidnota punctata* (**syn. n.**).

**Figure 4.**
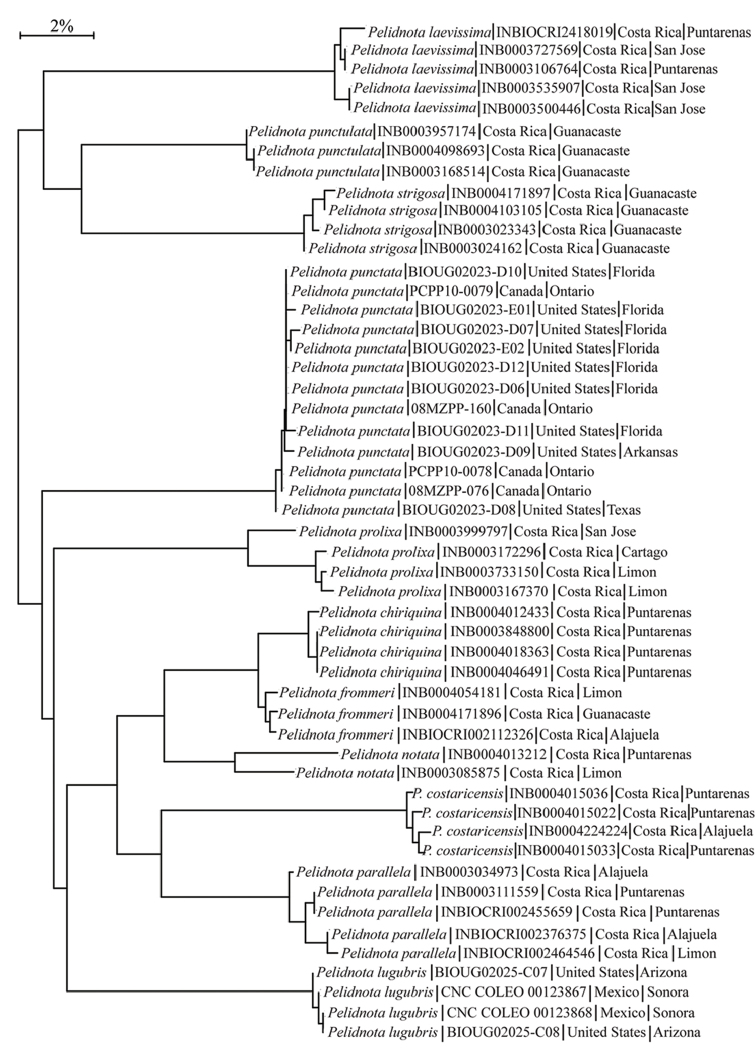
Neighbor-joining tree for individuals of *P. punctata* across the species’ distribution based on CO1 data. Between-species divergence is typically above 10% (e.g., *P. punctulata* and *P. strigosa*), whereas within species divergence is typically less than 1% (e.g., *P. punctata* and *P. lugubris*).

Soula (2009) also re-validated the names *Pelidnota lutea* (Olivier) and *Pelidnota texensis* Casey from synonymy with *Pelidnota punctata* for the Florida and Texas populations, respectively. He discussed some morphological differences between these populations and pointed out some variations in the male genitalia but also remarked that a DNA analysis would be necessary to determine the classification of this group of species. While we acknowledge that there are some morphological differences, we do not see stable and consistent differences between the populations or forms of *P. punctata* enough to warrant splitting this species at this time. DNA barcoding data is available for specimens from Ontario, Florida, Arkansas, and Texas (Fig. 4). Based on the lack of consistent morphological differences and the virtually identical CO1 barcoding data for specimens from the northern and southern extremes of the distribution, we hereby reinstate the junior synonymy of both *Pelidnota lutea* and *Pelidnota texensis* with *Pelidnota punctata*.

In the genus *Pelidnota*, analysis of the CO1 barcode data (Fig. 4) shows that between-species divergence is typically more than 10% (e.g., *P. punctulata* and *P. strigosa*). In contrast, the CO1 barcode region typically shows less than 1% divergence within species (e.g., *P. lugubris*), even when the individuals are separated by more than 200 miles (Sonora, Mexico to Arizona, USA). Similarly, individuals of *P. punctata* that were collected from across the species’s range (Florida, USA to Ontario, Canada) showed less than 1% CO1 divergence (mean: 0.24 % infraspecific divergence; max: 0.77 % infraspecific divergence). Individuals of *Pelidnota punctata* from Ontario, Canada (in the northern extent of the distribution) possess dark metallic green elytral spots, legs, and venter. Some individuals from Florida, USA (in the southern extent of the distribution) possess reduced elytral spots or no elytral spots and possess tan legs and venter. The CO1 data demonstrate that these phenotypic differences are not strong characters for species cohesion and provide support that all morphotypes of *P. punctata* are, in fact, conspecific (see “Annotated Catalog” for synthetic taxonomic information of *P. punctata* and synonyms).

## Annotated Catalog of the Pelidnotine Scarabs (Coleoptera: Scarabaeidae: Rutelinae: Rutelini)

### Tribe RUTELINI MacLeay, Subtribe RUTELINA MacLeay

#### Group Pelidnotine scarabs (paraphyletic)

27 genera (26 extant and 1 extinct) and 420 species and subspecies (419 extant and 1 extinct).

##### CATOCLASTUS

Taxon classificationAnimaliaColeopteraScarabaeidae

Solier, 1851

Catoclastus Solier, 1851: 95–96.

###### Type species.

*Catoclastus chevrolatii* Solier, 1851: 96-97, by monotypy.

###### Gender.

Masculine.

###### Species.

3 species.

##### Catoclastus chevrolatii

Taxon classificationAnimaliaColeopteraScarabaeidae

Solier, 1851

Catoclastus chevrolatii Solier, 1851: 96–97 [original combination].
Catoclastus chevrolati Solier [incorrect subsequent spelling by Harold 1869b: 1226].

###### Distribution.

PERU: Ayacucho (Ohaus 1918, 1934b, 1952, Blackwelder 1944, Machatschke 1972, Ratcliffe et al. 2015).

###### Types.

Soula (2010a: 4) designated the male neotype of *Catoclastus chevrolatii* at MNHN.

###### Remarks.

Solier (1851) described *C. chevrolatii* from “various parts of Chile” (translated from Spanish) and subsequent authors continued to cite these data (Harold 1869b, Reed 1876, Philippi 1887, Ohaus 1910c, 1918, 1934b, 1952, Blackwelder 1944, Machatschke 1972, Krajcik 2008, Soula 2010a). We have not examined any *Catoclastus* specimens from Chile and we consider these data erroneous. *Catoclastus chevrolatii* is currently known only from Peru.

##### Catoclastus jaumesi

Taxon classificationAnimaliaColeopteraScarabaeidae

Soula, 2010

Catoclastus jaumesi Soula, 2010a: 6 [original combination].

###### Distribution.

PERU (Soula 2010a, Ratcliffe et al. 2015).

###### Types.

**Figure 5.**
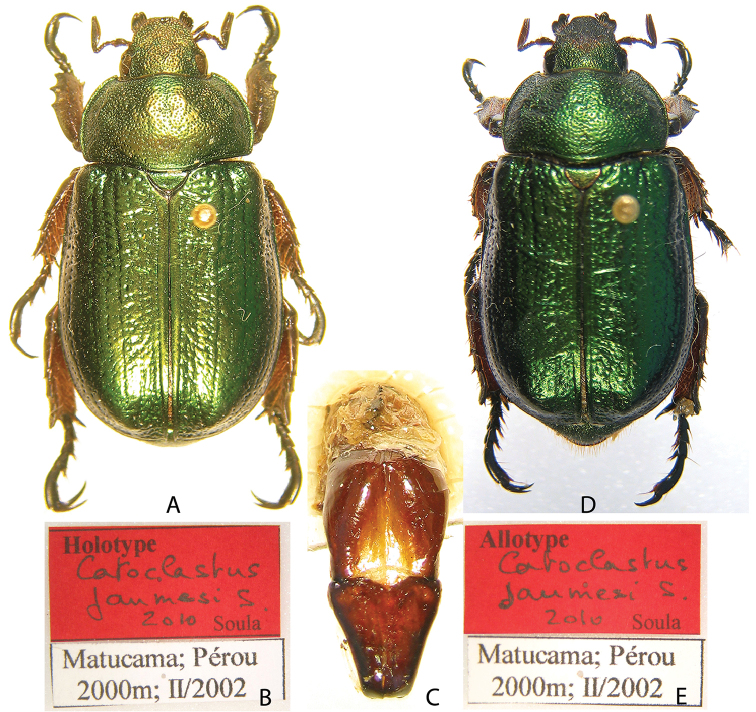
*Catoclastus jaumesi* Soula holotype male and allotype female from CCECL. **A** Dorsal habitus, holotype **B** Specimen labels, holotype **C** Male genitalia, dorsal view, holotype **D** Dorsal habitus, allotype **E** Specimen labels, allotype.

The following specimens are deposited at CCECL. 1 ♂ Holotype (Fig. 5A, B, C), 1 ♀ allotype (Fig. 5D, E), 8 ♂ paratypes, 4 ♀ paratypes: “Matucama; Pérou 2000m; II/2002//Holotype *Catoclastus jaumesi* S. 2010 Soula” (47031020); “Matucama; Pérou 2000m; II/2002//Allotype *Catoclastus jaumesi* S. 2010 Soula” (47031021); Eight paratypes with identical label data: “Matucama; Pérou 2000m; II/2002//Paratype *Catoclastus jaumesi* S. 2010 Soula” (47031022 to 47031028, exch58); “Chancho Moy Peru Kirsch//Paratype *Catoclastus jaumesi* S. 2010 Soula” (47031029); Three paratypes with identical label data: “Pérou coll. – SOULA//Paratype *Catoclastus jaumesi* S. 2010 Soula” (47031030 to 47031032). Genitalia card-mounted underneath the male holotype and five male paratypes. Box 4618690 SOULA.

##### Catoclastus rabinovichi

Taxon classificationAnimaliaColeopteraScarabaeidae

Martínez, 1971

Catoclastus rabinovichi Martínez, 1971: 79–81[original combination].

###### Distribution.

PERU: Cusco (Martínez 1971, Ratcliffe et al. 2015).

##### 

Taxon classificationAnimaliaColeopteraScarabaeidae

Burmeister, 1844

Chalcoplethis Burmeister, 1844: 410.
Pelidnota (Chalcoplethis) Burmeister [new subgenus status by Ohaus 1915b: 258–259].
Chalcoplethis Burmeister [revised genus status by Soula 2006: 98-99].

###### Type species.

*Chrysophora kirbii* Gray, 1832: 516, by monotypy.

###### Gender.

Feminine.

###### Species.

2 subspecies.

###### Remarks.

Krajcik (2012, 2013) considered *Chalcoplethis* to be a junior synonym of *Pelidnota*.

##### Chalcoplethis kirbii kirbii

Taxon classificationAnimaliaColeopteraScarabaeidae

(Gray, 1832)

Chrysophora kirbii Gray, 1832: 516 [original combination].
Chalcoplethis kirbii (Gray) [new combination by Burmeister 1844: 410–411].
Chalcoplethis kirbyi (Gray) [incorrect subsequent spelling by Harold 1869b: 1224].
Pelidnota (Chalcoplethis) kirbyi (Gray) [new subgeneric combination by Ohaus 1918: 29].
Chalcoplethis kirbyi (Gray) [revised combination by Soula 2006: 99–100].

###### Distribution.

BRAZIL: Bahia, Paraná, Espírito Santo, Rio Grande do Sul (Gray 1832, Burmeister 1844, Blanchard 1851, Harold 1869b, Ohaus 1918, 1934b, Blackwelder 1944, Machatschke 1972, Hardy 1975, Soula 2006, Krajcik 2008). COSTA RICA (Hardy 1975). PARAGUAY: Cororó (HNMB).

###### Types.

1 ♂ holotype of *Chrysophora kirbii* at BMNH (Soula 2006).

**Figure 6.**
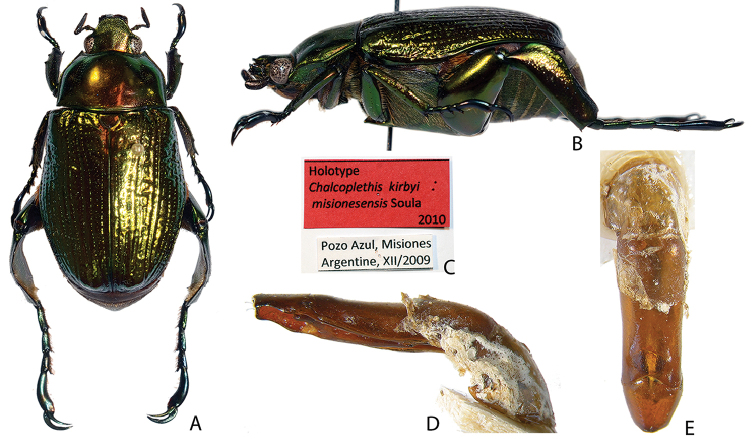
*Chalcoplethis kirbii misionesensis* Soula holotype male from CCECL. **A** Dorsal habitus **B** Lateral habitus **C** Specimen labels **D** Male genitalia, lateral view **E** Male genitalia, dorsal view.

##### Chalcoplethis kirbii misionesensis

Taxon classificationAnimaliaColeopteraScarabaeidae

Soula, 2010

Chalcoplethis kirbyi misionesensis Soula, 2010a: 46–47 [original combination].
Chalcoplethis kirbii misionesensis Soula [see suggested correct spelling by Moore and Jameson 2013: 383].

###### Distribution.

ARGENTINA: Misiones (Soula 2010a).

###### Types.

The following specimens are deposited at CCECL. 1 ♂ holotype (Fig. 6), 1 ♀ allotype, 2 ♂ paratypes, 2 ♀ paratypes: “Pozo Azul, Misiones Argentine, XII/2009//Holotype *Chalcoplethis kirbyi misionesensis* Soula 2010” (47030871); “Pozo Azul, Misiones Argentine, XII/2009//Allotype *Chalcoplethis kirbyi misionesensis* Soula 2010” (47030872); Three paratypes with identical label data: “Pozo Azul, Misiones Argentine, XII/2009//Paratype *Chalcoplethis kirbyi misionesensis* Soula 2010” (47030873, 47030956 and 47030957); “Misiones Arg. M. SOULA det. 19//Paratype *Chalcoplethis kirbyi misionesensis* Soula 2010” (47030874). Genitalia are card-mounted underneath the male holotype and the male paratype specimens. Box 4618649 SOULA and 4616345 PORION.

##### Chipita

Taxon classificationAnimaliaColeopteraScarabaeidae

Soula, 2008

Chipita Soula, 2008: 10.

###### Type species.

*Byrsopolis mexicana* Ohaus 1905: 324, by monotypy.

###### Gender.

Feminine.

###### Species.

1 species.

##### Chipita mexicana

Taxon classificationAnimaliaColeopteraScarabaeidae

(Ohaus, 1905)

Byrsopolis mexicana Ohaus, 1905: 324–325 [original combination].
Parhoplognathus mexicanus (Ohaus) [new combination by Ohaus 1915b: 257].
Chipita mexicana (Ohaus) [new combination by Soula 2008: 10].

###### Distribution.

MEXICO: Sinaloa (FSCA), Guerrero, Jalisco, Nayarit, Oaxaca (Ohaus 1905, 1918, 1934b, Blackwelder 1944, Machatschke 1972, Morón et al. 1988, Rodríguez-Palafox and Corona 2002, Soula 2008, Deloya et al. 2014).

###### Types.

**Figure 7.**
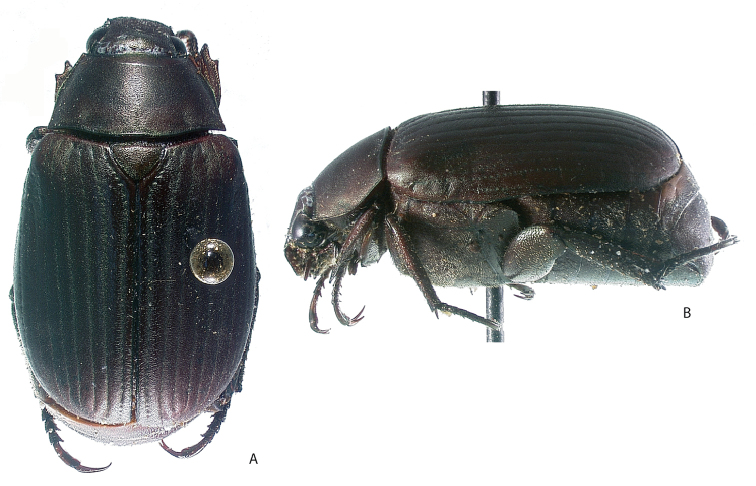
*Chipita mexicana* (Ohaus) female specimen from FSCA. **A** Dorsal habitus **B** Lateral habitus.

1 ♀ lectotype and 1 paralectotype of *Byrsopolis mexicana* at ZMHB (Soula 2006). An exemplar specimen is figured (Fig. 7).

##### CHRYSINA

Taxon classificationAnimaliaColeopteraScarabaeidae

Kirby, 1828

Chrysina Kirby, 1828: 522.
Plusiotis Burmeister, 1844 **synonym.**
Plusiotis Burmeister, 1844: 417. [Type species. *Pelidnota victorina* Hope, 1841, by original designation].
Chrysina Kirby [syn. by Hawks 2001: 2].
Plusiotina Casey, 1915 **synonym.**
Plusiotina Casey, 1915: 84. [Type species. *Plusiotina aeruginis* Casey, 1915 by subsequent designation (Hawks 2001: 2) (= *Chrysina lecontei* [Horn, 1882])].
Plusiotis Burmeister [syn. by Ohaus 1934b: 59].
Pelidnotopsis Ohaus, 1915b **synonym.**
Pelidnotopsis Ohaus, 1915b: 257. [Type species. *Pelidnota plusiotina* Ohaus, 1912, by monotypy].
Chrysina Kirby [syn. by Hawks 2001: 2].
Pelidnotopsis Ohaus [revised genus status by Soula 2010b: 11].
Chrysina Kirby [syn. by Moore and Jameson 2013: 381].

###### Type species.

*Chrysina peruviana* Kirby 1828: 523, by monotypy.

###### Gender.

Feminine.

###### Species.

113 species.

###### Remarks.

Casey (1915) did not designate a type species for the genus *Plusiotina*. No authors addressed this until Machatschke (1972) subsequently designated *Plusiotis woodi* Horn as the type of *Plusiotina*. This type designation was invalid based on ICZN Article 67.2 that states “A nominal species is only eligible to be fixed as the type species of a nominal genus or subgenus if it is an originally included nominal species”. *Plusiotis woodi* Horn was not an originally included nominal species of *Plusiotina* and was thus invalidly designated as the type species of *Plusiotina*. Hawks (2001) corrected this by subsequently designating *Plusiotina aeruginis* Casey (= *Chrysina lecontei* [Horn, 1882]), an originally included nominal species of *Plusiotina*, as the type species of *Plusiotina*.

##### Chrysina adelaida

Taxon classificationAnimaliaColeopteraScarabaeidae

(Hope, 1841)

Pelidnota adelaida Hope, 1841: 147 [original combination].
Plusiotis adelaida (Hope) [new combination by Burmeister 1844: 421].
Plusiotis adelaidae (Hope) [incorrect subsequent spelling by Nonfried 1891: 302].
Chrysina adelaida (Hope) [new combination by Hawks 2001: 7].
Pelidnota ornatissima Sturm, 1843 **synonym.**
Pelidnota ornatissima Sturm, 1843: 341-342 [original combination].
Plusiotis adelaida (Hope) [syn. by Burmeister 1844: 421].
Plusiotis adelaida pavonacea Casey, 1915 **synonym.**
Plusiotis adelaida pavonacea Casey, 1915: 84.
Chrysina adelaida (Hope) [**syn. n.**].

###### Distribution.

MEXICO: Chiapas, Chihuahua, Coahuila, Colima, Durango, Guerrero, Hidalgo, Jalisco, México, Michoacán, Morelos, Oaxaca, Puebla, Queretaro, San Luis Potosí, Tamaulipas, Tlaxcala, Veracruz (Hope 1841, Sturm 1843, Burmeister 1844, Lucas 1865, Harold 1869b, Boucard 1875, H. W. Bates 1888, Nonfried 1891, Casey 1915, Ohaus 1918, 1934b, Blackwelder 1944, Cazier 1951, Carrillo et al. 1966, Machatschke 1972, Morón 1985, 1990, 1991, 1994, Morón and Zaragoza 1976, Morón and Deloya 1991, Ratcliffe et al. 1992, Deloya et al. 1993, Lobo and Morón 1993, García-Montiel et al. 2003, Thomas et al. 2006, Delgado-Castillo and Márquez 2006, Krajcik 2008, Márquez 2008, Márquez and Sierra-Martínez 2008, Muñoz-Hernández et al. 2008, Morón and Márquez 2012, Márquez et al. 2013, Deloya et al. 2014).

###### Remarks.

Hawks (2006) provided an online “Checklist of *Chrysina* species” wherein he considered *C. adelaidapavonacea* Casey to be a synonym of *C. adelaida*. Casey (1915) proposed this subspecies for individuals from Guerrero, Mexico, which differed from the nominative form based on “feebly convex intervals”, “very shallow clypeal sinuation”, and slight differences in color. We agree that this subspecies is conspecific with the nominative form. Because the on-line checklist is not considered to be formally published for nomenclatural purposes, we synonymize this subspecies herein. *Chrysina adelaida* (=*C. ornatissima*) was reported from “San Gerónimo”, Guatemala (possibly San Jerónimo, Baja Verapaz) (H. W. Bates 1888, Ohaus 1918, 1934b, Blackwelder 1944). There have been no further published collection records for *C. adelaida* in Guatemala and these data need to be re-evaluated.

##### Chrysina adolphi

Taxon classificationAnimaliaColeopteraScarabaeidae

Chevrolat, 1859

Chrysina adolphi Chevrolat, 1859: 481 [original combination].
Chrysina macropus var. adolphi Chevrolat [new infrasubspecific status by H. W. Bates 1888: 285].
Chrysina macropus adolphi Chevrolat [new subspecific status by Machatschke 1972: 17].
Chrysina macropus (Francillon) [syn. by Morón 1990: 54].
Chrysina adolphi Chevrolat [revised species status by Hawks 2001: 2].

###### Distribution.

MEXICO: Guerrero, Oaxaca (Chevrolat 1859, Harold 1869b, Boucard 1875, H. W. Bates 1888, Ohaus 1918, Blackwelder 1944, Machatschke 1972, Hawks 2001, Thomas et al. 2006, Krajcik 2008).

###### Types.

1 ♀ lectotype of *Chrysina adolphi* at BMNH (Hawks 2001).

##### Chrysina aenigmatica

Taxon classificationAnimaliaColeopteraScarabaeidae

(Morón, 1990)

Plusiotis aenigmatica Morón 1990: 29 [original combination].
Chrysina aenigmatica (Morón) [new combination by Hawks 2001: 7].

###### Distribution.

MEXICO: México, Morelos (Morón 1990, Deloya et al. 1993, Krajcik 2008).

###### Types.

1 ♂ holotype, 1 ♀ allotype and 3 paratypes of *Plusiotis aenigmatica* at MXAL (Morón 1990); 1 paratype at MNHN (Morón 1990); 1 paratype at BMNH (Morón 1990); 2 paratypes at ZMHB (Morón 1990); 2 paratypes at CNC (Morón 1990); 2 paratypes at IEXA (Morón 1990).

##### Chrysina alfredolaui

Taxon classificationAnimaliaColeopteraScarabaeidae

(Hawks, 1995)

Plusiotis alfredolaui Hawks, 1995: 273–275 [original combination].
Chrysina alfredolaui (Hawks) [new combination by Hawks 2001: 8].

###### Distribution.

GUATEMALA (Thomas et al. 2006, Monzón 2010). MEXICO: Veracruz (Hawks 1995, Krajcik 2008, Thomas et al. 2006).

###### Types.

1 ♂ holotype of *Plusiotis alfredolaui* at CAS (Hawks 1995); 1 ♀ allotype at EMEC (Hawks 1995).

##### Chrysina alphabarrerai

Taxon classificationAnimaliaColeopteraScarabaeidae

(Morón, 1981)

Plusiotis alphabarrerai Morón, 1981: 57-63 [original combination].
Chrysina alphabarrerai (Morón) [new combination by Hawks 2001: 8].

###### Distribution.

MEXICO: Veracruz (Morón 1981, 1990, Lobo and Morón 1993, Krajcik 2008, Thomas et al. 2006).

###### Types.

1 ♂ holotype and 1 ♀ allotype at MXAL (Morón 1981).

##### Chrysina arellanoi

Taxon classificationAnimaliaColeopteraScarabaeidae

Monzón, 2012

Chrysina arellanoi Monzón, 2012: 1–4 [original combination].

###### Distribution.

MEXICO: Oaxaca (Monzón 2012, Thomas et al. 2013, Morón and Nogueira 2016).

###### Types.

1 ♂ holotype and 1 ♀ allotype at CNIN (UNAM) (Monzón 2012).

###### Remarks.

Morón and Nogueira (2016) considered the valid name for this species to be *Plusiotis arellanoi*. Lacking a clearly articulated and evidence-based rationale for this nomenclatural change, we use the name *Chrysina arellanoi*.

##### Chrysina argenteola

Taxon classificationAnimaliaColeopteraScarabaeidae

(H. W. Bates, 1888)

Plusiotis argenteola H. W. Bates, 1888: 277 [original combination].
Chrysina argenteola (H. W. Bates) [new combination by Hawks 2001: 8].

###### Distribution.

COLOMBIA: Antioquia, Cauca, Chocó, Nariño, Putumayo, Valle del Cauca (H. W. Bates 1888, Nonfried 1892, Ohaus 1903, 1918, 1934b, Blackwelder 1944, Machatschke 1972, Morón 1990, 1991, Arnaud 1994, Restrepo et al. 2003, Neita-Moreno et al. 2006, Neita-Moreno 2011, Thomas et al. 2006). ECUADOR: Bolívar, Cotopaxi, Esmeraldas, Pichincha (Ohaus 1903, 1908b; 1918, 1934b, Blackwelder 1944, Machatschke 1972, Morón 1990, 1991, Arnaud 1994, Paucar-Cabrera 2005, Thomas et al. 2006, Krajcik 2008, Camacho Cárdenas 2015). PERU: Junín, Lima (Ohaus 1903, 1918, 1934b, Blackwelder 1944, Machatschke 1972, Ratcliffe et al. 2015).

###### Types.

1 ♂ neotype at MNHN (Arnaud 1994).

##### Chrysina aurigans

Taxon classificationAnimaliaColeopteraScarabaeidae

(Rothschild & Jordan, 1894)

Plusiotis aurigans Rothschild & Jordan, 1894: 504–505 [original combination].
Chrysina aurigans (Rothschild and Jordan) [new combination by Hawks 2001: 8].
Plusiotis keithi Linell, 1895 **synonym.**
Plusiotis keithi Linell, 1895: 1–2 [original combination].
Plusiotis aurigans Rothschild and Jordan [syn. by Ohaus 1918: 18].

###### Distribution.

COSTA RICA: Alajuela, Cartago, San José (Rothschild and Jordan 1894, Linell 1895, Ohaus 1918, 1934b, Blackwelder 1944, 1952, Machatschke 1972, Morón 1990, Thomas et al. 2006, Krajcik 2008, García-López et al. 2013). PANAMA (Thomas et al. 2006).

##### Chrysina aurilisternum

Taxon classificationAnimaliaColeopteraScarabaeidae

Pérez-Flores, Villagomez, & Galindo, 2016

Chrysina aurilisternum Pérez-Flores, Villagomez, & Galindo, 2016: 607–610 [original combination].

###### Distribution.

MEXICO: Guanajuato (Pérez-Flores et al. 2016).

###### Types.

1 ♂ holotype, 1 ♀ allotype and 17 paratypes at CNIN (UNAM) (Pérez-Flores et al. 2016).

##### Chrysina auripes

Taxon classificationAnimaliaColeopteraScarabaeidae

Gray, 1832

Chrysina auripes Gray, 1832: 517 [original combination].
Plusiotis auripes (Gray) [new combination by Burmeister 1844: 419].
Chrysina auripes Gray [revised combination and revised application by Hawks 2001: 3].
Pelidnota auripes Hope, 1841 **synonym.**
Pelidnota auripes Hope 1841: 147 [original combination].
Plusiotis auripes (Gray) [syn. by Burmeister 1844: 419].
Chrysina auripes Gray [syn. by Hawks 2001: 3].
Plusiotis chalchihuitli Morón, 1990 **synonym.**
Plusiotis chalchihuitli Morón, 1990: 16, 36-37 [original combination].
Chrysina auripes Gray [syn. by Hawks 2001].

###### Distribution.

MEXICO: Nuevo León, Oaxaca, San Luis Potosi, Tamaulipas (Gray 1832, Laporte 1840, Burmeister 1844, Blanchard 1851, Boucard 1875, H. W. Bates 1888, Nonfried 1891, Ohaus 1918, 1934b, Blackwelder 1944, Machatschke 1972, Morón 1990, Hawks 2001, Thomas et al. 2006, Krajcik 2008).

###### Types.

1 ♂ lectotype at OUMNH (Hawks 2001).

###### Remarks.

Krajcik (2012, 2013) erroneously listed *C. chalchihuitli* as a valid name.

##### Chrysina aurofoveata

Taxon classificationAnimaliaColeopteraScarabaeidae

(Morón, 1981)

Plusiotis aurofoveata Morón, 1981: 50–57 [original combination].
Chrysina aurofoveata (Morón) [new combination by Hawks 2001: 7].

###### Distribution.

MEXICO: Hidalgo, Puebla (Morón 1981, 1990, 1993, 1994, Thomas et al. 2006, Delgado-Castillo and Márquez 2006, Krajcik 2008, Márquez 2008, Muñoz-Hernández et al. 2008, Márquez et al. 2013).

###### Types.

1 ♂ holotype, 1 ♀ allotype and paratypes at MXAL (Morón 1981).

##### Chrysina auropunctata

Taxon classificationAnimaliaColeopteraScarabaeidae

Ohaus, 1913

Plusiotis auropunctata Ohaus, 1913: 491 [original combination].
Chrysina auropunctata (Ohaus) [new combination by Hawks 2001: 7].

###### Distribution.

GUATEMALA: San Marcos (Morón 1990, Monzón 1995, Monzón et al. 1999, Thomas et al. 2006). MEXICO: Chiapas (Ohaus 1913, 1918, 1934b, Machatschke 1972, Morón 1990, 1991, Thomas 1993, Monzón 1995, Thomas et al. 2006, Krajcik 2008).

##### Chrysina aurora

Taxon classificationAnimaliaColeopteraScarabaeidae

(Boucard, 1875)

Plusiotis aurora Boucard, 1875: 119 [original combination].
Chrysina aurora (Boucard) [new combination by Hawks 2001: 8].

###### Distribution.

COSTA RICA: Alajuela, San José (Morón 1990, Thomas et al. 2007, García-López et al. 2013). NICARAGUA: Chontales (Maes 1987). PANAMA: Chiriquí (H. W. Bates 1888, Ohaus 1918, 1934b, Blackwelder 1944, Machatschke 1972, Maes 1987, Morón 1990, Ratcliffe 2002, Krajcik 2008, Thomas et al. 2007).

##### Chrysina badeni

Taxon classificationAnimaliaColeopteraScarabaeidae

(Boucard, 1878)

Plusiotis badeni Boucard, 1878: 298–295 [original combination].
Chrysina badeni (Boucard) [new combination by Hawks 2001: 4].

###### Distribution.

MEXICO: Hidalgo, Puebla, San Luis Potosí, Veracruz (Boucard 1878, H. W. Bates 1888, Nonfried 1892, Ohaus 1918, 1934b, Blackwelder 1944, Machatschke 1972, Morón 1990, 1991, 1993, 1994, Ratcliffe et al. 1992, Cano and Morón 1994, Morón et al. 1997, Hawks 2001, Thomas et al. 2006, Delgado-Castillo and Márquez 2006, Krajcik 2008, Márquez 2008, Morón and Márquez 2012, Márquez et al. 2013).

###### Types.

1 ♀ lectotype at ZMHB (Hawks 2001).

##### Chrysina baileyana

Taxon classificationAnimaliaColeopteraScarabaeidae

Monzón, 2010

Chrysina baileyana Monzón, 2010: 7–10 [original combination].

###### Distribution.

GUATEMALA: Huehuetenango (Monzón 2010).

###### Types.

**Figure 8.**
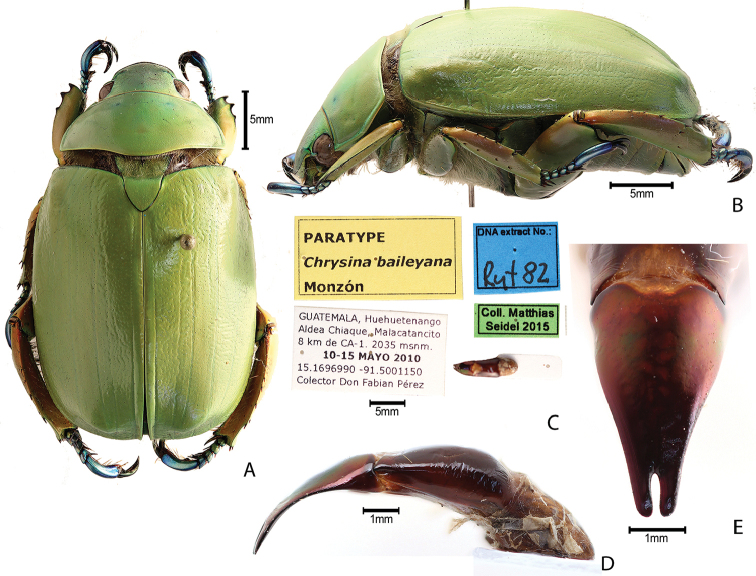
*Chrysina baileyana* Monzón paratype male from MSPC. **A** Dorsal habitus **B** Lateral habitus **C** Specimen labels and male genitalia **D** Male genitalia, lateral view **E** Male parameres, dorsal view.

1 ♂ holotype and 1 ♀ allotype at UVGC (Monzón 2010); paratypes at UVGC, FSCA and WSU (Monzón 2010). 2 paratypes at MSPC (Fig. 8).

##### Chrysina batesi

Taxon classificationAnimaliaColeopteraScarabaeidae

(Boucard, 1875)

Plusiotis batesi Boucard, 1875: 119–120 [original combination].
Chrysina batesi (Boucard) [new combination by Hawks 2001: 8].

###### Distribution.

COSTA RICA: Cartago, San José (Boucard 1875, 1878, H. W. Bates 1888, Nonfried 1891, Ohaus 1918, 1934b, Blackwelder 1944, Machatschke 1972, Morón 1990, Curoe 2001, Thomas et al. 2006, Krajcik 2008). PANAMA: Chiriquí (Morón 1990, Curoe 2001, Ratcliffe 2002, Thomas et al. 2006).

##### Chrysina beckeri

Taxon classificationAnimaliaColeopteraScarabaeidae

H. W. Bates, 1889

Chrysina beckeri H. W. Bates, 1889: 411 [original combination].

###### Distribution.

MEXICO: Durango (H. W. Bates 1889, Ohaus 1918, 1934b, Blackwelder 1944, Cazier 1951, Machatschke 1972, Morón 1990, Krajcik 2008, Thomas et al. 2007).

###### Types.

Holotype of *Chrysina beckeri* at MNHN.

##### Chrysina benesi

Taxon classificationAnimaliaColeopteraScarabaeidae

Pokorný & Curoe, 2012

Chrysina benesi Pokorný & Curoe, 2012: 111–116 [original combination].

###### Distribution.

MEXICO: Chiapas (Pokorný and Curoe 2012).

###### Types.

**Figure 9.**
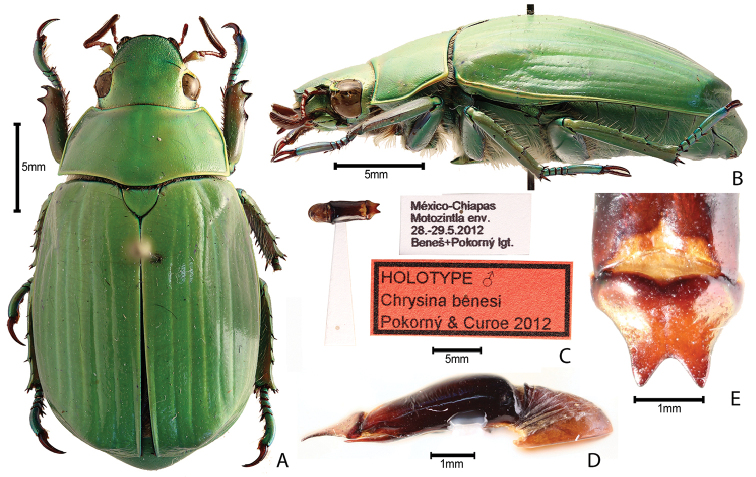
*Chrysina benesi* Pokorný and Curoe holotype male from NMPC. **A** Dorsal habitus **B** Lateral habitus **C** Specimen labels and male genitalia **D** Male genitalia, lateral view **E** Male parameres, caudal view.

1 ♂ holotype (Fig. 9) and 1 ♀ allotype at NMPC (Pokorný and Curoe 2012); 1 paratype at BMNH; additional paratypes at DJCC, MXAL, and other private collections (Pokorný and Curoe 2012).

##### Chrysina beraudi

Taxon classificationAnimaliaColeopteraScarabaeidae

(Warner, Hawks, & Bruyea, 1992)

Plusiotis beraudi Warner, Hawks, & Bruyea, 1992: 99–100 [original combination].
Chrysina beraudi (Warner, Hawks, and Bruyea) [new combination by Hawks 2001: 7].

###### Distribution.

COSTA RICA: San José (Warner et al. 1992, Thomas et al. 2006, Krajcik 2008).

###### Types.

1 ♂ holotype at CAS (Warner et al. 1992).

##### Chrysina beyeri

Taxon classificationAnimaliaColeopteraScarabaeidae

(Skinner, 1905)

Plusiotis beyeri Skinner, 1905: 289-290 [original combination].
Chrysina beyeri (Skinner) [new combination by Hawks 2001: 7].
Plusiotis ampliata Casey, 1915 **synonym.**
Plusiotis ampliata Casey, 1915: 82 [original combination].
Plusiotis beyeri Skinner [syn. by Cazier 1951: 5].
Plusiotis beyeri ocularis Casey, 1915 **synonym.**
Plusiotis beyeri ocularis Casey, 1915: 83 [original combination].
Plusiotis beyeri Skinner [syn. by Cazier 1951: 4].

###### Distribution.

MEXICO: Chihuahua, Sinaloa, Sonora (Coolidge 1911, Ohaus 1918, 1934b, Blackwelder 1944, Machatschke 1972, Thomas et al. 2006, Lugo et al. 2011). USA: Arizona (Skinner 1905, Biederman 1907, Coolidge 1911, Casey 1915, Ohaus 1918, 1934b, Leng 1920, Blackwelder 1939, 1944, Cazier 1951, Machatschke 1972, Gibson and Carrillo 1959, Morón 1990, 1991, Hardy 1991, Thomas et al. 2006, Krajcik 2008).

##### Chrysina blackalleri

Taxon classificationAnimaliaColeopteraScarabaeidae

Monzón & García, 2011

Chrysina blackalleri Monzón & García, 2011: 1–4 [original combination]

###### Distribution.

MEXICO: Oaxaca (Monzón and García 2011, Thomas et al. 2012).

###### Types.

1 ♂ holotype and 1 ♀ allotype at CNIN (UNAM) (Monzón and García 2011).

##### Chrysina boucardi

Taxon classificationAnimaliaColeopteraScarabaeidae

(Sallé, 1878)

Plusiotis boucardi Sallé, 1878: 21 [original combination].
Chrysina boucardi (Sallé) [new combination by Hawks 2001: 8].
Plusiotis magnificus Arrow, 1919 **synonym.**
Plusiotis magnificus Arrow, 1919: 380 [original combination].
Plusiotis boucardi Arrow [syn. by Morón 1990: 32].

###### Distribution.

COSTA RICA: Puntarenas, San José (Boucard 1878, H. W. Bates 1888, Ohaus 1913, 1918, 1934b, Blackwelder 1944, Machatschke 1972, Morón 1990, 1991, Curoe 1999, Thomas et al. 2006, Krajcik 2008). PANAMA: Chiriquí (Arrow 1919, Ohaus 1934b, Morón 1990, 1991, Curoe 1999, Ratcliffe 2002, Krajcik 2008).

##### Chrysina brevis

Taxon classificationAnimaliaColeopteraScarabaeidae

(Rothschild & Jordan, 1894)

Plusiotis brevis Rothschild & Jordan, 1894: 507 [original combination].
Chrysina brevis (Rothschild and Jordan) [new combination by Hawks 2001: 7].

###### Distribution.

MEXICO: Durango, Sinaloa (Rothschild and Jordan 1894, Ohaus 1918, 1934b, Blackwelder 1944, Cazier 1951, Machatschke 1972, Morón 1990, Morón et al. 1997, Krajcik 2008).

##### Chrysina bruyeai

Taxon classificationAnimaliaColeopteraScarabaeidae

(Hawks, 1999)

Plusiotis bruyeai Hawks, 1999: 22–24 [original combination].
Chrysina bruyeai (Hawks) [new combination by Hawks 2001: 7].

###### Distribution.

COSTA RICA: Alajuela, Cartago, Guanacaste, Heredia (Hawks 1999, Thomas et al. 2006, Krajcik 2008, García-López et al. 2013). HONDURAS: El Paraiso, Olancho (Hawks 1999, Thomas et al. 2006). NICARAGUA: Zelaya (Hawks 1999, Thomas et al. 2006).

###### Types.

1 ♂ holotype, 1 ♀ allotype and 31 paratypes at MNCR (Hawks 1999); 5 paratypes at MXAL (Hawks 1999); 1 paratype at BMNH (Natural History Museum 2014). 1 ♂ paratype at CMNC.

##### Chrysina cavei

Taxon classificationAnimaliaColeopteraScarabaeidae

Hawks & Bruyea, 1999

Chrysina cavei Hawks & Bruyea, 1999: 16–18 [original combination].

###### Distribution.

HONDURAS: Olancho, Yoro (Hawks and Bruyea 1999, Thomas et al. 2006, Krajcik 2008).

###### Types.

4 paratypes at BMNH (Natural History Museum 2014, BHG pers. obs. Aug. 2016); 3 ♂ paratypes at CMNC.

##### Chrysina centralis

Taxon classificationAnimaliaColeopteraScarabaeidae

(Morón, 1990)

Plusiotis centralis Morón 1990: 20 [original combination].
Chrysina centralis (Morón 1990) [new combination by Hawks 2001: 7].

###### Distribution.

GUATEMALA: Quetzaltenango, San Marcos (Morón 1990, 1991; Blackaller-Bages and Delgado 1994, Krajcik 2008, Thomas et al. 2010, Monzón 2010, Morón and Nogueira 2016).

###### Types.

1 ♂ holotype at MXAL (Morón 1990).

###### Remarks.

Morón and Nogueira (2016) considered the valid name for this species to be *Plusiotis centralis*. Lacking a clearly articulated and evidence-based rationale for this nomenclatural change, we use the name *Chrysina centralis*.

##### Chrysina chalcothea

Taxon classificationAnimaliaColeopteraScarabaeidae

(H. W. Bates, 1888)

Plusiotis chalcothea H. W. Bates, 1888: 284 [original combination].
Chrysina chalcothea (H. W. Bates) [new combination by Hawks 2001: 7].

###### Distribution.

COSTA RICA: Cartago, San José (H. W. Bates 1888, Nonfried 1892, Ohaus 1918, 1934b, Blackwelder 1944, Machatschke 1972, Morón 1990, 1991, Thomas et al. 2006, Krajcik 2008).

###### Types.

1 ♂ lectotype at BMNH (Natural History Museum 2014).

##### Chrysina chloreis

Taxon classificationAnimaliaColeopteraScarabaeidae

(H. W. Bates, 1888)

Plusiotis chloreis H. W. Bates, 1888: 282 [original combination].
Chrysina chloreis (H. W. Bates) [new combination by Hawks 2001: 8].

###### Distribution.

MEXICO: Chiapas, Oaxaca, Veracruz (H. W. Bates 1888, Nonfried 1892, Ohaus 1918, 1934b, Blackwelder 1944, Machatschke 1972, Morón 1981, 1990, Lobo and Morón 1993, Thomas 1993, Krajcik 2008).

##### Chrysina chrysargyrea

Taxon classificationAnimaliaColeopteraScarabaeidae

(Sallé, 1874)

Pelidnota chrysargyrea Sallé, 1874: 362 [original combination].
Plusiotis chrysargyrea (Sallé) [new combination by Boucard 1875: 120].
Chrysina chrysargyrea (Sallé) [new combination by Hawks 2001: 8].

###### Distribution.

COSTA RICA: Puntarenas, San José (Sallé 1874, Boucard 1875, 1878, H. W. Bates 1888, Nonfried 1891, Ohaus 1918, 1934b, Blackwelder 1944, Machatschke 1972, Morón 1990, Thomas et al. 2006, Krajcik 2008). PANAMA: Chiriquí (H. W. Bates 1888, Ohaus 1918, 1934b, Blackwelder 1944, Machatschke 1972, Morón 1990, Ratcliffe 2002, Thomas et al. 2006).

##### Chrysina chrysopedila

Taxon classificationAnimaliaColeopteraScarabaeidae

(H. W. Bates, 1888)

Plusiotis aurora var. chrysopedila H. W. Bates, 1888: 277 [original combination].
Plusiotis chrysopedila H.W. Bates [new species status by Ohaus 1912: 307].
Chrysina chrysopedila (H. W. Bates) [new combination by Hawks 2001: 4].

###### Distribution.

COSTA RICA (Morón 1990, Thomas et al. 2006). NICARAGUA: Chontales (H. W. Bates 1888, Nonfried 1892, Ohaus 1918, 1934b, Blackwelder 1944, Machatschke 1972, Maes 1987, Morón 1990, Thomas et al. 2006). PANAMA: Chiriquí (H. W. Bates 1888, Nonfried 1892, Ohaus 1918, 1934b, Blackwelder 1944, Machatschke 1972, Maes 1987, Morón 1990, Hawks 2001, Ratcliffe 2002, Thomas et al. 2006, Krajcik 2008).

###### Types.

1 ♀ lectotype and 8 paralectotypes of *Plusiotis aurora chrysopedila* at BMNH (Hawks 2001); 1 paralectotype at CNC (Hawks 2001).

##### Chrysina citlaltepetlamayatli

Taxon classificationAnimaliaColeopteraScarabaeidae

(Blackaller-Bages & Delgado, 1994)

Plusiotis citlaltepetlamayatli Blackaller-Bages & Delgado, 1994: 79–83 [original combination].
Chrysina citlaltepetlamayatli (Blackaller-Bages and Delgado) [new combination by Hawks 2001: 7].

###### Distribution.

MEXICO: Querétaro, Veracruz (Blackaller-Bages and Delgado 1994, Krajcik 2008, Thomas et al. 2007, Morón and Nogueira 2016).

###### Types.

1 ♂ holotype and 1 ♀ allotype at MXAL (declaration by authors of final deposition at CNIN [UNAM]) (Blackaller-Bages and Delgado 1994); paratypes at CAS and MXAL (Blackaller-Bages and Delgado 1994).

###### Remarks.

Morón and Nogueira (2016) considered the valid name for this species to be *Plusiotis citlaltepetlamayatli*. Lacking a clearly articulated and evidence-based rationale for this nomenclatural change, we use the name *Chrysina citlaltepetlamayatli*.

##### Chrysina clypealis

Taxon classificationAnimaliaColeopteraScarabaeidae

(Rothschild & Jordan, 1894)

Plusiotis clypealis Rothschild & Jordan, 1894: 505–506 [original combination].
Chrysina clypealis (Rothschild and Jordan) [new combination by Hawks 2001: 8].

###### Distribution.

COSTA RICA: Cartago, Limón (Rothschild and Jordan 1894, Ohaus 1918, 1934b, Blackwelder 1944, Machatschke 1972, Morón 1990, Hawks 1999, Krajcik 2008, Thomas et al. 2007).

##### Chrysina colima

Taxon classificationAnimaliaColeopteraScarabaeidae

(Morón, 1992)

Plusiotis colima Morón, 1992: 60-62 [original combination].
Chrysina colima (Morón) [new combination by Hawks 2001: 7].

###### Distribution.

MEXICO: Colima, Jalisco (Morón 1992, Thomas et al. 2006, Krajcik 2008).

###### Types.

1 ♂ holotype, 1 ♀ allotype and paratypes at MXAL (Morón 1992); 2 paratypes at ZMHB (Morón 1992).

##### Chrysina confusa

Taxon classificationAnimaliaColeopteraScarabaeidae

(Ohaus, 1913)

Plusiotis confusa Ohaus, 1913: 487–488 [original combination].
Chrysina confusa (Ohaus) [new combination by Hawks 2001: 7].

###### Distribution.

COSTA RICA (Ohaus 1913, 1918, 1934b, Blackwelder 1944, Machatschke 1972, Morón 1990, 1991, Krajcik 2008).

##### Chrysina costata

Taxon classificationAnimaliaColeopteraScarabaeidae

(Blanchard, 1851)

Plusiotis costata Blanchard, 1851: 210 [original combination].
Plusiotis psittacina var. costata Blanchard [new infrasubspecific status by Nonfried 1891: 304].
Plusiotis costata Blanchard [revised species status by Ohaus 1918: 16].
Chrysina costata (Blanchard) [new combination by Hawks 2001: 8].

###### Distribution.

MEXICO: México, Oaxaca, Puebla, Veracruz (Blanchard 1851, Boucard 1875, H. W. Bates 1888, Nonfried 1891, Ohaus 1918, 1934b, Blackwelder 1944, Carrillo et al. 1966, Machatschke 1972, Morón 1981, 1990, 2010, Ratcliffe et al. 1992, Thomas et al. 2006, Krajcik 2008, Muñoz-Hernández et al. 2008, Delgado-Castillo et al. 2012).

###### Types.

Holotype of *Chrysina costata* at MNHN.

##### Chrysina crassimargo

Taxon classificationAnimaliaColeopteraScarabaeidae

(Rothschild & Jordan, 1894)

Plusiotis crassimargo Rothschild & Jordan, 1894: 506 [original combination].
Chrysina crassimargo (Rothschild and Jordan) [new combination by Hawks 2001: 7].

###### Distribution.

MEXICO: Colima, Guerrero, Jalisco, México, Michoacán (Rothschild and Jordan 1894, Ohaus 1918, 1934b, Blackwelder 1944, Machatschke 1972, Morón 1990, García-Montiel et al. 2003, Thomas et al. 2006, Krajcik 2008, Deloya et al. 2014).

##### Chrysina cunninghami

Taxon classificationAnimaliaColeopteraScarabaeidae

(Curoe, 1999)

Plusiotis cunninghami Curoe, 1999: 1–4 [original combination].
Chrysina cunninghami (Curoe) [new combination by Hawks 2001: 8].

###### Distribution.

PANAMA: Bocas del Toro (Curoe 1999, Krajcik 2008, Thomas et al. 2007).

###### Types.

**Figure 10.**
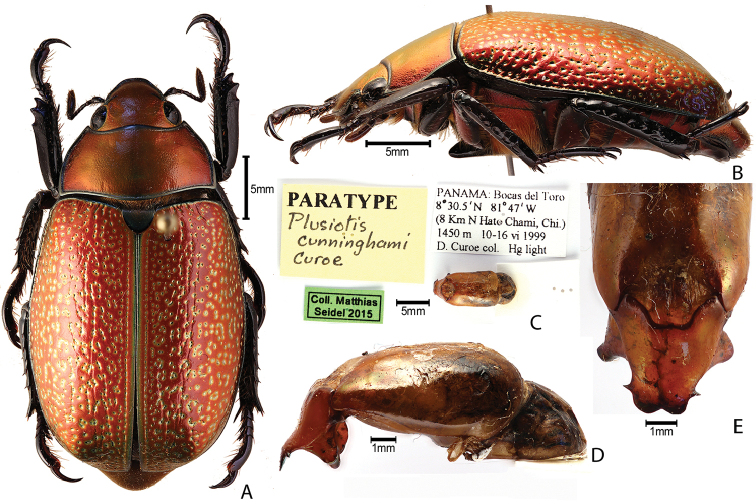
*Plusiotis cunninghami* (Curoe) (valid name *Chrysina cunninghami* [Curoe]) paratype male from MSPC. **A** Dorsal habitus **B** Lateral habitus **C** Specimen labels and male genitalia **D** Male genitalia, lateral view **E** Male parameres, caudal view.

1 ♂ holotype at MIUP (Curoe 1999); 1 ♀ allotype at UNSM (Curoe 1999); paratypes at MXAL (Curoe 1999); 1 ♂ paratype at MSPC (Fig. 10).

##### Chrysina cupreomarginata

Taxon classificationAnimaliaColeopteraScarabaeidae

(F. Bates, 1904)

Plusiotis cupreomarginata F. Bates, 1904: 272 [original combination].
Chrysina cupreomarginata (F. Bates) [new combination by Hawks 2001: 7].

###### Distribution.

COSTA RICA: Cartago, San José (F. Bates 1904, Ohaus 1918, 1934b, Blackwelder 1944, Machatschke 1972, Morón 1990, Thomas et al. 2006, Krajcik 2008).

###### Types.

1 ♂ lectotype and 1 ♀ paralectotype at BMNH (Natural History Museum 2014, BHG pers. obs. Aug. 2016).

##### Chrysina curoei

Taxon classificationAnimaliaColeopteraScarabaeidae

(Warner, LeBlanc, Hawks, & Bruyea, 1992)

Plusiotis curoei Warner, LeBlanc, Hawks, & Bruyea, 1992: 96–99 [original combination].
Chrysina curoei (Warner, LeBlanc, Hawks, and Bruyea) [new combination by Hawks 2001: 8].

###### Distribution.

COSTA RICA: San José (Warner et al. 1992, Krajcik 2008, Thomas et al. 2007).

###### Types.

1 ♂ holotype at CAS (Warner et al. 1992); 1 ♀ allotype at JPBC (Warner et al. 1992).

##### Chrysina cusuquensis

Taxon classificationAnimaliaColeopteraScarabaeidae

(Curoe, 1994)

Plusiotis cusuquensis Curoe, 1994: 35–37, 38 [original combination].
Chrysina cusuquensis (Curoe) [new combination by Hawks 2001: 7].

###### Distribution.

HONDURAS: Cortés (Curoe 1994, Krajcik 2008, Thomas et al. 2009, Jocque et al. 2013).

###### Types.

1 ♂ holotype and 1 ♀ allotype at CAS (Curoe 1994); paratypes at EAPZ, CNIN (UNAM), BMNH, MNHN, ZMHB and MXAL (Curoe 1994). The following specimen is deposited at CCECL. 1 ♂ paratype: “HONDURAS: CUSUCO EL CANTIL 1840 m 1-10/VII/94 luz Hg BOSQUE SECUNDARIO PINO LATIFOLIADO D. CUROE COL.//PARATIPO *Plusiotis cusuquensis* Curoe” (47030024). Genitalia card-mounted underneath specimen. Box 4618644 SOULA.

##### Chrysina dianae

Taxon classificationAnimaliaColeopteraScarabaeidae

(Ratcliffe & Taylor, 1992)

Plusiotis dianae Ratcliffe & Taylor, 1992: 62–63 [original combination].
Chrysina dianae (Ratcliffe and Taylor) [new combination by Hawks 2001: 7].

###### Distribution.

MEXICO: Veracruz, Oaxaca (Ratcliffe et al. 1992, Cano and Morón 1994, Thomas et al. 2006, Krajcik 2008).

###### Types.

1 ♂ holotype at MXAL (Ratcliffe et al. 1992); 1 ♀ allotype at UNSM (Ratcliffe et al. 1992); 2 paratypes at ZMHB (Ratcliffe et al. 1992).

##### Chrysina difficilis

Taxon classificationAnimaliaColeopteraScarabaeidae

(Morón, 1990)

Plusiotis difficilis Morón, 1990: 19–20 [original combination].
Chrysina difficilis (Morón) [new combination by Hawks 2001: 7].

###### Distribution.

MEXICO: Hidalgo, Querétaro, Tlaxcala (Morón 1990, Blackaller-Bages and Delgado 1994, Thomas et al. 2006, Delgado-Castillo and Márquez 2006, Krajcik 2008, Márquez 2008, Márquez et al. 2013, Morón and Nogueira 2016).

###### Types.

1 ♀ holotype and 1 paratype at MXAL (Morón 1990).

###### Remarks.

Morón and Nogueira (2016) considered the valid name for this species to be *Plusiotis difficilis*. Lacking a clearly articulated and evidence-based rationale for this nomenclatural change, we use the name *Chrysina difficilis*.

##### Chrysina diversa

Taxon classificationAnimaliaColeopteraScarabaeidae

(Ohaus, 1912)

Plusiotis diversa Ohaus, 1912: 306–307 [original combination].
Chrysina diversa (Ohaus) [new combination by Hawks 2001: 7].
Chalcochlamys nobilis Ohaus, 1935 **synonym.**
Chalcochlamys nobilis Ohaus, 1935: 125 [original combination].
Chrysina diversa (Ohaus) [syn. Jameson and Ratcliffe 2011: 39].

###### Distribution.

BELIZE: Cayo (Gillett 2009). GUATEMALA: Alta Verapaz, Quiche (Cano and Morón 1994, Thomas et al. 2006). MEXICO: Chiapas, Oaxaca, Tabasco, Veracruz (Ohaus 1912, 1918, 1934b, Blackwelder 1944, Machatschke 1972, Morón 1990, 1991, Morón et al. 1985, 1997, Thomas 1993, Lobo and Morón 1993, Cano and Morón 1994, Thomas et al. 2006, Krajcik 2008).

##### Chrysina donthomasi

Taxon classificationAnimaliaColeopteraScarabaeidae

Monzón & García, 2011

Chrysina donthomasi Monzón & García, 2011: 5–8 [original combination].

###### Distribution.

MEXICO: Nuevo León (Monzón and García 2011, Thomas et al. 2012).

###### Types.

1 ♂ holotype and 1 ♀ allotype at CNIN (UNAM) (Monzón and García 2011).

##### Chrysina dzidorhum

Taxon classificationAnimaliaColeopteraScarabaeidae

(Arnaud, 1994)

Plusiotis dzidorhum Arnaud, 1994: 36–37 [original combination].
Chrysina dzidorhum (Arnaud) [new combination by Hawks 2001: 8].

###### Distribution.

ECUADOR: Cañar, Pichincha (Arnaud 1994, Paucar-Cabrera 2005, Krajcik 2008).

###### Types.

1 ♂ holotype and 1 ♀ allotype at PAPC (Arnaud 1994); paratypes at BMNH, MNHN, UFRJ and MXAL (Arnaud 1994); 1 ♂ paratype at CMNC.

##### Chrysina ericsmithi

Taxon classificationAnimaliaColeopteraScarabaeidae

(Monzón & Cano, 1999)

Plusiotis ericsmithi Monzón & Cano, 1999: 213–214 [original combination].
Chrysina ericsmithi (Monzón and Cano) [new combination by Hawks 2001: 2].

###### Distribution.

GUATEMALA: Izabal (Monzón and Cano 1999, Krajcik 2008, Thomas et al. 2007).

###### Types.

1 ♂ holotype and 1 ♀ allotype at UVGC (Monzón and Cano 1999); paratypes at UVGC, FSCA, MXAL and UNSM (Monzón and Cano 1999).

##### Chrysina erubescens

Taxon classificationAnimaliaColeopteraScarabaeidae

H. W. Bates, 1889

Chrysina erubescens H. W. Bates, 1889: 411 [original combination].

###### Distribution.

MEXICO: Chihuahua, Durango, Nayarit, Sinaloa (H.W. Bates 1889, Ohaus 1918, 1934b, Blackwelder 1944, Cazier 1951, Machatschke 1972, Morón 1990, Morón and Deloya 1991, Lobo and Morón 1993, Thomas et al. 2006, Krajcik 2008).

###### Types.

Holotype of *Chrysina erubescens* at MNHN.

##### Chrysina expansa

Taxon classificationAnimaliaColeopteraScarabaeidae

(Ohaus, 1913)

Plusiotis expansa Ohaus, 1913: 489–490 [original combination].
Chrysina expansa (Ohaus) [new combination by Hawks 2001: 7].

###### Distribution.

MEXICO: Guerrero, Oaxaca (Ohaus 1913, 1918, 1934b, Blackwelder 1944, Machatschke 1972, Morón 1990, Thomas et al. 2006, Krajcik 2008).

##### Chrysina eyai

Taxon classificationAnimaliaColeopteraScarabaeidae

Curoe, 2012

Chrysina eyai Curoe, 2012: 9–15 [original combination].

###### Distribution.

PANAMA: Darien (Curoe 2012, Thomas et al. 2014).

###### Types.

1 ♂ holotype at EMEC (Curoe 2012).

##### Chrysina flohri

Taxon classificationAnimaliaColeopteraScarabaeidae

(Ohaus, 1905)

Plusiotis flohri Ohaus, 1905: 321 [original combination].
Chrysina flohri (Ohaus) [new combination by Hawks 2001: 8].

###### Distribution.

MEXICO: Durango, Nayarit (Ohaus 1905, 1918, 1934b, Blackwelder 1944, Cazier 1951, Machatschke 1972, Morón 1990, Morón et al. 1997, 1998, Thomas et al. 2006, Krajcik 2008).

##### Chrysina gaellae

Taxon classificationAnimaliaColeopteraScarabaeidae

Ebrard & Soula, 2010

Chrysina gaellae Ebrard & Soula, 2010: 7–9 [original combination].
Chrysina hawksi Monzón, 2010 **synonym.**
Chrysina hawksi Monzón, 2010: 4-7 [original combination].
Chrysina gaellae Ebrard and Soula [syn. by Soula 2011: 83].

###### Distribution.

GUATEMALA: Baja Verapáz, Huehuetenango, Zacapa (Ebrard and Soula 2010, Monzón 2010, Thomas et al. 2016, Morón and Nogueira 2016). MEXICO: Chiapas (Monzón 2010, Thomas et al. 2016, Morón and Nogueira 2016).

###### Types.

1 ♂ holotype of *Chrysina gaellae* at DEPC (Soula and Ebrard 2010).

###### Remarks.

Possibly with ill intentions, the names *Plusiotis hawksi* (Monzón 2010 [Oct. 15]) and *C. gaellae* (Ebrard and Soula 2010 [Oct. 1]) were published the same month (see discussion in Soula 2011: 83). Based on the date of publication provided in Soula (2011), *C. gaellae* is the valid name. Morón and Nogueira (2016) considered the valid name for this species to be *Plusiotis hawksi*. It is possible that they overlooked the description of *C. gaellae* (Ebrard and Soula 2010) and/or the synonymy by Soula (2011). Because Morón and Nogueira (2016) do not revalidate the genus *Plusiotis*, we use the currently valid genus *Chrysina*.

##### Chrysina gaitalica

Taxon classificationAnimaliaColeopteraScarabaeidae

Curoe & Hawks, 2012

Chrysina gaitalica Curoe & Hawks, 2012: 9–15 [original combination in Curoe 2012).

###### Distribution.

PANAMA: Coclé (Curoe 2012).

###### Types.

1 ♂ holotype at UCRC (Curoe 2012).

##### Chrysina giesberti

Taxon classificationAnimaliaColeopteraScarabaeidae

Monzón, 2010

Chrysina giesberti Monzón, 2010: 1 [original combination].

###### Distribution.

GUATEMALA: Huehuetenango, Quiché (Monzón 2010). MEXICO: Veracruz (Monzón 2010).

###### Types.

**Figure 11.**
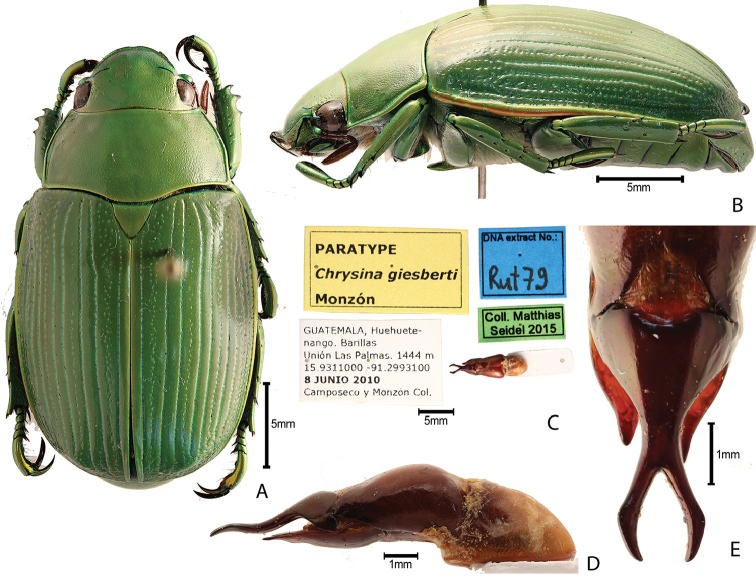
*Chrysina giesberti* Monzón paratype male from MSPC. **A** Dorsal habitus **B** Lateral habitus **C** Specimen labels and male genitalia **D** Male genitalia, lateral view **E** Male parameres, caudal view.

1 ♂ holotype and 1 ♀ allotype at UVGC (Monzón 2010); paratypes at UVGC, FSCA, WSU (Monzón 2010); 2 paratypes at MSPC (Fig. 11).

##### Chrysina gloriosa

Taxon classificationAnimaliaColeopteraScarabaeidae

(LeConte, 1854)

Plusiotis gloriosa LeConte, 1854: 221–222 [original combination].
Chrysina gloriosa (LeConte) [new combination by Hawks 2001: 8].

###### Distribution.

MEXICO: Chihuahua, Coahuila, Sonora (Coolidge 1911, Ohaus 1918, 1934b, Blackwelder 1944, Cazier 1951, Machatschke 1972, Morón 1990, Hardy 1991, Morón et al. 1997, Thomas et al. 2006). USA: Arizona, New Mexico, Texas (Boucard 1875, Nonfried 1891, Skinner 1911, Casey 1915, Ohaus 1918, 1934b, Leng 1920, Blackwelder 1939, 1944, Cazier 1951, Gibson and Carrillo 1959, Machatschke 1972, Morón 1990, 1991, Hardy 1991, Thomas et al. 2006, Krajcik 2008).

##### Chrysina gorda

Taxon classificationAnimaliaColeopteraScarabaeidae

Delgado, 2003

Chrysina gorda Delgado, 2003: 319–321 [original combination].

###### Distribution.

MEXICO: Hidalgo, Querétaro, Veracruz (Delgado 2003, Thomas et al. 2006, Delgado-Castillo and Márquez 2006, Krajcik 2008, Márquez 2008, Márquez et al. 2013).

###### Types.

1 ♂ holotype and 1 ♀ allotype at IEXA (Delgado 2003); paratypes at UAEH (Delgado 2003).

##### Chrysina guatemalensis

Taxon classificationAnimaliaColeopteraScarabaeidae

(Monzón, Cano, & Bailey, 1999)

Plusiotis guatemalensis Monzón, Cano, & Bailey, 1999: 183–184 [original combination].
Chrysina guatemalensis (Monzón, Cano, and Bailey) [new combination by Hawks 2001: 2].

###### Distribution.

GUATEMALA: San Marcos (Monzón et al. 1999, Thomas et al. 2006, Krajcik 2008, Monzón 2010).

###### Types.

1 ♂ holotype and 1 ♀ allotype at UVGC (Monzón et al. 1999); paratypes at UVGC, FSCA, and UNSM (Monzón et al. 1999).

##### Chrysina guaymi

Taxon classificationAnimaliaColeopteraScarabaeidae

(Curoe, 2001)

Plusiotis guaymi Curoe, 2001: 46–49 [original combination].
Chrysina guaymi (Curoe) [**comb. n.**].

###### Distribution.

PANAMA: Chiriquí (Curoe 2001; Thomas et al. 2009).

###### Types.

1 ♂ holotype and 1 ♀ allotype at MIUP (Curoe 2001); paratypes at STRI, MNCR, and MXAL (Curoe 2001).

###### Remarks.

The genus *Plusiotis* was synonymized with *Chrysina* in 2001 (Hawks 2001), the same year that Curoe described a new species from Panama (Curoe 2001). In Hawks’ (2006) online “Checklist of *Chrysina* species”, *Plusiotis guaymi* Curoe is listed as the new combination *Chrysina guaymi*. Because the online checklist is not considered to be formally published for nomenclatural purposes, we formalize this **new combination** herein.

##### Chrysina halffteri

Taxon classificationAnimaliaColeopteraScarabaeidae

(Morón, 1990)

Plusiotis halffteri Morón, 1990: 28 [original combination].
Chrysina halffteri (Morón) [new combination by Hawks 2001: 7].

###### Distribution.

GUATEMALA: Huehuetenango (Thomas et al. 2006). MEXICO: Chiapas (Morón 1990, 1991, Thomas 1993, Thomas et al. 2006, Krajcik 2008).

###### Types.

1 ♂ holotype and 1 ♀ allotype MXAL (Morón 1990).

##### Chrysina howdenorum

Taxon classificationAnimaliaColeopteraScarabaeidae

(Morón, 1990)

Plusiotis howdenorum Morón, 1990: 31–32 [original combination].
Chrysina howdenorum (Morón) [new combination by Hawks 2001: 7].

###### Distribution.

MEXICO: Oaxaca (Morón 1990, Krajcik 2008, Thomas et al. 2007).

###### Types.

The following specimens are deposited at CMNC. 1 ♂ holotype, 1 ♀ allotype, and 1 ♂ paratype: “7000’, 32mi. S. Valle Nacional Oax. Mex. V.21-24, 1971 H. Howden//H. & A. Howden Collection//HOLOTIPO//Plusiotis ♂ howdenorum Morón M. A. Morón, det. 1987//[barcode matrix] Canadian Museum of Musée canadien de la NATURE CMNEN 00011917”, allotype with identical collecting data label and database number CMNEN 00011918; 1 paratype at MXAL (Morón 1990).

##### Chrysina intermedia

Taxon classificationAnimaliaColeopteraScarabaeidae

(Ohaus, 1913)

Plusiotis intermedia Ohaus, 1913: 488–489 [original combination].
Chrysina intermedia (Ohaus) [new combination by Hawks 2001: 7].

###### Distribution.

MEXICO: Oaxaca (Ohaus 1913, 1918, 1934b, Blackwelder 1944, Machatschke 1972, Morón 1990, Krajcik 2008, Thomas et al. 2006).

##### Chrysina karschi

Taxon classificationAnimaliaColeopteraScarabaeidae

(Nonfried, 1891)

Plusiotis karschi Nonfried, 1891: 306 [original combination].
Chrysina karschi (Nonfried) [new combination by Ohaus 1912: 308].

###### Distribution.

GUATEMALA: Baja Verapaz, Zacapa (Monzón 1995, Young 1999, Thomas et al. 2006). HONDURAS: Cortés (Nonfried 1891, Ohaus 1918, 1934b, Blackwelder 1944, Machatschke 1972, Morón 1990, Monzón 1995, Thomas et al. 2006, Krajcik 2008, Jocque et al. 2013).

##### Chrysina lacordairei

Taxon classificationAnimaliaColeopteraScarabaeidae

(Boucard, 1875)

Plusiotis lacordairei Boucard, 1875: 122 [original combination].
Chrysina lacordairei (Boucard) [new combination by Hawks 2001: 7].

###### Distribution.

MEXICO: Guerrero, Oaxaca (Boucard 1875, H. W. Bates 1888, Nonfried 1891, Ohaus 1918, 1934b, Blackwelder 1944, Machatschke 1972, Morón 1990, Cano and Morón 1994, Morón et al. 1997, Thomas et al. 2006, Krajcik 2008, Deloya et al. 2014).

##### Chrysina laniventris

Taxon classificationAnimaliaColeopteraScarabaeidae

(Sturm, 1843)

Pelidnota laniventris Sturm, 1843: 339–340 [original combination].
Plusiotis laniventris (Sturm) [new combination by Burmeister 1844: 420].
Chrysina laniventris (Sturm) [new combination by Hawks 2001: 7].
Pelidnota latipennis Sturm, 1843 **synonym.**
Pelidnota latipennis Sturm, 1843: 338-339 [original combination].
Pelidnota laniventris Sturm [syn. by Burmeister 1844: 420].
Plusiotis mnizechii Boucard, 1875 **synonym.**
Plusiotis mnizechii Boucard, 1875: 124 [original combination].
Plusiotis mniszechii Boucard [incorrect subsequent spelling by Nonfried 1891: 304].
Plusiotis mniszechi Boucard [incorrect subsequent spelling by Ohaus 1918: 16].
Plusiotis laniventris Sturm [syn. by Morón 1990: 23].

###### Distribution.

MEXICO: Distrito Federal, Guerrero, Hidalgo, Jalisco, México, Michoacán, Morelos, Veracruz (Sturm 1843, Burmeister 1844, Boucard 1875, H. W. Bates 1888, Nonfried 1891, Ohaus 1918, 1934b, Blackwelder 1944, Machatschke 1972, Morón and Zaragoza 1976, Morón 1990, Deloya et al. 1993, Morón et al. 1997, Thomas et al. 2006, Delgado-Castillo and Márquez 2006, Krajcik 2008, Márquez 2008, Deloya et al. 2014).

###### Types.

1 ♂ lectotype of *Pelidnota laniventris* at BMNH (Natural History Museum 2014).

###### Remarks.

Boucard (1875) described *Plusiotis mnizechii* from Mexico and named this species for “Count Georges de Mnizech” of the Polish noble Mniszech family. Nonfried (1891) spelled the name *P. mniszechii* and Ohaus (1918) further shortened this to *P. mniszechi*. Ohaus’s (1918) spelling of *P. mniszechi* Boucard was used for this valid species by all subsequent authors until it was synonymized under *P. laniventris* (Sturm) (Ohaus 1918, 1934b, Blackwelder 1944, Machatschke 1972, Morón 1990). We think the original spelling of Boucard (1875) as *P. mnizechii* should be considered correct as there is no evidence of printing error or *lapsus calami* per ICZN Article 32.5. Indeed, Boucard (1878) referred to this species again as *P. mnizechii* in a figure plate legend. The spelling of this synonym has created some confusion in the literature (see “Remarks” under *Chrysina macropus* (Francillon)). If the misspelled name were to be given revalidated species status, it would be at risk of homonymy with Chrysina macropus var. mniszechi H. W. Bates. We stress that correct spelling of this synonym is *Plusiotis mnizechii* Boucard.

##### Chrysina lecontei

Taxon classificationAnimaliaColeopteraScarabaeidae

(Horn, 1882)

Plusiotis lecontei Horn, 1882: 120–121 [original combination].
Plusiotina lecontei (Horn) [new combination by Casey 1915: 87].
Plusiotis lecontei Horn [revised combination by Ohaus 1918: 17].
Chrysina lecontei (Horn) [new combination by Hawks 2001: 7].
Plusiotina aeruginis Casey, 1915 **synonym.**
Plusiotina aeruginis Casey, 1915: 85 [original combination].
Plusiotis aeruginis (Casey) [new combination by Ohaus 1934b: 62].
Plusiotis lecontei Horn [syn. by Cazier 1951: 7].
Plusiotina angusta Casey, 1915 **synonym.**
Plusiotina angusta Casey, 1915: 86 [original combination].
Plusiotis angustata (Casey) [new combination and incorrect subsequent spelling by Ohaus 1934b: 62].
Plusiotis lecontei Horn [syn. by Cazier 1951: 7].
Plusiotina sonorica Casey, 1915 **synonym.**
Plusiotina sonorica Casey, 1915: 87 [original combination].
Plusiotis sonorica (Casey) [new combination by Ohaus 1934b: 63].
Plusiotis lecontei Horn [syn. by Cazier 1951: 7].
Plusiotina subenodis Casey, 1915 **synonym.**
Plusiotina subenodis Casey, 1915: 86 [original combination].
Plusiotis subenodis (Casey) [new combination by Ohaus 1934b: 63].
Plusiotis lecontei Horn [syn. by Cazier 1951: 7].

###### Distribution.

MEXICO: Chihuahua, Durango, Sinaloa, Sonora (H. W. Bates 1888, Coolidge 1911, Casey 1915, Ohaus 1918, 1934b, Blackwelder 1939, 1944, Cazier 1951, Machatschke 1972, Morón 1990, Hardy 1991, Blackaller-Bages and Delgado 1994, Thomas et al. 2006, Morón and Nogueira 2016). USA: Arizona, New Mexico (Horn 1882, H. W. Bates 1888, Nonfried 1891, Casey 1915, Leng 1920, Blackwelder 1939, Ohaus 1918, 1934b, Cazier 1951, Gibson and Carrillo 1959, Hardy 1991, Morón 1990, 1991, Morón and Deloya 1991, Blackaller-Bages and Delgado 1994, Thomas et al. 2006, Krajcik 2008, Morón and Nogueira 2016).

###### Remarks.

Krajcik (2008) listed *Plusiotis angustata* Machatschke (1972) in synonymy with *Chrysina lecontei* (Horn). This is misleading as *P. angustata* is not a validly described species but rather is a subsequent misspelling by Ohaus (1934b) of *P. angusta* Casey. Morón and Nogueira (2016) considered the valid name for this species to be *Plusiotis lecontei*. Lacking a clearly articulated and evidence-based rationale for this nomenclatural change, we use the name *Chrysina lecontei*.

##### Chrysina limbata

Taxon classificationAnimaliaColeopteraScarabaeidae

(Rothschild & Jordan, 1894)

Plusiotis limbata Rothschild & Jordan, 1894: 505 [original combination].
Chrysina limbata (Rothschild and Jordan) [new combination by Hawks 2001: 8].

###### Distribution.

COSTA RICA: Cartago, Limón, San José (Morón 1990, Thomas et al. 2006, Krajcik 2008, García-López et al. 2013). PANAMA: Chiriquí (Morón 1990, Ratcliffe 2002, Thomas et al. 2006).

##### Chrysina luteomarginata

Taxon classificationAnimaliaColeopteraScarabaeidae

(Ohaus, 1913)

Plusiotis luteomarginata Ohaus, 1913: 492–493 [original combination].
Chrysina luteomarginata (Ohaus) [new combination by Hawks 2001: 4].

###### Distribution.

COSTA RICA: Alajuela, Cartago (Morón 1990, Monzón 1995, Young 2002, Thomas et al. 2006, García-López et al. 2013). GUATEMALA: Izabal (Monzón 1995, Young 2002). HONDURAS: Cortés (Thomas et al. 2006). NICARAGUA (Ohaus 1913, 1918, 1934b, Blackwelder 1944, Machatschke 1972, Maes 1987, Morón 1990, Monzón 1995, Hawks 2001, Young 2002, Krajcik 2008). PANAMA: Chiriquí, Veraguas (Morón 1990, Monzón 1995, Ratcliffe 2002, Young 2002, Thomas et al. 2006).

###### Types.

1 ♂ lectotype at ZMHB (Hawks 2001).

##### Chrysina macropus

Taxon classificationAnimaliaColeopteraScarabaeidae

(Francillon, 1795)

Scarabaeus macropus Francillon, 1795: 1–4 [original combination].
Trichius macropus (Francillon) [new combination by Gyllenhaal 1817: 262].
Chrysina macropus (Francillon) [new combination by Burmeister 1844: 416].
Chrysina mexicana Gray, 1832 **synonym.**
Chrysina mexicana Gray, 1832: 516–517 [original combination].
Chrysina macropus (Francillon) [syn. by Burmeister 1844: 416].
Chrysina macropus mniszechi H. W. Bates, 1888 **synonym.**
Chrysina macropus var. mniszechi H. W. Bates, 1888: 285 [original combination].
Chrysina macropus mniszechi H. W. Bates [new subspecific status by Machatschke 1972: 17].
Chrysina macropus (Francillon) [syn. by Morón 1990: 54].

###### Distribution.

MEXICO: Guerrero, Hidalgo, Oaxaca, Puebla, Querétaro, San Luis Potosí, Veracruz (Burmeister 1844, Gistel 1850, Blanchard 1851, H. W. Bates 1888, Nonfried 1891, Ohaus 1918, 1934b, Blackwelder 1944, Gibson and Carrillo 1959, Carrillo et al. 1966, Machatschke 1972, Morón 1985, 1990, 1993, 1994, Morón et al. 1997, Hawks 2001, Thomas et al. 2006, Krajcik 2008, Pacheco Flores et al. 2006, Delgado-Castillo and Márquez 2006, Márquez 2008, Muñoz-Hernández et al. 2008, Delgado-Castillo et al. 2012, Márquez et al. 2013, Deloya et al. 2014).

###### Remarks.

The name “Chrysina henrybatesi” is cited as a synonym of *C. macropus* on the website checklist of *Chrysina* species (Hawks 2006). As discussed by Moore and Jameson (2013), this name was proposed in a manuscript version of Hawks (2001), and it was removed before publication. Apparently the manuscript version of the published paper (Hawks 2001) was used for the website checklist. This name is a *nomen nudum* and should not be listed as a synonym of *C. macropus*.

The names *C. macropusmniszechi* H. W. Bates and *C. mnizechii* Boucard have created some confusion (see “Remarks” under *C. laniventris*). The spelling of these names differs, but revalidation of species status would risk homonymy.

##### Chrysina magnistriata

Taxon classificationAnimaliaColeopteraScarabaeidae

(Morón, 1990)

Plusiotis magnistriata Morón 1990: 32 [original combination].
Chrysina magnistriata (Morón) [new combination by Hawks 2001: 7].

###### Distribution.

PANAMA: Chiriquí (Morón 1990, 1991, Ratcliffe 2002, Thomas et al. 2006, Krajcik 2008).

###### Types.

1 ♂ holotype and 1 ♀ allotype at MNHN (Morón 1990).

##### Chrysina marginata

Taxon classificationAnimaliaColeopteraScarabaeidae

(Waterhouse, 1871)

Plusiotis marginata Waterhouse, 1871: 5–6 [original combination].
Chrysina marginata (Waterhouse) [new combination by Hawks 2001: 7].

###### Distribution.

COSTA RICA: Cartago, Limón, Puntarenas (Morón 1990, Cano and Morón 1994, Hawks 1999, Thomas et al. 2006, Krajcik 2008). PANAMA: Chiriquí (Waterhouse 1871, Boucard 1875, H. W. Bates 1888, Nonfried 1891, Ohaus 1918, 1934b, Blackwelder 1944, Machatschke 1972, Morón 1990, 1991, Cano and Morón 1994, Hawks 1999, Ratcliffe 2002, Thomas et al. 2006).

###### Types.

1 ♀ holotype at BMNH (Natural History Museum 2014).

##### Chrysina miguelangeli

Taxon classificationAnimaliaColeopteraScarabaeidae

Nogueira & Curoe, 2012

Chrysina miguelangeli Nogueira & Curoe, 2012: 2–5 [original combination].

###### Distribution.

MEXICO: Oaxaca (Nogueira and Curoe 2012, Thomas et al. 2014).

###### Types.

1 ♂ holotype and 1 ♀ allotype at MXAL (Nogueira and Curoe 2012); paratypes at MXAL, CNIN (UNAM), UAG and IEXA (Nogueira and Curoe 2012).

##### Chrysina misteca

Taxon classificationAnimaliaColeopteraScarabaeidae

(Morón, 1990)

Plusiotis misteca Morón, 1990: 37 [original combination].
Chrysina misteca (Morón) [new combination by Hawks 2001: 8].

###### Distribution.

MEXICO: Oaxaca (Morón 1990, Krajcik 2008, Thomas et al. 2010).

###### Types.

The following specimens are deposited at CMNC. 1 ♂ holotype: “MEXICO: Oaxaca Disto. de Yautepec Juquila Mixes VI. 1973 W. Miller//H. & A. Howden Collection//HOLOTIPO//Plusiotis ♂ misteca Morón M. A. Morón, det. 1989//[barcode matrix] Canadian Museum of Musée canadien de la NATURE CMNEN 00011919”. 2 paratypes at MXAL (Morón 1990).

##### Chrysina modesta

Taxon classificationAnimaliaColeopteraScarabaeidae

(Sturm, 1843)

Pelidnota modesta Sturm, 1843: 338 [original combination].
Chrysina macropus var. modesta (Sturm) [new combination and new infrasubspecific status by H. W. Bates 1888: 285].
Chrysina modesta (Sturm) [revised species status by Ohaus 1912: 308–309].

###### Distribution.

MEXICO: México, Michoacán (Sturm 1843, H. W. Bates 1888, Ohaus 1912, 1918, 1934b, Blackwelder 1944, Machatschke 1972, Morón 1990, Thomas et al. 2006, Krajcik 2008).

##### Chrysina moroni

Taxon classificationAnimaliaColeopteraScarabaeidae

(Curoe & Beraud, 1994)

Plusiotis moroni Curoe & Beraud, 1994: 31–33 [original combination].
Chrysina moroni (Curoe and Beraud) [new combination by Hawks 2001: 7].

###### Distribution.

GUATEMALA: San Marcos (Thomas et al. 2006). MEXICO: Chiapas (Morón et al. 1997, Morón-Ríos and Morón 2001, Thomas et al. 2006, Krajcik 2008).

###### Types.

1 ♂ holotype and 1 ♀ allotype at CNIN (UNAM) (Curoe and Beraud 1994); paratypes at UVGC and MXAL (Curoe and Beraud 1994).

##### Chrysina nogueirai

Taxon classificationAnimaliaColeopteraScarabaeidae

(Morón, 1992)

Plusiotis nogueirai Morón, 1992: 62-66 [original combination].
Chrysina nogueirai (Morón) [new combination by Hawks 2001: 7].

###### Distribution.

MEXICO: Aguascalientes, Jalisco (Morón 1992, Thomas et al. 2006, Krajcik 2008).

###### Types.

1 ♂ holotype, 1 ♀ allotype and paratypes at MXAL (Morón 1992); paratypes at ZMHB (Morón 1992).

##### Chrysina ofidiodontophallica

Taxon classificationAnimaliaColeopteraScarabaeidae

Curoe, 2011

Chrysina ofidiodontophallica Curoe, 2011: 2–4 [original combination].

###### Distribution.

PANAMA: Darien (Curoe 2011).

###### Types.

1 ♂ holotype at MIUP (Curoe 2011); 1 paratype at MXAL (Curoe 2011).

##### Chrysina ohausi

Taxon classificationAnimaliaColeopteraScarabaeidae

(Franz, 1928)

Plusiotis ohausi Franz, 1928: 3–5 [original combination].
Chrysina ohausi (Franz) [new combination by Hawks 2001: 8].

###### Distribution.

PANAMA (Franz 1928, Ohaus 1934b, Blackwelder 1944, Machatschke 1972, Morón 1990, Curoe 2001, Ratcliffe 2002, Krajcik 2008).

###### Remarks.

*Chrysina ohausi* (Franz) was known only from a single holotype female described from “Panama” and the holotype is apparently lost (Morón 1990; Curoe 2001). It is possible that *C. ohausi* (Franz) is a junior synonym of *C. batesi* (Boucard) based on the description of *C. ohausi* (Franz) and comparisons with other *Chrysina* species from Panama (Curoe 2001).

##### Chrysina optima

Taxon classificationAnimaliaColeopteraScarabaeidae

(H. W. Bates, 1888)

Plusiotis optima H. W. Bates, 1888: 279 [original combination].
Chrysina optima (H. W. Bates) [new combination by Hawks 2001: 8].
Plusiotis melior Rothschild & Jordan, 1894 **synonym.**
Plusiotis melior Rothschild & Jordan, 1894: 506 [original combination].
Plusiotis optima var. melior Rothschild and Jordan [new infrasubspecific status by Ohaus 1934b: 64].
Plusiotis optima melior (Rothschild and Jordan) [new subspecific status by Machatschke 1972: 16].
Plusiotis optima H. W. Bates [syn. by Morón 1990: 47].

###### Distribution.

COSTA RICA: Cartago (H. W. Bates 1888, Nonfried 1892, Rothschild and Jordan 1894, H. W. Bates 1888, Ohaus 1918, 1934b, Blackwelder 1944, Machatschke 1972, Morón 1990, Thomas et al. 2006, Krajcik 2008). PANAMA: Chiriquí (Morón 1990, Ratcliffe 2002, Thomas et al. 2006).

###### Types.

1 ♂ holotype of *Plusiotis optima* at BMNH (Natural History Museum 2014).

##### Chrysina oreicola

Taxon classificationAnimaliaColeopteraScarabaeidae

(Morón, 1992)

Plusiotis oreicola Morón, 1992: 72–73 [original combination].
Chrysina oreicola (Morón) [new combination by Hawks 2001: 8].

###### Distribution.

COSTA RICA: Limón (Morón 1992, Krajcik 2008, Thomas et al. 2013). PANAMA: Chiriquí (Ratcliffe 2002).

###### Types.

1 ♂ holotype at MNCR (Morón 1992); 1 ♀ allotype at MXAL (Morón 1992).

###### Remarks.

Hawks (2001), in reference to species groups in the genus *Chrysina*, listed *C. oreicola* as “*incertae sedis*” (meaning that he was not able to assign the species to a species group). In this usage, Hawks (2001) was not suggesting that the validity of the species was in question.

##### Chrysina orizabae

Taxon classificationAnimaliaColeopteraScarabaeidae

(H. W. Bates, 1889)

Plusiotis orizabae H. W. Bates, 1889: 410 [original combination].
Chrysina orizabae (H. W. Bates) [new combination by Hawks 2001: 5].
Plusiotis alticola H. W. Bates, 1889 **synonym.**
Plusiotis alticola H. W. Bates, 1889: 409–410 [original combination].
Chrysina orizabae (H. W. Bates) [syn. by Hawks 2001: 5].

###### Distribution.

MEXICO: Colima, Distrito Federal, Hidalgo, Jalisco, México, Morelos, Puebla, Tlaxcala, Veracruz (H. W. Bates 1889, Nonfried 1892, Casey 1915, Ohaus 1918, 1934b, Blackwelder 1944, Machatschke 1972, Morón 1990, Blackaller-Bages and Delgado 1994, Morón et al. 1997, Hawks 2001, Thomas et al. 2006, Krajcik 2008, Morón and Márquez 2012, Márquez et al. 2013, Morón and Nogueira 2016).

###### Types.

**Figure 12.**
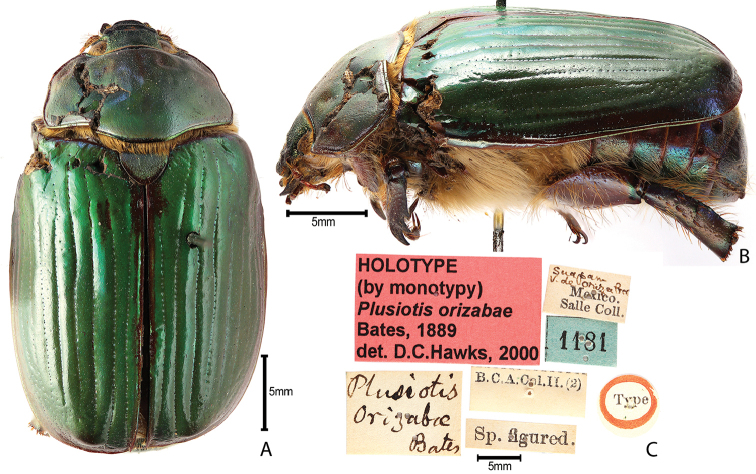
*Plusiotis orizabae* H. W. Bates (valid name *Chrysina orizabae* [H. W. Bates]) holotype from BMNH. **A** Dorsal habitus **B** Lateral habitus **C** Specimen labels.

1 ♀ holotype of *Plusiotis orizabae* at BMNH (Fig. 12); 1 ♂ holotype of *Pelidnota alticola* at BMNH.

###### Remarks.

Krajcik (2012, 2013) erroneously listed *C. alticola* as a valid name. Morón and Nogueira (2016) considered the valid name for this species to be *Plusiotis orizabae*. Lacking a clearly articulated and evidence-based rationale for this nomenclatural change, we use the name *Chrysina orizabae*.

##### Chrysina pastori

Taxon classificationAnimaliaColeopteraScarabaeidae

(Curoe, 1994)

Plusiotis pastori Curoe, 1994: 37–39 [original combination].
Chrysina pastori (Curoe) [new combination by Hawks 2001: 8].

###### Distribution.

HONDURAS: Cortés (Curoe 1994, Krajcik 2008, Thomas et al. 2007, Jocque et al. 2013).

###### Types.

1 ♂ holotype and 1 ♀ allotype at CAS (Curoe 1994); paratypes at EAPZ, CNIN (UNAM) and MXAL (Curoe 1994).

##### Chrysina pehlkei

Taxon classificationAnimaliaColeopteraScarabaeidae

(Ohaus, 1930)

Plusiotis pehlkei Ohaus, 1930b: 265–266 [original combination].
Chrysina pehlkei (Ohaus) [new combination by Hawks 2001: 7].

###### Distribution.

GUATEMALA: Chimaltenango, El Quiché, Quetzaltenango, Sacatepéquez, San Marcos, Sololá (Ohaus 1930b, 1934b, Blackwelder 1944, Machatschke 1972, Morón 1990, 1991, Blackaller-Bages and Delgado 1994, Thomas et al. 2006, Krajcik 2008, Morón and Nogueira 2016).

###### Remarks.

*Chrysina pehlkei* was reported from Chiapas and Oaxaca, Mexico (Thomas 1993, Morón et al. 1997), but these data refer to *C. rutelidedundeei* (pers. comm. from Don Thomas, Aug. 2016). Morón and Nogueira (2016) considered the valid name for this species to be *Plusiotis pehlkei*. Lacking a clearly articulated and evidence-based rationale for this nomenclatural change, we use the name *Chrysina pehlkei*.

##### Chrysina peruviana

Taxon classificationAnimaliaColeopteraScarabaeidae

Kirby, 1828

Chrysina peruviana Kirby 1828: 523 [original combination].
Chrysina macropus (Francillon) [syn. by Burmeister 1844: 416].
Chrysina peruviana Kirby [revised species status by Hawks 2001: 2].
Pelidnota aeruginosa Sturm, 1843 **synonym.**
Pelidnota aeruginosa Sturm, 1843: 337 [original combination].
Chrysina amoena (Sturm) [syn. by Burmeister 1844: 417].
Plusiotis hoegei Boucard, 1895 **synonym.**
Plusiotis högei Boucard, 1895: 4–5 [original combination].
Chrysina hoegei Boucard [emendation by Machatschke 1972: 17].
Chrysina amoena (Sturm) [syn. by Ohaus 1912: 308].
Pelidnota amoena Sturm, 1843 **synonym.**
Pelidnota amoena Sturm, 1843: 337–338 [original combination].
Chrysina amoena (Sturm) [new combination by Burmeister 1844: 417].
Chrysina peruviana Kirby [syn. by Hawks 2001: 2].

###### Distribution.

MEXICO: Hidalgo, Puebla, Querétaro, San Luis Potosí, Veracruz (Sturm 1843, Burmeister 1844, H. W. Bates 1888, Ohaus 1934b, Blackwelder 1944, Machatschke 1972, Morón 1990, Morón et al. 1997, Hawks 2001, Thomas et al. 2006, Delgado-Castillo and Márquez 2006, Krajcik 2008, Muñoz-Hernández et al. 2008, Márquez 2008, Márquez and Sierra-Martínez 2009, Márquez et al. 2013).

###### Types.

1 ♀ holotype at OUMNH (Hawks 2001).

###### Remarks.

Krajcik (2012, 2013) erroneously listed *C. amoena* as a valid name and omitted the name *C. peruviana*. *Chrysina peruviana* was reported from Guanajuato, Mexico (Morón and Márquez 2012), but these data refer to *C. aurilisternum* (Pérez-Flores et al. 2016).

##### Chrysina plusiotina

Taxon classificationAnimaliaColeopteraScarabaeidae

(Ohaus, 1912)

Pelidnota plusiotina Ohaus, 1912: 304–305 [original combination].
Pelidnotopsis plusiotina (Ohaus) [new combination by Ohaus 1915b: 257].
Chrysina plusiotina (Ohaus) [new combination by Hawks 2001:4].
Pelidnotopsis plusiotina (Ohaus) [revised combination by Soula 2010b: 11].
Chrysina plusiotina (Ohaus) [revised combination by Moore and Jameson 2013: 381].

###### Distribution.

MEXICO: Coahuila, Nuevo León (Ohaus 1912, 1915b, 1918, 1934b, Blackwelder 1944, Machatschke 1972, Morón 1990, Hawks 2001, Thomas et al. 2006, Krajcik 2008, Soula 2010b).

###### Types.

1 ♀ lectotype and 1 paralectotype at ZMHB (Hawks 2001).

###### Remarks.

Krajcik (2012, 2013), perhaps erroneously, considered *Pelidnotopis plusiotina* as valid.

##### Chrysina prasina

Taxon classificationAnimaliaColeopteraScarabaeidae

(Boucard, 1878)

Plusiotis prasina Boucard, 1878: 295 [original combination].
Chrysina prasina (Boucard) [new combination by Hawks 2001: 7].

###### Distribution.

MEXICO: Guerrero, Hidalgo, Oaxaca, Puebla, Veracruz (Boucard 1878, H. W. Bates 1888, Nonfried 1892, Ohaus 1918, 1934b, Blackwelder 1944, Machatschke 1972, Morón 1990, 1993, 1994, Ratcliffe et al. 1992, Morón et al. 1997, Thomas et al. 2006, Delgado-Castillo and Márquez 2006, Krajcik 2008, Márquez 2008, Márquez et al. 2013).

##### Chrysina prototelica

Taxon classificationAnimaliaColeopteraScarabaeidae

(Morón & Howden, 1992)

Plusiotis prototelica Morón & Howden, 1992: 205–209 [original combination].
Chrysina prototelica (Morón and Howden) [new combination by Hawks 2001: 7].

###### Distribution.

GUATEMALA: Baja Verapáz, Guatemala, Sacatepéquez (Morón 1991, Morón and Howden 1992, Monzón 1995, Krajcik 2008, Thomas et al. 2007).

###### Types.

The following specimens are deposited at CMNC. 1 ♂ holotype: “GUATML. B. Verapaz 5 km S Sn Jeronimo May 24-31 1989 4500’ J. E. Wappes//No match at BM 1990//SEM//HOLOTIPO//Plusiotis ♂ prototelica Morón-Howden M. A. Morón, det. 1990//[barcode matrix] Canadian Museum of Musée canadien de la NATURE CMNEN 00000040”, 4 ♂ and 5 ♀ paratypes at CMNC. 1 ♀ allotype at MXAL (Morón and Howden 1992); paratypes at UVGC and MXAL (Morón and Howden 1992).

##### Chrysina psittacina

Taxon classificationAnimaliaColeopteraScarabaeidae

(Sturm, 1843)

Pelidnota psittacina Sturm, 1843: 340 [original combination].
Plusiotis auripes (Gray) [syn. by Burmeister 1844: 419].
Plusiotis psittacina (Sturm) [revised species status by Boucard 1875: 123].
Chrysina psittacina (Sturm) [new combination and revised application by Hawks 2001: 3].
Plusiotis amalia Burmeister, 1844 **synonym.**
Plusiotis amalia Burmeister, 1844: 422 [original combination].
Plusiotis laeta Sturm [syn. by Boucard 1875: 121].
Plusiotis psittacina Sturm [syn. by H. W. Bates 1888: 281].
Pelidnota laeta Sturm, 1843 **synonym.**
Pelidnota laeta Sturm, 1843: 341 [original combination].
Plusiotis psittacina (Sturm) [syn. by Boucard 1878: 294].
Plusiotis adelaidae (Hope) [syn. by Nonfried 1891: 302].
Plusiotis psittacina var. laeta (Sturm) [syn. by Ohaus 1918: 17].

###### Distribution.

MEXICO: Chiapas, Hidalgo, Nuevo León, San Luis Potosi, Tamaulipas (Sturm 1843, Burmeister 1844, H. W. Bates 1888, Nonfried 1891, Ohaus 1918, 1934b, Blackwelder 1944, Machatschke 1972, Morón 1981, 1990, Thomas 1993, Hawks 2001, Thomas et al. 2006, Krajcik 2008, Márquez et al. 2013).

###### Types.

1 ♀ lectotype of *Pelidnota psittacina* at BMNH (Hawks 2001).

###### Remarks.

In reference to species groups, Hawks (2001) listed *C. amalia* as “*incertae sedis*” (that is, Hawks was not able to assign the species to a species group). In his usage of “*incertae sedis*”, Hawks (2001) was not suggesting that the validity of the species was in question. H. W. Bates (1888) noted that *P. amalia* had uncertain locality data but synonymized the species under *Plusiotis psittacina*. Morón (1990) maintained the synonymy. We cautiously list *C. amalia* here in synonymy with *Chrysina psittacina* until the validity of the species is reevaluated.

##### Chrysina purpurata

Taxon classificationAnimaliaColeopteraScarabaeidae

(Morón, 1990)

Plusiotis purpurata Morón, 1990: 20–21 [original combination].
Chrysina purpurata (Morón) [new combination by Hawks 2001: 7].

###### Distribution.

MEXICO: Guerrero (Morón 1990, Blackaller-Bages and Delgado 1994, Thomas et al. 2006, Krajcik 2008, Morón and Nogueira 2016).

###### Types.

1 ♂ holotype and 1 ♀ allotype at MXAL (Morón 1990); 1 paratype at CNIN (UNAM) (Morón 1990); 1 paratype at ZMHB (Morón 1990).

###### Remarks.

Morón and Nogueira (2016) considered the valid name for this species to be *Plusiotis purpurata*. Lacking a clearly articulated and evidence-based rationale for this nomenclatural change, we use the name *Chrysina purpurata*.

##### Chrysina purulhensis

Taxon classificationAnimaliaColeopteraScarabaeidae

(Warner & Monzón, 1993)

Plusiotis purulhensis Warner & Monzón, 1993: 211–213 [original combination].
Chrysina purulhensis (Warner & Monzón, 1993) [new combination Hawks 2001: 2].

###### Distribution.

BELIZE: Cayo (Warner and Monzón 1993, Thomas et al. 2006). GUATEMALA: Alta Verapáz, Huehuetenango, Quiché (Warner and Monzón 1993, Thomas et al. 2006, Krajcik 2008, Monzón 2010).

###### Types.

1 ♂ holotype at FSCA (Warner and Monzón 1993); 1 ♀ allotype at USNM (Warner and Monzón 1993); paratypes at FSCA and MXAL (Warner and Monzón 1993).

##### Chrysina quetzalcoatli

Taxon classificationAnimaliaColeopteraScarabaeidae

(Morón, 1990)

Plusiotis quetzalcoatli Morón, 1990: 22 [original combination].
Chrysina quetzalcoatli (Morón) [new combination by Hawks 2001: 7].

###### Distribution.

GUATEMALA: Alta Verapaz, El Quiché, Huehuetenango, Jutiapa, Sacatepéquez, San Marcos (Morón 1990, 1991, Thomas et al. 2006, Jocque et al. 2013). HONDURAS: Comayagua, Cortés (Morón 1991, Thomas et al. 2006, Schlein 2011, Jocque et al. 2013). MEXICO: Chiapas (Morón 1990, 1991, Thomas 1993, Morón-Ríos and Morón 2001, Thomas et al. 2006, Krajcik 2008, Jocque et al. 2013).

###### Types.

1 ♂ holotype, 1 ♀ allotype and 5 paratypes at MXAL (Morón 1990); 1 paratype at MNHN (Morón 1990); 2 paratypes at BMNH (Morón 1990, and BHG pers. obs. Aug. 2016); 2 paratypes at ZMHB (Morón 1990).

##### Chrysina quiche

Taxon classificationAnimaliaColeopteraScarabaeidae

(Morón, 1990)

Plusiotis quiche Morón, 1990: 41 [original combination].
Chrysina quiche (Morón) [new combination by Hawks 2001: 8].

###### Distribution.

GUATEMALA: Quetzaltenango, San Marco, Zacapa (Morón 1990, Monzón 1995, Cano and Morón 1994, Monzón et al. 1999, Krajcik 2008, Thomas et al. 2009). MEXICO: Chiapas (Morón-Ríos and Morón 2001).

###### Types.

1 ♂ holotype at MXAL (Morón 1990).

##### Chrysina ratcliffei

Taxon classificationAnimaliaColeopteraScarabaeidae

(Morón, 1990)

Plusiotis ratcliffei Morón, 1990: 44–45 [original combination].
Chrysina ratcliffei (Morón) [new combination by Hawks 2001: 7].

###### Distribution.

COSTA RICA: Limón, Puntarenas (Morón 1990, Hawks 1999, Thomas et al. 2006). PANAMA: Former Canal Zone, Colón, Darien, Panama (Morón 1990, 1991, Cano and Morón 1994, Hawks 1999, Ratcliffe 2002, Thomas et al. 2006, Krajcik 2008).

###### Types.

The following specimen is deposited at CMNC. 1 ♂ holotype “Panamá: Canal Zone Barro Colorado Is. 9°10'N 79°50'W//5. vi. 1977 H. A. Hespenheide//H. & A. Howden Collection//HOLOTIPO//Plusiotis ♂ ratcliffei Morón M. A. Morón, det. 1988//[barcode matrix] Canadian Museum of Musée canadien de la NATURE CMNEN 00011920”; 1 ♂ paratype at CMNC (Morón 1990).

##### Chrysina resplendens

Taxon classificationAnimaliaColeopteraScarabaeidae

(Boucard, 1875)

Plusiotis resplendens Boucard, 1875: 119 [original combination].
Chrysina resplendens (Boucard) [new combination by Hawks 2001: 7].

###### Distribution.

COSTA RICA: Puntarenas, San José (Boucard 1875, H. W. Bates 1888, Nonfried 1891, Ohaus 1934b, Blackwelder 1944, Machatschke 1972, Morón 1990, Thomas et al. 2006, Krajcik 2008). PANAMA: Chiriquí (H. W. Bates 1888, Nonfried 1891, Ohaus 1918, 1934b, Blackwelder 1944, Machatschke 1972, Morón 1990, Ratcliffe 2002, Thomas et al. 2006).

##### Chrysina rodriguezi

Taxon classificationAnimaliaColeopteraScarabaeidae

(Boucard, 1878)

Plusiotis rodriguezi Boucard, 1878: 295 [original combination].
Chrysina rodriguezi (Boucard) [new combination by Hawks 2001: 8].

###### Distribution.

GUATEMALA: Alta Verapaz, Baja Verapaz, Guatemala, Huehuetenango, Quetzaltenango, Quiché (Boucard 1878, Dohrn 1883, H. W. Bates 1888, Nonfried 1892, Ohaus 1918, 1934b, Blackwelder 1944, Machatschke 1972, Morón 1990, Cano and Morón 1994, Thomas et al. 2006, Krajcik 2008, Monzón 2010). MEXICO: Chiapas, Guerrero (H. W. Bates 1888, Ohaus 1918, 1934b, Blackwelder 1944, Machatschke 1972, Thomas 1993, Thomas et al. 2006).

##### Chrysina rutelidedundeei

Taxon classificationAnimaliaColeopteraScarabaeidae

Soula, 2012

Chrysina rutelidedundeei Soula, 2012: 5–6 [original combination].

###### Distribution.

MEXICO: Chiapas, Oaxaca (Thomas 1993, Morón et al. 1997, Soula 2012, Thomas et al. 2014).

###### Types.

The following specimens are deposited at CCECL. 1 ♂ holotype, 1 ♀ allotype: “Mexique Chiapas San Cristobal VII. 2011//Holotype 2012 *Chrysina ebrardi* S. Soula//*Chrysina rutelidedundeei* M. Soula det. 2012 Holotype ♂” (47030025); “Mexique Chiapas San Cristobal VII. 2011// *Chrysina rutelidedundeei* M. Soula det. 2012 Allotype ♀” (47030026). Genitalia card-mounted underneath the male holotype and female allotype. *Chrysina ebrardi* is a manuscript name and an invalid label on the male holotype. Box 4618645 SOULA.

###### Remarks.

The unavailable manuscript name *Chrysina ebrardi* appears on a label underneath the male holotype specimen of *C. rutelidedundeei*. The distributional data from Thomas (1993) and Morón et al. (1997) were associated with the name *C. pehlkei*, but these specimens are *C. rutelidedundeei* (pers. comm. from Don Thomas, Aug. 2016).

##### Chrysina sallaei

Taxon classificationAnimaliaColeopteraScarabaeidae

(Boucard, 1875)

Plusiotis sallaei Boucard, 1875: 123–124 [original combination].
Plusiotis sallei Boucard [incorrect subsequent spelling by Nonfried 1891: 304].
Chrysina sallaei (Boucard) [new combination by Hawks 2001: 7].

###### Distribution.

MEXICO: Hidalgo, Puebla, Veracruz (Boucard 1875, H. W. Bates 1888, Nonfried 1891, Ohaus 1918, 1934b, Blackwelder 1944, Machatschke 1972, Morón 1990, 1993, 1994, Ratcliffe et al. 1992, Morón et al. 1997, Thomas et al. 2006, Delgado-Castillo and Márquez 2006, Krajcik 2008, Márquez 2008, Muñoz-Hernández et al. 2008, Márquez et al. 2013).

##### Chrysina schusteri

Taxon classificationAnimaliaColeopteraScarabaeidae

(Monzón, Cano, & Bailey, 1999)

Plusiotis schusteri Monzón, Cano, & Bailey, 1999: 183, 184–185 [original combination].
Chrysina schusteri (Monzón, Cano, and Bailey) [new combination by Hawks 2001: 2].

###### Distribution.

GUATEMALA: San Marcos (Monzón et al. 1999, Krajcik 2008, Thomas et al. 2009).

###### Types.

1 ♂ holotype and 1 ♀ allotype at UVGC (Monzón et al. 1999); paratypes at UVGC, FSCA and UNSM (Monzón et al. 1999).

##### Chrysina sirenicola

Taxon classificationAnimaliaColeopteraScarabaeidae

(Solís & Morón, 1994)

Plusiotis sirenicola Solís & Morón, 1994: 31, 37–40 [original combination].
Chrysina sirenicola (Solís and Morón) [new combination by Hawks 2001: 7].

###### Distribution.

COSTA RICA: Puntarenas (Solís and Morón 1994, Hawks 1999, Krajcik 2008).

###### Types.

1 ♂ holotype, 1 ♀ allotype and 4 paratypes at MNCR (Solís and Morón 1994); 1 ♂ paratype at CMNC; 3 paratypes at MXAL (Solís and Morón 1994); (Solís and Morón 1994); 2 paratypes at ZMHB (Solís and Morón 1994).

##### Chrysina spectabilis

Taxon classificationAnimaliaColeopteraScarabaeidae

(Ratcliffe & Jameson, 1992)

Plusiotis spectabilis Ratcliffe & Jameson, 1992: 59–61 [original combination].
Chrysina spectabilis (Ratcliffe and Jameson) [new combination by Hawks 2001: 7].

###### Distribution.

HONDURAS: Cortés (Soula 2006, Thomas et al. 2006, Jocque et al. 2013).

###### Types.

1 ♀ holotype at FMNH (Ratcliffe et al. 1992).

##### Chrysina strasseni

Taxon classificationAnimaliaColeopteraScarabaeidae

(Ohaus, 1924)

Plusiotis strasseni Ohaus, 1924: 185–186 [original combination].
Chrysina strasseni (Ohaus) [new combination by Hawks 2001: 8].

###### Distribution.

GUATEMALA: Zacapa (Monzón 1995, Young 2002, Thomas et al. 2006, Jocque et al. 2013). HONDURAS: Cortés, Olancho, Yoro (Ohaus 1924, 1934b, Blackwelder 1944, Machatschke 1972, Morón 1990, 1991, Young 2002, Thomas et al. 2006, Krajcik 2008, Jocque et al. 2013).

##### Chrysina tapantina

Taxon classificationAnimaliaColeopteraScarabaeidae

(Morón, 1992)

Plusiotis tapantina Morón, 1992: 70–72 [original combination].
Chrysina tapantina (Morón) [new combination by Hawks 2001: 7].

###### Distribution.

COSTA RICA: Cartago (Morón 1992, Krajcik 2008, Thomas et al. 2013). PANAMA (Thomas et al. 2013).

###### Types.

1 ♀ holotype at MNCR (Morón 1992).

##### Chrysina taylori

Taxon classificationAnimaliaColeopteraScarabaeidae

(Morón, 1990)

Plusiotis taylori Morón, 1990: 31 [original combination].
Chrysina taylori (Morón) [new combination by Hawks 2001: 7].

###### Distribution.

MEXICO: Hidalgo, Veracruz (Morón 1990, 1994, Morón et al. 1997, Delgado-Castillo and Márquez 2006, Krajcik 2008, Thomas et al. 2007, Márquez 2008, Márquez et al. 2013).

###### Types.

1 ♂ holotype, 1 ♀ allotype and 1 paratype at MXAL (Morón 1990); 1 paratype at ZMHB (Morón 1990).

##### Chrysina tecunumani

Taxon classificationAnimaliaColeopteraScarabaeidae

(Cano & Morón, 1994)

Plusiotis tecunumani Cano & Morón, 1994: 2–8 [original combination].
Chrysina tecunumani (Cano and Morón) [new combination by Hawks 2001: 8].

###### Distribution.

GUATEMALA: El Progreso, Izabal (Cano and Morón 1994, Krajcik 2008, Monzón 2010).

###### Types.

1 ♂ holotype at UVGC (Cano and Morón 1994).

##### Chrysina terroni

Taxon classificationAnimaliaColeopteraScarabaeidae

(Morón, 1990)

Plusiotis terroni Morón, 1990: 35 [original combination].
Chrysina terroni (Morón) [new combination by Hawks 2001: 7].

###### Distribution.

MEXICO: Hidalgo (Morón 1990, 1993, 1994, Delgado-Castillo and Márquez 2006, Krajcik 2008, Thomas et al. 2007, Márquez 2008, Márquez et al. 2013).

###### Types.

1 ♂ holotype and 1 ♀ allotype at MXAL (Morón 1990).

##### Chrysina transvolcanica

Taxon classificationAnimaliaColeopteraScarabaeidae

(Morón & Nogueira, 2016)

Plusiotis transvolcanica Morón & Nogueira, 2016: 13–15 [original combination].
Chrysina transvolcanica (Morón and Nogueira) [**comb. n.**].

###### Distribution.

MEXICO: Jalisco, Méxcio, Michoacán, Morelos, Puebla, Tlaxcala (Morón and Nogueira 2016).

###### Types.

1 ♂ holotype and 1 ♀ allotype and paratypes at MXAL (Morón and Nogueira 2016).

###### Remarks.

The genus *Plusiotis* was synonymized with *Chrysina* (Hawks 2001). Morón and Nogueira (2016) continued using *Plusiotis* for reasons of practicality and lack of published molecular evidence in support of Hawks’s hypothesis. Because Morón and Nogueira (2016) did not revalidate the genus *Plusiotis*, we transfer *Plusiotis transvolcanica* to the currently valid genus *Chrysina*.

##### Chrysina tricolor

Taxon classificationAnimaliaColeopteraScarabaeidae

(Ohaus, 1922)

Plusiotis tricolor Ohaus, 1922: 323 [original combination].
Chrysina tricolor (Ohaus) [new combination by Hawks 2001: 8].

###### Distribution.

COSTA RICA: Cartago, San José (Ohaus 1922, 1934b, Blackwelder 1944, Machatschke 1972, Morón 1990, 1991, Cano and Morón 1994, Krajcik 2008, Thomas et al. 2007).

##### Chrysina triumphalis

Taxon classificationAnimaliaColeopteraScarabaeidae

Morón, 1990

Chrysina triumphalis Morón, 1990: 54–55 [original combination].

###### Distribution.

GUATEMALA: San Marcos (Young 2002, Thomas et al. 2006). MEXICO: Chiapas (Morón 1990, Thomas 1993, Morón-Ríos and Morón 2001, Young 2002, Thomas et al. 2006, Krajcik 2008).

###### Types.

1 ♂ holotype, 1 ♀ allotype and 1 paratype at MXAL (Morón 1990).

##### Chrysina tuerckheimi

Taxon classificationAnimaliaColeopteraScarabaeidae

(Ohaus, 1913)

Plusiotis türckheimi Ohaus, 1913: 491–492 [original combination].
Plusiotis tuerckheimi Ohaus [justified emendation by Machatschke 1972: 14].
Chrysina turckheimi (Ohaus) [new combination and incorrect subsequent spelling by Hawks 2001: 8].

###### Distribution.

GUATEMALA: San Marcos (Monzón et al. 1999, Thomas et al. 2006). MEXICO: Chiapas (Ohaus 1913, 1918, 1934b, Blackwelder 1944, Machatschke 1972, Morón 1990, Thomas 1993, Cano and Morón 1994, Thomas et al. 2006, Krajcik 2008).

##### Chrysina veraguana

Taxon classificationAnimaliaColeopteraScarabaeidae

(Ohaus, 1922)

Plusiotis veraguana Ohaus, 1922: 324 [original combination].
Chrysina veraguana (Ohaus) [new combination by Hawks 2001: 8].

###### Distribution.

PANAMA: Veraguas (Ohaus 1922, 1934b, Blackwelder 1944, Machatschke 1972, Morón 1990, 1991, Cano and Morón 1994, Ratcliffe 2002, Krajcik 2008).

###### Remarks.

In reference to species groups, Hawks (2001) listed *C. veraguana* as “*incertae sedis*” (that is, Hawks was not able to assign the species to a species group). In this usage, Hawks (2001) was not suggesting that the validity of the species was in question.

##### Chrysina victorina

Taxon classificationAnimaliaColeopteraScarabaeidae

(Hope, 1841)

Pelidnota victorina Hope, 1841: 147 [original combination].
Plusiotis victorina (Hope) [new combination by Burmeister 1844: 418].
Chrysina victorina (Hope) [new combination by Hawks 2001: 7].

###### Distribution.

MEXICO: Chiapas, Oaxaca, Veracruz (Hope 1841, Burmeister 1844, Blanchard 1851, Boucard 1875, H. W. Bates 1888, Nonfried 1891, Ohaus 1918, 1934b, Blackwelder 1944, Machatschke 1972, Morón 1990, Thomas 1993, Morón et al. 1997, Thomas et al. 2006, Krajcik 2008).

##### Chrysina wolfi

Taxon classificationAnimaliaColeopteraScarabaeidae

(Ohaus, 1912)

Plusiotis wolfi Ohaus, 1912: 305 [original combination].
Chrysina wolfi (Ohaus) [new combination by Hawks 2001: 8].

###### Distribution.

ECUADOR: Manabí, Pichincha (Ohaus 1912, 1918, 1934b, Blackwelder 1944, Machatschke 1972, Morón 1990, 1991, Arnaud 1994, Paucar-Cabrera 2005, Krajcik 2008).

##### Chrysina woodi

Taxon classificationAnimaliaColeopteraScarabaeidae

(Horn, 1884)

Plusiotis woodi Horn, 1884: xxxi [original combination].
Plusiotis woodii Horn [incorrect subsequent spelling by Horn 1885: 124].
Chrysina woodii (Horn) [new combination by Hawks 2001: 8].
Chrysina woodi (Horn) [suggested correct spelling by Moore and Jameson 2013: 383].

###### Distribution.

MEXICO: Chihuahua (H. W. Bates 1888, Blackwelder 1944, Cazier 1951, Hardy 1991, Thomas et al. 2006). USA: Texas (Horn 1884, 1885, H. W. Bates 1888, Nonfried 1892, Skinner 1911, Casey 1915, Ohaus 1918, 1934b, Leng 1920, Blackwelder 1939, 1944, Cazier 1951, Machatschke 1972, Morón 1990, 1991, Hardy 1991, Thomas et al. 2006, Krajcik 2008).

##### Chrysina xalixteca

Taxon classificationAnimaliaColeopteraScarabaeidae

(Morón, 1992)

Plusiotis xalixteca Morón, 1992: 66–70 [original combination].
Chrysina xalixteca (Morón) [new combination by Hawks 2001: 8].

###### Distribution.

MEXICO: Jalisco (Morón 1992, Thomas et al. 2006, Krajcik 2008).

###### Types.

1 ♂ holotype, 1 ♀ allotype and paratypes at MXAL (Morón 1992); paratypes at ZMHB (Morón 1992); paratypes at CNIN (UNAM) and ZMHB (Morón 1992).

##### Chrysina zapoteca

Taxon classificationAnimaliaColeopteraScarabaeidae

(Morón, 1990)

Plusiotis zapoteca Morón, 1990: 39–40 [original combination].
Chrysina zapoteca (Morón) [new combination by Hawks 2001: 7].

###### Distribution.

MEXICO: Oaxaca (Morón 1990, Cano and Morón 1994, Thomas et al. 2006, Krajcik 2008).

###### Types.

1 ♂ holotype and 1 paratype at MXAL (Morón 1990). 1 ♀ allotype at CMNC: “20mi. S. Juchatengo 6000’, Oax. Rt. 131, Mex. V.27-30, 1971 H. F. Howden//H. & A. Howden Collection//ALLOTYPE//ALLOTIPO//Plusiotis ♀ zapoteca Morón M. A. Morón, det. 1988//[barcode matrix] Canadian Museum of Musée canadien de la NATURE CMNEN 00011921”.

##### 

Taxon classificationAnimaliaColeopteraScarabaeidae

Dejean, 1821

Chrysophora Dejean, 1821: 60.

###### Type species.

*Melolontha chrysochlora* Latreille, 1812: 131, by monotypy.

###### Gender.

Feminine.

###### Types.

**Figure 13.**
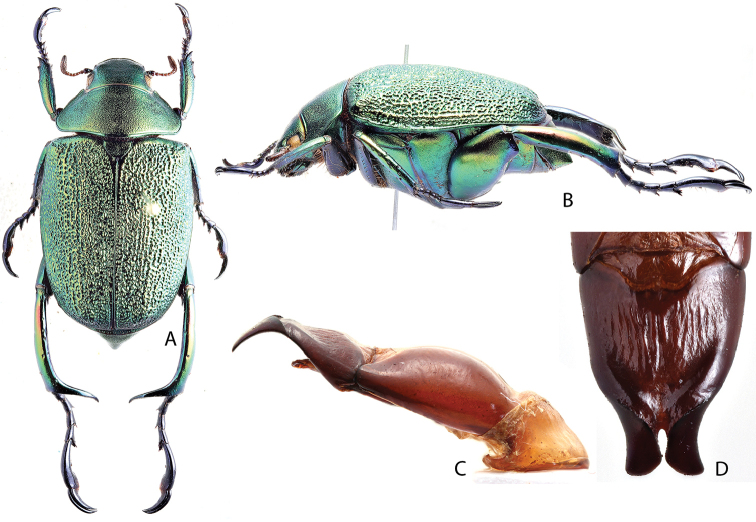
*Chrysophora chrysochlora* (Latreille) male exemplar specimen from MSPC. **A** Dorsal habitus **B** Lateral habitus **C** Male genitalia, lateral view **D** Parameres, dorsal view.

1 species (Fig. 13).

##### Chrysophora chrysochlora

Taxon classificationAnimaliaColeopteraScarabaeidae

(Latreille, 1812)

Melolontha chrysochlora Latreille, 1812: 131 [original combination].
Chrysophora chrysochlora (Latreille) [new combination by Dejean 1821: 60].

###### Distribution.

COLOMBIA: Antioquia, Boyacá, Caquetá, Cauca, Cesar, Chocó, Cundinamarca, Huila, Meta, Nariño, Norte de Santander, Tolima, Valle del Cauca (Guérin-Méneville 1834, Gistel 1850, Nonfried 1891, Gibson and Carrillo 1959, Morón 1990, Restrepo et al. 2003, Neita-Moreno et al. 2006, Pardo-Locarno and Morón 2007, Neita-Moreno 2011, López-García et al. 2015). ECUADOR: Los Ríos (FSCA), Esmeraldas, Guayas, Loja, Morona-Santiago, Napo, Pastaza, Pichincha, Sucumbíos (Blackwelder 1944, Ohaus 1903, 1918, 1934b, 1952, Machatschke 1972, Morón 1990, Paucar-Cabrera 2005, López-García et al. 2015). PERU: Huánuco (FSCA), Junín (FSCA), San Martín (FSCA), Loreto (Latreille 1812, Germar 1815, LePeletier and Serville 1828, Laporte 1840, Blackwelder 1944, Ohaus 1934b, 1952, Machatschke 1972, Morón 1990, Krajcik 2008, Ratcliffe et al. 2015, López-García et al. 2015).

##### 

Taxon classificationAnimaliaColeopteraScarabaeidae

Ohaus, 1915

Ectinoplectron Ohaus, 1915b: 257.

###### Type species.

*Homonyx oryctoides* Ohaus, 1905: 314–315, by monotypy.

###### Gender.

Neuter.

###### Species.

**Figure 14.**
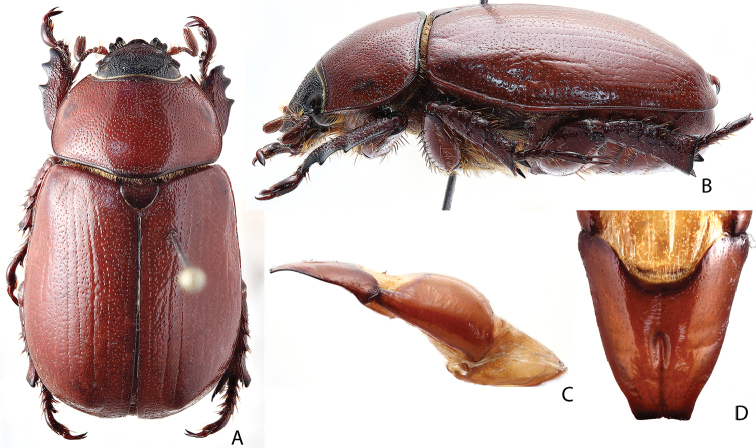
*Ectinoplectron oryctoides* (Ohaus) male exemplar specimen from MSPC. **A** Dorsal habitus **B** Lateral habitus **C** Male genitalia, lateral view **D** Parameres, dorsal view.

1 species (Fig. 14).

##### Ectinoplectron oryctoides

Taxon classificationAnimaliaColeopteraScarabaeidae

(Ohaus, 1905)

Homonyx oryctoides Ohaus, 1905: 314–315 [original combination].
Ectinoplectron oryctoides (Ohaus) [new combination by Ohaus 1915b: 257].
Pelidnota howdeni Hardy, 1975 **synonym.**
Pelidnota howdeni Hardy, 1975: 6, 14-15 [original combination].
Ectinoplectron oryctoides (Ohaus) [syn. by Morón et al. 1997: 26–27].

###### Distribution.

MEXICO: Chihuahua, Durango, Jalisco, Michoacán, Nayarit, Sinaloa, Sonora (Ohaus 1905, 1915b, 1918, 1934b, Blackwelder 1944, Machatschke 1972, Hardy 1975, Morón 1990, Morón et al. 1997, 1998, Bustos-Santana and Rivera-Cervantes 2003, Krajcik 2008, Soula 2009, Lugo et al. 2011, Morón and Márquez 2012, Zamora-Vuelvas et al. 2014).

###### Types.

1 ♂ lectotype of *Homonyx oryctoides* at ZMHB (Soula 2009); 1 ♂ *Pelidnota howdeni* paratype at CMNC.

##### 

Taxon classificationAnimaliaColeopteraScarabaeidae

F. Bates, 1904

Epichalcoplethis F. Bates, 1904: 253, 272–273.
Chalcoplethis Burmeister, 1844 [new synonym by Ohaus 1915b: 258].
Epichalcoplethis F. Bates [revised genus status by Soula 2006: 101].

###### Type species.

*Pelidnota velutipes* Arrow, 1900: 179, by monotypy.

###### Gender.

Feminine.

###### Species.

16 species and subspecies.

###### Remarks.

Krajcik (2012, 2013) considered *Epichalcoplethis* to be a junior synonym of *Pelidnota*.

##### Epichalcoplethis aciculata

Taxon classificationAnimaliaColeopteraScarabaeidae

(F. Bates, 1904)

Pelidnota aciculata F. Bates, 1904: 254, 261 [original combination].
Pelidnota (Chalcoplethis) aciculata F. Bates [new subgeneric combination by Ohaus 1918: 29].
Epichalcoplethis aciculata (F. Bates) [new combination by Soula 2006: 106–107].

###### Distribution.

BOLIVIA: Santa Cruz (WBWC). BRAZIL: Amazonas, Pará (INPA). FRENCH GUIANA: Cayenne, St.-Laurent du Maroni (F. Bates 1904, Ohaus 1918, 1934b, Blackwelder 1944, Machatschke 1972, Krajcik 2008, Soula 2006, 2010c).

###### Types.

**Figure 15.**
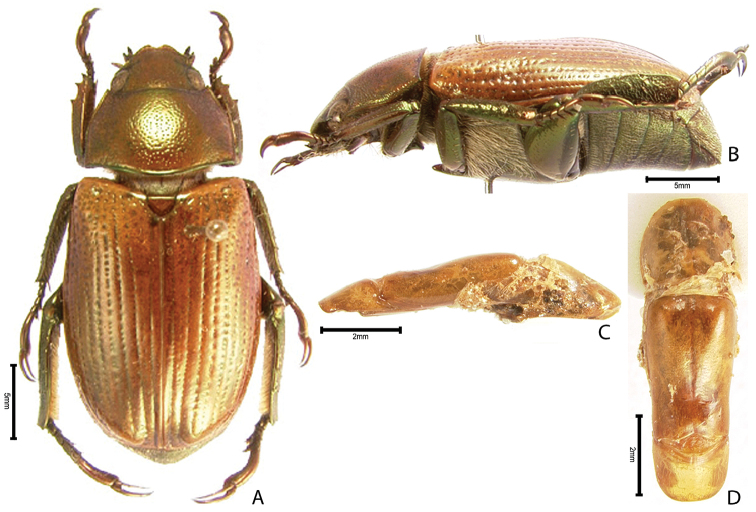
*Epichalcoplethis aciculata* (F. Bates), male specimen. **A** Dorsal habitus **B** Lateral habitus **C** Male genitalia, lateral view **D** Male genitalia, dorsal view.

1 ♂ holotype at IRSNB (Soula 2006). An exemplar specimen is figured (Fig. 15).

##### Epichalcoplethis benjamini

Taxon classificationAnimaliaColeopteraScarabaeidae

Bouchard & Soula, 2006

Epichalcoplethis benjamini Bouchard & Soula, 2006: 102, 107 [original combination].

###### Distribution.

BOLIVIA: La Paz (Soula 2006). PERU (Soula 2006, Ratcliffe et al. 2015).

###### Types.

The following specimens are deposited at CCECL. 1 ♂ holotype, 1 ♀ allotype, 1 ♂ paratype: “Coll. G. Lecourt Ixiamas 11/91 310 m. Bolivie//Holotype *Epichalcoplethis benjamini* 2006 S. Soula” (47030043); “Coll. G. Lecourt Ixiamas 11/91 310 m. Bolivie//Allotype *Epichalcoplethis benjamini* 2006 S. Soula” (47030044); “Coll. G. Lecourt Ixiamas 11/91 310 m. Bolivie//Paratype *Epichalcoplethis benjamini* 2006 S. Soula” (47030045). Genitalia card-mounted underneath male specimens. Box 4618648 SOULA.

**Figure 16.**
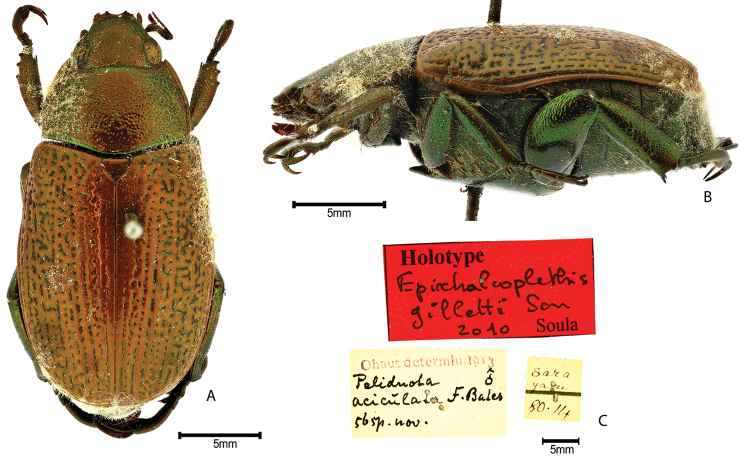
*Epichalcoplethis gilletti* Soula holotype male from BMNH. **A** Dorsal habitus **B** Lateral habitus **C** Specimen labels.

##### Epichalcoplethis blancoi

Taxon classificationAnimaliaColeopteraScarabaeidae

Soula, 2006

Epichalcoplethis blancoi Soula, 2006: 102, 109 [original combination].

###### Distribution.

VENEZUELA: Bolívar, Miranda (Soula 2006).

###### Types.

The following specimens are deposited at CCECL. 1 ♀ holotype, 2 ♀ paratypes: “Camp. Minero Payapal Rio Yuruan//Exp. Instituto Zool. Agricola//Venezuela Bolivar//El Dorado 190 m 23-30-V-87//Holotype *Epichalcoplethis blancoi* S. 2006 Soula” (47030055); “En la luz//VENEZUELA: Bolívar Guri 200 m 27-vi- al 6-vii-1998 L. J. Joly; J. L. García; Y. Zavala//Paratype *Epichalcoplethis blancoi* S. 2006 Soula” (47030056); “VENEZUELA: Miranda Tacarigua de Manporal 10°22’32”N 66°12’10”W 23-v-1998 Col. O. Hernández S.//Paratype 2006 *Epichalcoplethis blancoi* S. Soula Soula” (47030057). This is the entire series and it is noted in Soula (2006) that they are from the MIZA Collection. Box 4618648 SOULA.

##### Epichalcoplethis chamaeleon

Taxon classificationAnimaliaColeopteraScarabaeidae

(Herbst, 1789)

Scarabaeus chamaeleon Herbst, 1789: 247–248 [original combination].
Pelidnota chamaeleon (Herbst) [new combination by Burmeister 1844: 407].
Pelidnota ignita var. chamaeleon (Herbst) [new infrasubspecific status by F. Bates 1904: 259].
Pelidnota (Chalcoplethis) chamaeleon (Herbst) [new subgeneric combination and revised species status by Ohaus 1918: 29].
Epichalcoplethis chamaeleon (Herbst) [new combination by Soula 2006: 108].
Pelidnota equestris Laporte, 1840 **synonym.**
Pelidnota equestris Laporte, 1840: 122 [original combination].
Pelidnota ignita (Olivier) [syn. by Burmeister 1844: 408].
Chalcoplethis chamaeleon (Herbst) [syn. by Ohaus 1918: 29].
Epichalcoplethis chamaeleon (Herbst) [syn. by Soula 2006: 108].
Cetonia ignita Olivier, 1789 **synonym.**
Cetonia ignita Olivier, 1789: 69–70 [original combination].
Rutela ignita (Olivier) [new combination by Schönherr 1817: 150].
Pelidnota ignita (Olivier) [new combination by Burmeister 1844: 407].
Chalcoplethis chamaeleon var. ignita (Olivier) [new combination and new infrasubspecific status by Ohaus 1918: 29].
Chalcoplethis chamaeleon forma ignita (Olivier) [revised infrasubspecific status by Machatschke 1972: 32].
Epichalcoplethis chamaeleon (Herbst) [syn. by Soula 2006: 108].

###### Distribution.

BRAZIL: Amazonas, Roraima (INPA) (Laporte 1840, Ohaus 1918, 1934b, Blackwelder 1944, Machatschke 1972). COLOMBIA: Meta (Burmeister 1844, Blanchard 1851, Ohaus 1918, 1934b, Blackwelder 1944, Machatschke 1972, Restrepo et al. 2003, Krajcik 2008). FRENCH GUIANA: Cayenne, St.-Laurent du Maroni (Olivier 1789, Burmeister 1844, Ohaus 1918, Machatschke 1972, Krajcik 2008, Soula 2010c). GUYANA: Demerara-Mahaica (MNRJ) (Ohaus 1918). SURINAME (Olivier 1789, Ohaus 1934b, Machatschke 1972). TRINIDAD AND TOBAGO: Trinidad (Ohaus 1918, 1934b, Blackwelder 1944, Machatschke 1972, Soula 2006). VENEZUELA (Ohaus 1918, 1934b, Blackwelder 1944, Machatschke 1972, Soula 2006).

###### Remarks.

Voet (1769) is often cited as the author for this species. However, names in Voet’s *Catalogus Systematicus Coleopterorum* (1778 and subsequent editions) are not consistently binomial (ICZN, Art. 11.4). As such, all of Voet’s names are rejected (Löbl and Smetana 2011). Soula (2006) credited Herbst (1769) as the author of the species.

##### Epichalcoplethis gilletti

Taxon classificationAnimaliaColeopteraScarabaeidae

Soula, 2010

Epichalcoplethis gilletti Soula, 2010a: 48–49 [original combination].

###### Distribution.

ECUADOR: Pastaza (Soula 2010a). PERU (Soula 2010a, Ratcliffe et al. 2015).

###### Remarks.

According to Soula (2010a), the type specimen of this species should be conserved in the Soula Collection (CCECL). We located this holotype specimen (Fig. 16) at BMNH with the following data: “sara / yagu [indecipherable word] / [blue line] 80.14 // [printed and handwritten] Ohaus determin. 1913 / Pelidnota ♂ aciculata F. Bates / sbsp. nov. // [red label] [printed and handwritten] Holotype / Epichalcoplethis / gilletti Sou / 2010 Soula”.

##### Epichalcoplethis ledezmaae

Taxon classificationAnimaliaColeopteraScarabaeidae

Bouchard & Soula, 2006

Epichalcoplethis ledezmaae Bouchard & Soula, 2006: 102, 104–105 [original combination].

###### Distribution.

BOLIVIA: Santa Cruz (Soula 2006).

###### Types.

The following specimen is deposited at CCECL. 1 ♂ holotype: “Rte de Camiri à Sta. Cruz Bol. coll. – SOULA //Holotype *Epichalcoplethis ledezmaae* S. 2006 Soula” (47030042). The genitalia are card-mounted underneath this male specimen. Box 4618648 SOULA.

##### Epichalcoplethis monzoni

Taxon classificationAnimaliaColeopteraScarabaeidae

Soula, 2006

Epichalcoplethis monzoni Soula, 2006: 102, 112 [original combination].

###### Distribution.

BELIZE: Cayo (Soula 2006). GUATEMALA: Izabal, Petén (Soula 2006).

###### Types.

The holotype of *Epichalcoplethis monzoni* is deposited at UVGC (Soula 2006). The following specimens are deposited at CCECL. 11 ♂ paratypes, 2 ♀ paratypes: “Finca Firmeza, Sierra de Caral, Morales, Izabal, Guatemala, 450m, 20/V/2006//Paratype 2006 *Epichalcoplethis monzoni* S. Soula” (47030047 to 47030053); five paratypes with identical label data: “Finca Firmeza 20/V/2006 Sierra de Caral, 450 m Morales - Izabal, GUATEMALA José Monzon leg.//Paratype 2006 *Epichalcoplethis monzoni* S. Soula” (47030958 to 47030962); “Chiquibul Forest Reserve, Cayo, Belize, VI/2006//Paratype 2006 *Epichalcoplethis monzoni* S. Soula” (47030054). All male specimens with genitalia card-mounted. Box 4618648 SOULA and 4616345 PORION. The following specimen is deposited at CMNC: 1 ♂ paratype: “GUATEMALA. Izabal Morales. Finca Firmeza Sierra de Caral, 45 msnm 450m. 20 V 2006 José Monzón Coll. COLLECCION J. MONZON//Paratype 2006 *Epichalcoplethis monzoni* S. Soula”. The following paratype is deposited at BMNH: “Belize (Cayo) / Chiquibul Forest Reserve / Las Cuevas Research Station / 16°44'N 88°59'W / June 2006 / BMNH {E} 2006-141 / C. Gillet & J. Kitson // Paratype 2006 / Epichalcoplethis / monzoni S. / Soula”.

##### Epichalcoplethis navarropoloi

Taxon classificationAnimaliaColeopteraScarabaeidae

Soula, 2011

Epichalcoplethis navarropoloi Soula, 2011: 73 [original combination].

###### Distribution.

ECUADOR: Pastaza (Soula 2011).

###### Types.

The holotype ♂ is deposited at the Malý collection (Soula 2011).

##### Epichalcoplethis porioni

Taxon classificationAnimaliaColeopteraScarabaeidae

Soula, 2010

Epichalcoplethis porioni Soula, 2010a: 48 [original combination].

###### Distribution.

HONDURAS: Lempira (Soula 2010a).

###### Types.

The following specimen is deposited at CCECL. 1 ♂ holotype: “HONDURAS-LEMPIRA Montana de Celaque AOUT 1995 Thierry PORION Leg//Coll. TH. PORION//Holotype 2009 *Epichalcoplethis porioni* S. Soula” (47030955). Genitalia card-mounted underneath the male holotype. Box 4616345 PORION.

##### Epichalcoplethis richteri

Taxon classificationAnimaliaColeopteraScarabaeidae

(Ohaus, 1910)

Pelidnota richteri Ohaus, 1910a: 186 [original combination].
Pelidnota (Chalcoplethis) richteri Ohaus [new subgeneric combination by Ohaus 1918: 29].
Epichalcoplethis richteri (Ohaus) [new combination by Soula 2006: 103].

###### Distribution.

BRAZIL: Mato Gross do Sul (Ohaus 1910a, 1918, 1934b; Blackwelder 1944; Machatschke 1972; Krajcik 2008). PARAGUAY: Alto Paraguay (Ohaus 1910a; Soula 2006).

###### Types.

1 type specimen of *Pelidnota richteri* at MLPA.

##### Epichalcoplethis santistebani

Taxon classificationAnimaliaColeopteraScarabaeidae

Bouchard & Soula, 2006

Epichalcoplethis santistebani Bouchard & Soula, 2006: 102, 105 [original combination].

###### Distribution.

PERU: Huánuco (Soula 2006, Ratcliffe et al. 2015).

###### Types.

**Figure 17.**
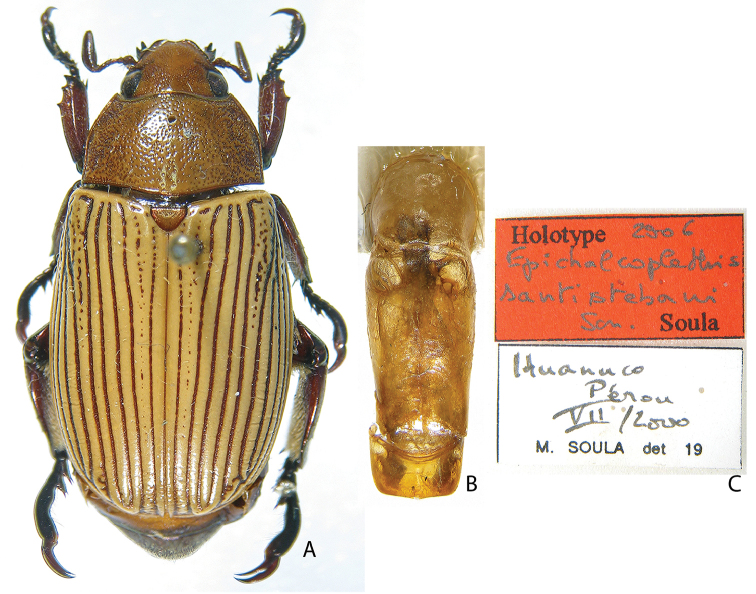
*Epichalcoplethis santistebani* Bouchard and Soula holotype male from CCECL. **A** Dorsal habitus **B** Male genitalia, dorsal view **C** Specimen labels.

The following specimen is deposited at CCECL (Fig. 17). 1 ♂ holotype: “Huanuco Pérou VII/2000 M. SOULA det. 19//Holotype 2006 *Epichalcoplethis santistebani* Sou. Soula” (47030046). Genitalia card-mounted underneath specimen. Box 4618648 SOULA.

##### Epichalcoplethis sanctijacobi

Taxon classificationAnimaliaColeopteraScarabaeidae

(Ohaus, 1905)

Pelidnota sanctijacobi Ohaus, 1905: 318 [original combination].
Pelidnota (Chalcoplethis) sanctijacobi Ohaus [new subgeneric combination by Ohaus 1918: 29].
Epichalcoplethis sanctijacobi (Ohaus) [new combination by Soula 2006: 103–104].

###### Distribution.

ARGENTINA: Córdoba, Salta, Santiago del Estero, Tucumán (Ohaus 1905, 1918, 1934b, Blackwelder 1944, Machatschke 1972, Soula 2006, Krajcik 2008).*B*RAZIL (Soula 2006). FRENCH GUIANA: Mana (Gruner 1971). PARAGUAY (Soula 2006). URUGUAY (Soula 2006).

###### Types.

**Figure 18.**
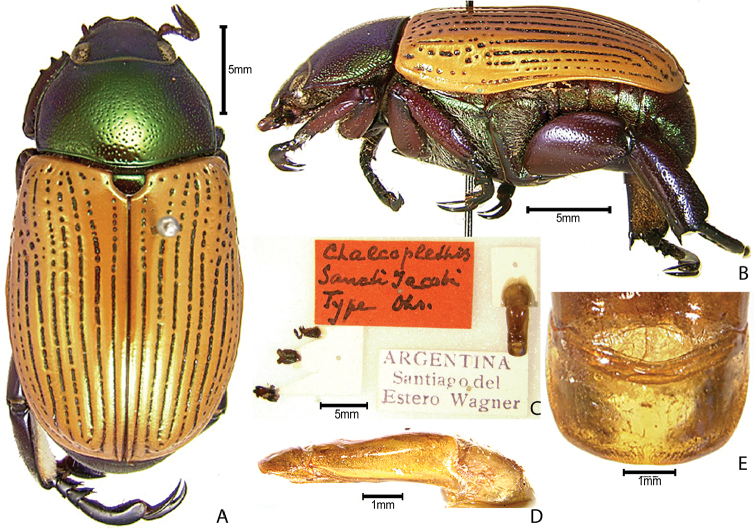
*Pelidnota sanctijacobi* Ohaus (valid name *Epichalcoplethis sanctijacobi* [Ohaus]) type male from ZMHB. **A** Dorsal habitus **B** Lateral habitus **C** Specimen labels, mouthparts, and male genitalia **D** Male genitalia, lateral view **E** Male parameres, dorsal view.

1 ♂ type specimen of *Pelidnota sanctijacobi* at ZMHB (Fig. 18); 1 type specimen at MLPA; 1 type specimen at SDEI; 1 ♂ paralectotype at NHMB (Soula 2006) (see “*Type Specimens and Lectotype Designation*” in Methods).

##### Epichalcoplethis schiffleri

Taxon classificationAnimaliaColeopteraScarabaeidae

Bouchard & Soula, 2006

Epichalcoplethis schiffleri Bouchard & Soula, 2006: 102, 107–108 [original combination].

###### Distribution.

PERU: Loreto, Piura (Soula 2006, Ratcliffe et al. 2015).

###### Types.

The following specimens are deposited at CCECL. 1 ♂ holotype, 4 ♂ paratypes: “Iquitos, Loreto Pérou; VIII/2003//Holotype 2006 *Epichalcoplethis schiffleri* S. Soula” (47030027); “Iquitos, Loreto Pérou; VIII/2003//Paratype 2005 *Epichalcoplethis schiffleri* S. Soula” (47030028); “Iquitos 100 m 9.03 Loreto/PERU//Paratype 2005 *Epichalcoplethis schiffleri* S. Soula” (47030030); “Yamamono River Iquitos (P) 6/88// Paratype 2005 *Epichalcoplethis schiffleri* S. Soula” (47030031); “Carbajal, Rio Itaya Piura, Pérou, IX/2005// Paratype 2005 *Epichalcoplethis schiffleri* S. Soula” (47030029). The Yamamono River locality does not appear in the Soula (2006) description. All 5 specimens have their genitalia card-mounted. Box 4618648 SOULA.

##### Epichalcoplethis seriatopunctata

Taxon classificationAnimaliaColeopteraScarabaeidae

(Ohaus, 1912)

Pelidnota seriatopunctata Ohaus, 1912: 304 [original combination].
Pelidnota (Chalcoplethis) seriatopunctata Ohaus [new subgeneric combination by Ohaus 1918: 29].
Epichalcoplethis seriatopunctata (Ohaus) [new combination by Soula 2006: 102–103].

###### Distribution.

BRAZIL (Ohaus 1912, 1918, 1934b, Blackwelder 1944, Machatschke 1972, Soula 2006, Krajcik 2008).

##### Epichalcoplethis velutipes romeroi

Taxon classificationAnimaliaColeopteraScarabaeidae

Soula, 2006

Epichalcoplethis velutipes romeroi Soula, 2006: 111–112 [original combination].

###### Distribution.

VENEZUELA: Bolívar (Soula 2006).

###### Types.

**Figure 19.**
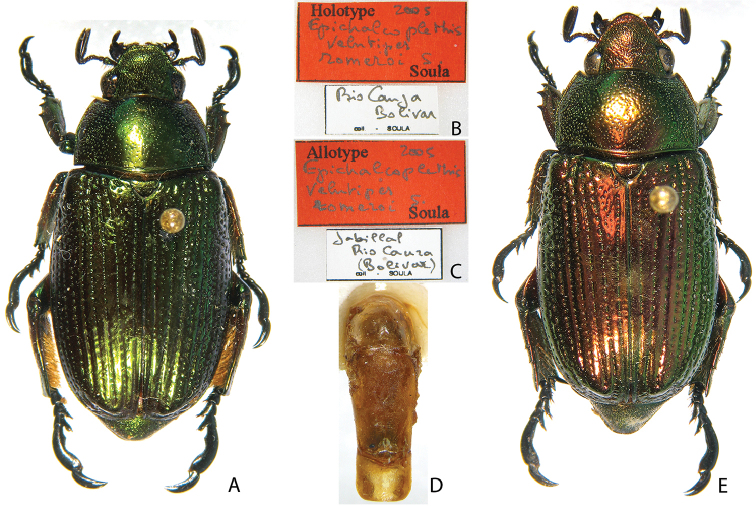
*Epichalcoplethis velutipes romeroi* Soula holotype male and allotype female from CCECL. **A** Dorsal habitus, holotype **B** Specimens labels, holotype **C** Specimen labels, allotype **D** Male genitalia dorsal view, holotype **E** Dorsal habitus, allotype.

The following specimens are deposited at CCECL. 1 ♂ holotype (Fig. 19A, B, D), 1 ♂ invalid holotype, 1 ♀ allotype (Fig. 19C, E), 3 ♂ paratypes, 4 ♀ paratypes: “Rio Cauja Bolivar coll. – SOULA//Holotype 2005 *Epichalcoplethis velutipes romeroi* S. Soula” (47030032); Jabillal Rio Caura (Bolivar) coll – SOULA [obverse] 03/94 //Allotype 2005 *Epichalcoplethis velutipes romeroi* S. Soula” (47030033); “N. Venezuela S. Klages 1904//Not valid Holotype probable paratype det. M. L. Jameson 2014//Holotype *Epichalcoplethis romeroi* Sou. Soula det. 2005” (47030034); “Rio Cauja (Bolivar) coll. – SOULA[obverse] 07/87 Venez. // Pel. (Chalcoplethis) velutipes (Arrow)//Paratype *Epichalcoplethis velutipes romeroi* 2005 Soula” (47030035); “N. Venezuela S. Klages 1904//Pel. (Chalcoplethis) velutipes (Arrow)//Museum Paris ex Coll. R. Oberthur// Paratype *Epichalcoplethis velutipes romeroi* 2005 Soula” (47030036); “P. N. Henri Pittier Choroni ; Venezuela V-VI/2005//Pel. (Chalcoplethis) velutipes (Arrow)//Paratype *Epichalcoplethis velutipes romeroi* S. 2005 Soula” (47030037); “Camp. minero Payapal Rio Yuruan//Exp. Instituto Zool. Agricola//Venezuela Bolivar//El Dorado 190 m 23-30-V-87 //Paratype 2005 *Chalcoplethis velutipes romeroi* S. Soula” (47030038); “Rio Cauja (Bolivar) coll. – SOULA [obverse] 07/87 //Pel. (Chalcoplethis) velutipes (Arrow)//Paratype 2005 *Epichalcoplethis velutipes romeroi* S. Soula” (47030039); “Choroni 200 m V/1998 M. SOULA det. 19 [obverse] Aragua Venezuela (Chez Romero) // Paratype 2005 *Epichalcoplethis velutipes romeroi* S. Soula” (47030040); “VENEZUELA: Bolívar Guri 200 m 27-vi-al 6-vii-1998 L. J. Joly; J. L. García; Y. Zavala//Paratype 2005 *Epichalcoplethis velutipes romeroi* S. Soula” (47030041). Genitalia card-mounted underneath the male holotype, the invalid male holotype, three male paratypes, and one female paratype (47030036, 47030037, 47030041, and 47030039). Box 4618648 SOULA.

###### Remarks.

The male holotype specimen labeled from “N. Venezuela” is in fact not the true holotype specimen according to Soula (2006). We labeled this specimen as a probable paratype.

##### Epichalcoplethis velutipes velutipes

Taxon classificationAnimaliaColeopteraScarabaeidae

(Arrow, 1900)

Pelidnota velutipes Arrow, 1900: 179 [original combination].
Epichalcoplethis velutipes (Arrow) [new combination by F. Bates 1904: 253, 272–273].
Pelidnota (Chalcoplethis) velutipes Arrow [revised combination and new subgeneric combination by Ohaus 1918: 29].
Epichalcoplethis velutipes (Arrow) [new combination by Soula 2006: 109–111].

###### Distribution.

GRENADA (Leng and Mutchler 1914, Machatschke 1972, Hardy 1975, Chalumeau 1985, Soula 2006, Krajcik 2008, Peck 2010, 2016). GUATEMALA: Petén (Hardy 1975, Chalumeau 1985, Soula 2006). HONDURAS: Atlántida (Hardy 1975, Chalumeau 1985, Soula 2006). MEXICO: Chiapas (Palacios-Rios et al. 1990, Thomas 1993, Soula 2006). ST. VINCENT AND THE GRENADINES: St. Vincent (Ohaus 1918, 1934b, Chalumeau 1985, Soula 2006, Peck 2010, 2016). TRINIDAD AND TOBAGO: Trinidad, Tobago (Ohaus 1918, 1934b, Chalumeau 1985, Peck et al. 2002, Peck 2016). VENEZUELA (Ohaus 1918, 1934b, Chalumeau 1985, Soula 2006, Peck 2010, 2016).

###### Types.

1 ♂ type at at BMNH (Hardy 1975).

##### Eremophygus

Taxon classificationAnimaliaColeopteraScarabaeidae

Ohaus, 1910

Eremophygus Ohaus, 1910c: 21–22.
Heterocallichloris Gutíerrez, 1951 **synonym.**
Heterocallichloris Gutíerrez 1951: 112–114. [Type species. *Heterocallichloris bicolor* Gutiérrez, 1951 by original designation].
Platycoelia Dejean [syn. by Machatschke 1965: 55].
Eremophygus Ohaus [syn. by Smith and Jameson 2001: 105].

###### Type species.

*Eremophygus philippii* Ohaus, 1910c: 22, by monotypy.

###### Gender.

Masculine.

###### Species.

6 species.

##### Eremophygus bicolor

Taxon classificationAnimaliaColeopteraScarabaeidae

(Gutiérrez, 1951)

Heterocallichloris bicolor Gutiérrez, 1951: 112, 114 [original combination].
Platycoelia bicolor (Gutiérrez) [new combination by Machatschke 1965: 60].
Eremophygus bicolor (Gutiérrez) [new combination by Smith and Jameson 2001: 105].

###### Distribution.

BOLIVIA (Smith and Jameson 2001).

###### Remarks.

*Heterocallichloris bicolor* was originally described in the ruteline tribe (subtribe ). As a result of a broad analysis of the (Smith 2003), the species was transferred to the genus *Eremophygus* (Smith and Jameson 2001).

##### Eremophygus calvus

Taxon classificationAnimaliaColeopteraScarabaeidae

Gutiérrez, 1952

Eremophygus calvus Gutiérrez, 1952: 223–224 [original combination].

###### Distribution.

BOLIVIA: La Paz (Gutiérrez 1952, Machatschke 1972).

###### Types.

**Figure 20.**
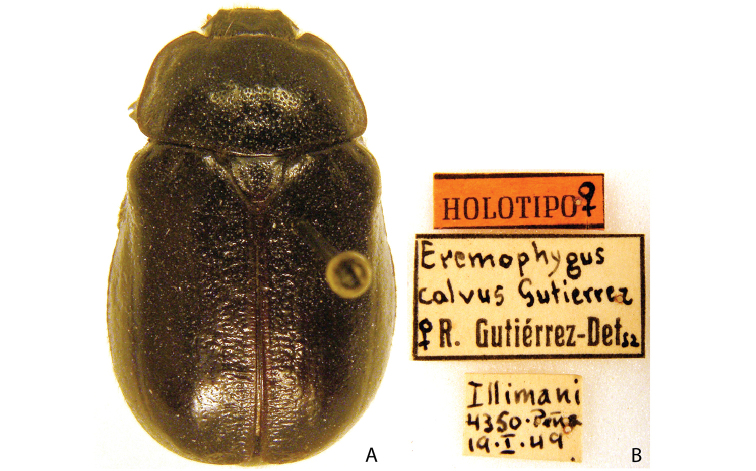
Holotype female of *Eremophygus calvus* Gutiérrez from UCCC. **A** Dorsal habitus **B** Specimen labels.

Holotype ♀ of *Eremophygus calvus* Gutiérrez at UCCC (Fig. 20).

##### Eremophygus lasiocalinus

Taxon classificationAnimaliaColeopteraScarabaeidae

Ohaus, 1915

Eremophygus lasiocalinus Ohaus, 1915a: 76–77 [original combination].

###### Distribution.

BOLIVIA: La Paz (Ohaus 1915a, 1918, 1934b, Blackwelder 1944, Gutiérrez 1949, 1950, Machatschke 1972, Ferrú and Elgueta 2011). CHILE: Arica and Parinacota (Gutiérrez 1949, 1950; Machatschke 1972, Ferrú and Elgueta 2011).

###### Types.

**Figure 21.**
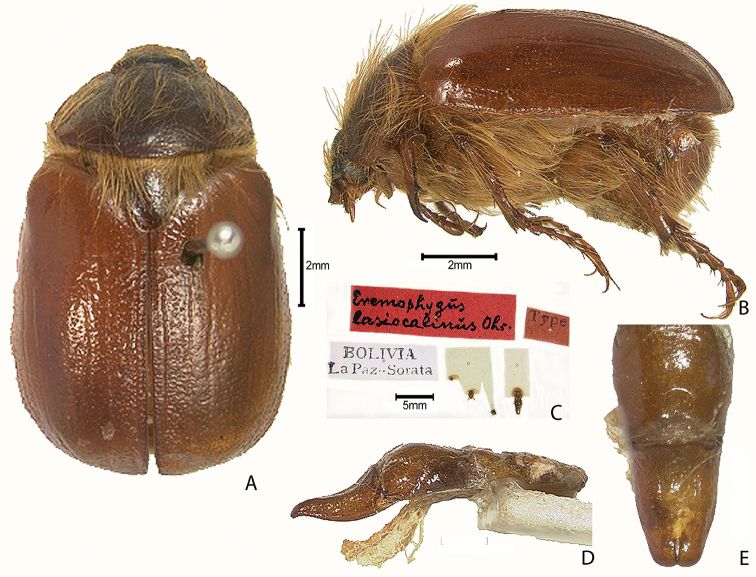
*Eremophygus lasiocalinus* Ohaus holotype male from ZMHB. **A** Dorsal habitus **B** Lateral habitus **C** Specimen labels, mouthparts, and male genitalia **D** Male genitalia, lateral view **E** Parameres, dorsal view.

Holotype ♂ of *Eremophygus lasiocalinus* at ZMHB (Fig. 21).

##### Eremophygus leo

Taxon classificationAnimaliaColeopteraScarabaeidae

Gutiérrez, 1951

Eremophygus leo Gutiérrez, 1951: 106 [original combination].

###### Distribution.

ARGENTINA: Jujuy (Gutiérrez 1951, Machatschke 1972).

###### Types.

**Figure 22.**
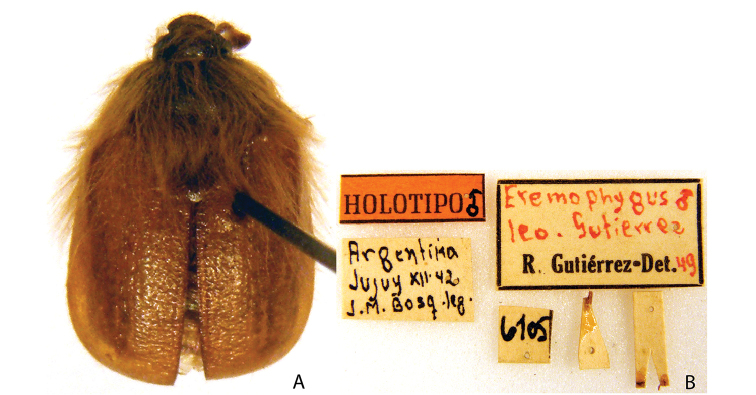
Holotype male of *Eremophygus leo* Gutiérrez from UCCC. **A** Dorsal habitus **B** Specimen labels, mouthparts, and male genitalia.

Holotype ♂ of *Eremophygus leo* Gutiérrez at UCCC (Fig. 22).

##### Eremophygus pachyloides

Taxon classificationAnimaliaColeopteraScarabaeidae

Ohaus, 1925

Eremophygus pachyloides Ohaus, 1925: 76 [original combination].

###### Distribution.

BOLIVIA (Ohaus 1925, 1934b, Blackwelder 1944, Gutiérrez 1949, Machatschke 1972).

###### Types.

**Figure 23.**
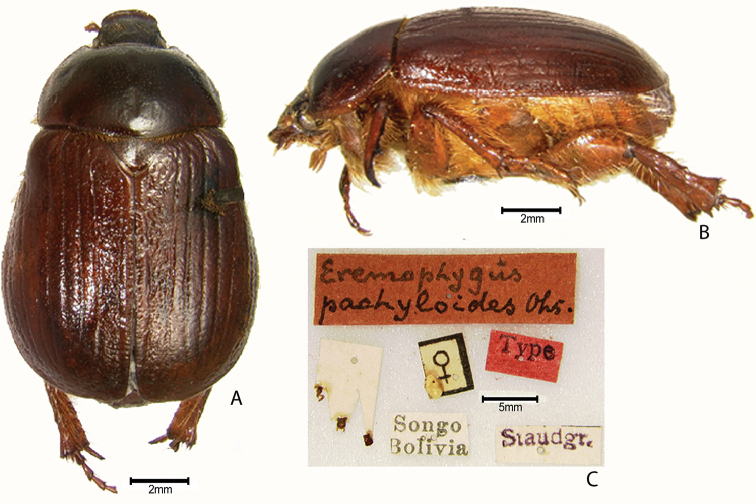
*Eremophygus pachyloides* Ohaus holotype female from ZMHB. **A** Dorsal habitus **B** Lateral habitus **C** Specimen labels, mouthparts, and egg.

Holotype ♀ of *Eremophygus pachyloides* at ZMHB (Fig. 23).

##### Eremophygus philippii

Taxon classificationAnimaliaColeopteraScarabaeidae

Ohaus, 1910

Eremophygus philippii Ohaus, 1910c: 22 [original combination].

###### Distribution.

CHILE: Arica and Parinacota; Tarapacá (Ohaus 1910c, 1918, 1934b, Blackwelder 1944, Gutiérrez 1949, 1950, Machatschke 1972). PERU (Ohaus 1952, Ratcliffe et al. 2015).

###### Types.

**Figure 24.**
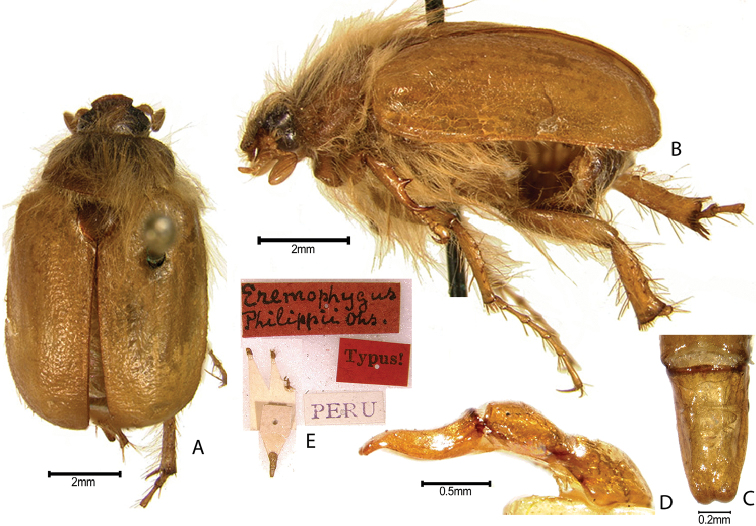
*Eremophygus philippii* Ohaus holotype male from ZMHB. **A** Dorsal habitus **B** Lateral habitus **C** Parameres, dorsal view **D** Male genitalia, lateral view **E** Specimen labels.

Holotype ♂ of *Eremophygus philippii* at ZMHB (Fig. 24).

##### 

Taxon classificationAnimaliaColeopteraScarabaeidae

Soula, 2006

Homeochlorota Soula, 2006: 148–149.

###### Type species.

*Pseudochlorota chiriquina* Ohaus, 1905: 306-307, by monotypy.

###### Gender.

Feminine.

**Figure 25.**
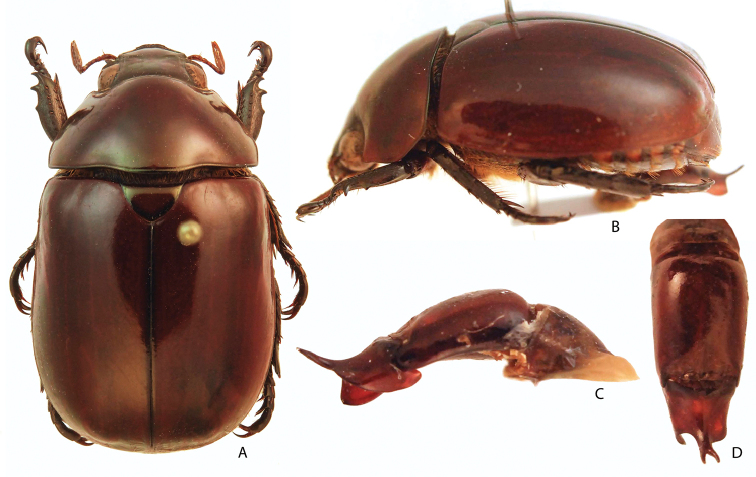
*Homeochlorota chiriquina* (Ohaus) male from DBPC. **A** Dorsal habitus **B** Lateral habitus **C** Male genitalia, lateral view **D** Male parameres, dorsal view.

###### Species.

1 species.

###### Remarks.

Krajcik (2012, 2013) considered *Homeochlorota* to be a synonym of *Pseudochlorota*. Because the rationale for this nomenclatural change was not provided, we use the name *Homeochlorota*.

##### Homeochlorota chiriquina

Taxon classificationAnimaliaColeopteraScarabaeidae

(Ohaus, 1905)

Pseudochlorota chiriquina Ohaus, 1905: 306–307 [original combination].
Homeochlorota chiriquina (Ohaus) [new combination by Soula 2006: 149–150].

###### Distribution.

COSTA RICA: Guanacaste (Soula 2006). PANAMA: Chiriquí (Ohaus 1905, 1918, 1934b, Machatschke 1972, Soula 2006, Krajcik 2008).

###### Types.

Lectotype male of *Pseudochlorota chiriquina* at ZMHB labeled: “Panama, V.d. Chiriqui”; “typus!” (red label, typed); male genitalia card mounted; “Pseudochlorota chiriquina Ohaus” (red label, handwritten). Paralectotype female at ZMHB labeled as lectotype with mouthparts card mounted. An exemplar specimen is shown in Fig. 25.

###### Remarks.

Krajcik (2012, 2013) considered the valid name for this species to be *Pseudochlorota chiriquina*. Lacking his rationale for this nomenclatural change, we use the name *H. chiriquina*.

##### 

Taxon classificationAnimaliaColeopteraScarabaeidae

Guérin-Méneville, 1839

Homonyx Guérin-Méneville, 1839: 299–300.

###### Type species.

*Homonyx cupreus* Guérin-Méneville, 1839: 300, by monotypy.

###### Gender.

Masculine.

###### Species.

14 species and subspecies.

##### Homonyx argentinus

Taxon classificationAnimaliaColeopteraScarabaeidae

Gutiérrez, 1952

Homonyx planicostatus argentinus Gutiérrez, 1952: 224, 225 [original combination].
Homonyx argentinus Gutiérrez [new species status by Soula 2010a: 16].

###### Distribution.

ARGENTINA: Jujuy, Mendoza, Salta, Tucumán (Gutiérrez 1952, Machatschke 1972, Krajcik 2008, Soula 2010a).

###### Types.

1 ♀ paratype at MNNC. 1 ♂ and 5 ♀ paratypes at CMNC. Gutiérrez (1952) stated the holotype male was deposited in his collection at UCCC.

###### Remarks.

Krajcik (2012, 2013) considered *H. argentinus* to be a subspecies of *H. planicostatus*.

##### Homonyx chalceus bahianus

Taxon classificationAnimaliaColeopteraScarabaeidae

Ohaus, 1913

Homonyx bahianus Ohaus, 1913: 495–496 [original combination].
Homonyx chalceus bahianus Ohaus [new species status by Soula 2010a: 14].

###### Distribution.

BRAZIL: Bahia (Ohaus 1913, 1918, 1934b; Machatschke 1972, Krajcik 2008, Soula 2010a).

###### Types.

**Figure 26.**
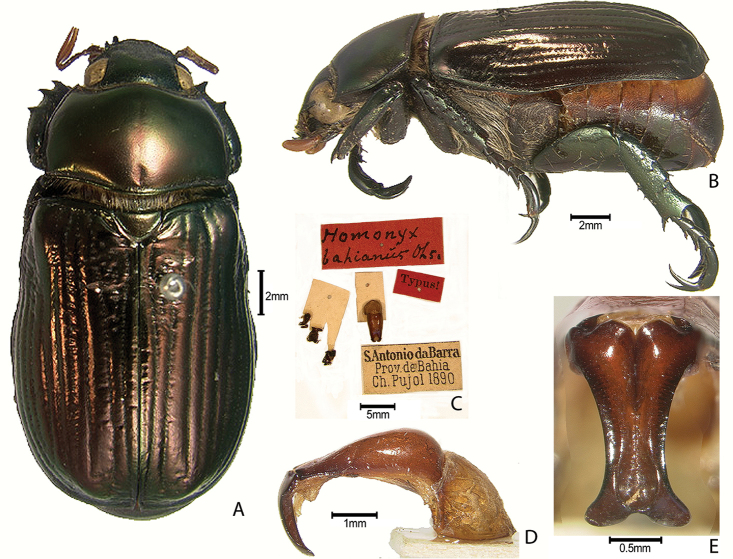
*Homonyx bahianus* Ohaus (valid name *H. chalceus bahianus*) type male (see “*Type specimens and lectotype designation*” in Methods) from ZMHB. **A** Dorsal habitus **B** Lateral habitus **C** Specimen labels, mouthparts, and genitalia **D** Male genitalia, lateral view **E** Parameres, caudal view.

1 ♂ lectotype and 1 paralectotype at ZMHB (Soula 2010a) (Fig. 26).

###### Remarks.

Krajcik (2012, 2013) considered *H. bahianus* to be a valid species rather than a subspecies of *H. chalceus*.

##### Homonyx chalceus chalceus

Taxon classificationAnimaliaColeopteraScarabaeidae

Blanchard, 1851

Homonyx chalceus Blanchard, 1851: 214 [original combination].

###### Distribution.

ARGENTINA: Corrientes, Mendoza, Salta, San Luis (Blanchard 1851, Burmeister 1855, Steinheil 1874, Ohaus 1918, 1934b, Blackwelder 1944, Machatschke 1972, Krajcik 2008, Soula 2010a).

###### Types.

**Figure 27.**
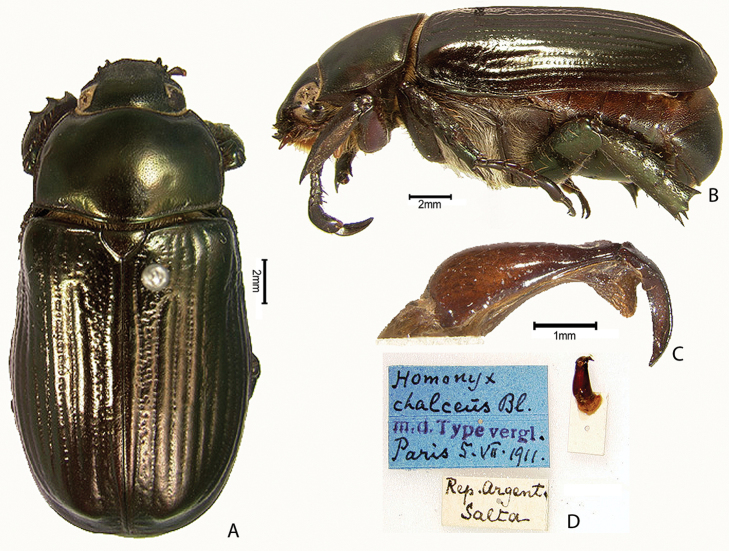
*Homonyx chalceus* Blanchard male (male specimen compared [by Ohaus] with Blanchard’s type deposited at MNHN). **A** Dorsal habitus **B** Lateral habitus **C** Male genitalia, lateral view **D** Specimen labels and male genitalia.

1 ♂ holotype at MNHN (Soula 2010a). An exemplar specimen identified by Ohaus and compared with Blanchard’s type specimen is figured (Fig. 27).

##### Homonyx cupreus

Taxon classificationAnimaliaColeopteraScarabaeidae

Guérin-Méneville, 1839

Homonyx cupreus Guérin-Méneville, 1839: 300 [original combination].

###### Distribution.

ARGENTINA: Corrientes, Salta (Burmeister 1844, Ohaus 1913, 1918, 1934b).

###### Remarks.

*Homonyx cupreus* Guérin-Méneville was erroneously reported from the extreme southern Chilean Magallanes Province and later from the specific locality of Port Famine (modern Puerto del Hambre) (Solier 1851, Reed 1876, Philippi 1887, Ohaus 1910c, 1918, 1934b, Machatschke 1972, Krajcik 2008). This locality is dubius based on the distribution of other known *Homonyx* species, which have their diversity centered in Peru, Ecuador, Bolivia, and central Argentina. Further collecting in southern Chile and southern Argentina is needed to establish whether *Homonyx* species indeed occur there.

##### Homonyx demezi

Taxon classificationAnimaliaColeopteraScarabaeidae

Soula, 2010

Homonyx demezi Soula, 2010a: 23 [original combination].

###### Distribution.

BRAZIL: Mato Grosso (Soula 2010a).

###### Types.

The following specimens are deposited at CCECL. 1 ♂ holotype, 1 ♀ allotype, 3 ♂ paratypes, 1 ♀ paratype: “Mato Grosso Brésil coll. – SOULA//Holotype 2010 *Homonyx demezi* S. Soula (47030999); “Matto Grosso Brésil coll. – SOULA//Allotype 2010 *Homonyx demezi* S. Soula (47031000); “Rosario Matto Grosso M. SOULA det 19 [obverse] 10/61//Paratype 2010 *Homonyx demezi* S. Soula (47031001); “Corumba Matt.//Paratype 2010 *Homonyx demezi* S. Soula (47031002); “Rosario Oeste Matto Grosso 01/72 coll. – Soula [obverse] Rosario Oeste//Paratype 2010 *Homonyx demezi* S. Soula (47031003); “Gob. de Los Andes//Paratype 2009 *Homonyx demezi* S. Soula (47031004). Genitalia card-mounted underneath the male holotype and the three male paratypes. Box 4618689 SOULA. The following specimen is deposited at CMNC. 1 ♀ paratype: “BRASIL Mato Grosso Rosario Oeste A. Maller-leg. Coll. Martínez Oct.-968// H. & A. HOWDEN COLLECTION *ex.* A. Martinez coll.//Paratype 2010 *Homonyx demezi* S. Soula”.

##### Homonyx elongatus

Taxon classificationAnimaliaColeopteraScarabaeidae

Blanchard, 1842

Rutela elongata Blanchard, 1842: plate 11 [original combination].
Homonyx elongatus (Blanchard) [new combination by Blanchard 1851: 214].

###### Distribution.

ARGENTINA (Blackwelder 1944). BOLIVIA: Pando (Blanchard 1851, Burmeister 1855, Ohaus 1918, 1934b, Blackwelder 1944, Machatschke 1972).

###### Types.

1 ♀ holotype at MNHN (Soula 2010a).

##### Homonyx feyeri

Taxon classificationAnimaliaColeopteraScarabaeidae

Ohaus, 1913

Homonyx feyeri Ohaus, 1913: 496–497 [original combination].

###### Distribution.

ECUADOR: Morona-Santiago (Ohaus 1913, 1918, 1934b, Blackwelder 1944, Machatschke 1972, Paucar-Cabrera 2005, Soula 2010a).

###### Types.

**Figure 28.**
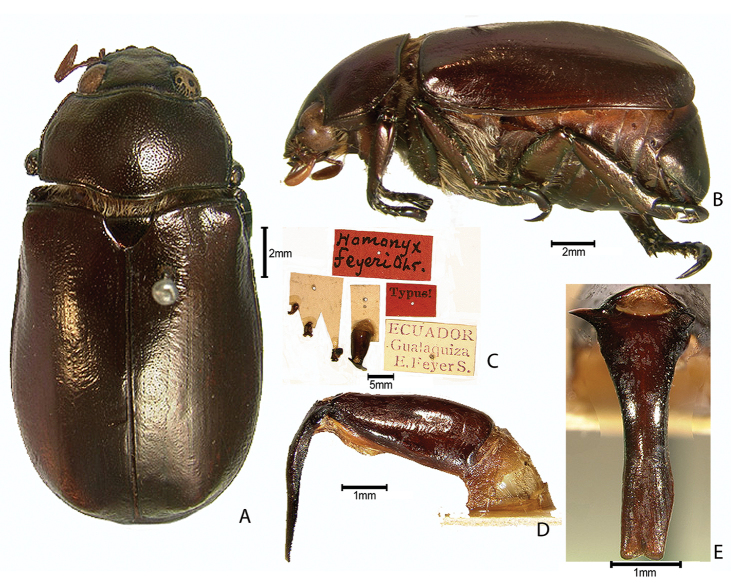
*Homonyx feyeri* Ohaus holotype male from ZMHB. **A** Dorsal habitus **B** Lateral habitus **C** Specimen labels, mouthparts, and male genitalia **D** Male genitalia, lateral view **E** Parameres, caudal view.

1 ♂ holotype specimen of *Homonyx feyeri* Ohaus at ZMHB (Fig. 28). Soula states that ♂ holotype is at ZMHB (Soula 2010a).

##### Homonyx fuscocupreus

Taxon classificationAnimaliaColeopteraScarabaeidae

(Ohaus, 1913)

Homonyx chalceus var. fuscocupreus Ohaus, 1913: 494 [original combination].
Homonyx chalceus fuscocupreus Ohaus [new subspecific status by Machatschke 1972: 19].
Homonyx fuscocupreus Ohaus [new species status by Soula 2011: 73–74].

###### Distribution.

ARGENTINA: Catamarca, Tucumán (Ohaus 1913, 1918, 1934b, Blackwelder 1944, Machatschke 1972, Krajcik 2008, Soula 2010a, 2011).

###### Types.

**Figure 29.**
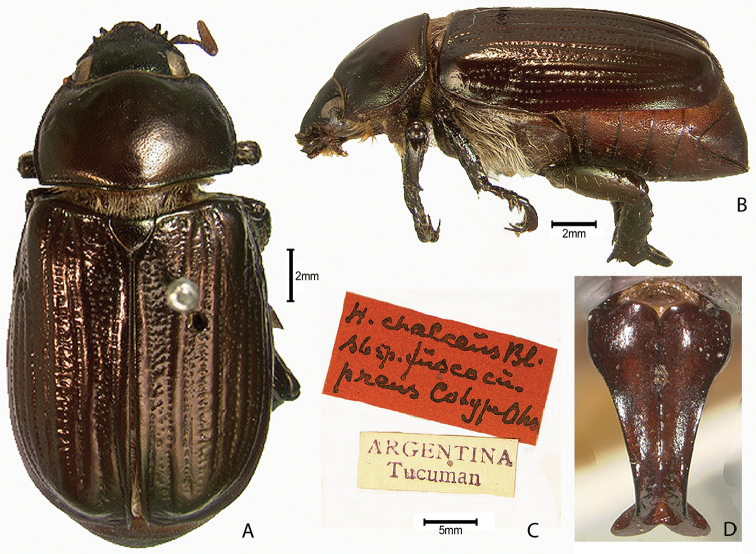
Homonyx chalceus var. fuscocupreus Ohaus (valid name *H. fuscocupreus*) type male from ZMHB. **A** Dorsal habitus **B** Lateral habitus **C** Specimens labels **D** Parameres, caudal view.

1 lectotype and 1 paralectotype at ZMHB (Soula 2010a). An exemplar specimen is shown in Figure 29.

###### Remarks.

Ohaus (1913) described *Homonyx chalceus* ssp. *uruguayanus, Homonyx chalceus* ssp. santiagensis, and Homonyx chalceus var. fuscocupreus. Thus, in the context of this publication, it is unambiguous that Homonyx chalceus var. fuscocupreus is infrasubspecific and should be interpreted in this manner. Some publications have treated the taxon as a subspecies (*Homonyx chalceus fuscocupreus*) according to ICZN Article 45.6.4.1, thus making this species-group name available. *Homonyx fuscocupreus* was elevated to species status by Soula (2011). Krajcik (2012, 2013) considered *H. fuscocupreus* to be a subspecies of *H. chalceus*.

##### Homonyx holligeri

Taxon classificationAnimaliaColeopteraScarabaeidae

Soula, 2010

Homonyx holligeri Soula, 2010a: 19–20 [original combination].

###### Distribution.

BOLIVIA: Santa Cruz (Soula 2010a).

###### Types.

The following specimens are deposited at CCECL. 1 ♂ holotype, 1 ♂ paratype: “Coroïco à Caranavi 850 m 10/88//Holotype 2010 *Homonyx holligeri* S. Soula (47030993); “Bolivia-Dept. Santa Cruz-800 m 25.X.1960-Zischka//Paratype 2010 *Homonyx holligeri* S. Soula (47030994). Genitalia card-mounted underneath the male holotype and the male paratype. Box 4618689 SOULA.

##### Homonyx maurettei

Taxon classificationAnimaliaColeopteraScarabaeidae

Soula, 2010

Homonyx maurettei Soula, 2010a: 18–19 [original combination].

###### Distribution.

PERU: Piura (Soula 2010a, Ratcliffe et al. 2015).

###### Types.

The following specimen is deposited at CCECL. 1 ♂ holotype: “Abra Porculla, Dt. Piura N-W Pérou; 1800m II/2007//Holotype *Homonyx maurettei* S. 2010 Soula (47030998). Genitalia card-mounted underneath the male holotype. Box 4618689 SOULA.

##### Homonyx peruanus

Taxon classificationAnimaliaColeopteraScarabaeidae

Ohaus, 1913

Homonyx planicostatus peruanus Ohaus, 1913: 496 [original combination].
Homonyx elongatus peruanus Ohaus [revised subspecies status by Ohaus 1918: 21].
Homonyx planicostatus peruanus Ohaus [revised subspecies status by Ohaus 1934b: 73].
Homonyx elongatus peruanus Ohaus [revised subspecies status by Ohaus 1952: 2].
Homonyx peruanus Ohaus [new species status by Soula 2010a: 18].

###### Distribution.

PERU (Blackwelder 1944, Ohaus 1913, 1934b, 1952, Machatschke 1972, Soula 2010a, Ratcliffe et al. 2015).

###### Types.

**Figure 30.**
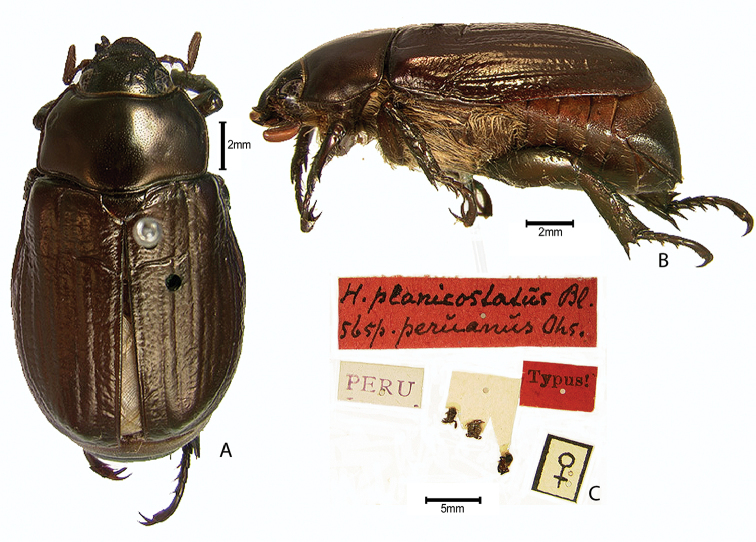
*Homonyx planicostatus peruanus* Ohaus (valid name *H. peruanus*) syntype female from ZMHB. **A** Dorsal habitus **B** Lateral habitus **C** Specimen labels and mouthparts.

1 ♀ syntype specimen of *Homonyx planicostatus peruanus* Ohaus at ZMHB (Fig. 30) (probably the ♀ holotype referred to by Soula [2010a]).

###### Remarks.

Krajcik (2012, 2013) considered *H. peruanus* to be a subspecies of *H. elongatus*.

##### Homonyx planicostatus

Taxon classificationAnimaliaColeopteraScarabaeidae

Blanchard, 1851

Homonyx planicostatus Blanchard, 1851: 214 [original combination].

###### Distribution.

ARGENTINA: Mendoza, Tucumán (Ohaus 1913, 1934b, Blackwelder 1944, Gutiérrez 1952, Machatschke 1972). BOLIVIA (Blanchard 1851, Burmeister 1855, Ohaus 1913, 1934b, Blackwelder 1944, Gutiérrez 1952, Machatschke 1972, Krajcik 2008, Soula 2010a).

###### Types.

**Figure 31.**
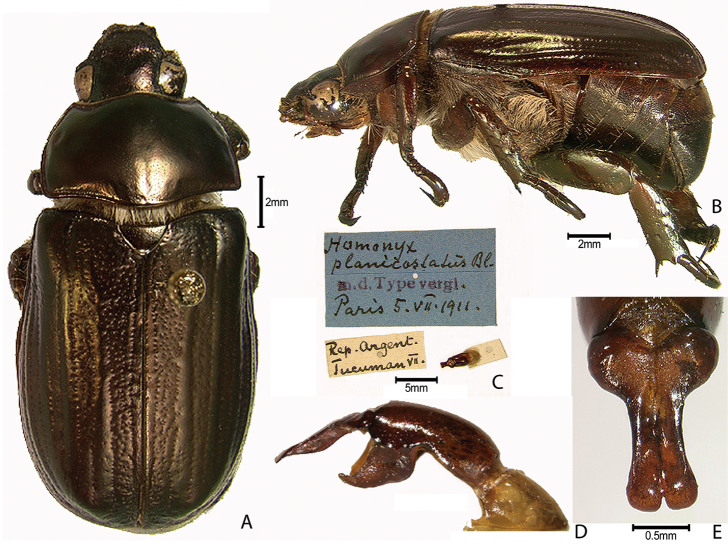
*Homonyx planicostatus* Blanchard (male specimen compared [by Ohaus] with Blanchard’s type from MNHN). **A** Dorsal habitus **B** Lateral habitus **C** Specimen labels and male genitalia **D** Male genitalia, lateral view **E** Male parameres, caudal view.

1 ♀ syntype at MNHN (Soula 2010a). An exemplar specimen identified by Ohaus and compared with Blanchard’s type specimen is figured (Fig. 31).

###### Remarks.

CCECL contains a *H. planicostatus* specimen labeled as a male ♂ alloréférent with the following data: “Vaccaguzman [arrow] Camiri coll. – SOULA [obverse] 1615m//Alloréférent ♂ de *Homonyx planicostatus* Bl. M. SOULA det 19 (47030995). Genitalia card-mounted underneath specimen. Box 4618689 SOULA.

##### Homonyx santiagensis

Taxon classificationAnimaliaColeopteraScarabaeidae

Ohaus, 1913

Homonyx chalceus santiagensis Ohaus, 1913: 494 [original combination].
Homonyx santiagensis Ohaus [new species status by Soula 2010a: 12].

###### Distribution.

ARGENTINA: Córdoba, Jujuy, Santiago del Estero (Ohaus 1913, 1934b, Blackwelder 1944, Machatschke 1972, Krajcik 2008, Soula 2010a).

###### Types.

**Figure 32.**
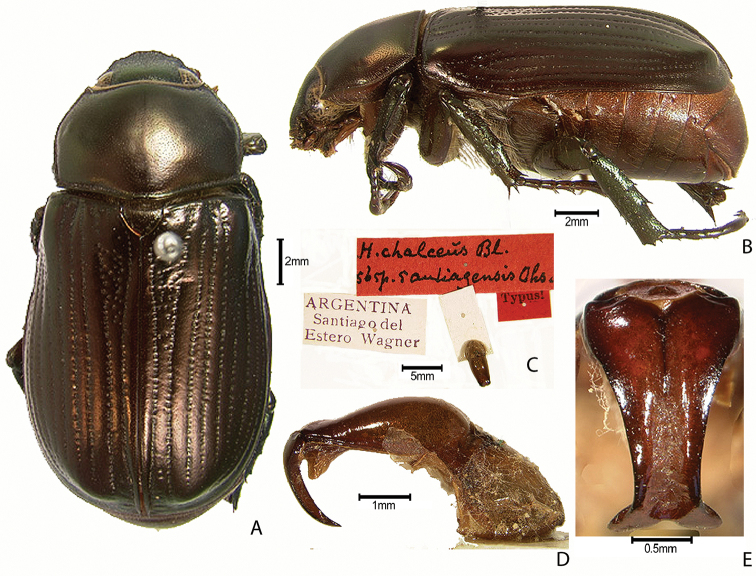
*Homonyx chalceus santiagensis* Ohaus (valid name *H. santiagensis*) type male (see “*Type specimens and lectotype designation*” in Methods) from ZMHB. **A** Dorsal habitus **B** Lateral habitus **C** Specimens labels and male genitalia **D** Male genitalia, lateral view **E** Parameres, caudal view.

1 ♂ lectotype and 1 paralectotype at ZMHB (Fig. 32) (Soula 2010a).

###### Remarks.

Krajcik (2012, 2013) considered *H. santiagensis* to be a subspecies of *H. chalceus*.

##### Homonyx uruguayanus

Taxon classificationAnimaliaColeopteraScarabaeidae

Ohaus, 1913

Homonyx chalceus uruguayanus Ohaus, 1913: 494 [original combination].
Homonyx uruguayensis Ohaus [new species status and incorrect subsequent spelling by Soula 2010a: 13].

###### Distribution.

ARGENTINA: Córdoba, Entre Ríos (Soula 2010a). URUGUAY (Ohaus 1913, 1934b, Blackwelder 1944, Machatschke 1972, Krajcik 2008, Soula 2010a).

###### Types.

**Figure 33.**
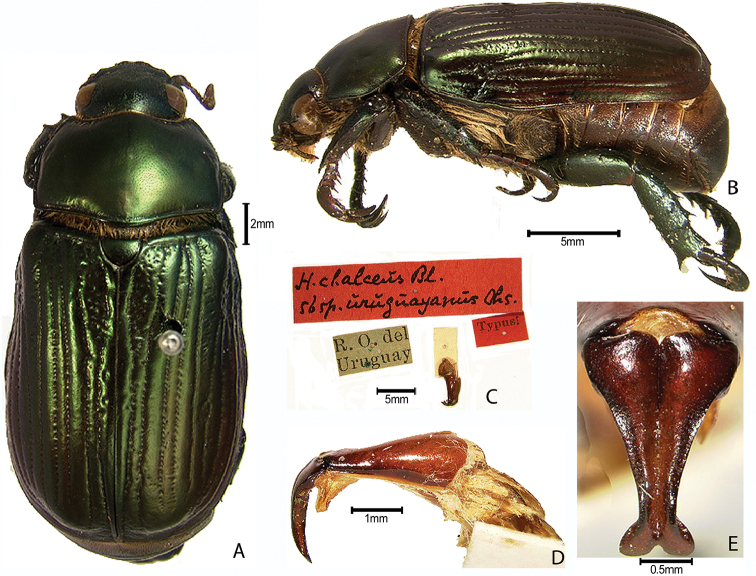
*Homonyx chalceus uruguayanus* Ohaus (valid name *H. uruguayanus*) syntype male from ZMHB. **A** Dorsal habitus **B** Lateral habitus **C** Specimens labels and male genitalia **D** Male genitalia, lateral view **E** Parameres, caudal view.

1 ♂ syntype of *Homonyx chalceus uruguayanus* at ZMHB (called a holotype by Soula 2010a) (Fig. 33).

###### Remarks.

Krajcik (2012, 2013) considered *H. uruguayensis* to be a subspecies of *H. chalceus*.

### Unavailable names in Homonyx(application of ICZN Article 16.4.2)

We consider the following names proposed by Soula in Homonyx as **unavailable** per ICZN Article 16.4.2 which states that fixation of holotype specimens for new names must be accompanied by the following information, “where the holotype or syntypes are extant specimens, by a statement of intent that they will be (or are) deposited in a collection and a statement indicating the name and location of that collection”. The names below were proposed by Soula (2010, 2011), but the descriptions did not state the intent to deposit the holotype specimens in a collection. By applying ICZN Article 16.4.2 herein, the following names are **unavailable**: *Homonyx digennaroi* Soula 2010, *Homonyx lecourti* Soula 2010, *Homonyx mulliei* Soula 2010, *Homonyx simoensi* Soula 2010, *Homonyx wagneri* Soula 2010, and *Homonyx zovii* Demez and Soula 2011. Below we report the complete taxonomic history of these names and the data from their invalid type specimens that are deposited at CCECL.

#### Homonyx digennaroi

Taxon classificationAnimaliaColeopteraScarabaeidae

Soula, 2010 Unavailable, invalid name

Homonyx digennaroi Soula, 2010a: 19, 21-22 [original combination, **unavailable, invalid name**].

##### Distribution.

BOLIVIA (Soula 2010a).

##### Types.

The following invalid type specimens are deposited at CCECL. 1 invalid ♂ holotype: “Rte de Camiri à Sta Cruz Bol. coll. – SOULA//Holotype 2010 *Homonyx digennaroi* S. Soula (47031008). Genitalia card-mounted underneath the male holotype. Box 4618689 SOULA.

#### Homonyx lecourti

Taxon classificationAnimaliaColeopteraScarabaeidae

Soula, 2010 Unavailable, invalid name

Homonyx lecourti Soula, 2010a: 19, 20 [original combination, **unavailable, invalid name**].

##### Distribution.

BOLIVIA: La Paz (Soula 2010a).

##### Types.

The following invalid type specimens are deposited at CCECL. 1 invalid ♂ holotype and 1 invalid ♀ allotype: “Yocumo 920 m 26/10/2000 M. SOULA det 19//Holotype 2010 *Homonyx lecourti* S. Soula (47031006); “Yocumo 920 m 26/X/2000 M. SOULA det 19//Allotype 2010 *Homonyx lecourti* S. Soula (47031007). Genitalia card-mounted underneath the male holotype. Box 4618689 SOULA.

#### Homonyx mulliei

Taxon classificationAnimaliaColeopteraScarabaeidae

Soula, 2010 Unavailable, invalid name

Homonyx mulliei Soula, 2010a: 23, 24 [original combination, **unavailable, invalid name**].

##### Distribution.

BOLIVIA (Soula 2010a).

##### Types.

The following invalid type specimen is deposited at CCECL. 1 invalid ♂ holotype: “Camiri à Sta Cruz coll. – Soula//Holotype 2010 *Homonyx mulliei* S. Soula (47031005). Genitalia card-mounted underneath the male holotype. Box 4618689 SOULA.

#### Homonyx simoensi

Taxon classificationAnimaliaColeopteraScarabaeidae

Soula, 2010 Unavailable, invalid name

Homonyx simoensi Soula, 2010a: 22, 23 [original combination, **unavailable, invalid name**].

##### Distribution.

BOLIVIA (Soula 2010a).

##### Types.

The following invalid type specimen is deposited at CCECL. 1 invalid ♂ holotype: “Camiri à Sta Cruz coll. – SOULA//Holotype 2010 *Homonyx simoensi* S. Soula (47031009). Genitalia card-mounted underneath the male holotype. Box 4618689 SOULA.

#### Homonyx wagneri

Taxon classificationAnimaliaColeopteraScarabaeidae

Soula, 2010 Unavailable, invalid name

Homonyx wagneri Soula, 2010a: 23, 25 [original combination, **unavailable, invalid name**].

##### Distribution.

ARGENTINA: Salta (Soula 2010a).

##### Types.

The following invalid type specimen is deposited at CCECL. 1 invalid ♂ holotype: “Salta Argentine XI/2006 M. SOULA det 19//Holotype 2010 *Homonyx wagneri* S. Soula (47030997). Genitalia card-mounted underneath the male holotype. Box 4618689 SOULA.

#### Homonyx zovii

Taxon classificationAnimaliaColeopteraScarabaeidae

Demez & Soula, 2011 Unavailable, invalid name

Homonyx zovii Demez & Soula, 2011: 74 [original combination, **unavailable, invalid name**].

##### Distribution.

PERU: San Martín (Soula 2011, Ratcliffe et al. 2015).

##### Types.

The following invalid type specimen is deposited at CCECL. 1 invalid ♂ holotype: “Janjui San Martin IX/2010 M. SOULA det 19//Holotype 2011 *Homonyx zovii* S. 2011 Soula (47030996). Genitalia card-mounted underneath the male holotype. Box 4618689 SOULA.

#### 

Taxon classificationAnimaliaColeopteraScarabaeidae

Ohaus, 1898

Homothermon Ohaus, 1898: 59-60.

##### Type species.

*Homothermon bugre* Ohaus, 1898: 60, original designation by Ohaus 1898: 59–60.

##### Gender.

Neuter.

##### Species.

4 species.

#### Homothermon bugre

Taxon classificationAnimaliaColeopteraScarabaeidae

Ohaus, 1898

Homothermon bugre Ohaus, 1898: 60 [original combination].

##### Distribution.

ARGENTINA: Misiones (Ohaus 1898, 1934b, Machatschke 1972, Krajcik 2008, Soula 2008). BRAZIL: Rio Grande do Sul, Santa Catarina (Ohaus 1898, 1918, 1934b, Machatschke 1972, Soula 2008).

##### Types.

1 ♂ lectotype and paralectotypes at ZMHB (Soula 2008); 1 paralectotype at MNHN (Soula 2010a).

#### Homothermon drumonti

Taxon classificationAnimaliaColeopteraScarabaeidae

Soula, 2008

Homothermon drumonti Soula, 2008: 33 [original combination].

##### Distribution.

BRAZIL: São Paulo (Soula 2008).

##### Types.

The following specimen is deposited at CCECL. 1 ♂ holotype: “Brasil OL. Guillot//Det Dr. Ohaus *Homothermon paulista* Ohaus//Holotype *Homothermon drumonti* S. 2007 Soula” (47031074). Genitalia card-mounted underneath the male holotype. Box 4618691 SOULA.

#### Homothermon praemorsus

Taxon classificationAnimaliaColeopteraScarabaeidae

(Burmeister, 1855)

Odontognathus praemorsus Burmeister, 1855: 521 [original combination].
Homothermon praemorsus (Burmeister) [new combination by Ohaus 1918: 30].
Homothermon paulista Ohaus, 1898 **synonym.**
Homothermon paulista Ohaus, 1898: 61 [original combination].
Homothermon praemorsus (Burmeister) [syn. by Ohaus 1918: 30].

##### Distribution.

BRAZIL: São Paulo (Burmeister 1855, Ohaus 1898, 1918, 1934b, Machatschke 1972, Krajcik 2008, Soula 2008).

##### Types.

**Figure 34.**
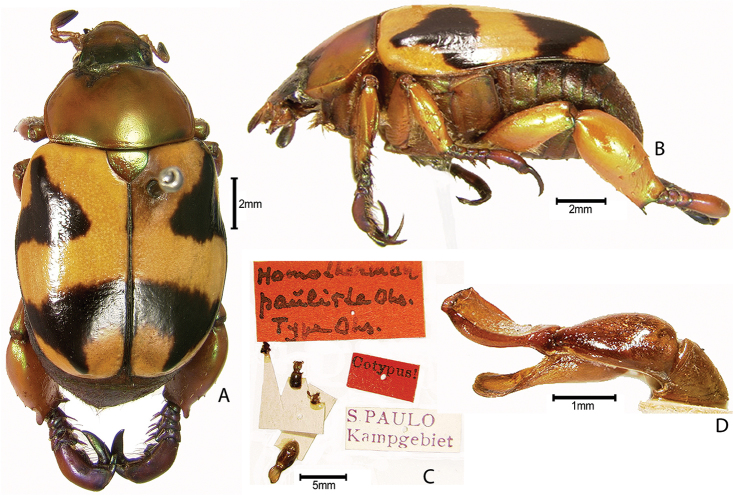
*Homothermon paulista* Ohaus (valid name *H. praemorsus* Burmeister) type male (see “*Type specimens and lectotype designation*” in Methods) from ZMHB. **A** Dorsal habitus **B** Lateral habitus **C** Specimens labels, mouthparts, and male genitalia **D** Male genitalia, lateral view.

1 ♀ syntype of *Odontognathus praemorsus* at ZMHB (Soula 2008). 1 ♂ syntype specimen of *Homothermon paulista* at ZMHB (Fig. 34); 1 syntype specimen of *Homothermon paulista* at SDEI. Soula (2008: 32) stated that he found the holotype at ZMHB, yet he provided an image of a lectotype specimen (see “*Type Specimens and Lectotype Designation*” in Methods).

#### Homothermon serrano

Taxon classificationAnimaliaColeopteraScarabaeidae

Ohaus, 1898

Homothermon serrano Ohaus, 1898: 60 [original combination].

##### Distribution.

ARGENTINA: Misiones (Soula 2008). BRAZIL: Rio Grande do Sul, Santa Catarina (Ohaus 1898, 1918, 1934b, Machatschke 1972, Krajcik 2008, Soula 2008).

##### Types.

**Figure 35.**
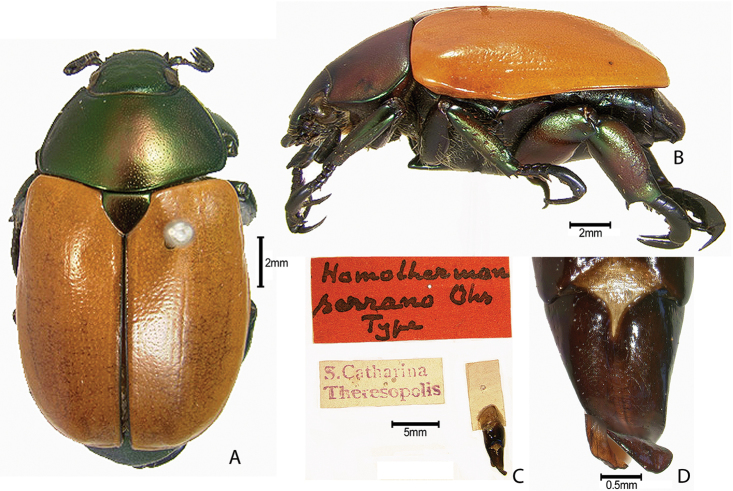
*Homothermon serrano* Ohaus paralectotype male from ZMHB. **A** Dorsal habitus **B** Lateral habitus **C** Specimens labels and male genitalia **D** Male parameres, caudal view.

1 ♂ lectotype and 2 paralectotypes at ZMHB (Fig. 35); 1 paralectotype at NHMB (Soula 2008).

#### HOPLOPELIDNOTA

Taxon classificationAnimaliaColeopteraScarabaeidae

F. Bates, 1904

Hoplopelidnota Bates, 1904: 253, 274–275.

##### Type species.

*Hoplopelidnota candezei* F. Bates, 1904: 274–275, by monotypy.

##### Gender.

Feminine.

##### Species.

1 species.

#### Hoplopelidnota metallica

Taxon classificationAnimaliaColeopteraScarabaeidae

(Laporte, 1840)

Pelidnota metallica Laporte, 1840: 122 [original combination].
Hoplopelidnota candezei F. Bates [syn. by Machatschke 1972: 12].
Hoplopelidnota metallica (Laporte) [revised species status by Soula 2008: 17].
Hoplopelidnota armata Ohaus, 1912 **synonym.**
Hoplopelidnota armata Ohaus, 1912: 309 [original combination; sometimes erroneously referred to as *H. armata* F. Bates].
Hoplopelidnota metallica (Laporte) [syn. by Moore and Jameson 2013: 381].
Hoplopelidnota candezei F. Bates, 1904 **synonym.**
Hoplopelidnota candezei F. Bates, 1904: 274–275 [original combination].
Hoplopelidnota metallica (Laporte) [syn. by Soula 2008: 17].

##### Distribution.

BRAZIL: Territorio de Amapa (Serra Navia). FRENCH GUIANA: Cayenne (Laporte 1840, F. Bates 1904, Ohaus 1912, 1918, 1934b, Blackwelder 1944, Machatschke 1972, Soula 2008, 2010c). GUYANA: Essequibo River, Moraballi Creek. VENEZUELA: Amazonas (Rio Negro).

##### Types.

**Figure 36.**
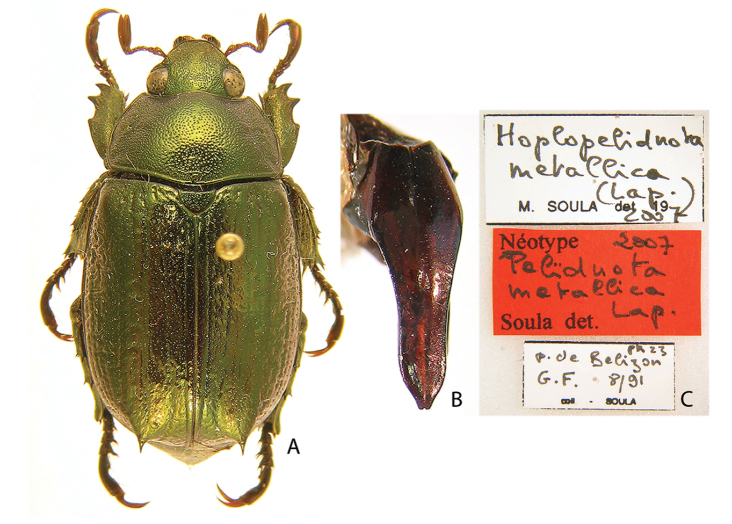
*Hoplopelidnota metallica* (Laporte) invalid neotype male from CCECL. **A** Dorsal habitus **B** Male parameres, caudal view **C** Specimen labels.

The following specimen is deposited at CCECL. 1 invalid ♂ neotype (Fig. 36): “pk 23 p. de Belizon G. F. 8/91 coll. – SOULA [obverse] pk 23//Néotype 2007 *Pelidnota metallica* Lap. Soula det.//*Hoplopelidnota metallica* (Lap.) M. SOULA 19 2007” (47031033). Genitalia card-mounted underneath the invalid neotype. Box 4618690 SOULA.

##### Remarks.

**Figure 37.**
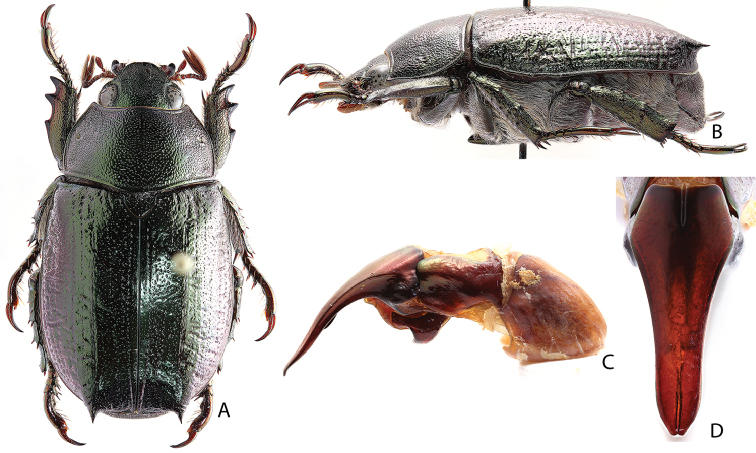
*Hoplopelidnota metallica* (Laporte) male from MSPC. **A** Dorsal habitus **B** Lateral habitus **C** Male genitalia, lateral view **D** Male parameres, caudal view.

The classification of *Hoplopelidnota metallica* (Figs 36, 37) has been tumultuous (Moore and Jameson 2013). Laporte (1840) named *P. metallica*, clearly indicating the unusual form of the elytral apex. Bates’s (1904) description of *Hoplopelidnota candezei* overlooked the conspecific *P. metallica. Hoplopelidnota candezei* was based on a single male specimen that was labeled “Pelidnota armata” by Candèze. The name “H. armata”, however, had not been validly described and therefore was not available. However, Ohaus (1912) provided a description of the female, he used the name “Hoplopelidnota armata” (rather than *H. candezei*). This act made the name *H. armata* an available name and a junior synonym of *H. metallica*. Most recently, Krajcik (2012, 2013) considered *H. candezei* to be a subspecies of *H. metallica*.

Soula (2008: 17–18) attempted to designate a neotype specimen for *Hoplopelidnota metallica*. Soula stated that the neotype is in “Collection Soula”, but Article 75.3.7 (ICZN 1999) requires a statement that the “neotype is, or immediately upon publication has become, the property of a recognized scientific or educational institution, cited by name, that maintains a research collection, with proper facilities for preserving name-bearing types, and that makes them accessible for study”. Because Soula’s collection was private at the time of designation, Soula’s neotype is invalid.

*Hoplopelidnota metallica* is distributed in northern South America. Prior to this work, *H. metallica* was only recorded from French Guiana. In addition to French Guiana, we record the species from Guyana (Moraballi Creek, Essequibo River), Venezuela (Amazonas Dept., Rio Negro) and Brazil (Territorio de Amapa, Serra Navia). The species is rare in collections, and is apparently much more wide spread in northern South America than previous data would indicate. Specimens are recorded from 140 m elevation in March, April, July, August, and November.

#### 

Taxon classificationAnimaliaColeopteraScarabaeidae

F. Bates, 1904

Mecopelidnota F. Bates, 1904: 252, 270–271.

##### Type species.

*Mecopelidnota arrowi* F. Bates, 1904: 271–272, by monotypy.

##### Gender.

Feminine.

##### Species.

8 species.

#### Mecopelidnota arrowi

Taxon classificationAnimaliaColeopteraScarabaeidae

F. Bates, 1904

Mecopelidnota arrowi F. Bates, 1904: 271–272 [original combination].
Pelidnota egregia Frey, 1967 **synonym.**
Pelidnota egregia Frey, 1967: 374–375 [original combination].
Pelidnota (Pelidnota) egregia Frey [new subgeneric combination by Machatschke 1972: 25].
Mecopelidnota arrowi F. Bates [syn. by Soula 2008: 23].

##### Distribution.

ECUADOR: Azuay, Guayas (F. Bates 1904, Ohaus 1908b, 1910c, 1918, 1934b, Blackwelder 1944, Frey 1967, Machatschke 1972, Paucar-Cabrera 2005, Krajcik 2008, Soula 2008). PERU (Ohaus 1934b, Blackwelder 1944, Machatschke 1972, Ratcliffe et al. 2015).

##### Types.

**Figure 38.**
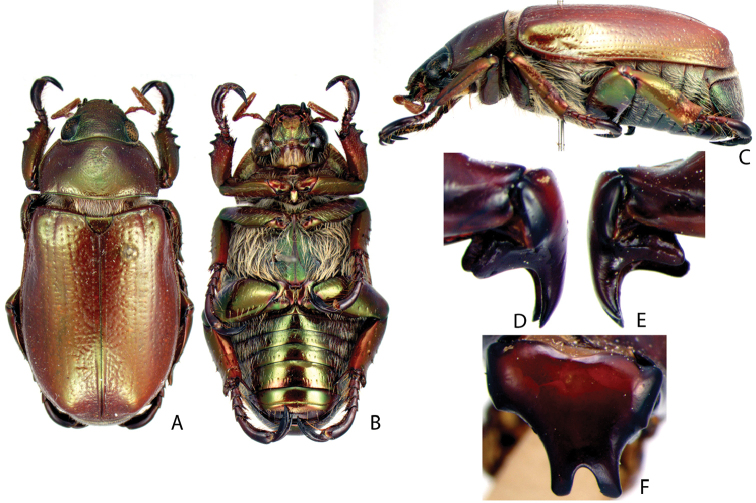
Pelidnota (Pelidnota) egregia Frey (valid name *Mecopelidnota arrowi* F. Bates) paratype male from CMNC. **A** Dorsal habitus **B** Ventral habitus **C** Lateral habitus **D** Male parameres, right lateral view **E** Male parameres, left lateral view **F** Male parameres, caudal view. Images by François Génier.

1 ♂ holotype specimen of *Mecopelidnota arrowi* at BMNH (Soula 2008). 2 paratype specimens of *Pelidnota egregia* Frey at CMNC (Fig. 38).

#### Mecopelidnota cylindrica

Taxon classificationAnimaliaColeopteraScarabaeidae

(Waterhouse, 1876)

Pelidnota cylindrica Waterhouse, 1876: 24 [original combination].
Mecopelidnota cylindrica (Waterhouse) [new combination by F. Bates 1904: 271].

##### Distribution.

ECUADOR: Guayas (Paucar-Cabrera 2005).

##### Types.

1 ♂ lectotype at BMNH (Soula 2008, BHG pers. obs. Aug. 2016).

##### Remarks.

*Mecopelidnota cylindrica* (Waterhouse) has been reported from Guatemala without further details (Waterhouse 1876, H. W. Bates 1888, Ohaus 1918, 1934b, Blackwelder 1944, Machatschke 1972, Krajcik 2008, Soula 2008). This is the only Central American record for the genus *Mecopelidnota*. To our knowledge *M. cylindrica* (Waterhouse) has not been collected in Guatemala since the original Waterhouse specimen was described. Research on the scarabs of Guatemala considered the reference of the species in Guatemala to be possibly erroneous (Monzón 1996). We consider *M. cylindrica* (Waterhouse) to be a South American species.

#### Mecopelidnota dewynteri

Taxon classificationAnimaliaColeopteraScarabaeidae

Soula, 2008

Mecopelidnota dewynteri Soula, 2008: 26 [original combination].

##### Distribution.

PERU: Cajamarca (Soula 2008, Ratcliffe et al. 2015).

##### Types.

The following specimens are deposited at CCECL. 1 ♂ holotype, 1 ♀ allotype, 2 ♂ paratypes, 7 ♀ paratypes: “Limon, Cajamarca N-Pérou, 1800m XII/2000//Holotype *Mecopelidnota dewinteri* (sic) S. 2007 Soula” (47031098); “Limon, Cajamarca N-Pérou, 1800m XII/2000//Allotype *Mecopelidnota dewynteri* S. 2007 Soula” (47031099); Nine paratypes with identical label data: “Limon, Cajamarca N-Pérou, 1800m XII/2000//Paratype *Mecopelidnota dewynteri* S. 2007 Soula” (47031100 to 47031106). Genitalia card-mounted underneath the male holotype and the male paratype. Box 4618692 SOULA.

#### Mecopelidnota gerardi

Taxon classificationAnimaliaColeopteraScarabaeidae

Soula, 2008

Mecopelidnota gerardi Soula, 2008: 25–26 [original combination].

##### Distribution.

ECUADOR: Azuay (Soula 2008).

##### Types.

Soula (2008) stated that the holotype male, allotype female, and a paratype series were deposited in his personal collection. We did not find these specimens at CCECL. Additional paratypes were deposited at PAPC and Gérard Duranton’s collection (Soula 2008).

#### Mecopelidnota marxi

Taxon classificationAnimaliaColeopteraScarabaeidae

Soula, 2008

Mecopelidnota marxi Soula, 2008: 25 [original combination].

##### Distribution.

ECUADOR: Loja (Soula 2008). PERU: Lambayeque, Piura (Soula 2008, Ratcliffe et al. 2015).

##### Types.

The following specimens are deposited at CCECL. 1 ♂ holotype, 1 ♀ allotype, 5 ♂ paratypes, 13 ♀ paratypes: “Abra Porculla, Dt. Piura N-W Pérou; 1800m II/2007//Holotype *Mecopelidnota marxi* S. 2007 Soula” (47031109); “Abra Porculla, Dt. Piura N-W Pérou; 1800m II/2007//Allotype *Mecopelidnota marxi* S. 2007 Soula” (47031110); Eleven paratypes with identical label data: “Abra Porculla, Dt. Piura N-W Pérou; 1800m II/2007//Paratype *Mecopelidnota marxi* S. 2007 Soula” (47031111 to 47031120, exch61); “PERU Sullana Hda. Mallares 9. III.58 W. MARKL//Paratype *Mecopelidnota marxi* S. 2007 Soula” (47031121); “PERU Sullana Hda. Mallares 28. II.57 W. MARKL//Paratype *Mecopelidnota marxi* S. 2007 Soula” (47031122); Two paratypes with label data “Catamayo Loja (Eq.) coll. – SOULA [obverse] 7/III/98 (1300m)//Paratype *Mecopelidnota marxi* S. 2007 Soula” (47031123). Genitalia card-mounted underneath the male holotype and three female paratypes. Box 4618692 SOULA.

#### Mecopelidnota mezai

Taxon classificationAnimaliaColeopteraScarabaeidae

Soula, 2008

Mecopelidnota mezai Soula, 2008: 28 [original combination].

##### Distribution.

PERU: Lambayeque, Piura (Soula 2008, Ratcliffe et al. 2015).

##### Types.

The following specimens are deposited at CCECL. 1 ♂ holotype, 1 ♀ allotype, 9 ♂ paratypes, 12 ♀ paratypes: “Abra Porculla, Dt. Piura N-W Pérou ; 1800m II/2007//Holotype 2007 *Mecopelidnota mezai* S. Soula” (47031076); “Abra Porculla, Dt. Piura N-W Pérou ; 1800m II/2007//Allotype 2007 *Mecopelidnota mezai* S. Soula” (47031077); Six paratypes with identical label data: “Abra Porculla, Dt. Piura N-W Pérou ; 1800m II/2007//Paratype 2007 *Mecopelidnota mezai* S. Soula” (47031078 to 47031083); “Abra Porculla Piura - Pérou coll. – SOULA [obverse] XI/2000 ~1000m//Paratype 2007 *Mecopelidnota mezai* S. Soula” (47031084); Eight paratypes with identical label data: “Penachi, 1800m Dto. Lambayeque N.W. Pérou, III/2007//Paratype 2007 *Mecopelidnota mezai* S. Soula” (47031085 to 47031091, exch60); “Penachi D^t^ Lambayeque Pérou M. SOULA det 19 [obverse] 2000 III/2007//Paratype 2007 *Mecopelidnota mezai* S. Soula” (47031093); “Huasmaca Piura 1700 m M. SOULA det 19 [obverse] N-W-Pérou 5-6/2006//Paratype *Mecopelidnota mezai* S. 2007 Soula” (47031094). Two paratypes with identical label data “Penachi, 1800m Dto. Lambayeque N. W. Pérou, III/2007//Paratype 2007 *Mecopelidnota mezai* S. Soula” (47031095 and 47031096). Genitalia card-mounted underneath the male holotype, the female allotype, one female paratype and four male paratypes. Box 4618691 SOULA.

#### Mecopelidnota obscura

Taxon classificationAnimaliaColeopteraScarabaeidae

(Taschenberg, 1870)

Pelidnota obscura Taschenberg, 1870: 184–185 [original combination].
Mecopelidnota obscura (Taschenberg) [new combination by Ohaus 1918: 22].

##### Distribution.

COLOMBIA (Taschenberg 1870, Ohaus 1918, 1934b, Blackwelder 1944, Machatschke 1972, Restrepo et al. 2003, Krajcik 2008, Soula 2008). ECUADOR: Guayas, Loja (Paucar-Cabrera 2005).

#### Mecopelidnota witti

Taxon classificationAnimaliaColeopteraScarabaeidae

Ohaus, 1913

Mecopelidnota witti Ohaus, 1913: 497–498 [original combination].

##### Distribution.

ECUADOR: Azuay, Loja (Ohaus 1913, 1918, 1934b, Blackwelder 1944, Machatschke 1972, Paucar-Cabrera 2005; Krajcik 2008; Soula 2008).

##### Types.

1 ♂ lectotype and 2 paralectotypes at ZMHB (Soula 2008).

### Invalid, unavailable names in *Mecopelidnota*

#### Mecopelidnota willersi

Taxon classificationAnimaliaColeopteraScarabaeidae

 in litt.; Unavailable, invalid name

##### Types.

The following specimens are deposited at CCECL. 1 ♂ invalid holotype, 1 ♀ invalid allotype, 3 ♂ invalid paratypes, 2 ♀ invalid paratypes: “Oña, Equat. 2500 m II/2001 M. SOULA det 19//Holotype *Mecopelidnota willersi* S. 2007 Soula//Invalid Holotype det. MR Moore ‘15” (47031128); “Oña, Equat. 2500 m II/2001 M. SOULA det 19//Allotype *Mecopelidnota willersi* S. 2007 Soula//Invalid Allotype det. MR Moore ‘15” (47031129); Two invalid paratypes with identical label data: “Equateur M. SOULA det 19//Paratype *Mecopelidnota willersi* S. 2007 Soula//Invalid Paratype det. MR Moore ‘15” (47031130 and 47031131); Three invalid paratyes with identical label data “Oña - Equ. II/2001 M. SOULA det 19 [obverse] 2500 m//Paratype *Mecopelidnota willersi* S. 2007 Soula//Invalid Paratype det. MR Moore ‘15” (47031132). Genitalia card-mounted underneath the invalid male holotype and the two invalid male paratypes. Box 4618692 SOULA.

##### Remarks.

The name *Mecopelidnota willersi* Soula has never been associated with a species description or holotype designation in the literature. These type specimens of *Mecopelidnota willersi* are thus invalid.

#### Mecopelidnota bondili

Taxon classificationAnimaliaColeopteraScarabaeidae

 in litt.; Unavailable, invalid name

##### Types.

The following specimens are deposited at CCECL. 1 invalid ♂ paratype: “Oña, Equateur 2500m II/2001 M. SOULA det 19//Paratype *Mecopelidnota bondili* Sou. 2007 Soula” (47031097)// Invalid paratype det. M. R. Moore ‘15”. Genitalia card-mounted (only apex of aedeagus) underneath the invalid male paratype. Box 4618691 SOULA.

##### Remarks.

The name *Mecopelidnota bondili* Soula has never been associated with a species description or holotype designation in the literature. This type specimen of *Mecopelidnota bondili* is thus invalid.

#### 

Taxon classificationAnimaliaColeopteraScarabaeidae

Ohaus, 1905

Mesomerodon Ohaus, 1905: 319.

##### Type species.

*Mesomerodon spinipenne* Ohaus, 1905: 320–321, by monotypy.

##### Gender.

Neuter.

##### Species.

2 species.

#### Mesomerodon gilletti

Taxon classificationAnimaliaColeopteraScarabaeidae

Soula, 2008

Mesomerodon gilletti Soula, 2008: 21 [original combination].

##### Distribution.

ECUADOR: Napo (Soula 2008).

##### Types.

**Figure 39.**
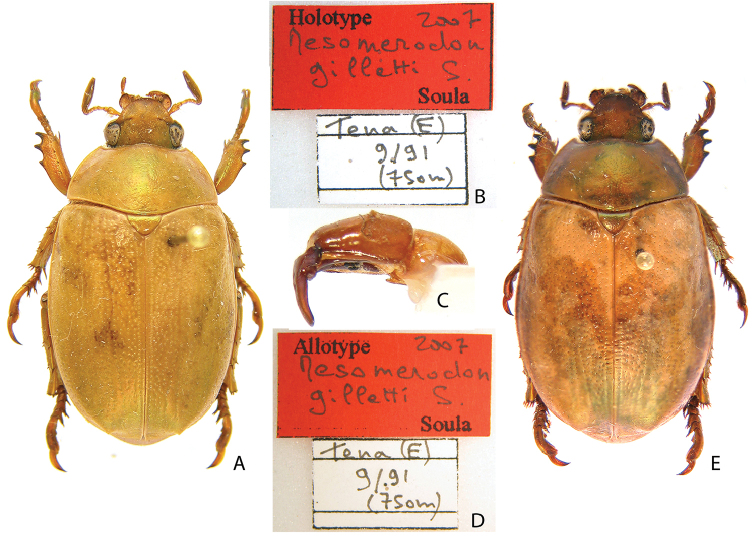
*Mesomerodon gilletti* Soula holotype male and allotype female from CCECL. **A** Dorsal habitus holotype **B** Specimen labels, holotype **C** Male genitalia, lateral view **D** Specimen labels, allotype **E** Dorsal habitus, allotype.

**Figure 40.**
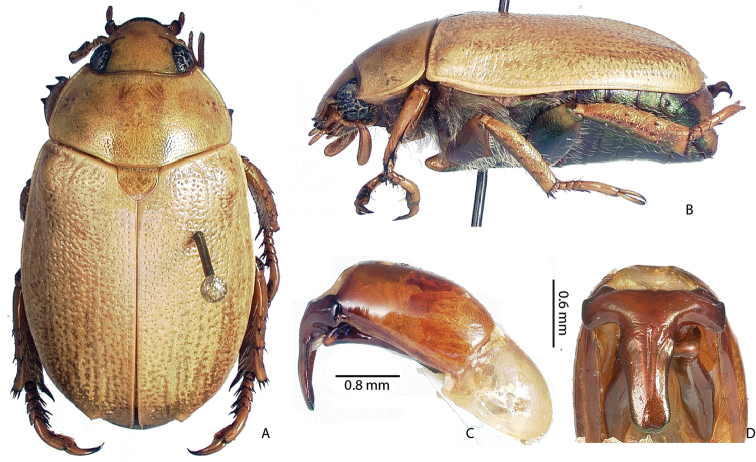
*Mesomerodon gilletti* Soula male from FSCA. **A** Dorsal habitus **B** Lateral habitus **C** Male genitalia, lateral view **D** Parameres, caudal view.

The following specimens are deposited at CCECL (Fig. 39). 1 ♂ holotype, 1 ♀ allotype, 4 ♂ paratypes, 4 ♀ paratypes: “Tena (E), 9/91, (750m)//Holotype, 2007, *Mesomerodon gilletti* S., Soula”; “Tena (E), 9/91, (750m)//Allotype, 2007, *Mesomerodon gilletti* S., Soula”; Three paratypes with identical label data: “Tena (E), 9/91, (750m)//Paratype, 2007, *Mesomerodon gilletti* S., Soula”; four paratypes with identical label data: “Misahuali (E.), 5/91” or “Misahuali (Eq.), 5/91//Paratype, 2007, *Mesomerodon gilletti* S., Soula”; “EQUATEUR: Prov. NAPO, MISAHUALLI ile ANACONDA, Alt. 350 m.; 17-22.9.1990, Leg. Joss//Paratype, 2007, *Mesomerodon gilletti* S., Soula”. Genitalia card-mounted underneath the male holotype and the four male paratypes. An exemplar specimen is figured (Fig. 40) showing the lateral view.

#### Mesomerodon spinipenne

Taxon classificationAnimaliaColeopteraScarabaeidae

Ohaus, 1905

Mesomerodon spinipenne Ohaus, 1905: 320–321 [original combination].

##### Distribution.

BOLIVIA: Cochabamba (WBWC), Santa Cruz (Blackwelder 1944, Ohaus 1918, 1934b, 1952, Gutiérrez 1951, Machatschke 1972). BRAZIL (Blackwelder 1944, Ohaus 1905, 1934b, 1952, Machatschke 1972, Krajcik 2008, Soula 2008). ECUADOR: Napo, Pastaza, Pichincha, Zamora Chinchipe (Blackwelder 1944, Ohaus 1918, 1934b, 1952, Machatschke 1972, Paucar-Cabrera 2005). PERU: Huánuco, Junín, Pasco (Blackwelder 1944, Ohaus 1905, 1918, 1934b, 1952, Machatschke 1972, Soula 2008, Ratcliffe et al. 2015).

##### Types.

1 ♂ lectotype and 2 paralaectotypes at ZMHB (Soula 2008).

#### 

Taxon classificationAnimaliaColeopteraScarabaeidae

Gutiérrez, 1951

Oogenius (Microogenius) Gutiérrez 1951: 107.
Lasiocala Blanchard [syn. by Martínez 1974: 306].
Minilasiocala Soula [syn. by Soula 2006: 139].
Microogenius Gutiérrez [new genus status by Moore and Jameson 2013: 380–381].
Minilasiocala Soula, 2006 **synonym.**
Minilasiocala Soula, 2006: 116, 139. [Type species. *Lasiocala arrowi* Ohaus, 1910b, by original designation].
Microogenius Gutiérrez [syn. by Moore and Jameson 2013: 380–381].

##### Type species.

Oogenius (Microogenius) martinezi Gutiérrez 1951: 107–109, by original designation.

##### Gender.

Masculine.

##### Species.

4 species.

##### Remarks.

Krajcik (2012, 2013) considered *Microogenius* to be a synonym of *Oogenius* and *Minilasiocala* a synonym of *Lasiocala*.

#### Microogenius arrowi

Taxon classificationAnimaliaColeopteraScarabaeidae

(Ohaus, 1910)

Lasiocala arrowi Ohaus, 1910b: 221–222 [original combination].
Minilasiocala arrowi (Ohaus) [new combination by Soula 2006: 139, 140–141].
Microogenius arrowi (Ohaus) [new combination by Moore and Jameson 2013: 380–381].

##### Distribution.

BOLIVIA: La Paz (Ohaus 1910b, 1918, 1934b, Machatschke 1972, Soula 2006, Krajcik 2008).

##### Types.

**Figure 41.**
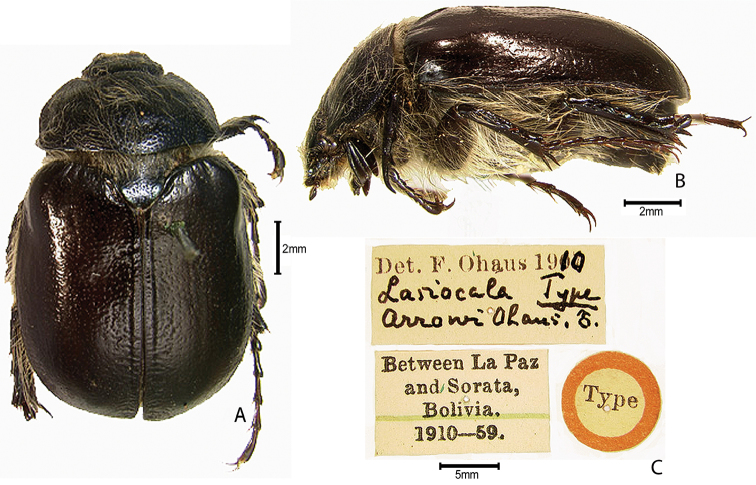
*Lasiocala arrowi* Ohaus (valid name *Microogenius arrowi* [Ohaus]) paralectotype male from BMNH. **A** Dorsal habitus **B** Lateral habitus **C** Specimen labels.

1 ♂ lectotype and 2 paralectotypes of *Microogenius arrowi* at ZMHB; 1 ♂ paralectotype at BMNH (Fig. 41). Soula (2006) indicated that additional paralectotypes are deposited at BMNH, and we located a total of seven paralectotypes at BMNH (pers. obs. BHG Aug. 2016).

##### Remarks.

Krajcik (2012, 2013) considered *M. arrowi* to be a member of the genus *Lasiocala*. The specific epithet “arrowi” is also used in the closely related genera *Lasiocala* and *Oogenius*. Care should be taken in associating these epithets and genera.

#### Microogenius gutierrezi

Taxon classificationAnimaliaColeopteraScarabaeidae

Martínez, 1953

Oogenius (Microogenius) gutierrezi Martínez, 1953: 81–86 [original combination].
Lasiocala gutierrezi (Martínez) [new combination by Martínez 1974: 306].
Oogenius (Microogenius) gutierrezi Martínez [revised combination and revised subgeneric combination by Mondaca 2005: 19].
Minilasiocala gutierrezi (Martínez) [new combination by Soula 2006: 142].
Microogenius gutierrezi (Martínez) [new combination by Moore and Jameson 2013: 380–381].

##### Distribution.

BOLIVIA: Cochabamba (Martínez 1953, 1974, Machatschke 1972, Mondaca 2005, Soula 2006, Krajcik 2008).

##### Types.

**Figure 42.**
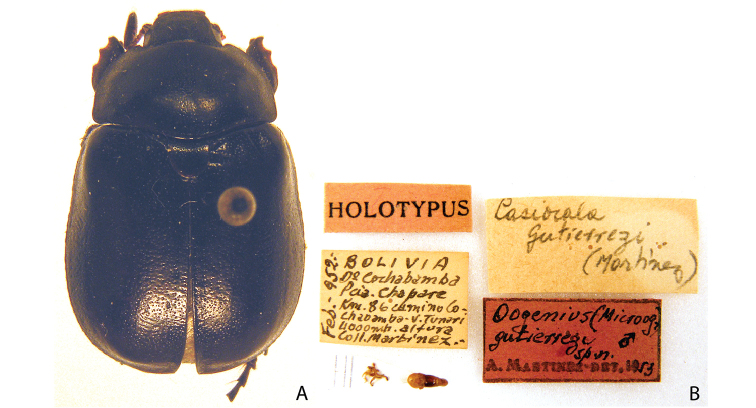
Oogenius (Microogenius) gutierrezi Martínez (valid name *Microogenius gutierrezi* Martínez) holotype male from MACN. **A** Dorsal habitus **B** Specimen labels and male genitalia.

Holotype ♂ of Oogenius (Microogenius) gutierrezi Martínez at MACN (Fig. 42).

##### Remarks.

Krajcik (2012, 2013) considered *Microogenius gutierrezi* to be a member of the genus *Oogenius*.

#### Microogenius lanterii

Taxon classificationAnimaliaColeopteraScarabaeidae

(Soula, 2006)

Minilasiocala lanterii Soula, 2006: 143 [original combination].
Microogenius lanterii (Soula) [new combination by Moore and Jameson 2013: 380–381].

##### Distribution.

ARGENTINA: Jujuy (Soula 2006).

##### Types.

Soula (2006) stated that the holotype was from his personal collection (“Un Mále argentin de ma collection représente”). He purposefully omitted the collector’s names (S & J Peck) from the published label data, thus allowing him to retain the unique, type specimen in his collection undetected. Based on the catalog at CMNC, this specimen unequivocally belongs at CMNC. It was returned from CCECL to its original collection at CMNC and has the following label data: 1 ♂ holotype: “ARG: Jujuy Prov, AbraPampa, 3500m 22-25. XII.87, S&JPeck sandy puna grassland carrion trap”. Genitalia card-mounted underneath the male holotype.

##### Remarks.

Krajcik (2012, 2013) considered *M. lanterii* to be a member of the genus *Lasiocala*.

#### Microogenius martinezi

Taxon classificationAnimaliaColeopteraScarabaeidae

Gutiérrez, 1951

Oogenius (Microogenius) martinezi Gutiérrez, 1951: 107 [original combination].
Lasiocala martinezi (Gutiérrez) [new combination by Martínez 1974: 306].
Oogenius (Microogenius) martinezi Gutiérrez [revised combination and revised subgeneric combination by Mondaca 2005:19].
Minilasiocala martinezi (Gutiérrez) [new combination by Soula 2006: 141–142].
Microogenius martinezi (Gutiérrez) [new combination by Moore and Jameson 2013: 380–381].

##### Distribution.

BOLIVIA: Cochabamba (Gutiérrez 1951, Martínez 1953, 1974, Machatschke 1972, Mondaca 2005, Soula 2006, Krajcik 2008).

##### Types.

**Figure 43.**
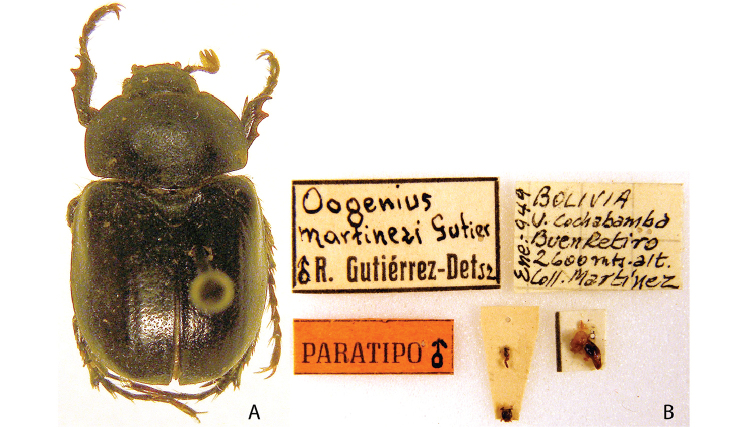
Oogenius (Microogenius) martinezi Gutiérrez (valid name *Microogenius martinezi* [Gutiérrez]) paratype male from UCCC. **A** Dorsal habitus **B** Specimen labels, mouthparts, and male genitalia.

Holotype specimen of O. (M.) martinezi at MACN; 1 ♂ paratype specimen of O. (M.) martinezi at UCCC (Fig. 43).

##### Remarks.

Krajcik (2012, 2013) considered *Microogenius martinezi* to be a member of the genus *Oogenius*.

#### 

Taxon classificationAnimaliaColeopteraScarabaeidae

Martínez, 1953

Neogutierrezia Martínez, 1953: 2.

##### Type species.

*Neogutierrezia mirabilis* Martínez, 1953: 2, by original designation.

##### Gender.

Feminine.

##### Species.

10 species.

#### Neogutierrezia affinis

Taxon classificationAnimaliaColeopteraScarabaeidae

Martínez, 1973

Neogutierrezia mirabilis affinis Martínez, 1973: 35 [original combination].
Neogutierrezia affinis Martínez [new species status by Ocampo et al. 2010: 90].

##### Distribution.

ARGENTINA: Río Negro (Martínez 1973, 1975a, Evans 2003, Evans and Smith 2009, Ocampo et al. 2010).

##### Types.

1 ♂ holotype at MACN (Ocampo et al. 2010); 1 ♂ paratype at IFML (Ocampo et al. 2010); 9 ♂ paratypes at CMNC.

#### Neogutierrezia araucana

Taxon classificationAnimaliaColeopteraScarabaeidae

Martínez, 1973

Neogutierrezia araucana Martínez, 1973: 36–41 [original combination].

##### Distribution.

ARGENTINA: Neuquén (Martínez 1973, 1975a, Evans 2003, Evans and Smith 2009, Ocampo et al. 2010).

##### Types.

1 ♂ holotype and 1 ♀ allotype at MACN (Ocampo et al. 2010); 1 ♂ paratype at IFML (Ocampo et al. 2010); 12 ♂ and 1 ♀ paratypes at CMNC.

#### Neogutierrezia bicolor

Taxon classificationAnimaliaColeopteraScarabaeidae

Ocampo & Ruiz-Manzanos, 2010

Neogutierrezia bicolor Ocampo & Ruiz-Manzanos, 2010: 95–98 [original combination].

##### Distribution.

ARGENTINA: Neuquén (Ocampo et al. 2010).

##### Types.

**Figure 44.**
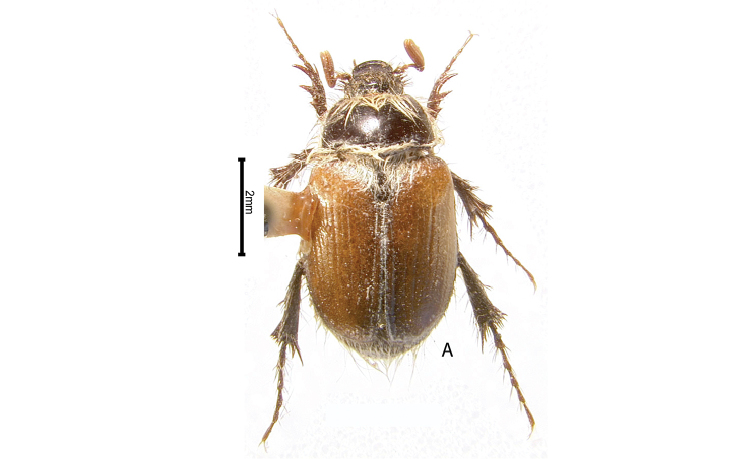
*Neogutierrezia bicolor* Ocampo & Ruiz-Manzanos paratype from IAZA. **A** Dorsal habitus. Photograph courtesy of Federico Ocampo, Pergamino, Argentina.

1 ♂ holotype and 1 ♂ paratype at IAZA (Fig. 44) (Ocampo et al. 2010).

#### Neogutierrezia chelii

Taxon classificationAnimaliaColeopteraScarabaeidae

Ocampo & Ruiz-Manzanos, 2010

Neogutierrezia chelii Ocampo & Ruiz-Manzanos, 2010: 98 [original combination].

##### Distribution.

ARGENTINA: Chubut (Ocampo et al. 2010).

##### Types.

1 ♂ holotype and 3 ♂ paratypes at IAZA (Ocampo et al. 2010).

#### Neogutierrezia galileoi

Taxon classificationAnimaliaColeopteraScarabaeidae

Ocampo & Ruiz-Manzanos, 2010

Neogutierrezia galileoi Ocampo & Ruiz-Manzanos, 2010: 98–99 [original combination].

##### Distribution.

ARGENTINA: Mendoza (Ocampo et al. 2010).

##### Types.

1 ♂ holotype and 1 ♂ paratype at IAZA (Ocampo et al. 2010).

#### Neogutierrezia lagosae

Taxon classificationAnimaliaColeopteraScarabaeidae

Ocampo & Ruiz-Manzanos, 2010

Neogutierrezia lagosae Ocampo & Ruiz-Manzanos, 2010: 99–100 [original combination].

##### Distribution.

ARGENTINA: Mendoza (Ocampo et al. 2010).

##### Types.

1 ♂ holotype and 11 ♂ paratypes at IAZA (Ocampo et al. 2010).

#### Neogutierrezia mirabilis

Taxon classificationAnimaliaColeopteraScarabaeidae

Martínez, 1953

Neogutierrezia mirabilis Martínez, 1953: 2 [original combination].
Neogutierrezia mirabilis mirabilis Martínez [new subspecific status by Martínez 1973: 31].
Neogutierrezia mirablis mirablis Martínez [incorrect spelling by Evans 2003: 220].
Neogutierrezia mirabilis Martínez [revised species status by Ocampo et al. 2010: 100].

##### Distribution.

ARGENTINA: Río Negro (Martínez 1953, 1973, 1975a, Evans 2003, Evans and Smith 2009, Ocampo et al. 2010).

##### Types.

1 ♂ holotype at MACN (Ocampo et al. 2010); 1 ♂ paratype at CMNC.

#### Neogutierrezia payuniensis

Taxon classificationAnimaliaColeopteraScarabaeidae

Ocampo & Ruiz-Manzanos, 2010

Neogutierrezia payuniensis Ocampo & Ruiz-Manzanos, 2010: 101–102 [original combination].

##### Distribution.

ARGENTINA: Mendoza (Ocampo et al. 2010).

##### Types.

1 ♂ holotype at IAZA (Ocampo et al. 2010).

#### Neogutierrezia scutata

Taxon classificationAnimaliaColeopteraScarabaeidae

Ocampo & Ruiz-Manzanos, 2010

Neogutierrezia scutata Ocampo & Ruiz-Manzanos, 2010: 102–103 [original combination].

##### Distribution.

ARGENTINA: Mendoza (Ocampo et al. 2010).

##### Types.

1 ♂ holotype and 13 ♂ paratypes at IAZA (Ocampo et al. 2010); 3 ♂ paratypes at CMNC.

#### Neogutierrezia variabilis

Taxon classificationAnimaliaColeopteraScarabaeidae

Ocampo & Ruiz-Manzanos, 2010

Neogutierrezia variabilis Ocampo & Ruiz-Manzanos, 2010: 103 [original combination].

##### Distribution.

ARGENTINA: Mendoza (Ocampo et al. 2010).

##### Types.

1 ♂ holotype and 13 ♂ paratypes at IAZA (Ocampo et al. 2010).

#### 

Taxon classificationAnimaliaColeopteraScarabaeidae

Solier, 1851

Oogenius Solier, 1851: 97-98.

##### Type species.

*Oogenius virens* Solier, 1851: 98, by monotypy.

##### Gender.

Masculine.

##### Species.

7 species.

#### Oogenius arrowi

Taxon classificationAnimaliaColeopteraScarabaeidae

Gutiérrez, 1949

Oogenius arrowi Gutiérrez, 1949: 27–29 [original combination].
Oogenius (Oogenius) arrowi Gutiérrez [new subgeneric combination by Gutiérrez 1951: 110].
Oogenius arrowi Gutiérrez [revised combination by Soula 2006: 139].

##### Distribution.

ARGENTINA: Mendoza (Gutiérrez 1949, 1951, Martínez 1953, Machatschke 1972, Mondaca 2005, Krajcik 2008, Mondaca 2016).

##### Types.

**Figure 45.**
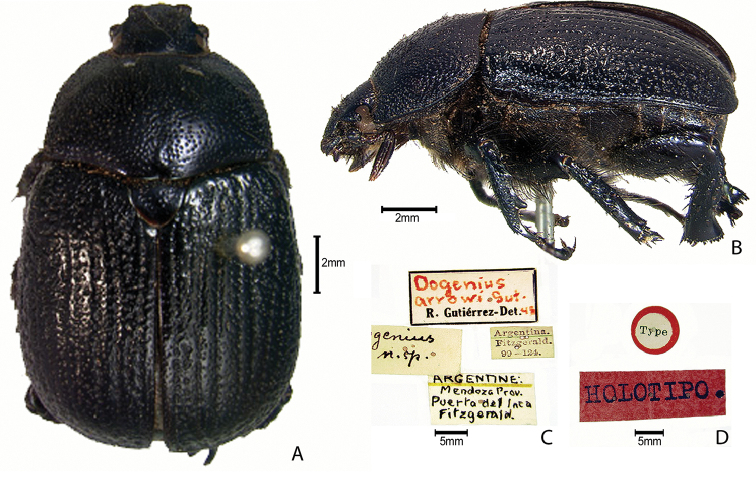
*Oogenius arrowi* Gutiérrez holotype from BMNH. **A** Dorsal habitus **B** Lateral habitus **C** Specimen labels **D** Type labels.

1 ♂ holotype specimen of *Oogenius arrowi* Gutiérrez at BMNH (Fig. 45).

##### Remarks.

The specific epithet “arrowi” is also used in the closely related genera *Lasiocala* and *Microogenius*. Care should be taken when associating epithets in these similar genera.

#### Oogenius castilloi

Taxon classificationAnimaliaColeopteraScarabaeidae

Martínez & Peña, 1990

Oogenius castilloi Martínez & Peña, 1990: 9–11 [original combination].
Oogenius (Oogenius) castilloi Martínez and Peña [new subgeneric combination by Mondaca 2005: 19].
Oogenius castilloi Martínez and Peña [revised combination by Soula 2006: 139].

##### Distribution.

CHILE: Coquimbo (Martínez and Peña-Guzmán 1990, Mondaca 2005, Krajcik 2008, Mondaca 2016).

##### Types.

**Figure 46.**
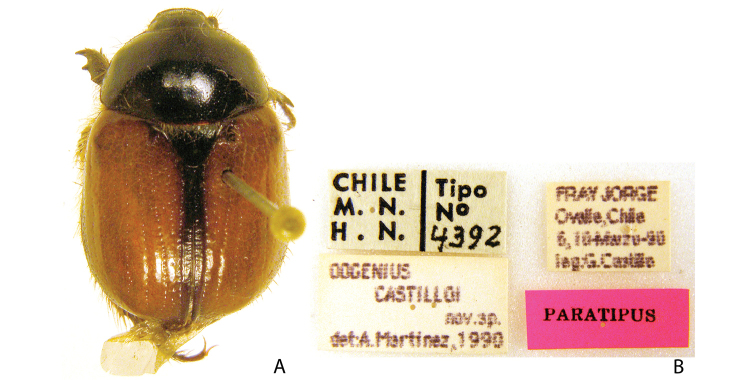
*Oogenius castilloi* Martínez and Peña paratype from MNNC. **A** Dorsal habitus **B** Specimen labels.

Holotype specimen and 20 paratypes of *Oogenius castilloi* at MNNC; 4 paratype specimens at UCCC (Fig. 46); 8 ♂ paratypes in CMNC.

#### Oogenius chilensis

Taxon classificationAnimaliaColeopteraScarabaeidae

Ohaus, 1905

Oogenius chilensis Ohaus, 1905: 326–327 [original combination]
Oogenius (Oogenius) chilensis Ohaus [new subgeneric combination by Gutiérrez 1951: 109].
Oogenius chilensis Ohaus [revised combination by Soula 2006: 139].
Oogenius chilensis barrosi Gutiérrez, 1949 **synonym.**
Oogenius chilensis var. barrosi Gutiérrez, 1949: 27 [original combination, available name per ICZN Article 45.6.4].
Oogenius chilensis forma barrosi Gutiérrez [revised infrasubspecific status by Machatschke 1972: 10].
Oogenius chilensis barrosi Gutiérrez [new subspecific status by Mondaca 2005: 18].
Oogenius chilensis Ohaus [syn. by Mondaca 2016: 9].

##### Distribution.

CHILE: Atacama, Coquimbo (CMNC), O’Higgins, Valparaíso, Metropolitan Region (FSCA), Maule (CMNC), Bío Bío (Ohaus 1905, 1918, 1934b, Blackwelder 1944, Gutiérrez 1949, 1951, Martínez 1953, Machatschke 1972, Mondaca 2005, Krajcik 2008, Mondaca 2016).

##### Types.

**Figure 47.**
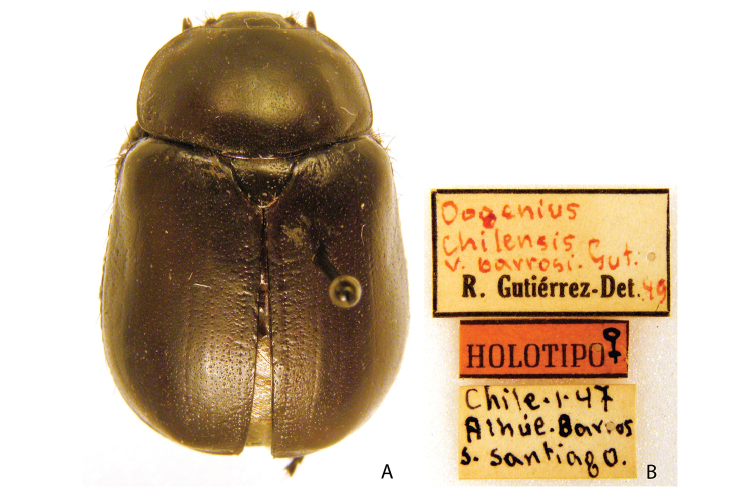
Oogenius chilensis var. barrosi Gutiérrez (valid name *Oogenius chilensis* Ohaus) holotype female from UCCC. **A** Dorsal habitus **B** Specimen labels.

Lectotype ♂ of *Oogenius chilensis* at ZMHB (Mondaca 2016). One paralectotype ♂ and two ♀ paralectotypes of *Oogenius chilensis* at ZSMC. Holotype ♀ of Oogenius chilensis var. barrosi at UCCC (Fig. 47).

##### Remarks.

*Oogenius chilensis barrosi* Gutiérrez was proposed originally as a “variety” of *O. chilensis* Ohaus (Gutiérrez 1949). Per ICZN Article 45.6.4 this name was not unambiguously infrasubspecific based on designation by the author or the other content of the work.

#### Oogenius kuscheli

Taxon classificationAnimaliaColeopteraScarabaeidae

Gutiérrez, 1949

Oogenius kuscheli Gutiérrez, 1949: 29–30 [original combination].
Oogenius (Oogenius) kuscheli Gutiérrez [new subgeneric combination by Gutiérrez 1951: 110].
Oogenius kuscheli Gutiérrez [revised combination by Soula 2006: 139].

##### Distribution.

CHILE: Bío Bío (Gutiérrez 1949, 1951, Martínez 1953, Machatschke 1972, Mondaca 2005, Krajcik 2008, Mondaca 2016).

##### Types.

Holotype ♂ of *Oogenius kuscheli* at UCCC (Gutiérrez 1949, Mondaca 2016).

#### Oogenius lariosae

Taxon classificationAnimaliaColeopteraScarabaeidae

Martínez, 1953

Oogenius (Oogenius) lariosae Martínez, 1953: 76, 77–81 [original combination].
Oogenius lariosae Martínez [removal of subgeneric classification by Soula 2006: 139].

##### Distribution.

ARGENTINA: Chubut (CMNC), Mendoza, Río Negro (Martínez 1953, Mondaca 2005, Krajcik 2008, Mondaca 2016).

##### Types.

**Figure 48.**
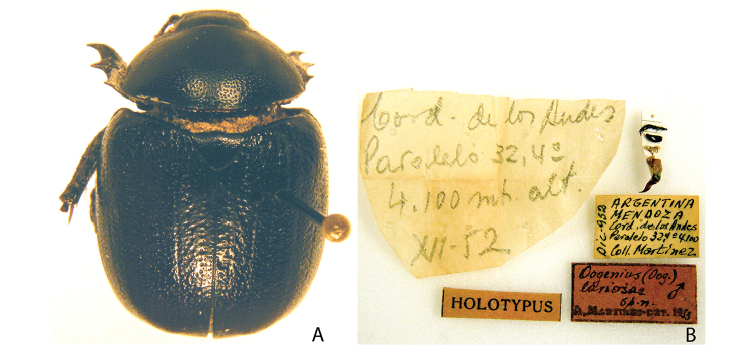
Oogenius (Oogenius) lariosae Martínez (valid name *Oogenius lariosae* Martínez) male holotype from MACN. **A** Dorsal habitus **B** Specimen labels, male genitalia, and specimen parts.

Holotype ♂ of Oogenius (Oogenius) lariosae Martínez at MACN (Fig. 48) (Mondaca 2016).

#### Oogenius penai

Taxon classificationAnimaliaColeopteraScarabaeidae

Mondaca, 2005

Oogenius gutierrezi Martínez & Peña, 1994 [original combination].
Oogenius (Oogenius) penai Mondaca [new replacement name by Mondaca 2005: 19].
Oogenius penai Mondaca [revised combination by Soula 2006: 139].

##### Distribution.

CHILE: Metropolitan Region (Martínez and Peña-Guzmán 1994, Mondaca 2005, Krajcik 2008, Mondaca 2016).

##### Types.

**Figure 49.**
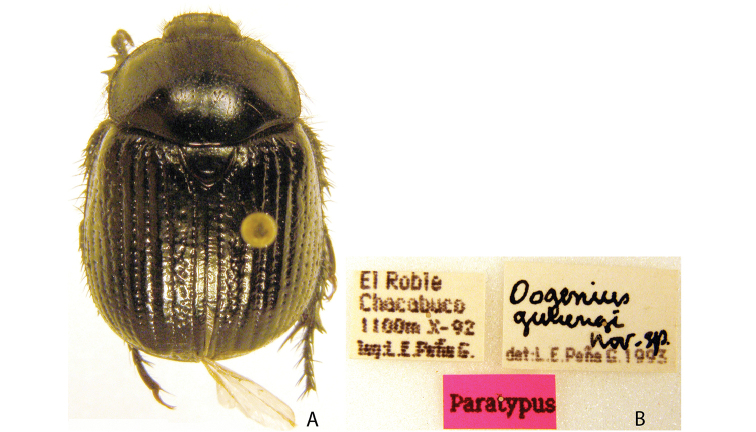
*Oogenius gutierrezi* Martínez and Peña (valid name *O. penai* Mondaca) paratype from PVGH. **A** Dorsal habitus **B** Specimen labels.

Holotype ♂ of *Oogenius gutierrezi* at MNNC (Mondaca 2016); 2 paratype specimens at MNNC; 1 paratype specimen at PVGH (Fig. 49); 6 ♂ paratypes in CMNC; 2 specimens at MACN that are labeled as holotype and allotype are not valid type specimens (pers. comm. José Mondaca, Aug. 2016).

#### Oogenius virens

Taxon classificationAnimaliaColeopteraScarabaeidae

Solier, 1851

Oogenius virens Solier, 1851: 98 [original combination].
Oogenius (Oogenius) virens Solier [new subgeneric combination by Gutiérrez 1951: 109].
Oogenius virens Solier [revised combination by Soula 2006: 139].

##### Distribution.

CHILE: Coquimbo, Valparaíso (Reed 1876, Philippi 1887, Ohaus 1905, 1910c, 1918, 1934b, Blackwelder 1944, Gutiérrez 1949, 1951, Martínez 1953, Machatschke 1972, Mondaca 2005, Krajcik 2008, Mondaca 2016).

##### Types.

Lectotype ♂ of *Oogenius virens* at MNHN (Mondaca 2016).

#### 

Taxon classificationAnimaliaColeopteraScarabaeidae

Soula, 2006

Pachacama Soula, 2006: 113–114.

##### Type species.

*Pachacama ocampoi ocampoi* Soula, 2006: 113–114, by original designation.

##### Gender.

Feminine.

##### Species.

2 subspecies.

#### Pachacama ocampoi cagnarensis

Taxon classificationAnimaliaColeopteraScarabaeidae

Soula, 2006

Pachacama ocampoi cagnarensis Soula, 2006: 115 [original combination].

##### Distribution.

ECUADOR: Cañar (Soula 2006).

##### Types.

The following specimen is deposited at CCECL. 1 ♂ holotype: “El Triumfo (500m) (E) Prov. de Cañar Cañar 2/90//Holotype 2006 *Pachacama ocampoi caniarensis* (sic) Soula det. S.” (47031072). Box 4618686 SOULA.

#### Pachacama ocampoi ocampoi

Taxon classificationAnimaliaColeopteraScarabaeidae

Soula, 2006

Pachacama ocampoi Soula, 2006: 113–114 [original combination].

##### Distribution.

ECUADOR: Pichincha (Soula 2006).

##### Types.

**Figure 50.**
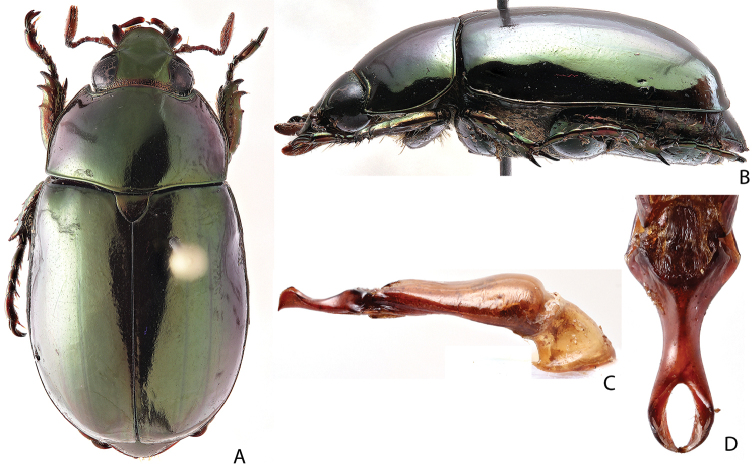
*Pachacama ocampoi* Soula male from MSPC. **A** Dorsal habitus **B** Lateral habitus **C** Male genitalia, lateral view **D** Male parameres, dorsal view.

The following specimens are deposited at CCECL. 1 ♂ holotype, 1 ♀ allotype: “ECUADOR OCCIDENTE PINCHINCHA ancienne rte. QUITO/SANTO DOMINGO Km 78 (1650 m) 25 dec. 1978 Rec. Th. PORION//COLL. TH. PORION//Holotype 2006. *Pachacama ocampoi* S. Soula det.” (47030951); “Pacto Pichincha Equateur M. SOULA det 19//Allotype 2006 *Pachacama ocampoi ocampoi* S. Soula” (47031071). Genitalia card-mounted underneath the male holotype. Box 4618686 SOULA and 4616344 PORION. An exemplar specimen is figured (Fig. 50).

#### 

Taxon classificationAnimaliaColeopteraScarabaeidae

Ohaus, 1915

Parhomonyx Ohaus, 1915b: 257–258.

##### Type species.

*Homonyx fuscoaeneus* Ohaus, 1905: 313–314, by monotypy.

##### Gender.

Masculine.

##### Species.

1 species.

#### Parhomonyx fuscoaeneus

Taxon classificationAnimaliaColeopteraScarabaeidae

(Ohaus, 1905)

Homonyx fuscoaeneus Ohaus, 1905: 313–314 [original combination].
Parhomonyx fuscoaeneus (Ohaus) [new combination by Ohaus 1915b: 257–258].

##### Distribution.

ARGENTINA: Catamarca (FSCA), San Luis (FSCA), Córdoba, Santa Fe, Santiago del Estero (Ohaus 1905, 1915b, 1918, 1934b, Blackwelder 1944, Machatschke 1972, Krajcik 2008, Soula 2010a).

##### Types.

**Figure 51.**
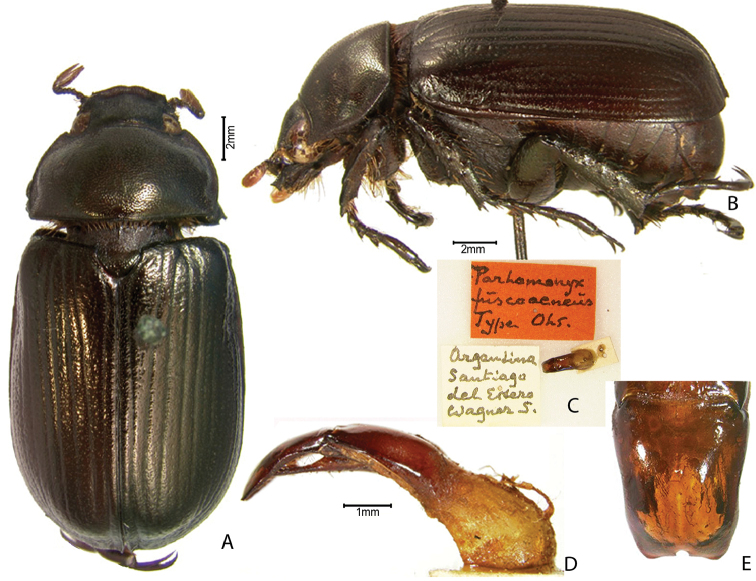
*Homonyx fuscoaeneus* Ohaus (valid name *Parhomonyx fuscoaeneus* [Ohaus]) male type (see “*Type specimens and lectotype designation*” in Methods) from ZMHB. **A** Dorsal habitus **B** Lateral habitus **C** Specimen labels, mouthparts, and male genitalia **D** Male genitalia, lateral view **E** Male parameres, dorsal view.

1 ♂ lectotype specimen of *Homonyx fuscoaeneus* and 3 paralectotypes at ZMHB (Soula 2010a) (Fig. 51).

##### Remarks.

One paralectotype of *P. fuscoaeneus*, labeled “R. d. JANEIRO Therespolis” (=Theresopolis, Rio de Janeiro, Brazil), is disjunct from all other known localities. Other than this specimen, we have not examined any specimens outside of northern Argentina, and we believe that the data on this label are in error.

#### 

Taxon classificationAnimaliaColeopteraScarabaeidae

Ohaus, 1915

Parhoplognathus Ohaus, 1915b: 257.

##### Type species.

*Areoda maculata* Gory, 1833b, by original designation.

##### Gender.

Masculine.

##### Species.

4 species.

#### Parhoplognathus bousqueti

Taxon classificationAnimaliaColeopteraScarabaeidae

Soula, 2008

Parhoplognathus bousqueti Soula, 2008: 9 [original combination].

##### Distribution.

BRAZIL (Soula 2008).

##### Types.

1 ♂ holotype at ZMHB (Soula 2008).

##### Remarks.

This species was described based on one male specimen from “Brazil”. Soula (2008) compares the species with *P. limbatipennis*, with which it shares many similarities.

#### Parhoplognathus limbatipennis

Taxon classificationAnimaliaColeopteraScarabaeidae

(Ohaus, 1905)

Hoplognathus limbatipennis Ohaus, 1905: 323–324 [original combination].
Parhoplognathus limbatipennis (Ohaus) [new combination by Ohaus 1915b: 257].

##### Distribution.

BRAZIL: Minas Gerais, Rio de Janeiro, (Ohaus 1905, 1918, 1934b, Blackwelder 1944, Machatschke 1972, Krajcik 2008, Soula 2008).

##### Types.

**Figure 52.**
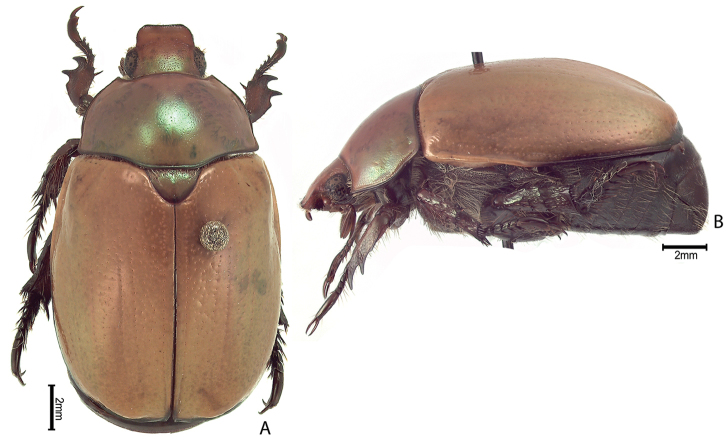
*Parhoplognathus limbatipennis* (Ohaus) from USNM. **A** Dorsal habitus **B** Lateral habitus.

1 ♂ lectotype and 1 paralectotype probably at ZMHB (Soula 2008). Exemplar specimen shown from USNM (Fig. 52).

#### Parhoplognathus maculatus

Taxon classificationAnimaliaColeopteraScarabaeidae

(Gory, 1833)

Areoda maculata Gory, 1833b: new taxon N°13 [original combination].
Hoplognathus maculatus (Gory) [new combination by Burmeister 1844: 429].
Parhoplognathus maculatus (Gory) [new combination by Ohaus 1915b: 257].
Pelidnota bimaculata Laporte, 1840 **synonym.**
Pelidnota bimaculata Laporte, 1840: 122–123 [original combination].
Hoplognathus maculatus (Gory) [syn. by F. Bates 1904: 260].

##### Distribution.

BRAZIL (Laporte 1840, Burmeister 1844, Ohaus 1918, 1934b, Blackwelder 1944, Machatschke 1972, Krajcik 2008, Soula 2008).

##### Types.

1 ♂ syntype of *Areoda maculata* at IRSNB (described as a holotype by Soula 2008).

#### Parhoplognathus parvulus

Taxon classificationAnimaliaColeopteraScarabaeidae

(Ohaus, 1905)

Hoplognathus parvulus Ohaus, 1905: 323 [original combination].
Parhoplognathus parvulus (Ohaus) [new combination by Ohaus 1915b: 257].

##### Distribution.

BRAZIL: Santa Catarina (Ohaus 1905, 1918, 1934b, Blackwelder 1944, Machatschke 1972, Krajcik 2008, Soula 2008).

##### Types.

1 ♂ lectotype and 1 paralectotype at ZMHB (Soula 2008).

##### Remarks.

Kracjik (2008) considered “P. parvulus var. rubripennis Ohaus” to be a synonym of *P. parvulus* (Ohaus). However, because the name P. parvulus var. rubripennis Ohaus is infrasubspecific (see “*Parhoplognathus rubripennis* Soula, 2008”) and therefore unavailable, this nomenclatural act was not valid. Machatschke (1972) had also maintained the infrasubspecific status of “P. parvulus var. rubripennis Ohaus” and he listed it as a “forma”.

## Unavailable names in *Parhoplognathus* (application of ICZN Articles 45.6 and 16.1)

Krajcik (2008) listed P. parvulus var. rubripennis in synonymy with *Parhoplognathus parvulus* (Ohaus). However, Parhoplognathus parvulus var. rubripennis was unambiguously described as infrasubspecific based on the content of Ohaus (1930a), wherein he described both subspecies and varieties. Subsequent usage of this name (Ohaus 1934b) referred to it in an infrasubspecific manner (“var. rubripennis”) and the name is unavailable according to ICZN Article 45.6.1. The name was referred to as “forma” (Machatschke 1972), thus establishing another unavailable name (Article 45.6.3). Soula (2008), however, elevated *Parhoplognathus rubripennis* to species status and attributed the name to Ohaus (1930a). Because Parhoplognathus parvulus var. rubripennis Ohaus was an unavailable name, Moore and Jameson (2013) mistakenly considered Soula (2006) the valid author of *Parhoplognathus rubripennis*. ICZN Article 16.1 states that “Every new name published after 1999, including new replacement names (*nomina nova*), must be explicitly indicated as intentionally new.” Soula (2008) did not explicitly state that *Parhoplognathus rubripennis* was an intentionally new name, rather he considered *P. rubripennis* as having “n. statut”. We apply ICZN Article 16.1 herein, and *Parhoplognathus rubripennis* Soula is an **unavailable name**.

### Parhoplognathus rubripennis

Taxon classificationAnimaliaColeopteraScarabaeidae

Soula, 2008 Unavailable, invalid name

Parhoplognathus parvulus var. rubripennis Ohaus, 1930a: 138 [original combination, **unavailable, invalid name** per ICZN Articles 45.6.1; 1.3.4].
Parhoplognathus parvulus forma rubripennis Machatschke, 1972: 9 [original combination, **unavailable, invalid name** per ICZN Article 45.6.3].
Parhoplognathus rubripennis Soula, 2008: 7 [original combination, **unavailable name** per ICZN Article 16.1].

#### Distribution.

BRAZIL: Bahia (Ohaus 1930a, 1934b, Machatschke 1972, Krajcik 2008, Soula 2008).

#### Types.

1 invalid ♀ holotype probably at ZMHB (Soula 2008).

### 

Taxon classificationAnimaliaColeopteraScarabaeidae

Soula, 2008

Patatra Soula, 2008: 40.

#### Type species.

*Patatra mathani* Soula, 2008: 40, by monotypy.

#### Gender.

Feminine.

#### Species.

1 species.

### Patatra mathani

Taxon classificationAnimaliaColeopteraScarabaeidae

Soula, 2008

Patatra mathani Soula, 2008: 40 [original combination].

#### Distribution.

BRAZIL: Pará (Soula 2008, 2009).

#### Types.

According to Soula (2008), the holotype and only known specimen of *Patatra mathani* (which represents a monotypic genus) was from the Oberthur Collection, and thus should have been deposited in the MNHN. However, a search for the holotype was unable to find the specimen. According to A. Mantilleri (MNHN), the specimen was not deposited at MNHN (pers. comm from A. Mantilleri, Aug. 2016).

#### Remarks.

This species was described based on one male specimen (at MNHN). Soula (2008, 2009) stated that the species possessed characters of both pelidnotine and anticheirine scarabs, and the species was ultimately classified in among the pelidnotine scarabs (Soula 2011). *Verbatim* descriptions of this taxon in two separate works (Soula 2008, 2009) created a double case of homonymy. To stabilize the nomenclature, replacement names were proposed for both the genus and the species (Moore and Jameson 2013), but this was incorrect and only further confused the issue. Both names, *Neopatatra* Moore and Jameson and *Neopatatra synonyma* Moore and Jameson, are **unavailable, invalid names** because they were not used as valid names in their original publication. Because the Soula names are objective synonyms with the same type species and type specimen, the junior synonyms will never be in a position where they will be considered a separate taxon in need of a replacement names.

### PELIDNOTA

Taxon classificationAnimaliaColeopteraScarabaeidae

MacLeay, 1819

Pelidnota MacLeay, 1819: 157–158.
Aglycoptera Sharp, 1885 **synonym.**
Aglycoptera Sharp, 1885: xxiii–xxiv. [Type species. *Aglycoptera lacerdae* Sharp, 1885 by monotypy (= *Pelidnota burmeisteri burmeisteri* Burmeister, 1844)].
Pelidnota MacLeay [syn. by Ohaus 1934b: 75].
Pelidnota (Ganonota) Ohaus, 1915b **synonym.**
Pelidnota (Ganonota) Ohaus, 1915b: 259. [Type species. *Rutela cuprea* Germar, 1824, by subsequent designation (Machatschke 1972: 26)].
Pelidnota (Strigidia) Burmeister [syn. by Machatschke 1970: 157].
Pelidnota MacLeay [syn. by Soula 2009: 115].
Pelidnota (Delipnia) Casey, 1915 **synonym.**
Pelidnota (Delipnia) Casey, 1915: 80. [Type species. *Pelidnota belti* Sharp, 1877 by monotypy].
Pelidnota (Ganonota) Ohaus [syn. by Ohaus 1934b: 82].
Pelidnota (Pelidnotidia) Casey, 1915 **synonym.**
Pelidnota (Pelidnotidia) Casey, 1915: 77. [Type species. *Pelidnota strigosa* Laporte, 1840, by original designation].
Pelidnota (Pelidnota) MacLeay [syn. by Hardy 1974: 89].
Strigidia Burmeister, 1844 **synonym.**
Strigidia Burmeister, 1844: 388–389. [Type species. *Rutela cuprea* Germar, 1824, by original designation (Burmeister 1844: 389)].
Pelidnota (Ganonota) Ohaus [syn. by Ohaus 1934b: 75].
Pelidnota (Strigidia) Burmeister [syn. by Machatschke 1970: 157].
Pelidnota (Odontognathus) Laporte [syn. by Hardy 1975: 4].
Pelidnota (Strigidia) Burmeister [syn. by Özdikmen 2009: 144].
Pelidnota MacLeay [syn. by Soula 2009: 115].
Odontognathus Laporte, 1840 **synonym.**
Odontognathus Laporte, 1840: 137. [Type species. *Odontognathus unicolor* Laporte, 1840, by monotypy (= *Pelidnota cuprea* (Germar, 1824))].
Pelidnota (Odontognathus) Laporte [new subgeneric status by Ohaus 1913: 504].
Pelidnota (Ganonota) Ohaus [syn. by Ohaus 1934b: 75].
Pelidnota (Strigidia) Burmeister [syn. by Machatschke 1970: 157].
Pelidnota (Odontognathus) Laporte [revised subgeneric status by Hardy 1975: 4].
Pelidnota (Strigidia) Burmeister [syn. by Özdikmen 2009: 144].
Pelidnota MacLeay [syn. by Soula 2009: 115].
Heteropelidnota Ohaus, 1912. **synonym.**
Heteropelidnota Ohaus, 1912: 309–310. [Type species. *Heteropelidnota kuhnti* Ohaus, 1912, by monotypy].
Pelidnota MacLeay [syn.].

#### Type species.

*Scarabaeus punctatus* Linnaeus, 1758: 350, by monotypy (MacLeay 1819: 158).

#### Gender.

Feminine.

#### Species.

194 species and subspecies.

### Pelidnota abracadabra

Taxon classificationAnimaliaColeopteraScarabaeidae

Soula, 2009

Pelidnota abracadabra Soula, 2009: 31, 62–63 [original combination].

#### Distribution.

MEXICO: Colima, Guerrero, Jalisco (Soula 2009).

#### Types.

The following specimens are deposited at CCECL. 1 ♂ holotype, 1 probable ♂ paratype: “Manzillo Mexique XII 86 Dr F. GARNIER//Holotype 2008 *Pelidnota abracadabra* S. Soula det.” (47030490); “MEXIQUE CHAMELA (JAL) STATION U N A M 6 7-IX-1984 D&B SIGWALT REC//[unwritten red label]//Probable paratype *Pelidnota abracadabra* S. det. MR Moore ‘15” (47030491). Genitalia card-mounted underneath the male holotype. Box 4618666 SOULA.

#### Remarks.

In his description, Soula (2009: 63) mentioned an additional male specimen from Jalisco, Mexico, and he illustrated the protarsal claw of this specimen (Soula 2009: 63). In the CCECL collection, one male specimen possesses a blank, red label, and we consider this specimen a probable paratype of *P. abracadabra* Soula.

### Pelidnota acconciai

Taxon classificationAnimaliaColeopteraScarabaeidae

Soula, 2009

Pelidnota acconciai Soula, 2009: 30, 49 [original combination].

#### Distribution.

VENEZUELA: Apure (Soula 2009).

#### Types.

The ♂ holotype of *Pelidnota acconciai* is at MNHN. The following specimen is deposited at CCECL. 1 ♂ paratype: 87//MUSEUM PARIS, RIVES DE L’ORÉNOQUE, CHAFFANJON 1887//Ohaus determ. *Pelidnota lucida* Brm.//Paratype 2008 *Pelidnota acconciai* S. Soula” (47030497). Genitalia are card-mounted underneath the male paratype. Box 4618668 SOULA.

### Pelidnota agnesae

Taxon classificationAnimaliaColeopteraScarabaeidae

Soula, 2010

Pelidnota agnesae Soula, 2010a: 56 [original combination].

#### Distribution.

BRAZIL: Mato Grosso (Soula 2010a).

#### Types.

The following specimen is deposited at CCECL. 1 probable ♂ holotype: “Mato Grosso BRESIL 2-1980 Coll. Th. PORION//Holotype [blank] Soula//Probable holotype *Pelidnota agnesae* Soula det. MR Moore ‘15” (47030948). Genitalia card-mounted underneath the probable male holotype. Box 4616343 PORION.

### Pelidnota alliacea

Taxon classificationAnimaliaColeopteraScarabaeidae

(Germar, 1824)

Rutela alliacea Germar, 1824: 117 [original combination].
Pelidnota glauca (Olivier) [syn. by Burmeister 1844: 401].
Pelidnota aeruginosa L. [syn. by Harold 1869b: 1221].
Pelidnota alliacea (Germar) [revised species status and new combination by Ohaus 1908a: 250].
Pelidnota (Pelidnota) alliacea (Germar) [new subgeneric combination by Ohaus 1918: 22].
Pelidnota alliacea (Germar) [revised combination by Soula 2009: 74].
Melolontha americana Herbst, 1790 **synonym.**
Melolontha americana Herbst, 1790: 66 [original combination].
Pelidnota glauca (Olivier) [syn. by Burmeister 1844: 402].
Pelidnota aeruginosa (Linnaeus) [syn. by Harold 1869b: 1221].
Pelidnota alliacea (Germar) [syn. by Soula 2009: 74].
Melolontha glauca Olivier, 1789 **synonym.**
Melolontha glauca Olivier, 1789: 21 [original combination].
Pelidnota glauca (Olivier) [new combination by Laporte 1840: 122].
Pelidnota aeruginosa (Linnaeus) [syn. by Harold 1869b: 1221].
Pelidnota alliacea (Germar) [syn. by Soula 2009: 74].
Rutela prasina Germar, 1824 **synonym.**
Rutela prasina Germar, 1824: 117–118 [original combination].
Pelidnota glauca (Olivier) [syn. by Burmeister 1844: 402].
Pelidnota aeruginosa (Linnaeus) [syn. by Harold 1869b: 1221].
Pelidnota alliacea (Germar) [syn. by Soula 2009: 74].

#### Distribution.

BRAZIL: Espírito Santo, Santa Catarina (Olivier 1789, 1802, Laporte 1840, Burmeister 1844, Blanchard 1851, Ohaus 1908a, 1918, 1934b, Blackwelder 1944, Machatschke 1972, Krajcik 2008, Soula 2009). SURINAME (Herbst 1790).

#### Types.

1 ♂ lectotype and 3 paralectotypes of *Rutela alliacea* at ZMHB (Soula 2009).

#### Remarks.

The species *Melolontha americana* Herbst, *Melolontha glauca* Olivier, and *Rutela prasina* Germar were previously treated as synonyms of *Pelidnota aeruginosa* (Linnaeus) (*nomen dubium*) in catalogs of Rutelini (Ohaus 1918, 1934b, Machatschke 1972). Krell et al. (2012) noted that the *nomen dubium* status of *P. aeruginosa* (Linnaeus) necessitates the re-examination of the primary type material, where possible, of several species to resolve issues of identity, nomenclatural priority, and proper synonymy of species previously compared to *P. aeruginosa* (Linnaeus). We agree with this strategy. *Melolontha glauca* would appear to have nomenclatural priority based on the publication year (1789), however the species associated with this name may be an anomaline rather than a pelidnotine (Krell et al. 2012). The following species are in need of evaluation to resolve this issue: *Pelidnota rioensis* Soula, *P. semiaurata semiaurata* Burmeister, *P. semiaurata citripennis* Ohaus, *Rutela alliacea* Germar, *R. prasina* Germar, *R. caesarea* Gistel, *Melolontha glauca* Olivier, and *M. americana* Herbst.

*Pelidnota aeruginosa* (Linnaeus) was designated as a “*nomen nullum*” by Soula (2009), but he did not clearly address all of the names listed in synonymy under this species. Instead, Soula (2009) considered *Melolontha glauca* and *M. americana* as synonyms of *P. alliacea* (Germar). *Rutela prasina* Germar was listed in synonymy with two species simultaneously: *Pelidnota arnaudi arnaudi* Soula (an unavailable name) and *P. alliacea* (Germar) (Soula 2009). The discussions of *P. alliacea* and *P. arnaudi arnaudi* included identical language that is not helpful for resolving what Soula (2009: 73, 74) meant by this double synonymy: “*Rutela prasina* Germar, 1824, Ins. spec. nov., p. 117. Burmeister la place en synonymie avec *aeruginosa* en 1844. Cette fois, il doit bien s’agir de notre *arnaudi*, puisque c’est lui qui a décrit les deux autres!”. Due to this confusing language, we cautiously list *Rutela prasina* Germar as a junior synonym of *Pelidnota alliacea* (Germar).

### Pelidnota alutacea

Taxon classificationAnimaliaColeopteraScarabaeidae

H. W. Bates, 1888

Pelidnota strigosa var. alutacea H. W. Bates, 1888: 276 [original combination].
Pelidnota (Pelidnota) strigosa Laporte [syn. by Hardy 1975: 18].
Pelidnota alutacea H. W. Bates [removal of subgeneric classification and new species status by Soula 2009: 59–60]

#### Distribution.

COSTA RICA (H. W. Bates 1888, Blackwelder 1944, Krajcik 2008, Soula 2009). PANAMA: Chiriquí (H. W. Bates 1888, Blackwelder 1944, Krajcik 2008, Soula 2009).

#### Types.

1 ♂ lectotype at BMNH (Soula 2009) and 2 paralectotypes at BMNH with following label data: “para-lecto-type [obverse] Syn-type [circle with blue border]//Costa Rica.//Van Patten//Strigosa var alutacea Bates//B.C.A, Coll., 11(2)//Pelidnota strigosa”.

### Pelidnota ancilla

Taxon classificationAnimaliaColeopteraScarabaeidae

F. Bates, 1904

Pelidnota ancilla F. Bates, 1904: 258, 267–268 [original combination].
Pelidnota (Pelidnota) ancilla F. Bates [new subgeneric combination by Ohaus 1918: 22].
Pelidnota ancilla F. Bates [removal of subgeneric classificationby Soula 2009: 109].

#### Distribution.

BRAZIL: Espírito Santo, Goiás, Santa Catarina (F. Bates 1904, Ohaus 1918, 1934b, Blackwelder 1944, Machatschke 1972, Soula 2009).

#### Types.

**Figure 53.**
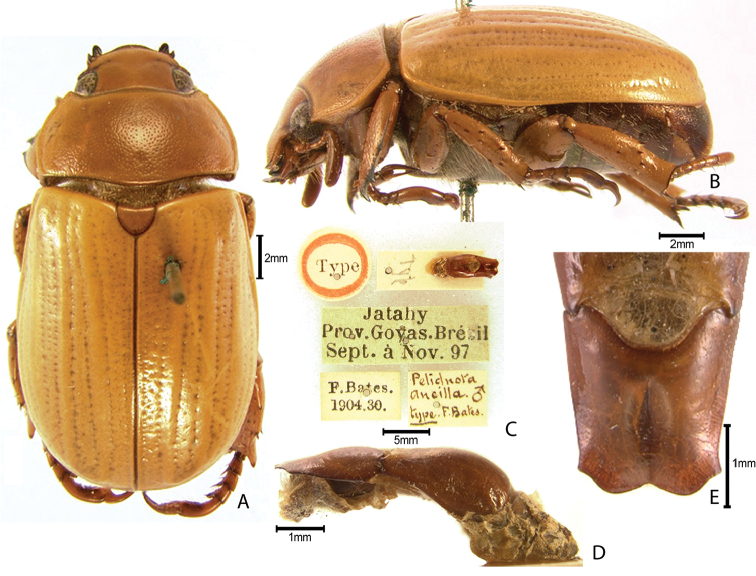
*Pelidnota ancilla* F. Bates male holotype from BMNH. **A** Dorsal habitus **B** Lateral habitus **C** Specimen labels and male genitalia **D** Male genitalia, lateral view **E** Male parameres, dorsal view.

1 ♂ holotype at BMNH (Soula 2009) (Fig. 53).

### Pelidnota angiae

Taxon classificationAnimaliaColeopteraScarabaeidae

Demez & Soula, 2010

Pelidnota angiae Demez & Soula, 2010a: 56–57 [original combination].

#### Distribution.

PERU: Junín (Soula 2010a, Ratcliffe et al. 2015).

#### Types.

The following specimens are deposited at CCECL. 1 ♂ holotype, 2 ♂ paratypes: “Atalaya, Ucayali Pérou, V/2010//Holotype 2010 *Pelidnota angiae* S. Soula” (47030232); “Atalaya, Ucayali Pérou, V/2010//Paratype 2010 *Pelidnota angiae* S. Soula” (47030233); “Satipo Junin XI/2007 M. SOULA det 19//Paratype 2010 *Pelidnota angiae* S. Soula” (47030234). Genitalia card-mounted underneath male holotype and one male paratype. Box 4618657 SOULA.

### Pelidnota aurescens

Taxon classificationAnimaliaColeopteraScarabaeidae

H. W. Bates, 1888

Pelidnota virescens var. aurescens H. W. Bates, 1888: 274 [original combination].
Pelidnota aurescens H. W. Bates [new species status by Ohaus 1913: 498].
Pelidnota (Pelidnota) aurescens H. W. Bates [new subgeneric combination by Ohaus 1918: 22].
Pelidnota aurescens H. W. Bates [removal of subgeneric classification by Soula 2009: 64–65].

#### Distribution.

GUATEMALA: Escuintla, Quetzaltenango, San Marcos, Sololá (H. W. Bates 1888, Ohaus 1913, 1918, 1934b, Blackwelder 1944, Machatschke 1972, Hardy 1975, Krajcik 2008, Soula 2009). MEXICO: Chiapas, Oaxaca, Veracruz (Carrillo et al. 1966, Hardy 1975, Thomas 1993, Morón et al. 1997, Pacheco Flores et al. 2008, Soula 2009).

#### Types.

1 ♂ lectotype at BMNH (Hardy 1975, Soula 2009); 5 paralectotypes at BMNH (Soula 2009); 3 paralectotypes at MNHN (Soula 2009). The following specimens are deposited at CCECL. 1 ♂ Paralectotype: “El Zumbador, 2500 ft. Champion.//H.W.Bates Biol.Cent.Amer.//2008 *Pelidnota aurescens* Bates M. SOULA det 19//Paralectotype 2008 Pelidnota virescens var. aurescens B. Soula det.” (47030487). Genitalia card-mounted underneath the male paralectotype. Box 4618666 SOULA.

### Pelidnota bahiana adriani

Taxon classificationAnimaliaColeopteraScarabaeidae

Martínez, 1982

Pelidnota (Odontognathus) adriani Martínez, 1982: 65–68 [original combination].
Strigidia bahiana adriani (Martínez) [new combination and new subspecific status by Soula 2006: 63–64].
Pelidnota (Strigidia) adrianae Martínez [revised combination, revised species status, and new subgeneric classification by Özdikmen 2009: 145].
Pelidnota bahiana adriani Martínez [removal of subgeneric classification and revised subspecific status by Soula 2009: 115].

#### Distribution.

BRAZIL: Espírito Santo (Martínez 1982, Soula 2006, Krajcik 2008).

#### Types.

**Figure 54.**
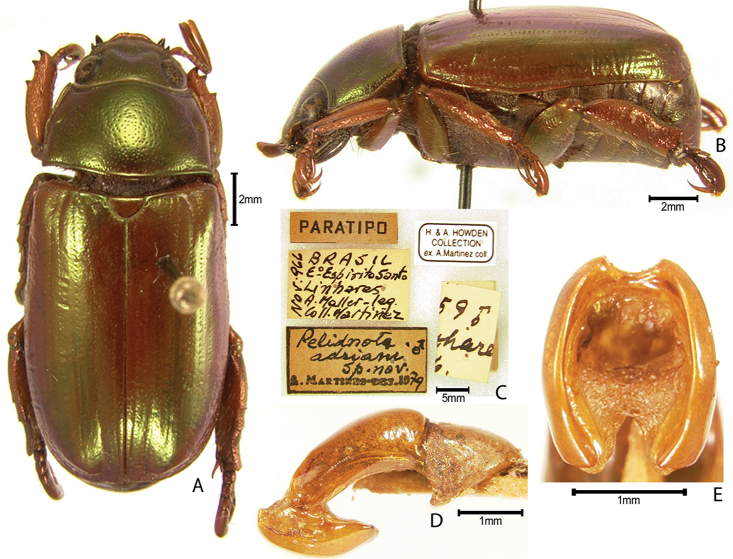
Pelidnota (Odontognathus) adriani Martínez (valid name *Pelidnota bahiana adriani* [Martínez]) paratype male from CMNC. **A** Dorsal habitus **B** Lateral habitus **C** Specimen labels **D** Male genitalia, lateral view **E** Male parameres, caudal view.

Holotype and allotype specimens of Pelidnota (Odontognathus) adriani at MACN; 1 ♂ (Fig. 54) paratype and 2 ♀ paratypes at CMNC.

### Pelidnota bahiana bahiana

Taxon classificationAnimaliaColeopteraScarabaeidae

Ohaus, 1905

Pelidnota bahiana Ohaus, 1905: 315–316 [original combination].
Pelidnota (Chalcoplethis) bahiana Ohaus [new subgeneric combination by Ohaus 1918: 29].
Strigidia bahiana (Ohaus) [new combination by Soula 2006: 63].
Pelidnota bahiana Ohaus [revised combination by Soula 2009: 115].

#### Distribution.

BRAZIL: Bahia (Ohaus 1905, 1918, 1934b, Blackwelder 1944, Machatschke 1972, Soula 2006, Krajcik 2008).

#### Types.

1 ♂ syntype at ZMHB (Soula 2006).

### Pelidnota belti belti

Taxon classificationAnimaliaColeopteraScarabaeidae

Sharp, 1877

Pelidnota belti Sharp, 1877: 132 [original combination].
Pelidnota (Delipnia) belti Sharp [new subgeneric combination by Casey 1915: 80].
Pelidnota (Ganonota) belti Sharp [new subgeneric combination by Ohaus 1918: 25].
Pelidnota (Strigidia) belti Sharp [new subgeneric combination by Machatschke 1970: 157].
Pelidnota (Odontognathus) belti Sharp [new subgeneric combination by Hardy 1975: 4].
Strigidia belti (Sharp) [new combination by Soula 2006: 71–72].
Pelidnota (Strigidia) belti Sharp [revised subgeneric combination by Özdikmen 2009: 145].
Pelidnota belti belti Sharp [removal of subgeneric classification and revised subspecific status by Soula 2009: 115].

#### Distribution.

COLOMBIA: Boyacá (Ohaus 1918, 1934b, Blackwelder 1944, Machatschke 1972, Hardy 1975, Maes 1987, Restrepo et al. 2003). COSTA RICA: Alajuela, Cartago (Hardy 1975, Solís and Morón 1994, García-López et al. 2013). NICARAGUA: Chontales (H. W. Bates 1888, Ohaus 1918, 1934b, Blackwelder 1944, Machatschke 1972, Hardy 1975, Maes 1987, Soula 2006, Krajcik 2008). PANAMA: Chiriquí, Panama (H. W. Bates 1888, Ohaus 1918, 1934b, Blackwelder 1944, Machatschke 1972, Hardy 1975, Maes 1987, Ratcliffe 2002).

#### Types.

1 ♀ lectotype specimen of *Pelidnota belti* at BMNH (Hardy 1975, Soula 2006); 1 ♂ paralectotype at BMNH; 1 paralectotype at MNHN (Soula 2006).

### Pelidnota belti boyacaensis

Taxon classificationAnimaliaColeopteraScarabaeidae

(Soula, 2006)

Strigidia belti boyacaensis Soula, 2006: 73 [original combination].
Pelidnota (Strigidia) belti boyacaensis (Soula) [new combination and new subgeneric combination by Özdikmen 2009: 145].
Pelidnota belti boyacaensis (Soula) [removal of subgeneric classification by Soula 2009: 115].

#### Distribution.

COLOMBIA: Boyacá (Soula 2006).

#### Types.

The following specimens are deposited at CCECL. 1 ♂ holotype, 1 ♀ allotype, 7 ♂ paratypes, 4 ♀ paratypes: “*Strigidia belti* Otanche (C) 4/89//Holotype 2006 *Strigidia belti boyacaensis* Soula” (47030320); “Otanche COLOMBIE 1987 Coll. A. Hayoz//Allotype 2006 *Strigidia belti boyacaensis* Soula” (47030321); “Otanche COLOMBIE 1987 Coll. A. Hayoz//Paratype 2006 *Strigidia belti boyacaensis* Soula” (47030322); “Otanche Colombie 1983 A. Hayoz//Paratype 2006 *Strigidia belti boyacaensis* Soula” (47030323); “Otanche Colombie 1987//Paratype 2006 *Strigidia belti boyacaensis* S. Soula” (47030324); “OTANCHE IV-1986//Paratype 2006 *Strigidia belti boyacaensis* S. Soula” (47030325); “Otanché Colombie V/86//Paratype 2006 *Strigidia belti boyacaensis* S. Soula” (47030326); “Otanche (Col.)//Paratype 2006 *Strigidia belti boyacaensis* S. Soula” (47030327); “Otanche. C. IX/85 Coll. Megard.//Paratype 2006 *Strigidia belti boyacaensis* S. Soula” (47030328); “Strigidia belti Otanche. C. IX/85 IX Coll. Megard.//Paratype 2006 *Strigidia belti boyacaensis* S. Soula” (47030329); “Nouvelle Grenade Etat Cundinamarca Cananche M.de Mathan 1^er^ Sem. 1900//Paratype 2006 *Strigidia belti boyacaensis* S. Soula” (47030330); “Muzo - Colombie 06/93 coll. – SOULA//Paratype 2006 *Strigidia belti boyacaensis* S. Soula” (47030331). “Muzo - Colombie//COLL. TH. PORION//Paratype 2006 *Strigidia belti boyacaensis* S. Soula” (47030942). Genitalia card-mounted underneath the male holotype and four male paratypes. Box 4618661 SOULA and 4616343 PORION. Two additional paratype specimens are deposited at BMNH.

### Pelidnota belti guatemalaensis

Taxon classificationAnimaliaColeopteraScarabaeidae

(Soula, 2006)

Strigidia belti guatemalaensis Soula, 2006: 72 [original combination].
Pelidnota (Strigidia) belti guatemalaensis (Soula) [new combination and new subgeneric combination by Özdikmen 2009: 145].
Pelidnota belti guatemalaensis (Soula) [removal of subgeneric classification by Soula 2009: 115].

#### Distribution.

GUATEMALA: Izabal (Soula 2006).

#### Types.

The following specimens are deposited at CCECL. 1 ♂ holotype, 1 ♀ allotype, 3 ♂ paratypes: “Finca Firmeza, Sierra de Caral, Morales, Izabal, Guatemala, 450m, 20/V/2006//Holotype 2006 *Strigidia belti guatemalensis* S. Soula” (47030342); “Finca Firmeza, Sierra de Caral, Morales, Izabal, Guatemala, 450m, 20/V/2006//Allotype 2006 *Strigidia belti guatemalensis* S. Soula” (47030343); Two paratypes with identical label data: “Finca Firmeza, Sierra de Caral, Morales, Izabal, Guatemala, 450m, 20/V/2006//Paratype 2006 *Strigidia belti guatemalensis* S. Soula” (47030344 and 47030345); “GUATEMALA, Izabal Morales, Junio 2000 600 m90m//COLL. TH. PORION// *Strigidia belti ssp* ? M. SOULA det 19//Paratype 2006 *Strigidia belti guatemalensis* S. Soula” (47030346). Genitalia card-mounted underneath the male holotype and the three male paratypes. Box 4618661 SOULA. One additional paratype specimen is deposited at BMNH.

### Pelidnota belti panamaensis

Taxon classificationAnimaliaColeopteraScarabaeidae

(Soula, 2006)

Strigidia belti panamaensis Soula, 2006: 72–73 [original combination].
Pelidnota (Strigidia) belti panamaensis (Soula) [new combination and new subgeneric combination by Özdikmen 2009: 145].
Pelidnota belti panamaensis (Soula) [removal of subgeneric classification by Soula 2009: 115].

#### Distribution.

PANAMA: Chiriquí (Soula 2006).

#### Types.

The following specimens are deposited at CCECL. 1 ♂ holotype, 1 ♀ allotype, 3 ♂ paratypes, 5 ♀ paratypes: “Chiriqui//H.W.Bates Biol.Cent.Amer. Muséum Paris ex Coll. R. Oberthür 1952//Holotype 2006 *Strigidia belti panamensis* Soula” (47030332); “V. de Chiriqui M.de Mathan 1901//Allotype 2006 *Strigidia belti panamensis* Soula” (47030333); “V. de Chiriqui M.de Mathan 1901//Muséum Paris ex Coll. R. Oberthür 1952//Paratype 2006 *Strigidia belti panamensis* S. Soula” (47030334); “Chiriqui//Muséum Paris ex Coll. R. Oberthür 1952//Paratype 2006 *Strigidia belti panamensis* S. Soula” (47030335); “Panama Chiriqui IV.86 col. DURANTON//Paratype 2006 *Strigidia belti panamensis* S. Soula” (47030336); “PANAMA CHIRIQUI prov. Santa Clara env. - 1440 m 18. 5. - 15. 6. 2003 Vlad. Malý lgt. P - 2//Pelidnota (Strigidia) belti Sharp. Det. V. Malý 2003//Paratype 2006 *Strigidia belti panamensis* S. Soula” (47030337); “PANAMA CHIRIQUI prov. Santa Clara env. - 1440 m 28. 5. - 23. 6. 2002 Vlad. Malý lgt. P - 1//Pelidnota (Strigidia) belti Sharp Det. V. Malý 200//Paratype 2006 *Strigidia belti panamensis* S. Soula” (47030338); “PANAMA CHIRIQUI Santa Clara env. - 1546 m 08°51'42.2"N:082°44'36.5"W 17.6.-4.7.06;V.Malý lgt. P7//Pelidnota (Strigidia) belti Sharp Det. Vl. Malý 2006//Paratype 2006 *Strigidia belti panamensis* S. Soula” (47030339); “Serro (sic!) Campana Panama M. SOULA det [obverse] 700 m V/07//Paratype 2006 *Strigidia belti panamensis* S. Soula” (47030340); Cerro Campana, 3000’, Panama. July 31, 1970, H. & A. Howden//Paratype 2006 *Strigidia belti panamensis* S. Soula” (47030341). Genitalia card-mounted underneath the male holotype and one male paratype. Box 4618661 SOULA. 1 ♂ and 3 ♀ paratypes at CMNC: 1 ♂ “Cerro Campana, 3000’ Panama. July 31, 1970, H. & A, Howden//H.&A. Howden Collection// H. & A. HOWDEN COLLECTION *ex.* A. Martinez coll.// *Pelidnota* ♂ *belti* Sharp det.A.R.Hardy 1970//Paratype *Strigidia belti panamensis* S. Soula”, 1 ♀ “Cerro Campana, 3000’ Panama. July 31, 1970, H. & A, Howden//H.&A. Howden Collection//*Pelidnota* ♀ *belti* Sharp det.A.R.Hardy 1970//Paratype 2006 *Strigidia belti panamensis* S. Soula”, 1 ♀ “Panama Chiriqui Prov Santa Clara 4000’ Col: R.Hartmann 3 May 197// Paratype 2006. *Strigidia belti panamensis* S. Soula”, 1 ♀ “Panamá: Panamá Pr. Cerro Campana, 850M 8°40’N 79°56’W//24 iv. 1970 H. A. Hespenheide//ON PALM//Paratype 2006 *Strigidia belti panamensis* S. Soula//*Pelidnota belti* Sharp DET. H.F. HOWDEN 70”.

### Pelidnota beniouioui

Taxon classificationAnimaliaColeopteraScarabaeidae

Soula, 2010

Pelidnota beniouioui Soula, 2010a: 41–42 [original combination].

#### Distribution.

BOLIVIA: Beni (Soula 2010a).

#### Types.

The following specimens are deposited at CCECL. 1 ♂ holotype, 1 ♀ allotype, 6 ♂ paratypes: “Beni 1000 m. Bol. coll. – SOULA [obverse] Bolivie VIII/96.//Holotype 2010 *Pelidnota beniouioui* S. Soula” (47030224); “Beni (1000 m) Bolivie coll. - SOULA [obverse] VIII/96//Allotype *Pelidnota beniouioui* S. 2010 Soula” (47030225); “Beni, La Paz [arrow] coll. – SOULA [obverse] Rurrenabaque pk 298 VIII/94 1000 m//Paratype 2010 *Pelidnota beniouioui* Soula” (47030226); Five paratypes with identical labels “Beni, La Paz [arrow] coll. – SOULA [obverse] Rurrenabaque pk 298 1000 m 1/VIII/94//Paratype 2010 *Pelidnota beniouioui* Soula” (47030227 to 47030231). Genitalia card-mounted underneath the holotype, allotype and 5 paratypes. Box 4618656 SOULA.

### Pelidnota beraudi

Taxon classificationAnimaliaColeopteraScarabaeidae

Soula, 2009

Pelidnota beraudi Soula, 2009: 33, 105 [original combination].

#### Distribution.

COLOMBIA: Caldas (Soula 2009).

#### Types.

The holotype, allotype, and some paratypes are deposited at MNHN (Soula 2009). The following specimens are deposited at CCECL. 1 ♂ paratype, 1 ♀ paratype: “ A. M. Patino//Paratype *Pelidnota beraudi* S. Soula det. 2008” (47030669); “ A. M. Patino//Muséum Paris Coll. R. Oberthür//Paratype 2008 *Pelidnota beraudi* S. Soula” (47030670). Genitalia card-mounted underneath the male paratype. Box 4618679 SOULA.

### Pelidnota bertrandi

Taxon classificationAnimaliaColeopteraScarabaeidae

Soula, 2009

Pelidnota bertrandi Soula, 2009: 31, 59 [original combination].

#### Distribution.

NICARAGUA: Rivas (Soula 2009).

#### Types.

The following specimens are deposited at CCECL. 1 ♂ holotype, 1 ♀ allotype, 7 ♂ paratypes, 5 ♀ paratypes: “Route MANAGUA/RIVAS Km 14,5 (NICARAGUA) IX-1969 [7 crossed out] Chasses M. DARGE//Holotype 2007 *Pelidnota bertrandi* S. Soula” (47030473); “Route MANAGUA/RIVAS Km 14,5 (NICARAGUA) IX-1969 [7 crossed out] Chasses M. DARGE//Allotype 2007 *Pelidnota bertrandi* S. Soula” (47030474); “Route MANAGUA/RIVAS Km 14,5 (NICARAGUA) IX-1969 [7 crossed out] Chasses M. DARGE//Paratype 2007 *Pelidnota bertrandi* S. Soula” (47030475); Eleven paratypes with identical labels “Route MANAGUA/RIVAS Km 14,5 (NICARAGUA) IX-1969 [7 crossed out] Chasses M. DARGE//Paratype 2008 *Pelidnota bertrandi* S. Soula” (47030476 to 47030484, exch26 and exch27). Genitalia card-mounted underneath the holotype and two male paratypes. Box 4618665 SOULA.

### Pelidnota bivittata

Taxon classificationAnimaliaColeopteraScarabaeidae

(Swederus, 1787)

Scarabaeus bivattatus Swederus, 1787: 189 [original combination].
Rutela bivittata (Swederus) [new combination by Schönherr 1817: 155].
Pelidnota bivittata (Swederus) [new combination by Burmeister 1844: 550–551].
Pelidnota (Ganonota) bivittata (Swederus) [new subgeneric combination by Ohaus 1918: 28].
Pelidnota (Strigidia) bivittata (Swederus) [new subgeneric combination by Machatschke 1970: 157].
Pelidnota (Odontognathus) bivittata (Swederus) [new subgeneric combination by Hardy 1975: 4].
Strigidia bivittata (Swederus) [new combination by Soula 2006: 44–45].
Pelidnota (Strigidia) bivittata (Swederus) [revised subgeneric combination by Özdikmen 2009: 145].
Pelidnota bivittata (Swederus) [removal of subgeneric classification by Soula 2009: 115].

#### Distribution.

BRAZIL: Espírito Santo (Ohaus 1918, 1934b, Blackwelder 1944, Machatschke 1972, Soula 2006, Krajcik 2008).

### Pelidnota bleuzeni

Taxon classificationAnimaliaColeopteraScarabaeidae

(Bouchard, 2003)

Chalcoplethis bleuzeni Bouchard, 2003: 103, 105–107 [original combination].
Strigidia bleuzeni (Bouchard) [new combination by Soula 2008: 34].
Pelidnota bleuzeni (Bouchard) [new combination by Soula 2009: 115].

#### Distribution.

FRENCH GUIANA (Bouchard 2003, Krajcik 2008, Soula 2008, 2010c). VENEZUELA: Bolivar (MLJC).

#### Types.

The following specimens are deposited at CCECL. 2 ♂ paratypes, 12 ♀ paratypes: “Mgne de Kaw G. F. 08/92//*Chalcoplethis bleuzeni* sp. n. PARATYPE” (47030155); Three paratypes with identical label data “KAW. PK 40 27/8/84 [obverse] P. L.//*Chalcoplethis bleuzeni* sp. n. PARATYPE” (47030156 and 47030157, exch08); “KAW. PK 40 23/8/84 [obverse] P. L.//*Chalcoplethis bleuzeni* sp. n. PARATYPE” (47030158); “Guyane f. Kourou VIII 90//*Chalcoplethis bleuzeni* sp. n. PARATYPE” (47030164); “GUYANE Française Roura I 85 J. P. MARECHAL//*Chalcoplethis bleuzeni* sp. n. PARATYPE” (47030166); “08/1997 P.K. 39-Rte de KAW GUYANE FRANCAISE FRENCH GUIANA//*Chalcoplethis bleuzeni* sp. n. PARATYPE” (47030162); Two paratypes with identical label data “Petit Saut G. F. 07/92//*Chalcoplethis bleuzeni* sp. n. PARATYPE” (47030167 and 47030168); “M de Kaw Guyane fr. 8.90//*Chalcoplethis bleuzeni* sp. n. PARATYPE” (47030163); “KAW. KAW. PK34 21/9/84 [obverse] P. L.//*Chalcoplethis bleuzeni* sp. n. PARATYPE” (47030160); “Kaw PK 34 P. L. 28/7/84 Kaw//*Chalcoplethis bleuzeni* sp. n. PARATYPE” (47030159); “Coll P. BLEUZEN Mgne de Kaw PK 31 GUYANE FR. 17 IX 1985//*Chalcoplethis bleuzeni* sp. n. PARATYPE” (47030165); “GUYANE FRANCAISE Piste de Kaw pK 38 11-VII-1996 H. de Toulgoët & J. Navatte réc//*Chalcoplethis bleuzeni* sp. n. PARATYPE” (47030161). Genitalia card-mounted underneath two male paratypes and two female paratypes. Box 4618655 SOULA.

### Pelidnota bondili

Taxon classificationAnimaliaColeopteraScarabaeidae

(Soula, 2006)

Strigidia bondili Soula, 2006: 10, 38–39 [original combination].
Pelidnota bondili (Soula) [new combination by Soula 2009: 115].

#### Distribution.

PERU: Amazonas (Soula 2006, Ratcliffe et al. 2015).

#### Types.

The holotype ♂ of *P. bondili* should be at CCECL (Soula 2006), but we did not find it there.

### Pelidnota boulangeri

Taxon classificationAnimaliaColeopteraScarabaeidae

Soula, 2009

Pelidnota boulangeri Soula, 2009: 33, 96–97 [original combination].

#### Distribution.

VENZUELA: Aragua, Distrito Federal, Merida (Soula 2009).

#### Types.

The following specimens are deposited at CCECL. 1 ♂ holotype, 1 ♀ allotype, 7 ♂ paratypes, 7 ♀ paratypes: “P. N. Henri Pittier Choroni; Venezuela V-VI/2005//Holotype 2008 *Pelidnota boulangeri* S. Soula” (47030625); “P. N. Henri Pittier Choroni; Venezuela V-VI/2005//Allotype 2008 *Pelidnota boulangeri* S. Soula” (47030626); six paratypes with identical label data: “P. N. Henri Pittier Choroni; Venezuela V-VI/2005//Paratype 2008 *Pelidnota boulangeri* Soula” (47030627 to 47030631, exch36); “VENEZUELA Rancho Grande 1150^m^ 3-VII-1986//Paratype 2008 *Pelidnota boulangeri* Soula” (47030632); “Caracas (V) 9/87//Paratype 2008 *Pelidnota boulangeri* Soula” (47030633); “Caracas Venezuela IX/87//Paratype 2008 *Pelidnota boulangeri* Soula” (47030634); “Caracas et environs//Paratype 2008 *Pelidnota boulangeri* Soula” (47030635); two paratypes with identical label data: “N. Venezuela S. Klages 1904//Paratype 2008 *Pelidnota boulangeri* Soula” (47030636 and 47030637); two paratypes with identical label data: “N. Venezuela S. Klages 1904//Paratype *Pelidnota boulangeri* S. 2008-2009” (47030638 and 47030639). Genitalia card-mounted underneath the male holotype and five male paratypes. Box 4618676 SOULA.

### Pelidnota boyi

Taxon classificationAnimaliaColeopteraScarabaeidae

Ohaus, 1929

Pelidnota (Ganonota) boyi Ohaus, 1929: 389–390 [original combination].
Pelidnota (Strigidia) boyi Ohaus [new subgeneric combination by Machatschke 1970: 157].
Pelidnota (Odontognathus) boyi Ohaus [new subgeneric combination by Hardy 1975: 4].
Strigidia boyi (Ohaus) [new combination by Soula 2006: 25–26].
Pelidnota (Strigidia) boyi Ohaus [revised subgeneric combination by Özdikmen 2009: 145].
Pelidnota boyi Ohaus [removal of subgeneric classification by Soula 2009: 115].

#### Distribution.

BRAZIL: Amazonas (Ohaus 1929, 1934b, Blackwelder 1944, Machatschke 1972, Ohaus 1934b, Machatschke 1972, Soula 2006, Krajcik 2008).

#### Types.

**Figure 55.**
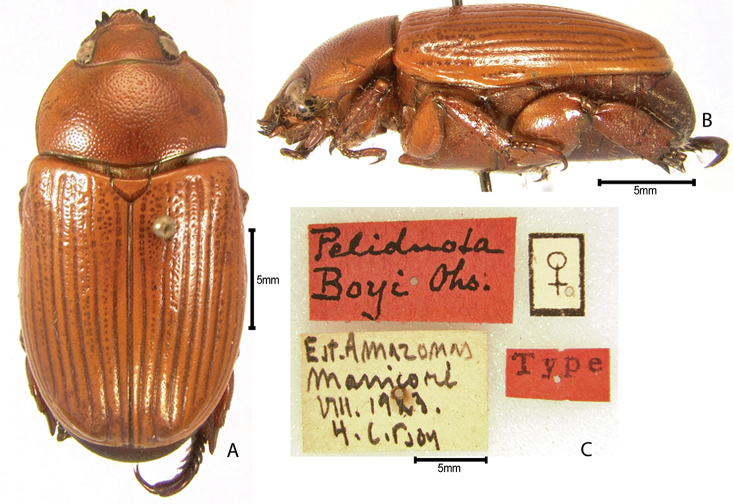
Pelidnota (Ganonota) boyi Ohaus (valid name *Pelidnota boyi* Ohaus) holotype female from ZMHB. **A** Dorsal habitus **B** Lateral habitus **C** Specimen labels.

1 ♀ holotype specimen of Pelidnota (Ganonota) boyi Ohaus at ZMHB (Fig. 55).

### Pelidnota burmeisteri burmeisteri

Taxon classificationAnimaliaColeopteraScarabaeidae

Burmeister, 1844

Pelidnota burmeisteri Burmeister, 1844: 409 [original combination].
Pelidnota (Pelidnota) burmeisteri Burmeister [new subgeneric combination by Ohaus 1918: 25].
Pelidnota burmeisteri Burmeister [removal of subgeneric classification by Soula 2009: 37–38].
Aglycoptera lacerdae Sharp, 1885 **synonym.**
Aglycoptera lacerdae Sharp, 1885: 23–24 [original combination].
Pelidnota (Pelidnota) burmeisteri Burmeister [syn. by Ohaus 1918: 25].

#### Distribution.

BRAZIL: Bahia, Minas Gerais (Ohaus 1918, 1934b, Blackwelder 1944, Machatschke 1972, Krajcik 2008, Soula 2009), Mato Grosso (WBWC).

#### Types.

**Figure 56.**
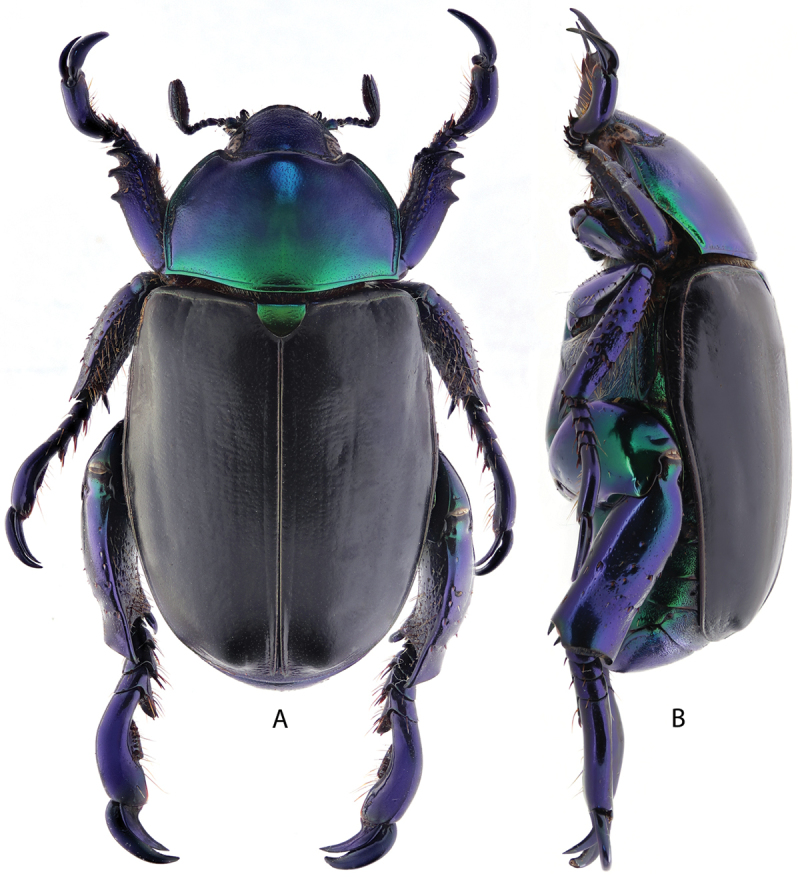
*Pelidnota burmeisteri burmeisteri* Burmeister male specimen from MSPC. **A** Dorsal habitus **B** Lateral habitus.

The type of *Pelidnota burmeisteri burmeisteri* is not at MLUH and is possibly lost (Soula 2009). An exemplar specimen from MSCP is shown (Fig. 56).

### Pelidnota burmeisteri tricolor

Taxon classificationAnimaliaColeopteraScarabaeidae

Nonfried, 1894

Pelidnota tricolor Nonfried, 1894: 123–124 [original combination].
Pelidnota sumptuosa var. tricolor Nonfried [new infrasubspecific status by F. Bates 1904: 260].
Pelidnota burmeisteri var. tricolor Nonfried [revised infrasubspecific status by Ohaus 1905: 317].
Pelidnota (Pelidnota) burmeisteri var. tricolor Nonfried [new subgeneric combination by Ohaus 1918: 25].
Pelidnota burmeisteri tricolor Nonfried [new combination and new subpecific status by Soula 2009: 38–39].
Pelidnota ludovici Ohaus, 1905 **synonym.**
Pelidnota ludovici Ohaus, 1905: 317 [original combination].
Pelidnota (Pelidnota) ludovici Ohaus [new subgeneric combination by Ohaus 1918: 25].
Pelidnota ludovici Ohaus [removal of subgeneric classification by Soula 2009: 39–40].
Pelidnota burmeisteri tricolor Nonfried [**syn. n.**].

#### Distribution.

BRAZIL: Bahia, Espírito Santo, Mato Grosso (Ohaus 1905, 1918, 1934b, Blackwelder 1944, Machatschke 1972, Krajcik 2008, Soula 2009).

#### Types.

**Figure 57.**
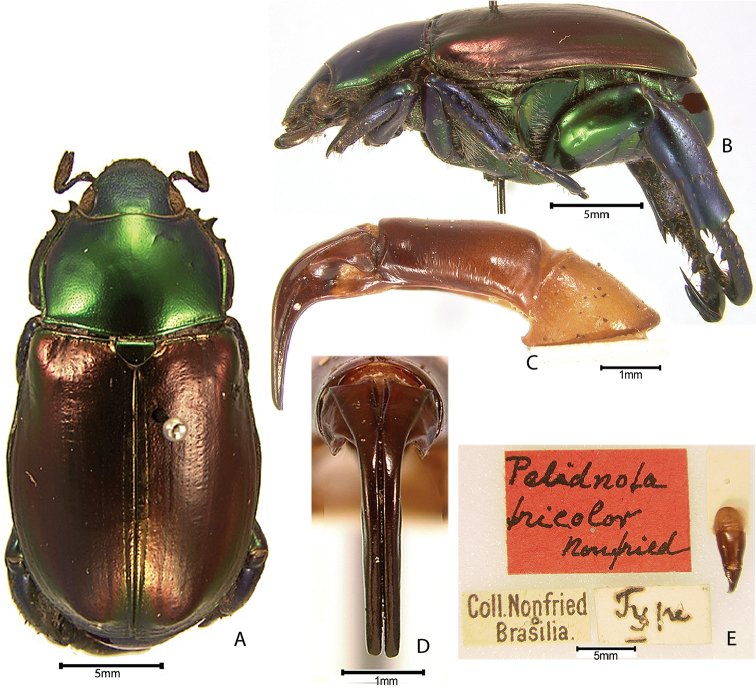
*Pelidnota tricolor* Nonfried (valid name *Pelidnota burmeisteri tricolor* Nonfried) syntype male from ZMHB. **A** Dorsal habitus **B** Lateral habitus **C** Male genitalia, lateral view **D** Male parameres, caudal view **E** Specimen labels.

1 ♂ syntype specimen of *Pelidnota tricolor* Nonfried at ZMHB (Fig. 57). 1 ♂ holotype of *Pelidnota ludovici* at ZMHB (Soula 2009).

#### Remarks.

Ohaus (1905) compared *P. ludovici* with *P. burmeisteri*. The holotype specimen was collected on the flowers of “mimosa”. The species was described based on a single male specimen that was collected by his brother in the state of Espírito Santo on the bank of the Rio Doce between Baixo Guandu and Timbuhy (collecting date Dec. 21, 1898) (Ohaus 1905). *Pelidnota ludovici* is a metallic green morphotype of *P. burmeisteri*. We considered that the holotype may represent a teneral specimen of *P. burmeisteri*, but we examined two specimens both from Espírito Santo (probably representing different collecting events). The ventral surface is metallic rufous with metallic green shine in *P. ludovici* (black with metallic green in *P. burmeisteri*); legs are metallic rufous or purple (black in *P. burmeisteri*); head is shiny, metallic green (also in *P. burmeisteri*); pronotum, scutellum, and elytra are metallic rufous with green shine (pronotum and scutellum metallic green, elytra black and shiny in *P. burmeisteri*). Based on comparison of types of *P. ludovici* and *P. burmeisteri tricolor* (male genitalia and other characters), we consider these taxa to be conspecific. *Pelidnota ludovici* Ohaus is a **new synonym** of *Pelidnota burmeisteri tricolor*.

### Pelidnota carlettii

Taxon classificationAnimaliaColeopteraScarabaeidae

Soula, 2009

Pelidnota carlettii Soula, 2009: 32, 77–78 [original combination].

#### Distribution.

ARGENTINA: Misiones (Soula 2009).

#### Types.

The following specimens are deposited in CCECL. 1 ♂ holotype, 1 ♀ allotype, 9 ♂ paratypes, 3 ♀ paratypes, 1 ♂ invalid paratype, 4 ♀ invalid paratypes: “Oberá Misiones Ar I/99 M. SOULA det 19//Holotype 2008 *Pelidnota carlettii* S. Soula” (47030588); “Eldorado - Misiones ARGENTINE (I/93)//Allotype 2008 *Pelidnota carlettii* S. Soula” (47030589); Three paratypes with identical label data: “Eldorado - Misiones ARGENTINE (I/93)//Paratype 2008 *Pelidnota carlettii* S. Soula” (47030590 to 47030592); Three paratypes with identical label data: “Puerto Iguazu-ARG XII/88.//Paratype 2008 *Pelidnota carlettii* S. Soula” (47030593 to 47030595); “Puerto Iguazu ARGENTINE (I/93)//Paratype 2008 *Pelidnota carlettii* S. Soula” (47030596); “Oberá - Misiones ARGENTINA-I/99 Col. Andrés Varga//Paratype 2008 *Pelidnota carlettii* S. Soula” (47030597); Two paratypes with identical label data: Iguazu Misiones (Ar.) coll. – SOULA//Paratype 2008 *Pelidnota carlettii* S. Soula” (47030598 and 47030599); “*Pelidnota sordida* ♀ Puerto Iguazu Misiones Argentina 22-DIC 1987 det. Forster” (47030600); “Argentina//Paratype 2008 *Pelidnota carlettii* S. Soula” (47030601); Two invalid paratypes with identical label data: “Puerto Iguazu Misiones, Argentine II/1995//Paratype *Pelidnota carlettii* S. 2006 Soula//Invalid paratype *Pelidnota carlettii* Soula det. MR Moore ‘15” (47030602 and 47030603)”; “Puerto Iguazu Misiones, Argentine II/1995//Paratype 2006 *Pelidnota carlettii* S. Soula//Invalid paratype *Pelidnota carlettii* Soula det. MR Moore ‘15” (47030604)”; Two invalid paratypes with identical label data: “Dos de Mayo Misiones M. SOULA det 19 [obverse] 1/II/89//Paratype *Pelidnota carlettii* S. 2007 Soula//Invalid paratype *Pelidnota carlettii* Soula det. MR MOORE ‘15” (47030605 and 47030606). Genitalia card-mounted underneath the male holotype, nine male paratypes and one female paratype. Box 4618671 SOULA.

#### Remarks.

Five specimens labeled as *Pelidnota carlettii* Soula paratypes in CCECL are considered invalid. These specimens have label data that are not reported in Soula (2009) and are thus invalid.

### Pelidnota cayennensis

Taxon classificationAnimaliaColeopteraScarabaeidae

F. Bates, 1904

Pelidnota cayennensis F. Bates, 1904: 258, 269–270 [original combination].
Pelidnota laevissima cayennensis F. Bates [new subspecific status by Ohaus 1913: 499].
Pelidnota (Pelidnota) laevissima cayennensis F. Bates [new subgeneric combination by Ohaus 1918: 23].
Pelidnota cayennensis F. Bates [removal of subgeneric classification and revised species status by Soula 2009: 107–108].

#### Distribution.

FRENCH GUIANA: St.-Laurent du Maroni (F. Bates 1904, Ohaus 1913, 1918, 1934b, Blackwelder 1944, Machatschke 1972, Krajcik 2008, Soula 2009). VENEZUELA: Delta Amacuro (F. Bates 1904, Soula 2009).

#### Types.

1 ♂ lectotype and 1 paralectotype of *Pelidnota cayennensis* at BMNH (Soula 2009).

#### Remarks.

Krajcik (2012, 2013) considered *P. cayennensis* to be a subspecies of *P. laevissima*.

### Pelidnota centroamericana

Taxon classificationAnimaliaColeopteraScarabaeidae

Ohaus, 1913

Pelidnota punctata centroamericana Ohaus, 1913: 499–500 [original combination].
Pelidnota (Pelidnota) punctata centroamericana Ohaus [new subgeneric combination by Ohaus 1918: 24].
Pelidnota (Pelidnota) lutea centroamericana Ohaus [revised subspecific status by Ohaus 1934b: 80].
Pelidnota (Pelidnota) centroamericana Ohaus [new species status by Hardy 1975: 34].
Pelidnota centroamericana Ohaus [removal of subgeneric classification by Soula 2009: 67–68].

#### Distribution.

BELIZE: Corozal (Hardy 1975, Morón et al. 1985, 1997, Soula 2009). GUATEMALA (Ohaus 1913, 1918, 1934b, Blackwelder 1944, Hardy 1975, Morón et al. 1985, 1997, Krajcik 2008, Soula 2009). HONDURAS: Cortés (Ohaus 1913, 1918, 1934b, Hardy 1975, Morón et al. 1985, 1997, Soula 2009). MEXICO: Campeche, Chiapas, Quintana Roo, Tabasco, Veracruz, Yucatán (Blackwelder 1944, Hardy 1975, Palacios-Rios et al. 1990, Thomas 1993, Morón et al. 1985, 1997, Reyes Novelo and Morón 2005, Soula 2009, Morón-Ríos and Morón 2016).

#### Types.

1 ♂ lectotype of *Pelidnota punctata centroamericana* at ZMHB (Hardy 1975, Soula 2006).

### Pelidnota cerdai

Taxon classificationAnimaliaColeopteraScarabaeidae

(Soula, 2006)

Strigidia cerdai Soula, 2006: 11, 48–49 [original combination].
Pelidnota cerdai (Soula) [new combination by Soula 2009: 115].

#### Distribution.

FRENCH GUIANA: Cayenne (Soula 2006, 2010c).

#### Types.

The following specimens are deposited at CCECL. 1 ♂ holotype, 1 ♀ allotype, 1 ♂ paratype: “FRG PK12 PL. 18/2/85//Holotype *Strigidia cerdai* S. 2005. Soula” (47030421). “Cayenne//So named in Reiches Collection. C.W.//67.45//*nitidula* Reiche Cayenne//*Pelidnota* sp. ♀. *Belti* mit falsch. Fündort //534// Allotype *Strigidia cerdai* S. 2005 Soula” (47030422). “Kaw PK 37 PL. 24/12/84//Paratype *Strigidia cerdai* S. 2005 Soula” (47030423). Male genitalia card-mounted underneath male holotype and paratype. Box 4618663 SOULA.

### Pelidnota chalcopus

Taxon classificationAnimaliaColeopteraScarabaeidae

H. W. Bates, 1888

Pelidnota virescens var. chalcopus H. W. Bates, 1888: 275 [original combination].
Pelidnota (Pelidnota) virescens var. chalcopus H. W. Bates [new subgeneric combination by Ohaus 1918: 24].
Pelidnota (Pelidnota) virescens chalcopus H. W. Bates [new subspecific status by Machatschke 1972: 25].
Pelidnota (Pelidnota) aurescens H. W. Bates [syn. by Hardy 1975: 24].
Pelidnota chalcopus H. W. Bates [removal of subgeneric classification and new species status by Soula 2009: 67].

#### Distribution.

BELIZE: Cayo (H. W. Bates 1888, Hardy 1975, Krajcik 2008, Soula 2009). GUATEMALA (H. W. Bates 1888, Ohaus 1918, Ohaus 1934b, Machatschke 1972, Krajcik 2008, Soula 2009). HONDURAS (Ohaus 1918, Ohaus 1934b, Machatschke 1972).

#### Types.

1 ♂ lectotype and 3 paralectotypes of Pelidnota virescens var. chalcopus at BMNH (Soula 2009).

### Pelidnota chalcothorax

Taxon classificationAnimaliaColeopteraScarabaeidae

Perty, 1830

Pelidnota chalcothorax Perty, 1830: 48 [original combination].
Pelidnota (Pelidnota) chalcothorax Perty [new subgeneric combination by Ohaus 1918: 22].
Pelidnota chalcothorax Perty [removal of subgeneric classification by Soula 2009: 93–94].

#### Distribution.

BRAZIL: Mato Grosso (WBWC), Espírito Santo, Minas Gerais, Rio de Janeiro, São Paulo (Perty 1830, Laporte 1840, Burmeister 1844, 1855, Blanchard 1851, Ohaus 1908a, 1918, 1934b, Blackwelder 1944, Machatschke 1972, Krajcik 2008, Soula 2009).

### Pelidnota championi

Taxon classificationAnimaliaColeopteraScarabaeidae

F. Bates, 1904

Pelidnota championi F. Bates, 1904: 258, 267 [original combination].
Pelidnota (Pelidnota) fulva championi F. Bates [new subgeneric combination and new subspecific status by Ohaus 1918: 23].
Pelidnota championi F. Bates [removal of subgeneric classification and revised species status by Soula 2009: 88–89].

#### Distribution.

ARGENTINA: Córdoba, Misiones, Tucumán (F. Bates 1904, Ohaus 1918, 1934b, Machatschke 1972, Krajcik 2008, Soula 2009).

#### Types.

1 ♂ lectotype of *Pelidnota championi* F. Bates at BMNH (Soula 2009).

#### Remarks.

Krajcik (2012, 2013) considered *P. championi* to be a subspecies of *P. fulva*.

### Pelidnota chiapasensis

Taxon classificationAnimaliaColeopteraScarabaeidae

Soula, 2009

Pelidnota chiapasensis Soula, 2009: 31, 65–66 [original combination].

#### Distribution.

MEXICO: Chiapas (Soula 2009).

#### Types.

The following specimens are deposited at CMNC. 1 ♂ holotype, 1 ♀ allotype, 2 ♀ paratypes: “MEXICO, Chiapas, Cinco Cerros, Km30 on Hwy 190 1500m 8. VI.1989. H.Howden//at light//Holotype 2008 *Pelidnota chiapasensis* Sou Soula//[barcode matrix] Canadian Museum of Musée canadien de la NATURE CMNEN 00010903”, allotype with identical collecting data label and database number CMNEN 00010904, paratypes with identical collecting data label and database numbers CMNEN 00010905 and CMNEN 00010906. The following specimens are deposited at CCECL. 2 ♀ paratypes, 1 invalid ♂ paratype: “MEXICO. Chiapas, Cinco Cerros 860m. 9. VI. 1990 H. & A. Howden//at light//Paratype 2008 *Pelidnota chiapasensis* S. Soula det.” (47030492); “MEXICO. Chiapas ElAguacero, 16 km W Ocozocoautla. 680m 5. VI. 1990 H. & A. Howden//Paratype 2008 *Pelidnota chiapasensis* S. Soula det.” (47030493); “MEXICO. Chiapas ElAguacero, 16 km W Ocozocoautla. 680m 10. VI. 1990 H. & A. Howden//at light//Paratype 2008 *Pelidnota chiapasensis* S. Soula//Invalid Paratype *Pelidnota chiapasensis* S. det. M.R. Moore ‘15” (47030494). Genitalia card-mounted underneath the invalid male paratype. Box 4618666 SOULA.

#### Remarks.

Box 4618666 SOULA contains a male *P. chiapasensis* Soula from Mexico (Chiapas, El Aguacero) labeled as a paratype. This specimen is an invalid paratype as this specific specimen was not reported in Soula (2009: 65–66). The paratype label is also a slightly different color than the other two paratypes, indicating that this paratype was added to the series after publication of Soula (2009).

### Pelidnota chibchana

Taxon classificationAnimaliaColeopteraScarabaeidae

Ohaus, 1922

Pelidnota chibchana Ohaus, 1922: 324–325 [original combination].
Pelidnota (Chalcoplethis) chibchana Ohaus [new subgeneric combination by Ohaus 1934b: 85].
Strigidia chibchana (Ohaus) [new combination by Soula 2006: 84].
Pelidnota chibchana Ohaus [removal of subgeneric classification by Soula 2009: 115].

#### Distribution.

COLOMBIA: Cundinamarca, Distrito Capital, Santander (Ohaus 1922, 1934b, Blackwelder 1944, Machatschke 1972, Restrepo et al. 2003, Soula 2006, Krajcik 2008, López-García et al. 2015).

#### Types.

Soula (2006) stated that 1 ♂ lectotype existed. This is probably at ZMHB.

### Pelidnota chimborazoensis

Taxon classificationAnimaliaColeopteraScarabaeidae

Soula, 2009

Pelidnota chimborazoensis Soula, 2009: 32, 87 [original combination].

#### Distribution.

ECUADOR: Bolívar, Chimborazo (Soula 2009).

#### Types.

The holotype ♂ of *Pelidnota chimborazoensis* is at MNHN. The following specimens are deposited at CCECL. 4 ♂ paratypes, 3 ♀ paratypes: “Equateur La Chima M.de Mathan 1^er^ Semestre 1893//Muséum Paris Coll. R. Öberthür//Paratype 2008 *Pelidnota chimborazoensis* S. Soula det.” (47030454); “Chimbo Equateur M.de Mathan 1897//Paratype 2008 *Pelidnota chimborazoensis* S. Soula det.” (47030455); Two paratypes with identical label data “Equateur Chimbo M.de Mathan 1^er^ Semestre 1892//Paratype 2008 *Pelidnota chimborazoensis* S. Soula det.” (47030456 and 47030457); “La Chima (Equateur) IV 93 coll. – SOULA//Paratype 2008 *Pelidnota chimborazoensis* S. Soula det.” (47030458); “Equateur//Paratype 2008 *Pelidnota chimborazoensis* S. Soula” (47030459); “Balzar mountains Ecuador. Illingworth 1879//Ex-Musæo D.Sharp 1890//Paratype 2009 *Pelidnota chimborazoensis* S. Soula” (47030460). Genitalia card-mounted underneath four male and one female paratypes. Box 4618664 SOULA.

#### Remarks.

“Chimbo 1897” is recorded as 1891 in the original description (Soula 2009). The specimen labeled from “Balzar mountains” had “Illingworth” omitted from the description (Soula 2009).

### Pelidnota chiriquicola

Taxon classificationAnimaliaColeopteraScarabaeidae

Ohaus, 1913

Pelidnota laevissima chiriquicola Ohaus, 1913: 499 [original combination].
Pelidnota (Pelidnota) laevissima chiriquicola Ohaus [new subgeneric combination by Ohaus 1918: 23].
Pelidnota chiriquicola Ohaus [removal of subgeneric classification and new species status by Soula 2009: 102–103].

#### Distribution.

COSTA RICA: Puntarenas (Hardy 1975; Solís and Morón 1994; Soula 2009). PANAMA: Chiriquí (Ohaus 1913, 1918, 1934b, Blackwelder 1944, Machatschke 1972, Hardy 1975, Ratcliffe 2002, Krajcik 2008, Soula 2009).

#### Remarks.

Krajcik (2012, 2013) considered *P. chiriquicola* to be a subspecies of *P. laevissima*.

### Pelidnota chiriquina

Taxon classificationAnimaliaColeopteraScarabaeidae

F. Bates, 1904

Pelidnota chiriquina F. Bates, 1904: 257, 265–266 [original combination].
Pelidnota (Pelidnota) chiriquina F. Bates [new subgeneric combination by Ohaus 1918: 23].
Pelidnota chiriquina F. Bates [removal of subgeneric classification by Soula 2009: 53–54].

#### Distribution.

COLOMBIA: Chocó (Neita-Moreno et al. 2006, Neita-Moreno 2011). COSTA RICA: Puntarenas (Hardy 1975, Solís and Morón 1994, Soula 2009). PANAMA: Chiriquí (F. Bates 1904, Ohaus 1918, 1934b, Blackwelder 1944, Machatschke 1972, Hardy 1975, Ratcliffe 2002, Krajcik 2008, Soula 2009).

#### Types.

1 ♂ lectotype of *Pelidnota chiriquina* at BMNH (Hardy 1975, Soula 2009).

### Pelidnota chlorana

Taxon classificationAnimaliaColeopteraScarabaeidae

Erichson, 1847

Pelidnota chlorana Erichson, 1847: 99 [original combination].
Pelidnota (Pelidnota) chlorana Erichson [new subgeneric combination by Ohaus 1918: 23].
Pelidnota chlorana Erichson [removal of subgeneric classification by Soula 2009: 98].

#### Distribution.

BOLIVIA: La Paz, Santa Cruz (Blackwelder 1944, Ohaus 1918, 1934b, 1952, Machatschke 1972). BRAZIL: Amazonas (Ohaus 1952, Machatschke 1972). COLOMBIA: Boyacá, Tolima (Ohaus 1952, Machatschke 1972). ECUADOR: Morona-Santiago, Napo, Sucumbíos, Zamora-Chinchipe (Blackwelder 1944, Ohaus 1918, 1934b, 1952, Machatschke 1972, Paucar-Cabrera 2005). PERU: Junín, San Martín (Blackwelder 1944, Ohaus 1918, 1952, Machatschke 1972, Krajcik 2008, Soula 2009, Ratcliffe et al. 2015).

#### Types.

1 ♀ syntype of *Pelidnota chlorana* at ZMHB (Soula 2009).

### Pelidnota costaricensis

Taxon classificationAnimaliaColeopteraScarabaeidae

H. W. Bates, 1888

Pelidnota costaricensis H. W. Bates, 1888: 274 [original combination].
Pelidnota (Pelidnota) costaricensis H. W. Bates [new subgeneric combination by Ohaus 1918: 23].
Pelidnota costaricensis H. W. Bates [removal of subgeneric classification by Soula 2009: 68–69].

#### Distribution.

COSTA RICA: Alajuela, Guanacaste, Heredia, Puntarenas, San José (H. W. Bates 1888, Ohaus 1918, 1934b, Blackwelder 1944, Machatschke 1972, Hardy 1975, Solís and Morón 1994, Hilje 1996, Krajcik 2008, Soula 2009). PANAMA (Hardy 1975, Soula 2009).

#### Types.

1 ♀ lectotype of *Pelidnota costaricensis* at BMNH (Hardy 1975, Soula 2009); 4 paralectotypes at BMNH (Soula 2009); 4 paralectotypes at MNHN (Soula 2009).

### Pelidnota courtini

Taxon classificationAnimaliaColeopteraScarabaeidae

Soula, 2009

Pelidnota courtini Soula, 2009: 111–112 [original combination].

#### Distribution.

BRAZIL: Bahia, Minas Gerais (Soula 2009).

#### Types.

The following specimens are deposited at CCECL. 1 ♂ holotype, 1 ♀ allotype, 1 ♂ paratype, 4 ♀ paratypes: “Facenda Baxinha - 450 m Amargosa - Bahia - Brésil 28. III.89 COLL - B. COURTIN//Holotype 2008 *Pelidnota courtini* S. Soula” (47030779); “Facenda Baxinha - 450 m Amargosa (Bahia - Brésil) 15. III.89 - B. Courtin//Allotype 2008 *Pelidnota courtini* S. Soula” (47030780); “Pelichnota (sic) palidipennis Bahia Brésil Amargosa - 15. III-89 COLL - B. COURTIN//Paratype 2008 *Pelidnota courtini* S. Soula” (47030781); “Pelichnota (sic) palidipennis Bahia Brésil Amargosa - 15. III-89 COLL - B. COURTIN//Paratype 2008 *Pelidnota courtini* S. Soula” (47030781); “Pelichnota (sic) palidipennis Facenda Baxinha - 400m Amargosa (Bahia - Brésil) 15-III-89 - B. Courtin//Paratype 2008 *Pelidnota courtini* S. Soula” (47030782); “Facenda Baxinha - 450 m Amargosa - Bahia - Brésil 15. III.89 COLL - B. COURTIN//Paratype 2008 *Pelidnota courtini* S. Soula” (47030783); “Facenda Baxinha Amargosa - 400 m Bahia - Brésil 15. III.89 B. Courtin//Paratype 2008 *Pelidnota courtini* S. Soula” (47030784); “MUSEUM PARIS BRÉSIL BAHIA P. SERRE 1913//Ohaus determ. *Pelidnota palidipeñis* F. Bates//Paratype 2009 *Pelidnota courtini* S. Soula” (47030785). Genitalia card-mounted underneath the male holotype and the male paratype. Box 4618680 SOULA. The following specimens are deposited at CMNC. 1 ♂ paratype “BRASIL Eo de BAHIA Santa Ana Bondar-coleg. Coll. Martínez Jul.-927// H. & A. HOWDEN COLLECTION *ex.* A. Martinez coll.//Paratype 2008 *Pelidnota courtini* S. Soula”.

### Pelidnota crassipes

Taxon classificationAnimaliaColeopteraScarabaeidae

Ohaus, 1905

Pelidnota crassipes Ohaus, 1905: 319 [original combination].
Pelidnota (Ganonota) crassipes Ohaus [new subgeneric combination by Ohaus 1918: 28].
Pelidnota (Strigidia) crassipes Ohaus [new subgeneric combination by Machatschke 1970: 157].
Pelidnota (Odontognathus) crassipes Ohaus [new subgeneric combination by Hardy 1975: 4].
Pelidnota (Ganonota) crassipes Ohaus [revised subgeneric combination by Frey 1976: 345].
Strigidia crassipes (Ohaus) [new combination by Soula 2006: 21].
Pelidnota (Strigidia) crassipes Ohaus [revised combination and revised subgeneric combination by Özdikmen 2009: 145].
Pelidnota crassipes Ohaus [removal of subgeneric classification by Soula 2009: 115].

#### Distribution.

ARGENTINA: Misiones (Ohaus 1905, 1918, 1934b, Blackwelder 1944, Machatschke 1972, Soula 2006, Krajcik 2008). BOLIVIA (Ohaus 1918, 1934b, Blackwelder 1944). BRAZIL: Minas Gerais, Goiás, Mato Grosso (Soula 2006). PARAGUAY: Asunción (Ohaus 1905, 1918, 1934b, Blackwelder 1944, Machatschke 1972, Soula 2006).

#### Types.

**Figure 58.**
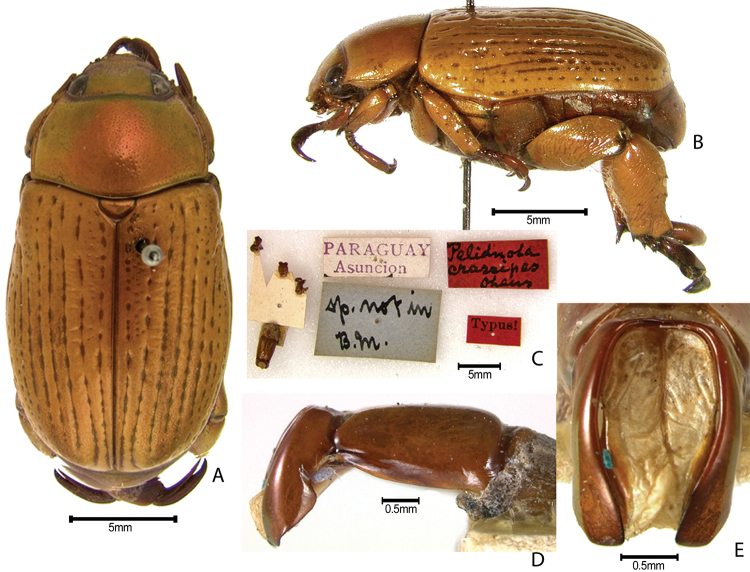
*Pelidnota crassipes* Ohaus type male (see “*Type specimens and lectotype designation*” in Methods) from ZMHB. **A** Dorsal habitus **B** Lateral habitus **C** Specimen labels, mouthparts, and male genitalia **D** Male genitalia, lateral view **E** Male parameres, caudal view.

1 ♂ lectotype and 2 paralectotypes of *Pelidnota crassipes* at ZMHB (Soula 2006) (Fig. 58).

### Pelidnota cribrata

Taxon classificationAnimaliaColeopteraScarabaeidae

(Ohaus, 1913)

Heteropelidnota cribrata Ohaus, 1913: 506 [original combination].
Pelidnota cribrata (Ohaus) [new combination by Soula 2008: 16].

#### Distribution.

BRAZIL: Amazonas (INPA), Rondônia (INPA), Pará (Ohaus 1913, 1918, 1934b, Machatschke 1972, Soula 2008). COLOMBIA: Quindío (Restrepo et al. 2003). FRENCH GUIANA: Cayenne, St.-Laurent du Maroni (Ohaus 1913, 1918, 1934b, Machatschke 1972, Krajcik 2008).

#### Types.

1 ♂ lectotype of *Heteropelidnota cribrata* from Para, Brazil at ZHMB (Soula 2008). 2 paralectotypes of *H. cribrata* are also paratypes of *P. touroulti* Soula.

#### Remarks.

According to Soula (2008), the type series of *H. cribrata* included two, distinct species: the nominate species (*H. cribrata*) and a cryptic species that Soula gave the name *P. touroulti*. In his redescription of *P. cribrata*, Soula incorrectly provided an image of the male parameres of *P. ustarani* (Soula 2008: 16). The image provided appears to be directly from Martínez’s description of *P. ustarani* (Martínez 1967). Then, in Soula’s comparison of *P. cribrata* with *P. touroulti* and *P. werneri*, Soula provided a different image of the male parameres of *P. cribrata*. The form of the parameres in this image (Soula 2008: 38, image on left) is apparently the form that is associated with the lectotype of *P. cribrata*. Soula assigned Ohaus’ two paralectotypes of *H. cribrata* with the new species *P. touroulti* (Soula 2008: 38, image in middle).

### Pelidnota cuprea

Taxon classificationAnimaliaColeopteraScarabaeidae

(Germar, 1824)

Rutela cuprea Germar, 1824: 120–121 [original combination].
Pelidnota cuprea (Germar) [new combination by Perty 1830: 49].
Strigidia cuprea (Germar) [new combination by Burmeister 1844: 389].
Odontognathus cupreus (Germar) [new combination by Blanchard 1851: 214–215].
Strigidia cuprea (Germar) [new combination by Lacordaire 1856: 355].
Odontognathus cupreus (Germar) [revised combination by Harold 1869b: 1221].
Pelidnota (Odontognathus) cuprea (Germar) [revised combination and new subgeneric combination by Ohaus 1913: 504].
Pelidnota (Ganonota) cuprea (Germar) [new subgeneric combination by Ohaus 1918: 27].
Pelidnota (Strigidia) cuprea (Germar) [new subgeneric combination by Machatschke 1970: 157].
Pelidnota (Odontognathus) cuprea (Germar) [revised subgeneric combination by Hardy 1975: 4].
Pelidnota (Ganonota) cuprea (Germar) [revised subgeneric combination by Frey 1976: 344].
Strigidia cuprea (Germar) [revised combination by Soula 2006: 13–16].
Pelidnota (Strigidia) cuprea (Germar) [revised combination and revised subgeneric combination by Özdikmen 2009: 145].
Pelidnota cuprea (Germar) [removal of subgeneric classification by Soula 2009: 115].
Odontognathus unicolor Laporte, 1840 **synonym.**
Odontognathus unicolor Laporte, 1840: 137 [original combination].
Strigidia fulvipennis (Germar) [syn. by Burmeister 1844: 390].
Odontognathus fulvipennis var. unicolor Germar [revised combination and new infrasubspecific status by Harold 1869b: 1221].
Pelidnota (Ganonota) cuprea (Germar) [syn. by Ohaus 1918: 27].
Rutela fulvipennis Germar, 1824 **synonym.**
Rutela fulvipennis Germar, 1824: 121 [original combination].
Strigidia fulvipennis (Germar) [new combination by Burmeister 1844: 390].
Odontognathus cupreus var. fulvipennis (Germar) [new combination and new infrasubspecific status by Blanchard 1851: 215].
Pelidnota (Odontognathus) cuprea var. fulvipennis (Germar) [new combination and new subgeneric combination by Ohaus 1913: 504].
Pelidnota (Ganonota) cuprea var. fulvipennis (Germar) [new subgeneric combination by Ohaus 1918: 27].
Pelidnota (Strigidia) cuprea var. fulvipennis (Germar) [new subgeneric combination by Machatschke 1970: 157].
Pelidnota (Strigidia) cuprea forma fulvipennis (Germar) [revised infrasubspecific status by Machatschke 1972: 29].
Pelidnota (Odontognathus) cuprea forma fulvipennis (Germar) [revised subgeneric combination by Hardy 1975: 4].
Pelidnota (Ganonota) cuprea forma fulvipennis (Germar) [revised subgeneric combination by Frey 1976: 344].
Strigidia cuprea var. fulvipennis (Germar) [revised combination and revised infrasubspecific status by Soula 2006: 15].
Pelidnota (Strigidia) cuprea var. fulvipennis (Germar) [revised combination and revised subgeneric combination by Özdikmen 2009: 145].
Pelidnota cuprea var. fulvipennis (Germar) [removal of subgeneric classification by Soula 2009: 115].
Pelidnota cuprea (Germar) [**syn. n.**].

#### Distribution.

ARGENTINA (Soula 2006). BOLIVIA (Soula 2006). BRAZIL: Bahia, Goiás, Rio de Janeiro, Rio Grande do Sul, Santa Catarina (Germar 1824, Perty 1830, Burmeister 1844, Blanchard 1851, Ohaus 1913, 1918, 1934b, Blackwelder 1944, Machatschke 1972, Soula 2006, Krajcik 2008). PARAGUAY (Ohaus 1913, 1918, 1934b, Machatschke 1972, Soula 2006).

#### Remarks.

Color variation in this species is found within populations. At least 80 specimens were collected in a single collecting event and single collecting locality (Rio Je Janeiro, Brazil). From this collecting event, Ohaus’s determinations refer to P. cuprea var. cuprea (blackish and shiny cupreous reflections), P. cuprea var. coerulea (black with shiny green reflections), and P. cuprea var. fulvipennis (castaneous with shiny green reflections). Research should examine if this variation is intraspecific or, instead, indicative of interspecific variation in several sympatric species. Relationships of the species in the *Pelidnota cuprea* complex require analysis. Species in the group have bounced to and from the genera *Pelidnota, Odontognathus, Ganonota*, and *Strigidia*, demonstrating historical classification difficulties and illustrating the need for phylogenetic analysis within the broader context of the Rutelini. Male species in the “cuprea complex” (*P. rubripennis, P. riedeli, P. cuprea, P. ebenina*) share a concavity on the disc of the sternites.

**Figure 59.**
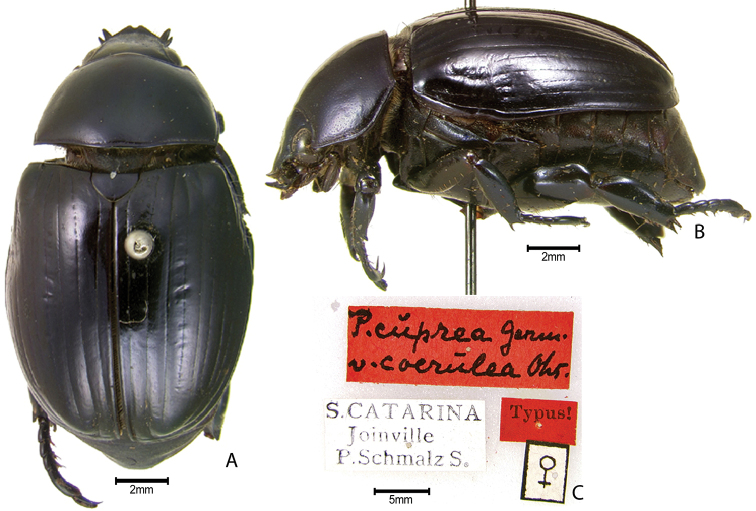
Pelidnota cuprea var. coerulea Ohaus (unavailable name) (valid name *Pelidnota cuprea* [Germar]) invalid type female from ZMHB. **A** Dorsal habitus **B** Lateral habitus **C** Specimen labels.

**Figure 60.**
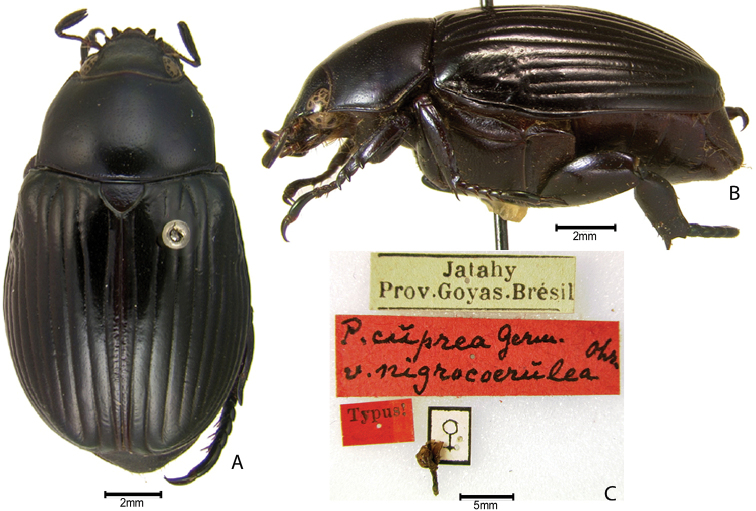
Pelidnota cuprea var. nigrocoerulea Ohaus (unavailable name) (valid name *Pelidnota cuprea* [Germar]) invalid type female from ZMHB. **A** Dorsal habitus **B** Lateral habitus **C** Specimen labels.

**Figure 61.**
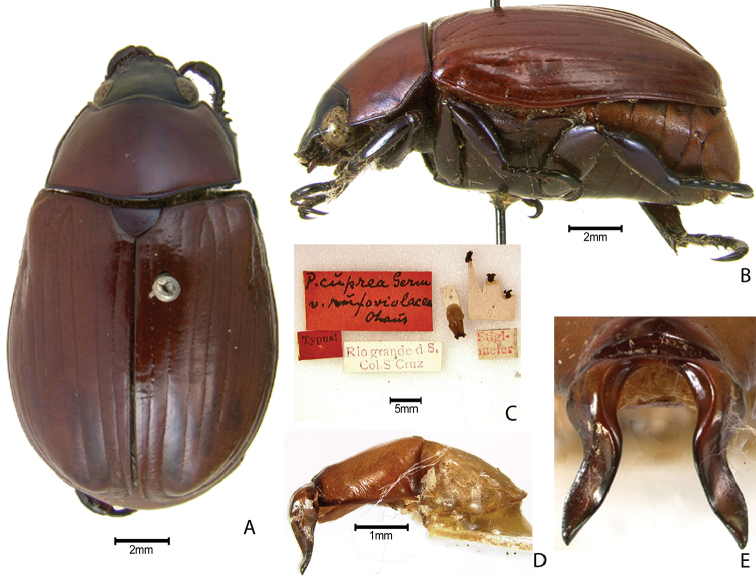
Pelidnota cuprea var. rufoviolacea Ohaus (unavailable name) (valid name *Pelidnota cuprea* [Germar]) invalid type male from ZMHB. **A** Dorsal habitus **B** Lateral habitus **C** Specimen labels, mouthparts, and male genitalia **D** Male genitalia, lateral view **E** Male parameres, caudal view.

Ohaus’s (1913) publication described both subspecies and varieties (sometimes both for the same species, e.g., *Homonyx chalceus*), thus unambiguously allowing us to treat these names in an infrasubspecific manner. Ohaus (1913) named several varieties of *Pelidnota cuprea*, but these names are unambiguously infrasubspecific and are **unavailable** according to ICZN Article 45.6.1: Pelidnota (Odontognathus) cuprea var. coerulea Ohaus (**unavailable name**) (Fig. 59), P. (Odontognathus) cuprea var. rufoviolacea Ohaus (**unavailable name**) (Fig. 61), and P. (Odontognathus) cuprea var. nigrocoerulea (**unavailable name**) (Fig. 60) (Moore and Jameson 2013). *Rutela fulvipennis* Germar is an available name that was subsequently treated as an infrasubspecific color variant of *Pelidnota cuprea*. *Rutela fulvipennis* maintained infrasubspecific status through Soula (2006). Krajcik (2008) listed the name in synonymy with *Pelidnota cuprea* (Germar). Because we do not consider Krajcik (2008) to contain express taxonomic changes and we do not recognize infrasubspecific entities, we formalize the synonymy herein: *Rutela fulvipennis* Germar is a **new synonym** of *Pelidnota cuprea* (Germar).

### Pelidnota cupripes cupripes

Taxon classificationAnimaliaColeopteraScarabaeidae

Perty, 1830

Pelidnota cupripes Perty, 1830: 48 [original combination].
Pelidnota (Ganonota) cupripes Perty [new subgeneric combination by Ohaus 1918: 25].
Pelidnota (Strigidia) cupripes Perty [new subgeneric combination by Machatschke 1970: 157].
Pelidnota (Odontognathus) cupripes Perty [new subgeneric combination by Hardy 1975: 4].
Strigidia cupripes (Perty) [new combination by Soula 2006: 52–53].
Pelidnota (Strigidia) cupripes Perty [revised combination and revised subgeneric combination by Özdikmen 2009: 145].
Pelidnota cupripes Perty [removal of subgeneric classification by Soula 2009: 115].

#### Distribution.

BRAZIL: Espírito Santo, Rio de Janeiro, São Paulo, Santa Catarina (Perty 1830, Burmeister 1844, Blanchard 1851, Ohaus 1908a, 1918, 1934b, Blackwelder 1944, Machatschke 1972, Soula 2006, Krajcik 2008).

### Pelidnota cupripes goyasensis

Taxon classificationAnimaliaColeopteraScarabaeidae

(Soula, 2006)

Strigidia cupripes goyasensis Soula, 2006: 53 [original combination].
Pelidnota (Strigidia) cupripes goyasensis (Soula) [new combination and new subgeneric combination by Özdikmen 2009: 145].
Pelidnota cupripes goyasensis (Soula) [removal of subgeneric classification by Soula 2009: 115].

#### Distribution.

BRAZIL: Goiás (Soula 2006).

#### Types.

The following specimen is deposited at CCECL. 1 ♂ holotype: “Goyaz, Bresil//*Pelidnota viridana*//MUSÉUM PARIS 1930 COLL SICARD//Holotype 2005 *Strigidia cupripes goyasensis* Sou. Soula det.” (47030397). Genitalia card-mounted underneath holotype. Box 4618663 SOULA.

### Pelidnota cupripes surinamensis

Taxon classificationAnimaliaColeopteraScarabaeidae

(Soula, 2006)

Strigidia cupripes surinamensis Soula, 2006: 53 [original combination].
Pelidnota (Strigidia) cupripes surinamensis (Soula) [new combination and new subgeneric combination by Özdikmen 2009: 145].
Pelidnota cupripes surinamensis (Soula) [removal of subgeneric classification by Soula 2009: 116].

#### Distribution.

SURINAME (Soula 2006).

#### Types.

The following specimens are deposited at CCECL. 1 ♀ holotype, 1 ♀ paratype: “Surinam//*Rutela glabrata*// MUSÉUM PARIS 1930 COLL SICARD//Holotype 2005 *Strigidia cupripes surinamensis* Sou. Soula det.” (47030398); “Surinam//*Rutela glabrata*//MUSÉUM PARIS 1930 COLL SICARD//Paratype 2005 *Strigidia cupripes surinamensis* Sou. Soula det.” (47030399). Box 4618663 SOULA.

### Pelidnota cyanipes

Taxon classificationAnimaliaColeopteraScarabaeidae

(Kirby, 1819)

Rutela cyanipes Kirby, 1819: 406–407 [original combination].
Pelidnota cyanipes (Kirby) [new combination by Laporte 1840: 122].
Pelidnota (Chalcoplethis) cyanipes (Kirby) [new subgeneric combination by Ohaus 1918: 29].
Pelidnota cyanipes (Kirby) [removal of subgeneric classification by Soula 2009: 35–36].

#### Distribution.

ARGENTINA: Misiones (Gutiérrez 1951, Soula 2009). BRAZIL: Bahia, Pará, Rio de Janeiro, Rio Grande do Sul (Laporte 1840, Burmeister 1844, 1855, Blanchard 1851, Ohaus 1908a, 1918, 1934b, Blackwelder 1944, Machatschke 1972, Krajcik 2008, Soula 2009), Santa Catarina, Mato Grosso (WBWC).

#### Types.

Most of Kirby’s type specimens are located at BMNH. A search for the type specimen of *P. cyanipes* did not locate the specimen in the collection.

### Pelidnota cyanitarsis

Taxon classificationAnimaliaColeopteraScarabaeidae

(Gory, 1833)

Rutela cyanitarsis Gory, 1833a: 67–68 [original combination].
Pelidnota cyanitarsis (Gory) [new combination by Burmeister 1844: 407].
Pelidnota (Pelidnota) cyanitarsis (Gory) [new subgeneric combination by Ohaus 1918: 25].
Pelidnota cyanitarsis (Gory) [removal of subgeneric classification by Soula 2009: 40–41].
Rutela nitidissima Guérin-Méneville, 1834 **synonym.**
Rutela nitidissima Guérin-Méneville, 1834: 91 [original combination].
Pelidnota (Pelidnota) cyanitarsis (Gory) [syn. by Ohaus 1934b: 81].

#### Distribution.

BRAZIL: Bahia, Minas Gerais, Pará (Guérin-Méneville 1834, Burmeister 1844, Blanchard 1851, Ohaus 1918, 1934b, Blackwelder 1944, Machatschke 1972, Krajcik 2008, Soula 2009).

#### Remarks.

Two spectacular species, *P. cyanitarsis* and *P. sumptuosa* Vigors, have been confused in collections and the literature. Both species are brilliant metallic blue, green, or blue-green with enlarged metatibia. Several characters serve to separate these species (*P. cyanitarsis* with well-developed fovea on pronotal margin whereas *P. sumptuosa* has a strigate patch on the pronotal margins; *P. cyanitarsis* male with well developed medial tooth on foreclaw and *P. sumptuosa* male with well-developed subapical tooth on foreclaw), and male parameres are also diagnostic (see Soula 2009: 41 and 42).

### Pelidnota discicollis

Taxon classificationAnimaliaColeopteraScarabaeidae

Ohaus, 1912

Pelidnota discicollis Ohaus, 1912: 303 [original combination].
Pelidnota (Ganonota) discicollis Ohaus [new subgeneric combination by Ohaus 1918: 25].
Pelidnota (Strigidia) discicollis Ohaus [new subgeneric combination by Machatschke 1970: 157].
Pelidnota (Odontognathus) discicollis Ohaus [new subgeneric combination by Hardy 1975: 4].
Strigidia discicollis (Ohaus) [new combination by Soula 2006: 54].
Pelidnota (Strigidia) discicollis Ohaus [revised combination and revised subgeneric combination by Özdikmen 2009: 145].
Pelidnota discicollis Ohaus [removal of subgeneric classification by Soula 2009: 115].

#### Distribution.

BRAZIL: Pará (Ohaus 1912, 1918, 1934b, Blackwelder 1944, Soula 2006, Krajcik 2008). VENEZUELA: Bolivar (MIZA)

#### Types.

**Figure 62.**
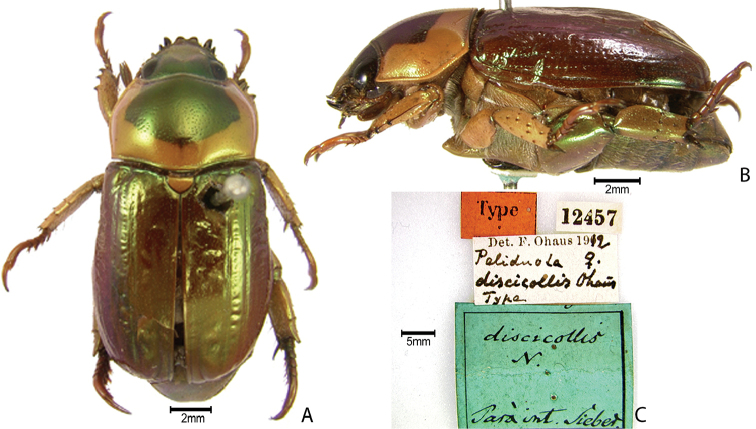
*Pelidnota discicollis* Ohaus holotype female from ZMHB. **A** Dorsal habitus **B** Lateral habitus **C** Specimen labels.

1 ♀ holotype specimen of *Pelidnota discicollis* Ohaus at ZMHB (Fig. 62).

#### Remarks.

CCECL contains a specimen of *P. discicollis* labeled as a male alloréférent with the following data: 1 ♂ alloréférent: “Para (Brésil) de Mathan//Alloréférent de *Strigidia discicollis* Oh. M. SOULA det 19 2006” (47030430). Genitalia card-mounted underneath the alloréférent. Box 4618663 SOULA. The male specimen from Venezuela represents a new country record.

### Pelidnota dobleri

Taxon classificationAnimaliaColeopteraScarabaeidae

Frey, 1967

Pelidnota dobleri Frey, 1967: 375–376 [original combination].
Pelidnota (Pelidnota) doblerae Frey [new subgeneric combination and incorrect subsequent spelling by Machatschke 1972: 22].
Strigidia doblerae (Frey) [new combination by Soula 2006: 19–20].
Pelidnota doblerae Frey [revised combination by Soula 2009: 115].

#### Distribution.

BOLIVIA (Soula 2006). BRAZIL: Mato Grosso (Soula 2006). PERU: Madre de Dios (Frey 1967, Machatschke 1972, Soula 2006, Krajcik 2008, Ratcliffe et al. 2015).

#### Types.

1 ♂ holotype of *Pelidnota dobleri* at NHMB (Soula 2006). 1 ♂ paratype at CMNC.

### Pelidnota drumonti

Taxon classificationAnimaliaColeopteraScarabaeidae

Soula, 2009

Pelidnota drumonti Soula, 2009: 34, 113–114 [original combination].

#### Distribution.

BRAZIL: São Paulo (Soula 2009).

#### Types.

The following specimens are deposited at CCECL. 1 ♂ holotype, 1 ♀ allotype, 12 ♂ paratypes, 5 ♀ paratypes: “Fazenda Rhodia Paulinia, São Paulo 19/01/92//Holotype 2008 *Pelidnota drumonti* S.Soula” (47030762); “Fazenda Rhodia Paulinia, São Paulo 19/01/92/Allotype 2008 *Pelidnota drumonti* S.Soula” (47030763); Six paratypes with identical label data: “Fazenda Rhodia Paulinia, São Paulo 19/01/92/Paratype 2008 *Pelidnota drumonti* S. Soula” (47030764 to 47030769); Nine paratypes with identical label data: “Fazenda Rhodia Paulinia, São Paulo 19/01/92/Paratype *Pelidnota drumonti* S. 2008-2009” (47030770 to 47030778). Genitalia card-mounted underneath the male holotype and two male paratypes. Box 4618680 SOULA. The following specimens are deposited at CMNC: 26 ♂ paratypes, 27 ♀ paratypes.

### Pelidnota dubia

Taxon classificationAnimaliaColeopteraScarabaeidae

F. Bates, 1904

Pelidnota dubia F. Bates, 1904: 254, 262–263 [original combination].
Pelidnota (Ganonota) dubia F. Bates [new subgeneric combination by Ohaus 1918: 25].
Pelidnota (Strigidia) dubia F. Bates [new subgeneric combination by Machatschke 1970: 157].
Pelidnota (Odontognathus) dubia F. Bates [new subgeneric combination by Hardy 1975: 4].
Strigidia dubia (F. Bates) [new combination by Soula 2006: 77].
Pelidnota (Strigidia) dubia F. Bates [revised combination and revised subgeneric combination by Özdikmen 2009: 145].
Pelidnota dubia F. Bates [removal of subgeneric classification by Soula 2009: 115].

#### Distribution.

COLOMBIA: Caldas, Cauca (F. Bates 1904, Ohaus 1918, 1934b, Blackwelder 1944, Machatschke 1972, Restrepo et al. 2003, Soula 2006, Krajcik 2008).

#### Types.

**Figure 63.**
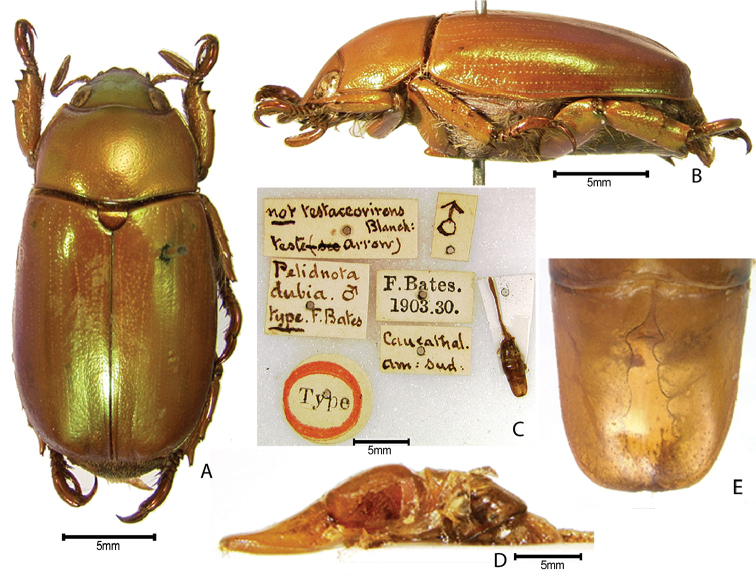
*Pelidnota dubia* F. Bates syntype male from BMNH. **A** Dorsal habitus **B** Lateral habitus **C** Specimen labels and male genitalia **D** Male genitalia, lateral view **E** Male parameres, dorsal view.

1 ♂ lectotype specimen of *Pelidnota dubia* F. Bates at BMNH (Fig. 63) and 1 paralectotype specimen at BMNH.

#### Remarks.

F. Bates (1904) may have named this species “dubia” because of the overall similarity with *P. testaceovirens*. He stated that *P. dubia* may be conspecific with *P. testaceovirens* (F. Bates 1904: 263), but he hypothesized that *P. dubia* was distinct from *P. testaceovirens* based on differences in size (“23-24 mm” for *P. testaceovirens* versus “19.5-22 mm” for *P. dubia*) as well as form of the pygidium (densely striate and glabrous in *P. testaceovirens* versus “aciculate-rugulose” and with “long, grayish hairs” in *P. dubia*). He noted that some specimens had “slight green reflections” (F. Bates 1904: 263).

### Pelidnota durantonorum

Taxon classificationAnimaliaColeopteraScarabaeidae

Soula, 2009

Pelidnota durantonorum Soula, 2009: 33, 106–107 [original combination].

#### Distribution.

FRENCH GUIANA: Iracoubo, Kourou (Soula 2009, 2010c).

#### Types.

The following specimens are deposited at CCECL. 1 ♂ holotype, 1 ♀ allotype, 34 ♂ paratypes, 25 ♀ paratypes, 1 ♂ invalid paratype: “Patagaïe G. F. 08/2001 M. SOULA det 19//Holotype 2008 *Pelidnota durantonorum* S. Soula” (47030671); “Patagaïe (G. F.) 08/2001 M. SOULA det 19//Allotype 2008 *Pelidnota durantonorum* S. Soula” (47030672); Three paratypes with identical label data: “GUYANE FRANÇAISE Piste de Nancibo pK 1,5 11-VIII-1996 H. de Toulgoët & J. Navatte réc.//Paratype *Pelidnota durantonorum* S. 2008-2009” (47030673 and 47030674, exch38); “GUYANE FRANÇAISE Piste de Kaw pK 39 8-VII-1996 H. de Toulgoët & J. Navatte réc.//Paratype *Pelidnota durantonorum* S. 2008-2009” (47030675); “GUYANE FRANÇAISE Route de Coralie pK 2,2 15-VII-1996 H. de Toulgoët & J. Navatte réc.//Paratype *Pelidnota durantonorum* S. 2008-2009” (47030676); Two male paratypes with identical label data: “GUYANE FRANÇAISE Route de Régina pK 79 18-VII-1996 H. de Toulgoët & J. Navatte réc.//Paratype *Pelidnota durantonorum* S. 2008-2009” (47030677 and 47030678); “GUYANE FRANÇAISE Piste de Kaw pK 36 6-VIII-1996 H. de Toulgoët & J. Navatte réc.//Paratype *Pelidnota durantonorum* S. 2008-2009” (47030679); Three paratypes with identical label data: “Guyane fr. (Est) M. SOULA det 19//Paratype *Pelidnota durantonorum* S. 2008-2009” (47030680, 47030683, exch39); Two paratypes with identical label data: “Patagaïe Guyane fr. M. SOULA det 19 [obverse] VIII 2001//Paratype *Pelidnota durantonorum* S. 2008-2009” (47030681 and 47030682); “Guyane fr. Bélizon M. SOULA det 19//Paratype *Pelidnota durantonorum* S. 2008-2009” (47030684); “Nancibo PK 6 P.L. 20/7/85//Paratype *Pelidnota durantonorum* S. 2008-2009” (47030685); “K [Kaw] - PK 40 25/8/84 P.L. //Paratype *Pelidnota durantonorum* S. 2008-2009” (47030686); “Mgne de Kaw G. F. 8/92//Paratype *Pelidnota durantonorum* S. 2008-2009” (47030687); “Piste de Kaw 8/92//Paratype *Pelidnota durantonorum* S. 2008-2009” (47030688); “Kaw 7/87//Paratype *Pelidnota durantonorum* S. 2008-2009” (47030689); Two paratypes with identical label data: “KAW PK 34 29/8/84 [obverse] P.L.//Paratype *Pelidnota durantonorum* S. 2008-2009” (47030690, exch40); “KAW PK 34 28/7/84//Paratype *Pelidnota durantonorum* S. 2008-2009” (47030691); Two paratypes with identical label data: “Rocoucova P.L. PK4 23/7/84//Paratype *Pelidnota durantonorum* S. 2008-2009” (47030692 and 47030694); “Rocoucova PK4 23/7/84 P.L.//Paratype *Pelidnota durantonorum* S. 2008-2009” (47030693); Five paratypes with identical label data: “Rocoucova 26/6/85 P.L.//Paratype *Pelidnota durantonorum* S. 2008-2009” (47030695 to 47030698, exch41); “S^t^ Jean Laurent du Maroni 1980 - 82//Paratype *Pelidnota durantonorum* S. 2008-2009” (47030699); “Coll. BLEUZEN M^gne^ de KAW PK 10 Guyane Fr. 12 Juillet 1983//Paratype *Pelidnota durantonorum* S. 2008-2009” (47030700); “St Georges VIII/87//Paratype *Pelidnota durantonorum* S. 2008-2009” (47030701); “Saül M^t^ la Fumée G.F.//Paratype *Pelidnota durantonorum* S. 2008-2009” (47030702); “Saül Mt la Fumée G.F. 08/92//Paratype *Pelidnota durantonorum* S. 2008-2009” (47030703); “Rio Juruti Obidos (Para) coll. – SOULA//Paratype *Pelidnota durantonorum* S. 2008-2009” (47030704); “Saül G.F. 8/92//Paratype *Pelidnota durantonorum* S. 2008-2009” (exch42); “Les 2 flots G.G. VIII/01 M. SOULA det 2002//Paratype *Pelidnota durantonorum* S. 2008-2009” (47030705); “Kaw, pk 37 IX/1998 M. SOULA det 19//Paratype *Pelidnota durantonorum* S. 2008-2009” (47030706); “Degrad Saramaca G.F. 7/92//Paratype *Pelidnota durantonorum* S. 2008-2009” (47030707); “*P. pallidipennis* coll. – SOULA//Guyane F. coll. – SOULA//Paratype *Pelidnota durantonorum* S. 2008-2009” (47030708); “*P. laevissima* coll. – SOULA// Maroni Guyane F. coll. – SOULA//Paratype *Pelidnota durantonorum* S. 2008-2009” (47030709); “Le Chateau Cacao G.F. M. Soula det. 20 [obverse] 5/09/2008//Paratype *Pelidnota durantonorum* S. 2008-2009” (47030710); “Région de Cacao Guyane Franç.//Paratype *Pelidnota durantonorum* S. 2008-2009” (47030711); “15-août-07 [Guyane française] RN2 ; PK 72//Paratype *Pelidnota durantonorum* S. 2008-2009” (47030712); “15 km au S de Kourou G-F 20/7/83//Paratype *Pelidnota durantonorum* S. 2008-2009” (47030713); Two female paratypes with identical label data: “NANCIBO GUYANE FR 25-26 VIII 84//Paratype *Pelidnota durantonorum* S. 2008-2009” (47030714 and 47030725); “Saint Laurent Guyane Fse VI 1984 M.Duranton Recolt//Paratype *Pelidnota durantonorum* S. 2008-2009” (47030715); “R^te^ Paul Isnard PK 19//Saint Laurent Guyane Fse VI 1984 M.Duranton Recolt//Paratype *Pelidnota durantonorum* S. 2008-2009” (47030716); Two paratypes with identical label data: “494 64//MUSEUM PARIS GUYANE FR. LA MANA MÉLINON 1864//Paratype *Pelidnota durantonorum* S. 2008-2009” (47030717 and 47030718); “Guyana Cayensis Deyr.//ZOOL. MUSEUM DK COPENHAGEN//Paratype *Pelidnota durantonorum* S. 2008-2009” (47030719); “GUYANE Française Mission M. Boulard et P. Pompanon Muséum PARIS//KOUROU FORÊT 3-7-VIII-1975//Paratype *Pelidnota durantonorum* S. 2008-2009” (47030720); “Paratype *Pelidnota durantonorum* S. 2008-2009” (exch43); “*PELIDNOTA* MADRONA 300 m. Panama 15. III.90 Coll. B. COURTIN//Paratype *Pelidnota durantonorum* S. 2008-2009//Invalid Paratype *Pelidnota durantonorum* Soula det. Moore ‘15” (47030721); “Patagaïe G. F. 08/2001 M. SOULA det 19//Paratype *Pelidnota durantonorum* S. 2008-2009” (47030722); “Kaw PK 40 25/8/84 P.L. //Paratype *Pelidnota durantonorum* S. 2008-2009” (47030723); “Piste de Kaw 8/92 G. F. coll. – SOULA//Paratype *Pelidnota durantonorum* S. 2008-2009” (47030724); “Coll. P. BLEUZEN Gonbolo [sic pro Gonfolo] Kourou Guyane Fr. 8 Août 1983//Paratype *Pelidnota durantonorum* S. 2008-2009” (47030726). Genitalia card-mounted underneath the male holotype, 27 male paratypes and the invalid male paratype. Box 4618679 SOULA.

### Pelidnota ebenina

Taxon classificationAnimaliaColeopteraScarabaeidae

(Blanchard, 1842)

Anomala ebenina Blanchard, 1842: plate 11 [original combination].
Odontognathus ebeninus (Blanchard) [new combination by Blanchard 1851: 215].
Strigidia ebenina (Blanchard) [new combination by Lacordaire 1856: 355].
Odontognathus ebeninus (Blanchard) [revised combination by Harold 1869b: 1221].
Pelidnota (Ganonota) ebenina (Blanchard) [new combination and new subgeneric combination by Ohaus 1918: 26].
Pelidnota (Strigidia) ebenina (Blanchard) [new subgeneric combination by Machatschke 1970: 157].
Pelidnota (Odontognathus) ebenina (Blanchard) [new subgeneric combination by Hardy 1975: 4].
Strigidia ebenina (Blanchard) [revised combination by Soula 2006: 16–17].
Pelidnota (Strigidia) ebenina (Blanchard) [revised combination and revised subgeneric combination by Özdikmen 2009: 145].
Pelidnota ebenina (Blanchard) [removal of subgeneric classification by Soula 2009: 115].
Odontognathus gounellei Ohaus, 1908c **synonym.**
Odontognathus gounellei Ohaus, 1908c: 307 [original combination].
Pelidnota (Ganonota) gounellei (Ohaus) [new combination and new subgeneric combination by Ohaus 1918: 26].
Pelidnota (Strigidia) gounellei (Ohaus) [new subgeneric combination by Machatschke 1970: 157].
Pelidnota (Odontognathus) gounellei (Ohaus) [new subgeneric combination by Hardy 1975: 4].
Strigidia ebenina (Blanchard) [syn. by Soula 2006: 17].
Pelidnota (Strigidia) gounellei (Ohaus) [revised subgeneric combination and revised species status by Özdikmen 2009: 145].
Pelidnota ebenina (Blanchard) [**revised synonymy**].

#### Distribution.

ARGENTINA (Soula 2006). BRAZIL: Bahia, Pará (Ohaus 1908c, 1918, 1934b, Blackwelder 1944, Machatschke 1972, Soula 2006, Krajcik 2008). BOLIVIA: Santa Cruz (Blanchard 1851, Ohaus 1918, 1934b, Blackwelder 1944, Machatschke 1972, Soula 2006; Krajcik 2008).

#### Types.

**Figure 64.**
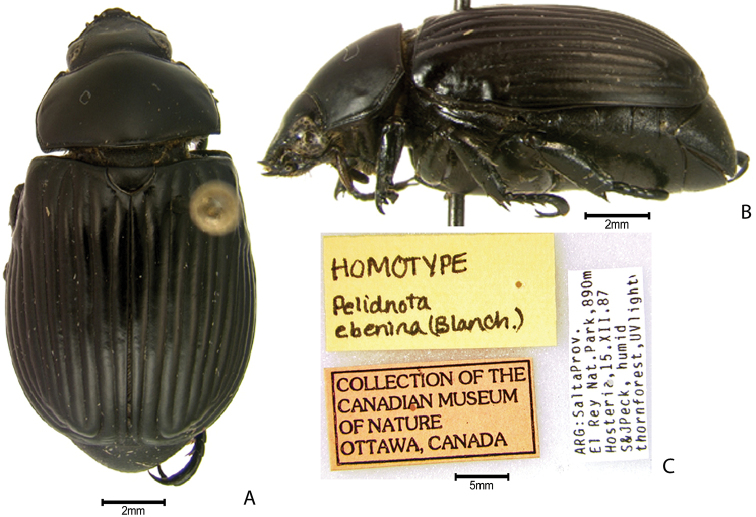
*Pelidnota ebenina* (Blanchard) female specimen compared [by Jameson] with syntype from MNHN. **A** Dorsal habitus **B** Lateral habitus **C** Specimen labels.

1 ♀ syntype (lacking head) at MNHN (Soula 2006). An exemplar specimen identified by Jameson and compared with Blanchard’s type specimen is figured (Fig. 64).

#### Remarks.

CCECL contains a *P. ebenina* specimen labeled as a male ♂ alloréférent with the following data: “Camiri [arrow] Sta Cruz 650 m coll. – SOULA/Alloreferent ♂ de *Strigidia ebenina* (Bl.) M. SOULA det. 19” (47030122). Genitalia card-mounted underneath specimen. Box 4618652 SOULA. Ohaus (1908c) described *P. gounellei* based on a male specimen from San Antonio da Barra, Bahia, Brazil. It was collected by Mr. Gounelle, to whom Ohaus dedicated the species. Ohaus (1908c) compared *P. gounellei* with *P. cuprea fulvipennis* (which he remarked was quite variable in form) and stated that *P. gounellei* is a western Brazilian variety of *P. cuprea fulvipennis*. Soula (2006) synonymized *P. gounellei* with *P. ebenina* (Fig. 64). Based on outward appearance, the two species are very similar. *Pelidnota gounellei* and *P. ebenina* differ based on type localities (*P. ebenina* in the western slopes of the Andes in Bolivia and Argentina; *P. gounellei* on Bahia and Minas Gerias in the eastern regions of Brazil). Type specimens associated with these three names (*P. ebenina, P. gounellei*, and *P. cuprea fulvipennis*) will assist in clarifying the validity of these species. Özdikmen (2009) did not acknowledge Soula (2006) and listed P. (Strigidia) gounellei (Ohaus) as a valid name. We follow Soula (2006) and consider *Odontognathus gounellei* Ohaus a **revised synonymy** of *Pelidnota ebenina* (Blanchard).

### Pelidnota egana

Taxon classificationAnimaliaColeopteraScarabaeidae

Ohaus, 1912

Pelidnota egana Ohaus, 1912: 298, 300 [original combination].
Pelidnota (Chalcoplethis) egana Ohaus [new subgeneric combination by Ohaus 1918: 28].
Strigidia egana (Ohaus) [new combination by Soula 2006: 70–71].
Pelidnota egana Ohaus [revised combination by Soula 2009: 115].

#### Distribution.

BRAZIL: Amazonas (Ohaus 1912, 1918, 1934b, Blackwelder 1944, Machatschke 1972, Soula 2006, Krajcik 2008).

### Pelidnota equatoriana

Taxon classificationAnimaliaColeopteraScarabaeidae

Soula, 2009

Pelidnota equatoriana Soula, 2009: 32, 86–87 [original combination].

#### Distribution.

ECUADOR: Esmeraldas, Napo, Pichincha, Santo Domingo (Soula 2009).

#### Types.

The following specimens are deposited at CCECL. 1 ♂ holotype, 7 ♂ paratypes, 10 ♀ paratypes: “Pacto Equateur 4/2001 M. SOULA det 19//Holotype 2007 *Pelidnota equatoriana* S. Soula” (47030439); “Pacto Equateur 4/2001 M. SOULA det 19//Paratype 2007 *Pelidnota equatoriana* S. Soula” (47030440); “Tena (E) 9/90 [0 crossed out] 1//Paratype 2007 *Pelidnota equatoriana* S. Soula” (47030441); “Tena (E) 9/91//Paratype 2007 *Pelidnota equatoriana* S. Soula” (47030442); “Celica Pichincha Eq. III/97 M. SOULA det 19//Paratype 2007 *Pelidnota equatoriana* S. Soula” (47030443); Two paratypes with identical label data “Malimpia (Esmereldas [*sic*]) Equateur M. SOULA det 19 [obverse] II/2008//Paratype 2008 *Pelidnota equatoriana* S. Soula” (47030444 and 47030445); “Lita [arrow] San Lorenzo coll. – SOULA [obverse] pk 7,5 770m 15/08/93//Paratype 2007 *Pelidnota equatoriana* S. Soula” (47030446); “Ecuador St Domingo Avril 1982//Paratype 2007 *Pelidnota equatoriana* S. Soula” (47030447); “Santo Domingo Equateur M. SOULA det 19 [obverse] V/2007//Paratype 2008 *Pelidnota equatoriana* S. Soula” (47030448); Two paratypes with identical label data “Alluriquin – 800m. VI/2000 Equateur M. SOULA det 19//Paratype 2008 *Pelidnota equatoriana* S. Soula” (47030449 and 47030450); “San José Quinindé Equateur M. SOULA det 19 [obverse] 15/4/76 (Dzido)//Paratype 2008 *Pelidnota equatoriana* S. Soula” (47030451); “Equateur M. SOULA det 19//Paratype 2007 *Pelidnota equatoriana* S. Soula” (exch24); “Equateur M. SOULA det 19//Paratype 2008 *Pelidnota equatoriana* S. Soula” (exch25);. “Quinindé Equateur XII/91 M. SOULA det 19//Paratype 2008 *Pelidnota equatoriana* S. Soula” (47030452); “P. notata coll. – SOULA” // S^to^ Domingo (Equateur) coll. – SOULA//Paratype 2007 *Pelidnota equatoriana* S. Soula” (47030453). Genitalia card-mounted underneath the male holotype and seven male paratypes. Box 4618664 SOULA. The following specimen is deposited at CMNC. 1 ♀ paratype: “ECUADOR, 700’ RioPalenque 47 km S. St. Domingo Feb 22-27 1976 H. & A. Howden//Paratype 2007 *Pelidnota equatoriana* S. Soula”.

#### Remarks.

Soula mentions an allotype in the original description but no specimen is labeled as such in CCECL. The allotype should have label data “Pacto, Pichincha, Equateur 4/2001.” These data are recorded only for the holotype and one paratype (“Pichincha” from original description). However, the number of specimens correlates with the number in the original description. Soula cites three labels with the locality “Malimpia”, but there are only two at CCECL. No paratypes were found with the locality “Palenque Howden” as cited in the original description, but there are two non-type ♀’s with locality “Rio Palenque”. The specimen labeled as “P. notata” was not included in original description.

### Pelidnota estebanabadiei

Taxon classificationAnimaliaColeopteraScarabaeidae

Soula, 2009

Pelidnota estebanabadiei Soula, 2009: 34, 112 [original combination].

#### Distribution.

BRAZIL: Rio de Janeiro (Soula 2009).

#### Types.

The following specimens are deposited at CCECL. 1 ♂ holotype, 1 ♀ allotype, 2 ♂ paratypes, 1 ♀ paratype: “Boca do Mato Cochoeiras de Macaçu-II/1995 Rio de Janeiro//Holotype 2008 *Pelidnota estebanabadiei* Soula” (47030786); “Boca do Mato Cochoeiras de Macaçu-II/1995 Rio de Janeiro//Allotype 2008 *Pelidnota estebanabadiei* Soula” (47030787); Two male paratypes with identical label data: “Boca do Mato Cochoeiras de Macaçu-II/1995 Rio de Janeiro//Paratype *Pelidnota estebanabadiei* Soula” (47030788 and 47030789); “Ex-Musæo H.W. BATES 1892//Rio J.//Paratype 2009 *Pelidnota estebanabadiei* S. Soula” (47030790). Genitalia card-mounted underneath the male holotype and the two male paratypes. Box 4618680 SOULA.

### Pelidnota estebandurani ecuatoriana

Taxon classificationAnimaliaColeopteraScarabaeidae

(Soula, 2006)

Strigidia estebandurani ecuatoriana Soula, 2006: 25 [original combination].
Pelidnota estebandurani ecuatoriana (Soula) [new combination by Soula 2009: 115].

#### Distribution.

ECUADOR: Napo (Soula 2006).

#### Types.

The following specimen is deposited at CCECL. 1 ♀ allotype: “ECUADOR NAPO SC STATION YASUNI PUCE 400m 27NOV 1995 ITapia//Allotype 2005 *Strigidia estebandurani ecuatoriana* Sou. Soula det.” (47030297). Box 4618652 SOULA.

### Pelidnota estebandurani estebandurani

Taxon classificationAnimaliaColeopteraScarabaeidae

(Soula, 2006)

Strigidia estebandurani estebandurani Soula, 2006: 12, 24–25 [original combination].
Pelidnota estebandurani estebandurani (Soula) [new combination by Soula 2009: 115].

#### Distribution.

COLOMBIA: Huila (Soula 2006).

#### Types.

The following specimens are deposited at CCECL. 1 ♂ holotype, 1 ♀ allotype, 1 ♂ paratype, 1 ♀ paratype: “Albania, Colombie 20-31/VII/1975//Holotype 2005 *Strigidia estebandurani* Sou. Soula det.” (47030293); “Albania, Colombie 20-31/VII/1975//Allotype 2005 *Strigidia estebandurani* Sou. Soula det.” (47030294); “Colombia Gigante Huila (parte alta cordillera)//Zona n˚3//Col. O. Rojas//Paratype 2005 *Strigidia estebandurani* Sou. Soula det.” (47030296); “Gazon (*sic*); 900 m 20/VII/1975 Colombie M. SOULA det 19//Paratype 2005 *Strigidia estebandurani* Soula Soula det.” (47030295). Genitalia card-mounted underneath the male holotype, female allotype, and one male paratype. Box 4618658 SOULA.

### Pelidnota fabricelavalettei

Taxon classificationAnimaliaColeopteraScarabaeidae

Soula, 2009

Pelidnota fabricelavalettei Soula, 2009: 131–132 [original combination].
Pelidnota lavalettei Soula [syn. by Soula 2011: 84].
Pelidnota fabricelavalettei Soula [**stat. rev.**].

#### Distribution.

FRENCH GUIANA (Soula 2008, 2009, 2010c).

#### Types.

The following specimen is deposited at CCECL. 1 ♂ holotype: “Guyane fr. Est. du dép. M. SOULA det. 20//Holotype 2008 *Pelidnota lavalettei* S. Soula//Holotype of *P. fabricelavalettei* Soula 2009 det. M. R. Moore 2014” (47030132). Genitalia card-mounted underneath holotype. Box 4618654 SOULA.

#### Remarks.

The same holotype specimen was described twice, resulting in a case synonymy created by Soula (2008, 2009). *Pelidnota lavalettei* Soula 2008 was considered the senior synonym and valid name, however, this name is **unavailable** per ICZN Article 16.4. (see section on unavailable names in *Pelidnota*). Because *P. fabricelavalettei* is an available name, we give it **revised status** here as a valid species. The genitalia of the holotype specimen appear to be slightly broken or deformed at the apex.

### Pelidnota filippiniae

Taxon classificationAnimaliaColeopteraScarabaeidae

Soula, 2009

Pelidnota filippiniae Soula, 2009: 108–109 [original combination].

#### Distribution.

BRAZIL: Pará (Soula 2009).

#### Types.

Holotype ♂ at MNHN (Soula 2009). The following specimens are deposited at CCECL. 5 ♂ paratypes, 3 ♀ paratypes: “Pará//Ex-Musæo H.W. BATES 1892//Paratype *Pelidnota filippiniae* Soula” (47030791); “Pará//Ex-Musæo H.W. BATES 1892//Paratype 2009 *Pelidnota filippiniae* S. Soula” (47030792); “Pará//Ex-Musæo H.W. BATES 1892//Paratype 2008 *Pelidnota filippiniae* S. Soula” (47030793); Two paratypes with identical label data: “Para//Paratype 2009 *Pelidnota filippiniae* S. Soula” (47030794 and 47030795); “Para//Paratype 2008 *Pelidnota filippiniae* S. Soula” (47030796); “Taperinha, Santarém, Pará, Brasilien 22 II 1970 S.L. Tuxen & Ove Jensen//ZOOL. MUSEUM DK COPENHAGEN//Paratype 2008 *Pelidnota filippiniae* Soula” (47030797); “San Antonio de Tauà Para - Bré M. Soula det. 20 [obverse] 15-22/X/1979//Paratype 2009 *Pelidnota filippiniae* Soula” (47030798). Genitalia card-mounted underneath four male paratypes. Box 4618681 SOULA. The following specimens are deposited at CMNC. 1 ♀ allotype: “BRASIL Eo do Para, Tucurui Alvarenga-leg. Coll. Martínez Ene.-979//H. & A. HOWDEN COLLECTION *ex.* A. Martinez coll.//Allotype 2009 *Pelidnota filippiniae* Soula”; 1 ♂ and 1 ♀ paratypes with the same labels except “Paratype” on the type label; 1 ♀ paratype: “BRASIL Belem PARA Dirings// H. & A. HOWDEN COLLECTION *ex.* A. Martinez coll.//Paratype 2009 *Pelidnota filippiniae* S. Soula”.

### Pelidnota flavovittata

Taxon classificationAnimaliaColeopteraScarabaeidae

(Perty, 1830)

Rutela flavovittata Perty, 1830: 49 [original combination].
Pelidnota liturella var. flavovittata (Perty) [new combination and new infrasubspecific status by Burmeister 1844: 397].
Pelidnota (Ganonota) liturella var. flavovittata (Perty) [new subgeneric combination by Ohaus 1918: 28].
Pelidnota (Strigidia) flavovittata (Perty) [new subgeneric combination by Machatschke 1970: 157].
Pelidnota (Odontognathus) flavovittata (Perty) [new subgeneric combination by Hardy 1975: 4].
Strigidia flavovittata (Perty) [new combination by Soula 2006: 42–43].
Pelidnota (Strigidia) flavovittata (Perty) [revised combination and revised subgeneric combination by Özdikmen 2009: 145].
Pelidnota flavovittata (Perty) [removal of subgeneric classification by Soula 2009: 115].

#### Distribution.

BRAZIL: Minas Gerais (Ohaus 1929, 1934b, Blackwelder 1944, Machatschke 1972, Soula 2006, Krajcik 2008).

### Pelidnota fracida

Taxon classificationAnimaliaColeopteraScarabaeidae

F. Bates, 1904

Pelidnota fracida F. Bates, 1904: 258, 269 [original combination].
Pelidnota (Pelidnota) fracida F. Bates [new subgeneric combination by Ohaus 1918: 23].
Pelidnota fracida F. Bates [removal of subgeneric classification by Soula 2009: 105–106].
Pelidnota (Pelidnota) testaceipes Casey, 1915 **synonym.**
Pelidnota (Pelidnota) testaceipes Casey, 1915: 75 [original combination].
Pelidnota (Pelidnota) fracida F. Bates [syn. by Ohaus 1925: 76].

#### Distribution.

BRAZIL: Amazonas, Pará (F. Bates 1904, Casey 1915, Ohaus 1918, 1925, 1934b, Blackwelder 1944, Machatschke 1972, Krajcik 2008, Soula 2009).

#### Types.

**Figure 65.**
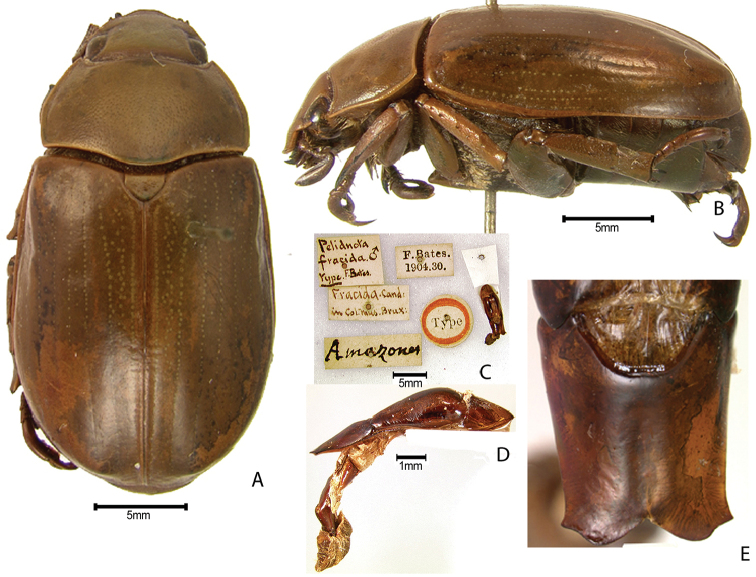
*Pelidnota fracida* F. Bates syntype male from BMNH. **A** Dorsal habitus **B** Lateral habitus **C** Specimen labels and male genitalia **D** Male genitalia, lateral view **E** Male parameres, dorsal view.

1 ♂ syntype at BMNH (Soula 2009) (Fig. 65).

### Pelidnota frommeri

Taxon classificationAnimaliaColeopteraScarabaeidae

Hardy, 1975

Pelidnota (Pelidnota) frommeri Hardy, 1975: 7, 25–26 [original combination].
Pelidnota frommeri Hardy [removal of subgeneric classification by Soula 2009: 54–55].

#### Distribution.

COSTA RICA: Alajuela, Cartago, Guanacaste, Heredia, Limón, San José (Hardy 1975, Morón 1979, Maes 1987, Solís and Morón 1994, Krajcik 2008, Soula 2009, García-López et al. 2013). ECUADOR: Guayas (Hardy 1975, Morón 1979, Maes 1987). HONDURAS (Hardy 1975, Soula 2009). MEXICO: Chiapas, Oaxaca, Tabasco, Veracruz (Hardy 1975, Morón 1979, Maes 1987, Lobo and Morón 1993, Morón et al. 1997). NICARAGUA: Chontales (Hardy 1975, Morón 1979, Maes 1987, Soula 2009).

#### Types.

1 ♂ holotype and 1 ♀ allotype at USNM (Hardy 1975, Soula 2009); 4 ♀ paratypes at CMNC; 5 paratypes at BMNH; additional paratypes at CAS, CNC, LACM, MCZ, NHMB and USNM (Hardy 1975).

### Pelidnota fulva

Taxon classificationAnimaliaColeopteraScarabaeidae

Blanchard, 1851

Pelidnota fulva Blanchard, 1851: 211 [original combination].
Pelidnota (Pelidnota) fulva Blanchard [new subgeneric combination by Ohaus 1918: 23].
Pelidnota fulva Blanchard [removal of subgeneric classification by Soula 2009: 90–91].

#### Distribution.

BOLIVIA: Chuquisaca (Blanchard 1851, Burmeister 1855, Ohaus 1918, 1934b, Blackwelder 1944, Machatschke 1972, Krajcik 2008, Soula 2009). BRAZIL: Bahia, Mato Grosso do Sul, Minas Gerais (Burmeister 1855, Ohaus 1908a, Rodrigues and da Silva Falco 2011, Rodrigues et al. 2012, Garcia et al. 2013).

#### Types.

1 ♂ lectotype and 1 paralectotype at MNHN (Soula 2009).

### Pelidnota fusciventris columbica

Taxon classificationAnimaliaColeopteraScarabaeidae

(Soula, 2006)

Strigidia fusciventris columbica Soula, 2006: 24 [original combination].
Pelidnota (Strigidia) fusciventris columbica (Soula) [new combination and new subgeneric combination by Özdikmen 2009: 145].
Pelidnota fusciventris columbica (Soula) [removal of subgeneric classification by Soula 2009: 115].

#### Distribution.

COLOMBIA: Cundinamarca, Valle del Cauca (Soula 2006).

#### Types.

The following specimen is deposited at CCECL. 1 ♂ holotype: “Columbia Cumaral 400 m [obverse] RU 137 ♂ 2.59.//Holotype 2005 *Strigidia fusciventris columbica* Sou. Soula det.” (47030284). Genitalia card-mounted underneath the male holotype. Box 4618658 SOULA.

### Pelidnota fusciventris fusciventris

Taxon classificationAnimaliaColeopteraScarabaeidae

Ohaus, 1905

Pelidnota fusciventris Ohaus, 1905: 318–319 [original combination].
Pelidnota (Ganonota) fusciventris Ohaus [new subgeneric combination by Ohaus 1918: 28].
Pelidnota (Strigidia) fusciventris Ohaus [new subgeneric combination by Machatschke 1972: 30].
Pelidnota (Odontognathus) fusciventris Ohaus [new subgeneric combination by Hardy 1975: 4].
Pelidnota (Ganonota) fusciventris Ohaus [revised subgeneric combination by Frey 1976: 345].
Strigidia fusciventris (Ohaus) [new combination by Soula 2006: 22–23].
Pelidnota (Strigidia) fusciventris Ohaus [revised combination and revised subgeneric combination by Özdikmen 2009: 145].
Pelidnota fusciventris Ohaus [removal of subgeneric classification by Soula 2009: 115].

#### Distribution.

PERU: Junín, Pasco (Ohaus 1905, 1918, 1934b, 1952, Blackwelder 1944, Machatschke 1972, Frey 1976, Soula 2006, Krajcik 2008, Ratcliffe et al. 2015). BRAZIL: Mato Grosso (Frey 1976).

#### Types.

**Figure 66.**
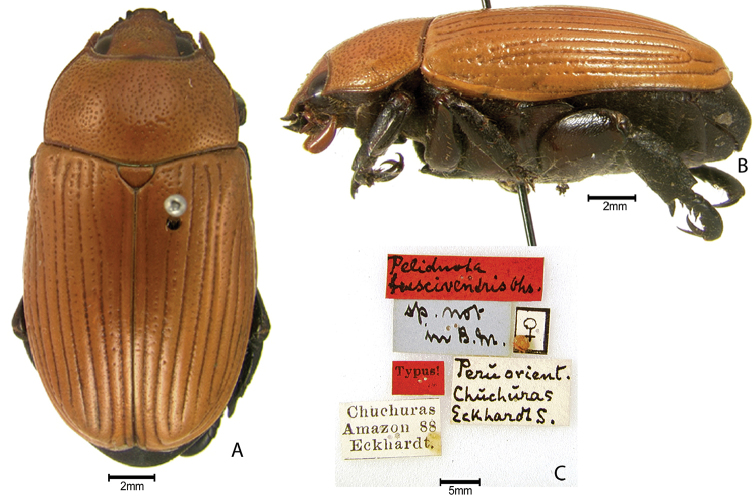
*Pelidnota fusciventris* Ohaus type female from ZMHB. **A** Dorsal habitus **B** Lateral habitus **C** Specimen labels and egg.

1 paralectotype of *Pelidnota fusciventris* at ZMHB (Soula 2006) and ♀ type specimen at ZMHB (Fig. 66) (see “*Type Specimens and Lectotype Designation*” in Methods).

### Pelidnota fusciventris lecourti

Taxon classificationAnimaliaColeopteraScarabaeidae

(Soula, 2006)

Strigidia fusciventris lecourti Soula, 2006: 23–24 [original combination].
Pelidnota (Strigidia) fusciventris lecourti (Soula) [new combination and new subgeneric combination by Özdikmen 2009: 145].
Pelidnota fusciventris lecourti (Soula) [removal of subgeneric classification by Soula 2009: 115].

#### Distribution.

BOLIVIA: La Paz, Cochabamba (Soula 2006).

#### Types.

The following specimens are deposited at CCECL. 1 ♂ holotype, 1 ♀ allotype, 4 ♂ paratypes, 3 ♀ paratypes: “Caranavi 1000 m III/2002 M. SOULA det 19//Holotype 2005 *Strigidia fusciventris lecourti* Sou. Soula det.” (47030275); “Caranavi 1000 m ? III/2002 M. SOULA det 19//Allotype *Strigidia fusciventris lecourti* Sou. Soula det.” (47030276); “Cristal Mayu Chaparé (B) 8/87//Paratype 2005 *Strigidia fusciventris lecourti* Sou. Soula det.” (47030278); Two paratypes with identical label data “Cristal Mayu Chapare (B) 10/87//Paratype 2005 *Strigidia fusciventris lecourti* Sou. Soula det.” (47030279 and 47030280); “Caranavi 800 m 21/X/2000 M. SOULA det 19//Paratype 2006 *Strigidia fusciventris lecourti* Sou. Soula det.” (47030277); “Cristal Mayu Chaparé (B) 10/88//Paratype 2005 *Strigidia fusciventris lecourti* Sou. Soula det.” (47030281); “Bolivie M. SOULA det 19//Paratype 2005 *Strigidia fusciventris lecourti* Sou. Soula det.” (47030283); “Ron 1531//Paratype 2005 *Strigidia fusciventris lecourti* Sou. Soula det.” (47030282). Genitalia card-mounted underneath the male holotype and four male paratypes. Box 4618658 SOULA.

### Pelidnota fuscoviridis

Taxon classificationAnimaliaColeopteraScarabaeidae

Ohaus, 1913

Pelidnota fuscoviridis Ohaus, 1913: 500–501 [original combination].
Pelidnota (Pelidnota) fuscoviridis Ohaus [new subgeneric combination by Ohaus 1918: 24].
Pelidnota fuscoviridis Ohaus [removal of subgeneric classification by Soula 2009: 44].

#### Distribution.

VENEZUELA (Ohaus 1913, 1918, 1934b, Blackwelder 1944, Machatschke 1972, Krajcik 2008, Soula 2009).

#### Types.

1 ♀ syntype of *Pelidnota fuscoviridis* at ZMHB (Soula 2009).

### Pelidnota gabrielae

Taxon classificationAnimaliaColeopteraScarabaeidae

Martínez, 1979

Pelidnota (Odontognathus) gabrielae Martínez, 1979: 1–3 [original combination].
Strigidia gabrielae (Martínez) [new combination by Soula 2006: 47–48].
Pelidnota (Strigidia) gabrielae Martínez [revised combination and new subgeneric combination by Özdikmen 2009: 145].
Pelidnota gabrielae Martínez [removal of subgeneric classification by Soula 2009: 115].

#### Distribution.

VENEZUELA: Amazonas, Bolívar (Martínez 1979, Soula 2006).

#### Types.

**Figure 67.**
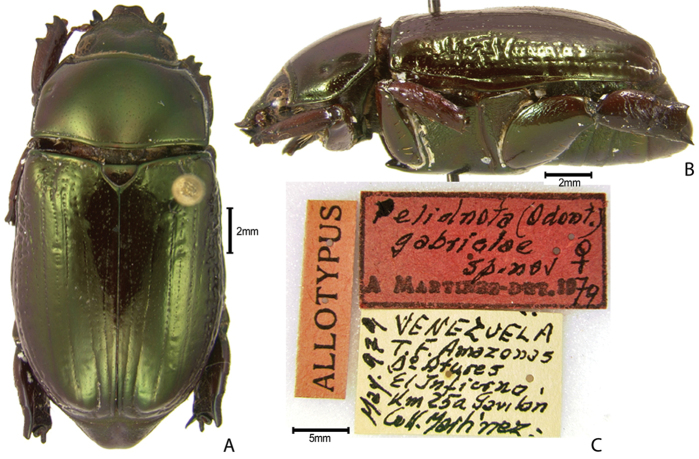
Pelidnota (Odontognathus) gabrielae Martínez (valid name *Pelidnota gabrielae* Martínez) allotype female from MACN. **A** Dorsal habitus **B** Lateral habitus **C** Specimen labels.

Allotype specimen (♀) of Pelidnota (Odontognathus) gabrielae at MACN (Fig. 67). Martínez (1979) stated that the holotype ♂ was deposited in his collection (MACN). 1 ♂ paratype in CMNC.

#### Remarks.

Based on examination of specimens including type specimens, it is possible that *P. labyrinthophallica* is a junior synonym of *P. gabrielae*. Soula’s illustration of the male genitalia of *P. gabrielae* (Soula 2006: 48) differ slightly from the illustration of the male genitalia of *P. labyrinthophallica* (Soula 2006: 49), likely due to position of the parameres. The differences in these illustrations may also have prevented Soula from recognizing that the two species are very likely conspecific.

Martínez (1979) provided drawings of the male genitalia (dorsal and lateral views). He named the species in honor of his daughter, Gabriela I. C. de Martínez who helped collect it. The type series included the holotype male, allotype female, and one male paratype. He placed the species in the subgenus Odontognathus and compared it with others in the subgenus (*P. soederstroemi, P. viridicuprea, P. adriani*, and *P. pulchella*). Martínez (1979) commented that the species was collected at light in a tropical humid forest at 450 m elevation. Soula (2006) commented on the “remarkable” form of sternite 4 that is very short and ventrally produced (also observed in *P. neitamorenoi* Soula).

### Pelidnota genieri

Taxon classificationAnimaliaColeopteraScarabaeidae

(Soula, 2006)

Strigidia genieri Soula, 2006: 76 [original combination].
Pelidnota genieri Soula [new combination by Soula 2009: 115].

#### Distribution.

VENEZUELA: Tachira Betania (Soula 2006).

#### Types.

The following specimen is deposited at CMNC. 1 ♂ holotype: “Venezuela-Tachi-na. 2425 m. 16-20-III-1983//Exp. Instituto Zoologia Agricola Fac. Agronomia//Betania via Paramo El Tama//Collection François Génier//Holotype *Strigidia genieri* S. 2006 Soula”.

#### Remarks.

*Strigidia genieri* Soula, 2006 was transferred to *Pelidnota* by Soula (2009), thus creating a case of secondary homonymy with *Pelidnota genieri* Soula, 2009 (ICZN Article 52.1) making the name invalid and requiring a replacement name. *Pelidnota francoisgenieri* (=*P. punctata* [Linnaeus]) was proposed as a replacement name for *Pelidnota genieri* Soula, 2009 (Moore and Jameson 2013).

### Pelidnota gilletti

Taxon classificationAnimaliaColeopteraScarabaeidae

Soula, 2009

Pelidnota gilletti Soula, 2009: 31, 55–56 [original combination].

#### Distribution.

MEXICO: Chiapas, Veracruz (Soula 2009).

#### Types.

The following specimens are deposited at CCECL. 1 ♂ holotype, 1 ♀ allotype, 1 ♂ paratype, 1 ♀ paratype, 2 ♂ invalid paratypes, 3 ♀ invalid paratypes: “*P. centro-americana* Sta Rosa Chiapas 8/90//Holotype *Pelidnota gilleti* S. Soula det. 2006” (47030463); “*P. centro-americana* Sta Rosa Chiapas 8/90//Allotype *Pelidnota gilleti* S. Soula det. 2006” (47030464); Two paratypes with identical label data “Sta Rosa Chiapas 8/90 (Mex.)//Paratype 2006 *Pelidnota gilleti* S. Soula” (47030465 and 47030466); Three invalid paratypes with identical label data “San Pedro de Soteapan VERACRUZ – 500 m. MEXIQUE – Sept. 1987 Thierry PORION Leg.//Paratype 2006 *Pelidnota gilleti* S. Soula//Invalid Paratype det. M. R. Moore 2014” (47030467 to 47030469); “San Pedro de Soteapan VERACRUZ – 500 m. MEXIQUE – Sept. 1987 Thierry PORION Leg.//Paratype *Pelidnota gilleti* Sou. 2006 Soula//Invalid Paratype det. M. R. Moore 2014” (47030470); “METATEZ OAXACA MEXIQUE IX. 85//METATEZ OAXACA MEXIQUE IX. 85//Paratype 2006 *Pelidnota gilleti* S. Soula//Invalid Paratype det. M. R. Moore 2014” (47030471). Genitalia card-mounted underneath the male holotype. Box 4618665 SOULA.

#### Remarks.

We designated five paratypes as invalid because these specimens do not have label data that matches Soula’s (2009) description of the species. Soula wrote “gilleti” (*sic*) on all labels.

### Pelidnota girardi

Taxon classificationAnimaliaColeopteraScarabaeidae

(Bouchard, 2003)

Chalcoplethis girardi Bouchard, 2003: 103–108 [original combination].
Strigidia girardi (Bouchard) [new combination by Soula 2006: 59-60].
Pelidnota girardi (Bouchard) [new combination by Soula 2009: 115].

#### Distribution.

FRENCH GUIANA (Bouchard 2003, Krajcik 2008, Soula 2006, 2010c).

#### Types.

The following specimens are deposited at CCECL. 4 ♂ paratypes, 15 ♀ paratypes: Two paratypes with identical label data “Piste de Belizon (G. F.) coll. – SOULA//*Chalcoplethis girardi* sp. n. PARATYPE” (47030264 and 47030265); Two paratypes with identical label data “M. Kaw I. 89 guyane F.//*Chalcoplethis girardi* sp. n. PARATYPE” (47030270 and 47030271); “Dd Saramaca PK. 6 Kourou/Guyane Fse 16.17 XII 1987 M.Duranton Recolt.//*Chalcoplethis girardi* sp. n. PARATYPE” (47030274); Two paratypes with identical label data “Guyane f. Kourou VIII 1990//*Chalcoplethis girardi* sp. n. PARATYPE” (47030258, exch17); “Tingo Maria (Pé) 9/93 coll. – SOULA//*Chalcoplethis girardi* sp. n. PARATYPE” (47030267); “Rocoucova PK 3 P.L. 25/1/85//*Chalcoplethis girardi* sp. n. PARATYPE” (47030269); “P. de Kaw G. Franç. coll. – SOULA//*Chalcoplethis girardi* sp. n. PARATYPE” (47030263); Two paratypes with identical label data “M. de Kaw guyane Fr. 8. 90//*Chalcoplethis girardi* sp. n. PARATYPE” (47030272 and 47030273); “Coralie G. F. 15/12/92//*Chalcoplethis girardi* sp. n. PARATYPE” (47030268); Three paratypes with identical label data “Saut Dalles G. F. 7/03/92//*Chalcoplethis girardi* sp. n. PARATYPE” (47030260, 47030261, exch16); “Piste de Kaw G. F. 02/93 coll. – SOULA//*Chalcoplethis girardi* sp. n. PARATYPE” (47030262); “ et 2/ 90 [obverse] Piste de Belizon coll. – SOULA//Piste de Belizon G. F. coll. – SOULA//*Chalcoplethis girardi* sp. n. PARATYPE” (47030266); “Petit Saut G. F. 07/92//*Chalcoplethis girardi* sp. n. PARATYPE” (47030259). Genitalia card-mounted underneath three male paratypes and three female paratypes. Box 4618657 SOULA.

### Pelidnota glaberrima glaberrima

Taxon classificationAnimaliaColeopteraScarabaeidae

Blanchard, 1851

Pelidnota glaberrima Blanchard, 1851: 213–214 [original combination].
Pelidnota (Ganonota) glaberrima Blanchard [new subgeneric combination by Ohaus 1918: 26].
Pelidnota (Strigidia) glaberrima Blanchard [new subgeneric combination by Machatschke 1970: 157].
Pelidnota (Odontognathus) glaberrima (Blanchard) [new subgeneric combination by Hardy 1975: 4].
Strigidia glaberrima (Blanchard) [new combination by Soula 2006: 36–37].
Pelidnota (Strigidia) glaberrima Blanchard [revised combination and revised subgeneric combination by Özdikmen 2009: 145].
Pelidnota glaberrima glaberrima Blanchard [removal of subgeneric classification by Soula 2009: 115].

#### Distribution.

ARGENTINA: Misiones (MLJC). BRAZIL: Espírito Santo, Minas Gerais, Rio de Janeiro, São Paulo (Blanchard 1851, Ohaus 1918, 1934b, Blackwelder 1944, Machatschke 1972, Soula 2006, Krajcik 2008).

#### Types.

1 ♂ syntype of *Pelidnota glaberrima* at MNHN (Soula 2006).

#### Remarks.

CCECL contains a specimen of *P. glaberrima glaberrima* that is labeled as a female alloréférent with the following data: 1 ♀ alloréférent: “Jtatiaya R.d Janeiro//*Ganonota glaberrima* Bl Burgeon L. 1930 [0 crossed out] 1 det://R. I. Sc. N. B. 16.117 L. Burgeon, coll. et det. ://Alloréférent ♀ de *Strigidia glaberrima* (Bl.) M. SOULA det 19 2005” (47030396). Box 4618663 SOULA.

### Pelidnota glaberrima meridionalis

Taxon classificationAnimaliaColeopteraScarabaeidae

(Soula, 2006)

Strigidia glaberrima meridionalis Soula, 2006: 37 [original combination].
Pelidnota (Strigidia) glaberrima meridionalis (Soula) [new combination and new subgeneric combination by Özdikmen 2009: 145].
Pelidnota glaberrima meridionalis (Soula) [removal of subgeneric classification by Soula 2009: 115].

#### Distribution.

ARGENTINA: Misiones (Soula 2006). BRAZIL: Rio de Janeiro (Soula 2006). PARAGUAY (Soula 2006).

#### Types.

The following specimens are deposited at CCECL. 1 ♂ holotype, 1 ♀ allotype, 3 ♂ paratypes, 15 ♀ paratypes: “Puerto Iguazu ARGENTINE (I/93)//Holotype 2004 *Strigidia glaberrima meridionalis* Sou. Soula det.” (47030400); “Puerto Iguazu, ARGENTINE (I/93)//Allotype 2004 *Strigidia glaberrima meridionalis* Soula det. Sou.” (47030401); Eight paratypes with identical label data “Puerto Iguazu ARGENTINE (I/93)//Paratype 2005 *Strigidia glaberrima meridionalis* Sou. Soula det.” (47030402 to 47030407, exch22 and exch23); Three paratypes with identical label data “Tayao Aeguazu coll. – SOULA [obverse] Paraguay, 10/10/98//Paratype 2005 *Strigidia glaberrima meridionalis* Sou. Soula det.” (47030411 to 47030413); “Iguazu Misiones (Arg.) coll. – SOULA//Paratype 2005 *Strigidia glaberrima meridionalis* Sou. Soula det.” (47030415); “Puerto Iguazu-ARG-XII/88//Paratype 2005 *Strigidia glaberrima meridionalis* Sou Soula det..” (47030414); Three paratypes with identical label data “Eldorado – Misiones, ARGENTINE (I/93)//Paratype 2005 *Strigidia glaberrima meridionalis* Sou. Soula det.” (47030408 to 47030410); “ARGENTINE: Iguazu, Misiones, XII-88//Paratype 2005 *Strigidia glaberrima meridionalis* Sou. Soula det.” (47030416); “Nova Friborgo (sic for Firburgo) – R.J. XII/92 – BRESIL//Paratype 2005 *Strigidia glaberrima meridionalis* Sou. Soula det.” (47030417). Genitalia card-mounted underneath the male holotype and paratypes. Box 4618663 SOULA.

### Pelidnota glaberrima septentrionalis

Taxon classificationAnimaliaColeopteraScarabaeidae

(Soula, 2006)

Strigidia glaberrima septentrionalis Soula, 2006: 37–38 [original combination].
Pelidnota (Strigidia) glaberrima septentrionalis (Soula) [new combination and new subgeneric combination by Özdikmen 2009: 145].
Pelidnota glaberrima septentrionalis (Soula) [removal of subgeneric classification by Soula 2009: 116].

#### Distribution.

BRAZIL: Bahia (Soula 2006).

#### Types.

The following specimen is deposited at CCECL. 1 ♂ holotype: “Cachimbo Prov.de.Bahia Ch Pujol 1890//Muséum Paris ex Coll. R. Oberthür 1952//Holotype 2005 *Strigidia glaberrima septentrionalis* Sou. Soula det.” (47030418). Genitalia card-mounted underneath the specimen. Box 4618663 SOULA.

### Pelidnota glabra audureaui

Taxon classificationAnimaliaColeopteraScarabaeidae

Soula, 2009

Pelidnota glabra audureaui Soula, 2009: 130–131 [original combination].

#### Distribution.

NICARAGUA: Granada (Soula 2009).

#### Types.

The following specimens are deposited at CCECL. 1 ♂ holotype, 1 ♀ allotype, 1 ♂ paratype, 6 ♀ paratypes: “Reserva Silvestre de Domitila Granada prov. PL NICARAGUA 09-21.06.2007 Alain Audureau leg.//Holotype 2008 *Pelidnota glabra audureaui* S. Soula” (47030347); “Reserve sylvestre de Domitila M. SOULA det 19 [obverse] Granada Prov Nicaragua 09-21/6/2007 P.L.//Allotype 2008 *Pelidnota glabra audureaui* S. Soula” (47030348); “Reserve sylv. de Domitila Granada Prov. M. SOULA det 19 [obverse] Nicaragua 09-21/6/2007//Paratype 2008 *Pelidnota glabra audureaui* S. Soula” (47030349); “Reserve sylv. de Domitila Granada Prov. M. SOULA det 19 [obverse] Nicaragua P. L. 1-5/6/2005//Paratype 2008 *Pelidnota glabra audureaui* S. Soula” (47030350); “Reserve syl. de Domitila Granada Prov. M. SOULA det 19 [obverse] Nicaragua P. L. 1-5/6/2005//Paratype 2008 *Pelidnota glabra audureaui* Soula” (47030351); “Reserva silvestra de Domitila PL Granade prov. Nicaragua 01-05.06.2005 Alain Audureau leg.//Paratype 2008 *Pelidnota glabra audureaui* S. Soula” (47030352); “Reserva silvestra privada de Domitila PL Granada prov. NICARAGUA 13-16/06/2004 Alain Audureau legit//Paratype 2008 *Pelidnota glabra audureaui* S. Soula” (47030353); “Bartola lodge PL Rio San Juan Nicaragua 06-13. VI.2005 Alain & Sylvaine Audureau//Paratype 2008 *Pelidnota glabra audureaui* S. Soula” (47030354); “Bartola lodge PL Rio San Juan Nicaragua 06-13. VI.2005 Alain & Sylvaine Audureau leg.//Paratype 2008 *Pelidnota glabra audureaui* S. Soula” (47030355). Genitalia card-mounted underneath the male holotype and the three male paratypes. Box 4618661 SOULA.

### Pelidnota glabra glabra

Taxon classificationAnimaliaColeopteraScarabaeidae

Ohaus, 1922

Pelidnota glabra Ohaus, 1922: 324 [original combination].
Pelidnota (Chalcoplethis) glabra Ohaus [new subgeneric combination by Ohaus 1934b: 84].
Strigidia glabra (Ohaus) [new combination by Soula 2006: 57].
Pelidnota glabra Ohaus [revised combination by Soula 2009: 115].

#### Distribution.

COSTA RICA: Cartago, Guanacaste, Limón (Ohaus 1922, 1934b, Blackwelder 1944, Machatschke 1972, Hardy 1975, Solís and Morón 1994, Soula 2006). PANAMA: Colón, Panama (Ratcliffe 2002).

#### Types.

1 ♂ syntype of *Pelidnota glabra* at ZMHB (Hardy 1975, Soula 2006).

### Pelidnota gracilis debahia

Taxon classificationAnimaliaColeopteraScarabaeidae

(Soula, 2006)

Strigidia gracilis debahia Soula, 2006: 30 [original combination].
Pelidnota (Strigidia) gracilis debahia (Soula) [new combination and new subgeneric combination by Özdikmen 2009: 145].
Pelidnota gracilis debahia (Soula) [removal of subgeneric classification by Soula 2009: 115].

#### Distribution.

BRAZIL: Bahia (Soula 2006).

#### Types.

The following specimens are deposited at CCECL. 1 ♂ holotype, 1 ♀ allotype, 3 ♀ paratypes: “Bahia Bresil//*Pelidnota gracilis*//Holotype 2006 *Strigidia gracilis debahia* Sou. Soula” (47030299); “Cachimbo Prov. de Bahia Ch. Pujol 1890//Museum Paris ex. Coll. R. Oberthur//Allotype 2006 *Strigidia gracilis debahia* Sou. Soula” (47030300); Three paratypes with identical label data “Brésil//Allotype 2006 *Strigidia gracilis debahia* Sou. Soula” (47030301 to 47030303). Genitalia card-mounted underneath the male holotype. Box 4618659 SOULA.

### Pelidnota gracilis gracilis

Taxon classificationAnimaliaColeopteraScarabaeidae

(Gory, 1834)

Rutela gracilis Gory, 1834: 111 [original combination].
Pelidnota gracilis (Gory) [new combination by Burmeister 1844: 395-396].
Pelidnota (Ganonota) gracilis (Gory) [new subgeneric combination by Ohaus 1918: 27].
Pelidnota (Strigidia) gracilis (Gory) [new subgeneric combination by Machatschke 1970: 157].
Pelidnota (Odontognathus) gracilis (Gory) [new subgeneric combination by Hardy 1975: 4].
Strigidia gracilis (Gory) [new combination by Soula 2006: 29–30].
Pelidnota (Strigidia) gracilis (Gory) [revised combination and revised subgeneric combination by Özdikmen 2009: 145].
Pelidnota gracilis gracilis (Gory) [removal of subgeneric classification by Soula 2009: 115].

#### Distribution.

BRAZIL: Espírito Santo, Minas Gerais, Rio de Janeiro (Gory 1834, Burmeister 1844, Blanchard 1851, Ohaus 1908a, 1918, 1934b, Blackwelder 1944, Machatschke 1972, Soula 2006, Krajcik 2008). PARAGUAY: Guairá (WBWC).

### Pelidnota gracilis wagneri

Taxon classificationAnimaliaColeopteraScarabaeidae

(Soula, 2006)

Strigidia gracilis wagneri Soula, 2006: 30 [original combination].
Pelidnota (Strigidia) gracilis wagneri (Soula) [new combination and new subgeneric combination by Özdikmen 2009: 145].
Pelidnota gracilis wagneri (Soula) [removal of subgeneric classification by Soula 2009: 116].

#### Distribution.

ARGENTINA: Misiones (Soula 2006).

#### Types.

The following specimens are deposited at CCECL. 1 ♂ holotype, 1 ♀ allotype, 4 ♂ paratypes, 1 ♀ paratype, 1 invalid ♀ allotype: “Iguazu Misiones (Arg.) coll. – SOULA//Holotype 2006 *Strigidia gracilis wagneri* Sou. Soula” (47030304); “Puerto Iguazu-ARG XII/88//Allotype 2006 *Strigidia gracilis wagneri* Sou. Soula” (47030305); “Puerto Iguazu (Arg.) coll. – SOULA [obverse] 11/89//Invalid ♀ Allotype probable paratype of *P. gracilis wagneri* Soula det. M. R. 2014//Allotype 2006 *Strigidia gracilis wagneri* S. Soula” (47030306); Two paratypes with identical label data “Puerto Iguazu (Arg.) coll. – SOULA [obverse] 11/87//Paratype 2006 *Strigidia gracilis wagneri* Sou. Soula” (47030310 and 47030311); Two paratypes with identical label data “Iguazu Misiones (Arg.) coll. – SOULA//Paratype 2006 *Strigidia gracilis wagneri* Sou. Soula” (47030307 and 47030308); “Iguazu Misiones (Ar.) coll. – SOULA//Paratype 2006 *Strigidia gracilis wagneri* Sou. Soula” (47030309). Genitalia card-mounted underneath the male holotype and four male paratypes. Box 4618659 SOULA.

#### Remarks.

The female allotype specimen labeled from “Puerto Iguazu (Arg.)” is not the valid allotype specimen. Soula (2006) did not report the exact label data and number of paratypes for this species. However, the holotypes and allotypes were typically arranged side-by-side in his collection. The invalid allotype female was not directly next to the holotype male. Additionally, the invalid allotype has a red type label that is a slightly different color. We labeled this specimen as a probable paratype.

### Pelidnota grangesi

Taxon classificationAnimaliaColeopteraScarabaeidae

(Soula, 2006)

Strigidia grangesi Soula, 2006: 10, 39 [original combination].
Pelidnota grangesi (Soula) [new combination by Soula 2009: 115].

#### Distribution.

BOLIVIA: Cochabamba, La Paz (Soula 2006).

#### Types.

The following specimens are deposited at CCECL. 1 ♂ holotype, 1 ♀ allotype, 3 ♂ paratypes, 2 ♀ paratypes: “Coroïco à Caranavi 850 m (B) 10/90//Holotype 2005 *Strigidia grangesi* Sou. Soula det.” (47030286); “De Coroïco à Caranavi 850 m 10/88//Allotype 2005 *Strigidia grangesi* Sou. Soula det.” (47030287); Two paratypes with identical labels “Coroïco à Caranavi 850 m (B) 10/90//Paratype 2006 *Strigidia grangesi* Sou. Soula det.” (47030289 and 47030290); “De Coroïco à Caranavi 850 m 10/88//Paratype 2006 *Strigidia grangesi* Sou. Soula” (47030288); “N. Yungas (Bo.) coll. – SOULA//Paratype 2006 *Strigidia grangesi* Sou. Soula” (47030291); “Cochabamba a Villa Tunasi (sic for Tunari) pk 102 (2000 m) [obverse] 10/88 (B)//Paratype 2006 *Strigidia grangesi* Sou. Soula” (47030292). Genitalia card-mounted underneath the male holotype and three male paratypes. Box 4618658 SOULA.

### Pelidnota granulata

Taxon classificationAnimaliaColeopteraScarabaeidae

(Gory, 1834)

Rutela granulata Gory, 1834: 112 [original combination].
Pelidnota granulata (Gory) [new combination by Burmeister 1844: 399].
Pelidnota (Chalcoplethis) granulata (Gory) [new subgeneric combination by Ohaus 1918: 28].
Strigidia granulata (Gory) [new combination Soula 2006: 67-68].
Pelidnota granulata (Gory) [revised combination by Soula 2009: 115].

#### Distribution.

BRAZIL: Amazonas (INPA). FRENCH GUIANA: Cayenne, St.-Laurent du Maroni (Gory 1834, Burmeister 1844, Blanchard 1851, Ohaus 1912, 1934b, Blackwelder 1944, Machatschke 1972, Krajcik 2008, Soula 2006, 2010a, c). GUYANA: Cuyuni-Mazaruni (Ohaus 1912, 1918, 1934b, Blackwelder 1944, Machatschke 1972).

#### Types.

1 ♂ neotype of *Rutela granulata* at MNHN (Soula 2010a).

### Pelidnota grossiorum

Taxon classificationAnimaliaColeopteraScarabaeidae

Soula, 2009

Pelidnota grossiorum Soula, 2009: 34, 110–111 [original combination].

#### Distribution.

BRAZIL: Minas Gerais (Soula 2009).

#### Types.

The following specimens are deposited at CCECL. 1 ♂ holotype, 1 ♀ allotype, 3 ♂ paratypes, 2 ♀ paratypes, 1 ♂ invalid paratype: “Ipatinga M. G. XI/992 (sic) - BRESIL//Holotype 2008 *Pelidnota grossiorum* S. Soula” (47030799); “BRASIL: MG Cordisburgo Faz. Pontinha XII/1993 F. Z. Vaz de Mello//Allotype 2008 *Pelidnota grossiorum* S. Soula” (47030800); Three paratypes with identical label data: “Ipatinga M. G. XI/992 (sic) - BRESIL//Paratype 2008 *Pelidnota grossiorum* Soula” (47030801 to 47030803); “BRASIL: MG Cordisburgo Faz. Pontinha XII/1993 F. Z. Vaz de Mello//Paratype 2009 *Pelidnota grossiorum* S. Soula” (47030804); “Vale Rio Doce Minas Geraes 10/86 [obverse] Minas Geraes//Paratype 2009 *Pelidnota grossiorum* S. Soula” (47030805); “Ipatinga M.G. 11/94 coll. – SOULA//Paratype 2008 *Pelidnota grossiorum* S. Soula//Invalid Paratype *Pelidnota grossiorum* Soula det. MR Moore ‘15” (47030806). Genitalia card-mounted underneath the male holotype, the female allotype and the invalid male paratype. Box 4618681 SOULA.

#### Remarks.

One male specimen labeled as a paratype bears a collecting date that was not reported in Soula (2009). This specimen is considered an invalid paratype.

### Pelidnota guatemalensis

Taxon classificationAnimaliaColeopteraScarabaeidae

H. W. Bates, 1888

Pelidnota costaricensis var. guatemalensis H. W. Bates, 1888: 274 [original combination].
Pelidnota (Pelidnota) costaricensis guatemalensis H. W. Bates [new subgeneric combination and new subspecific status by Ohaus 1918: 23].
Pelidnota (Pelidnota) guatemalensis H. W. Bates [new species status by Hardy 1975: 17–18].
Pelidnota guatemalensis H. W. Bates [removal of subgeneric classification by Soula 2009: 70–71].
Pelidnota (Pelidnota) composita Casey, 1915 **synonym.**
Pelidnota (Pelidnota) composita Casey, 1915: 71 [original combination].
Pelidnota (Pelidnota) guatemalensis H. W. Bates [syn. by Hardy 1975: 17].

#### Distribution.

BELIZE: Toledo (H. W. Bates 1888, Blackwelder 1944, Hardy 1975, Alcázar-Ruiz et al. 2003, Soula 2009). GUATEMALA: Sacatepéquez (H. W. Bates 1888, Ohaus 1918, 1934b, Machatschke 1972, Hardy 1975, Alcázar-Ruiz et al. 2003, Krajcik 2008, Soula 2009). HONDURAS: Cortés (Ohaus 1918, 1934b, Blackwelder 1944, Machatschke 1972, Hardy 1975). MEXICO: Chiapas (Hardy 1975, Thomas 1993, Alcázar-Ruiz et al. 2003, Soula 2009).

#### Types.

1 ♂ lectotype of Pelidnota costaricensis var. guatemalensis at BMNH (Hardy 1975, Soula 2009); 4 paralectotypes at BMNH; additional paralectotypes at MNHN (Soula 2009).

### Pelidnota gwendolinae

Taxon classificationAnimaliaColeopteraScarabaeidae

(Soula, 2006)

Strigidia gwendolinae Soula, 2006: 10, 81 [original combination].
Pelidnota gwendolinae (Soula) [new combination by Soula 2009: 115].

#### Distribution.

BOLIVIA: Cochabamba, La Paz (Soula 2006).

#### Types.

The following specimens are deposited at CCECL. 1 ♂ holotype, 1 ♀ allotype, 7 ♂ paratypes, 7 ♀ paratypes: “Inca Huara 1450 m. 11/95 coll. – SOULA//Holotype 2006 *Strigidia gwendolinae* S. Soula det” (47030058); “Carasco 1450 m M. SOULA det. 19 [obverse] La Paz Prov. 22/10/97//Allotype 2006 *Strigidia gwendolinae* Soula det. S.” (47030059); Three paratype males with identical labels “Inca Huara 1450 m. 11/95 coll. – SOULA//Paratype 2006 *Strigidia gwendolinae* S. Soula” (47030060 and 47030061, exch01); “Cochabamba à Villa Tunasi (sic) pk 102 (B) (2000 m) [obverse] 10/88 //Paratype 2006 *Strigidia gwendolinae* S. Soula” (47030062); “Cochabamba à Villa Tunari pk 102 (B) (2000 m) 10/88//*Chalc. hoefigi* coll. – SOULA//Paratype 2006 *Strigidia gwendolinae* S. Soula” (47030063); “ Route de Coroico à Coranavi [pro Caranavi] (Bolivie)//[retranscription of faded label]//Paratype 2006 *Strigidia gwendolinae* S. Soula” (47030064); “Region des Yungas Bolivie//Paratype 2006 *Strigidia gwendolinae* S. Soula” (47030066); “Bolivie M. SOULA det 19//Paratype 2006 *Strigidia gwendolinae* S. Soula” (47030067); “5 km de Chuspipata coll. – SOULA [obverse] 2007 m la Paz Prov. 4/10/1996//Paratype 2006 *Strigidia gwendolinae* S. Soula” (47030065); One male and one female paratype with identical label data “Col. G LECOURT Rte de La Paz. Yocumo 1000m. Km 301 31/03/98 Prov. Alto Beni. Bolivie//Paratype 2006 *Strigidia gwendolinae* S. Soula” (47030068 to 47030069); “BOLIVIE – CARANAVI NOR YUNGAS – ALT.900m DU 16 AU 30/11/89 COLLECTION LECOURT//Paratype 2006 *Strigidia gwendolinae* S. Soula” (47030072); “Coll. P. BLEUZEN Nor - Yungas Bolivie XI 1990//Paratype 2006 *Strigidia gwendolinae* S. Soula” (47030071); “Col G. LECOURT Yungus (sic) Coroïco 1 700 m BOLIVIE [the date 03/1986 is crossed out] [obverse] 12 - 90//Paratype 2006 *Strigidia gwendolinae* S. Soula” (47030070)”. Genitalia card-mounted underneath the male holotype and 6 male paratypes. Box 4618650 SOULA.

### Pelidnota herbacea

Taxon classificationAnimaliaColeopteraScarabaeidae

Blanchard, 1851

Pelidnota herbacea Blanchard, 1851: 212 [original combination].
Pelidnota (Pelidnota) chlorana Erichson [syn. by Ohaus 1918: 23].
Pelidnota herbacea Blanchard [revised combination and revised species status by Soula 2009: 98–99].

#### Distribution.

BOLIVIA (Blanchard 1851, Ohaus 1918, 1934b, Machatschke 1972, Krajcik 2008, Soula 2009).

#### Types.

1 ♀ syntype of *Pelidnota herbacea* at MNHN (Soula 2009).

### Pelidnota hernanlequericai

Taxon classificationAnimaliaColeopteraScarabaeidae

(Soula, 2006)

Strigidia hernanlequericai Soula, 2006: 10, 40 [original combination].
Pelidnota hernanlequericai (Soula) [new combination by Soula 2009: 115].

#### Distribution.

PERU: Loreto (Soula 2006, Ratcliffe et al. 2015).

#### Types.

The following specimens are deposited at CCECL. 1 ♂ holotype, 1 ♀ invalid allotype: “Iquitos, Loreto Pérou, I-II/2005//Holotype 2006 *Strigidia hernanlequericai* Sou. Soula det.” (47030419); “Iquitos, Loreto Pérou; VIII/2003//Allotype 2006 Strigidia hernanlequericai Sou. Soula det.//Invalid Allotype ♀, see Soula 2006: 40 det. M. R. Moore ‘15” (47030420). Genitalia card-mounted underneath holotype. Box 4618663 SOULA.

#### Remarks.

Because there was no description or mention of an allotype specimen or paratype series of *P. hernanlequericai* (Soula 2006), it is likely that the allotype label was added after the publication of the name. We consider this specimen to be an invalid allotype.

### Pelidnota hirsutiphallica

Taxon classificationAnimaliaColeopteraScarabaeidae

Ratcliffe & Jameson, 1989

Pelidnota hirsutiphallica Ratcliffe & Jameson, 1989: 259–261 [original combination].
Strigidia santidomini (*sic*) (Ohaus) [syn. by Soula 2006: 78].
Pelidnota hirsutiphallica Ratcliffe and Jameson [revised combination and revised species status by Soula 2010a: 57].

#### Distribution.

COSTA RICA (Solís and Morón 1994). NICARAGUA: Jinotega (Maes et al. 1997). PANAMA: Bocas del Toro, Colón, Veraguas (Ratcliffe and Jameson 1989, Krajcik 2008, Soula 2010a).

#### Types.

1 ♂ holotype and 1 ♀ allotype of *Pelidnota hirsutiphallica* at UNSM (Ratcliffe and Jameson 1989).

#### Remarks.

Krajcik (2012, 2013) omitted this name from his catalogs.

### Pelidnota hoefigi

Taxon classificationAnimaliaColeopteraScarabaeidae

Ohaus, 1912

Pelidnota hoefigi Ohaus, 1912: 318 [original combination].
Pelidnota (Chalcoplethis) hoefigi Ohaus [new subgeneric combination by Ohaus 1918: 29].
Strigidia hoefigi (Ohaus) [new combination by Soula 2006: 80–81].
Pelidnota hoefigi Ohaus [revised combination by Soula 2009: 115].

#### Distribution.

FRENCH GUIANA: Saint-Georges (Gruner 1971). PERU: Cusco, Lima (Ohaus 1912, 1918, 1934b, 1952, Blackwelder 1944, Machatschke 1972, Soula 2006, Krajcik 2008, Ratcliffe et al. 2015).

#### Types.

**Figure 68.**
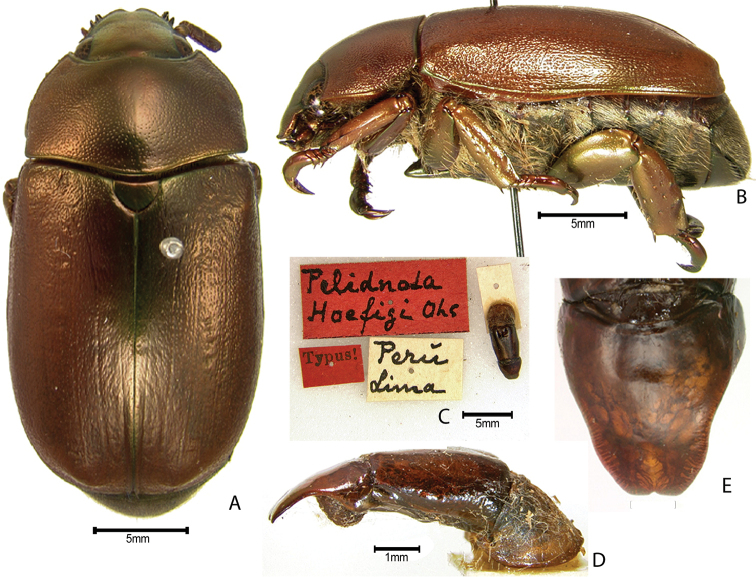
*Pelidnota hoefigi* Ohaus syntype male from ZMHB. **A** Dorsal habitus **B** Lateral habitus **C** Specimen labels and male genitalia **D** Male genitalia, lateral view **E** Male parameres, dorsal view.

1 ♂ syntype specimen of *Pelidnota hoefigi* Ohaus at ZMHB (Fig. 68).

### Pelidnota huetheri

Taxon classificationAnimaliaColeopteraScarabaeidae

Howden, 1998

Pelidnota (Pelidnota) huetheri Howden, 1998: 171–173 [original combination].
Pelidnota huetheri Howden [removal of subgeneric classification by Soula 2009: 56].

#### Distribution.

PANAMA: Chiriquí (Howden 1998, Ratcliffe 2002, Krajcik 2008, Soula 2009).

#### Types.

The following specimens are deposited at CMNC. 1 ♂ holotype and 1 ♀ allotype: “PANAMA Chiriqui Prv vic Hornito 4200’ 14-18 May 1996 Wappes Huether & Morris//HOLOTYPE *Pelidnota huetheri* H. Howden//Holotype *Pelidnota huetheri* How. Soula det. 2009//[barcode matrix] Canadian Museum of Musée canadien de la NATURE CMNEN 00011035”, allotype with identical collecting data label and database number CMNEN 00010902.

### Pelidnota impressicollis

Taxon classificationAnimaliaColeopteraScarabaeidae

Ohaus, 1925

Pelidnota (Ganonota) impressicollis Ohaus, 1925: 76–77 [original combination].
Pelidnota (Strigidia) impressicollis Ohaus [new subgeneric combination by Machatschke 1970: 157].
Pelidnota (Odontognathus) impressicollis Ohaus [new subgeneric combination by Hardy 1975: 4].
Strigidia impressicollis (Ohaus) [new combination by Soula 2006: 51–52].
Pelidnota (Strigidia) impressicollis Ohaus [revised combination and revised subgeneric combination by Özdikmen 2009: 145].
Pelidnota impressicollis Ohaus [removal of subgeneric classification by Soula 2009: 115].

#### Distribution.

BRAZIL: Mato Grosso (Ohaus 1925, 1934b, Blackwelder 1944, Machatschke 1972, Soula 2006, Krajcik 2008).

#### Types.

1 ♂ holotype of Pelidnota (Ganonota) impressicollis at ZMHB (Soula 2006).

### Pelidnota incerta

Taxon classificationAnimaliaColeopteraScarabaeidae

(Soula, 2006)

Strigidia incerta Soula, 2006: 9, 20 [original combination].
Pelidnota incerta (Soula) [new combination by Soula 2009: 115].

#### Distribution.

PERU: Huánuco (Soula 2006, Ratcliffe et al. 2015).

#### Types.

**Figure 69.**
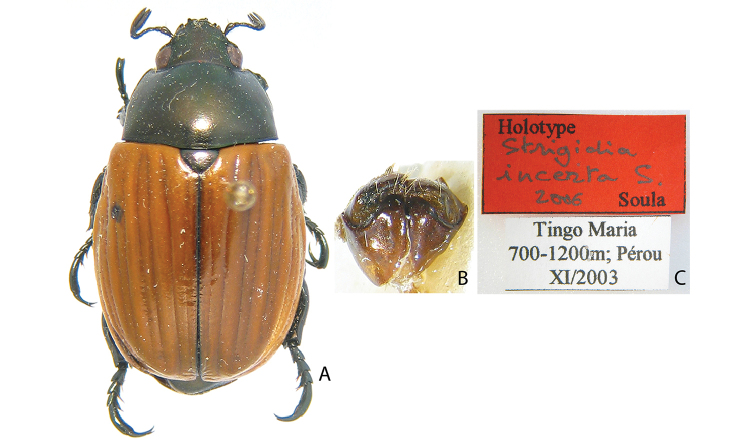
*Strigidia incerta* Soula holotype female from CCECL (valid name *Pelidnota incerta* (Soula)). **A** Dorsal habitus **B** Female gonocoxites, dorsal view **C** Specimen labels.

The following specimen is deposited at CCECL (Fig. 69). 1 ♀ holotype: “Tingo Maria 700-1200m; Pérou XI/2003//Holotype *Strigidia incerta* S. 2006 Soula” (47030123). Genitalia card-mounted underneath female holotype specimen. Box 1418652 SOULA.

### Pelidnota instabilis

Taxon classificationAnimaliaColeopteraScarabaeidae

Ohaus, 1912

Pelidnota instabilis Ohaus, 1912: 302–303 [original combination].
Pelidnota (Chalcoplethis) instabilis Ohaus [new subgeneric combination by Ohaus 1918: 28].
Strigidia instabilis (Ohaus) [new combination by Soula 2006: 64–65].
Pelidnota instabilis Ohaus [removal of subgeneric classification by Soula 2009: 115].

#### Distribution.

BRAZIL: Espírito Santo, Rio de Janeiro, São Paulo (Ohaus 1912, 1918, 1934b, Blackwelder 1944, Machatschke 1972, Soula 2006, Krajcik 2008).

#### Types.

1 ♂ syntype of *Pelidnota instabilis* is possibly at ZMHB, but this is unclear (Soula 2006).

### Pelidnota jalapensis

Taxon classificationAnimaliaColeopteraScarabaeidae

H. W. Bates, 1888

Pelidnota virescens var. jalapensis H. W. Bates, 1888: 275 [original combination].
Pelidnota (Pelidnota) virescens var. jalapensis H. W. Bates [new subgeneric combination by Ohaus 1918: 24].
Pelidnota (Pelidnota) virescens jalapensis H. W. Bates [new subspecific status by Machatschke 1972: 24].
Pelidnota (Pelidnota) jalapensis H. W. Bates [new species status by Hardy 1975: 7, 16].
Pelidnota jalapensis H. W. Bates [removal of subgeneric classification by Soula 2009: 66].

#### Distribution.

MEXICO: Guerrero, Oaxaca, Veracruz (H. W. Bates 1888, Ohaus 1918, 1934b, Machatschke 1972, Hardy 1975, Delgado-Castillo et al. 1988, Krajcik 2008, Soula 2009, Deloya et al. 2014).

#### Types.

1 ♂ lectotype at BMNH (Hardy 1975, Soula 2006); 1 paralectotype at BMNH (Soula 2006); 8 paralectotypes and 2 paralectotypes at MNHN. The following type specimens are deposited at CCECL. 2 ♂ Paralectotypes: “Jalapa, Mexico. Hoege//MUSEUM PARIS AMÉRIQUE CENTR. COLL OU BIOL CENTR AMÉR GODMAN 1908//*Pelidnota virescens* v. *jalapensis* Bts.//2008 *Pelidnota jalapensis* Bates M. SOULA det 19//Paralectotype 2008 Pelidnota virescens var. jalapensis Ba Soula det.” (47030485); “Jalapa, Mexico. Hoege.//H.W.Bates Biol.Cent.Amer.//2008 *Pelidnota jalapensis* Bates M. SOULA det 19//Paralectotype 2008 Pelidnota virescens var. jalapensis Ba. Soula det.” (47030486). Genitalia card-mounted underneath one male paralectotype. Box 4618666 SOULA.

### Pelidnota jolyi

Taxon classificationAnimaliaColeopteraScarabaeidae

Martínez, 1982

Pelidnota (Chalcoplethis) jolyi Martínez, 1982: 61–65 [original combination].
Strigidia jolyi (Martínez) [new combination by Soula 2006: 66–67].
Pelidnota jolyi Martínez [new combination by Soula 2009: 115].

#### Distribution.

BRAZIL: Acre (Martínez 1982, Soula 2006, Krajcik 2008). VENEZUELA: Bolívar (Martínez 1982, Soula 2006).

#### Types.

**Figure 70.**
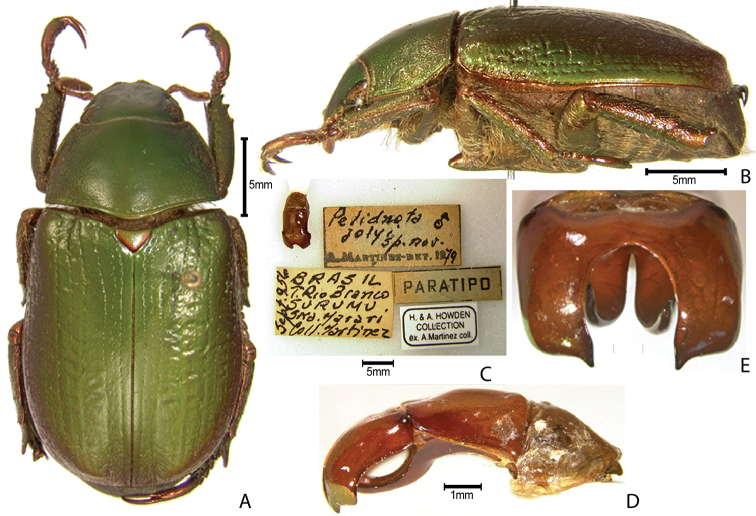
Pelidnota (Chalcoplethis) jolyi Martínez (valid name *Pelidnota jolyi* Martínez) paratype male from CMNC. **A** Dorsal habitus **B** Lateral habitus **C** Specimen labels and male genitalia **D** Male genitalia, lateral view **E** Male parameres, caudal view.

Holotype and allotype specimens of Pelidnota (Chalcoplethis) jolyi at MACN; 1 ♂ (Fig. 70) and 1 ♀ paratypes at CMNC.

### Pelidnota kirschi kirschi

Taxon classificationAnimaliaColeopteraScarabaeidae

F. Bates, 1904

Pelidnota kirschi F. Bates, 1904: 254, 261–262 [original combination].
Pelidnota (Chalcoplethis) kirschi F. Bates [new subgeneric combination by Ohaus 1918: 29].
Strigidia kirschi (F. Bates) [new combination by Soula 2006: 82–83].
Pelidnota kirschi F. Bates [revised combination by Soula 2009: 115].

#### Distribution.

COLOMBIA: Caldas, Cauca (F. Bates 1904, Ohaus 1918, 1934b, Blackwelder 1944, Machatschke 1972, Restrepo et al. 2003, Soula 2006, Krajcik 2008).

#### Types.

1 ♂ lectotype and 1 paralectotype of *Pelidnota kirschi* at BMNH (Soula 2006).

### Pelidnota kirschi tenuistriata

Taxon classificationAnimaliaColeopteraScarabaeidae

F. Bates, 1904

Pelidnota kirschi var. tenuistriata F. Bates, 1904: 254, 262 [original combination].
Pelidnota (Chalcoplethis) kirschi var. tenuistriata F. Bates [new subgeneric combination by Ohaus 1918: 29].
Pelidnota (Chalcoplethis) kirschi forma tenuistriata F. Bates [revised infrasubspecific status by Machatschke 1972: 32].
Strigidia kirschi tenuistriata (F. Bates) [new combination and new subspecific status by Soula 2006: 83–84].
Pelidnota kirschi tenuistriata F. Bates [revised combination by Soula 2009: 116].

#### Distribution.

VENEZUELA (F. Bates 1904, Ohaus 1918, 1934b, Blackwelder 1944, Machatschke 1972, Soula 2006).

#### Types.

**Figure 71.**
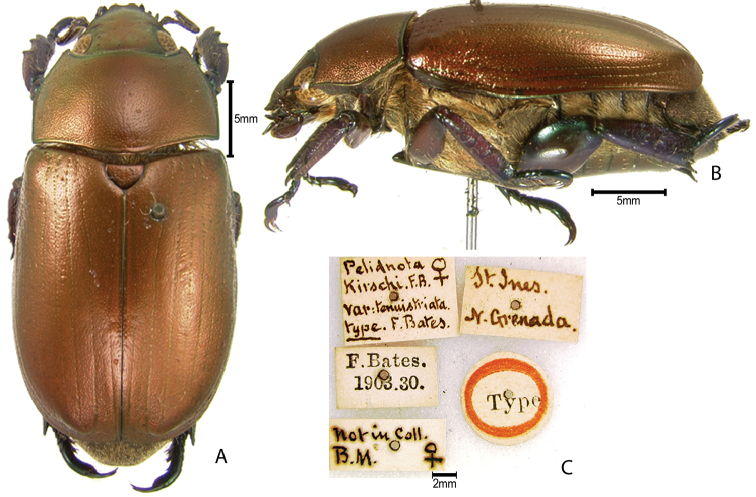
Pelidnota kirschi var. tenuistriata F. Bates (valid name *Pelidnota kirschi tenuistriata* F. Bates) holotype female from BMNH. **A** Dorsal habitus **B** Lateral habitus **C** Specimen labels.

1 ♀ holotype specimen of Pelidnota kirschi var. tenuistriata F. Bates at BMNH (Fig. 71).

### Pelidnota kuhnti

Taxon classificationAnimaliaColeopteraScarabaeidae

(Ohaus, 1912)

Heteropelidnota kuhnti Ohaus, 1912: 310–311 [original combination].
Pelidnota kuhnti (Ohaus) [**comb. n.**].

#### Distribution.

PARAGUAY: Paraguarí (Ohaus 1912, 1918, 1934b, Machatschke 1972, Krajcik 2008, Soula 2008).

#### Types.

**Figure 72.**
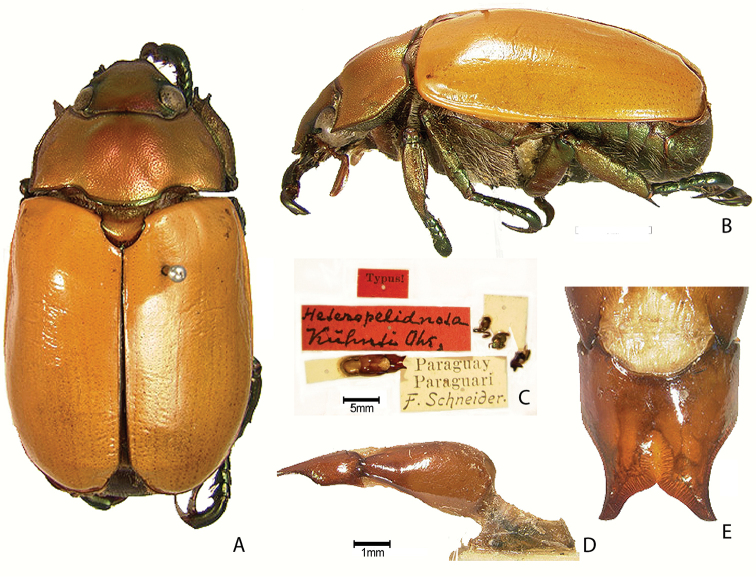
*Heteropelidnota kuhnti* Ohaus (valid name *Pelidnota kuhnti* [Ohaus]) holotype male from ZMHB. **A** Dorsal habitus **B** Lateral habitus **C** Specimen labels, mouthparts, and male genitalia **D** Male genitalia, lateral view **E** Male parameres, dorsal view.

Holotype ♂ at ZMHB (Fig. 72) with labels: a) “Paraguay Paraguari F. Schneider” (white label, typeset), b) male genitalia card-mounted, c) mouthparts cardmounted, d) “Typus!” (red label, typeset).

#### Remarks.

Ohaus (1912) described the genus *Heteropelidnota*, and in it he placed *H. kuhnti*. He compared *H. kuhnti* with P. aeruginosa var. citripennis (valid name *P. semiaurata citripennis*). He commented that the new genus was near *Hoplopelidnota*. Based on the original description, Ohaus (1912) had one male specimen given to him by Paul Kuhnt (for whom the species is dedicated).

The specimen on which the species is named appears to be a teratological deviant. The base of the pronotum is weakly triemarginate and surface sculpturing of pronotum appears weakly protuberant anterior to the emarginations. Additionally, the inner apices of the elytra are rounded, the apices of the meta- and mesotibia are eroded, the metacoxae and metafemur are quite gracile (about ½ the width of any pelidnotine scarab). The male genitalia are quite similar to *P. semiaurata citripennis* as well as the coloration, head, protibia, and protarsal claws. The apices of the elytra are poorly developed, thus exposing dense setae (one of the characters for which the genus was proposed). Other than the holotype specimen, no additional specimens are identified as *H. kuhnti*. It is possible that this specimen is a teratological deviant of *P. semiaurata citripennis*. Indeed, Soula (2008) also seemed to imply that *H. kuhnti* was a member of the genus *Pelidnota*. We synonymize the genus *Heteropelidnota* with *Pelidnota*. Lacking certainty of the species association due to the extreme deformities, we retain the species name and transfer the species to the genus *Pelidnota* as a **new combination**.

### Pelidnota labyrinthophallica

Taxon classificationAnimaliaColeopteraScarabaeidae

Solís & Morón, 1994

Pelidnota (Odontognathus) labyrinthophallica Solís & Morón, 1994: 31–35 [original combination].
Strigidia labyrinthophallica (Solís and Morón) [new combination by Soula 2006: 49–50].
Pelidnota (Strigidia) labyrinthophallica Solís and Morón [revised combination and new subgeneric combination by Özdikmen 2009: 145].
Pelidnota labyrinthophallica Solís and Morón [removal of subgeneric classfication by Soula 2009: 115].

#### Distribution.

COSTA RICA: Puntarenas (Solís and Morón 1994, Soula 2006, Krajcik 2008).

#### Types.

1 ♂ holotype, 1 ♀ allotype and 2 paratypes of Pelidnota (Odontognathus) labyrinthophallica at MNCR (Solís and Morón 1994); 3 paratypes at MXAL (Solís and Morón 1994); 1 ♂ and 1 ♀ paratypes at CMNC; 2 paratypes at ZMHB (Solís and Morón 1994).

#### Remarks.

Solís and Morón (1994) placed this species in the P. (Odontognathus) pulchella group based on distribution of members between Nicaragua, Brazil, and Peru. They commented that the species was similar to *P. glaberrima, P. xanthopyga*, and *P. belti*, but the form of the genitalia easily separated the new species. They also compared *P. labyrinthophallica* with *P. dubia* (“from Colombia”), but color and genitalia serve to separate the species. The etymology is derived from the Greek “labyrinthos” (=a tortuous passage) and “phallus” (=penis), alluding to the male genitalia of the species. They described the species from southwestern Costa Rica (Puntarenas Province) in Parque Nacional Corcovado and the Coto Brus region. Soula (2006) maintained species status of *P. labyrinthophallica*. Based on comparison of specimens (including type specimens), it is possible that *P. labyrinthophallica* is conspecific with *P. gabrielae*.

### Pelidnota lacazei

Taxon classificationAnimaliaColeopteraScarabaeidae

Soula, 2010

Pelidnota lacazei Soula, 2010a: 38 [original combination].

#### Distribution.

PERU: Junín (Soula 2010a, Ratcliffe et al. 2015).

#### Types.

The following specimens are deposited at CCECL. 1 ♂ holotype, 1 ♀ allotype, 8 ♂ paratypes, 1 ♀ paratype: “Satipo, Junin Pérou, X/2003//Holotype 2010 *Pelidnota lacazei* S. Soula” (47030179); “Satipo, Junin Pérou, X/2003//Allotype 2010 *Pelidnota lacazei* S. Soula” (47030180); Seven paratypes with identical labels “Satipo, Junin Pérou, X/2003 M. SOULA det 19//Paratype 2010 *Pelidnota lacazei* Soula” (47030181 to 47030186, exch09); “Satipo Pérou X-XI/2002//Paratype 2010 *Pelidnota lacazei* Soula” (47030188); “PERU Satipo VI.1989 Pres. by. [illegible] Perry B. M. 1989-258//Paratype 2010 *Pelidnota lacazei* Soula” (47030187). Genitalia card-mounted underneath the holotype and five male paratypes. Box 4618656 SOULA.

### Pelidnota laevissima

Taxon classificationAnimaliaColeopteraScarabaeidae

Burmeister, 1855

Pelidnota laevissima Burmeister, 1855: 522 [original combination].
Pelidnota (Pelidnota) laevissima Burmeister [new subgeneric combination by Ohaus 1918: 23].
Pelidnota laevissima Burmeister [removal of subgeneric classification by Soula 2009: 104].

#### Distribution.

COLOMBIA: Atlántico, Caldas, Valle del Cauca (Ohaus 1913, 1918, 1934b, Blackwelder 1944, Machatschke 1972, Restrepo et al. 2003). PANAMA: Chiriquí (Ohaus 1913). TRINIDAD & TOBAGO: Tobago, Trinidad (Ohaus 1913, 1918, 1934b, Blackwelder 1944, Machatschke 1972, Peck et al. 2002, Soula 2009). VENEZUELA: Distrito Capital (Burmeister 1855, Ohaus 1913, 1918, 1934b, Blackwelder 1944, Machatschke 1972, Hardy 1975, Krajcik 2008, Soula 2009).

#### Types.

1 paralectotype of *Pelidnota laevissima* at MLUH (Soula 2009). Soula (2009) stated that 1 ♀ holotype and 1 ♀ paratype resided at MNHN (see “*Type Specimens and Lectotype Designation*” in Methods).

#### Remarks.

Krajcik (2012, 2013) considered *P. cayennensis* and *P. chiriquicola* to be subspecies of *P. laevissima*.

### Pelidnota lagoi

Taxon classificationAnimaliaColeopteraScarabaeidae

Soula, 2011

Pelidnota lagoi Soula, 2011: 78–79 [original combination].

#### Distribution.

BRAZIL: Goiás (Soula 2011).

#### Types.

The holotype ♂ and allotype ♀ are deposited at the Malý collection (Soula 2011).

### Pelidnota langsdorffi

Taxon classificationAnimaliaColeopteraScarabaeidae

(Mannerheim, 1829)

Rutela langsdorffi Mannerheim, 1829: 48–49 [original combination].
Pelidnota langsdorffi (Mannerheim) [new combination by Burmeister 1844: 554].
Pelidnota (Pelidnota) langsdorffi (Mannerheim) [new subgeneric combination by Ohaus 1918: 25].
Pelidnota langsdorffi (Mannerheim) [removal of subgeneric classification by Soula 2009: 43].

#### Distribution.

BRAZIL (Mannerheim 1829, Burmeister 1844, Ohaus 1918, 1934b, Machatschke 1972, Soula 2009). FRENCH GUIANA: Cayenne (Harold 1869b).

### Pelidnota liturella assumpta

Taxon classificationAnimaliaColeopteraScarabaeidae

Ohaus, 1929

Pelidnota assumpta Ohaus, 1929: 388-389 [original combination].
Pelidnota (Ganonota) assumpta Ohaus [new subgeneric combination by Ohaus 1934b: 84].
Pelidnota (Strigidia) assumpta Ohaus [new subgeneric combination by Machatschke 1970: 157].
Pelidnota (Odontognathus) assumpta Ohaus [new subgeneric combination by Hardy 1975: 4].
Strigidia liturella assumpta (Ohaus) [new combination and new subspecific status by Soula 2006: 41–42].
Pelidnota (Strigidia) assumpta Ohaus [revised combination, revised species status, and revised subgeneric status by Özdikmen 2009: 145].
Pelidnota liturella assumpta Ohaus [removal of subgeneric classification and revised status by Soula 2009: 115].

#### Distribution.

BRAZIL: Minas Gerais (Soula 2006). PARAGUAY: Asunción (Ohaus 1929, 1934b, Blackwelder 1944, Machatschke 1972, Krajcik 2008).

#### Types.

**Figure 73.**
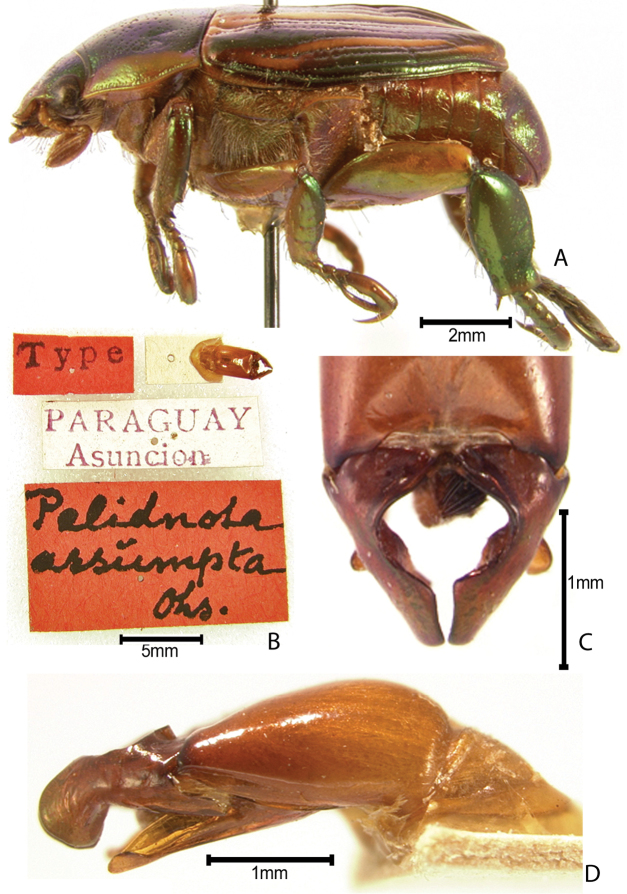
*Pelidnota assumpta* Ohaus (valid name *Pelidnota liturella assumpta* Ohaus) holotype male from ZMHB. **A** Lateral habitus **B** Specimen labels and male genitalia **C** Male parameres, dorsal view **D** Male genitalia, lateral view.

1 ♂ holotype of *Pelidnota assumpta* at ZMHB (Soula 2006) (Fig. 73).

### Pelidnota liturella liturella

Taxon classificationAnimaliaColeopteraScarabaeidae

(Kirby, 1819)

Rutela liturella Kirby, 1819: 406 [original combination].
Pelidnota liturella (Kirby) [new combination by MacLeay 1819: 155].
Pelidnota (Ganonota) liturella (Kirby) [new subgeneric combination by Ohaus 1918: 28].
Pelidnota (Strigidia) liturella (Kirby) [new subgeneric combination by Machatschke 1970: 157].
Pelidnota (Odontognathus) liturella (Kirby) [new subgeneric combination by Hardy 1975: 4].
Strigidia liturella (Kirby) [new combination by Soula 2006: 40–41].
Pelidnota (Strigidia) liturella (Kirby) [revised combination and revised subgeneric combination by Özdikmen 2009: 145].
Pelidnota liturella liturella (Kirby) [removal of subgeneric classification by Soula 2009: 115].

#### Distribution.

ARGENTINA: Misiones (Soula 2006). BRAZIL: Bahia, Espírito Santo, Goiás, Minas Gerais, Paraná, Rio de Janeiro, Rio Grande do Sul, Santa Catarina (Burmeister 1844, 1855, Blanchard 1851, Ohaus 1908a, 1918, 1929, 1934b, Machatschke 1972, Soula 2006, Krajcik 2008).

#### Types.

Most of Kirby’s type specimens are located at the BMNH. A search for the type specimen of *P. liturella* did not locate the specimen in the collection.

### Pelidnota louzadai

Taxon classificationAnimaliaColeopteraScarabaeidae

(Soula, 2006)

Strigidia louzadai Soula, 2006: 12, 55–56 [original combination].
Pelidnota louzadai (Soula) [new combination by Soula 2009: 115].

#### Distribution.

BRAZIL: Mato Grosso (Soula 2006).

#### Types.

The following specimens are deposited at CCECL. 1 ♂ holotype, 1 ♀ allotype: “BRASIL – MT Faz.Sao Tiago 12.35S-56.20W XI-81//Holotype 2006 *Strigidia louzadai* S. Soula” (47030437); “BRASIL – MT Faz.Sao Tiago 12.35S-56.20W XI-81//Allotype 2006 *Strigidia louzadai* S. Soula” (47030438). The genitalia are card-mounted underneath the male holotype. Box 4618663 SOULA.

### Pelidnota lucae

Taxon classificationAnimaliaColeopteraScarabaeidae

LeConte, 1863

Pelidnota lucae LeConte, 1863: 78 [original combination].
Pelidnota (Pelidnota) lucae LeConte [new subgeneric combination by Casey 1915: 76].
Pelidnota lucae LeConte [removal of subgeneric classification by Soula 2009: 50].

#### Distribution.

MEXICO: Baja California Norte, Baja California Sur (LeConte 1863, Casey 1915, Leng 1920, Ohaus 1918, 1934b, Blackwelder 1939, 1944, Machatschke 1972, Hardy 1975, 1991, Krajcik 2008, Soula 2009).

#### Types.

Syntype of *Pelidnota lucae* at MCZ (Hardy 1975).

### Pelidnota lucida

Taxon classificationAnimaliaColeopteraScarabaeidae

Burmeister, 1844

Pelidnota lucida Burmeister, 1844: 401 [original combination].
Pelidnota (Pelidnota) lucida Burmeister [new subgeneric combination by Ohaus 1918: 24].
Pelidnota lucida Burmeister [removal of subgeneric classification by Soula 2009: 47].

#### Distribution.

COLOMBIA (Burmeister 1844, Harold 1869b, Ohaus 1918, 1934b, Blackwelder 1944, Machatschke 1972, Restrepo et al. 2003, Krajcik 2008, Soula 2009). TRINIDAD & TOBAGO: Trinidad (Ohaus 1918, 1934b, Blackwelder 1944, Machatschke 1972). VENEZUELA (Ohaus 1918, 1934b, Blackwelder 1944, Machatschke 1972).

#### Types.

1 ♂ lectotype and 2 paralectotypes of *Pelidnota lucida* at MLUH (Soula 2009).

### Pelidnota lugubris

Taxon classificationAnimaliaColeopteraScarabaeidae

LeConte, 1874

Pelidnota lugubris LeConte, 1874: 54 [original combination].
Pelidnota (Pelidnota) lugubris LeConte [new subgeneric combination by Casey 1915: 76].
Pelidnota lugubris LeConte [removal of subgeneric classification by Soula 2009: 49].

#### Distribution.

MEXICO: Sinaloa, Sonora (Ohaus 1934b, Blackwelder 1944, Carrillo et al. 1966, Hardy 1975, 1991, Soula 2009, Lugo et al. 2014). USA: Arizona, New Mexico (LeConte 1874, Casey 1915, Leng 1920, Ohaus 1918, 1934b, Blackwelder 1939, 1944, Machatschke 1972, Hardy 1975, 1991, Krajcik 2008, Soula 2009).

#### Types.

1 syntype of *Pelidnota lugubris* at MCZ (Hardy 1975).

### Pelidnota luridipes

Taxon classificationAnimaliaColeopteraScarabaeidae

Blanchard, 1851

Pelidnota luridipes Blanchard, 1851: 212 [original combination].
Pelidnota (Pelidnota) luridipes Blanchard [new subgeneric combination by Ohaus 1918: 23].
Pelidnota luridipes Blanchard [removal of subgeneric classification by Soula 2009: 100].

#### Distribution.

BRAZIL: Mato Grosso (Blanchard 1851, Ohaus 1918, 1934b, Blackwelder 1944, Krajcik 2008, Soula 2009).

#### Types.

1 ♀ syntype at MNHN (Soula 2009).

#### Remarks.

CCECL contains a *P. luridipes* specimen labeled as a male alloréférent with the following data: 1 ♂ alloréférent: “Corumba Matt. Grosso//*P. luridipes* coll. – SOULA//Alloréférent ♂ de *Pelidnota luridipes* Bl. M. SOULA det 19” (47030640). Genitalia card-mounted underneath the male alloréférent. Box 4618678 SOULA.

### Pelidnota malyi

Taxon classificationAnimaliaColeopteraScarabaeidae

Soula, 2010

Pelidnota malyi Soula 2010a: 58 [original combination].
Pelidnota vladimalyi [new replacement name by Moore and Jameson 2013: 380].
Pelidnota malyi Soula [**revised status**].

#### Distribution.

ECUADOR: Imbabura (Soula 2010a).

#### Types.

**Figure 74.**
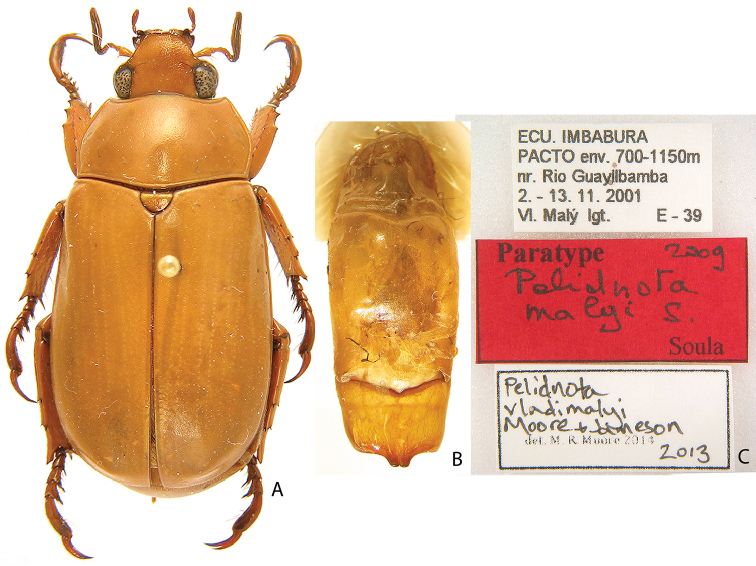
*Pelidnota malyi* Soula male paratype from CCECL. **A** Dorsal habitus **B** Male genitalia, dorsal view **C** Specimen labels.

The holotype ♂ is deposited at the Malý collection (Soula 2010a). The following specimens are deposited at CCECL. 2 ♂ paratypes with identical label data: “ECU. IMBABURA PACTO env. 700-1150m nr. Rio Guayllbamba 2. - 13. 11. 2001 Vl Malý lgt. E – 39//Paratype 2010 *Pelidnota malyi* S. Soula//*Pelidnota vladimalyi* Moore & Jameson det. M. R. Moore 2014 2013” (47030461 and 47030462) (Fig. 74). Genitalia card-mounted underneath the two male paratypes.

#### Remarks.

The species name *P. vladimalyi* was a replacement name for a homonym that Soula created by using the specific epithet “malyi” twice for two separate, distinct taxa in the genus *Pelidnota* (Moore and Jameson 2013), both of which are from Ecuador (not Peru and Ecuador, as stated in Moore and Jameson [2013]). The valid species *P. malyi* Soula (2010a: 36-37) was named for a metallic green species and *P. vladimalyi* is a testaceous species. In a slip of the pen, Soula used the specific epithet “vladislavmalyi” in reference to “malyi” described on page 58 (Soula 2010a). For this reason, “P. vladislavmalyi” was regarded as as an unavailable name (Moore and Jameson 2013). Soula later noted his error of homonymy (2011), and replaced this name with “P. vladislavmalyi Soula, 2011”. To avoid confusion and further nomenclatural stability, however, the name *P. vladimalyi* Moore and Jameson was proposed for *P. malyi* Soula, 2010a: 58 (Moore and Jameson 2013). However, because *Pelidnota malyi* Soula, 2010a: 36–37 is an unavailable name (see section on unavailable *Pelidnota* names), the replacement name *P. vladimalyi* is invalid. We correct this herein as *P. malyi* Soula, **revised status**.

### Pelidnota mantillerii

Taxon classificationAnimaliaColeopteraScarabaeidae

Soula, 2009

Pelidnota mantillerii Soula, 2009: 132 [original combination].

#### Distribution.

BRAZIL: Amazonas (Soula 2009).

#### Types.

The following specimen is deposited at CCECL. 1 ♂ holotype: “Taffé Amaz. Brésil M. Soula det. 20//Holotype *Pelidnota mantillerii* S. Soula” (47030128). Genitalia card-mounted underneath the holotype. Box 4618654 SOULA.

### Pelidnota matogrossensis

Taxon classificationAnimaliaColeopteraScarabaeidae

Frey, 1976

Pelidnota (Ganonota) matogrossensis Frey, 1976: 346 [original combination].
Strigidia matogrossensis (Frey) [new combination by Soula 2006: 38].
Pelidnota (Strigidia) matogrossensis Frey [revised combination and new subgeneric combination Özdikmen 2009: 145].
Pelidnota matogrossensis Frey [removal of subgeneric classification by Soula 2009: 115].

#### Distribution.

BOLIVIA: Santa Cruz (BMNH). BRAZIL: Mato Grosso (Frey 1976, Soula 2006, Krajcik 2008).

#### Types.

**Figure 75.**
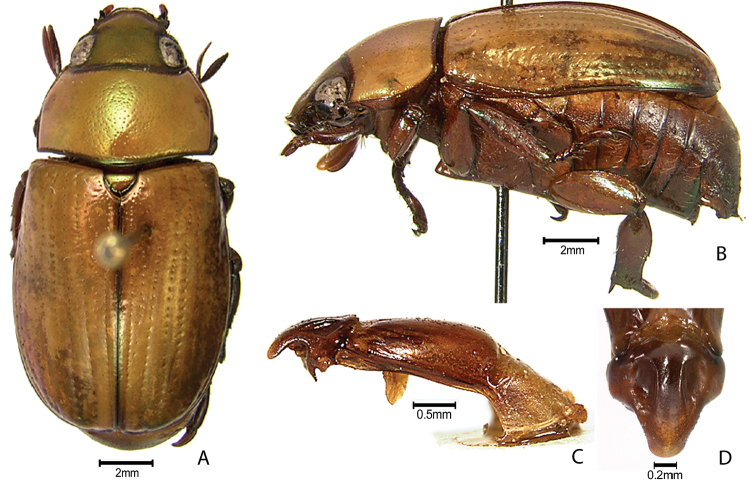
Pelidnota (Ganonota) matogrossensis Frey (valid name *Pelidnota matogrossensis* Frey) paratype male from ZMHB. **A** Dorsal habitus **B** Lateral habitus **C** Male genitalia, lateral view **D** Male parameres, dorsal view.

1 ♂ holotype and paratypes Pelidnota (Ganonota) matogrossensis at NHMB (Frey 1976, Soula 2006). 1 male paratype at ZMHB (Fig. 75).

### Pelidnota micobalaguerae micobalaguerae

Taxon classificationAnimaliaColeopteraScarabaeidae

(Soula, 2006)

Strigidia micobalaguerae Soula, 2006: 10, 82 [original combination].
Pelidnota micobalaguerae micobalaguerae (Soula) [new combination by Soula 2009: 115].

#### Distribution.

ECUADOR: Guayas, Napo (Soula 2006).

#### Types.

The following specimens are deposited at CCECL. 1 ♂ holotype, 5 ♂ paratypes, 1 ♀ paratype: “Cosanga Napo Ecuador 12/2005//Holotype 2006 *Strigidia micobalaguerae* S. Soula” (47030073); “Guacamayos Equateur M. SOULA det. 19//Paratype 2006 *Strigidia micobalaguerae* S. Soula” (47030074); “Antizana Guacamayos Napo Eq. M. SOULA det. 19 [obverse] V/2006//Paratype 2006 *Strigidia micobalaguerae* S. Soula” (47030075); “Lila [arrow] San Lorenzo coll. – SOULA [obverse] pk 7,5 770 m 15/08/93//Paratype 2006 *Strigidia micobalaguerae* S. Soula” (47030076); “ECUADOR OCCIDENTE GUAYAS Rte Machala-Guayaquil env. Naranjal niv. mer 29 janv. 79 Rec. Th. PORION//COLL. TH. PORION//Paratype 2006 *Strigidia micobalaguerae* S. Soula” (47030939); Two paratypes with identical label data: “ECUADOR OCCIDENTE GUAYAS Rte Machala-Guayaquil env. Naranjal niv. mer 29 janv. 79 Rec. Th. PORION//COLL. TH. PORION//Paratype *Pelidnota micobalaguerae*” (47030940 and 47030941). Genitalia card-mounted underneath the male holotype and four male paratypes. Box 4618650 SOULA and 4616343 PORION.

### Pelidnota micobalaguerae occidentalis

Taxon classificationAnimaliaColeopteraScarabaeidae

Soula, 2009

Pelidnota micobalaguerae occidentalis Soula, 2009: 133 [original combination].

#### Distribution.

ECUADOR: Cañar (Soula 2009).

#### Types.

The holotype male of *Pelidnota micobalaguerae occidentalis* is deposited in the Chichery Collection (Soula 2009).

### Pelidnota neitamorenoi neitamorenoi

Taxon classificationAnimaliaColeopteraScarabaeidae

(Soula, 2006)

Strigidia neitamorenoi Soula, 2006: 11, 50 [original combination].
Pelidnota neitamorenoi (Soula) [new combination by Soula 2009: 115].

#### Distribution.

BOLIVIA: La Paz (Soula 2006).

#### Types.

The following specimens are deposited at CCECL. 1 ♂ holotype, 1 ♀ allotype: “Caranavi 800m, 21/X/2000, M. SOULA det 19//Holotype 2005 *Strigidia neitmorenoi* (*sic*) Sou. Soula det.” (47030432); “Caranavi 800m, 21/X/2000, M. SOULA det 19//Allotype 2005 *Strigidia neitmorenoi* (*sic*) Sou. Soula det.” (47030433). The genitalia are card-mounted underneath the male holotype. Box 4618663 SOULA.

### Pelidnota neitamorenoi rodriguezdemendozaensis

Taxon classificationAnimaliaColeopteraScarabaeidae

Soula, 2010

Pelidnota neitamorenoi rodriguezdemendozaensis Soula, 2010a: 59 [original combination].

#### Distribution.

PERU: Amazonas (Soula 2010a, Ratcliffe et al. 2015).

#### Types.

The following specimen is deposited at CCECL. 1 ♂ holotype: “Rodriguez de Mendosa 1600m Col.Galic 07.76//Holotype 2010 *Pelidnota neitamorenoi* S. *rodriguezdemendozaensis* Soula” (47030427). Genitalia are card-mounted underneath the male holotype. Box 4618663 SOULA.

### Pelidnota nitescens

Taxon classificationAnimaliaColeopteraScarabaeidae

(Vigors, 1825)

Rutela nitescens Vigors, 1825: 417 [original combination].
Pelidnota nitescens (Vigors) [new combination by Burmeister 1844: 398].
Pelidnota (Ganonota) nitescens (Vigors) [new subgeneric combination by Ohaus 1918: 27].
Pelidnota (Strigidia) nitescens (Vigors) [new subgeneric combination by Machatschke 1970: 157].
Pelidnota (Odontognathus) nitescens (Vigors) [new subgeneric combination by Hardy 1975: 4].
Strigidia nitescens (Vigors) [new combination by Soula 2006: 35–36].
Pelidnota (Strigidia) nitescens (Vigors) [revised combination and revised subgeneric combination by Özdikmen 2009: 145].
Pelidnota nitescens (Vigors) [removal of subgeneric classification by Soula 2009: 115].
Rutela strigata (Mannerheim, 1829) **synonym.**
Rutela strigata Mannerheim, 1829: 50 [original combination].
Pelidnota nitescens (Vigors) [syn. by Burmeister 1844: 398].

#### Distribution.

BRAZIL: Minas Gerais, Paraná, São Paulo (Mannerheim 1829, Burmeister 1844, 1855, Blanchard 1851, Ohaus 1918, 1934b, Guimarães 1944, Blackwelder 1944, Machatschke 1972, Soula 2006, Krajcik 2008).

#### Types.

Holotype ♀ at BMNH with following labels: a) “Type” (round, white label with red circle, typeset), b) “5957 Vigors’ Coll.” (typeset), c) “Brazil” (typeset), d) “Rutela nitescens, Vigors type” (handwritten) and reverse side “identified from descriptions as Vigors’ type. GRA” (handwritten by Gilbert Arrow), e) “Rutela nitescens Vigors [female symbol] Det. Jameson 2000 Holotype (red label, printed and handwritten), f) “Holotype Rutela nitescens Vig. 2006 Soula” (red label, printed and handwritten), g) “Strigidia nitescens (Vig.) M. SOULA det. 19 2006” (white label, printed and handwritten). Soula (2006: 35) provided a photograph of the female syntype from the BMNH.

#### Remarks.

This distinctive species possesses striate, reddish-brown and black-striped elytra. Adults have been recorded feeding on leaves of *Psidium granifolium* Mart. ex DC. (Myrtaceae).

### Pelidnota notata

Taxon classificationAnimaliaColeopteraScarabaeidae

Blanchard, 1851

Pelidnota notata Blanchard, 1851: 212 [original combination].
Pelidnota (Pelidnota) notata Blanchard [new subgeneric combination by Ohaus 1918: 23].
Pelidnota notata Blanchard [removal of subgeneric classification by Soula 2009: 84].

#### Distribution.

BELIZE: Toledo (Hardy 1975, Morón et al. 1985). COLOMBIA: Boyacá, Chocó, Valle del Cauca (Ohaus 1918, 1934b, Blackwelder 1944, Machatschke 1972, Maes 1987, Restrepo et al. 2003, Neita-Moreno 2011). COSTA RICA: Alajuela, Cartago, Heredia, Limón, Puntarenas, San José (H. W. Bates 1888, Ohaus 1934b, Blackwelder 1944, Machatschke 1972, Hardy 1975, Morón et al. 1985, Maes 1987, Solís and Morón 1994, Soula 2009, García-López et al. 2013). ECUADOR: Cotopaxi, Esmeraldas, Guayas, Los Riós, Napo, Pichincha, Tungurahua (Ohaus 1908b, 1918, 1934b, Blackwelder 1944, Machatschke 1972, Hardy 1975, Morón 1979, Morón et al. 1985, Maes 1987, Paucar-Cabrera 2005, Soula 2009). GUATEMALA: Alto Verapaz, Izabal, Petén, Quetzaltenango, San Marcos (H. W. Bates 1888, Ohaus 1934b, Blackwelder 1944, Machatschke 1972, Hardy 1975, Morón 1979, Morón et al. 1985, Maes 1987). HONDURAS (Ohaus 1934b, Blackwelder 1944, Machatschke 1972, Maes 1987). MEXICO: Chiapas, Oaxaca, Tabasco, Veracruz (Blanchard 1851, H. W. Bates 1888, Ohaus 1908b, 1934b, Blackwelder 1944, Machatschke 1972, Hardy 1975, Morón 1979, Maes 1987, Palacios-Rios et al. 1990, Morón et al. 1985, 1997, Thomas 1993, Lobo and Morón 1993, Krajcik 2008, Soula 2009, Rivera-Gasperín et al. 2013). NICARAGUA: Chontales (H. W. Bates 1888, Ohaus 1934b, Blackwelder 1944, Machatschke 1972, Hardy 1975, Morón et al. 1985, Maes 1987). PANAMA: Bocas del Toro, Chiriquí, Colón, Former Canal Zone, Panama, Veraguas (H. W. Bates 1888, Ohaus 1908b, 1934b, Blackwelder 1944, Machatschke 1972, Hardy 1975, Morón et al. 1985, Maes 1987, Ratcliffe 2002, Soula 2009). VENEZUELA: Zulia (Hardy 1975, Morón 1979, Morón et al. 1985, Maes 1987).

#### Types.

1 ♀ syntype at MNHN (Soula 2009).

#### Remarks.

CCECL contains a *P. notata* specimen labeled as a male alloréférent with the following data: 1 ♂ alloréférent: “Oaxaca (M) 9/69 //Alloreferent ♂ de *Pelidnota notata* (Bl.) M. SOULA det. 19 2007”. Genitalia card-mounted underneath specimen. Box 4618664 SOULA.

### Pelidnota ohausi ohausi

Taxon classificationAnimaliaColeopteraScarabaeidae

Frey, 1976

Pelidnota (Ganonota) ohausi Frey, 1976: 345 [original combination].
Strigidia ohausi (Frey) [new combination by Soula 2006: 22].
Pelidnota (Strigidia) ohausi Frey [revised combination and revised subgeneric combination by Özdikmen 2009: 145].
Pelidnota ohausi ohausi Frey [removal of subgeneric classification by Soula 2009: 115].

#### Distribution.

BRAZIL: Mato Grosso (Frey 1976, Soula 2006, Krajcik 2008).

#### Types.

**Figure 76.**
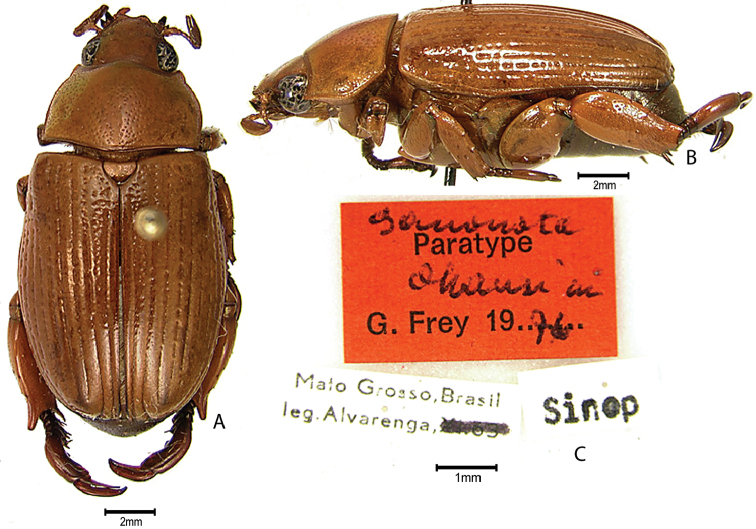
Pelidnota (Ganonota) ohausi Frey (valid name *Pelidnota ohausi ohausi* Frey) paratype from ZMHB. **A** Dorsal habitus **B** Lateral habitus **C** Specimen labels.

1 ♂ holotype and paratypes at NHMB (Frey 1976, Soula 2006). 1 ♂ paratype of Pelidnota (Ganonota) ohausi Frey at ZMHB (Fig. 76).

### Pelidnota ohausi piurensis

Taxon classificationAnimaliaColeopteraScarabaeidae

(Soula, 2006)

Strigidia ohausi piurensis Soula, 2006: 22 [original combination].
Pelidnota (Strigidia) ohausi piurensis (Soula) [new combination and new subgeneric combination by Özdikmen 2009: 145].
Pelidnota ohausi piurensis (Soula) [removal of subgeneric classification by Soula 2009: 115].

#### Distribution.

PERU: Piura (Soula 2006, Ratcliffe et al. 2015).

#### Types.

The following specimen is deposited at CCECL. 1 ♂ holotype: “Carabal, Rio Itaya Piura, Pérou, IX/2005//Holotype 2006 *Strigidia ohausi piurensis* Sou. Soula” (47030285). Genitalia card-mounted underneath male holotype. Box 4618658 SOULA.

### Pelidnota osculatii

Taxon classificationAnimaliaColeopteraScarabaeidae

Guérin-Méneville, 1855

Pelidnota osculatii Guérin-Méneville, 1855: 585 [original combination].
Pelidnota (Chalcoplethis) osculatii Guérin-Méneville [new subgeneric combination by Ohaus 1918: 29].
Strigidia osculatii (Guérin-Méneville) [new combination by Soula 2006: 68–69].
Pelidnota osculatii Guérin-Méneville [revised combination by Soula 2009: 115].

#### Distribution.

COLOMBIA: Boyacá, Cundinamarca (Ohaus 1912, Restrepo et al. 2003, Soula 2010a, López-García et al. 2015). ECUADOR: Morona-Santiago, Napo, Pastaza (Ohaus 1912, 1918, 1934b, Blackwelder 1944, Machatschke 1972, Paucar-Cabrera 2005, Soula 2010a). PERU: Loreto (Soula 2010a).

#### Types.

The following specimen is deposited at CCECL. 1 invalid ♂ neotype: “Iquitos, V/2002, M. SOULA det 19//Neotype *Pelidnota osculatii* Gué. 2010 Soula det.” (47030356). Genitalia card-mounted underneath invalid male neotype. Box 4618662 SOULA.

#### Remarks.

The original description of *P. osculatii* indicated that there were two specimens in the type series. Soula (2006) mentioned that a syntype (Soula incorrectly referred to a holotype) specimen of *P. osculatii* is from “versant oriental des Andes.” Soula (2006) stated that he was unable to find a type of *P. osculatii* at MNHN. While identifying type material from the Soula collection deposited at CCECL we discovered an invalidly designated neotype of *P. osculatii* (label details are provided above). This neotype designation was unpublished (i.e., it does not appear anywhere in the literature) and is thus invalid.

### Pelidnota pallidipennis

Taxon classificationAnimaliaColeopteraScarabaeidae

F. Bates, 1904

Pelidnota pallidipennis F. Bates, 1904: 258, 268–269 [original combination].
Pelidnota (Pelidnota) pallidipennis F. Bates [new subgeneric combination by Ohaus 1918: 23].
Pelidnota pallidipennis F. Bates [removal of subgeneric classification by Soula 2009: 103–104].

#### Distribution.

BRAZIL: Bahia, Goiás, Mato Grosso, Minas Gerais, Pernambuco, São Paulo (F. Bates 1904, Ohaus 1918, 1934b, Guimarães 1944, Blackwelder 1944, Machatschke 1972, Krajcik 2008, Soula 2009).

#### Types.

1 ♂ lectotype of *Pelidnota pallidipennis* at BMNH (Soula 2009).

### Pelidnota paraguayensis

Taxon classificationAnimaliaColeopteraScarabaeidae

F. Bates, 1904

Pelidnota paraguayensis F. Bates, 1904: 258, 266–267 [original combination].
Pelidnota (Pelidnota) fulva Blanchard [syn. by Ohaus 1918: 23].
Pelidnota paraguayensis F. Bates [revised species status by Soula 2009: 87–88].

#### Distribution.

PARAGUAY: Asunción (F. Bates 1904, Ohaus 1918, 1934b, Blackwelder 1944, Machatschke 1972, Krajcik 2008, Soula 2009).

#### Types.

1 ♂ lectotype of *Pelidnota paraguayensis* at BMNH (Soula 2009); 1 ♀ paralectotype at BMNH.

#### Remarks.

Krajcik (2012, 2013) omitted this name from his catalogs.

### Pelidnota parallela

Taxon classificationAnimaliaColeopteraScarabaeidae

Hardy, 1975

Pelidnota (Pelidnota) parallela Hardy, 1975: 6, 28–30 [original combination].
Pelidnota parallela Hardy [removal of subgeneric classification by Soula 2009: 52–53].

#### Distribution.

COLOMBIA: Chocó, Santander, Valle del Cauca (Hardy 1975, Soula 2009, Neita-Moreno 2011, López-García et al. 2015). COSTA RICA: Alajuela, Cartago, Guanacaste, Limón, Puntarenas (Hardy 1975, Solís and Morón 1994, Krajcik 2008, Soula 2009, García-López et al. 2010, García-López et al. 2013, López-García et al. 2015). PANAMA: Former Canal Zone (Hardy 1975, Ratcliffe 2002, Soula 2009, López-García et al. 2015).

#### Types.

1 ♂ holotype and 1 ♀ allotype of Pelidnota (Pelidnota) parallela at CNC (Hardy 1975); paratypes at CNC, CMNC, LACM and ZMHB (Hardy 1975).

### Pelidnota parvasedmagnifica

Taxon classificationAnimaliaColeopteraScarabaeidae

(Soula & Moragues, 2006)

Strigidia parvasedmagnifica Soula & Moragues, 2006: 12, 74–75 [original combination].
Pelidnota parvasedmagnifica (Soula and Moragues) [new combination by Soula 2009: 115].

#### Distribution.

FRENCH GUIANA (Soula 2006, 2010c).

#### Types.

The following specimens are deposited at CCECL. 1 ♂ holotype, 1 ♀ allotype, 11 ♂ paratypes, 15 ♀ paratypes: “Petit Saut pk 9 coll. – SOULA [obverse] 12/08/99//Holotype 2005 *Strigidia parvasedmagnifica* Sou. Soula det.” (47030235); “Piste DANGER Dd SARAMACA GUYANE FR 12 VIII 1988 [M. Duranton]//Allotype 2005 *Strigidia parvasedmagnifica* Sou. Soula det.” (47030236); Two paratypes with identical label data “GUYANE FRANCAISE Forêt de Tamanoir Pk 47 PL 7/8 IX 2002 M. DURANTON Coll.//Paratype 2006 *Strigidia parvasedmagnifica* S. Soula” (47030251, exch13); “GUYANE FRANCAISE Ft de Tamanoir Pk 49 PL 7/8 IX 2004 M & S DURANTON Coll.//Paratype 2006 *Strigidia parvasedmagnifica* S. Soula” (47030250); “GUYANE FRANCAISE Forêt de Tamanoir Pk 51 PL 23/24 VIII 2003 M & S DURANTON Coll.//Paratype 2006 *Strigidia parvasedmagnifica* Sou. Soula” (47030249); “GUYANE FRANCAISE Forêt de Patagaïe Pk 10 PL 10/11 VIII 2002 M. DURANTON Coll.//Paratype 2006 *Strigidia parvasedmagnifica* S. Soula” (47030252); “GUYANE FRANCAISE Forêt de Patagaïe Pk 10 PL 10/11 IX 2004 M & S DURANTON Coll.//Paratype 2006 *Strigidia parvasedmagnifica* S. Soula” (47030253); Three paratypes with identical label data “GUYANE FRANCAISE Saut Ananas Haute Mana 20/27 IX 1995 M DURANTON Coll.//Paratype 2006 *Strigidia parvasedmagnifica* S. Soula” (47030254, 47030255, exch14); “Guyane française M. SOULA det 19//Paratype 2006 *Strigidia parvasedmagnifica* S. Soula” (47030256); “Pte. de Kaw pk 37,5 3/III/98 M. SOULA det 19//Paratype 2006 *Strigidia parvasedmagnifica* S. Soula” (47030257); “Mgne de Singes Dd Saramaca Guyane Fr. 19 VII 1990//Paratype 2006 *Strigidia parvasedmagnifica* S. Soula” (47030243); “Mgne de Singes Dd Saramaca Guyane Fr. 19 VII 1990//Paratype 2006 *Strigidia parvasedmagnifica* Soul. Soula” (47030244); “Mgne des Singes Dd Saramaca GUYANE Fr. 19 VII 1990//Paratype Soula *Strigidia parvasedmagnifica* Sou. Soula” (exch15); “Dg. Saramaca G. F. 12/08/88 coll. – SOULA//Paratype 2005*Strigidia parvasedmagnifica* Sou. Soula det.” (47030248); “Dd Saramaca PK. Rte des Compagnons Guyane Fse 11.12 VIII 1990 M.Duranton Recolt.//Paratype 2005 *Strigidia parvasedmagnifica* S. Soula det.” (47030247); “Piste CORALIE RN2 GUYANE Fr. VIII 1990//Paratype 2006 *Strigidia parvasedmagnifica* S. Soula” (47030246); “Dd Saramaca PK. Rte des Compagnons Guyane Fse 26.27 IX 1984 M.Duranton Recolt.//Paratype 2006 *Strigidia parvasedmagnifica* S. Soula” (47030245); “CORALIE GUYANE Fr. 16.17 VIII 1990//Paratype 2006 *Strigidia parvasedmagnifica* S. Soula” (47030242); “Dd Saramaca Mgne des Singes 13 IX 1989 M.Duranton Recolt.//Paratype 2006 *Strigidia parvasedmagnifica* S. Soula” (47030241); “8.90 Piste S^t^ Elie Guyane//Paratype 2006 *Strigidia parvasedmagnifica* S. Soula” (47030240); “Petit Saut pk 9 12/08/99 coll. – SOULA//Paratype 2005 *Strigidia parvasedmagnifica* Sou. Soula det.” (47030239); “Dd Saramaca PK. Rte des Compagnons Guyane Fse 16.17 VIII 1985 M.Duranton Recolt.//Paratype 2006 *Strigidia parvasedmagnifica* S. Soula” (47030238); “Piste de BELIZON GUYANE FRSE VII 1994 D. CAMUS Leg//Paratype 2006 *Strigidia parvasedmagnifica* S. Soula” (47030237); “GUYANE F. St-Jean/Maroni 5.2.78 PORION//COLL. TH. PORION//Paratype 2006 *Strigidia parvasedmagnifica* S. Soula” (47030943); “GUYANE F. St-Jean/Maroni 2.I.78 PORION//COLL. TH. PORION//Paratype 2006 *Strigidia parvasedmagnifica* S. Soula” (47030944). Genitalia card-mounted underneath the male holotype and nine male paratypes. Box 4618657 SOULA and 4616343 PORION.

### Pelidnota pennata

Taxon classificationAnimaliaColeopteraScarabaeidae

Ohaus, 1912

Pelidnota pennata Ohaus, 1912: 298, 299–300 [original combination].
Pelidnota (Chalcoplethis) pennata Ohaus [new subgeneric combination by Ohaus 1918: 29].
Strigidia pennata (Ohaus) [new combination by Soula 2006: 69–70].
Pelidnota pennata Ohaus [revised combination by Soula 2009: 115].

#### Distribution.

BRAZIL: Amazonas, Pará (Ohaus 1912, 1918, 1934b, Blackwelder 1944, Machatschke 1972, Hardy 1975, Krajcik 2008, Soula 2006, 2010a). NICARAGUA: Managua (Hardy 1975, Soula 2006). PANAMA: Chiriquí (Ratcliffe 2002).

#### Types.

1 ♂ lectotype of *Pelidnota pennata* at ZMHB (Hardy 1975, Soula 2006); paralectotypes at ZMHB (Soula 2006).

### Pelidnota perplexa

Taxon classificationAnimaliaColeopteraScarabaeidae

Hardy, 1975

Pelidnota (Pelidnota) perplexa Hardy, 1975: 7, 15–16 [original combination].
Pelidnota perplexa Hardy [removal of subgeneric classification by Soula 2009: 69–70].

#### Distribution.

MEXICO: Hidalgo, Nuevo León, San Luis Potosí, Veracruz (Hardy 1975, Morón 1993, 1994, Morón et al. 1997, Delgado-Castillo and Márquez 2006, Krajcik 2008, Soula 2009).

#### Types.

**Figure 77.**
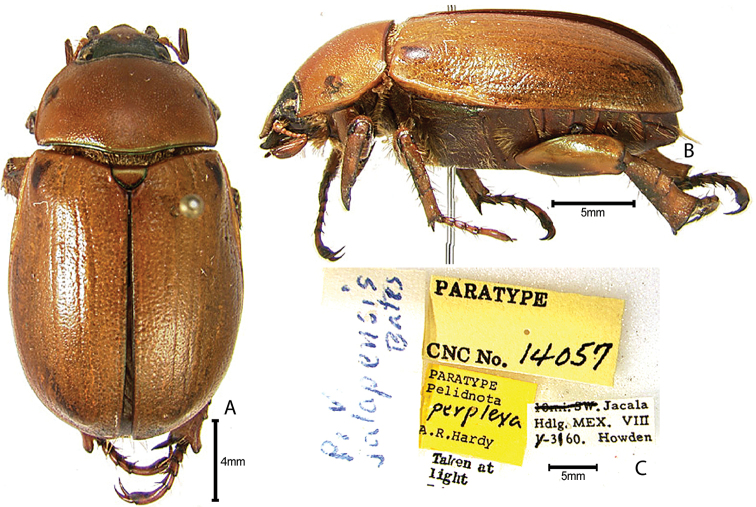
Pelidnota (Pelidnota) perplexa Hardy (valid name *Pelidnota perplexa* Hardy) paratype female from CNC. **A** Dorsal habitus **B** Lateral habitus **C** Specimen labels.

1 ♂ holotype of Pelidnota (Pelidnota) perplexa at USNM (Hardy 1975); 1 ♀ allotype and 1 paratype at CAS (Hardy 1975); 2 paratypes at CNC (Fig. 77) (Hardy 1975).

### Pelidnota peslieri

Taxon classificationAnimaliaColeopteraScarabaeidae

Soula, 2009

Pelidnota peslieri Soula, 2009: 133 [original combination]

#### Distribution.

PERU: Loreto (Soula 2009, Ratcliffe et al. 2015).

#### Types.

The following specimen is deposited at CCECL. 1 ♂ holotype: “Iquitos; Loreto Pérou ; XI-XII/2004//Holotype 2008 *Strigidia peslieri* S. Soula” (47030127). Genitalia mounted underneath the holotype. Box 4618654 SOULA.

### Pelidnota polita cupritarsis

Taxon classificationAnimaliaColeopteraScarabaeidae

H. W. Bates, 1888

Pelidnota cupritarsis H. W. Bates, 1888: 275 [original combination].
Pelidnota lucida Burmeister [syn. by F. Bates 1904: 257].
Pelidnota (Pelidnota) polita (Latreille) [syn. by Ohaus 1918: 24–25].
Pelidnota polita cupritarsis H. W. Bates [removal of subgeneric classification and new subspecific status by Soula 2009: 45–46].

#### Distribution.

COLOMBIA (H. W. Bates 1888, Krajcik 2008, Soula 2009). PANAMA (H. W. Bates 1888, Ohaus 1934b, Machatschke 1972, Hardy 1975, Krajcik 2008).

#### Types.

1 ♀ lectotype and 1 paralectotype of *Pelidnota cupritarsis* at MNHN (Soula 2009).

#### Remarks.

This subspecies is apparently sympatric with the nominal subspecies. The validity of the taxon should be addressed in future studies.

### Pelidnota polita polita

Taxon classificationAnimaliaColeopteraScarabaeidae

(Latreille, 1812)

Rutela polita Latreille, 1812: 134 [original combination].
Pelidnota polita (Latreille) [new combination by Burmeister 1844: 552].
Pelidnota (Pelidnota) polita (Latreille) [new subgeneric combination by Ohaus 1918: 24–25].
Pelidnota polita polita (Latreille) [removal of subgeneric classification and new subspecific status by Soula 2009: 44–45].

#### Distribution.

BRAZIL (Ohaus 1918, 1934b, Machatschke 1972). COLOMBIA: Atlántico, Boyacá, Chocó, Cundinamarca, Magdalena, Meta (WBWC) (Ohaus 1918, 1934b, Machatschke 1972, Restrepo et al. 2003, Neita-Moreno 2011, García-Atencia and Martínez-Hernández 2015, López-García et al. 2015). PANAMA: Colón, Former Canal Zone, Oeste (Ohaus 1918, 1934b, Blackwelder 1944, Machatschke 1972, Hardy 1975, Ratcliffe 2002, López-García et al. 2015). PERU (Latreille 1812, Germar 1815, Ohaus 1918, 1934b, Machatschke 1972, Hardy 1975, Krajcik 2008, Soula 2009, Ratcliffe et al. 2015). VENEZUELA (Ohaus 1918, 1934b, Machatschke 1972, López-García et al. 2015).

#### Types.

1 ♂ neotype of *Rutela polita* at MNHN (Soula 2009).

### Pelidnota porioni

Taxon classificationAnimaliaColeopteraScarabaeidae

Soula, 2010

Pelidnota porioni Soula, 2010a: 59-60 [original combination].

#### Distribution.

PERU: Cusco (Soula 2010a, Ratcliffe et al. 2015).

#### Types.

The following specimens are deposited at CCECL. 1 ♂ holotype, 1 ♀ allotype, 1 ♀ paratype: “Pérou Cuzco Rte Cuzco Manu K 171 700 m 12/14-XII-79 T. Porion leg.//Holotype *Pelidnota porioni* S. 2009 Soula” (47030945). “Pérou Cuzco Rte Cuzco Manu K 160 1200 m 9/12-XII-79 T. Porion leg.//Allotype *Pelidnota porioni* S. 2009 Soula” (47030946); “Pérou Cuzco Rte Cuzco Manu K 151 1650 m 15/18-XII-79 T. Porion leg.//Paratype *Pelidnota porioni* S. 2009 Soula” (47030947). Genitalia card-mounted underneath the male holotype. Box 4616343 PORION.

### Pelidnota prasina

Taxon classificationAnimaliaColeopteraScarabaeidae

Burmeister, 1844

Pelidnota prasina Burmeister, 1844: 402–403 [original combination].
Pelidnota (Pelidnota) prasina Burmeister [new subgeneric combination by Ohaus 1918: 23].
Pelidnota prasina Burmeister [removal of subgeneric classification by Soula 2009: 95].

#### Distribution.

BRAZIL: Rondônia (WBWC). COLOMBIA: Antioquia, Boyacá, Cauca, Caquetá, Casanare, Cundinamarca, Meta, Risaralda, Santander, Tolima, Valle del Cauca (Burmeister 1844, Ohaus 1918, 1934b, Machatschke 1972, Restrepo et al. 2003, Krajcik 2008, Soula 2009, Pardo-Locarno et al. 2005, 2011, Torres Martínez and Guevara Correal 2012, Pardo-Locarno 2013, López-García et al. 2015). ECUADOR: Napo, Pastaza, Pichincha, Tungurahua, Zamora Chinchipe (Paucar-Cabrera 2005). PERU: Leoncio Prado (WBWC). VENEZUELA (Ohaus 1918, 1934b, Machatschke 1972).

#### Types.

1 ♂ lectotype and 2 paralectotypes of *Pelidnota prasina* at MLUH (Soula 2009).

### Pelidnota prolixa

Taxon classificationAnimaliaColeopteraScarabaeidae

Sharp, 1877

Pelidnota prolixa Sharp, 1877: 132–133 [original combination].
Pelidnota (Pelidnota) prolixa Sharp [new subgeneric combination by Ohaus 1918: 23].
Pelidnota prolixa Sharp [removal of subgeneric classification by Soula 2009: 51].

#### Distribution.

COLOMBIA: Chocó, Valle del Cauca (Hardy 1975, Restrepo et al. 2003, Soula 2009, Neita-Moreno 2011). COSTA RICA: Limón, Puntarenas (Hardy 1975, Solís and Morón 1994, Soula 2009). ECUADOR: Esmeraldas, Napo, Pichincha (Ohaus 1918, 1934b, Machatschke 1972, Hardy 1975, Maes 1987, Paucar-Cabrera 2005, Soula 2009). NICARAGUA: Chontales (Ohaus 1918, 1934b, Machatschke 1972, Hardy 1975, Maes 1987, Krajcik 2008, Soula 2009). PANAMA: Chiriquí, Former Canal Zone (H. W. Bates 1888, Ohaus 1918, 1934b, Machatschke 1972, Hardy 1975, Maes 1987, Ratcliffe 2002, Soula 2009).

#### Types.

1 ♀ lectotype of *Pelidnota prolixa* at BMNH (Hardy 1975). Soula (2009) stated that 1 ♀ holotype and 1 ♀ paratype resided at MNHN (see “*Type Specimens and Lectotype Designation*” in Methods).

### Pelidnota pulchella altoparanaensis

Taxon classificationAnimaliaColeopteraScarabaeidae

(Soula, 2006)

Strigidia pulchella altoparanaensis Soula, 2006: 29 [original combination].
Pelidnota (Strigidia) pulchella altoparanaensis (Soula) [new combination and new subgeneric combination by Özdikmen 2009: 145].
Pelidnota pulchella altoparanaensis (Soula) [removal of subgeneric classification by Soula 2009: 115].

#### Distribution.

PARAGUAY (Soula 2006).

#### Types.

The following specimens are deposited at CCECL. 1 ♂ holotype, 1 ♀ allotype, 3 ♀ paratypes: “H^t^ Parana Paraguay III 2005 M. Soula det 19//Holotype 2006 *Strigidia pulchella altoparanaensis* S. Soula.” (47030313); “H^t^ Parana Paraguay III/2005 M. Soula det 19//Allotype 2006 *Strigidia pulchella altoparanaensis* S. Soula.” (47030314); Three paratypes with identical label data “H^t^ Parana Paraguay III/2005 M. Soula det 19//Paratype 2006 *Strigidia pulchella altoparanaensis* S. Soula.” (47030315 to 47030317). Genitalia card-mounted underneath the male holotype and the female allotype. Box 4618660 SOULA.

### Pelidnota pulchella pulchella

Taxon classificationAnimaliaColeopteraScarabaeidae

(Kirby, 1819)

Rutela pulchella Kirby, 1819: 405–406 [original combination].
Pelidnota pulchella (Kirby) [new combination by MacLeay 1819: 154].
Rutela pulchella (Kirby) [new combination by Kirby 1824: 118].
Pelidnota pulchella (Kirby) [revised combination by Burmeister 1844: 394–395].
Pelidnota (Ganonota) pulchella (Kirby) [new subgeneric combination by Ohaus 1918: 26].
Pelidnota (Strigidia) pulchella (Kirby) [new subgeneric combination by Machatschke 1970: 157].
Pelidnota (Odontognathus) pulchella (Kirby) [new subgeneric combination by Hardy 1975: 4].
Strigidia pulchella (Kirby) [new combination by Soula 2006: 27–29].
Pelidnota (Strigidia) pulchella (Kirby) [revised combination and revised subgeneric combination by Özdikmen 2009: 145].
Pelidnota pulchella pulchella (Kirby) [removal of subgeneric classification by Soula 2009: 115].
Pelidnota pulchella blanda Burmeister, 1844 **synonym.**
Pelidnota pulchella var. blanda Burmeister, 1844: 394 [original combination, name is available as a subspecies per ICZN Article 45.6.4].
Pelidnota pulchella forma blanda Burmeister [revised infrasubspecific status by Machatschke 1972: 27].
Pelidnota pulchella var. blanda Burmeister [revised infrasubspecific status by Soula 2006: 28].
Pelidnota pulchella pulchella (Kirby) [**syn. n.**].
Pelidnota pulchella scapularis Burmeister, 1844 **synonym.**
Pelidnota pulchella var. scapularis Burmeister, 1844: 394 [original combination, name is available as a subspecies per ICZN Article 45.6.4].
Pelidnota pulchella forma scapularis Burmeister [revised infrasubspecific status by Machatschke 1972: 27].
Pelidnota pulchella var. scapularis Burmeister [revised infrasubspecific status by Soula 2006: 28].
Pelidnota pulchella pulchella (Kirby) [**syn. n.**].
Pelidnota xanthogramma Perty, 1830 **synonym.**
Pelidnota xanthogramma Perty, 1830: 49 [original combination].
Pelidnota pulchella var. xanthogramma Perty [new infrasubspecific status by Burmeister 1844: 394].
Pelidnota pulchella forma xanthogramma Perty [revised infrasubspecific status by Machatschke 1972: 27].
Pelidnota pulchella var. xanthogramma Perty [revised infrasubspecific status by Soula 2006: 28].
Pelidnota pulchella pulchella (Kirby) [**syn. n.**].

#### Distribution.

ARGENTINA (Soula 2006). BRAZIL: Espírito Santo, Minas Gerais, Rio de Janeiro, Rio Grande do Sul, Santa Catarina, São Paulo (Kirby 1824, Perty 1830, Laporte 1840, Burmeister 1844, 1855, Blanchard 1851, Ohaus 1913, 1918, 1934b, Guimarães 1944, Machatschke 1972, Soula 2006, Krajcik 2008). PARAGUAY: Caaguazú (Dreschsel 2014), Guairá (WBWC).

#### Types.

**Figure 78.**
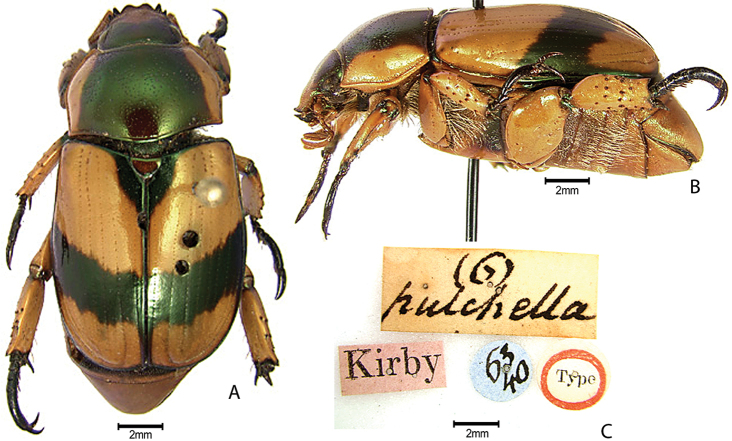
*Rutela pulchella* Kirby (valid name *Pelidnota pulchella pulchella* [Kirby]) syntype female from BMNH. **A** Dorsal habitus **B** Lateral habitus **C** Specimen labels.

Syntype ♀ of *Rutela pulchella* at BMNH (Fig. 78).

#### Remarks.

Kirby (1819) described *Rutela puchella* from “Brasilia” based on specimen(s) from “D. Hancock”, and J. Curtis provided illustrations of the overall body form, antenna, labrum, mentum, mandible, and maxilla (table 21, fig. 10, p. 479). Based on Raphael (1970) this work was published in 1819 rather than 1818. Female specimens of *P. pulchella* have a narrower horizontal elytral band (mid-elytra to ¾ length of elytra), whereas males have a broader horizontal elytral band (mid elytra to apex or near apex). Some varieties may have been named based on this sexually dimorphic trait. Soula (2006) stated that the species is distributed in the Atlantic forest, north to Argentina and the state of Espírito Santo, and he named a new subspecies, *P. pulchella altoparanaensis*, for a “little series from Paraguay” (Soula 2006).

Three names were proposed by Ohaus (1913) as infrasubspecific taxa of *Pelidnota pulchella pulchella*. These names are unavailable as per ICZN Article 45.6 (see Moore and Jameson 2013 for interpretation of Ohaus’s [1913] varieties). Ohaus (1913) names and describes both subspecies and infrasubspecific entities, even within the same genus (e.g., *Homonyx*). Thus, the following unambiguously infrasubspecific names were proposed by Ohaus (1913: 501, 502): Pelidnota pulchella var. fulvopunctata (misspelled as *fuscopunctata* in Machatschke [1972: 27]), P. pulchella var. sellata, and P. pulchella var. reducta. These names have never been treated as subspecific and were maintained as infrasubspecific entities (var. or forma) in later catalogs (Ohaus 1918, 1934b, Machatschke 1972). Because these Ohaus (1913) names were not used as valid species or subspecies nor treated as senior homonyms before 1985 we consider them **unavailable**: Pelidnota pulchella var. fulvopunctata Ohaus (**unavailable name**) (type female at ZMHB labeled: “*P. pulchella* Kirby v. *fulvopunctata* Ohaus”), P. pulchella var. sellata Ohaus (**unavailable name**) (type female at ZMHB labeled: “*P. pulchella* Kirby v. *sellata* Ohaus”), and P. pulchella var. reducta Ohaus (**unavailable name**) (type female at ZMHB labeled: “*P. pulchella* Kirby v. *reducta* Ohaus”).

Two “varieties” of *P. pulchella pulchella* were described by Burmeister (1844: 394): Pelidnota pulchella var. blanda and Pelidnota pulchella var. scapularis. These names are ambiguously infrasubspecific because Burmeister (1844) did not expressly designate them as such and the rest of the work does not discuss subspecies as separate entities from varieties. ICZN Article 45.6.4 is applied here and these two Burmeister (1844) names should be considered available and subspecific from their original description. Burmeister (1844) also treated *Pelidnota xanthogramma* Perty (an available name originally proposed as a species) as a variety of *P. pulchella*. These three names were treated as infrasubspecific (var. or forma) by subsequent authors (Ohaus 1913, 1918, Machatschke 1972, Soula 2006) to circumscribe dorsal and ventral color variation in *P. pulchella* (see Soula [2006: 29] for images of *P. pulchella* color variation). These three available names were later listed as synonyms of *P. pulchella pulchella* by Krajcik (2008). Because we do not consider Krajcik’s (2008, 2012, 2013) checklists of world scarabaeoids to contain authoritative taxonomic decisions and we do not recognize infrasubspecific entities, we formally list the following taxa in synonymy with *P. pulchella pulchella* (Kirby): Pelidnota pulchella var. scapularis Burmeister **syn. n.**, Pelidnota pulchella var. blanda Burmeister **syn. n.**, and *Pelidnota xanthogramma* Perty **syn. n.**.

### Pelidnota punctata

Taxon classificationAnimaliaColeopteraScarabaeidae

(Linnaeus, 1758)

Scarabaeus punctatus Linnaeus, 1758: 350 [original combination].
Melolontha punctata (Linnaeus) [new combination by Fabricius 1775: 33].
Rutela punctata (Linnaeus) [new combination by Latreille 1802: 151].
Pelidnota punctata (Linnaeus) [new combination by MacLeay 1819: 154].
Pelidnota (Pelidnota) punctata (Linnaeus) [new subgeneric combination by Casey 1915: 71].
Pelidnota punctata (Linnaeus) [removal of subgeneric classification by Soula 2009: 84].
Pelidnota francoisgenieri Moore & Jameson, 2013 [**syn. n.**]. **synonym.**
Pelidnota genieri Soula 2009: 32, 81–82 [original combination and junior secondary homonym]. **synonym.**
Pelidnota francoisgenieri [replacement name by Moore and Jameson 2013: 379–380].
Pelidnota punctata (Linnaeus) [**syn. n.**].
Melolontha lutea Olivier, 1789 **synonym.**
Melolontha lutea Olivier, 1789: 23 [original combination].
Pelidnota punctata (Linnaeus) [syn. by Burmeister 1844: 400].
Pelidnota (Pelidnota) lutea (Olivier) [new subgeneric combination and revised species status by Casey 1915: 73].
Pelidnota (Pelidnota) punctata lutea (Olivier) [new subspecific status by Ohaus 1918: 24].
Pelidnota (Pelidnota) lutea (Olivier) [revised species status by Ohaus 1934b: 80].
Pelidnota (Pelidnota) punctata (Linnaeus) [syn. by Hardy 1974: 89].
Pelidnota lutea (Olivier) [removal of subgeneric classification and revised species status by Soula 2009: 82].
Pelidnota punctata (Linnaeus) [**revised synonymy**].
Pelidnota (Pelidnota) lutea brevicollis Casey, 1915 **synonym.**
Pelidnota (Pelidnota) lutea brevicollis Casey, 1915: 74 [original combination].
Pelidnota (Pelidnota) punctata (Linnaeus) [syn. by Hardy 1974: 89].
Pelidnota (Pelidnota) lutea hudsonica Casey, 1915 **synonym.**
Pelidnota (Pelidnota) lutea hudsonica Casey, 1915: 74 [original combination].
Pelidnota (Pelidnota) punctata (Linnaeus) [syn. by Hardy 1974: 89].
Pelidnota (Pelidnota) lutea pallidipes Casey, 1915 **synonym.**
Pelidnota (Pelidnota) lutea pallidipes Casey, 1915: 74 [original combination].
Pelidnota (Pelidnota) punctata (Linnaeus) [syn. by Hardy 1974: 89].
Pelidnota (Pelidnota) oblonga debiliceps Casey, 1915 **synonym.**
Pelidnota (Pelidnota) oblonga debiliceps Casey, 1915: 73 [original combination].
Pelidnota (Pelidnota) punctata (Linnaeus) [syn. by Hardy 1974: 89].
Pelidnota (Pelidnota) oblonga oblonga Casey, 1915 **synonym.**
Pelidnota (Pelidnota) oblonga oblonga Casey, 1915: 72-73 [original combination].
Pelidnota (Pelidnota) punctata (Linnaeus) [syn. by Hardy 1974: 89].
Pelidnota (Pelidnota) oblonga ponderella Casey, 1915 **synonym.**
Pelidnota (Pelidnota) oblonga ponderella Casey, 1915: 73 [original combination].
Pelidnota (Pelidnota) punctata (Linnaeus) [syn. by Hardy 1974: 89].
Pelidnota (Pelidnota) punctata brevis Casey, 1915 **synonym.**
Pelidnota (Pelidnota) punctata brevis Casey, 1915: 72 [original combination].
Pelidnota (Pelidnota) punctata (Linnaeus) [syn. by Hardy 1974: 89].
Pelidnota (Pelidnota) punctata strenua Casey, 1915 **synonym.**
Pelidnota (Pelidnota) punctata strenua Casey, 1915: 72 [original combination].
Pelidnota (Pelidnota) punctata (Linnaeus) [syn. by Hardy 1974: 89].
Pelidnota (Pelidnota) tarsalis Casey, 1915 **synonym.**
Pelidnota (Pelidnota) tarsalis Casey, 1915: 74 [original combination].
Pelidnota (Pelidnota) punctata (Linnaeus) [syn. by Hardy 1974: 89].
Pelidnota (Pelidnota) lutea texensis Casey, 1915 **synonym.**
Pelidnota (Pelidnota) lutea texensis Casey, 1915: 74 [original combination].
Pelidnota (Pelidnota) punctata (Linnaeus) [syn. by Hardy 1974: 89].
Pelidnota texensis Casey [removal of subgeneric classification and new species status by Soula 2009: 83].
Pelidnota punctata (Linnaeus) [**revised synonymy**].

#### Distribution.

CANADA: Manitoba, Ontario, Quebec (Burmeister 1844, Wickham 1894, Ohaus 1918, 1934b, Roberts 1946, 1962, Hicks 1965, Hardy 1985, 1991, Soula 2009, Moore and Jameson 2013).

USA: Alabama, Arkansas, Arizona, Connecticut, Delaware, District of Columbia, Florida, Georgia, Illinois, Indiana, Iowa, Kansas, Kentucky, Louisiana, Maine, Maryland, Massachusetts, Michigan, Mississippi, Missouri, Nebraska, New Hampshire, New Jersey, New York, North Carolina, Ohio, Oklahoma, Pennsylvania, Rhode Island, South Carolina, South Dakota, Tennessee, Texas, Virginia, West Virginia, Wisconsin (Laporte 1840, Burmeister 1844, Blanchard 1851, Bubna 1902, King 1914, Casey 1915, Leng 1920, Ortenburger and Hatch 1926, Hayes 1928, 1929, Hayes and McColloch 1928, Ohaus 1918, 1934b, Montgomery and Amos 1940, Blackwelder 1939, 1944, Landin 1956, Gibson and Carrillo 1959, Carrillo et al. 1966, Machatschke 1972, Kirk and Balsbaugh 1975, Morrill 1979, Hardy 1975, 1985, 1991, McNamara 1991, Peck and Thomas 1998, Harpootlian 2001, Kriska and Young 2002, Buss 2006, Ratcliffe and Paulsen 2008, Soula 2009, Chong and Hinton 2015).

#### Types.

**Figure 79.**
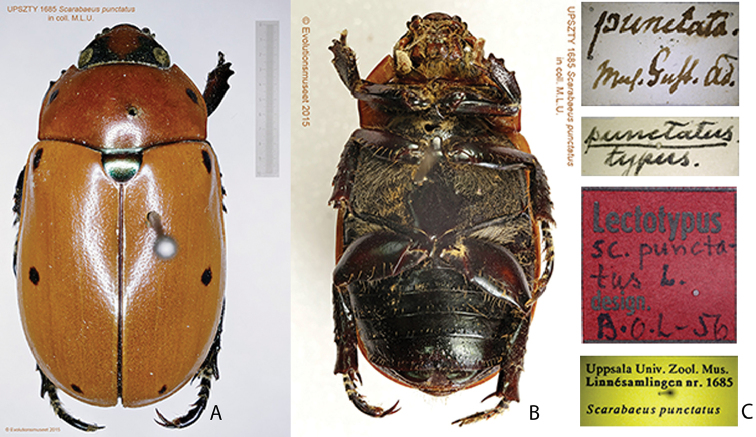
*Scarabaeus punctatus* Linnaeus (valid name *Pelidnota punctata* [Linnaeus]) female lectotype from UUZM. **A** Dorsal habitus **B** Ventral habitus **C** Specimen labels. Photographs courtesy of Dr. Hans Mejlon, Museum of Evolution, Uppsala University, Sweden.

The *Scarabaeus punctatus* Linnaeus, 1758 lectotype ♀ (Fig. 79) is deposited at UUZM labeled a) “punctatus. / typus.” (handwritten) b) “Lectotypus / Sc. puncta- / tus L. / design. / B.O.L-56” (red label, typeset and handwritten), c) “*Pelidnota* / *punctata* (Linnaeus) / Det: A.B.T. Smith 2015 ♀” (typeset). **Lectotype here designated**.

The *Melolontha lutea* Olivier, 1789 neotype ♂ is deposited at CMNC labeled a) “FLORIDA: Monroe Co. / Big Pine Key / Watsons Hammock / 6–30. VII.81 S. Peck / for. intercept-mal” (typeset), b) “Neotype 2009 / Melolontha / lutea Ol / Soula det.” (red label, typeset and handwritten), c) “Pelidnota / lutea (Ol.) / M Soula det 20” (handwritten and typeset), d) “*Pelidnota* / *punctata* (Linnaeus) / Det: A.B.T. Smith 2015 ♂”.

The *Pelidnota brevicollis* Casey, 1915 lectotype ♂ is deposited at USNM labeled a) “Jacksnvle / 8.02 Fla.” (typeset and handwritten), b) “♂” (typeset) c) “CASEY / bequest / 1925” (typeset), d) “TYPE USNM / 48537” (red label, typeset and handwritten), e) “brevicollis / Csy” (handwritten), f) “PELIDNOTA / BREVICOLLIS / CASEY, 1915 / LECTOTYPE / A.B.T. SMITH ♂” (red label, handwritten and typeset), g) “*Pelidnota* / *punctata* (Linnaeus) / Det: A.B.T. Smith 2015 ♂” (typeset). **Lectotype here designated**.

The *Pelidnota brevis* Casey, 1915 lectotype ♀ is deposited at USNM labeled a) “Brooklyn” (light blue label, handwritten), b) “CASEY / bequest / 1925” (typeset), c) “TYPE USNM / 48529” (red label, typeset and handwritten), d) “brevis / Csy.” (handwritten), e) “PELIDNOTA BREVIS / CASEY, 1915 / LECTOTYPE / A.B.T. SMITH ♀” (red label, handwritten and typeset), f) “*Pelidnota* / *punctata* (Linnaeus) / Det: A.B.T. Smith 2015 ♀” (typeset). **Lectotype here designated**.

The *Pelidnota debiliceps* Casey, 1915 lectotype ♂ is deposited at USNM labeled a) “Atlantic City, / N.J.” (handwritten), b) “CASEY / bequest / 1925” (typeset), c) “TYPE USNM / 48533” (red label, typeset and handwritten), d) “debiliceps / Csy.” (handwritten), e) “PELIDNOTA DEBILICEPS / CASEY, 1915 / LECTOTYPE / A.B.T. SMITH ♀” (red label, handwritten and typeset), f) “*Pelidnota* / *punctata* (Linnaeus) / Det: A.B.T. Smith 2015 ♀” (typeset). **Lectotype here designated**.

The *Pelidnota hudsonica* Casey, 1915 lectotype ♂ is deposited at USNM labeled a) “CASEY / bequest / 1925” (typeset), b) “hudsonica- 2 / PARATYPE USNM / 48536” (red label, typeset and handwritten), c) “PELIDNOTA HUDSONICA / CASEY, 1915 / LECTOTYPE / A.B.T. SMITH ♂” (red label, handwritten and typeset), d) “*Pelidnota* / *punctata* (Linnaeus) / Det: A.B.T. Smith 2015 ♂” (typeset). **Lectotype here designated**. One paralectotype ♀ is deposited at USNM labeled a) “Peekskill / NY” (typeset), b) “CASEY / bequest / 1925” (typeset), c) “TYPE USNM / 48536” (red label, handwritten and typeset), d) “hudsonica / Csy.” (handwritten), e) “PELIDNOTA HUDSONICA / CASEY, 1915 / PARALECTOTYPE / A.B.T. SMITH ♀” (yellow label, handwritten and typeset), f) “*Pelidnota* / *punctata* (Linnaeus) / Det: A.B.T. Smith 2015 ♀” (typeset). One paralectotype ♀ is deposited at USNM labeled a) (round white label), b) “CASEY / bequest / 1925” (typeset), c) “CASEY determ. / hudsonica-3” (typeset and handwritten), d) “PELIDNOTA HUDSONICA / CASEY, 1915 / PARALECTOTYPE / A.B.T. SMITH ♀” (yellow label, handwritten and typeset), e) “*Pelidnota* / *punctata* (Linnaeus) / Det: A.B.T. Smith 2015 ♀” (typeset).

The *Pelidnota oblonga* Casey, 1915 lectotype ♂ is deposited at USNM labeled a) “La.” (typeset), b) “CASEY / bequest / 1925” (typeset), c) “TYPE USNM / 48532” (red label, typeset and handwritten), d) “oblonga / Csy.” (handwritten), e) “PELIDNOTA / OBLONGA / CASEY, 1915 / LECTOTYPE / A.B.T. SMITH ♂” (red label, handwritten and typeset), f) “*Pelidnota* / *punctata* (Linnaeus) / Det: A.B.T. Smith 2015 ♂” (typeset). **Lectotype here designated**. One paralectotype ♀ is deposited at USNM labeled a) “La.” (typeset), b) “CASEY / bequest / 1925” (typeset), c) “CASEY determ. / ponderella 2” (typeset and handwritten), d) “Paratype of / oblonga” (red label, handwritten), e) “PELIDNOTA / OBLONGA / CASEY, 1915 / PARALECTOTYPE / A.B.T. SMITH ♀” (yellow label, handwritten and typeset), f) “*Pelidnota* / *punctata* (Linnaeus) / Det: A.B.T. Smith 2015 ♀” (typeset).

The *Pelidnota pallidipes* Casey, 1915 lectotype ♂ is deposited at USNM labeled a) “Newport News, Va / 6/25/89” (typeset and handwritten), b) “♂” (typeset), c) “CASEY / bequest / 1925” (typeset), d) “TYPE USNM / 48535” (red label, typeset and handwritten), e) “pallidipes / Csy.” (handwritten), f) “PELIDNOTA / PALLIDIPES / CASEY, 1915 / LECTOTYPE / A.B.T. SMITH ♂” (red label, handwritten and typeset), g) “*Pelidnota* / *punctata* (Linnaeus) / Det: A.B.T. Smith 2015 ♂” (typeset). **Lectotype here designated**. One paralectotype ♂ is deposited at USNM labeled a) “Southern Pines / VI-26 N.C 01 / A. H. Maneo” (typeset), b) “CASEY / bequest / 1925” (typeset), c) “CASEY determ. / pallidipes-9” (typeset and handwritten), d) “PELIDNOTA / PALLIDIPES / CASEY, 1915 / PARALECTOTYPE / A.B.T. SMITH ♂” (yellow label, handwritten and typeset), e) “*Pelidnota* / *punctata* (Linnaeus) / Det: A.B.T. Smith 2015 ♂” (typeset). One paralectotype ♂ is deposited at USNM labeled a) “Del” (handwritten), b) “♂” (typeset), c) “CASEY / bequest / 1925” (typeset), d) “CASEY determ. / pallidipes-2” (typeset and handwritten), e) “PELIDNOTA / PALLIDIPES / CASEY, 1915 / PARALECTOTYPE / A.B.T. SMITH ♂” (yellow label, handwritten and typeset), f) “*Pelidnota* / *punctata* (Linnaeus) / Det: A.B.T. Smith 2015 ♂” (typeset). One paralectotype ♂ is deposited at USNM labeled a) “Va” (typeset), b) “♂” (typeset), c) “CASEY / bequest / 1925” (typeset), d) “pallidipes. 3 / PARATYPE USNM / 48535” (red label, typeset and handwritten), e) “PELIDNOTA / PALLIDIPES / CASEY, 1915 / PARALECTOTYPE / A.B.T. SMITH ♂” (yellow label, handwritten and typeset), f) “*Pelidnota* / *punctata* (Linnaeus) / Det: A.B.T. Smith 2015 ♂” (typeset). One paralectotype ♀ is deposited at USNM labeled a) “Newport News, Va / 6/10/89” (typeset and handwritten), b) “♀” (typeset), c) “CASEY / bequest / 1925” (typeset), d) “pallidipes. 4 / PARATYPE USNM / 48535” (red label, typeset and handwritten), e) “PELIDNOTA / PALLIDIPES / CASEY, 1915 / PARALECTOTYPE / A.B.T. SMITH ♀” (yellow label, handwritten and typeset), f) “*Pelidnota* / *punctata* (Linnaeus) / Det: A.B.T. Smith 2015 ♀” (typeset). One paralectotype ♀ is deposited at USNM labeled a) “Miss” (typeset), b) “CASEY / bequest / 1925” (typeset), c) “pallidipes. 6 / PARATYPE USNM / 48535” (red label, typeset and handwritten), d) “PELIDNOTA / PALLIDIPES / CASEY, 1915 / PARALECTOTYPE / A.B.T. SMITH ♀” (yellow label, handwritten and typeset), e) “*Pelidnota* / *punctata* (Linnaeus) / Det: A.B.T. Smith 2015 ♀” (typeset). One paralectotype ♀ is deposited at USNM labeled a) “Miss” (typeset), b) “CASEY / bequest / 1925” (typeset), c) “pallidipes. 7 / PARATYPE USNM / 48535” (red label, typeset and handwritten), d) “PELIDNOTA / PALLIDIPES / CASEY, 1915 / PARALECTOTYPE / A.B.T. SMITH ♀” (yellow label, handwritten and typeset), e) “*Pelidnota* / *punctata* (Linnaeus) / Det: A.B.T. Smith 2015 ♀” (typeset). One paralectotype ♀ is deposited at USNM labeled a) “Miss” (typeset), b) “♀” (handwritten), c) “CASEY / bequest / 1925” (typeset), d) “pallidipes. 8 / PARATYPE USNM / 48535” (red label, typeset and handwritten), e) “PELIDNOTA / PALLIDIPES / CASEY, 1915 / PARALECTOTYPE / A.B.T. SMITH ♀” (yellow label, handwritten and typeset), f) “*Pelidnota* / *punctata* (Linnaeus) / Det: A.B.T. Smith 2015 ♀” (typeset). One paralectotype female at USNM labeled a) “Jacksnvle / Fla” (typeset), b) “♀” (handwritten), c) “CASEY / bequest / 1925” (typeset), d) “CASEY determ. / pallidipes-5” (typeset and handwritten), e) “PELIDNOTA / PALLIDIPES / CASEY, 1915 / PARALECTOTYPE / A.B.T. SMITH ♀” (yellow label, handwritten and typeset), f) “*Pelidnota* / *punctata* (Linnaeus) / Det: A.B.T. Smith 2015 ♀” (typeset).

The *Pelidnota ponderella* Casey, 1915 lectotype ♀ is deposited at USNM labeled a) “NE / U.S.” (handwritten), b) “CASEY / bequest / 1925” (typeset), c) “TYPE USNM / 48534” (red label, typeset and handwritten), d) “ponderella / Csy.” (handwritten), e) “PELIDNOTA / PONDERELLA / CASEY, 1915 / LECTOTYPE / A.B.T. SMITH ♀” (red label, handwritten and typeset), f) “*Pelidnota* / *punctata* (Linnaeus) / Det: A.B.T. Smith 2015 ♀” (typeset). **Lectotype here designated**.

The *Pelidnota strenua* Casey, 1915 lectotype ♀ is deposited at USNM labeled a) (pink disc with no text), b) “CASEY / bequest / 1925” (typeset), c) “TYPE USNM / 48528” (red label, typeset and handwritten), d) “strenua / Csy” (handwritten), e) “PELIDNOTA / STRENUA / CASEY, 1915 / LECTOTYPE / A.B.T. SMITH ♀” (red label, handwritten and typeset), f) “*Pelidnota* / *punctata* (Linnaeus) / Det: A.B.T. Smith 2015 ♀” (typeset). **Lectotype here designated**.

The *Pelidnota tarsalis* Casey, 1915 lectotype ♀ is deposited at USNM labeled a) “Peekskill / NY” (handwritten), b) “♀” (handwritten), c) “CASEY / bequest / 1925” (typeset), d) “TYPE USNM / 48539” (red label, typeset and handwritten), e) “tarsalis / Csy.” (handwritten), f) “PELIDNOTA / TARSALIS / CASEY, 1915 / LECTOTYPE / A.B.T. SMITH ♀” (red label, handwritten and typeset), g) “*Pelidnota* / *punctata* (Linnaeus) / Det: A.B.T. Smith 2015 ♀” (typeset). **Lectotype here designated**.

The *Pelidnota texensis* Casey, 1915 lectotype ♂ is deposited at USNM labeled a) “Horristo / Tex.” (handwritten), b) “CASEY / bequest / 1925” (typeset), c) “texensis- 3 / PARATYPE USNM / 48538” (red label, typeset and handwritten), d) “PELIDNOTA / TEXENSIS / CASEY, 1915 / LECTOTYPE / A.B.T. SMITH ♂” (red label, handwritten and typeset), e) “*Pelidnota* / *punctata* (Linnaeus) / Det: A.B.T. Smith 2015 ♂” (typeset). **Lectotype here designated**. One paralectotype ♂ is deposited at USNM labeled a) “Horristo / Tex.” (handwritten), b) “CASEY / bequest / 1925” (typeset), c) “texensis- 4 / PARATYPE USNM / 48538” (red label, handwritten and typeset), d) “PELIDNOTA / TEXENSIS / CASEY, 1915 / PARALECTOTYPE / A.B.T. SMITH ♂” (yellow label, handwritten and typeset), e) “*Pelidnota* / *punctata* (Linnaeus) / Det: A.B.T. Smith 2015 ♂” (typeset). One paralectotype ♀ is deposited at USNM labeled a) “Horristo / Tex.” (handwritten), b) “♀” (handwritten), c) “CASEY / bequest / 1925” (typeset), d) “TYPE USNM / 48538” (red label, handwritten and typeset), e) “texensis / Csy” (handwritten), f) “PELIDNOTA / TEXENSIS / CASEY, 1915 / PARALECTOTYPE / A.B.T. SMITH ♀” (yellow label, handwritten and typeset), g) “*Pelidnota* / *punctata* (Linnaeus) / Det: A.B.T. Smith 2015 ♀” (typeset). One paralectotype ♀ is deposited at USNM labeled a) “Horristo / Tex.” (handwritten), b) “CASEY / bequest / 1925” (typeset), c) “texensis- 2 / PARATYPE USNM / 48538” (red label, handwritten and typeset), d) “PELIDNOTA / TEXENSIS / CASEY, 1915 / PARALECTOTYPE / A.B.T. SMITH ♀” (yellow label, handwritten and typeset), e) “*Pelidnota* / *punctata* (Linnaeus) / Det: A.B.T. Smith 2015 ♀” (typeset).

The *Pelidnota genieri* Soula, 2009 holotype ♂ is deposited at CMNC labeled a) “Ottawa, ONT. / 5. VIII.1971 / J. E. H. Martin” (typeset), b) “C4269” (typeset), c) “CANADIAN / SCARAB / DATABASE / CSD014159” (typeset with two-dimensional barcode), d) “Holotype 2009 / Pelidnota / genieri S. / Soula” (red label, typeset and handwritten), e) “PELIDNOTA PUNCTATA (LINNAEUS) ♂ / Det:A.B.T.Smith 2015”. Allotype ♀ is deposited at CMNC labeled a) “Ottawa, ONT. / 5. VIII.1971 / J. E. H. Martin” (typeset), b) “C4280” (typeset), c) “Pelidnota / punctata / “(L.) / Det. J. McNamara 1974” (handwritten and typeset), d) “Allotype 2009 / Pelidnota / genieri S. / Soula” (red label, typeset and handwritten), e) “Canadian Museum of / Musée canadien de la / NATURE / CMNEN 00028000” (typeset with two-dimensional barcode), “PELIDNOTA PUNCTATA (LINNAEUS) ♀ / Det:A.B.T.Smith 2015”. 3 male paratypes, and 3 female paratypes at CMNC; 1 ♂ paratype labeled a) “Ottawa, ONT. / 5. VIII.1971 / J. E. H. Martin” (typeset), b) “C4302” (typeset), c) “CANADIAN / SCARAB / DATABASE / CSD014160” (typeset with two-dimensional barcode), d) “PELIDNOTA PUNCTATA (LINNAEUS) ♂ / Det:A.B.T.Smith 2015” e) “Paratype 2009 / Pelidnota / genieri S. / Soula” (red label, typeset and handwritten); 1 ♂ paratype labeled a) “ONT.: Ottawa / 14. VII.1988 / I. Dworakowska” (typeset), b) “CANADIAN / SCARAB / DATABASE / CSD014162” (typeset with two-dimensional barcode), c) “PELIDNOTA PUNCTATA (LINNAEUS) ♂ / Det:A.B.T.Smith 2015” d) “Paratype 2009 / Pelidnota / genieri S. / Soula” (red label, typeset and handwritten); 1 ♂ paratype labeled a) “OTTAWA, ONT. / VI. 1980 / H. & A. Howden / reared from elm / stump, 2 years as larva” (typeset and handwritten), b) “CANADIAN / SCARAB / DATABASE / CSD014163” (typeset with two-dimensional barcode), c) “PELIDNOTA PUNCTATA (LINNAEUS) ♂ / Det:A.B.T.Smith 2015” d) “Paratype 2009 / Pelidnota / genieri S. / Soula” (red label, typeset and handwritten); 1 ♀ paratype labeled a) “Ottawa, ONT. / 5. VIII.1971 / J. E. H. Martin” (typeset), b) “C4277” (typeset), c) “CANADIAN / SCARAB / DATABASE / CSD014165” (typeset with two-dimensional barcode), d) “PELIDNOTA PUNCTATA (LINNAEUS) ♀ / Det:A.B.T.Smith 2015” e) “Paratype 2008 / Pelidnota / genieri S. / Soula” (red label, typeset and handwritten); 1 ♀ paratype labeled a) “CANADA: Ont. / Ottawa / 16. VIII.1993 / H. & A. Howden” (typeset and handwritten), b) “CANADIAN / SCARAB / DATABASE / CSD014166” (typeset with two-dimensional barcode), c) “PELIDNOTA PUNCTATA (LINNAEUS) ♀ / Det:A.B.T.Smith 2015” e) “Paratype 2008 / Pelidnota / genieri S. / Soula” (red label, typeset and handwritten); 1 ♀ paratype labeled a) “Ottawa , Ont. / 14. VII 72 / H. F. HOWDEN” (handwritten), b) “CANADIAN / SCARAB / DATABASE / CSD014167” (typeset with two-dimensional barcode), c) “PELIDNOTA PUNCTATA (LINNAEUS) ♀ / Det:A.B.T.Smith 2015” e) “Paratype 2009 / Pelidnota / genieri S. / Soula” (red label, typeset and handwritten). The following specimens are deposited at CCECL. 2 ♂ paratypes, 3 ♀ paratypes: “OTTAWA, ONT. VII.5.76 M. SANBORNE//[matrix barcode] CANADIAN SCARAB DATABASE CSD014161//*PELIDNOTA PUNCTATA* (LINNAEUS) ♂ det. A.B.T.Smith 2015//Paratype 2009 *Pelidnota genieri* S. Soula” (47030610); “ONT. RUSSEL CO. Cumberland Village VII.31.72 L. Ling//[matrix barcode] CANADIAN SCARAB DATABASE CSD014164//PELIDNOTA PUNCTATA (LINNAEUS) ♂ det. A.B.T.Smith 2015//Paratype 2009 *Pelidnota genieri* S. Soula” (47030611); “CANADA: Ont. Ottawa 10. VIII.1992 N. House//[matrix barcode] CANADIAN SCARAB DATABASE CSD014168//*PELIDNOTA PUNCTATA* (LINNAEUS) ♀ det. A.B.T.Smith 2015//Paratype 2009 *Pelidnota genieri* S. Soula” (47030612); “Constance Bay Carieton Co. ONT VII.18.77//[matrix barcode] CANADIAN SCARAB DATABASE CSD014169//*PELIDNOTA PUNCTATA* (LINNAEUS) ♀ det. A.B.T.Smith 2015//Paratype 2009 *Pelidnota genieri* S. Soula” (47030613); “Ottawa, Ont. 12. VII.1977 A.T. Howden//Paratype 2009 *Pelidnota genieri* S. Soula//*Pelidnota francoisgenieri* Moore + Jameson 2013 det. MR Moore ‘15” (47030614). Genitalia card-mounted underneath one male paratype (47030614). Box 4618674 SOULA.

### Pelidnota punctulata

Taxon classificationAnimaliaColeopteraScarabaeidae

H. W. Bates, 1888

Pelidnota punctulata H. W. Bates, 1888: 276 [original combination].
Pelidnota (Pelidnotidia) punctulata H. W. Bates [new subgeneric combination by Casey 1915: 80].
Pelidnota (Pelidnota) punctulata H. W. Bates [new subgeneric combination by Ohaus 1918: 24].
Pelidnota punctulata H. W. Bates [removal of subgeneric classification by Soula 2009: 78–80].

#### Distribution.

BELIZE: Toledo (H. W. Bates 1888, Blackwelder 1944, Hardy 1975, Maes 1987, Soula 2009). COLOMBIA: Chocó (Hardy 1975, Morón 1979, Maes 1987, Neita-Moreno 2011). COSTA RICA: Cartago, Guanacaste (Hardy 1975, 1985, Maes 1987, Solís and Morón 1994). ECUADOR (Hardy 1975, Morón 1979, Maes 1987). EL SALVADOR: La Libertad, San Salvador (Hardy 1975). GUATEMALA: Escuintla, Petén, San Marcos, Suchitepéquez (H. W. Bates 1888, Ohaus 1918, 1934b, Blackwelder 1944, Machatschke 1972, Hardy 1975, Morón 1979, Maes 1987). HONDURAS: Cortés (Casey 1915, Ohaus 1918, 1934b, Machatschke 1972). NICARAGUA: Chontales, León, Managua (H. W. Bates 1888, Ohaus 1918, 1934b, Blackwelder 1944, Machatschke 1972, Hardy 1975, Maes 1987). MEXICO: Campeche, Chiapas, Oaxaca, Puebla, Quintana Roo, Tabasco, Veracruz, Yucatán (H. W. Bates 1888, Ohaus 1934b, Blackwelder 1944, Carrillo et al. 1966, Machatschke 1972, Hardy 1975, Morón 1979, Maes 1987, Palacios-Rios et al. 1990, Thomas 1993, Lobo and Morón 1993, Morón et al. 1997, Sánchez-Soto 1997, Reyes Novelo and Morón 2005, Pacheco Flores et al. 2008, Krajcik 2008, Soula 2009, Morón-Ríos and Morón 2016). PANAMA: Chiriquí (Hardy 1975, Maes 1987, Ratcliffe 2002). VENEZUELA (Maes 1987).

#### Types.

1 ♀ lectotype of *Pelidnota punctulata* at BMNH (Hardy 1975, Soula 2009); 17 type specimens at BMNH (Soula 2009); 5 type specimens at MNHN (Soula 2009); 1 type specimen at IRSNB (Soula 2009).

### Pelidnota purpurea esperitosantensis

Taxon classificationAnimaliaColeopteraScarabaeidae

(Soula, 2006)

Strigidia purpurea esperitosantensis Soula, 2006: 34-35 [original combination].
Pelidnota (Strigidia) purpurea esperitosantensis (Soula) [new combination and new subgeneric classification by Özdikmen 2009: 145].
Pelidnota purpurea esperitosantensis (Soula) [removal of subgeneric classification by Soula 2009:115].

#### Distribution.

BRAZIL: Espírito Santo (Soula 2006).

#### Types.

The following specimens are deposited at CMNC. 1 ♂ holotype, 1 ♀ allotype: “BRASIL E. SANTO. Linhares Sooretama NOV.62 A. Martínez//H. & A. HOWDEN COLLECTION *ex.* A. Martinez coll.//*Pelidnota purpurea* ♂ Burm. A. MARTINEZ-DET.1965//Holotype Pelid? *Strigidia purpurea esperitosanten-sis* Soula Soula”. Allotype with same labels except “♀” on determination label.

#### Remarks.

Soula (2006) named this subspecies for the Brazilian state of Espírito Santo (although the spelling of the subspecific epithet is incorrectly formed). Soula (2006) illustrated the form of the male parameres which, in all respects, are of the nominotypical form. The subspecies was based on the following characters: punctation finer, overall coloration, pronotum less convex, elytra slightly longer.

### Pelidnota purpurea purpurea

Taxon classificationAnimaliaColeopteraScarabaeidae

Burmeister, 1844

Pelidnota purpurea Burmeister, 1844: 394 [original combination].
Pelidnota (Ganonota) purpurea Burmeister [new subgeneric combination by Ohaus 1918: 26].
Pelidnota (Strigidia) purpurea Burmeister [new subgeneric combination by Machatschke 1970: 157].
Pelidnota (Odontognathus) purpurea Burmeister [new subgeneric combination by Hardy 1975: 4].
Strigidia purpurea (Burmeister) [new combination by Soula 2006: 33–34].
Pelidnota (Strigidia) purpurea Burmeister [revised combination and revised subgeneric combination by Özdikmen 2009: 145].
Pelidnota purpurea purpurea Burmeister [removal of subgeneric classification by Soula 2009: 115].

#### Distribution.

BRAZIL: Rio de Janeiro (Burmeister 1844, Blanchard 1851, Ohaus 1918, 1934b, Blackwelder 1944, Machatschke 1972, Soula 2006, Krajcik 2008).

#### Types.

1 ♀ lectotype and 1 paralectotype of *Pelidnota purpurea* at MLUH (Soula 2006).

#### Remarks.

CCECL contains a *P. purpurea purpurea* specimen labeled as a male alloréférent with the following data: 1 ♂ alloréférent: “MUSÉUM PARIS Rio de Castelnau 1844//Alloréférent de *Strigidia purpurea* Burm. M. SOULA det 19” (47030312). Genitalia card-mounted underneath the male alloréférent. Box 4618659 SOULA.

### Pelidnota quadripunctata

Taxon classificationAnimaliaColeopteraScarabaeidae

F. Bates, 1904

Pelidnota quadripunctata F. Bates, 1904: 253, 260 [original combination].
Pelidnota (Ganonota) quadripunctata F. Bates [new subgeneric combination by Ohaus 1918: 27].
Pelidnota (Strigidia) quadripunctata F. Bates [new subgeneric combination by Machatschke 1970: 157].
Pelidnota (Odontognathus) quadripunctata F. Bates [new subgeneric combination by Hardy 1975: 4].
Strigidia quadripunctata (F. Bates) [new combination by Soula 2006: 84–85].
Pelidnota (Strigidia) quadripunctata F. Bates [revised combination and revised subgeneric combination by Özdikmen 2009: 145].
Pelidnota quadripunctata F. Bates [removal of subgeneric classification by Soula 2009: 115].

#### Distribution.

FRENCH GUIANA: Cayenne (F. Bates 1904, Ohaus 1913, 1918, 1934b, Blackwelder 1944, Machatschke 1972, Krajcik 2008, Soula 2006, 2010c).

#### Types.

1 ♀ holotype of *Pelidnota quadripunctata* at IRSNB (Soula 2010a).

### Pelidnota recondita

Taxon classificationAnimaliaColeopteraScarabaeidae

Delgado-Castillo, Deloya, & Morón, 1988

Pelidnota (Pelidnota) recondita Delgado-Castillo, Deloya, & Morón, 1988: 132, 133–139 [original combination].
Pelidnota (Pelidnota) jalapensis H. W. Bates [syn. by Morón et al. 1997: 27].
Pelidnota recondita Delgado-Castillo, Deloya, & Morón, 1988 [removal of subgeneric classification and revised species status by Soula 2009: 63–64].

#### Distribution.

MEXICO: Guerrero (Delgado-Castillo et al. 1988, Krajcik 2008, Soula 2009).

#### Types.

1 ♂ holotype, 1 ♀ allotype of Pelidnota (Pelidnota) recondita in MXAL; additional paratypes at ZMHB and IEXA (Delgado-Castillo et al. 1988).

#### Remarks.

Krajcik (2012, 2013) omitted this name from his catalogs.

### Pelidnota rioensis

Taxon classificationAnimaliaColeopteraScarabaeidae

Soula, 2009

Pelidnota arnaudi rioensis Soula, 2009: 73–74 [original combination].
Pelidnota rioensis Soula [**new status**].

#### Distribution.

BRAZIL: Rio de Janeiro (Soula 2009).

#### Types.

Male holotype, female allotype, and three paratypes at MNHN (Soula 2009). The following specimens are deposited at CCECL. 4 ♂ paratypes, 1 ♀ paratype: “Etat de Rio Brazil. M. SOULA det 19//Paratype 2008 *Pelidnota arnaudi rioensis* S. Soula” (47030583); four paratypes with identical label data: “Nova Friborgo - R. J. XII/92 - BRESIL//Paratype 2008 *Pelidnota arnaudi rioensis* S. Soula” (47030584 to 47030587). Genitalia card-mounted underneath three male paratypes. Box 4618670 SOULA.

#### Remarks.

Because *Pelidnota arnaudi arnaudi* Soula is an unavailable name and *P. arnaudi rioensis* is available, it is given herein **new status** as *P. rioensis*.

### Pelidnota rivascanteroi

Taxon classificationAnimaliaColeopteraScarabaeidae

(Soula, 2006)

Strigidia rivascanteroi Soula, 2006: 12, 54–55 [original combination].
Pelidnota rivascanteroi (Soula) [new combination by Soula 2009: 115].

#### Distribution.

BRAZIL: Ceará (Soula 2006).

#### Types.

The following specimens are deposited at CCECL. 1 ♀ holotype, 2 ♀ paratypes: “UBAJARA mt. 800 CEARÁ-BRASILE, GEN. 95 MIGLIOLI//Holotype 2006 *Strigidia rivascanteroi* Sou Soula” (47030424); “UBAJARA mt. 800, CEARÁ-BRASILE, GEN. 95 MIGLIOLI//Paratype 2006 *Strigidia rivascanteroi* Sou. Soula” (47030425); “Cametá//Paratype 2006 *Strigidia rivascanteroi* S. Soula” (47030426). Genitalia are card-mounted underneath the female holotype and a female paratype. Box 4618663 SOULA.

#### Remarks.

Soula (2006) compared this species with *P. discicollis* and the image that accompanies the description looks remarkably similar to other specimens of *P. discicollis*. Future research should examine the validity of this species.

### Pelidnota rostrata

Taxon classificationAnimaliaColeopteraScarabaeidae

Burmeister, 1844

Pelidnota rostrata Burmeister, 1844: 406 [original combination].
Heteropelidnota rostrata (Burmeister) [new combination by Ohaus 1918: 30].
Pelidnota rostrata Burmeister [revised combination by Soula 2008: 14–15].
Pelidnota viridana Blanchard, 1851 **synonym.**
Pelidnota viridana Blanchard, 1851: 213 [original combination].
Heteropelidnota rostrata (Burmeister, 1844) [syn. by Ohaus 1918: 30].

#### Distribution.

BRAZIL: Minas Gerais, Rio de Janeiro, Santa Catarina, São Paulo (Burmeister 1844, 1855, Blanchard 1851, Ohaus 1908a, 1918, 1934b, Martinez 1967, Machatschke 1972, Krajcik 2008, Soula 2008).

#### Types.

**Figure 80.**
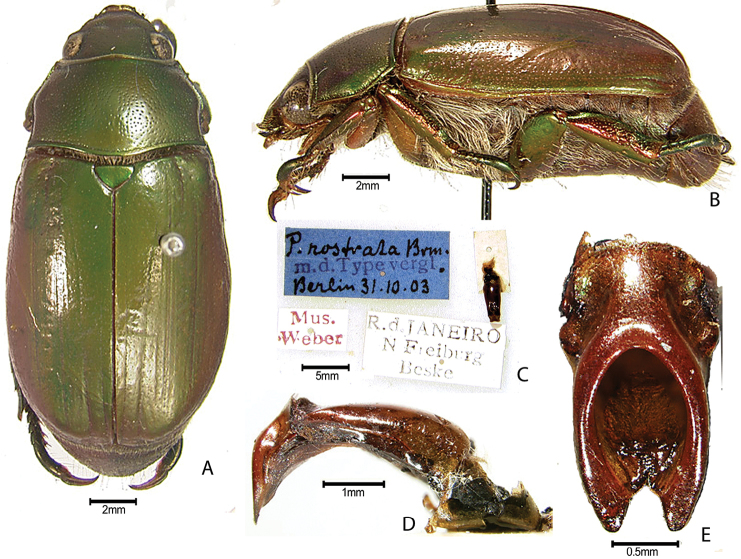
*Pelidnota rostrata* Burmeister (male specimen compared with Burmeister’s syntype by Ohaus from the Weber Collection). **A** Dorsal habitus **B** Lateral habitus **C** Specimen labels and male genitalia **D** Male genitalia, lateral view **E** Male parameres, dorsal view.

**Figure 81.**
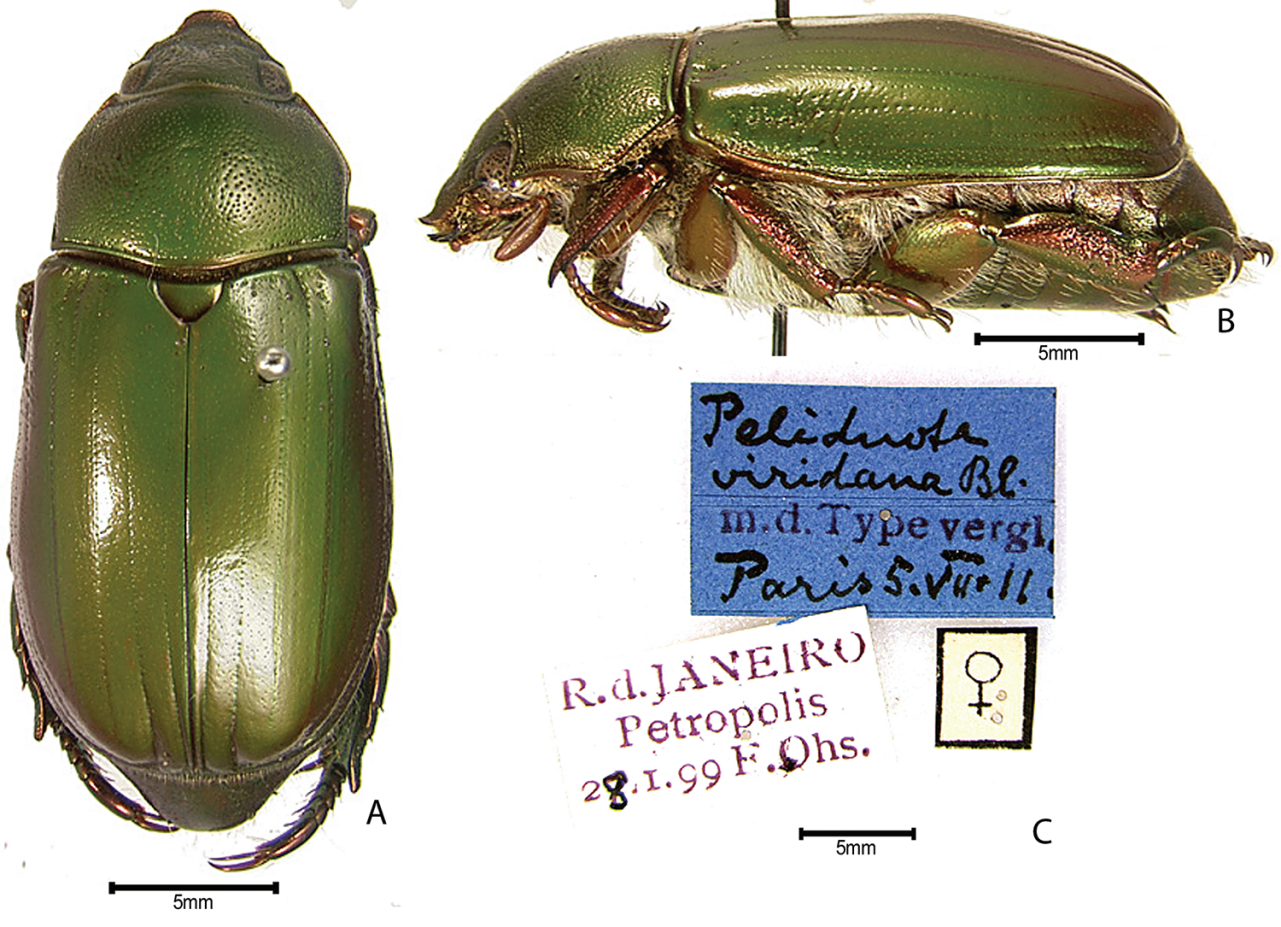
*Pelidnota viridana* Blanchard (female specimen compared with Blanchard’s syntype by Ohaus which is deposited at MNHN) (valid name *Pelidnota rostrata* Burmeister). **A** Dorsal habitus **B** Lateral habitus **C** Specimen labels.

1 ♀ lectotype and 1 paralectotype of *Pelidnota rostrata* at MLUH (Soula 2008). An exemplar of *P. rostrata* identified by Ohaus and compared with Burmeister’s type specimen is figured (Fig. 80). A female exemplar of *P. viridana* identified by Ohaus and compared with Blanchard’s syntype specimen is figured (Fig. 81)

#### Remarks.

Females of *P. rostrata* possess a longitudinal carina at the apex of the pygidium. Soula provided an image of the male parameres of *P. rostrata* (Soula 2008: 15). This image appears to be directly from Martínez’s discussion of *P. rostrata* (Martínez 1967).

### Pelidnota rouchei

Taxon classificationAnimaliaColeopteraScarabaeidae

(Soula, 2006)

Strigidia rouchei Soula, 2006: 11, 76–77 [original combination].
Pelidnota rouchei (Soula) [new combination by Soula 2009: 115].

#### Distribution.

VENEZUELA: Merida (Soula 2006).

#### Types.

The following specimens are deposited at CCECL. 1 ♂ holotype, 1 ♀ allotype, 1 ♀ paratype: “VENEZUELA Edo. Merida NP Sierra Nevada, La Mucuy 2400m 13. IV.1995 leg. Hornburg, Krause//Holotype 2006 *Strigidia rouchei* Sou. Soula” (47030135); “Merida; 1850 m V/1999; Venez.//Allotype 2006 *Strigidia rouchei* Sou. Soula” (47030136); “Merida; 1850 m V/1999; Venez.//Paratype 2006 *Strigidia rouchei* Sou. Soula” (47030137). Genitalia card-mounted underneath the holotype. Box 4618654 SOULA.

### Pelidnota rubripennis riedeli

Taxon classificationAnimaliaColeopteraScarabaeidae

(Ohaus, 1905)

Odontognathus riedeli Ohaus, 1905: 312–313 [original combination].
Pelidnota (Ganonota) riedeli (Ohaus) [new combination and new subgeneric combination by Ohaus 1918: 27].
Pelidnota (Strigidia) riedeli (Ohaus) [new subgeneric combination by Machatschke 1970: 157].
Pelidnota (Odontognathus) riedeli (Ohaus) [new subgeneric combination by Hardy 1975: 4].
Strigidia riedeli (Ohaus) [new combination by Soula 2006: 31].
Strigidia rubripennis riedeli (Ohaus) [new subspecific status by Soula 2006: 33].
Pelidnota (Strigidia) riedeli (Ohaus) [revised combination and revised species status by Özdikmen 2009: 145].
Pelidnota rubripennis riedeli (Ohaus) [**revised status and revised combination**].

#### Distribution.

BRAZIL: São Paulo (Ohaus 1905, 1918, 1934b, Blackwelder 1944, Machatschke 1972, Soula 2006, Krajcik 2008).

#### Types.

**Figure 82.**
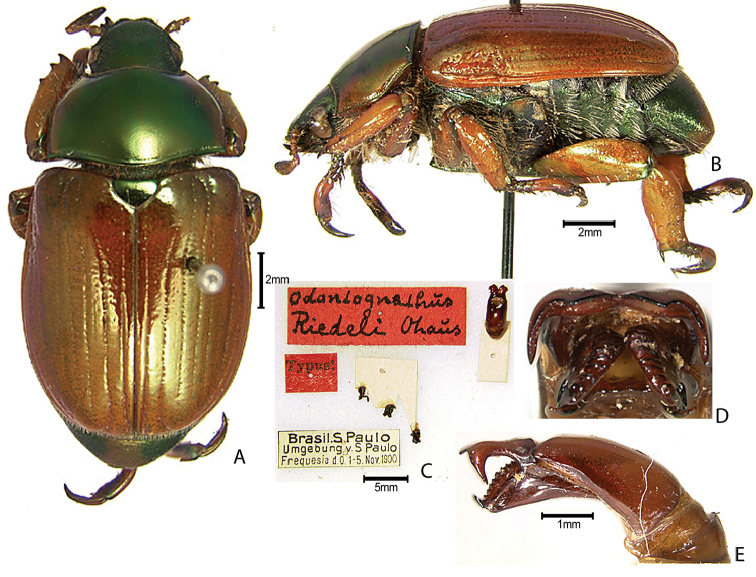
*Odontognathus riedeli* Ohaus (valid name *Pelidnota rubripennis riedeli* [Ohaus]) syntype male from ZMHB. **A** Dorsal habitus **B** Lateral habitus **C** Specimen labels, mouthparts, and male genitalia **D** Male parameres, caudal view **E** Male genitalia, lateral view.

1 ♂ syntype of *Odontognathus riedeli* was recorded by Soula (2006), this may be the same specimen from ZMHB (Fig. 82).

#### Remarks.

Özdikmen (2009) did not acknowledge Soula (2006) and listed P. (Strigidia) riedeli (Ohaus) as a valid species name. For taxonomic stability, we follow Soula (2006) and consider *Pelidnota rubripennis riedeli* (Ohaus) as a subspecies, **revised status**, in the genus *Pelidnota*, **revised combination**, until the validity of this taxon can be further evaluated.

### Pelidnota rubripennis rubripennis

Taxon classificationAnimaliaColeopteraScarabaeidae

(Burmeister, 1844)

Strigidia rubripennis Burmeister, 1844: 390 [original combination].
Odontognathus rubripennis (Burmeister) [new combination by Harold 1869b: 1221].
Pelidnota (Ganonota) rubripennis (Burmeister) [new combination and new subgeneric combination by Ohaus 1918: 27].
Pelidnota (Strigidia) rubripennis (Burmeister) [new subgeneric combination by Machatschke 1970: 157].
Pelidnota (Odontognathus) rubripennis (Burmeister) [new subgeneric combination by Hardy 1975: 4].
Strigidia rubripennis Burmeister [revised combination by Soula 2006: 32-33].
Pelidnota (Strigidia) rubripennis (Burmeister) [revised combination and revised subgeneric combination by Özdikmen 2009: 145].
Pelidnota rubripennis rubripennis (Burmeister) [removal of subgeneric classification by Soula 2009: 115].
Pelidnota rufipennis Waterhouse, 1876 **synonym.**
Pelidnota rufipennis Waterhouse, 1876: 23 [original combination].
Pelidnota (Ganonota) rubripennis (Burmeister) [syn. by Ohaus 1918: 27].
Pelidnota (Strigidia) rubripennis forma rufipennis (Waterhouse) [new infrasubspecific status and new subgeneric combination by Machatschke 1972: 29].
Pelidnota (Odontognathus) rubripennis forma rufipennis (Waterhouse) [new subgeneric combination by Hardy 1975: 4].
Strigidia rubripennis Burmeister [syn. by Soula 2006: 33].

#### Distribution.

BRAZIL: Pernambuco, Rio de Janeiro (Burmeister 1844, Ohaus 1918, 1934b, Blackwelder 1944, Machatschke 1972, Soula 2006, Krajcik 2008).

#### Types.

**Figure 83.**
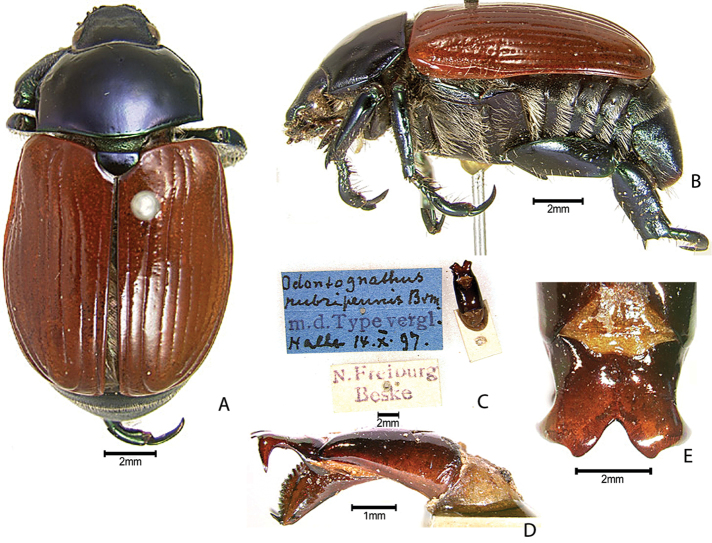
*Pelidnota rubripennis rubripennis* (Burmeister) (male specimen compared with Burmeister’s syntype by Ohaus from MLUH). **A** Dorsal habitus **B** Lateral habitus **C** Specimen labels and male genitalia **D** Male genitalia, lateral view **E** Male parameres, dorsal view.

**Figure 84.**
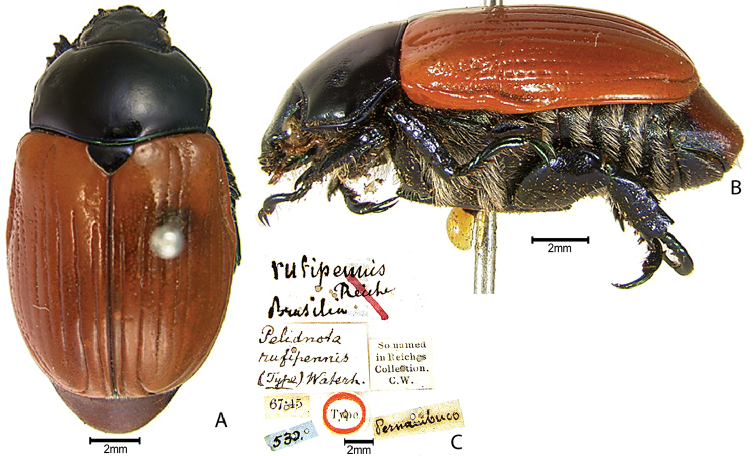
*Pelidnota rufipennis* Waterhouse (valid name *Pelidnota rubripennis* [Burmeister]) type female (see “*Type specimens and lectotype designation*” in Methods) from BMNH. **A** Dorsal habitus **B** Lateral habitus **C** Specimen labels.

1 ♂ syntype specimen of *Strigidia rubripennis* Burmeister is deposited at MLUH; 1 ♀ type specimen of *Strigidia rufipennis* (Waterhouse) is deposited at BMNH (Fig. 84); Soula (2006) recorded 1 ♂ lectotype and paralectotypes (institution not provided). An exemplar specimen identified by Ohaus and compared with the type specimen of *S. rubripennis* is figured (Fig. 83).

### Pelidnota rubriventris

Taxon classificationAnimaliaColeopteraScarabaeidae

Blanchard, 1851

Pelidnota rubriventris Blanchard, 1851: 213 [original combination].
Pelidnota (Chalcoplethis) rubriventris Blanchard [new subgeneric combination by Ohaus 1918: 28].
Strigidia rubriventris (Blanchard) [new combination by Soula 2006: 75–76].
Pelidnota rubriventris Blanchard [revised combination by Soula 2009: 115].

#### Distribution.

COLOMBIA: Antioquia, Boyacá, Valle del Cauca (Ohaus 1918, 1934b, Machatschke 1972, Restrepo et al. 2003, Soula 2006, López-García et al. 2015). PANAMA (Ratcliffe 2002).

#### Types.

Soula (2006) recorded 1 ♀ syntype of *Pelidnota rubriventris* but he did not provide the institution.

### Pelidnota rugulosa rugulosa

Taxon classificationAnimaliaColeopteraScarabaeidae

Burmeister, 1844

Pelidnota rugulosa Burmeister, 1844: 398–399 [original combination].
Pelidnota (Chalcoplethis) rugulosa Burmeister [new subgeneric combination by Ohaus 1918: 28].
Strigidia rugulosa (Burmeister) [new combination by Soula 2006: 65–66].
Pelidnota rugulosa rugulosa Burmeister [revised combination by Soula 2009: 115].

#### Distribution.

BRAZIL: Rio de Janeiro, Sáo Paulo (Burmeister 1844, 1855, Blanchard 1851, Ohaus 1918, 1934b, Blackwelder 1944, Machatschke 1972, Soula 2006, Krajcik 2008).

### Pelidnota rugulosa santacatarinensis

Taxon classificationAnimaliaColeopteraScarabaeidae

(Soula, 2006)

Strigidia rugulosa santacatarinensis Soula, 2006: 66 [original combination].
Pelidnota rugulosa santacatarinensis (Soula) [new combination by Soula 2009: 116].

#### Distribution.

BRAZIL: Santa Catarina (Soula 2006).

#### Types.

The following specimens are deposited at CCECL. 1 ♂ holotype, 1 ♀ allotype, 13 ♂ paratypes, 4 ♀ paratypes: “Sao Bento do Sul. 11/94 S.C. coll. – SOULA//Holotype Sou. *Chalcoplethis rugulosa santacatarinensis* Soula det. [obverse] 2005” (47030138); “Sao Bento do Sul (S. C.) 11/94 coll. – SOULA//Allotype Sou. 2005 *Chalcoplethis rugulosa santacatarinensis* Sou. Soula det.” (47030139); “Sao Bento do Sul S. C. 11/94 coll. – SOULA//Paratype 2005 *Chalcoplethis rugulosa santacatarinensis* S. Soula det.” (47030140); eight paratypes with identical label data “São Bento do Sul Santa Catarina Brésil//Paratype 2005 *Chalcoplethis rugulosa santacatarinensis* S. Soula det.” (47030141 to 47030146, exch06 and exch07); “Sao Bento S. C. coll. – SOULA//Paratype 2005 *Chalcoplethis rugulosa santacatarinensis* S. Soula det.” (47030147); “São Bento do Sul S. C. M. SOULA det 19//Nevinson Coll. 1918-14.//Paratype 2005 *Chalcoplethis rugulosa santacatarinensis* S. Soula det.” (47030148); two paratypes with identical label data “Rio Vermelho Santa Catarina Brésil M. SOULA det 19 [obverse] 12/89//Paratype 2005 *Chalcoplethis rugulosa santacatarinensis* S. Soula det.” (47030149 and 47030150); “Rio Vermelho Santa Catarina 12/89 Brasil M. SOULA det 19//Paratype 2005 *Chalcoplethis rugulosa santacatarinensis* S. Soula det.” (47030151); “Rio Vermelho Santa Catarina 12/89 M. SOULA det 19//Paratype *Chalcoplethis rugulosa santacatarinensis* S. Soula det.” (47030152); “Caripo alegre Santa Catarina BRESIL Fev. 1991 Coll. Th. Porion//Paratype 2005 *Chalcoplethis rugulosa santacatarinensis* S. Soula det.” (47030153). “JOINVILLE SANTA CATARINA DECEMBRE 1986 BRESIL T. PORION LEG.//*Chalcoplethis rugulosa rugulosa*//Paratype 2005 *Chalcoplethis rugulosa santacatarinensis* S. Soula det.” (47030154). Genitalia card-mounted underneath the holotype male, allotype female, thirteen male paratypes, and two female paratypes. Box 4618655 SOULA. The following specimen is deposited at CMNC. 1 ♀ paratypes: “BRASIL Sta. Catarina Sao Bento A. Maller-leg. Coll. Martínez Oct.-967//H. & A. HOWDEN COLLECTION *ex.* A. Martinez coll.//*Pelidnota rugulosa* ♂ Burm. A. MARTINEZ-DET.1969//Paratype 2006 *Strigidia rugulosa santacatarinen-sis* Soula Soula”.

### Pelidnota sanctidomini caliensis

Taxon classificationAnimaliaColeopteraScarabaeidae

(Soula, 2006)

Strigidia santidomini (*sic*) *caliensis* Soula, 2006: 79 [original combination].
Pelidnota (Strigidia) sanctidomini caliensis (Soula) [new combination and new subgeneric combination by Özdikmen 2009: 145].
Pelidnota sanctidomini caliensis (Soula) [removal of subgeneric classification by Soula 2009: 115].

#### Distribution.

COLOMBIA: Valle del Cauca (Soula 2006).

#### Types.

The following specimen is deposited at CCECL. 1 ♂ holotype: “(Colombie) Cali 06/92//Holotype 2006 *Strigidia santidomini caliensis* Soula Soula” (47030126). Genitalia card-mounted underneath the holotype. Box 4618654 SOULA.

#### Remarks.

Soula (2006) described the new subspecies based on its slightly longer body, slightly longer clypeus that is more emarginate, punctures that are larger and deeper, and more robust tarsomeres. He stated that the male parameres are very near the nominotypical species. The subspecies is, evidently, described based on a single male specimen (the holotype) from Cali, Colombia (label date “06/92”).

### Pelidnota sanctidomini sanctidomini

Taxon classificationAnimaliaColeopteraScarabaeidae

Ohaus, 1905

Pelidnota sanctidomini Ohaus, 1905: 317 [original combination].
Pelidnota (Ganonota) sanctidomini Ohaus [new subgeneric combination by Ohaus 1918: 26].
Pelidnota (Strigidia) sanctidomini Ohaus [new subgeneric combination by Machatschke 1970: 157].
Pelidnota (Odontognathus) sanctidomini Ohaus [new subgeneric combination by Hardy 1975: 4].
Pelidnota (Strigidia) sanctidomini Ohaus [revised subgeneric combination by Chalumeau 1985: 257].
Strigidia sanctidomini (Ohaus) [new combination by Soula 2006: 77–78].
Pelidnota (Strigidia) santidomini (*sic*) Ohaus [revised combination and revised subgeneric combination by Özdikmen 2009: 145].
Pelidnota santidomini santidomini (*sic*) Ohaus [removal of subgeneric classification by Soula 2009: 116].
Pelidnota sanctidomini sanctidomini Ohaus [valid name].
Pelidnota pubes Ohaus, 1913: 502–503 [original combination]. **synonym.**
Pelidnota (Ganonota) pubes Ohaus [new subgeneric combination by Ohaus 1918: 26].
Pelidnota (Strigidia) pubes Ohaus [new subgeneric combination by Machatschke 1970: 157].
Pelidnota (Odontognathus) pubes Ohaus [new subgeneric combination by Hardy 1975: 4].
Strigidia santidomini (*sic*) (Ohaus) [syn. by Soula 2006: 78].

#### Distribution.

CENTRAL AMERICA (Chalumeau 1985). COLOMBIA: Chocó (Neita-Moreno 2011). ECUADOR: Imbabura, Pichincha (Ohaus 1913, 1918, 1934b, Machatschke 1972, Paucar-Cabrera 2005, Krajcik 2008). NICARAGUA: Atlántico Norte (MLJC). PANAMA: Colón (Ratcliffe 2002).

#### Types.

**Figure 85.**
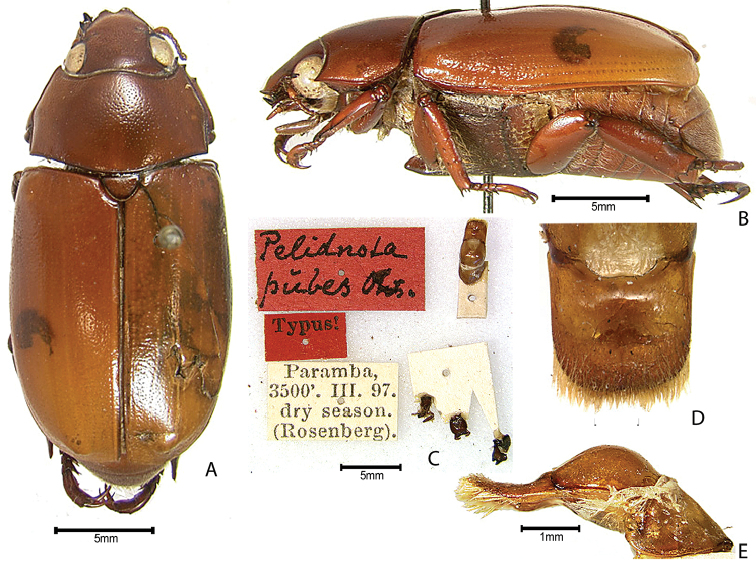
*Pelidnota pubes* Ohaus syntype male from ZMHB (valid name *Pelidnota sanctidomini sanctidomini* Ohaus). **A** Dorsal habitus **B** Lateral habitus **C** Specimen labels, mouthparts, and male genitalia **D** Male parameres, dorsal view **E** Male genitalia, lateral view.

**Figure 86.**
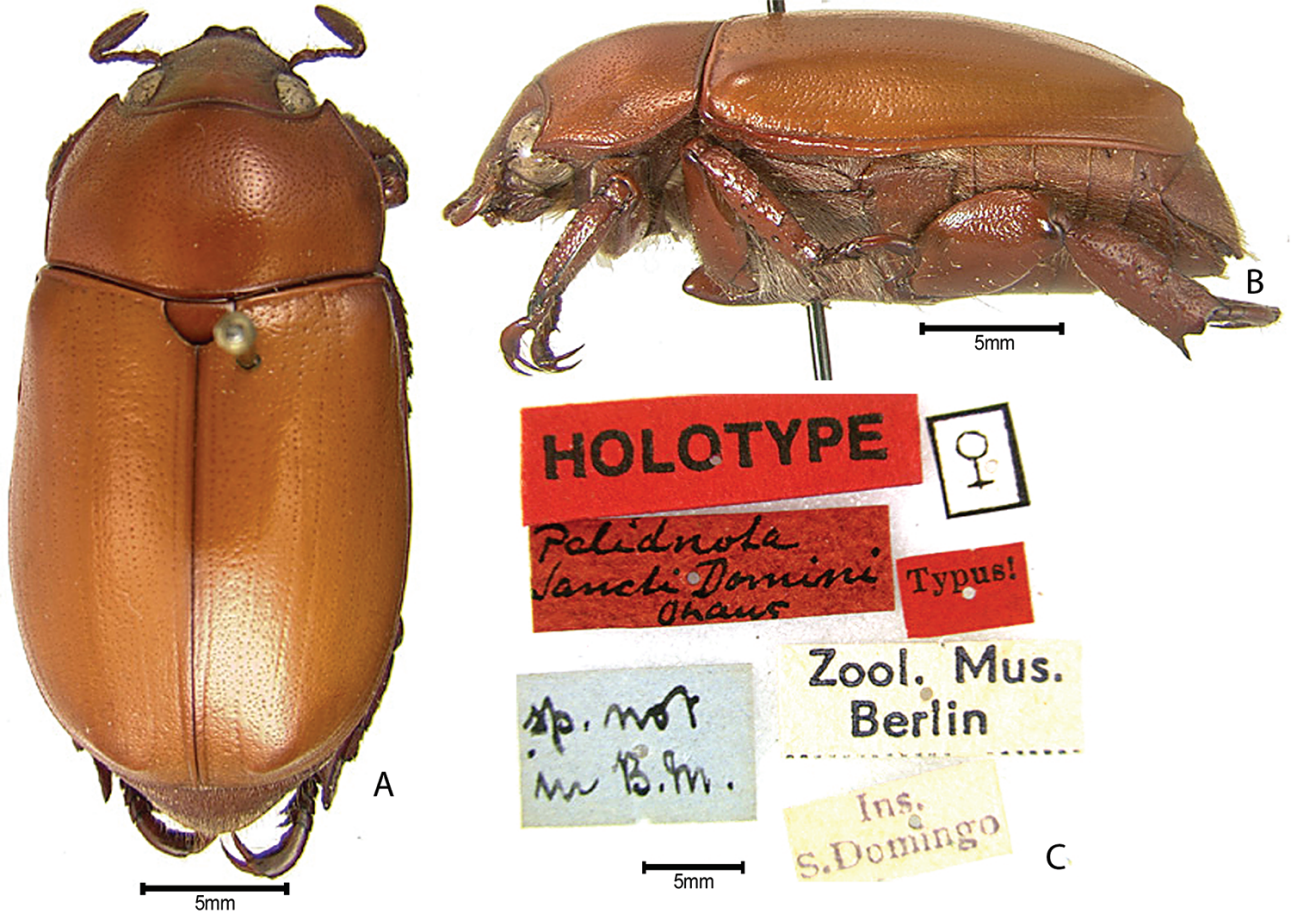
*Pelidnota sanctidomini sanctidomini* Ohaus holotype female from ZMHB. **A** Dorsal habitus **B** Lateral habitus **C** Specimen labels.

1 ♀ holoype specimen of *Pelidnota sanctidomini sanctidomini* Ohaus is deposited at ZMHB (Fig. 86). 1 ♂ syntype specimen of *Pelidnota pubes* Ohaus at ZMHB (Fig. 85).

#### Remarks.

Ohaus (1905) compared *P. sanctidomini* with *P. prolixa*. He based the species description on a single female specimen from “Santo Domingo Island”, referring to the Caribbean island of Hispanola. Soula (2006) synonymized *S. pubes* and *S. hirsutiphallica* (erroneously called “hirsutipenis”) with *P. sanctidomini*. The synonymy of *P. hirustiphallica* was later retracted (Soula 2010a). Soula (2006) also created a new subspecies of *P. sanctidomini, S. sanctidominicaliensis*, from Cali, Colombia. This species has erroneously been reported from the West Indies (e.g., from Haiti [Leng and Mutchler 1914]). The validity of these taxa and *P. hirsutiphallica* requires further study.

### Pelidnota satipoensis

Taxon classificationAnimaliaColeopteraScarabaeidae

Demez & Soula, 2010

Pelidnota satipoensis Demez & Soula, 2010: 60–61 [original combination].

#### Distribution.

PERU: Junín (Soula 2010a, Ratcliffe et al. 2015).

#### Types.

The following specimens are deposited at CCECL. 1 ♂ holotype, 1 ♀ allotype, 1 ♂ paratype: “Rio Tambo Val Paraiso Tuncana M. SOULA det. 19 [obverse] XI/2007//Holotype *Pelidnota satipoensis* S. 2010 Soula” (47030077); “Rio Tambo Val Paraison (sic!) Tuncana M. SOULA det. 19 [obverse] Junin XI/2007//Allotype *Pelidnota satipoensis* S. 2010 Soula” (47030078); “Satipo, Junin Rio Negro, 1-15/IV/2010//Paratype *Pelidnota satipoensis* S. 2010 Soula” (47030079). Genitalia card-mounted underneath the male holotype and paratype specimens. Box 4618650 SOULA.

### Pelidnota semiaurata citripennis

Taxon classificationAnimaliaColeopteraScarabaeidae

Ohaus, 1900

Pelidnota aeuruginosa citripennis Ohaus, 1900: 185 [original combination].
Pelidnota (Pelidnota) aeruginosa citripennis Ohaus [new subgeneric combination by Ohaus 1918: 22].
Pelidnota semiaurata var. citripennis Ohaus [removal of subgeneric classification and new infrasubspecific status by Soula 2009: 77].
Pelidnota semiaurata citripennis Ohaus [revised subspecific status by Moore and Jameson 2013: 384].

#### Distribution.

BRAZIL: Minas Gerais, Rio Grande do Sol (Blackwelder 1944, Machatschke 1972, Frietas et al. 2002, Krajcik 2008, Bernardi et al. 2010, Moore and Jameson 2013).

#### Remarks.

Both subspecific and infrasubspecific names (e.g., varieties) are used by Soula (2009). Because both are entities used in this work, variety names should be considered unambiguously infrasubspecific (ICZN Article 45.6.1). To stabilize nomenclature, Pelidnota semiaurata var. citripennis (see Soula 2009: 77) was elevated to *Pelidnota semiaurata citripennis* (Moore and Jameson 2013).

### Pelidnota semiaurata semiaurata

Taxon classificationAnimaliaColeopteraScarabaeidae

Burmeister, 1844

Pelidnota glauca var. semiaurata Burmeister, 1844: 402 [original combination].
Pelidnota (Pelidnota) aeruginosa semiaurata Burmeister [new subgeneric combination and new subspecific status by Ohaus 1918: 22].
Pelidnota semiaurata Burmeister [removal of subgeneric classification and new species status by Soula 2009: 76].
Pelidnota semiaurata semiaurata Burmeister [**revised status**].

#### Distribution.

BRAZIL: Rio de Janeiro (INPA), Rio Grande do Sol, Santa Catarina (Ohaus 1918, 1934b, Machatschke 1972, Krajcik 2008, Soula 2009).

#### Types.

1 ♂ lectotype and 1 paralectotype at ZMHB (Soula 2009).

### Pelidnota sericeicollis

Taxon classificationAnimaliaColeopteraScarabaeidae

Frey, 1976

Pelidnota (Ganonota) sericeicollis Frey, 1976: 344 [original combination].
Strigidia sericeicollis (Frey) [new combination by Soula 2006: 18–19].
Pelidnota (Strigidia) sericeicollis Frey [revised combination and new subspecific combination by Özdikmen 2009: 145].
Pelidnota sericeicollis Frey [removal of subgeneric classification by Soula 2009: 116].

#### Distribution.

BRAZIL: Bahia (Frey 1976, Soula 2006, Krajcik 2008).

#### Types.

Soula (2006) recorded 1 ♂ holotype and some paratypes, but he did not clearly indicate the depository. It is possible that these specimens reside at NHMB.

### Pelidnota sikorskii

Taxon classificationAnimaliaColeopteraScarabaeidae

(Soula, 2006)

Strigidia sikorskii Soula, 2006: 10, 20-21 [original combination].
Pelidnota sikorskii (Soula) [new combination by Soula 2009: 116].

#### Distribution.

BRAZIL: Bahia (Soula 2006).

#### Types.

The following specimen is deposited at CCECL. 1 ♂ paratype: “Cachimbo Prov.de.Bahia Ch Pujol 1890//Museum Paris ex Coll. R. Oberthur//Paratype *Strigidia sikorskii* S. 2006 Soula” (47030431). Genitalia mounted underneath the male paratype. Box 4618663 SOULA.

### Pelidnota soederstroemi

Taxon classificationAnimaliaColeopteraScarabaeidae

Ohaus, 1908

Pelidnota söderströmi Ohaus, 1908b: 402–403 [original combination].
Pelidnota Ganonota söderströmi Ohaus [new subgeneric combination by Ohaus 1918: 26].
Pelidnota Strigidia söderströmi Ohaus [new subgeneric combination by Machatschke 1970: 157].
Pelidnota (Strigidia) soederstroemi Ohaus [justified emendation by Machatschke 1972: 28].
Pelidnota (Odontognathus) soederstroemi Ohaus [new subgeneric combination by Hardy 1975: 4].
Strigidia soederstroemi (Ohaus) [new combination by Soula 2006: 79–80].
Pelidnota (Strigidia) soederstroemi Ohaus [revised combination and revised subgeneric combination by Özdikmen 2009: 145].
Pelidnota soederstroemi Ohaus [removal of subgeneric classification by Soula 2009: 116].

#### Distribution.

ECUADOR: Cotopaxi, Pichincha (Ohaus 1908b, 1918, 1934b, Blackwelder 1944, Machatschke 1972, Paucar-Cabrera 2005, Soula 2006, Krajcik 2008).

#### Types.

**Figure 87.**
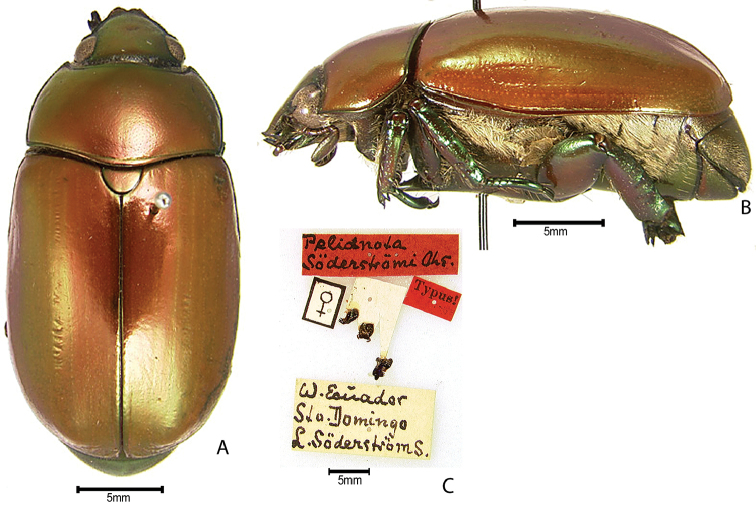
*Pelidnota söderströmi* Ohaus (valid name *Pelidnota soederstroemi* Ohaus) holotype female from ZMHB. **A** Dorsal habitus **B** Lateral habitus **C** Specimen labels and mouthparts.

Holotype ♀ of Pelidnota (Strigidia) soederstroemi at ZMHB (Fig. 87) with labels: a) “W. Ecuador Sto. Domingo L. Söderström S.” (handwritten, white label), b) “[female symbol]” (typeset with black border), c) mouthparts card mounted, d) “Typus!” (red label, type set), e) Pelidnota Söderströmi Ohs.” (red label, handwritten). The specimen is lacking one protarsus, one mesothoracic leg, and both metatarsi.

#### Remarks.

Ohaus (1908b) based his description on one female specimen. Because the label data match the original description, we refer to this specimen as a holotype. Ohaus dedicated the species name to Ludwig Söderström in Quito who collected the specimen en route from Quito to Manabi.

### Pelidnota sordida

Taxon classificationAnimaliaColeopteraScarabaeidae

(Germar, 1824)

Rutela sordida Germar, 1824: 118–119 [original combination].
Pelidnota sordida (Germar) [new combination by Burmeister 1844: 404].
Pelidnota (Pelidnota) sordida (Germar) [new subgeneric combination by Ohaus 1918: 24].
Pelidnota sordida (Germar) [removal of subgeneric classification by Soula 2009: 75–76].

#### Distribution.

ARGENTINA (Ohaus 1918, 1934b, Blackwelder 1944, Machatschke 1972). BRAZIL: Bahia, Goiás, Minas Gerais, Parana, Rio de Janeiro, São Paulo (Burmeister 1844, 1855, Ohaus 1908a, 1918, 1934b, Guimarães 1944, Blackwelder 1944, Machatschke 1972, Krajcik 2008, Soula 2009). PARAGUAY (Ohaus 1918, 1934b, Blackwelder 1944, Machatschke 1972).

#### Types.

1 ♂ lectotype and 2 paralectotypes at ZMHB (Soula 2009).

### Pelidnota striatopunctata

Taxon classificationAnimaliaColeopteraScarabaeidae

(Kirsch, 1885)

Odontognathus striatopunctatus Kirsch, 1885: 222 [original combination].
Pelidnota (Ganonota) striatopunctata (Kirsch) [new combination and new subgeneric combination Ohaus 1918: 26].
Pelidnota (Strigidia) striatopunctata (Kirsch) [new subgeneric combination by Machatschke 1970: 157].
Pelidnota (Odontognathus) striatopunctata (Kirsch) [new subgeneric combination by Hardy 1975: 4].
Strigidia striatopunctata (Kirsch) [new combination by Soula 2006: 18].
Pelidnota (Strigidia) striatopunctata (Kirsch) [revised combination and revised subgeneric combination by Özdikmen 2009: 145].
Pelidnota striatopunctata (Kirsch) [removal of subgeneric classification by Soula 2009: 116].

#### Distribution.

BOLIVIA: La Paz (Ohaus 1918, 1934b, Blackwelder 1944, Machatschke 1972, Soula 2006, Krajcik 2008).

#### Types.

1 ♀ syntype of *Odontognathus striatopunctatus* at MTD (Soula 2006).

#### Remarks.

CCECL contains a *P. striatopunctata* specimen labeled as a male alloréférent with the following data: 1 ♂ alloréférent: “Prov. de La Paz XI/2001 M. SOULA det 19 [obverse] Bolivie//Alloréferent ♂ de *Strigidia striatopunctata* (K.) M. SOULA det 19” (47030121). Genitalia card-mounted underneath the alloréférent male. Box 1418652 SOULA.

### Pelidnota strigosa

Taxon classificationAnimaliaColeopteraScarabaeidae

Laporte, 1840

Pelidnota strigosa Laporte, 1840: 122 [original combination].
Pelidnota (Pelidnotidia) strigosa Laporte [new subgeneric combination by Casey 1915: 78].
Pelidnota (Pelidnota) strigosa Laporte [new subgeneric combination by Ohaus 1918: 24].
Pelidnota strigosa Laporte [removal of subgeneric classification by Soula 2009: 57].
Pelidnota (Pelidnotidia) cuprascens Casey, 1915 **synonym.**
Pelidnota (Pelidnotidia) cuprascens Casey, 1915: 78 [original combination].
Pelidnota (Pelidnota) cuprascens Casey [new subgeneric combination by Ohaus 1934b: 79].
Pelidnota (Pelidnota) strigosa Laporte [syn. by Hardy 1975: 18].
Pelidnota (Pelidnotidia) obscurella Casey, 1915 **synonym.**
Pelidnota (Pelidnotidia) obscurella Casey, 1915: 79 [original combination].
Pelidnota (Pelidnota) obscurella Casey [new subgeneric combination by Ohaus 1934b: 80].
Pelidnota (Pelidnota) strigosa Laporte [syn. by Hardy 1975: 18].
Pelidnota (Pelidnotidia) refulgens Casey, 1915 **synonym.**
Pelidnota (Pelidnotidia) refulgens Casey, 1915: 79 [original combination].
Pelidnota (Pelidnota) refulgens Casey [new subgeneric combination by Ohaus 1934b: 81].
Pelidnota (Pelidnota) strigosa Laporte [syn. by Hardy 1975: 18].

#### Distribution.

BELIZE (Hardy 1975, Delgado-Castillo and Márquez 2006). COLOMBIA: Córdoba (Casey 1915, Ohaus 1934b, Blackwelder 1944, Machatschke 1972, Hardy 1975, Restrepo et al. 2003, Krajcik 2008, Pardo-Locarno et al. 2012). COSTA RICA: Alajuela, Guanacaste, Puntarenas, San José (H. W. Bates 1888, Ohaus 1934b, Blackwelder 1944, Machatschke 1972, Hardy 1975, Maes 1987, Solís and Morón 1994, Delgado-Castillo and Márquez 2006). EL SALVADOR: Cuscatlán, La Libertad, La Unión, San Salvador, Santa Ana (Hardy 1975, Delgado-Castillo and Márquez 2006). GUATEMALA: Escuintla, Izabal, San Marcos (H. W. Bates 1888, Ohaus 1934b, Blackwelder 1944, Casey 1915, Machatschke 1972, Hardy 1975, Maes 1987, Delgado-Castillo and Márquez 2006, Krajcik 2008). HONDURAS: Atlántida, Copán, Cortés, Francisco Morazán, Gracias a Dios (Blackwelder 1944, Machatschke 1972, Hardy 1975, Delgado-Castillo and Márquez 2006, Krajcik 2008). MEXICO: Campeche, Chiapas, Coahuila, Distrito Federal, Hidalgo, Oaxaca, Puebla, San Luis Potosi, Tabasco, Tamaulipas, Veracruz (Laporte 1840, Blanchard 1851, H. W. Bates 1888, Ohaus 1918, 1934b, Blackwelder 1944, Carrillo et al. 1966, Machatschke 1972, Hardy 1975, Morón 1979, Maes 1987, Thomas 1993, Lobo and Morón 1993, Morón 1993, 1994, Morón et al. 1997, Sánchez-Soto 1997, Carrillo-Ruiz and Morón 2003, Delgado-Castillo and Márquez 2006, Krajcik 2008, Pacheco Flores et al. 2008, Soula 2009, Delgado-Castillo et al. 2012, Rivera-Gasperín et al. 2013). NICARAGUA: Boaco, Carazo, Chontales, León, Managua, Matagalpa (H. W. Bates 1888, Ohaus 1934b, Blackwelder 1944, Machatschke 1972, Hardy 1975, Maes 1987, Delgado-Castillo and Márquez 2006). PANAMA: Chiriquí, Coclé, Darien, Former Canal Zone, Herrera, Los Santos, Panama, Veraguas (H. W. Bates 1888, Ohaus 1918, 1934b, Blackwelder 1944, Machatschke 1972, Hardy 1975, Maes 1987, Ratcliffe 2002, Delgado-Castillo and Márquez 2006, Soula 2009). VENEZUELA (Hardy 1975, Morón 1979, Maes 1987, Delgado-Castillo and Márquez 2006).

#### Types.

1 ♂ neotype of *Pelidnota strigosa* at MNHN (Soula 2009).

#### Remarks.

CCECL contains a specimen of *P. strigosa* that is labeled as a female alloréférent with the following data: 1 ♀ alloréférent: “Soteapan 500 m Vera Cruz, Mex M. SOULA det 19 [obverse] VIII/2006//Alloréferent ♀ de *Strigidia strigosa* (Lap.) M. SOULA det 19” (47030472). Genitalia card-mounted underneath the alloréférent female. Box 1418665 SOULA.

### Pelidnota subandina orellanai

Taxon classificationAnimaliaColeopteraScarabaeidae

(Soula, 2006)

Strigidia subandina orellanai Soula, 2006: 58-59 [original combination].
Pelidnota subandina orellanai (Soula) [new combination by Soula 2009: 115].

#### Distribution.

VENEZUELA: Barinas, Táchira (Soula 2006).

#### Types.

1 ♂ holotype, 1 ♀ allotype and paratypes of *Strigidia subandina orellanai* at MIZA (Soula 2006).

### Pelidnota subandina subandina

Taxon classificationAnimaliaColeopteraScarabaeidae

Ohaus, 1905

Pelidnota subandina Ohaus, 1905: 316 [original combination].
Pelidnota (Chalcoplethis) subandina Ohaus [new subgeneric combination by Ohaus 1918: 28].
Strigidia subandina (Ohaus) [new combination by Soula 2006: 58-59].
Pelidnota subandina subandina Ohaus [removal of subgeneric classification and new subspecific status by Soula 2009: 116].

#### Distribution.

BRAZIL: Amazonas (Ohaus 1905, 1918, 1934b, Blackwelder 1944, Machatschke 1972, Soula 2006, Krajcik 2008). ECUADOR: Morona-Santiago (Blackwelder 1944, Ohaus 1918, 1934b, 1952, Paucar-Cabrera 2005). PERU: San Martín (Ohaus 1905, 1934b, Blackwelder 1944, Ohaus 1905, 1918, 1952, Machatschke 1972, Soula 2006, Ratcliffe et al. 2015).

#### Types.

**Figure 88.**
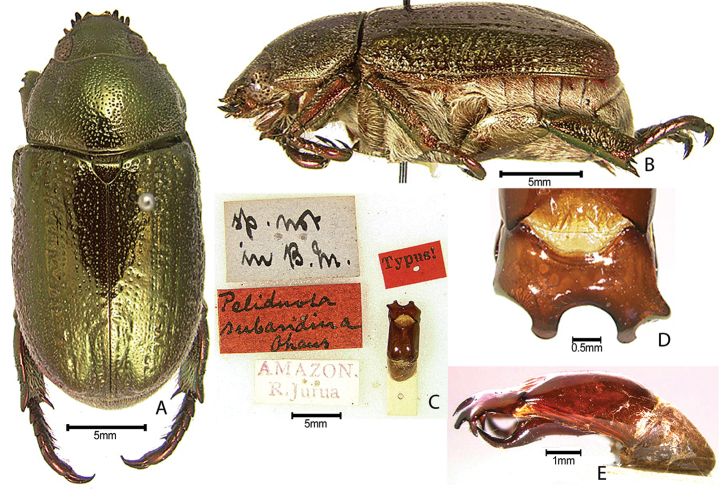
*Pelidnota subandina* Ohaus (valid name *Pelidnota subandina subandina* Ohaus) syntype male from ZMHB. **A** Dorsal habitus **B** Lateral habitus **C** Specimen labels and male genitalia **D** Male parameres, dorsal view **E** Male genitalia, lateral view.

1 ♂ syntype specimen of *Pelidnota subandina* Ohaus at ZMHB (Fig. 88).

### Pelidnota sumptuosa

Taxon classificationAnimaliaColeopteraScarabaeidae

(Vigors, 1825)

Rutela sumptuosa Vigors, 1825: 542 [original combination].
Pelidnota sumtuosa (Vigors) (*sic*) [new combination by Burmeister 1844: 406–407].
Pelidnota (Pelidnota) sumptuosa (Vigors) [new subgeneric combination by Ohaus 1918: 25].
Pelidnota (Pelidnota) ludovici Ohaus [syn. by Machatschke 1972: 25].
Pelidnota sumptuosa (Vigors) [removal of subgeneric classification and revised species status by Soula 2009: 41–42].
Rutela smaragdina Perty, 1830 **synonym.**
Rutela smaragdina Perty, 1830: 50 [original combination].
Pelidnota sumtuosa (Vigors) (*sic*) [syn. by Burmeister 1844: 406–407].
Pelidnota (Pelidnota) luxuriosa Blackwelder [syn. by Machatschke 1972: 26].
Pelidnota sumptuosa (Vigors) [syn. by Soula 2009: 41].
Rutela smaragdina var. plicata Gory, 1846 **synonym.**
Rutela smaragdina var. plicata Gory, 1846: 192 [original combination].
Pelidnota sumptuosa (Vigors) [syn. by Harold 1869b: 1223].
Pelidnota (Pelidnota) luxuriosa Blackwelder [syn. by Machatschke 1972: 25].
Pelidnota sumptuosa (Vigors) [syn. by Soula 2009: 41].

#### Distribution.

BRAZIL: Bahia, Goiás, Mato Grosso, Minas Gerais, Pará, São Paulo (Vigors 1825, Burmeister 1844, 1855, Blanchard 1851, Ohaus 1918, 1934b, Blackwelder 1944, Machatschke 1972, Krajcik 2008, Soula 2009). COLOMBIA: Caquetá, Meta (Restrepo et al. 2003, Soula 2009, Pardo-Locarno et al. 2011). PARAGUAY (Soula 2009).

#### Types.

1 ♀ holotype of *Rutela sumptuosa* at BMNH (Soula 2009).

#### Remarks.

*Pelidnota sumptuosa* and *P. cyanitarsis* are superficially similar and have been confused in collections and the literature. Both species are bright metallic blue, green, or blue-green with enlarged metatibia in male specimens. Several characters serve to separate these species (see “Remarks” for *P. cyanitarsis*), and male parameres are also diagnostic (see Soula 2009: 41 and 42). Label data indicate that adults have been found feeding on flowers of *Miconia albicans* (Sw.) Steud. () in the month of October.

### Pelidnota teocuitlamayatli

Taxon classificationAnimaliaColeopteraScarabaeidae

Delgado-Castillo, Deloya, & Morón, 1988

Pelidnota (Pelidnota) teocuitlamayatli Delgado-Castillo, Deloya, & Morón, 1988: 132, 139–141 [original combination].
Pelidnota teocuitlamayatli Delgado-Castillo, Deloya, and Morón [removal of subgeneric classification by Soula 2009: 36–37].

#### Distribution.

MEXICO: Guerrero (Delgado-Castillo et al. 1988, Krajcik 2008, Soula 2009, Deloya et al. 2014).

#### Types.

The following specimen is deposited at CMNC. 1 ♂ holotype: “24 mi. south Iguala Gro. MEXICO VII 18 1963//H. & A. Howden Collection//HOLOTIPO//*Pelidnota* ♂ *teocuitlamayatli* Delgado, Deloya, Morón 1988. L.L. Delgado det. 1988.//CMNEN 1999-0383”.

#### Remarks.

This species is metallic silver and thus it strongly resembles species in the genus *Chrysina*.

### Pelidnota testaceovirens felipemezai

Taxon classificationAnimaliaColeopteraScarabaeidae

(Soula, 2006)

Strigidia testaceovirens felipemezai Soula, 2006: 62 [original combination].
Pelidnota (Strigidia) testaceovirens felipemezai (Soula) [new combination and new subgeneric combination by Özdikmen 2009: 145].
Pelidnota testaceovirens felipemezai (Soula) [removal of subgeneric classification by Soula 2009: 115].

#### Distribution.

PERU: Junín (Soula 2006, Ratcliffe et al. 2015).

#### Types.

The following specimens are deposited at CCECL. 10 ♂ paratypes, 4 ♀ paratypes: six paratypes with identical label data “Satipo, Junin Pérou, X/2003//Paratype 2006 *Strigidia testaceovirens felipemezai* S. Soula” (47030080 to 47030084, exch02); “Satipo, Junin Pérou, X/XI/2002//Paratype 2006 *Strigidia testaceovirens felipemezai* S. Soula” (47030085); “Satipo Pérou IX/2003 M. SOULA det. 19//Paratype *Pelidnota testaceovirens mezai* S. Soula” (47030086); “Satipo Junin Pérou M. SOULA det. 19//Paratype 2006 *Strigidia testaceovirens felipemezai* S. Soula” (47030087); “Satipo XI/2007 M. SOULA det. 19//Paratype 2004 *Strigidia testaceovirens felipemezai* S. Soula” (47030088); “Satipo (P) 10/88//Paratype 2006 *Strigidia testaceovirens felipemezai* S. Soula” (47030089); “Satipo E. Peru Dec. 2002 //Paratype 2006 *Strigidia testaceovirens felipemezai* S. Soula” (47030090); two paratypes with identical label data “Pérou Chanchamayo La Merced C. O. Schunke Recu Novembre 1904//Paratype 2006 *Strigidia testaceovirens felipemezai* S. Soula” (47030091 and 47030092). Genitalia card-mounted underneath five male paratypes and one female paratype. Box 4618651 SOULA.

### Pelidnota testaceovirens noaensis

Taxon classificationAnimaliaColeopteraScarabaeidae

Soula, 2009

Pelidnota testaceovirens noaensis Soula, 2009: 134 [original combination].

#### Distribution.

ARGENTINA: Jujuy (Soula 2009).

#### Types.

The following specimens are deposited at CCECL. 1 ♂ holotype, 1 ♀ allotype, 6 ♂ paratypes, 2 ♀ paratypes: “Calilegua, 1110m NOA, 26/01/06 Leg. P. Schmit//Holotype 2008 *Pelidnota testaceovirens noaensis* Soula” (47030111); “Calilegua, 1110m NOA, 26/01/06 Leg. P. Schmit//Allotype 2008 *Pelidnota testaceovirens noaensis* S. Soula” (47030112); eight paratypes with identical label data “Calilegua, 1110m NOA, 26/01/06 Leg. P. Schmit//Paratype 2008 *Pelidnota testaceovirens noaensis* Soula” (47030113 to 47030119, exch05). Genitalia card-mounted underneath male holotype. Box 4618651 SOULA.

### Pelidnota testaceovirens testaceovirens

Taxon classificationAnimaliaColeopteraScarabaeidae

Blanchard, 1851

Pelidnota testaceovirens Blanchard, 1851: 213 [original combination].
Pelidnota (Ganonota) testaceovirens Blanchard [new subgeneric combination by Ohaus 1918: 26].
Pelidnota (Strigidia) testaceovirens Blanchard [new subgeneric combination by Machatschke 1970: 157].
Pelidnota (Odontognathus) testaceovirens Blanchard [new subgeneric combination by Hardy 1975: 4].
Strigidia testaceovirens (Blanchard) [new combination by Soula 2006: 60–61].
Pelidnota (Strigidia) testaceovirens Blanchard [revised combination and revised subgeneric combination by Özdikmen 2009: 145].
Pelidnota testaceovirens testaceovirens Blanchard [removal of subgeneric classification and new subspecies status by Soula 2009: 116].

#### Distribution.

BOLIVIA: La Paz, Santa Cruz (Blanchard 1851, Ohaus 1918, 1934b, 1952, Machatschke 1972, Soula 2006, Krajcik 2008). BRAZIL: Goiás, Mato Grosso, São Paulo (Ohaus 1918, 1952, Machatschke 1972). PERU (Ohaus 1918, 1934b, Machatschke 1972).

#### Types.

1 ♀ lectotype at MNHN (Soula 2006).

### Pelidnota testaceovirens vittipennis

Taxon classificationAnimaliaColeopteraScarabaeidae

F. Bates, 1904

Pelidnota vittipennis F. Bates, 1904: 256, 264 [original combination].
Pelidnota testaceovirens Blanchard [syn. by Ohaus 1905: 316].
Strigidia testaceovirens vittipennis (F. Bates) [new combination and new subspecies status by Soula 2006: 61–62].
Pelidnota (Strigidia) testaceovirens vittipennis F. Bates [revised combination and new subgeneric combination by Özdikmen 2009: 145].
Pelidnota testaceovirens vittipennis F. Bates [removal of subgeneric classification by Soula 2009: 116].

#### Distribution.

ARGENTINA (Ohaus 1905). BOLIVIA (Ohaus 1905). BRAZIL: Goiás (F. Bates 1904, Ohaus 1905, 1934b, Machatschke 1972, Soula 2006, Krajcik 2008).

#### Types.

**Figure 89.**
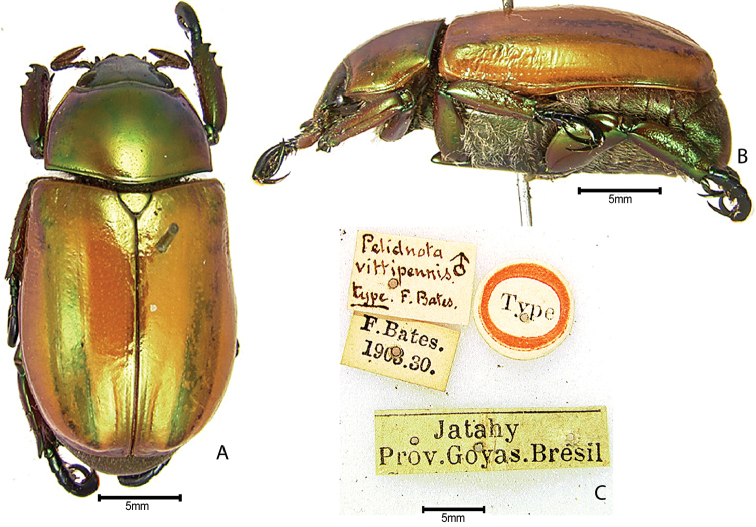
*Pelidnota vittipennis* F. Bates (valid name *Pelidnota testaceovirens vittipennis* F. Bates) syntype male from BMNH. **A** Dorsal habitus **B** Lateral habitus **C** Specimen labels.

1 ♂ syntype specimen of *Pelidnota vittipennis* F. Bates deposited at BMNH (Fig. 89).

### Pelidnota testaceovirens xinguensis

Taxon classificationAnimaliaColeopteraScarabaeidae

(Soula, 2006)

Strigidia testaceovirens xinguensis Soula, 2006: 62-63 [original combination].
Pelidnota (Strigidia) testaceovirens xinguensis (Soula) [new combination and new subgeneric combination by Özdikmen 2009:145].
Pelidnota testaceovirens xinguensis (Soula) [removal of subgeneric classification by Soula 2009: 116].

#### Distribution.

BRAZIL: Pará (Soula 2006).

#### Types.

The following specimen is deposited at CCECL. 1 ♂ holotype: “SAO FELIX DO XINGU 29-30-IX-1975//Holotype 2006 *Strigidia testaceovirens xinguensis* S. Soula” (47030120). Genitalia card-mounted underneath male holotype. Box 4618651 SOULA.

### Pelidnota thiliezi

Taxon classificationAnimaliaColeopteraScarabaeidae

Soula, 2009

Pelidnota thiliezi Soula, 2009: 34, 112–113 [original combination].

#### Distribution.

BRAZIL: Goiás (Soula 2009).

#### Types.

**Figure 90.**
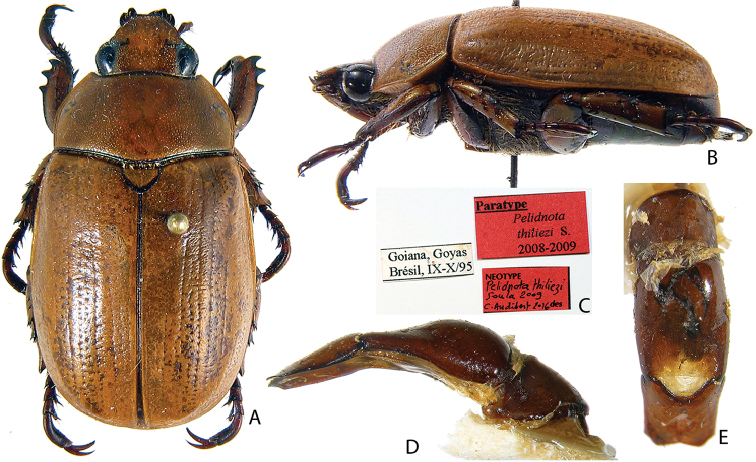
*Pelidnota thiliezi* male, neotype from CCECL. **A** Dorsal habitus **B** Lateral habitus **C** Specimen labels **D** Male genitalia, lateral view **E** Male genitalia, dorsal view.

The following specimens are deposited at CCECL. 1 ♂ neotype, 25 ♂ paratypes, 9 ♀ paratypes, 2 probable ♂ paratypes: 21 paratypes with identical label data: “Goiana, Goyas Brésil, IX-X/95//Paratype *Pelidnota thiliezi* S. 2008-2009” (47030732 to 47030748, exch44 to exch47); “Goiana, Goyas Brésil, IX-X/95//Paratype *Pelidnota grossiorum* S. 2008-2009//Probable *Pelidnota thiliezi* C. Audibert 2016” (47030749); “Goias Goiañia coll. – SOULA//Paratype *Pelidnota grossiorum* S. 2008-2009//Probable *Pelidnota thiliezi* C. Audibert 2016” (47030750); five paratypes with identical label data: “Goias Goiañia coll. – SOULA//Paratype *Pelidnota thiliezi* S. 2008-2009” (47030751 to 47030755); three paratypes with identical label data: “Goias Goiañia 11/93 coll. – SOULA//Paratype *Pelidnota thiliezi* S. 2008-2009” (47030756 to 47030758); “Goiar K.P. Klausen 30/11 15//ZOOL. MUSEUM DK COPENHAGEN//Paratype *Pelidnota thiliezi* S. 2008-2009” (47030759); two paratypes with identical label data: “Goiar K.P. Klausen 30/11 1915//ZOOL. MUSEUM DK COPENHAGEN//Paratype *Pelidnota thiliezi* S. 2008-2009” (47030760 to 47030761). **Neotype here designated and deposited at CCECL**: “Goiana, Goyas Brésil, IX-X/95//Paratype *Pelidnota thiliezi* S. 2008-2009//Neotype *Pelidnota thiliezi* Soula 2009 C. Audibert des.” (47030731) (Fig. 90). Genitalia card-mounted underneath the male neotype and eight male paratypes. Box 4618680 SOULA. The following specimens are deposited at CMNC. 5 ♂ paratypes: “BRASIL GOIAS Jatay Oliveira – leg. Coll. Martínez. Nov.-972//H. & A. HOWDEN COLLECTION *ex.* A. Martinez coll.//Paratype *Pelidnota thiliezi* S. 2008-2009”.

**Diagnosis.** Soula (2009) described *P. thiliezi* as a “population” from Goías, Brazil that is “close to” *P. estebanabadiei* from Brazil, Ecuador, and Colombia. Soula (2009) stated that *P. thiliezi* shares similarities with *P. ancilla* (Fig. 53), and it differs only in a few characters which we consider to be highly variable within species. According to Soula (2009), *P. thiliezi* is darker than *P. ancilla*. In comparison to *P. ancilla*, the clypeus of the male is “rather short, subtrapezoidal, with a subtruncate anterior margin, somewhat large, a little reflexed, and quite distinctly concave from behind” and the clypeus of the female is “more elongated, broadly parabolic rather than truncate” (Soula 2009: 112, translated from French). In addition, Soula (2009) stated that the mandibular teeth of the male are smaller than those of *P. estebanabadiei*.

#### Remarks.

Soula (2009) indicated that he deposited the holotype specimen of *P. thiliezi* at CCECL. Our study of the pelidnotine specimens at CCECL revealed that the holotype of this species is missing and it is presumed lost. We designated a neotype at CCECL (data above) from Soula’s (2009) paratype series in order to clarify the taxonomic status of *P. thiliezi* and to secure the stability of nomenclature. The neotype is from the type locality of *P. thiliezi* (“Goiana, Goias”) (Soula 2009). We examined all of the available *Pelidnota thiliezi* paratype material at CCECL. Features of the neotype specimen correspond closely to the original description (Soula 2009), and the specimen shares the clypeal shape, coloration, and paramere morphology of the lost holotype.

### Pelidnota tibialis aenigmatica

Taxon classificationAnimaliaColeopteraScarabaeidae

(Soula, 2006)

Strigidia tibialis aenigmatica Soula, 2006: 47 [original combination].
Pelidnota (Strigidia) tibialis aenigmatica (Soula) [new combination and new subgeneric combination by Özdikmen 2009: 145].
Pelidnota tibialisaenigmatica (sic) (Soula) [removal of subgeneric classification by Soula 2009: 115].

#### Distribution.

BRAZIL (Soula 2006).

#### Types.

The following specimen is deposited at CCECL. 1 ♂ holotype: “Brésil. coll. – SOULA//Holotype 2006 *Strigidia tibialis incerta* S. Soula.//Holotype ♂ *Strigidia tibialis aenigmatica* Soula 2006: 47 det. M.R. Moore 2014 nec S. t. incerta” (47030318). Genitalia card-mounted underneath the male holotype. Box 4618660 SOULA.

#### Remarks.

The holotype specimen is labeled “*Pelidnota tibialis incerta*”, a name that is not found in the literature. We compared the holotype specimen (labeled “*Pelidnota tibialis incerta*”), description, and image (Soula 2006: 47), and we conclude that Soula mislabeled this specimen. The specimen is labeled “aenigmatica nec incerta” by MR Moore.

### Pelidnota tibialis pernambucoensis

Taxon classificationAnimaliaColeopteraScarabaeidae

(Soula, 2006)

Strigidia tibialis pernambucoensis Soula, 2006: 47 [original combination].
Pelidnota (Strigidia) tibialis pernambucoensis (Soula) [new combination and new subgeneric combination by Özdikmen 2009: 145].
Pelidnota tibialis pernambucoensis (Soula) [removal of subgeneric classification by Soula 2009: 115].

#### Distribution.

BRAZIL: Pernambuco (Soula 2006).

#### Types.

The following specimen is deposited at CCECL. 1 ♂ holotype: “Pernambuco Brésil M. SOULA det 19//Holotype 2006 *Strigidia tibialis pernambucoensis* Sou. Soula det.” (47030319). Genitalia card-mounted underneath the male holotype. Box 4618660 SOULA.

### Pelidnota tibialis tibialis

Taxon classificationAnimaliaColeopteraScarabaeidae

Burmeister, 1844

Pelidnota tibialis Burmeister, 1844: 396–397 [original combination].
Pelidnota (Ganonota) tibialis Burmeister [new subgeneric combination by Ohaus 1918: 27].
Pelidnota (Strigidia) tibialis Burmeister [new subgeneric combination by Machatschke 1970: 157].
Pelidnota (Odontognathus) tibialis Burmeister [new subgeneric combination by Hardy 1975: 4].
Strigidia tibialis (Burmeister) [new combination by Soula 2006: 45–46].
Pelidnota (Strigidia) tibialis Burmeister [revised combination and revised subgeneric combination by Özdikmen 2009: 145].
Pelidnota tibialis tibialis Burmeister [removal of subgeneric classification and new subspecies status by Soula 2009: 116].
Pelidnota zikani Ohaus, 1922 **synonym.**
Pelidnota zikani Ohaus, 1922: 324 [original combination].
Pelidnota (Ganonota) zikani Ohaus [new subgeneric combination Ohaus 1934b: 84].
Pelidnota (Strigidia) zikani Ohaus [new subgeneric combination by Machatschke 1970: 157].
Pelidnota (Odontognathus) zikani Ohaus [new subgeneric combination by Hardy 1975: 4].
Pelidnota (Ganonota) zikani Ohaus [revised subgeneric combination by Frey 1976: 346].
Strigidia tibialis (Ohaus) [syn. by Soula 2006: 46].
Pelidnota (Strigidia) zikani Ohaus [revised combination and revised subgeneric combination by Özdikmen 2009: 145].
Pelidnota tibialis tibialis Burmeister [**revised synonymy**].

#### Distribution.

BRAZIL: Minas Gerais, Rio de Janeiro (Burmeister 1844, Blanchard 1851, Harold 1869b, Ohaus 1918, 1922, 1934b, Blackwelder 1944, Machatschke 1972, Krajcik 2008).

#### Types.

**Figure 91.**
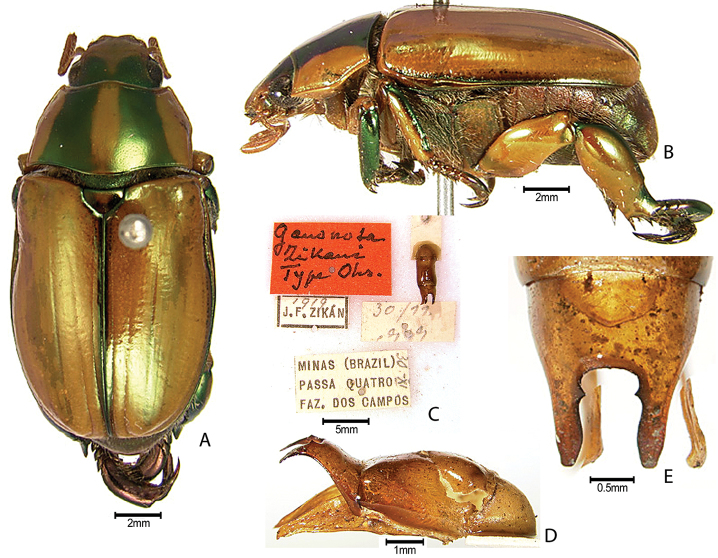
*Pelidnota zikani* Ohaus (valid name *Pelidnota tibialis tibialis* Burmeister) syntype male from ZMHB. **A** Dorsal habitus **B** Lateral habitus **C** Specimen labels and male genitalia **D** Male genitalia, lateral view **E** Male parameres, dorsal view.

1 ♂ lectotype of *P. tibialis tibialis* Burmeister at MLUH (Soula 2006). 1 ♂ syntype specimen of *Pelidnota zikani* is deposited at ZMHB (Fig. 91).

#### Remarks.

Özdikmen (2009) did not acknowledge Soula (2006) and listed P. (Strigidia) zikani (Ohaus) as a valid name. We follow Soula (2006) and consider *Pelidnota zikani* Ohaus a **revised synonym** under *P. tibialis tibialis* Burmeister.

### Pelidnota toulgoeti

Taxon classificationAnimaliaColeopteraScarabaeidae

(Soula, 2006)

Strigidia toulgoeti Soula, 2006: 11, 50–51 [original combination].
Pelidnota toulgoeti (Soula) [new combination by Soula 2009: 116].

#### Distribution.

PERU: Huánuco, Piura (Soula 2006, Ratcliffe et al. 2015).

#### Types.

The following specimens are deposited at CCECL. 1 ♂ holotype, 1 ♀ allotype, 1 ♀ paratype: “Carbajal, Rio Itaya, Piura Pérou 9/2005 M. SOULA det 19//Holotype 2006 *Strigidia toulgoueti* (*sic*) Sou. Soula” (47030434); “Carbajal, Rio Itaya Piura, Pérou, IX/2005//Allotype 2006 *Strigidia toulgoueti* (*sic*) Sou. Soula” (47030435); “Tingo Maria, Huanuco, Pérou 800m, III/2004//Paratype *Strigidia toulgoeti* Sou. Soula” (47030436). The genitalia are card-mounted underneath the male holotype and female paratype. Box 4618663 SOULA.

### Pelidnota touroulti

Taxon classificationAnimaliaColeopteraScarabaeidae

Soula, 2008

Pelidnota touroulti Soula, 2008: 37–38 [original combination].

#### Distribution.

FRENCH GUIANA (Soula 2008, 2010c).

#### Types.

**Figure 92.**
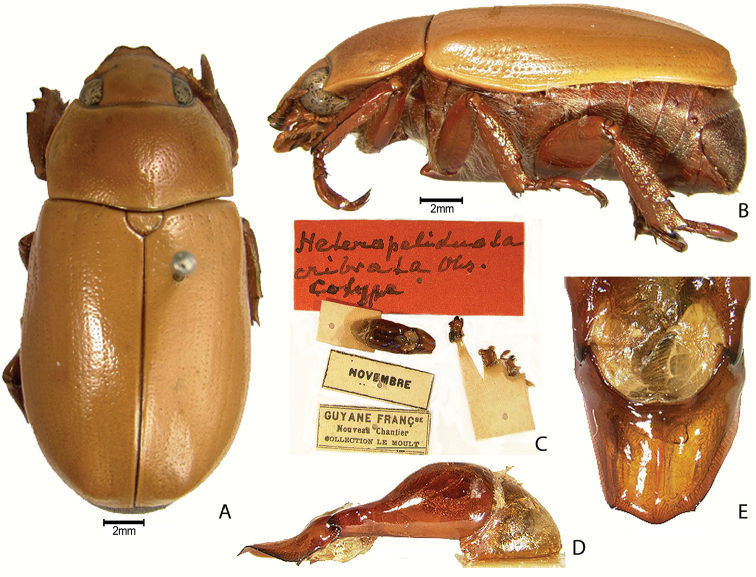
*Heteropelidnota cribrata* Ohaus paralectotype male (valid name *Pelidnota touroulti* Soula) from ZMHB. **A** Dorsal habitus **B** Lateral habitus **C** Specimen labels, mouthparts, and male genitalia **D** Male genitalia, lateral view **E** Male parameres, dorsal view.

2 ♂ paratypes (= paralectotypes of *Pelidnota cribrata* [Ohaus]) at ZMHB (Fig. 92). The following specimens are deposited at CCECL. 1 ♂ holotype, 1 ♀ allotype, 33 ♂ paratypes, 22 ♀ paratypes: “GUYANE FRANÇAISE Piste de Kaw pK 13 8-VIII-1996 H. de Toulgoët & J. Navatte réc.//Holotype 2007 *Pelidnota touroulti* S. Soula” (47030809); “Piste de Kaw 9/92//Allotype *Pelidnota touroulti* S. 2007 Soula” (47030810); two paratypes with identical label data: “Piste de Kaw G. F. 9/92//Paratype *Pelidnota touroulti* S. Soula det. 2007” (47030811 and 47030812); four paratypes with identical label data: “Piste de Kaw G. F. 8/92//Paratype *Pelidnota touroulti* S. Soula det. 2007” (47030813 to 47030816); “Piste de Kaw G. F. 7/92//Paratype *Pelidnota touroulti* S. Soula det. 2007” (47030817); “Piste de Kaw 8/92//Paratype *Pelidnota touroulti* S. Soula det. 2007” (47030818); “Piste de Kaw 25/7/87//Paratype *Pelidnota touroulti* S. Soula det. 2007” (47030819); two paratypes with identical label data: “K [Kaw] PK 40 25/8/84 P.L.//Paratype *Pelidnota touroulti* S. Soula det. 2007” (47030820 and 47030821); two paratypes with identical label data: “Kaw PK 34 P.L. 23/10/84//Paratype *Pelidnota touroulti* S. Soula det. 2007” (47030822 and 47030823); “KAW. PK 40 25/8/84 P.L.//Paratype *Pelidnota touroulti* S. 2007 Soula” (47030824); “KAW. PK 34 21/9/84 [obverse] P.L.//Paratype *Pelidnota touroulti* S. Soula det. 2007” (47030825); “Kaw 7/87//Paratype 2007 *Pelidnota touroulti* S. Soula det.” (47030826); “Piste de Kaw pk 45 P.L. 24/7/87 W.//Paratype *Pelidnota touroulti* S. 2007 Soula” (47030827); “M^gne^ de Kaw G. F. 8/92//Paratype *Pelidnota touroulti* S. 2007 Soula” (47030828); “Coll. P. BLEUZEN M^gne^ de KAW PK 37,5 GUYANE FR. 9 VIII 1985//Paratype 2007 *Pelidnota touroulti* S. Soula” (47030829); four paratypes with identical label data: “Pte de Kaw pk 37,5 G. F. 4/08/1997 M. SOULA det 19//Paratype *Pelidnota touroulti* S. 2007 Soula” (47030830 to 47030833); two paratypes with identical label data: “GUYANE FRANÇAISE Piste de Kaw pk 13 8-VIII-1996 H. de Toulgoët & J. Navatte réc.//Paratype *Pelidnota touroulti* S. Soula det. 2007” (47030834 and 47030835); “08/1997 P.K. 39-Rte de KAW GUYANE FRANCAISE FRENCH GUIANA//Paratype 2007 *Pelidnota touroulti* S. Soula” (47030836); “09/1997 P.K. 39-Rte de KAW GUYANE FRANCAISE FRENCH GUIANA//Paratype 2007 *Pelidnota touroulti* S. Soula” (47030837); two paratypes with identical label data: “Nancibo PK6 17/7/85 P.L.//Paratype *Pelidnota touroulti* S. Soula det. 2007” (47030838 and 47030839); two paratypes with identical label data: “FRG 19/7/85 [Nancibo] PK6 P.L.//Paratype *Pelidnota touroulti* S. Soula det. 2007” (47030840 and 47030841); “Nancibo VIII 84 etale le 15/9//Paratype *Pelidnota touroulti* S. Soula det. 2007” (47030842); two paratypes with identical label data: “Patagaïe G.F. 08/2001 M. SOULA det 19//Paratype 2007 *Pelidnota touroulti* S. Soula” (47030843 and 47030844); “Patagaïe G.F. 08/2001 M. SOULA det 2001//Paratype 2007 *Pelidnota touroulti* S. Soula” (47030845); “Bélizon Guyane Fr. M. SOULA det 19//Paratype *Pelidnota touroulti* S. 2007 Soula” (47030846); “Piste Plomb Pk 5 IX/2000 M. SOULA det 20//Paratype 2007 *Pelidnota touroulti* S. Soula det.” (47030847); two paratypes with identical label data: “Guyane franç. Est du départ. VIII/2001//Paratype *Pelidnota touroulti* S. Soula det. 2007” (47030848 and 47030849); “Cacao 7/87//Paratype 2007 *Pelidnota touroulti* S. Soula” (47030850); three paratypes with identical label data: “Cacao G. F. coll. – SOULA//Paratype 2007 *Pelidnota touroulti* S. Soula det.” (47030851 and 47030852, exch50); “S^t^ Georges VIII/87//Paratype *Pelidnota touroulti* S. 2007 Soula” (47030853); “Piste des eaux claires 7/92 G.F.//Paratype *Pelidnota touroulti* S. 2007 Soula” (47030854); “Coll. P. BLEUZEN Gonfolo Kourou GUYANE FR. 18/19 Juillet 1983//Paratype 2007 *Pelidnota touroulti* S. Soula det.” (47030855); “Barrage de Petit Saut Guyane 973 - 02/09/96 P. Cerdan leg.//Paratype 2005 *Pelidnota touroulti* S. Soula” (47030856); “Barrage de Petit Saut Guyane 973 - 30.09.97 P. Cerdan leg.//Piègeage lumineux//Paratype 2005 *Pelidnota touroulti* So. Soula” (47030857); “*H. cribrata* Piste Coralie 8/90 G.F.//Paratype *Pelidnota touroulti* S. Soula det. 2007” (47030858); “Dd Saramaca PK. 12 Rte des Compagnons Guyane Fse 7.X.1983 M. Duranton Recolt.//Paratype *Pelidnota touroulti* S. Soula det. 2007” (47030859); “GUYANE//Ohaus determ. *Pelidnota fracida* Bates//Paratype *Pelidnota touroulti* S. 2007 Soula” (47030860); two paratypes with identical label data: “G. française coll. – SOULA//Paratype *Pelidnota touroulti* S. 2007 Soula” (exch51 and exch52); “Guyane Franç. coll. – SOULA//Paratype *Pelidnota touroulti* S. 2007 Soula” (exch53); “Guyane française M. SOULA det 19//Paratype *Pelidnota touroulti* S. Soula det. 2007” (exch54). Genitalia card-mounted underneath the invalid male holotype and 23 male paratypes. Box 4618682 SOULA.

#### Remarks.

According to Soula (2008), the type series of *H. cribrata* included two, distinct species: the nominate species (*H. cribrata*) and a cryptic species that Soula referred to as *P. touroulti*. Soula compared *P. touroulti* with *P. cribrata* and *P. werneri* (see image in Soula 2008: 38). Soula assigned Ohaus’s two paralectotypes of *H. cribrata* with the new species *P. touroulti* (Soula 2008: 38, image in middle), and he apparently assigned the lectotype of *P. cribrata* with the paramere form on the left (Soula 2008: 38, image on left).

### Pelidnota ulianai

Taxon classificationAnimaliaColeopteraScarabaeidae

Soula, 2010

Pelidnota ulianai Soula, 2010a: 40-41 [original combination].

#### Distribution.

BOLIVIA: La Paz (Soula 2010a).

#### Types.

The following specimens are deposited at CCECL. 1 ♂ holotype, 1 ♀ allotype, 3 ♂ invalid paratypes, 4 ♀ invalid paratypes: “Inca Huara 1450 m. (Bo.) 11/94 coll. - SOULA//Holotype 2010 *Pelidnota ulianai* S. Soula” (47030215); “N. Yungas Bolivie coll. - SOULA//Allotype 2010 *Pelidnota ulianai* S. Soula” (47030216); “Route de Coroico à Coranavi [pro Caranavi] (Bolivie)//Paratype 2010 *Pelidnota ulianai* Soula//Invalid paratype see Soula 2010 det. M. R. Moore 2014” (47030217); “Région des Yungas Bolivie//Paratype 2010 *Pelidnota ulianai* Soula//Invalid paratype see Soula 2010 det. M. R. Moore 2014 ” (47030218); two paratypes with identical labels “Inca Huara 1400 m (BO.) coll. – SOULA//Paratype 2010 *Pelidnota ulianai* Soula//Invalid paratype see Soula 2010 det. M. R. Moore 2014” (47030219 and 47030220); two paratypes with identical labels “Caranavi [arrow] Tocumo [pro Yucumo ?] (860 m) coll. – SOULA//Paratype 2010 *Pelidnota ulianai* Soula//Invalid paratype See Soula 2010 det. M. R. Moore 2014” (47030221 and 47030222); “Yungas 1600 m 2/2003 M. SOULA det 19//Paratype 2010 *Pelidnota ulianai* Soula//Invalid Paratype See Soula 2010 det. M. R. Moore 2014” (47030223). Genitalia card-mounted underneath the holotype, allotype and 5 invalid paratypes. Box 4618656 SOULA.

#### Remarks.

There is no mention of a paratype series of *P. ulianai* in Soula (2010a). The paratype labels on these specimens are of a different style than the type labels on the holotype and allotype specimens. It is likely that these paratype labels were added after the publication of the name and are thus invalid paratypes.

### Pelidnota uncinata

Taxon classificationAnimaliaColeopteraScarabaeidae

Ohaus, 1930

Pelidnota uncinata Ohaus, 1930a: 139–140 [original combination].
Pelidnota (Ganonota) uncinata Ohaus [new subgeneric combination by Ohaus 1934b: 84].
Pelidnota (Strigidia) uncinata Ohaus [new subgeneric combination by Machatschke 1970: 157].
Pelidnota (Odontognathus) uncinata Ohaus [new subgeneric combination by Hardy 1975: 4].
Strigidia uncinata (Ohaus) [new combination by Soula 2006: 31].
Pelidnota (Strigidia) uncinata (Ohaus) [revised combination and revised subgeneric combination by Özdikmen 2009: 145].
Pelidnota uncinata Ohaus [removal of subgeneric classification by Soula 2009: 116].

#### Distribution.

BOLIVIA: Cochabamba, Santa Cruz (Ohaus 1930a, 1934b, Machatschke 1972, Soula 2006, Krajcik 2008). BRAZIL: Amazonas (Ohaus 1930a). ECUADOR: Napo (Ohaus 1930a, 1934b, Paucar-Cabrera 2005). PERU: Amazonas (Ohaus 1930a, Soula 2006, Ratcliffe et al. 2015).

#### Types.

**Figure 93.**
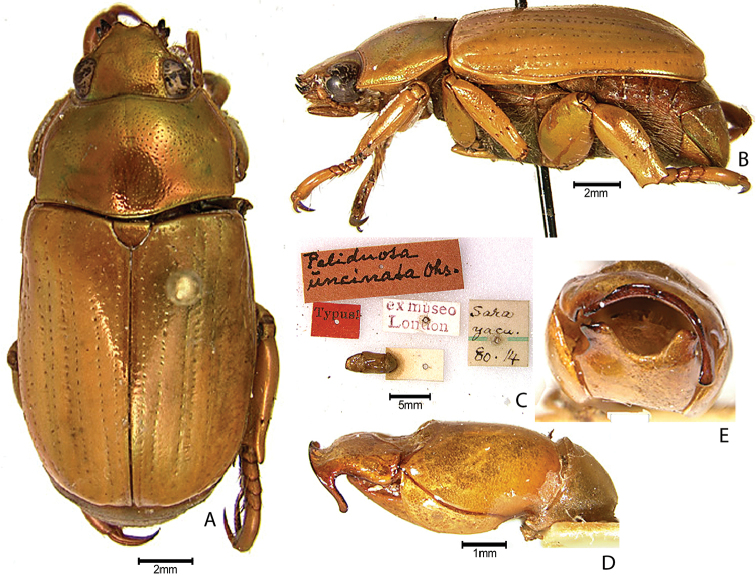
*Pelidnota uncinata* Ohaus type male from ZMHB. **A** Dorsal habitus **B** Lateral habitus **C** Specimen labels and male genitalia **D** Male genitalia, lateral view **E** Male parameres, caudal view.

1 ♂ type specimen of *Pelidnota uncinata* Ohaus at ZMHB (Fig. 93). Soula (2006) also recorded 1 ♂ lectotype, 1 paralectotype, and 1 “paratype” at ZMHB (see “*Type Specimens and Lectotype Designation*” in Methods).

### Pelidnota unicolor bonariensis

Taxon classificationAnimaliaColeopteraScarabaeidae

Burmeister, 1855

Pelidnota bonariensis Burmeister, 1855: 522 [original combination].
Pelidnota unicolor bonariensis Burmeister [new subspecific status by Ohaus 1913: 500].
Pelidnota (Pelidnota) unicolor bonariensis Burmeister [new subgeneric combination by Ohaus 1918: 24].
Pelidnota unicolor bonariensis Burmeister [removal of subgeneric classification by Soula 2009: 93].

#### Distribution.

ARGENTINA: Buenos Aires (Burmeister 1855, Harold 1869b, Ohaus 1918, 1934b, Blackwelder 1944, Machatschke 1972, Soula 2009). URUGUAY (Ohaus 1918, 1934b, Blackwelder 1944, Machatschke 1972, Soula 2009).

### Pelidnota unicolor unicolor

Taxon classificationAnimaliaColeopteraScarabaeidae

(Drury, 1782)

Scarabeus unicolor Drury, 1782: 61 [original combination].
Pelidnota unicolor (Drury) [new combination by Blanchard 1851: 211]
Pelidnota (Pelidnota) unicolor (Drury) [new subgeneric combination by Ohaus 1918: 24]
Pelidnota unicolor (Drury) [removal of subgeneric combination by Soula 2009: 91–93]
Melolontha druryana Herbst, 1790 **synonym.**
Melolontha druryana Herbst, 1790: 163 [original combination].
Pelidnota unicolor (Drury) [syn. by Blanchard 1851: 211].
Pelidnota testacea Laporte, 1840 **synonym.**
Pelidnota testacea Laporte, 1840: 122 [original combination].
Pelidnota druryana (Drury) [syn. by Burmeister 1844: 403].

#### Distribution.

BRAZIL: Espírito Santo, Minas Gerais, Pernambuco, Rio de Janeiro, São Paulo, Santa Catarina (Herbst 1790; Laporte 1840; Burmeister 1844; Blanchard 1851; Harold 1869b; Ohaus 1908a, 1913, 1918, 1934b; Guimarães 1944; Machatschke 1972; Krajcik 2008; Soula 2009). PERU (Ratcliffe et al. 2015).

#### Types.

The following specimen is at CCECL. 1 invalid ♂ neotype: “Salesopolis São Paulo Brésil M. SOULA det 19 [obverse] II/2000//*Pelidnota unicolor* (Dr.) M. SOULA det 2008//Néotype 2008 *Scarabeus unicolor* Dr Soula det.” (47030861). Genitalia card-mounted underneath the invalid neotype. Box 4618683 SOULA.

#### Remarks.

Soula (2009: 92) designated a neotype specimen for *Pelidnota unicolor unicolor*. Soula (2009) did not state where this neotype was deposited. Article 75.3.7 (ICZN 1999) requires a statement that the “neotype is, or immediately upon publication has become, the property of a recognized scientific or educational institution, cited by name, that maintains a research collection, with proper facilities for preserving name-bearing types, and that makes them accessible for study”. We recovered Soula’s invalid neotype specimen in his formerly private collection (now at CCECL). Soula´s (2009) neotype is invalid because Soula’s collection was private and Soula (2009) did not make a statement of neotype deposition.

**Figure 94.**
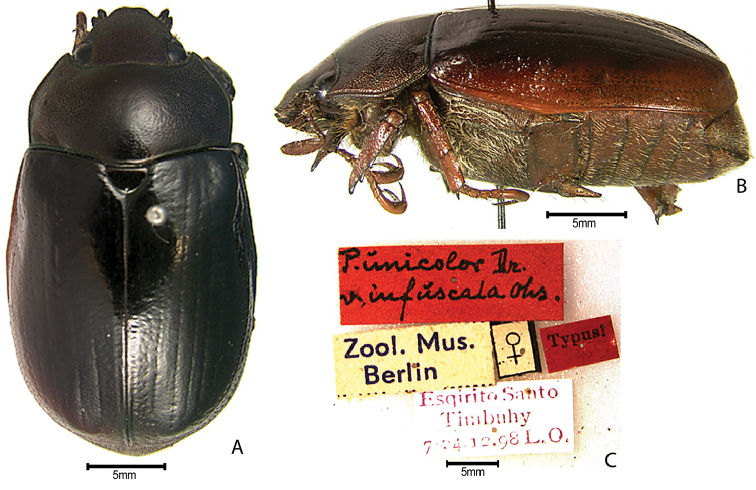
Pelidnota unicolor var. infuscata Ohaus (unavailable name) (valid name *Pelidnota unicolor* [Drury]) invalid type female from ZMHB. **A** Dorsal habitus **B** Lateral habitus **C** Specimen labels.

We treat Pelidnota unicolor var. infuscata Ohaus (Fig. 94) as unambiguously infrasubspecific and thus as an **unavailable name**. Ohaus (1913) clearly described this taxon as a variety. For the purpose of Art. 45.6.4. ICZN, Ohaus’s (1913) publication described both subspecies and varieties (sometimes both for the same species, e.g., *Homonyx chalceus*), thus unambiguously allowing us to treat this name in an infrasubspecific manner. Ohaus (1913) clearly and unambiguously described “var. infuscata” as an infrasubspecific name under *P. unicolor*. As such, this name is not treated as a valid species group name. Ohaus (1908) made reference to this dark variety and subsequently named it as a variety (*infuscata*) in 1913. The specimen in ZMHB labeled as a type is, in fact, a dark color morph of *P. unicolor* based on comparisons with the nominotypical form. This specimen was labeled as an invalid type in ZMHB.

### Pelidnota ustarani

Taxon classificationAnimaliaColeopteraScarabaeidae

(Martínez, 1967)

Heteropelidnota ustarani Martínez, 1967: 147–152 [original combination].
Pelidnota ustarani (Martínez) [new combination by Soula 2008: 15].

#### Distribution.

BRAZIL: Espírito Santo (Martínez 1967, Krajcik 2008, Soula 2008).

#### Types.

**Figure 95.**
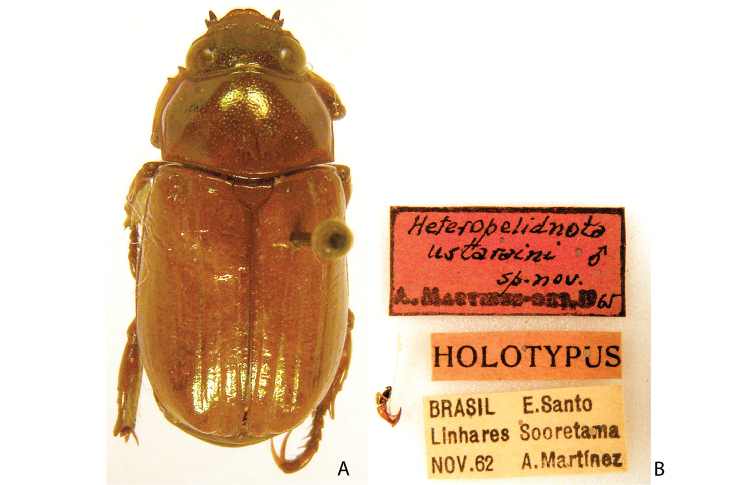
*Heteropelidnota ustarani* Martínez (valid name *Pelidnota ustarani* [Martínez]) holotype male from MACN. **A** Dorsal habitus **B** Specimen labels and male genitalia.

1 ♂ holotype of *Heteropelidnota ustarani* at MACN (Fig. 95). The following specimen is deposited at CMNC. 1 ♀ allotype: BRASIL Est. E. Santo Mun. Linhares P.N. Sooretama Coll. Martínez Nov.-962//H. & A. HOWDEN COLLECTION *ex.* A. Martinez coll.//ALLOTYPE//Heteropelidnota ustarani ♀ sp. nov A. MARTINEZ-DET.1966//[barcode matrix] Canadian Museum of Musée canadien de la NATURE CMNEN 00011914”.

### Pelidnota vanderberghi

Taxon classificationAnimaliaColeopteraScarabaeidae

Soula, 2010

Pelidnota vanderberghi Soula, 2010a: 39-40 [original combination].

#### Distribution.

BOLIVIA: La Paz (Soula 2010a).

#### Types.

The following specimens are at CCECL. 1 ♂ holotype, 1 ♀ allotype, 18 ♂ paratypes, 9 ♀ paratypes: “Région des Yungas Bolivie//Holotype 2010 *Pelidnota vanderberghi* S. Soula” (47030189); “COLL. LECOURT G. INCA-HUARA, 1450 m. NOR-YUNGAS XI.1995. BOLIVIE//Allotype 2010 *Pelidnota vanderberghi* S. Soula” (47030190); seven paratypes with identical labels “Région des Yungas Bolivie//Paratype 2010 *Pelidnota vanderberghi* Soula” (47030191 to 47030194, exch10 to exch12); two paratypes with identical labels “Nord-Yungas, 1500-1800m Bolivie//Paratype 2010 *Pelidnota vanderberghi* S. Soula” (47030195 and 47030196); three paratypes with identical labels “Yungas Bolivie M. SOULA det. 20 [obverse] XI/2010//Paratype 2010 *Pelidnota vanderberghi* S. Soula” (47030197 to 47030199); “Yungas (Bo) 520 m coll. – SOULA//Paratype 2010 *Pelidnota vanderberghi* Soula” (47030200); “Caranavi [arrow] Tocumo [pro Yucumo ?] (850 m) coll. – SOULA//Paratype 2010 *Pelidnota vanderberghi* Soula” (47030201); “Appolo [arrow] Guanay (Bo.) coll. – SOULA [obverse] (Bol.)//Paratype 2010 *Pelidnota vanderberghi* Soula” (47030202); “N. Yungas Bolivie (en 90)//Paratype 2010 *Pelidnota vanderberghi* Soula” (47030203); “Route de Coroico à Coranavi [pro Caranavi] (Bolivie)//Paratype 2010 *Pelidnota vanderberghi* Soula” (47030204); “Inca Huara 1450 m. 11/94 coll. – SOULA//Paratype 2010 *Pelidnota vanderberghi* Soula” (47030205); two paratypes with identical labels “Inca Huara 1450 m (Bo.) 11/94 coll. – SOULA//Paratype 2010 *Pelidnota vanderberghi* Soula” (47030206 and 47030207); three paratypes with identical labels “Inca Huara 1400 m (Bo.) coll. – SOULA//Paratype 2010 *Pelidnota vanderberghi* Soula” (47030208 to 47030210); “Inca-Huara (1450m) N. Yungas-Bolivie XI/95-Lecourt leg.//Paratype 2010 *Pelidnota vanderberghi* Soula” (47030211); “Col G. LECOURT Chappare km 95 1900 m/[the date 08.1984 is crossed out] BOLIVIE [obverse] 10.88//Paratype 2010 *Pelidnota vanderberghi* Soula” (47030212); “BOLIVIE - CARANAVI NOR YUNGAS - ALT.900m Du 15 AU 30/11/89 COLLECTION LECOURT//Paratype 2010 *Pelidnota vanderberghi* Soula” (47030213); “Caranavi 1000m Nor Yungas BOLIVIA 1.90 [fade] coll. M. Büche//Paratype 2010 *Pelidnota vanderberghi* Soula” (47030214). Genitalia card-mounted underneath the holotype and 16 paratypes. Box 4618656 SOULA.

### Pelidnota vazdemelloi

Taxon classificationAnimaliaColeopteraScarabaeidae

(Soula, 2006)

Strigidia vazdemelloi Soula, 2006: 12, 55 [original combination].
Pelidnota vazdemelloi (Soula) [new combination by Soula 2009: 116].

#### Distribution.

BRAZIL: Mato Grosso, Mato Grosso do Sul (Soula 2006, Garcia et al. 2013, Oliveira et al. 2016).

#### Types.

The following specimens are at CCECL. 1 ♂ holotype, 1 ♀ allotype: “Mato Grosso, Brasil leg Alvarenga, XI 63 [crossed out]//Holotype 2006 *Strigidia vazdemelloi* Sou. Soula” (47030428); “Sinop//Allotype 2006 *Strigidia vazdemelloi* Sou. Soula” (47030429). Genitalia are card-mounted underneath the male holotype. Box 4618663 SOULA.

#### Remarks.

Soula (2006) compared this species with *P. discicollis* and the image that accompanies the description looks remarkably similar to other specimens of *P. discicollis*.

### Pelidnota villavicencioensis

Taxon classificationAnimaliaColeopteraScarabaeidae

Soula, 2010

Pelidnota villavicencioensis Soula, 2010a: 61 [original combination].

#### Distribution.

COLOMBIA: Meta (Soula 2010a).

#### Types.

The following specimen is deposited at CCECL. 1 ♂ holotype: “Colombie coll. – SOULA [obverse] Villavicencio//Holotype 2010 *Pelidnota villaviciencoensis* (*sic*) S. Soula” (47030495). Genitalia card-mounted underneath the male holotype. Box 4618666 SOULA.

### Pelidnota virescens

Taxon classificationAnimaliaColeopteraScarabaeidae

Burmeister, 1844

Pelidnota virescens Burmeister, 1844: 403 [original combination].
Pelidnota (Pelidnota) virescens Burmeister [new subgeneric combination by Ohaus 1918: 24].
Pelidnota virescens Burmeister [removal of subgeneric classification by Soula 2009: 60–61].
Pelidnota (Pelidnotidia) permicans Casey, 1915 **synonym.**
Pelidnota (Pelidnotidia) permicans Casey, 1915: 77 [original combination].
Pelidnota (Pelidnota) permicans Casey [new subgeneric combination by Ohaus 1934b: 80].
Pelidnota (Pelidnota) virescens Burmeister [syn. by Hardy 1975: 22].
Pelidnota (Pelidnota) virescens planipennis Ohaus, 1918 **synonym.**
Pelidnota (Pelidnota) virescens var. planipennis Ohaus, 1918: 24 [original combination].
Pelidnota (Pelidnota) virescens planipennis Ohaus [new subspecific status by Machatschke 1972: 24].
Pelidnota permicans Casey [syn. by Soula 2009: 61–62].

#### Distribution.

COSTA RICA: San José (Hardy 1975, Soula 2009, Deloya et al. 2014). HONDURAS (Hardy 1975, Soula 2009, Deloya et al. 2014). MEXICO: Baja California Sur, Chiapas, Colima, Distrito Federal, Durango, Guerrero, Jalisco, México, Michoacán, Morelos, Nayarit, Oaxaca, Puebla, Sinaloa, Sonora, Veracruz (Burmeister 1844, Blanchard 1851, H. W. Bates 1888, Casey 1915, Ohaus 1918, 1934b, Blackwelder 1944, Gibson and Carrillo 1959, Carrillo et al. 1966, Machatschke 1972, Hardy 1975, Maes 1987, Deloya et al. 1993, 2014, Morón et al. 1988, 1997, 1998, Thomas 1993, Rodríguez-Palafox and Corona 2002, Morón and Deloya 2002, Pacheco Flores at al. 2006, Krajcik 2008, Soula 2009, García et al. 2009, Yanes-Gómez and Morón 2010, 2013, Aragón-García et al. 2012, Pérez-Torres et al. 2013, Cuate et al. 2013, Lugo et al. 2012, 2013, 2014, Castañeda-Osorio et al. 2015). NICARAGUA: Managua (Maes 1987).

#### Types.

1 ♂ neotype of *Pelidnota virescens* at MNHN (Soula 2009). 1 ♂ lectotype of Pelidnota (Pelidnota) virescens var. planipennis at BMNH (Soula 2009); The following specimens of Pelidnota (Pelidnota) virescens var. planipennis are deposited at CCECL. 1 ♂ paralectotype, 1 ♀ paralectotype: “Acapulco, Guerrero. Höge.//*Pelidnota planipennis* Oh. M. SOULA det 2008//Paralectotype 2008 *Pelidnota virescens* v. *planipennis* Oh. Soula det.” (47030488); “Acapulco, Guerrero. Höge.//H.W.Bates Biol.Cent.Amer.//2008 *Pelidnota planipennis* Ohaus M. SOULA det 19//Paralectotype 2008 Pelidnota virescens var. planipennis Bates Soula det.” (47030489). The paralectotypes were apparently retained from the MNHN type series. Box 4618666 SOULA.

#### Remarks.

Soula’s (2009) language that indicated the synonymy of these species is confusing. Soula (2009) listed *P. virescens* Burmeister and *P. planipennis* Ohaus as valid species. However, in his summary of synonyms he listed *P. planipennis* as a new synonym of *P. permicans*, while simultaneously treating *P. permicans* as a synonym of *P. virescens* (Soula 2009: 62). This is in agreement with Hardy (1975), who studied these species and determined that the parameres of *P. virescens* vary along a north-south cline. We follow Hardy (1975), and possibly Soula (2009), and list P. (Pelidnotidia) permicans Casey and P. (Pelidnota) virescens var. planipennis Ohaus as synonyms of *P. virescens* Burmeister.

### Pelidnota viridicuprea

Taxon classificationAnimaliaColeopteraScarabaeidae

Ohaus, 1908

Pelidnota viridicuprea Ohaus, 1908b: 401–402 [original combination].
Pelidnota (Chalcoplethis) viridicuprea Ohaus [new subgeneric combination by Ohaus 1918: 29].
Strigidia viridicuprea (Ohaus) [new combination by Soula 2006: 73–74].
Pelidnota viridicuprea Ohaus [revised combination by Soula 2009: 116].

#### Distribution.

ECUADOR: Napo, Pastaza (Ohaus 1908b, 1918, 1934b, Machatschke 1972, Paucar-Cabrera 2005, Soula 2006, Krajcik 2008).

#### Types.

**Figure 96.**
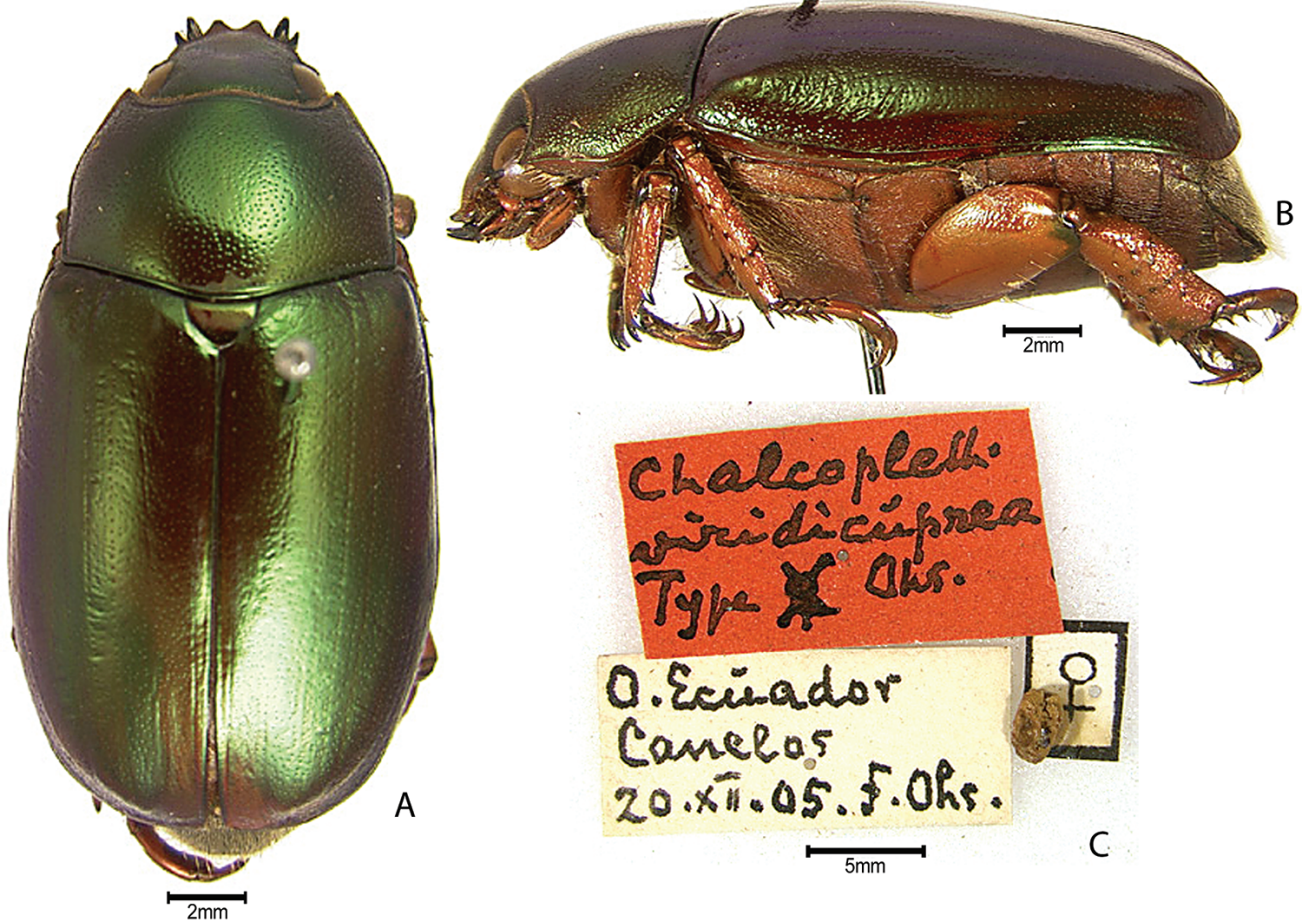
*Pelidnota viridicuprea* Ohaus holotype female from ZMHB. **A** Dorsal habitus **B** Lateral habitus **C** Specimen labels and egg.

1 holotype ♀ of *Pelidnota viridicuprea* at ZMHB (Fig. 96).

### Pelidnota vitalisi

Taxon classificationAnimaliaColeopteraScarabaeidae

Ohaus, 1925

Pelidnota (Ganonota) vitalisi Ohaus, 1925: 77–78 [original combination].
Pelidnota (Strigidia) vitalisi Ohaus [new subgeneric combination by Machatschke 1970: 157].
Pelidnota (Odontognathus) vitalisi Ohaus [new subgeneric combination by Hardy 1975: 4].
Pelidnota (Ganonota) vitalisi Ohaus [revised subgeneric combination by Frey 1976: 344].
Strigidia vitalisi (Ohaus) [new combination by Soula 2006: 16].
Pelidnota (Strigidia) vitalisi Ohaus [revised combination and revised subgeneric combination by Özdikmen 2009: 145].
Pelidnota vitalisi Ohaus [removal of subgeneric classification by Soula 2009: 116].

#### Distribution.

BRAZIL: Mato Grosso (Ohaus 1925, 1934b, Machatschke 1972, Soula 2006, Krajcik 2008).

#### Types.

**Figure 97.**
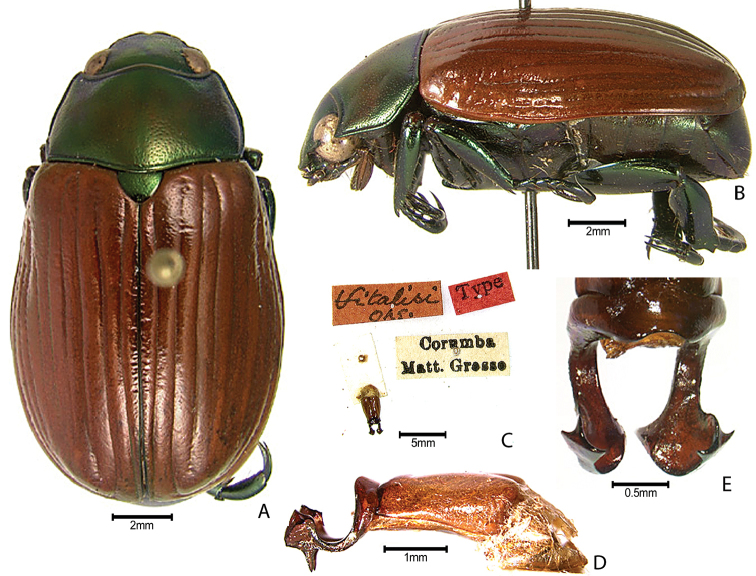
Pelidnota (Ganonota) vitalisi Ohaus (valid name *Pelidnota vitalisi* Ohaus) type male (see “*Type specimens and lectotype designation*” in Methods) from ZMHB. **A** Dorsal habitus **B** Lateral habitus **C** Specimen labels and male genitalia **D** Male genitalia, lateral view **E** Male parameres, dorsal view.

Lectotype of Pelidnota (Ganonota) vitalisi in ZMHB and an unknown number of paralectotypes should be at MNHN, but were not recorded in Soula (Soula 2006). Soula (2006) mentioned a female paralectotype at IRSNB. Lectotype ♂ at ZMHB with labels: a) “Corumba Matt. Grosso” (typeset, white label), b) male genitalia card mounted, c) “Type” (red label, typeset), d) “Vitalisi Ohs” (red label, handwritten but not in Ohaus’ handwriting) (Fig. 97).

#### Remarks.

Ohaus (1925) compared *P. vitalisi* with P. (Odontognathus) cuprea and *P. rubripennis* Burmeister. He placed the species in Pelidnota (Ganonota). Based on the original description, Ohaus (1925) had at least one female and one male specimen from Corumba, Mato Grosso, Brazil. Ohaus (1925: fig. 1, p. 78) provided an illustration of the male parameres in dorsal and lateral views. The species is named for Mr. R. Vitalis de Salvaza, to whom some type specimens were donated. Vitalis de Salvaza’s collection eventually went to Le Moult’s collection and then to MNHN. With the exception of the male genitalia, this species is not easily distinguished from others in the *P. cuprea*-complex. The head, pronotum, scutellum, pygidium, and venter are metallic green; elytra are deeply striated and tan. However, the color alone is not sufficient to identify the species.

### Pelidnota vitticollis

Taxon classificationAnimaliaColeopteraScarabaeidae

Burmeister, 1844

Pelidnota vitticollis Burmeister, 1844: 396 [original combination].
Pelidnota bivittata (Swederus) [syn. by F. Bates 1904: 257].
Pelidnota vitticollis Burmeister [revised species status by Ohaus 1913: 504–506].
Pelidnota (Ganonota) vitticollis Burmeister [new subgeneric combination by Ohaus 1918: 28].
Pelidnota (Strigidia) vitticollis Burmeister [new subgeneric combination by Machatschke 1970: 157].
Pelidnota (Odontognathus) vitticollis Burmeister [new subgeneric combination by Hardy 1975: 4].
Strigidia vitticollis (Burmeister) [new combination by Soula 2006: 43–44].
Pelidnota (Strigidia) vitticollis Burmeister [revised combination and revised subgeneric combination by Özdikmen 2009: 145].
Pelidnota vitticollis Burmeister [removal of subgeneric classification by Soula 2009: 116].

#### Distribution.

BRAZIL: Espírito Santo, Rio de Janeiro, Santa Catarina (Burmeister 1844, Ohaus 1918, 1934b, Machatschke 1972, Soula 2006, Krajcik 2008).

#### Types.

Types of *Pelidnota vitticollis* at MHNN (Soula 2006).

### Pelidnota werneri

Taxon classificationAnimaliaColeopteraScarabaeidae

(Soula, 2006)

Strigidia werneri Soula, 2006: 10, 85 [original combination].
Pelidnota werneri (Soula, 2006) [new combination by Soula 2009: 116].

#### Distribution.

PERU: Loreto (Soula 2006, Ratcliffe et al. 2015).

#### Types.

The following specimens are deposited at CCECL. 1 ♂ holotype, 1 ♀ allotype, 1 ♀ paratype: “Iquitos, Loreto Pérou; XI/2003//Holotype 2006 *Strigidia werneri* S. Soula” (47030129); “Iquitos, Loreto Pérou; XI/2003//Allotype *Strigidia werneri* S. 2006 Soula” (47030130); “Iquitos, Loreto Pérou; VIII/2003//Paratype 2006 *Strigidia werneri* S. Soula” (47030131). Genitalia card-mounted underneath holotype. Box 4618654 SOULA.

### Pelidnota xanthopyga

Taxon classificationAnimaliaColeopteraScarabaeidae

Hardy, 1975

Pelidnota (Odontognathus) xanthopyga Hardy, 1975: 6, 12 [original combination].
Strigidia xanthopyga (Hardy) [new combination by Soula 2006: 56].
Pelidnota (Strigidia) xanthopyga Hardy [revised combination and new subgeneric combination by Özdikmen 2009: 145].
Pelidnota xanthospyga (*sic*) Hardy [removal of subgeneric classification by Soula 2009: 116].

#### Distribution.

COLOMBIA: Santander (López-García et al. 2015). HONDURAS (Hardy 1975, Soula 2006). PANAMA: Chiriquí (Hardy 1975, Ratcliffe 2002, Soula 2006, Krajcik 2008, López-García et al. 2015).

#### Types.

1 ♂ holotype and 1 ♀ allotype of Pelidnota (Odontognathus) xanthopyga at USNM (Hardy 1975); 1 paratype at NHMB (Hardy 1975).

### Pelidnota xanthospila

Taxon classificationAnimaliaColeopteraScarabaeidae

(Germar, 1824)

Rutela xanthospila Germar, 1824: 119 [original combination].
Pelidnota xanthospila (Germar) [new combination by Burmeister 1844: 393–394].
Pelidnota (Ganonota) xanthospila (Germar) [new subgeneric combination by Ohaus 1918: 26].
Pelidnota (Strigidia) xanthospila (Germar) [new subgeneric combination by Machatschke 1970: 157].
Pelidnota (Odontognathus) xanthospila (Germar) [new subgeneric combination by Hardy 1975: 4].
Strigidia xanthospila (Germar) [new combination by Soula 2006: 26–27].
Pelidnota (Strigidia) xanthospila (Germar) [revised combination and revised subgeneric combination by Özdikmen 2009: 145].
Pelidnota xanthospila (Germar) [removal of subgeneric classification by Soula 2009: 116].
Rutela ornata Perty, 1830 **synonym.**
Rutela ornata Perty, 1830: 49 [original combination].
Pelidnota xanthospila (Germar) [syn. by Ohaus 1918: 26].
Rutela rubiginosa Laporte, 1840 **synonym.**
Rutela rubiginosa Laporte, 1840: 120 [original combination].
Pelidnota xanthospila var. rubiginosa (Laporte) [new combination and new infrasubspecific status by Ohaus 1918: 26].
Pelidnota xanthospila forma rubiginosa (Laporte) [revised infrasubspecific status by Machatschke 1972: 28].
Pelidnota xanthospila (Germar) [syn. by Krajcik 2008: 98].

#### Distribution.

BRAZIL: Bahia, Espírito Santo, Minas Gerais, Santa Catarina, São Paulo, Rio de Janeiro (Laporte 1840, Burmeister 1844, Blanchard 1851, Ohaus 1918, 1934b, Machatschke 1972, Soula 2006, Krajcik 2008).

#### Remarks.

This species has a great deal of color variation and variation in elytral maculae (broad, yellow to narrow and confined to near the base). The species is distributed in the Brazilian coastal states.

### Pelidnota yungasensis

Taxon classificationAnimaliaColeopteraScarabaeidae

Soula, 2009

Pelidnota yungasensis Soula, 2009: 32, 89–90 [original combination].

#### Distribution.

BOLIVIA: La Paz (Soula 2009).

#### Types.

The following specimens are deposited at CCECL. 1 ♂ holotype, 1 ♀ allotype, 9 ♂ paratypes: “N. Yungas (Bo.) coll. – SOULA//Holotype 2008 *Pelidnota fulva yungasensis* S. Soula//*Pelidnota yungasensis* Soula det. MR Moore ‘15” (47030615); “N. Yungas (Bo.) coll. – SOULA//Allotype 2008 *Pelidnota fulva yungasensis* S. Soula//*Pelidnota yungasensis* Soula det. MR Moore ‘15” (47030616); nine paratypes with identical label data: “N. Yungas (Bo.) coll. – SOULA//Paratype 2008 *Pelidnota fulva yungasensis* S. Soula//*Pelidnota yungasensis* Soula det. MR Moore ‘15” (47030617 to 47030624, exch35). Genitalia card-mounted underneath the male holotype and eight male paratypes. Box 4618675 SOULA.

#### Remarks.

Soula’s (2009) original description referred to “*P. yungasensis* n. ssp.”. In the index, key to species, and accompanying figures, *P. yungasensis* was treated as a species. We treat the “spp. n.” as a lapsus and consider *P. yungasensis* to have been proposed as a species (Moore and Jameson 2013).

### Pelidnota zovii

Taxon classificationAnimaliaColeopteraScarabaeidae

Soula, 2010

Pelidnota zovii Soula, 2010a: 39 [original combination].

#### Distribution.

PERU: Huánuco, Junín (Soula 2010a, Ratcliffe et al. 2015).

#### Types.

Soula (2010a) indicated that the holotype ♂ should be at CCECL, but we did not find it there.

## *Pelidnota names nomen dubium*

### Pelidnota aeruginosa

Taxon classificationAnimaliaColeopteraScarabaeidae

(Linnaeus, 1767) nomen dubium

Scarabaeus aeruginosus Linnaeus, 1767: 558 [original combination].
Pelidnota aeruginosa (Linnaeus) [new combination by Hope 1837: 17].

#### Remarks.

This name is widely used in collections and the literature, but the identity of the species is uncertain (F. Bates 1904, Krajcik 2008, Soula 2009, Krell et al. 2012, Moore and Jameson 2013). It is likely that the complication originated as a misidentification by Drury (1773). The problem is further complicated by the homonym *Pelidnota aeruginosa* Sturm, 1843 (=*Chrysina peruviana* Kirby, 1828 [1827]) (Hawks 2001a) and the name *Pelidnota aeruginosa var. citripennis* Ohaus (=*Pelidnota semiaurata citripennis* Ohaus) (see Moore and Jameson 2013). The name *Pelidnota aeruginosa* is currently considered a *nomen dubium* (uncertain name; see discussion by Krell et al. 2012).

## *Pelidnota* species *incertae sedis*

### Pelidnota emerita

Taxon classificationAnimaliaColeopteraScarabaeidae

(Olivier, 1789) incertae sedis

Cetonia emerita Olivier, 1789: 71 [original combination].
Rutela emerita (Olivier) [new combination by Schönherr 1817: 152].
Pelidnota emerita (Olivier) [new combination by Burmeister 1844: 409].

#### Distribution.

SOUTH AMERICA (Olivier 1789, Schönherr 1817, Burmeister 1844).

#### Remarks.

*Cetonia emerita* Olivier was described based on a specimen from “Amérique méridionale” (Olivier 1789). Olivier (1789) stated that his new species was slightly larger than *Cetonia chrysis* (=*Macraspis chrysis* [Fabricius]). The description indicates that the type specimen is hairless, coppery-green dorsally, and green ventrally (Olivier 1789). The elytra have obvious striae and the sternum (=mesosternal process) is projected forward and pointed (Olivier 1789). The tibiae are tridentate (Olivier 1789). Schönherr (1817) transferred the species into *Rutela*. Burmeister (1844) did not see the type specimen but transferred the species into *Pelidnota* based on the description. *Pelidnota emerita* (Olivier) was not mentioned in the literature again until the catalogs of world Rutelinae where it was listed as *incertae sedis* (Ohaus 1918, 1934b, Machatschke 1972). We have not examined the type specimen of this species and the validity of this taxon is unknown to us.

### Pelidnota fallax

Taxon classificationAnimaliaColeopteraScarabaeidae

Gistel, 1857 incertae sedis

Pelidnota fallax Gistel, 1857: 80 [original combination].

#### Distribution.

BRAZIL (Gistel 1857, Blackwelder 1944, Krajcik 2008, Soula 2009).

#### Remarks.

Gistel (1857) provided a very brief Latin description of this species which he compared to *P. glauca*. *Pelidnota fallax* was described as being brass-green in color (Gistel 1857). The pronotum is “glittering copper-green” with yellow edges (Gistel 1857). This species was not included in catalogs of world Rutelinae (Ohaus 1918, 1934b, Machatschke 1972, 1974). Krajcik (2008) correctly listed *P. fallax* as a valid species. We have not examined the type specimen of this species and the validity of this taxon is unknown to us. Because *P. fallax* was compared to *P. glauca* (synonym of *P. alliacea* [Germar]) by Gistel (1857), examination of the type specimen (possibly lost) of *P. fallax* Gistel could be important for stabilizing the taxonomy and nomenclature of species previously compared to *P. aeruginosa* (Linnaeus).

### Pelidnota sybarita

Taxon classificationAnimaliaColeopteraScarabaeidae

Harold, 1869 incertae sedis

Pelidnota sumptuosa Laporte, 1840: 123 [original combination, junior homonym of *Pelidnota sumptuosa* (Vigors, 1825)].
Pelidnota sybarita Harold, 1869a: 124 [original combination, new replacement name for *Pelidnota sumptuosa* Laporte].
Pelidnota luxuriosa Blackwelder, 1944 **synonym.**
Pelidnota luxuriosa Blackwelder, 1944: 237 [original combination, new replacement name for *Pelidnota sumptuosa* Laporte].
Pelidnota sumptuosa (Vigors) [syn. by Machatschke 1970: 158].
Pelidnota (Pelidnota) luxuriosa Blackwelder [new subgeneric combination and revised species status by Machatschke 1972: 25].
Pelidnota luxuriosa Blackwelder [removal of subgeneric classification by Soula 2009: 43].
Pelidnota sybarita Harold [**objective synonymy**].

#### Distribution.

BRAZIL (Laporte 1840, Harold 1869b; Blackwelder 1944, Soula 2009).

#### Remarks.

Laporte (1840) described *Pelidnota sumptuosa* from Brazil. Laporte’s (1840) specimen is coppery-green with bronze reflections. The antennae are brownish-red and the elytra are “large”, smooth, and have three weak striae (Laporte 1840). The venter is setose, the protibiae are tridentate, and the tarsi are “thickened” (Laporte 1840). The name *Pelidnota sumptuosa* Laporte is a junior homonym of *Pelidnota sumptuosa* (Vigors). Harold (1869a) detected this case of homonymy and replaced the preoccupied name *Pelidnota sumptuosa* Laporte with *Pelidnota sybarita*. Subsequent authors (e.g., Ohaus) ignored the case of homonymy and Harold’s (1869a) proposed replacement name *Pelidnota sybarita*. Blackwelder (1944), probably unaware of Harold’s (1869a) replacement name, proposed the name *Pelidnota luxuriosa* as a replacement for *Pelidnota sumptuosa* Laporte. Because Harold’s (1869a) name has nomenclatural priority and these names are based on the same type specimen, we consider *Pelidnota luxuriosa* Blackwelder an **objective synonym** of *Pelidnota sybarita* Harold.

Machatschke (1970, 1972) continued the use of *Pelidnota luxuriosa* as the valid name for this species in his discussions and catalogs. Machatschke (1970) discussed this case homonymy and concluded that *P. luxuriosa* Blackwelder and *P. sumptuosa* Vigors were synonyms and that *P. sumptuosa* Vigors had nomenclature priority. However, Machatschke’s (1972) catalog did not reflect these proposed changes. Instead, *P. sumptuosa* Vigors was listed as a junior synonym of *P. ludovici* Ohaus, while *P. luxuriosa* was considered a valid name (Machatschke 1972). Soula (2009) revalidated *P. sumptuosa* Vigors and discussed Laporte’s (1840) description of *P. sumptuosa* (=*P. sybarita*), but he was unable to find the type specimen. Soula (2009) did not recognize Harold’s (1869a) replacement name, *P. sybarita*. We list *P. sybarita* as *incertae sedis* until the type specimen of Laporte (1840) is discovered and the identity of this species can be evaluated.

*Pelidnota luxuriosa* Blackwelder is the name for this species in prevailing usage and has been cited as such since Blackwelder (1944). However, reversal of precedence is not possible under ICZN Article 23.9.1 which states, “prevailing usage must be maintained when the following conditions are both met: (23.9.1.1.) the senior synonym or homonym has not been used as a valid name after 1899, and (23.9.1.2.) the junior synonym or homonym has been used for a particular taxon, as its presumed valid name, in at least 25 works, published by at least 10 authors in the immediately preceding 50 years and encompassing a span of not less than 10 years”. In this case, Article 23.9.1.1 is satisfied but Article 23.9.1.2 is not satisfied, because the name *P. luxuriosa* Blackwelder has only appeared in four publications (Blackwelder 1944, Machatschke 1970, 1972, Soula 2009). We do not think it is desirable to suppress the name *P. sybarita* Harold under Article 23.9.3 because the type specimen for this species is apparently lost and the species is so poorly known.

### Pelidnota versicolor

Taxon classificationAnimaliaColeopteraScarabaeidae

(Billberg, 1820) incertae sedis

Rutela versicolor Billberg, 1820: 384 [original combination].
Pelidnota versicolor (Billberg) [new combination by Burmeister 1844: 409].

#### Distribution.

BRAZIL (Billberg 1820, Burmeister 1844, Blackwelder 1944).

#### Remarks.

Billberg (1820) described *Rutela versicolor* from Brazil. Burmeister (1844) included the species in *Pelidnota* based on the description, though he mentioned that he had not seen specimens of *Rutela versicolor*. Ohaus (1918) listed *Pelidnota versicolor* (Billberg) as *incertae sedis* and speculated that it was the female of *Rhinaspis aenea* (Billberg). Subsequent catalogs maintained the species as *incertae sedis* (Ohaus 1934b, Machatschke 1972). The validity of this species is unknown to us and we list it here as being *incertae sedis*.

### Rutela caesarea

Taxon classificationAnimaliaColeopteraScarabaeidae

Gistel, 1857 incertae sedis

Rutela caesarea Gistel, 1857: 29 [original combination].

#### Distribution.

COLOMBIA (Gistel 1857, Blackwelder 1944, Krajcik 2008, Soula 2009).

#### Remarks.

Gistel (1857) provided only a very short Latin description of this species and some brief notes in German. He described *R. caesarea* having a polished thorax with fine punctation and elevated striae on the elytra (Gistel 1857). The tarsi are bluish-green and the specimen has yellow eyes (Gistel 1857). In German, Gistel (1857) stated that *R. caesarea* is of similar size to *Pelidnota semiaurata* Burmeister, but “thinner”. The specimen is also described as having shiny, golden-green reflections (Gistel 1857). The name *R. caesarea* Gistel did not appear in catalogs of world Rutelinae (Ohaus 1918, 1934b, Machatschke 1972, 1974). Blackwelder (1944) was aware of the name and listed it under *Rutela* in his catalog. Krajcik (2008) listed *R. caesarea* as a probable synonym of *Pelidnota aeruginosa* (Linnaeus), a *nomen dubium*, probably based on the original description’s comparison to *P. semiaurata*. We think that there is no basis for listing *R. caesarea* in synonymy at this time because its type specimen, and thus the validity of the species, is unknown to us. *Rutela caesarea* is listed here as *incertae sedis* until the type specimen can be found and examined to establish the validity of the species. Gistel’s collection could be deposited at the Bavarian State Collection of Zoology (Munich, Germany), though Gistel’s specimens were accessioned into the collection without appropriate type labels (Schülke 2004, Jelínek and Audisio 2009). Gistel’s ruteline types will likely be very difficult to locate and identify.

### Rutela runica

Taxon classificationAnimaliaColeopteraScarabaeidae

Gistel, 1850 incertae sedis

Rutela runica Gistel, 1850: 381 [original combination].

#### Distribution.

FRENCH GUIANA: Cayenne (Gistel 1850, Krajcik 2008).

#### Remarks.

Gistel (1850) provided only a short description of this species in German. He described *R. runica* being a quarter-inch long and having a chocolate-brown sternum, pro- and mesotarsomeres, and metafemora (Gistel 1850). *Rutela runica* has multiple abdominal spots with the head and prothorax having a stripe that runs through them (Gistel 1850). The scutellum and rune-like markings on the elytra are straw-yellow (Gistel 1850). There are brown dots on either side of the pronotum (Gistel 1850). Catalogs of world Rutelinae omitted *R. runica* (Ohaus 1918, 1934b, Machatschke 1972, 1974). Krajcik (2008), without explanation, listed *R. runica* as a probable synonym of *Pelidnota terminata* Laporte. We think that there is no basis for listing *R. runica* in synonymy at this time because its type specimen, and thus the validity of the species, is unknown to us. From the description, *R. runica* is similar to the following species of *Rutela* that occur in French Guiana: *R. histrio* Sahlberg, *R. lineola* (Linnaeus), and *R. tricolorea* Ohaus. Additionally, there are some *Pelidnota* with similar coloration (e.g., *Pelidnota xanthospila* (Germar) from Brazil) as that described by Gistel (1850) for *R. runica*.

### Rutela tristis

Taxon classificationAnimaliaColeopteraScarabaeidae

Gistel, 1850 incertae sedis

Rutela tristis Gistel, 1850: 381 [original combination].

#### Distribution.

BRAZIL (Gistel 1850, Krajcik 2008).

#### Remarks.

Gistel (1850) provided only a short description of this species in German. He stated that *R. tristis* is narrower than *R. runica*. *Rutela tristis* is black and shiny dorsally with the outer margin of the prothorax yellow. The description indicates that the ventral segments are yellow, in particular the specimen has a yellow sternum and yellow markings on the femora (Gistel 1850). Catalogs of world Rutelinae omitted *R. tristis* (Ohaus 1918, 1934b, Machatschke 1972, 1974). Krajcik (2008), without explanation, listed *R. tristis* as a probable synonym of *Pelidnota terminata* Laporte. We think that there is no basis for listing *R. tristis* in synonymy at this time because its type specimen, and thus the validity of the species, is unknown to us.

## Unavailable, invalid names in *Pelidnota*

### Pelidnota auripes

Taxon classificationAnimaliaColeopteraScarabaeidae

in litt.; Unavailable, invalid name

#### Remarks.

Perty (1830) listed the name *Pelidnota auripes* for figure 8, plate 10. This figure referred to the description of *Pelidnota cupripes* Perty in the text and the misspelling in the figure legend was considered a lapsus (Perty 1830, Ohaus 1918, 1934b, Machatschke 1972).

### Pelidnota demergesi

Taxon classificationAnimaliaColeopteraScarabaeidae

in litt.; Unavailable, invalid name

#### Types.

The following specimens are deposited at CCECL. 1 invalid ♂ holotype, 3 invalid ♂ paratypes: “Tingo Maria Pérou, X/2005//Holotype 2010 *Pelidnota demergesi* S. Soula//Invalid Holotype name in litt. det. M. R. Moore 2014” (47030175); two invalid paratypes with identical label data “Tingo Maria Las cuevas de las pavas M. SOULA det 19 [obverse] 15/IV/2010//Paratype 2010 *Pelidnota demergesi* S. Soula//Invalid Paratype name in litt. det. M. R. Moore 2014” (47030176 and 47030177); “Rio Ucayali Pérou M. SOULA det 19//Paratype 2010 *Pelidnota demergesi* S. Soula//Invalid Paratype name in litt. det. M. R. Moore 2014” (47030178). Genitalia card-mounted underneath the invalid holotype and 2 invalid paratypes. Box 4618656 SOULA.

#### Remarks.

The name *Pelidnota demergesi* has never been associated with a species description or type designation. The name appears only in a figure legend in Soula (2010a) and is currently unavailable (Moore and Jameson 2013).

### Pelidnota desantacatarina

Taxon classificationAnimaliaColeopteraScarabaeidae

 in litt.; Unavailable, invalid name

#### Types.

The following specimens are deposited at CCECL. 1 ♂ invalid holotype, 1 ♀ invalid allotype, 4 ♂ invalid paratypes: “São Bento do Sul. S. C. I/94 M. SOULA det 19//Holotype 2008 *Pelidnota desantacatarina* S. Soula”//Invalid holotype det. Moore ’15” (47030862); “Corupa. S.C. II/1938 M. SOULA det 19//Allotype 2008 *Pelidnota desantacatarina* S. Soula”//Invalid Allotype det. Moore ’15” (47030863); “Santa Catarina II/92 M. SOULA det 19//Paratype 2008 *Pelidnota desantacatarina* S. Soula”//Invalid Paratype det. Moore ’15” (47030864); “Déc. 1963 Corupa, S.C. Brazil//Paratype 2008 *Pelidnota desantacatarina* S. Soula”//Invalid Paratype det. Moore ’15” (47030865); “06.01.1985 Corupa Santa Caterina Brasilien//Paratype 2008 *Pelidnota desantacatarina* S. Soula”//Invalid Paratype det. Moore ’15” (47030866); “*Pelidnota unicolor*//Brésil Hansa. S.C.//Ru 22.//1 1932//Paratype 2008 *Pelidnota desantacatarina* S. Soula”//Invalid Paratype det. Moore ’15” (47030867). Genitalia card-mounted underneath the invalid male holotype and two invalid male paratypes. Box 4618683 SOULA.

#### Remarks.

The name *Pelidnota desantacatarina* Soula does not appear in the literature nor has the name been associated with a species description. These type specimens are considered invalid and the name *Pelidnota desantacatarina* is **unavailable**.

### Pelidnota rectificata

Taxon classificationAnimaliaColeopteraScarabaeidae

 in litt.; Unavailable, invalid name

#### Types.

The following specimen is deposited at CCECL. 1 ♂ invalid holotype: “Faz. Aceiro Jatai, Goiás - Brasil X.1962//Holotype 2008 *Pelidnota rectificata* S. Soula//Invalid Holotype det. MR Moore ‘15” (47030808). Genitalia card-mounted underneath the invalid male holotype. Box 4618681 SOULA. The following specimen is deposited at CMNC. 1 ♀ invalid allotype: “BRASIL GOIAZ Jatai J. Guetin-leg. Coll. Martinez Oct. 953//H. & A. HOWDEN COLLECTION *ex.* A. Martinez coll.//Allotype 2008 *Pelidnota rectificata* S. Soula”.

#### Remarks.

The name *Pelidnota rectificata* Soula does not appear in the literature nor has the name been associated with a species description. This specimen is considered an invalid type and the name *Pelidnota rectificata* is **unavailable**.

### Pelidnota unicolor desantacatarina

Taxon classificationAnimaliaColeopteraScarabaeidae

 in litt.; Unavailable, invalid name

#### Types.

The following specimens are deposited at CCECL. 3 ♂ invalid paratypes: “Santa Catarina Br. I/97 M. SOULA det 19//Paratype 2006 *Pelidnota unicolor desantacatarina* Soula”//Invalid Paratype det. Moore ’15” (47030868); “Bré. Santa Catarina I/97 M. SOULA det 19//Paratype 2006 *Pelidnota unicolor desantacatarina* S. Soula”//Invalid Paratype det. Moore ’15” (47030869); “Santa Catarina Brés. I/97 M. SOULA det 19//Paratype 2006 *Pelidnota unicolor* S. *desantacatarina* Soula”//Invalid Paratype det. Moore ’15” (47030870). Box 4618683 SOULA.

#### Remarks.

The name *Pelidnota unicolor desantacatarina* Soula does not appear in the literature nor has the name been associated with a species description. These type specimens are considered invalid and the name *Pelidnota unicolor desantacatarina* is **unavailable**.

### Pelidnota unicolor occidentalis

Taxon classificationAnimaliaColeopteraScarabaeidae

 in litt.; Unavailable, invalid name

#### Types.

The following specimen is deposited at CCECL. 1 ♂ invalid holotype: “PÉROU CHANCHAMAYO 2000 M//Muséum Paris ex Coll. R. Oberthür 1952//Holotype 2008 *Pelidnota unicolor occidentalis* S. Soula//Invalid Holotype det. MR Moore ‘15” (47030807). Genitalia card-mounted underneath the invalid male holotype. Box 4618681 SOULA.

#### Remarks.

The name *Pelidnota unicolor occidentalis* Soula does not appear in the literature nor has the name been associated with a species description. This specimen is considered an invalid holotype and the name *Pelidnota unicolor occidentalis* is **unavailable**.

## Unavailable names in *Pelidnota* (application of ICZN Article 16.4.1)

The name *Strigidia testaceovirens argentinica* Soula was proposed for a subspecies from Misiones, Argentina (Soula 2006). ICZN Article 16.4.1 states that new specific and subspecific names published after 1999 must be accompanied in the original publication “by the explicit fixation of a holotype, or syntypes, for the nominal taxon”. There is no mention of a holotype specimen of *Pelidnota testaceovirens argentinica* in Soula (2006), but paratypes are mentioned. Per ICZN Article 16.4.1 we consider the following name **unavailable**: *Strigidia testaceovirens argentinica* Soula. Below we report the taxonomic history of the subspecies and the label data of the invalid type specimens deposited at CCECL.

### Pelidnota testaceovirens argentinica (Soula, 2006) Unavailable, invalid name

Taxon classificationAnimaliaColeopteraScarabaeidae

Strigidia testaceovirens argentinica Soula, 2006: 62 [original combination, **unavailable, invalid name**].
Pelidnota (Strigidia) testaceovirens argentinica (Soula) [new combination and new subgeneric combination by Özdikmen 2009: 145, **unavailable, invalid name**].
Pelidnota testaceovirens argentinica (Soula) [removal of subgeneric classification by Soula 2009: 115, **unavailable, invalid name**].

#### Distribution.

ARGENTINA: Misiones (Soula 2006).

#### Types.

The following invalid type specimens are deposited at CCECL. 9 invalid ♂ paratypes, 11 invalid ♀ paratypes: six invalid paratypes with identical label data “Puerto Iguazu ARGENTINE (I/93)//Paratype 2006 *Strigidia testaceovirens argentinica* S. Soula” (47030093 to 47030096, exch03 and exch04); three invalid paratypes with identical label data “Puerto Iguazu Arg. M. SOULA det 19//Paratype 2006 *Strigidia testaceovirens argentinica* S. Soula” (47030097 to 47030099); two invalid paratypes with identical label data “Oberá – Misiones ARGENTINA-I/99 Col. Andrés Varga//Paratype 2006 *Strigidia testaceovirens argentinica* S. Soula” (47030100 and 47030101); three invalid paratypes with identical label data “Puerto Iguazu Misiones, Argentine II/1995//Paratype 2006 *Strigidia testaceovirens argentinica* S. Soula” (47030102 to 47030104); “Puerto Iguazu-ARG XII/88.//Paratype 2006 *Strigidia testaceovirens argentinica* S. Soula” (47030110); “ARGENTINA Iguazu Misiones 1996 Coll. M. DURANTON//Paratype 2006 *Strigidia testaceovirens argentinica* S. Soula” (47030105); “ARGENTINE Misiones Iguazu 1997 Coll. M. DURANTON//Paratype 2006 *Strigidia testaceovirens argentinica* S. Soula” (47030106); “Puerto Iguazu 22/11/87 coll. – SOULA [obverse] Misiones (Arg.)//Paratype 2006 *Strigidia testaceovirens argentinica* S. Soula” (47030107); “Puerto Iguazu (Ar.) coll. – SOULA [obverse] Misiones (Arg.) 22/11/87//2006 *Strigidia testaceovirens argentinica* S. Soula” (47030108); “Calilegua NOA 1110 m, 26/01/06 M. SOULA det. 19//2006 *Strigidia testaceovirens argentinica* S. Soula” (47030109). Genitalia card-mounted underneath six of the invalid male paratypes. Box 4618651 SOULA.

#### Remarks.

There is no mention of holotype or allotype specimens of *Pelidnota testaceovirens argentinica* in Soula (2006). Soula (2006) did mention the paratype series, but did not say how many specimens it contains.

## Unavailable names in *Pelidnota* (application of ICZN Article 16.4.2)

We consider the following names proposed by Soula in *Pelidnota* and *Strigidia* as **unavailable** per ICZN Article 16.4.2. which states that fixation of holotype specimens for new names must be accompanied by the following information, “where the holotype or syntypes are extant specimens, by a statement of intent that they will be (or are) deposited in a collection and a statement indicating the name and location of that collection”. The names below were proposed by Soula (2008, 2009, 2010a, 2011), but the descriptions did not state the intent to deposit the holotype specimens in a collection. By applying ICZN Article 16.4.2 herein, the following names are **unavailable**: *Pelidnota arnaudi* Soula 2009, *Pelidnota brusteli* Soula 2010, *Pelidnota chalcothorax septentrionalis* Soula 2009, *Pelidnota degallieri* Soula 2010, *Pelidnota lavalettei* Soula 2008, *Pelidnota lavalettei* Soula 2009, *Pelidnota dieteri* Soula 2011, *Strigidia gracilis decaensi* Soula 2008, *Pelidnota halleri* Demez and Soula 2011, *Pelidnota injantepalominoi* Demez and Soula 2011, *Pelidnota kucerai* Soula 2009, *Pelidnota malyi* Soula 2010: 36–37, *Pelidnota mezai* Soula 2009, *Pelidnota polita darienensis* Soula 2009, *Pelidnota polita orozcoi* Soula 2009, *Pelidnota polita pittieri* Soula 2009, *Pelidnota punctulata decolombia* Soula 2009, *Pelidnota punctulata venezolana* Soula 2009, *Pelidnota raingeardi* Soula 2009, *Pelidnota schneideri* Soula 2010, *Pelidnota simoensi* Soula 2009, and *Pelidnota unicolor subandina* Soula 2009. Below we report the complete taxonomic history of these names and the data from their invalid type specimens that are deposited at CCECL.

### Pelidnota arnaudi

Taxon classificationAnimaliaColeopteraScarabaeidae

Soula, 2009 Unavailable, invalid name

Pelidnota arnaudi Soula, 2009: 32, 72-73 [original combination, **unavailable, invalid name**].

#### Distribution.

BRAZIL: Espírito Santo, São Paulo (Soula 2009).

#### Types.

The following invalid type specimens are deposited at CCECL. 1 ♂ invalid holotype, 1 ♀ invalid allotype, 17 ♂ invalid paratypes, 1 probable ♂ invalid paratype, 1 ♀ invalid paratype: “Paulinia, São Paulo 12/95 M. SOULA det 19//Holotype 2008 *Pelidnota arnaudi* S. Soula” (47030564); “Paulinia, Sao Paulo M. SOULA det 19 [obverse] 12/95//Allotype 2008 *Pelidnota arnaudi* S. Soula” (47030565); four paratypes with identical label data (once São is spelled Sao): “Paulinia, São Paulo 12/95 M. SOULA det 19//Paratype 2008 *Pelidnota arnaudi* S. Soula” (47030566 to 47030569); “Paulinia 12/95 Sao Paulo M. SOULA det 19//Paratype 2008 *Pelidnota arnaudi* S. Soula” (47030570); nine paratypes with identical label data: “Esp. Santo 7/III/97 coll. – SOULA//Paratype 2008 *Pelidnota arnaudi* S. Soula” (47030571 to 47030577, exch33 and exch34); “Esp. Santo 7/III/97 coll. – SOULA//Probable paratype *Pelidnota arnaudi* Soula det. MR Moore ‘15//*Pelidnota arnaudi* Soula Paratype probable” (47030578); three invalid paratypes with identical label data: “Nova Friburgo Rio, XI/2008//Paratype 2009 *Pelidnota arnaudi* S. Soula//Invalid Paratype *Pelidnota arnaudi* Soula det. MR. Moore ‘15” (47030579 and 47030581); “*Pelidnota glauca* Oliv. Brésil//Paratype Soula//Invalid paratype See Soula 2009:73 det. MR Moore ‘15” (47030582). Genitalia card-mounted underneath the invalid male holotype, twelve invalid male paratypes, the probable invalid male paratype and one invalid female paratype. Box 4618670 SOULA.

#### Remarks.

Soula (2009) reported ten *P. arnaudi* Soula paratypes from Espírito Santo, Brazil. Box 4618670 at CCECL contains 9 labeled paratypes from this locality with another specimen, not labeled as a paratype, from this locality. This specimen is considered a probable male paratype of *P. arnaudi* (Soula 2009). Additionally, this series contained a specimen without locality data and an undated, blank Marc Soula paratype label. This male specimen is considered an invalid paratype. Three specimens labeled as being from “Nova Friburgo, Rio, XI/2008” were included in the Box 4618670 Soula near the type series of *P. arnaudi rioensis*. These specimens were labeled as paratypes of *P. arnaudi* and are invalid based on Soula (2009). These specimens were probably mislabeled and were likely intended to be paratypes of *P. arnaudi rioensis* Soula.

### Pelidnota brusteli

Taxon classificationAnimaliaColeopteraScarabaeidae

Soula, 2010 Unavailable, invalid name

Pelidnota brusteli Soula, 2010a: 33 [original combination, **unavailable, invalid name**].

#### Distribution.

PERU: (Soula 2010a, Ratcliffe et al. 2015).

#### Types.

The following invalid type specimen is deposited at CCECL. 1 invalid ♂ holotype: “Tingo Maria Pérou M. SOULA det. 20 [obverse] XII/2005//Holotype 2010 *Pelidnota brusteli* S. Soula” (47030125). Genitalia card-mounted underneath the invalid holotype. Box 4618653 SOULA.

### Pelidnota chalcothorax septentrionalis

Taxon classificationAnimaliaColeopteraScarabaeidae

Soula, 2009 Unavailable, invalid name

Pelidnota chalcothorax septentrionalis Soula, 2009: 94 [original combination, **unavailable, invalid name**].

#### Distribution.

BRAZIL: Bahia, Minas Gerais (Soula 2009).

#### Types.

The following invalid type specimens are deposited at CCECL. 2 invalid ♂ paratypes, 1 invalid ♀ paratype: “S. Antonio da Barra Prov. de Bahia Ch. Pujol 1890//Paratype 2008 *Pelidnota chalcothorax septentrionalis* S. Soula” (47030607); “Villa Victoria Prov. de Bahia Ch. Pujol 1890//Paratype 2008 *Pelidnota chalcothorax septentrionalis* S. Soula” (47030608); “Aguas Vermillas [pro Vermelhas] (B) M. Geraes 3/92 coll. – SOULA//Paratype 2008 *Pelidnota chalcothorax septentrionalis* S. Soula” (47030609). Genitalia card-mounted underneath the invalid male paratypes. Box 4618673 SOULA.

### Pelidnota degallieri

Taxon classificationAnimaliaColeopteraScarabaeidae

Soula, 2010 Unavailable, invalid name

Pelidnota degallieri Soula, 2010a: 32-33 [original combination, **unavailable, invalid name**].

#### Distribution.

VENEZUELA: Bolívar (Soula 2010a).

#### Types.

The following invalid type specimen is deposited at CCECL. 1 invalid ♂ holotype: “Le 15 VII 1986 Route de SANTA ELENA P. K. 35 U. V. Etat du BOLIVAR VENEZUELA J. HAXAIRE & P. BLEUZEN Leg.//Holotype 2010 *Pelidnota degallieri* S. Soula” (47030124). Genitalia card-mounted underneath the invalid holotype. Box 4618653 SOULA.

### Pelidnota dieteri

Taxon classificationAnimaliaColeopteraScarabaeidae

Soula, 2011 Unavailable, invalid name

Pelidnota lavalettei Soula, 2009: 110 [original combination and secondary junior homonym, **unavailable, invalid name**].
Pelidnota dieteri [new replacement name by Soula 2011: 84, **unavailable, invalid name**].

#### Distribution.

BRAZIL: Mato Grosso (Soula 2009).

#### Types.

The following invalid type specimens are deposited at CCECL. 1 invalid ♂ holotype, 1 invalid ♀ allotype, 1 invalid ♀ paratype: “Matto Grosso Brésil M. SOULA det 19//Holotype 2008 *Pelidnota lavalettei* Soula//*Pelidnota dieteri* Soula det. MR MOORE ‘15” (47030727). “Matto Grosso//Allotype 2008 *Pelidnota lavalettei* Soula//*Pelidnota dieteri* Soula det. MR MOORE ‘15” (47030728). “Ipatinga (Minais G) 12/89 M. Soula det. 20//[blank]” (47030729). Genitalia card-mounted underneath the invalid male holotype. Box 4618680 SOULA.

### Pelidnota gracilis decaensi (Soula, 2008) Unavailable, invalid name

Taxon classificationAnimaliaColeopteraScarabaeidae

Strigidia gracilis decaensi Soula, 2008: 35 [original combination, **unavailable, invalid name**].
Pelidnota (Strigidia) gracilis decaensi (Soula) [new combination and new subgeneric combination by Özdikmen 2009: 145, **unavailable, invalid name**].
Pelidnota gracilis decaensi (Soula) [removal of subgeneric classification by Soula 2009: 115, **unavailable, invalid name**].

#### Distribution.

BRAZIL: Paraná (Soula 2008).

#### Types.

The following invalid type specimen is deposited at CCECL. 1 invalid ♂ holotype: “Londrina Paraná 600 m, X/2005 M. SOULA det 19//Holotype *Strigidia gracilis decaensi* S. Soula” (47030298). Genitalia card-mounted underneath the invalid male holotype. Box 4618659 SOULA.

### Pelidnota halleri

Taxon classificationAnimaliaColeopteraScarabaeidae

Demez & Soula, 2011 Unavailable, invalid name

Pelidnota halleri Demez & Soula, 2011: 77 [original combination, **unavailable, invalid name**].
Pelidnota helleri Demez and Soula [incorrect subsequent spelling by Soula 2011: 85].

#### Distribution.

PERU: Loreto (Soula 2011, Ratcliffe et al. 2015).

#### Types.

The following invalid type specimen is deposited at CCECL. 1 invalid ♂ holotype: “Iquitos Loreto VI/2011 M. SOULA det. 19//Holotype 2011 *Pelidnota halleri* D. et S. Soula” (47030133). Genitalia card-mounted underneath the invalid holotype. Box 4618654 SOULA.

#### Remarks.

Soula (2011: 85) misspelled “halleri” as “helleri” in the index.

### Pelidnota injantepalominoi

Taxon classificationAnimaliaColeopteraScarabaeidae

Demez & Soula, 2011 Unavailable, invalid name

Pelidnota injantepalominoi Demez & Soula, 2011: 77–78 [original combination, **unavailable, invalid name**].

#### Distribution.

PERU: Loreto (Soula 2011, Ratcliffe et al. 2015).

#### Types.

The following invalid type specimen is deposited at CCECL. 1 invalid ♂ holotype: “Iquitos VI/2011 M. SOULA det. 19//Holotype *Pelidnota injantepalominoi* D. et S. Soula” (47030134). Genitalia card-mounted underneath the invalid holotype. Box 4618654 SOULA.

### Pelidnota kucerai

Taxon classificationAnimaliaColeopteraScarabaeidae

Soula, 2009 Unavailable, invalid name

Pelidnota kucerai Soula, 2009: 31, 55 [original combination, **unavailable, invalid name**].

#### Distribution.

COLOMBIA: Valle del Cauca (Soula 2009).

#### Types.

The following invalid type specimen was deposited at CCECL. 1 invalid ♂ holotype: “Anchicaya, Valle del Cauca, Colombie, V/93//Holotype 2008 *Pelidnota bucerai* S. Soula” (47030496). Genitalia card-mounted underneath the invalid holotype. Box 4618667 SOULA.

### Pelidnota lavalettei

Taxon classificationAnimaliaColeopteraScarabaeidae

Soula, 2008 Unavailable, invalid name

Pelidnota lavalettei Soula, 2008: 39 [original combination, **unavailable, invalid name**].

#### Distribution.

FRENCH GUIANA (Soula 2008, 2009, 2010c).

#### Types.

The following type specimen is deposited at CCECL. 1 ♂ holotype: “Guyane fr. Est. du dép. M. SOULA det. 20//Holotype 2008 *Pelidnota lavalettei* S. Soula//Holotype of *P. fabricelavalettei* Soula 2009 det. M. R. Moore 2014” (47030132). Genitalia card-mounted underneath holotype. Box 4618654 SOULA.

#### Remarks.

This specimen was the holotype specimen for two species: *P. lavalettei* Soula 2008 (unavailable name) and *P. fabricelavalettei* Soula 2009. *Pelidnota lavalettei* Soula 2008 could have been the senior synonym of *P. lavalettei* Soula 2009, however, the name is unavailable per ICZN Article 16.4. The valid name for this species is *Pelidnota fabricelavalettei* Soula 2009. The genitalia of this holotype specimen appear to be slightly broken or deformed at the apex.

### Pelidnota malyi

Taxon classificationAnimaliaColeopteraScarabaeidae

Soula, 2010 Unavailable, invalid name

Pelidnota malyi Soula, 2010a: 36–37 [original combination, **unavailable, invalid name**].

#### Distribution.

ECUADOR: Napo (Soula 2010a).

#### Types.

The following invalid type specimens are deposited at CCECL. 1 invalid ♂ holotype, 1 invalid ♀ allotype, 2 invalid ♂ paratypes, 2 invalid ♀ paratypes: “Misahuali Oriente Ecuador M. SOULA det 19 [obverse] II/2006//Holotype 2010 *Pelidnota malyi* S. Soula” (47030169); “Misahuali Oriente Ecuador M. SOULA det 19 [obverse] II/2006//Allotype 2010 *Pelidnota malyi* S. Soula” (47030170); “Misahuali Oriente Ecuador M. SOULA det 19 [obverse] II/2006//Paratype *Pelidnota malyi* Soula 2010” (47030171); “PERU - NAPO Misahualli Tijuana 2.06 coll. V. Malý//2,-€//STRIGIDIA Sp. sp. n. ?//coll. V. Malý CZ - Praha//Paratype *Pelidnota malyi* Soula 2010” (47030172); “ECUADOR-Napo Misahualli 450 m 17-21. 4. 93 L. & T. Racheli leg. [obverse] Chalco.//Paratype *Pelidnota malyi* Soula 2010” (47030173); “ECUADOR-Napo Misahualli 450 m 18-20. 10. 1993 L. & T. Racheli leg.//Paratype *Pelidnota malyi* Soula 2010” (47030174). Box 4618656 SOULA.

#### Remarks.

The specific epithet “*malyi*” was used for two separate, distinct species of *Pelidnota* in the same publication (Soula 2010a): *P. malyi* Soula 2010a: 36-37, a metallic green species, and *P. malyi* Soula 2010a: 58, a testaceous species. *Pelidnota malyi* Soula 2010a: 36-37 is an unavailable name.

Soula (2010a) stated that the holotype male was labeled: “Ecuador 2-13/11.2001 Prov. Pichincha Pacto env. 860. VM lgt.” Based on examination of the holotype of *P. malyi* Soula (at CCECL), the label data do not match the description. Rather, the holotype at CCECL is labeled identically to the paratypes of the species. This holotype label is considered invalid and was labeled as a probable paratype male. Soula (2010a: 37) provided label data for the type series, and recorded one label as “PERU (erreur évidemment) – Napo”. Thus, *P. malyi* is known only from Ecuador (contrary to the distribution provided in Moore and Jameson (2013)).

### Pelidnota mezai

Taxon classificationAnimaliaColeopteraScarabaeidae

Soula, 2009 Unavailable, invalid name

Pelidnota mezai Soula, 2009: 33, 101–102 [original combination, **unavailable, invalid name**].

#### Distribution.

PERU (Soula 2009, Ratcliffe et al. 2015).

#### Types.

The following invalid type specimen is deposited at CCECL. 1 invalid ♂ holotype: “Tingo Maria Pérou, X-XI/2005//Holotype 2008 *Pelidnota mezai* S. Soula” (47030730). Genitalia card-mounted underneath the invalid male holotype. Box 4618680 SOULA.

### Pelidnota polita darienensis

Taxon classificationAnimaliaColeopteraScarabaeidae

Soula, 2009 Unavailable, invalid name

Pelidnota polita darienensis Soula, 2009: 46 [original combination, **unavailable, invalid name**].

#### Distribution.

PANAMA: Darien (Soula 2009).

#### Types.

The following invalid type specimens are deposited at CCECL. 1 invalid ♂ holotype, 1 invalid ♀ allotype, 9 invalid ♂ paratypes, 26 invalid ♀ paratypes: Meteti Darien 12/XI/2003 M. SOULA det 19 [obverse] Panama//Holotype 2007 *Pelidnota polita darienensis* S. Soula” (47030498); “Meteti Darien 12/XI/2003 M. SOULA det 19 [obverse] 12/XI/2003//Allotype 2007 P*elidnota politadarienensis* S. Soula” (47030499); four invalid paratypes with identical label data “Meteti Darien Panama M. SOULA det 19 [obverse] 12/XI/2003//Paratype 2007 *Pelidnota polita darienensis* S. Soula” (47030524 to 47030527); “Meteti Darien Panama [obverse] 12/XI/2003//Paratype 2007 *Pelidnota polita darienensis* S. Soula” (47030528); “Meteti Darien Panama M. SOULA det 19 [obverse] II/2004//Paratype 2007 *Pelidnota polita darienensis* S. Soula” (47030529); “Panama Darien M. SOULA det 19//Paratype 2007 *Pelidnota polita darienensis* S. Soula” (47030530); twelve invalid paratypes with identical label data “Meteti, Darien Panama, 12/XI/2003//Paratype 2007 *Pelidnota polita darienensis* S. Soula” (47030515 to 47030523, exch28 to exch30); six invalid paratypes with identical label data “Rio Iglesia, Darien Panama, 20/XI/03//Paratype 2007 *Pelidnota polita darienensis* S. Soula” (47030500 to 47030505); “Rio Iglesia, Darien Panama, 20/XI/03//Paratype 2008 *Pelidnota polita darienensis* S. Soula”//Invalid paratype see Soula 2009:96 det. MR Moore ‘15” (47030506); seven paratypes with identical label data “Aruza abajo Darien 12-25/II/2004, M. SOULA det 19//Paratype 2007 *Pelidnota polita darienensis* S. Soula” (47030507 to 47030512); “Aruza abajo II/2004 M. SOULA det 19//Paratype 2007 *Pelidnota polita darienensis* S. Soula” (47030513); “Aruza abajo Darien II/2004 M. SOULA det 19//Paratype 2007 *Pelidnota polita darienensis* S. Soula” (47030514); “Tocumen Panama 5/91//Paratype 2007 *Pelidnota polita darienensis* S. Soula” (47030531). The genitalia are card-mounted underneath the invalid male holotype, 9 invalid male paratypes,, and 2 invalid female paratypes. Box 4618668 SOULA.

### Pelidnota polita orozcoi

Taxon classificationAnimaliaColeopteraScarabaeidae

Soula, 2009 Unavailable, invalid name

Pelidnota polita orozcoi Soula, 2009: 45 [original combination, **unavailable, invalid name**].

#### Distribution.

COLOMBIA: Meta (Soula 2009).

#### Types.

The invalid holotype ♂ of *Pelidnota polita orozcoi* is at MNHN. The following invalid type specimens are deposited at CCECL. 1 invalid ♀ allotype, 2 invalid ♂ paratypes: “Coll. Nonfried. Columbia.//Allotype 2007 *Pelidnota polita orozcoi* S. Soula” (47030549); “Carimagua; Meta; Colombie; 175m VII/VIII 1999//Paratype 2008 *Pelidnota polita orozcoi* S. Soula det.” (47030550); San Juan de Cordova M. SOULA det 19 [obverse] Cianaga Colombie //Paratype 2008 *Pelidnota polita orozcoi* S. Soula det. (47030551)”. Genitalia card-mounted underneath the two invalid male paratypes. Box 4618669 SOULA.

### Pelidnota polita pittieri

Taxon classificationAnimaliaColeopteraScarabaeidae

Soula, 2009 Unavailable, invalid name

Pelidnota polita pittieri Soula, 2009: 46 [original combination, **unavailable, invalid name**].

#### Distribution.

VENEZUELA: Aragua (Soula 2009).

#### Types.

The following invalid type specimens are deposited at CCECL. 1 invalid ♂ holotype, 1 invalid ♀ allotype, 14 invalid ♂ paratypes, 2 invalid ♀ paratypes, 1 probable invalid ♀ paratype: “P.N. Henri Pittier Choroni; Venezuela V-VI/2005//Holotype 2007 *Pelidnota polita pittieri* S. Soula” (47030532); “P.N. Henri Pittier Choroni; Venezuela V-VI/2005//Allotype 2007 *Pelidnota polita pittieri* S. Soula” (47030533); twelve paratypes with identical label data “P.N. Henri Pittier Choroni ; Venezuela V-VI/2005//Paratype 2007 *Pelidnota polita pittieri* S. Soula” (47030534 to 47030543, exch31 and exch32); “P.N. Henri Pittier Choroni; Venezuela V-VI/2005//Paratype 2007 *Pelidnota polita darienensis* S. Soula//*Pelidnota polita pittieri* Soula probable paratype det. MR Moore ’15” (47030544); “Caracas. Mus: Drews.//ZOOL. MUSEUM DK COPENHAGEN//Paratype 2008 *Pelidnota polita darienensis* S. Soula” (47030545); “Caracas//ZOOL. MUSEUM DK COPENHAGEN//Paratype 2008 *Pelidnota polita darienensis* S. Soula” (47030546); two paratypes with identical label data “N. Venezuela S. Klages 1904//Paratype 2008 *Pelidnota polita pittieri* S. Soula” (47030547 and 47030548). Genitalia card-mounted underneath the invalid male holotype, 5 invalid male paratypes, and 1 probable invalid female paratype. Box 4618668 SOULA.

#### Remarks.

The probable female paratype from P. N. Henri Pittier has a paratype label indicating that this specimen was determined as *Pelidnota polita darienensis* Soula. Soula (2009: 46) mentions 13 paratypes from this locality and only twelve are labeled *P. polita pittieri* paratypes. The matching locality data and proximity to *P. polita pittieri* in Box 461669 SOULA indicated this specimen is likely the 13^th^ invalid paratype from this locality.

### Pelidnota punctulata decolombia

Taxon classificationAnimaliaColeopteraScarabaeidae

Soula, 2009 Unavailable, invalid name

Pelidnota punctulata decolombia Soula, 2009: 80 [original combination, **unavailable, invalid name**].

#### Distribution.

COLOMBIA (Soula 2009).

#### Types.

The invalid holotype ♂ of *Pelidnota punctulata decolombia* is at MNHN.

### Pelidnota punctulata venezolana

Taxon classificationAnimaliaColeopteraScarabaeidae

Soula, 2009 Unavailable, invalid name

Pelidnota punctulata venezolana Soula, 2009: 80 [original combination, **unavailable, invalid name**].

#### Distribution.

VENEZUELA (Soula 2009).

#### Types.

The invalid holotype ♂ of *Pelidnota punctulata venezolana* is at MNHN.

### Pelidnota raingeardi

Taxon classificationAnimaliaColeopteraScarabaeidae

Soula, 2009 Unavailable, invalid name

Pelidnota raingeardi Soula, 2009: 33, 97 [original combination, **unavailable, invalid name**].

#### Distribution.

ECUADOR: Napo (Soula 2009).

#### Types.

The following invalid type specimens are deposited at CCECL. 1 invalid ♂ holotype, 1 invalid ♀ allotype, 13 invalid ♂ paratypes, 4 invalid ♀ paratypes: “Napo - Coca Ecuador VIII-1982 Onoré leg.//Holotype 2008 *Pelidnota raingeardi* Soula” (47030651); “Napo - Coca Ecuador VIII-1982 Onoré leg.//Allotype 2008 *Pelidnota raingeardi* Soula” (47030652); three invalid female paratypes with identical label data: “Napo - Coca Ecuador VIII-1982 Onoré leg.//Paratype 2008 *Pelidnota raingeardi* S. Soula” (47030653 to 47030655); three invalid paratypes with identical label data: “Baeza (Eq.) 08/91 coll. – SOULA//Paratype 2008 *Pelidnota raingeardi* S. Soula” (47030656 to 47030658); “Tena [arrow] Loreto (E) pk 30 8/90//Paratype 2008 *Pelidnota raingeardi* Soula” (47030659); “Tena [arrow] Loreto (E) 7/90//Paratype 2008 *Pelidnota raingeardi* Soula” (47030660); “Tena (Equateur) 05/91//Paratype 2008 *Pelidnota raingeardi* Soula” (47030661);“Tena (E) 9/90 pk 30 //Paratype 2008 *Pelidnota raingeardi* Soula” (47030662); “[arrow] Loreto (E) 8/90//Paratype 2008 *Pelidnota raingeardi* Soula” (exch37); “Loreto (E) 9/90//Paratype 2008 *Pelidnota raingeardi* Soula” (47030663); “San Jorge ? (E) 8/90 [obverse] Equateur !//Paratype 2008 *Pelidnota raingeardi* Soula” (47030664); “Misahuali 9/91//Paratype 2008 *Pelidnota raingeardi* Soula” (47030665); “Misahuali 9/91//Paratype 2008 *Pelidnota raingeardi* Soula” (47030665); “Piste Puyo Macas P.L. Rio Pastaza (800m) 30/7/88 Pastaza (E) [obverse] 30/7/88 Pastaza (E)//Paratype 2008 *Pelidnota raingeardi* Soula” (47030666); “Route de Baños E 30/7/88 Macas Rio Pastaza [obverse] Rio Pastaza//Paratype 2008 *Pelidnota raingeardi* Soula” (47030667); “Rurrerabaque (sic) Bolivie M. SOULA det 19 [obverse] II/98//Paratype 2008 *Pelidnota raingeardi* Soula//Invalid Paratype *Pelidnota raingeardi* Soula det. MR Moore ‘15” (47030668). Genitalia mounted underneath the invalid male holotype and ten invalid male paratypes. Box 4618678 SOULA.

#### Remarks.

Box 4618678 SOULA contains a male specimen from Ruzzezbaque, Bolivia labeled as a paratype of *Pelidnota raingeardi* Soula. Soula (2009) does not list this specimen, or any specimens from Bolivia, as being part of the type series of *P. raingeardi* and thus this specimen is an invalid paratype. This male specimen is very different (small and brown) from the relatively large, green specimens in the *P. raingeardi* type series. This specimen was likely accidentally labeled as a paratype.

### Pelidnota schneideri

Taxon classificationAnimaliaColeopteraScarabaeidae

Soula, 2010 Unavailable, invalid name

Pelidnota schneideri Soula, 2010a: 35 [original combination, **unavailable, invalid name**].

#### Distribution.

PERU: Loreto (Soula 2010a, Ratcliffe et al. 2015).

#### Types.

The following invalid type specimens are deposited at CCECL. 1 invalid ♂ holotype, 1 invalid ♀ allotype, 18 invalid ♂ paratypes, 23 invalid ♀ paratypes: “Iquitos, Loreto, Pérou, II/2010//Holotype 2010 *Pelidnota schneideri* S. Soula” (47030357); “Iquitos; Loreto, Pérou; 200m X/2002//Allotype *Pelidnota schneideri* S. 2010 Soula” (47030358); two specimens with identical label data “Iquitos; Loreto, Pérou; X/2002//Paratype 2010 *Pelidnota schneideri* Soula” (47030359 and 47030360); “Iquitos; Loreto Pérou; 200m X/2002//Paratype 2010 *Pelidnota schneideri* Soula” (47030375); three invalid paratypes with identical label data “Iquitos, Loreto Pérou, II/2010//Paratype 2010 *Pelidnota schneideri* Soula” (47030361 to 47030363); three invalid paratypes with identical label data “Iquitos ; Loreto Pérou; XI-XII/2004//Paratype 2010 *Pelidnota schneideri* Soula” (47030364 to 47030366); three invalid paratypes with identical label data “Iquitos, Loreto, Pérou, I-II/2005//Paratype 2010 *Pelidnota schneideri* Soula” (47030367 and 47030368, exch21); “Iquitos, Loreto Pérou, VI/2010 [crossed out] [obverse] XI/2009//Paratype 2010 *Pelidnota schneideri* Soula” (47030369); “Iquitos, Loreto Pérou, VI [crossed out] XI/2010 [obverse] XI/2009//Paratype 2010 *Pelidnota schneideri* Soula” (47030370); three invalid paratypes with identical label data “Iquitos, Loreto Pérou, X/XI/2004//Paratype 2010 *Pelidnota schneideri* Soula” (47030371 to 47030373); “Iquitos, Loreto Pérou, II/2005//Paratype 2010 *Pelidnota schneideri* Soula” (47030374); “Iquitos Loreto Pérou M. SOULA det 19//Paratype 2010 Pelidnota schneideri Soula” (47030376); “Iquitos, Loreto Pérou; VIII/2003//Paratype 2010 *Pelidnota schneideri* Soula” (47030377); “Iquitos V/2002 M. SOULA det 19//Paratype 2010 *Pelidnota schneideri* Soula” (47030378); “Iquitos Pérou II-III/2004 M. SOULA det 19//Paratype 2010 *Pelidnota schneideri* Soula” (47030379); four invalid paratypes with identical label data “Iquitos, Loreto Pérou, VI/2010//Paratype 2010 *Pelidnota schneideri* Soula” (47030380 and 47030381, exch19 and exch20); two invalid paratypes with identical label data “San Pablo Loreto Pérou M. Soula det 19 [obverse] X/2003//Paratype 2010 *Pelidnota schneideri* Soula” (47030382 and 47030383); “Colombie Caqueta M. SOULA det 19//Paratype 2010 *Pelidnota schneideri* Soula” (47030384); “*C. osculatii*? 1/9/91 Tena (E)//Paratype 2010 *Pelidnota schneideri* Soula” (47030385); four invalid paratypes with identical label data “San Jorge (Equateur) 8/90//Paratype 2010 *Pelidnota schneideri* Soula” (47030386 to 47030388, exch18); three invalid paratypes with identical label data “San Jorge (E) 8/90//Paratype 2010 *Pelidnota schneideri* Soula” (47030389 to 47030391); three invalid paratypes with identical label data “San Jorge 8/90 (Eq.) coll. – SOULA//Paratype 2010 *Pelidnota schneideri* Soula”(47030392 to 47030394); “Macas Ecuador or.//Paratype 2010 *Pelidnota schneideri* Soula” (47030395). Genitalia card-mounted underneath the invalid male holotype and invalid paratype specimens. Box 4618662 SOULA. There are probably more invalid paratypes of *Pelidnota schneideri* at ZMHB (Soula 2010a).

### Pelidnota simoensi

Taxon classificationAnimaliaColeopteraScarabaeidae

Soula, 2009 Unavailable, invalid name

Pelidnota simoensi Soula, 2009: 33, 99–100 [original combination, **unavailable, invalid name**].

#### Distribution.

BOLIVIA: La Paz (Soula 2009).

#### Types.

The following invalid type specimens are deposited at CCECL. 1 invalid ♂ holotype, 1 invalid ♀ allotype, 5 invalid ♂ paratypes, 3 invalid ♀ paratypes: “Région des Yungas Bolivie//Holotype 2009 *Pelidnota simoensi* S. Soula” (47030641); “Nord-Yungas 1800m; Bolivie//Allotype 2008 *Pelidnota simoensi* S. Soula” (47030642); “*P. brevissima* Caranavi N. Yungas 10/90 (B)//Paratype 2008 *Pelidnota simoensi* S. Soula” (47030643); “Yungas (Bol.) coll. – SOULA//Paratype 2008 *Pelidnota simoensi* S. Soula” (47030644); “Inca-Huara (1450m) - Bolivie XI/95 Lecourt leg.//Paratype 2008 *Pelidnota simoensi* S. Soula” (47030645); “Pointe Villa 1500 m coll. – SOULA [obverse] 11/10/96 La Paz Prov.//Paratype 2008 *Pelidnota simoensi* S. Soula” (47030646); “Pointe Villa 1500 m 11/10/96 coll. – SOULA [obverse] 11/10/96 La Paz Prov.//Paratype 2008 *Pelidnota simoensi* S. Soula” (47030647); “Pointe Villa (1500 m) coll. – SOULA [obverse] La Paz Prov.//Paratype 2009 *Pelidnota simoensi* S. Soula” (47030648); “N. Venezuela S. Klages 1904//*Pelidnota prasina* Burm.//Paratype 2008 *Pelidnota simoensi* S. Soula//Invalid Paratype *Pelidnota simoensi* Soula det. MR Moore ‘15” (47030649); “P.N. Henri Pittier Choroni; Venezuela V-VI/2005//Paratype 2008 *Pelidnota simoensi* S. Soula//Invalid Paratype *Pelidnota simoensi* Soula det. MR Moore ‘15” (47030650). Genitalia mounted underneath the invalid male holotype and five invalid male paratypes. Box 4618678 SOULA.

#### Remarks.

Two male specimens labeled as paratypes of *P. simoensi* Soula are considered invalid paratypes because their localities are not reported in Soula (2009). These specimens have paratype labels from 2008. It is likely that these specimens were accidentally omitted from publication (Soula 2009).

### Pelidnota unicolor subandina

Taxon classificationAnimaliaColeopteraScarabaeidae

Soula, 2009 Unavailable, invalid name

Pelidnota unicolor subandina Soula, 2009: 93 [original combination, **unavailable, invalid name**].

#### Distribution.

PERU: Junín (Soula 2009, Ratcliffe et al. 2015).

#### Remarks.

Soula (2009) stated there was a holotype male of *Pelidnota unicolor subandina* from “Chanchamayo, Pérou, 2000m” but we did not find this specimen at CCECL.

### †Pelidnotites

Taxon classificationAnimaliaColeopteraScarabaeidae

Cockerell, 1920

Pelidnotites Cockerell, 1920: 462–463.

#### Type species.

*Pelidnotites atavus* Cockerell, 1920: 463, by monotypy.

#### Gender.

Masculine.

#### Species.

1 species.

### †Pelidnotites atavus

Taxon classificationAnimaliaColeopteraScarabaeidae

Cockerell, 1920

Pelidnotites atavus Cockerell, 1920: 462–463 [original combination].

#### Distribution.

ENGLAND [EOCENE] (Cockerell 1920, Carpenter 1992).

#### Remarks.

The true identity of this species is uncertain. Cockerell (1920) stated that the species was similar to *Pelidnota* and *Cotalpa*. The original description provided an illustration of the basal portion of the elytron as well as the abdomen (in ventral view) (Cockerell 1920: 463, Figure 6). The specimen was identified as British Museum number 19004 (J. S. Gardner).

### 

Taxon classificationAnimaliaColeopteraScarabaeidae

Mondaca & Valencia, 2016

Peruquime Mondaca & Valencia, 2016: 3–4.

#### Type species.

*Peruquime arequipensis* Mondaca & Valencia, 2016: 4–6, by monotypy.

#### Gender.

Feminine.

#### Species.

1 species.

### Peruquime arequipensis

Taxon classificationAnimaliaColeopteraScarabaeidae

Mondaca & Valencia, 2016

Peruquime arequipensis Mondaca & Valencia, 2016: 4–6 [original combination].

#### Distribution.

PERU: Arequipa (Mondaca and Valencia 2016).

#### Types.

**Figure 98.**
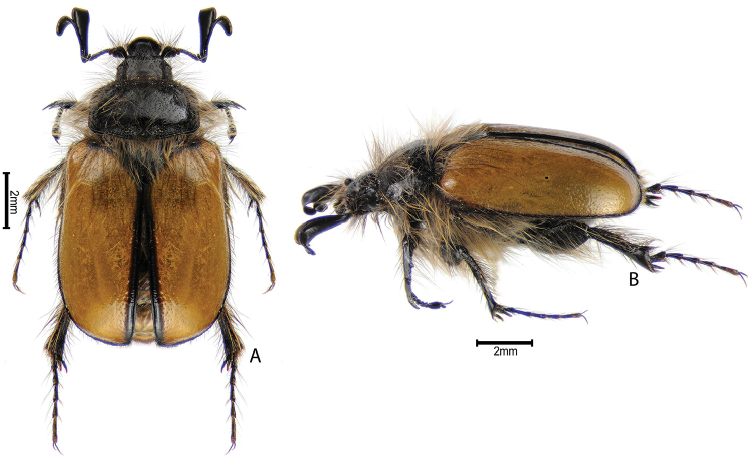
*Peruquime arequipensis* Mondaca and Valencia male paratype specimen from JMEC. **A** Dorsal habitus **B** Lateral habitus. Photographs courtesy of José Mondaca, Santiago, Chile.

Holotype ♂ at MHNP. Male paratypes (40) distributed at several institutions including CMNC, IEXA, MHNP, and UNSM (Mondaca and Valencia 2016). An exemplar specimen is figured (Fig. 98).

### 

Taxon classificationAnimaliaColeopteraScarabaeidae

Ohaus, 1910

Pseudogeniates Ohaus, 1910a: 179–180.

#### Type species.

*Pseudogeniates richterianus* Ohaus, 1910a: 180, by monotypy.

#### Gender.

Masculine.

#### Species.

3 species.

### Pseudogeniates cordobaensis

Taxon classificationAnimaliaColeopteraScarabaeidae

Moore, Jameson, Garner, Audibert, Smith, and Seidel, sp. n.

Pseudogeniates cordobaensis Soula, 2009: 122 [original combination, **unavailable name**].
Pseudogeniates cordobaensis Soula [in Jameson & Ocampo, 2012, **unavailable name**].
Pseudogeniates cordobaensis Moore, Jameson, Garner, Audibert, Smith, and Seidel, **sp. n.**

#### Distribution.

ARGENTINA: Catamarca, Córdoba (Soula 2009, Jameson and Ocampo 2012).

#### Types.

Holotype and 7 paratypes. 1 ♂ holotype at ZMHB (= paralectotype of *Pseudogeniates intermedius*) (see Jameson and Ocampo 2012) from Ohaus’s type series of *P. intermedius*, at ZMHB labeled (from Jameson and Ocampo 2012: 41): “Argentina S. d. Cordoba J. Hubrich S.” (typeset, white label)//male symbol //Pseudogeniates intermedius cotype Ohs. (Ohaus’s handwritten, red label)//SYNTYPUS Pseudogeniates intermedius Ohaus, 1914 labeled by MNHUS 2007 (typeset, red label)//Paralectotype 2009 Pseudogeniates intermedius Oh. Soula det. (typeset and handwritten, red label)//Holotype 2009 Pseudogeniates cordobaensis Soula Soula (handwritten and typeset, red label)//Pseudogeniates cordobaensis Moore, Jameson, Garner, Audibert, Smith, and Seidel 2016 HOLOTYPE”. The holotype specimen (previously used by Soula) is the male paralectotype of *Ps. intermedius* Ohaus with data “Argentina/S. d. Cordoba/J Hubrich S.” (Soula 2009: 122). Ohaus’ type series for *Ps. intermedius* included three specimens from Santiago del Estero in Argentina and one specimen (=*Ps. cordobaensis*) from Huerta Grande in the Sierra de Cordóba, Córdoba Province, Argentina (Ohaus 1914). 1 ♂ paratype with pronotum damaged at ZMHB labeled: “ARGENTINA: Catamarca, Salar de Pipanaco, Pio Brizuela 37 km S Andalgalá, 27°49’34”S, 66°14’47”W, XII-5-2003. F. C. Ocampo//Pseudogeniates cordobaensis Soula det M.L. Jameson 2012//Pseudogeniates cordobaensis Moore, Jameson, Garner, Audibert, Smith, and Seidel 2016 PARATYPE”. 1 ♂ paratype at CCECL labeled as ZMHB paratype and with mouthparts, hindwing, and tarsomere card-mounted under specimen. 2 ♂ paratypes at IAZA labeled as ZMHB paratype except one includes the label: “Pseudogeniates sp. Det. F.C. Ocampo 2007”. 1 ♂ paratype at UNSM labeled as ZMHB paratype. 1 ♂ paratype at MSPC labeled as ZMHB paratype and with hindwing card-mounted under specimen. 1 ♂ paratype at MLJC labeled: “Ra Catamarca 37 km S Andalgalá Salar Pipanaco Pío Brizuelas 06-XII-03 S Roig 27°49’34”S 66°14’47”W 751 msm//mouthparts, spiculum gastrale, male genitalia card-mounted//wing card-mounted//Pseudogeniates cordobaensis Soula Det M.L. Jameson 2012 (type set and hand-written)//Pseudogeniates cordobaensis Moore, Jameson, Garner, Audibert, Smith, and Seidel 2016 PARATYPE det. M.L. Jameson 2016 (hand-written, yellow label)”.

#### Remarks.

For all new species-group names, the holotype and the type depository must be explicitly stated for the name to be deemed available (ICZN Art. 16.4). Because Soula (2009) did not explicitly state the location of the holotype specimen for *Ps. cordobaensis*, the original combination is unavailable. Jameson and Ocampo (2012), in their revision of the genus *Pseudogeniates*, did not notice this nomenclatural problem. They redescribed the species, attributing the name to Soula. Because Art. 16.1. (ICZN 1999) states that new names, including replacement names, must be explicitly indicated as intentionally new, *Ps. cordobaensis* cannot be attributed to Jameson and Ocampo (2012). Since *Ps. cordobaensis* has never been properly made available, we describe it here as a new species.

#### Description of *Pseudogeniates cordobaensis*, new species.

**Figure 99.**
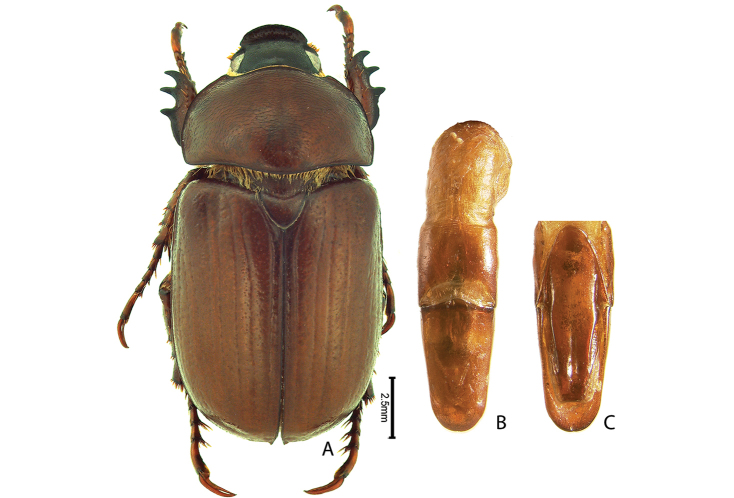
*Pseudogeniates cordobaensis*, sp. n. male paratype from MLJC. **A** Dorsal habitus **B** Male genitalia, dorsal view **C** Male parameres, ventral view.

A full redescription of the species was provided in (Jameson and Ocampo 2012, see http://species-id.net/wiki/Pseudogeniates_cordobaensis). This species is separated from other species in the genus *Pseudogeniates* by the form of the mentum that is pentagonal (width subequal to length) and with the inner apex that projects anteriorly with an inner shelf (Jameson and Ocampo 2012: Fig. 8). Congeners, in comparison, possess a mentum that is longer than wide. Additionally, *Ps. cordobaensis* is distinguished by the ventral plate of the male parameres that is nearly as long as the dorsal plate and the apex that is quadrate (Jameson and Ocampo 2012: Fig. 19). In comparison, the ventral plate of *Ps. richterianus* is short (about half the length of the dorsal plate) and converges to a quadrate apex, whereas *Ps. intermedius* possesses a ventral plate that is nearly as long as the dorsal plate, but with sides that are constricted preapically and with a rounded apex (Fig. 99).

### Pseudogeniates intermedius

Taxon classificationAnimaliaColeopteraScarabaeidae

Ohaus, 1914

Pseudogeniates intermedius Ohaus, 1914: 303 [original combination].

#### Distribution.

ARGENTINA: Santiago del Estero (Ohaus 1914, 1918, 1934b, Blackwelder 1944, Machatschke 1972, Krajcik 2008, Soula 2009, Jameson and Ocampo 2012).

#### Types.

1 ♂ lectotype and 3 paralectotypes at ZMHB (Jameson and Ocampo 2012).

### Pseudogeniates richterianus

Taxon classificationAnimaliaColeopteraScarabaeidae

Ohaus, 1910

Pseudogeniates richterianus Ohaus, 1910a: 180 [original combination].
Pseudogeniates richteri Ohaus, 1934b: T. 2, f. 6 [*lapsus*]

#### Distribution.

ARGENTINA: Buenos Aires, Mendoza, Neuquén, Río Negro, San Juan, Santa Fe (Ohaus 1910a, 1914, 1918, 1934b, Blackwelder 1944, Machatschke 1972, Krajcik 2008, Soula 2009, Jameson and Ocampo 2012).

#### Types.

1 ♀ lectotype and 1 paralectotype at ZMHB (Jameson and Ocampo 2012).

### 

Taxon classificationAnimaliaColeopteraScarabaeidae

Soula, 2006

Sorocha Soula, 2006: 86–87.

#### Type species.

*Pelidnota acutipennis* F. Bates, 1904: 255, 263–264, original designation.

#### Gender.

Feminine.

#### Species.

16 species and subspecies.

#### Remarks.

Krajcik (2012, 2013) considered *Sorocha* to be a junior synonym of *Pelidnota*. While clarifying the subgeneric classification of *Pelidnota* (due to homonymy of the genus-group name *Odontognathus* Laporte), Özdikmen (2009) included five species classified by Soula in the genus *Sorocha* Soula (including the type species *P. acutipennis* F. Bates) in Pelidnota (Strigidia).

### Sorocha acutipennis

Taxon classificationAnimaliaColeopteraScarabaeidae

(F. Bates, 1904)

Pelidnota acutipennis F. Bates, 1904: 255, 263–264 [original combination].
Pelidnota (Ganonota) acutipennis F. Bates [new subgeneric combination by Ohaus 1918: 25].
Pelidnota (Strigidia) acutipennis F. Bates [new subgeneric combination by Machatschke 1970: 157].
Pelidnota (Odontognathus) acutipennis F. Bates [new subgeneric combination by Hardy 1975: 4].
Sorocha acutipennis (F. Bates) [new combination by Soula 2006: 87].
Pelidnota (Strigidia) acutipennis F. Bates [revised combination and revised subgeneric combination by Özdikmen 2009: 145].
Sorocha acutipennis (F. Bates) [**revised combination**].

#### Distribution.

VENEZUELA: Merida, Tachira (F. Bates 1904, Ohaus 1918, 1934b, Blackwelder 1944, Machatschke 1972, Soula 2006, Krajcik 2008).

#### Types.

Soula designated 1 ♀ syntype, but he did not provide the depository (Soula 2006). The holotype ♀ of *P. acutipennis* is at the BMNH.

#### Remarks.

CCECL contains a specimen of *S. acutipennis* labeled as a male alloréférent with the following data: 1 ♂ alloréférent: “Zea-Mérida I/2002 VENEZUELA Col. Andrés Varga//Alloréférent ♂ de *Strigidia acutipennis* (Oh.) M. SOULA det 2005” (47030980). Genitalia card-mounted underneath the male alloréférent. Box 4618687 SOULA. While clarifying the subgeneric classification of *Pelidnota* (due to homonymy of the genus-group name *Odontognathus* Laporte), Özdikmen (2009) listed *Pelidnota acutipennis* within Pelidnota (Strigidia). We think that Özdikmen (2009) was unaware of Soula’s (2006) erection of the genus *Sorocha* for some species previously classified in various subgenera of *Pelidnota*. We classify this species in *Sorocha* as *S. acutipennis* (F. Bates) until the validity of *Sorocha* is evaluated by phylogenetic analysis.

### Sorocha bousqueti

Taxon classificationAnimaliaColeopteraScarabaeidae

Soula, 2006

Sorocha bousqueti Soula, 2006: 88–89 [original combination].

#### Distribution.

PERU: San Martin (Soula 2006, Ratcliffe et al. 2015).

#### Types.

The following specimen is deposited at CCECL. 1 ♂ holotype: “Pérou Moyobamba M. de Mathan 1888//Holotype *Sorocha bousqueti* Sou. Soula” (47030964). Genitalia card-mounted underneath the male holotype. Box 4618687 SOULA.

### Sorocha champenoisi

Taxon classificationAnimaliaColeopteraScarabaeidae

Soula, 2006

Sorocha champenoisi Soula, 2006: 94 [original combination].

#### Distribution.

PERU: Huánuco (Soula 2006, Ratcliffe et al. 2015).

#### Types.

The following specimen is deposited at CMNC. 1 ♂ holotype: “PERU Huanuco Tingo Maria Universidad Coll. Martínez Dic.-974//H. & A. HOWDEN COLLECTION *ex.* A. Martinez coll.//Holotype 2006 *Sorocha champenoisi* S. Soula”.

### Sorocha chapellei

Taxon classificationAnimaliaColeopteraScarabaeidae

Demez & Soula, 2011

Sorocha chapellei Demez & Soula, 2011: 79–80 [original combination].

#### Distribution.

PERU: Ucayali, Junín (Soula 2011, Ratcliffe et al. 2015).

#### Types.

The following specimens are deposited at CCECL. 1 ♂ holotype, 1 ♀ allotype, 1 ♂ paratype: “Atalaya Pérou VIII/2010 M. SOULA det 19//Holotype 2010 *Sorocha chapellei* S. Soula” (47030967); “Satipo Rio Tambo M. SOULA det 19 [obverse] IX/2010//Allotype 2010 *Sorocha chapellei* S. Soula” (47030968); “Satipo Rio Tambo M. SOULA det 19 [obverse] IX/2010//Paratype 2010 *Sorocha chapellei* S. Soula” (47030969). Genitalia card-mounted underneath the male holotype. Box 4618687 SOULA.

### Sorocha damasoi

Taxon classificationAnimaliaColeopteraScarabaeidae

Soula, 2006

Sorocha damasoi Soula, 2006: 92 [original combination].

#### Distribution.

PERU: Huánuco (Soula 2006, Ratcliffe et al. 2015).

#### Types.

The following specimens are deposited at CCECL. 1 ♂ holotype, 2 ♂ paratypes: “Chinchao//Holotype 2005 *Sorocha damasoi* Sou. Soula” (47030977); “Tingo Maria Pérou, X/2005//Paratype 2005 *Sorocha damasoi* Sou. Soula det.” (47030978); “Tingo Maria Pérou, X/2005//Paratype *Sorocha damasoi* Sou. Soula det. 2005” (47030979). Genitalia card-mounted underneath the male holotype and the two male paratypes. Box 4618687 SOULA.

### Sorocha lamasi lamasi

Taxon classificationAnimaliaColeopteraScarabaeidae

Soula, 2006

Sorocha lamasi lamasi Soula, 2006: 91–92 [original combination].

#### Distribution.

PERU: Pasco (Soula 2006, Ratcliffe et al. 2015).

#### Types.

The following specimens are deposited at CCECL. 1 ♂ holotype, 1 ♀ allotype, 1 ♂ paratype, 2 ♀ paratypes: “Oxapampa (1800m) 10/88//Holotype *Sorocha lamasi* Sou. Soula det. 2005” (47030970); “Oxapampa (1800m) 10/88//Allotype *Sorocha lamasi* Sou. Soula det. 2005” (47030971); three paratypes with identical label data: “Oxapampa (1800m) 10/88//Paratype *Sorocha lamasi* Sou. Soula det. 2005” (47030972 to 47030974). Genitalia card-mounted underneath the male holotype. Box 4618687 SOULA.

### Sorocha lamasi satipoensis

Taxon classificationAnimaliaColeopteraScarabaeidae

Soula, 2006

Sorocha lamasi satipoensis Soula, 2006: 92 [original combination].

#### Distribution.

PERU: Junín (Soula 2006, Ratcliffe et al. 2015).

#### Types.

The following specimens are deposited at CCECL. 1 ♂ holotype, 1 ♀ allotype: “Satipo Junin Pérou, II/III/2004//Holotype 2006 *Sorocha lamasi satipoensis* Soula det. Sou.” (47030975); “Satipo Junin Pérou, II/III/2004//Allotype 2006 *Sorocha lamasi satipoensis* Soula det. Sou.” (47030976). Genitalia card-mounted underneath the male holotype. Box 4618687 SOULA.

### Sorocha martinezi

Taxon classificationAnimaliaColeopteraScarabaeidae

Soula, 2006

Sorocha martinezi Soula, 2006: 93–94 [original combination].

#### Distribution.

BOLIVIA: Cochabamba (Soula 2006).

#### Types.

The following specimen is deposited at CCECL. 1 ♂ paratype: “BOLIVIA D° Cochabamba Pcia. Chapare Yungas del Palmas LOCOTAL, 1200 m Coll. Martínez Marz. 952//*Pelidnota* ♂ [*plicipennis* crossed out] Ohs A. MARTINEZ-DET.1967//H. & A. HOWDEN COLLECTION ex A. Martinez coll.//Paratype *Sorocha martinezi* S. 2006 Soula” (47030981). Box 4618688 SOULA. The following specimens are deposited at CMNC: 5 ♂ and 4 ♀ paratypes all with the same locality label as the paratype above, 1 ♂ paratype “BOLIVIA D° Cochabamba Pcia. Chapare Km. 145.-1200 mts. Locotal Coll. Martínez. Feb.-952//H. & A. HOWDEN COLLECTION ex A. Martinez coll.//*Pelidnota* ♂ *plicipennis* Ohs. A. MARTINEZ-DET.1967//Paratype *Sorocha martinezi* S. 2006 Soula”.

#### Remarks.

The holotype of *Sorocha martinezi* is deposited at CMNC (Soula 2006, Genier pers. comm. 2015).

### Sorocha marxi

Taxon classificationAnimaliaColeopteraScarabaeidae

Soula, 2006

Sorocha marxi Soula, 2006: 90 [original combination].

#### Distribution.

ECUADOR: Napo (Soula 2006).

#### Types.

**Figure 100.**
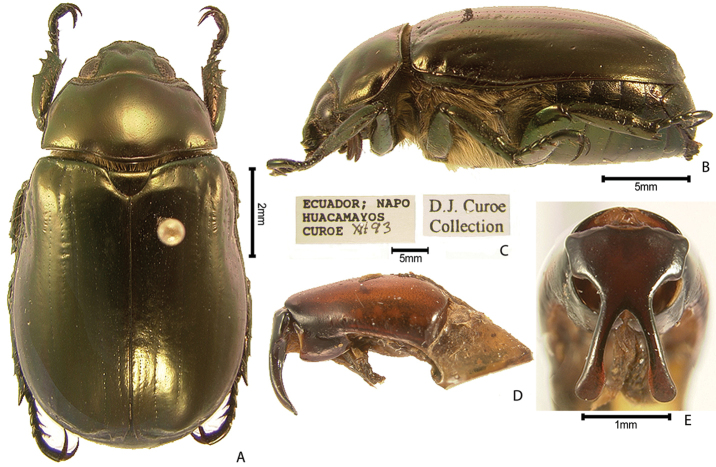
*Sorocha marxi* Soula male specimen from DJCC. **A** Dorsal habitus **B** Lateral habitus **C** Specimen labels **D** Male genitalia, lateral view **E** Male parameres, caudal view.

The following specimens are deposited at CCECL. 1 ♂ holotype, 1 ♂ probable paratype, 1 ♀ paratype: “ECUADOR NAPO SUMACO 10-20 NOV 1995 ABrarrágan//Holotype 2005 *Sorocha marxi* Sou. Soula” (47030965); “ECUADOR OCCIDENTE CANAR Rte Gun El Triumfo parroquia Chontamarca (500 m) 14 mars 1980 Rec. PORION-BERTRAND//Paratype *Sorocha marxi* S. 2005 Soula” (47030966); “ECUADOR OCCIDENTE CANAR Rte Gun El Triumfo parroquia Chontamarca (500 m) 09 mars 1980 Rec. PORION-BERTRAND//Holotype 2006 *Sorocha marxi occidentalis* S. Soula//Probable paratype *Sorocha marxi* Soula det. M. R. Moore ‘15” (47030952). Genitalia card-mounted underneath the male holotype. Box 4618687 SOULA and 4616344 PORION. An exemplar specimen is figured (Fig. 100).

### Sorocha maylini

Taxon classificationAnimaliaColeopteraScarabaeidae

Soula, 2006

Sorocha maylini Soula, 2006: 90–91 [original combination].

#### Distribution.

BOLIVIA: Santa Cruz (DJCC). PERU: Puno (Soula 2006, Ratcliffe et al. 2015).

#### Types.

**Figure 101.**
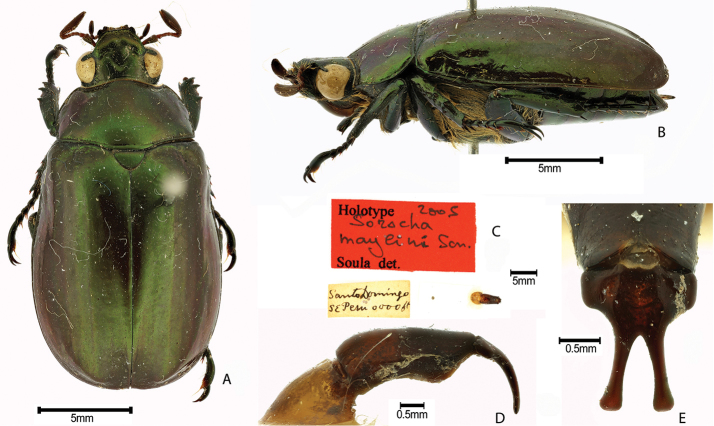
*Sorocha maylini* Soula holotype male from BMNH. **A** Dorsal habitus **B** Lateral habitus **C** Specimen labels and male genitalia **D** Male genitalia, lateral view **E** Male parameres, caudal view.

According to Soula (2006), the holotype of *Sorocha maylini* should have been housed at CCECL (Soula 2006). We located the holotype (Fig. 101) ♂ , allotype, 2 paratypes at BMNH with the following label data: holotype ♂ (dissected) “[handwritten] Santo domingo / SE Peru 6000ft //[red label] [printed and handwritten] Holotype 2005 / Sorocha / maylini Sou. / Soula det.”; allotype “[handwritten] Santo domingo / SE Peru 6000ft //[red label] [printed and handwritten] Allotype / Sorocha / maylini Sou. / Soula det. 2005”; 1 paratype labeled “[handwritten] Santo domingo / SE Peru 6000 //[red label] [printed and handwritten] Paratype / Sorocha / maylini Sou. / Soula det.”; a second paratype ♂ dissected and labeled “[handwritten] Santo domingo / SE Peru 6000ft //[red label] [printed and handwritten] Paratype 2005 / Sorocha / maylini Sou. / Soula det. //[printed] Nevinson Coll. / 1918-14.”

### Sorocha nadiae

Taxon classificationAnimaliaColeopteraScarabaeidae

(Martínez, 1978)

Pelidnota (Odontognathus) nadiae Martínez, 1978: 125–129 [original combination].
Sorocha nadiae (Martínez) [new combination by Soula 2006: 96].
Pelidnota (Strigidia) nadiae Martínez [revised combination and new subgeneric combination by Özdikmen 2009: 145].
Sorocha nadiae (Martínez) [**revised combination**].

#### Distribution.

ECUADOR: Pichincha (Martínez 1978, Paucar-Cabrera 2005, Soula 2006, Krajcik 2008).

#### Types.

Holotype specimen of Pelidnota (Odontognathus) nadiae at MACN; 1 ♂ paratype at CMNC.

#### Remarks.

CCECL contains a *S. nadiae* specimen labeled as a female alloréférent with the following data: 1 ♀ alloréférent: “Pacto Pichincha Equa. M. SOULA det 19/*Sorocha nadiae* (Mar.) M. SOULA det [19 crossed out] 2006//Alloreferent ♀ *Sorocha nadiae* det. M. R. Moore 2014 (M)” (47030992). Box 4618688 SOULA. While clarifying the subgeneric classification of *Pelidnota* (due to homonymy of the genus-group name *Odontognathus* Laporte), Özdikmen (2009) listed *Pelidnota nadiae* within Pelidnota (Strigidia). We think that Özdikmen (2009) was unaware of Soula’s (2006) erection of the genus *Sorocha* for some species previously classified in various subgenera of *Pelidnota*. We classify this species in *Sorocha* as *S. nadiae* (Martínez) until the validity of *Sorocha* is evaluated by phylogenetic analysis.

### Sorocha plicipennis

Taxon classificationAnimaliaColeopteraScarabaeidae

(Ohaus, 1934)

Pelidnota (Ganonota) plicipennis Ohaus, 1934a: 10–11 [original combination].
Pelidnota (Strigidia) plicipennis Ohaus [new subgeneric combination by Machatschke 1970: 157].
Pelidnota (Odontognathus) plicipennis Ohaus [new subgeneric combination by Hardy 1975: 4].
Sorocha plicipennis (Ohaus) [new combination by Soula 2006: 92–93].
Pelidnota (Strigidia) plicipennis (Ohaus) [revised combination and revised subgeneric combination by Özdikmen 2009: 145].
Sorocha plicipennis (Ohaus) [**revised combination**].

#### Distribution.

BOLIVIA: La Paz (Ohaus 1934a, b, Blackwelder 1944, Machatschke 1972, Soula 2006, Krajcik 2008).

#### Types.

**Figure 102.**
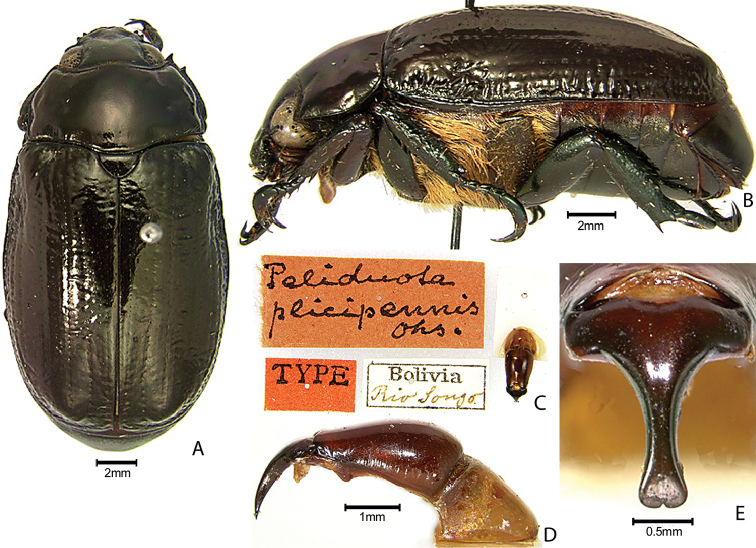
Pelidnota (Ganonota) plicipennis Ohaus (valid name *Sorocha plicipennis* [Ohaus]) type male from ZMHB. **A** Dorsal habitus **B** Lateral habitus **C** Specimen labels and male genitalia **D** Male genitalia, lateral view **E** Male parameres, caudal view.

1 ♂ type specimen of Pelidnota (Ganonota) plicipennis Ohaus at ZMHB (Fig. 102). Soula (2006) recorded 1 ♂ lectotype and 1 paralectotype at ZMHB.

#### Remarks.

While clarifying the subgeneric classification of *Pelidnota* (due to homonymy of the genus-group name *Odontognathus* Laporte), Özdikmen (2009) listed *Pelidnota plicipennis* within Pelidnota (Strigidia). We think that Özdikmen (2009) was unaware of Soula’s (2006) erection of the genus *Sorocha* for some species previously classified in various subgenera of *Pelidnota*. We classify this species in *Sorocha* as *S. plicipennis* (Ohaus) until the validity of *Sorocha* is evaluated by phylogenetic analysis.

### Sorocha similis

Taxon classificationAnimaliaColeopteraScarabaeidae

(Ohaus, 1908)

Pelidnota similis Ohaus, 1908b: 400–401 [original combination].
Pelidnota (Ganonota) similis Ohaus [new subgeneric combination by Ohaus 1934b: 82].
Pelidnota (Strigidia) similis Ohaus [new subgeneric combination by Machatschke 1970: 157].
Pelidnota (Odontognathus) similis Ohaus [new subgeneric combination by Hardy 1975: 4].
Sorocha similis (Ohaus) [new combination by Soula 2006: 89–90].
Pelidnota (Strigidia) similis Ohaus [revised combination and revised subgeneric combination by Özdikmen 2009: 145].
Sorocha similis (Ohaus) [**revised combination**].

#### Distribution.

ECUADOR: Morona-Santiago, Zamora Chinchipe (1908b, 1918, 1925, 1934b, Blackwelder 1944, Machatschke 1972, Paucar-Cabrera 2005, Soula 2006, Krajcik 2008). PERU: Amazonas (Soula 2006, Ratcliffe et al. 2015).

#### Types.

**Figure 103.**
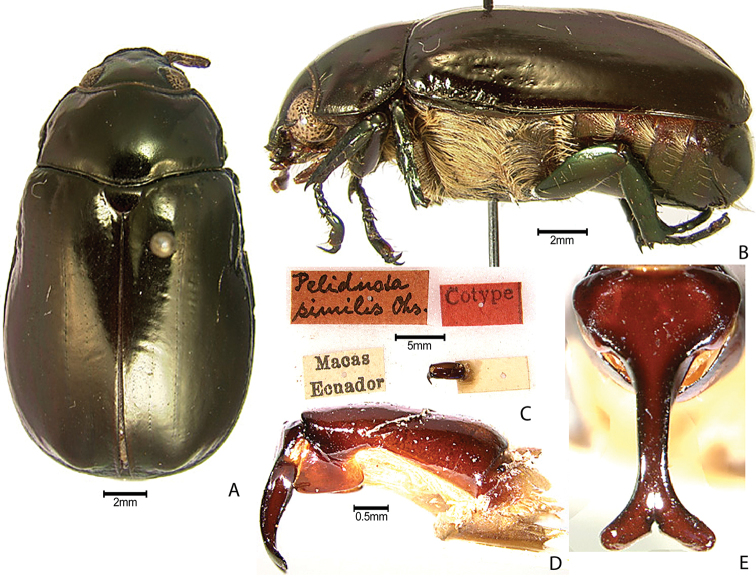
*Pelidnota similis* Ohaus (valid name *Sorocha similis* [Ohaus]) male specimen (possibly invalid type) from ZMHB. **A** Dorsal habitus **B** Lateral habitus **C** Specimen labels and male genitalia **D** Male genitalia, lateral view **E** Male parameres, caudal view.

1 possible ♂ holotype of *Pelidnota similis* Ohaus at ZMHB (Fig. 103). Soula (2006) recorded 1 ♀ holotype, but he did not provide the institutional depository.

#### Remarks.

While clarifying the subgeneric classification of *Pelidnota* (due to homonymy of the genus-group name *Odontognathus* Laporte), Özdikmen (2009) listed *Pelidnota similis* within Pelidnota (Strigidia). We think that Özdikmen (2009) was unaware of Soula’s (2006) erection of the genus *Sorocha* for some species previously classified in various subgenera of *Pelidnota*. We classify this species in *Sorocha* as *S. similis* (Ohaus) until the validity of *Sorocha* is evaluated by phylogenetic analysis.

### Sorocha tolimana

Taxon classificationAnimaliaColeopteraScarabaeidae

(Ohaus, 1935)

Pelidnota tolimana Ohaus, 1935: 121–122 [original combination].
Pelidnota (Pelidnota) tolimana Ohaus [new subgeneric combination by Machatschke 1972: 24].
Sorocha tolimana (Ohaus) [new combination by Soula 2006: 95–96].

#### Distribution.

COLOMBIA: Tolima (Ohaus 1935, Machatschke 1972, Restrepo et al. 2003, Soula 2006, Krajcik 2008).

#### Types.

**Figure 104.**
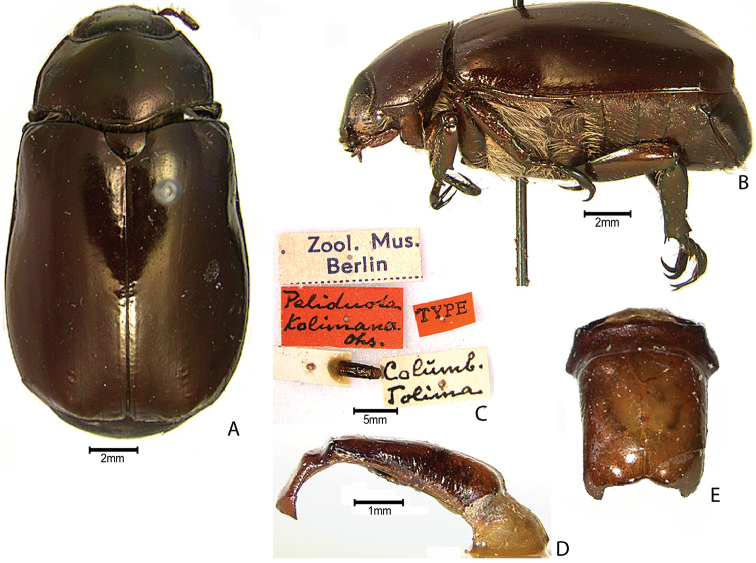
*Pelidnota tolimana* Ohaus (valid name *Sorocha tolimana* [Ohaus]) male syntype from ZMHB. **A** Dorsal habitus **B** Lateral habitus **C** Specimen labels and male genitalia **D** Male genitalia, lateral view **E** Male parameres, caudal view.

1 ♂ syntype specimen of *Pelidnota tolimana* Ohaus at ZMHB (Fig. 104). Soula (2006) recorded 1 ♂ syntype and 1 ♀ syntype, possibly at ZMHB.

### Sorocha touroulti

Taxon classificationAnimaliaColeopteraScarabaeidae

Soula, 2009

Sorocha touroulti Soula, 2009: 135 [original combination].

#### Distribution.

BOLIVIA (Soula 2009).

#### Types.

The following specimen is deposited at CCECL. 1 ♂ holotype: “Rte de Chaparé pk 96. BO. 2000 m M. SOULA det 19 [obverse] 4/10/07//Holotype 2009 *Sorocha touroulti* S. Soula” (47030982). Genitalia card-mounted underneath the male holotype. Box 4618688 SOULA.

### Sorocha yungana

Taxon classificationAnimaliaColeopteraScarabaeidae

(Ohaus, 1934)

Pelidnota (Ganonota) yungana Ohaus, 1934a: 11 [original combination].
Pelidnota (Strigidia) yungana Ohaus [new subgeneric combination by Machatschke 1970: 157].
Pelidnota (Odontognathus) yungana Ohaus [new subgeneric combination by Hardy 1975: 4].
Sorocha yungana (Ohaus) [new combination by Soula 2006: 94–95].
Pelidnota (Strigidia) yungana Ohaus [revised combination and revised subgeneric combination by Özdikmen 2009: 145].
Sorocha yungana (Ohaus) [**revised combination**].

#### Distribution.

BOLIVIA: La Paz (Ohaus 1934a, b, Machatschke 1972, Soula 2006, Krajcik 2008).

#### Types.

**Figure 105.**
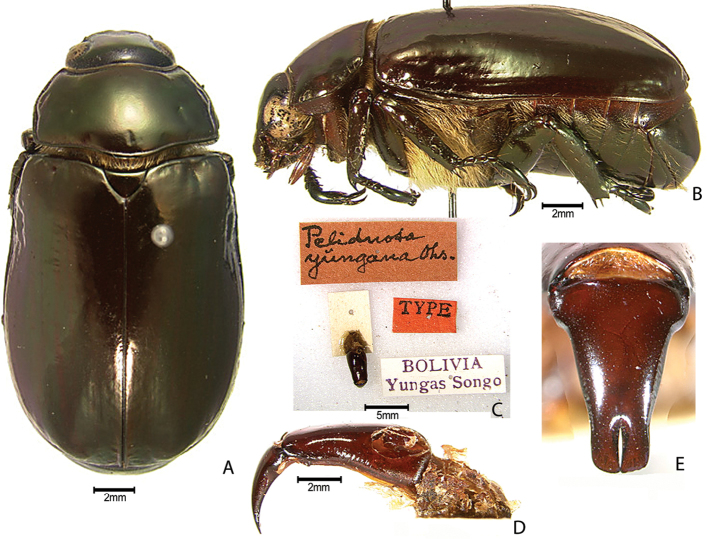
Pelidnota (Ganonota) yungana Ohaus (valid name *Sorocha yungana* [Ohaus]) male holotype from ZMHB. **A** Dorsal habitus **B** Lateral habitus **C** Specimen labels and male genitalia **D** Male genitalia, lateral view **E** Male parameres, caudal view.

Soula (2006) recorded the ♂ holotype of Pelidnota (Ganonota) yungana at ZMHB, so it is possible that Fig. 105 is the holotype specimen.

#### Remarks.

While clarifying the subgeneric classification of *Pelidnota* (due to homonymy of the genus-group name *Odontognathus* Laporte), Özdikmen (2009) listed *Pelidnota yungana* within Pelidnota (Strigidia). We think that Özdikmen (2009) was unaware of Soula’s (2006) erection of the genus *Sorocha* for some species previously classified in various subgenera of *Pelidnota*. We classify this species in *Sorocha* as *S. yungana* (Ohaus) until the validity of *Sorocha* is evaluated by phylogenetic analysis.

## Unavailable, invalid names in *Sorocha*

### Sorocha ferrandi

Taxon classificationAnimaliaColeopteraScarabaeidae

 in litt.; Unavailable, invalid name

#### Types.

The following specimen is deposited at CCECL. 1 invalid ♂ holotype: “Route de Loreto à Coca P.L. pk 11 - Napo. (1450 m) (E) [obverse] 13/8/88 P.W.//Holotype 2006 *Sorocha ferrandi* Sou. Soula//Invalid type not described det. M. R. Moore 2014” (47030983). Genitalia card-mounted underneath the invalid male holotype. Box 4618688 SOULA.

#### Remarks.

We regard the name “*Sorocha ferrandi*” as an unavailable name because it was not associated with a species description and because its continued use may lead to further nomenclatural instability. The male specimen in CCECL is an invalid holotype specimen because the species name is not associated with a species description or type designation in the published literature.

## Unavailable names in *Sorocha* (application of ICZN Article 16.4.2)

We consider the following names proposed by Soula in *Sorocha* as **unavailable** per ICZN Article 16.4.2. which states that fixation of holotype specimens for new names must be accompanied by the following information, “where the holotype or syntypes are extant specimens, by a statement of intent that they will be (or are) deposited in a collection and a statement indicating the name and location of that collection”. The names below were proposed by Soula (2008, 2011), but the descriptions did not state the intent to deposit the holotype specimens in a collection. By applying ICZN Article 16.4.2 herein, the following names are **unavailable**: *Sorocha carloti* Demez and Soula 2011, *Sorocha castroi* Soula 2008, *Sorocha fravali* Soula 2011, *Sorocha jeanmaurettei* Demez and Soula 2011, *Sorocha yelamosi* Soula 2011. Below we report the complete taxonomic history of these names and the data from their invalid type specimens that are deposited at CCECL.

### Sorocha carloti

Taxon classificationAnimaliaColeopteraScarabaeidae

Demez & Soula, 2011 Unavailable, invalid name

Sorocha carloti Demez & Soula, 2011: 79 [original combination, **unavailable, invalid name**].

#### Distribution.

PERU: Pasco (Soula 2011, Ratcliffe et al. 2015).

#### Types.

The following invalid type specimens are deposited at CCECL. 1 invalid ♂ holotype, 1 invalid ♀ allotype: “Oxapampa Pérou I/2011 M. SOULA det 19//Holotype 2011 *Sorocha carloti* S. Soula det.” (47030986); “Oxapampa Pérou I/2011 M. SOULA det 19//Allotype 2011 *Sorocha carloti* S. Soula det.” (47030987). Genitalia card-mounted underneath the invalid male holotype. Box 4618688 SOULA.

### Sorocha castroi

Taxon classificationAnimaliaColeopteraScarabaeidae

Soula, 2008 Unavailable, invalid name

Sorocha castroi Soula, 2008: 35–36 [original combination, **unavailable, invalid name**].

#### Distribution.

PERU: San Martin (Soula 2008, Ratcliffe et al. 2015).

#### Types.

The following invalid type specimen is deposited at CCECL. 1 invalid ♂ holotype: “San Martin Pérou 10/2006 M. SOULA det 19//Holotype *Sorocha castroi* S. Soula det. 2007” (47030984). Genitalia card-mounted underneath the invalid male holotype. Box 4618688 SOULA.

### Sorocha fravali

Taxon classificationAnimaliaColeopteraScarabaeidae

Soula, 2011 Unavailable, invalid name

Sorocha fravali Soula, 2011: 80–81 [original combination, **unavailable, invalid name**].

#### Distribution.

VENEZUELA: Yaracuy (Soula 2011).

#### Types.

The following invalid type specimen is deposited at CCECL. 1 invalid ♂ holotype: “VENEZUELA Yaracuy Via Cocorote - El Candelo 10,36889°N - 68,82689°W 1650m 15-21-x-2001//R. Briceño; J. Clavijo; L.J. Joly; F. Díaz; E. Arcaya; R. Paz Proyecto S1-2000000479//Holotype 2011 *Sorocha fravali* S. Soula” (47030963). Genitalia card-mounted underneath the invalid male holotype. Box 4618687 SOULA.

### Sorocha jeanmaurettei

Taxon classificationAnimaliaColeopteraScarabaeidae

Demez & Soula, 2011 Unavailable, invalid name

Sorocha jeanmaurettei Demez & Soula, 2011: 81 [original combination, **unavailable, invalid name**].

#### Distribution.

PERU: Cusco (Soula 2011, Ratcliffe et al. 2015).

#### Types.

The following invalid type specimens are deposited at CCECL. 1 invalid ♂ holotype, 1 invalid ♀ allotype, 1 invalid ♂ paratype, 1 invalid ♀ paratype: “Valle Cosnipata 2600 m Paucartambo M. SOULA det 19 [obverse] Cusco III/2011//Holotype 2011 *Sorocha jeanmaurettei* D. et S. Soula” (47030988); “Valle Cosnipa Paucartambo 2800 m M. Soula det. 20 [obverse] III/2011 Cusco//Allotype 2011 *Sorocha jeanmaurettei* D. et S. Soula” (47030989); “Valle Cosnipa Paucartambo 2800 m M. Soula det. 20 [obverse] III/2011//Paratype *Sorocha jeanmaurettei* D. et S. Soula” (47030990); “Valle Cosnipa Paucartambo 2800 m M. Soula det. 20 [obverse] III/2011 Cusco//Paratype 2011 *Sorocha jeanmaurettei* D. et S. Soula” (47030991). Genitalia card-mounted underneath the invalid male holotype and the invalid male paratype. Box 4618688 SOULA.

### Sorocha yelamosi

Taxon classificationAnimaliaColeopteraScarabaeidae

Soula, 2011 Unavailable, invalid name

Sorocha yelamosi Soula, 2011: 82 [original combination, **unavailable, invalid name**].

#### Distribution.

PERU: Junín (Soula 2011, Ratcliffe et al. 2015).

#### Types.

The following invalid type specimen is deposited at CCECL. 1 invalid ♂ holotype: “Satipo Pérou IX/2003 M. SOULA det 19//Holotype 2011 *Sorocha yelamosi* S. Soula det.” (47030985). Genitalia card-mounted underneath the invalid male holotype. Box 4618688 SOULA.

### 

Taxon classificationAnimaliaColeopteraScarabaeidae

F. Bates, 1904

Xenopelidnota F. Bates, 1904: 253, 275–276.

#### Type species.

*Plusiotis anomala* Burmeister, 1844: 422-423, subsequent designation (monotypy) by F. Bates 1904: 275–276.

#### Gender.

Feminine.

#### Species.

3 species and subspecies.

### Xenopelidnota anomala anomala

Taxon classificationAnimaliaColeopteraScarabaeidae

(Burmeister, 1844)

Plusiotis anomala Burmeister, 1844: 422–423 [original combination].
Pelidnota anomala (Burmeister) [new combination by Blanchard 1851: 211].
Xenopelidnota anomala (Burmeister) [new combination by F. Bates 1904: 253, 275–276].

#### Distribution.

BOLIVIA (Chalumeau 1985, Peck 2010). COLOMBIA: Atlántico (Burmeister 1844, Harold 1869b, Ohaus 1918, 1934b, Blackwelder 1944, Gutiérrez 1951, Machatschke 1972, Chalumeau 1985, Restrepo et al. 2003, Krajcik 2008, Soula 2009, Peck 2010, García-Atencia and Martínez-Hernández 2015, García-Atencia et al. 2015). TRINIDAD AND TOBAGO: Trinidad (Chalumeau 1985, Peck 2010). VENEZUELA (Cerro del Naiguatá) (Ohaus 1918, 1934b, Gutiérrez 1951, Chalumeau 1985, Peck 2010).

#### Types.

1 ♂ neotype of *Plusiotis anomala* at MNHN (Soula 2009).

#### Remarks.

CCECL contains a *X. anomala* specimen labeled as a female alloréférent with the following data: 1 ♀ alloréférent: “Colüm-bia//Alloréferent ♀ de *Plusiotis anomala* Burm M. SOULA det 19” (47030552). Box 4618669 SOULA.

### Xenopelidnota anomala porioni

Taxon classificationAnimaliaColeopteraScarabaeidae

Chalumeau, 1985

Xenopelidnota anomala porioni Chalumeau, 1985: 236–237 [original combination].
Xenopelidnota pittieri porioni Chalumeau [new combination by Soula 2009: 127].
Xenopelidnota anomala porioni Chalumeau [**revised subspecific status**].

#### Distribution.

GRENADA (FSCA) (Peck 2016). ST. VINCENT AND THE GRENADINES: St. Vincent (Chalumeau 1985, Krajcik 2008, Soula 2009, Peck 2010).

#### Types.

**Figure 106.**
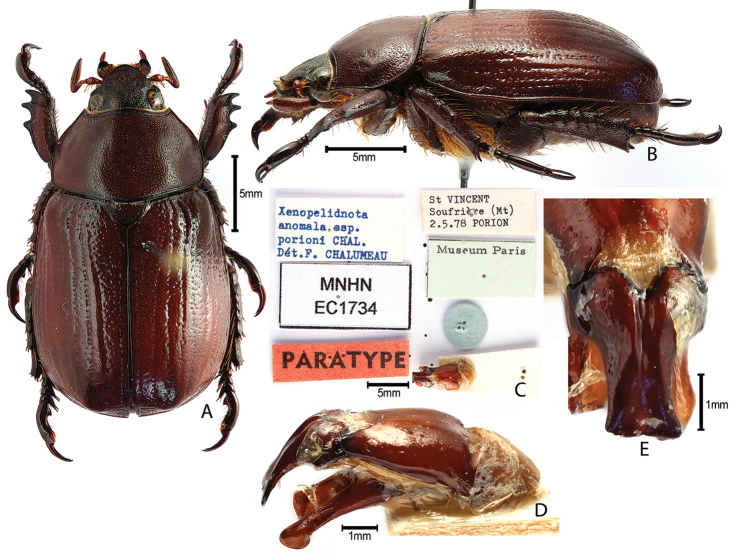
*Xenopelidnota anomala porioni* Chalumeau male paratype from MNHN. **A** Dorsal habitus **B** Lateral habitus **C** Specimen labels and male genitalia **D** Male genitalia, lateral view **E** Male parameres, caudal view.

1 ♂ holotype at IREC. Paratypes at IREC, MNHN (Fig. 106), USNM, BMNH, and CAS (Alan Hardy’s Collection) (Chalumeau 1985, Soula 2009). Searching for the paratype at BMNH did not reveal the specimen (BHG pers. obs. Aug. 2016).

#### Remarks.

Soula (2009) considered *Xenopelidnota anomala porioni* to be a subspecies of his new species *X. pittieri*. However, *Xenopelidnota pittieri pittieri* Soula is an **unavailable name** (see section below). We correct this herein and consider the valid name to be *Xenopelidnota anomala porioni* Chalumeau, **revised subspecific status**. Future research should examine the status of this subspecies as it is distinctive and may be more appropriately treated as a species.

### Xenopelidnota fuscoaenea

Taxon classificationAnimaliaColeopteraScarabaeidae

(Blanchard, 1851)

Pelidnota fuscoaenea Blanchard, 1851: 211 [original combination].
Xenopelidnota anomala (Burmeister) [syn. by Ohaus 1918: 15].
Xenopelidnota fuscoaenea (Blanchard) [revised species status by Soula 2009: 125–126].

#### Distribution.

COLOMBIA (Soula 2009). VENEZUELA (Blackwelder 1944, Soula 2009).

#### Types.

**Figure 107.**
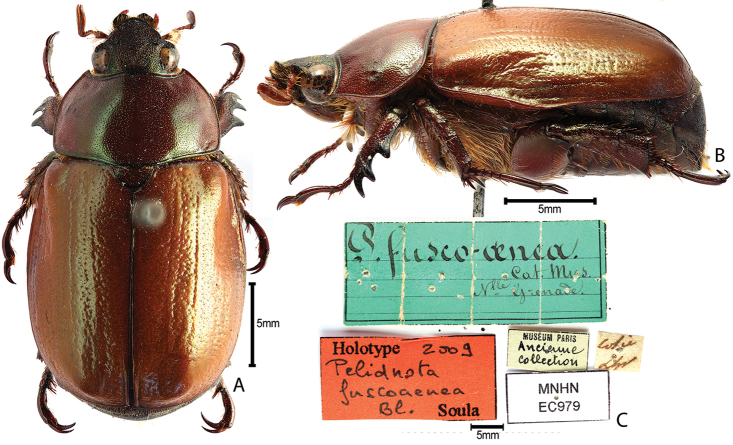
*Pelidnota fuscoaenea* Blanchard (valid name *Xenopelidnota fuscoaenea* [Blanchard]) female syntype from MNHN. **A** Dorsal habitus **B** Lateral habitus **C** Specimen labels.

1 ♀ syntype of *Pelidnota fuscoaenea* at MNHN (Soula 2009) (Fig. 107).

#### Remarks.

Specific locality data reported for this species in the literature is highly uncertain. Per Blanchard (1851) the specimen was from “Nouv.-Grenade, rives de l’Oyapok”.

## Unavailable names in *Xenopelidnota* (application of ICZN Article 16.4.2)

We consider the following names proposed by Soula in *Xenopelidnota* as **unavailable** per ICZN Article 16.4.2 which states that fixation of holotype specimens for new names must be accompanied by the following information, “where the holotype or syntypes are extant specimens, by a statement of intent that they will be (or are) deposited in a collection and a statement indicating the name and location of that collection”. The names below were proposed by Soula (2009), but the descriptions did not state the intent to deposit the holotype specimens in a collection. By applying ICZN Article 16.4.2 herein, the following names are **unavailable**: *Xenopelidnota bolivari* Soula 2009 and *Xenopelidnota pittieri pittieri* Soula 2009. Below we report the complete taxonomic history of these names and the data from their invalid type specimens that are deposited at CCECL.

### Xenopelidnota bolivari

Taxon classificationAnimaliaColeopteraScarabaeidae

Soula, 2009 Unavailable, invalid name

Xenopelidnota bolivari Soula, 2009: 125 [original combination, **unavailable, invalid name**].

#### Distribution.

VENEZUELA: Bolívar (Soula 2009).

#### Types.

The following invalid type specimens are deposited at CCECL. 1 invalid ♂ holotype, 1 invalid ♀ allotype: “Jabillal Bolivar Venez. 7/89 coll. – SOULA//Holotype 2009 *Xenopelidnota bolivari* S. Soula” (47030553); “Jabillal Bolivar Venez. 7/89 M. SOULA det. 19//Allotype 2009 *Xenopelidnota bolivari* S. Soula” (47030554). Box 4618669 SOULA.

### Xenopelidnota pittieri pittieri

Taxon classificationAnimaliaColeopteraScarabaeidae

Soula, 2009 Unavailable, invalid name

Xenopelidnota pittieri pittieri Soula, 2009: 126–127 [original combination, **unavailable, invalid name**].

#### Distribution.

VENEZUELA: Aragua, Distrito Federal (Soula 2009).

#### Types.

The following invalid type specimens are deposited at CCECL. 1 invalid ♂ holotype, 1 invalid ♀ allotype, 2 invalid ♂ paratypes, 5 invalid ♀ paratypes: “P. N. Henri Pittier Choroni, Venezuela V-VI/2005//Holotype 2009 *Xenopelidnota pittieri* S. Soula” (47030555); “P. N. Henri Pittier Choroni, Venezuela V-VI/2005//Allotype 2009 *Xenopelidnota pittieri* S. Soula” (47030556); Five paratypes with identical label data “P. N. Henri Pittier Choroni, Venezuela V-VI/2005//Paratype 2009 *Xenopelidnota pittieri* S. Soula” (47030557 to 47030561); “Caracas M. SOULA det. 19//Paratype 2009 *Xenopelidnota pittieri* S. Soula” (47030562); “Guiana M. SOULA det. 19//Paratype 2009 *Xenopelidnota pittieri* S. Soula” (47030563). Genitalia card-mounted underneath the invalid male holotype, invalid female allotype, and two invalid male paratypes. Box 4618669 SOULA.

#### Remarks.

Soula (2009) described *Xenopelidnota pittieri* and revised the status of *Xenopelidnota anomala porioni* Chalumeau and treated it as a subspecies of his new taxon (*X. pittieri pittieri* Soula and *X. pittieri porioni* Chalumeau). Soula (2009) overlooked the priority of the subspecific name Chalumeau (1985) established.
